# The acquisition of novel N-glycosylation sites in conserved proteins during human evolution

**DOI:** 10.1186/s12859-015-0468-5

**Published:** 2015-01-28

**Authors:** Dong Seon Kim, Yoonsoo Hahn

**Affiliations:** Department of Life Science, Research Center for Biomolecules and Biosystems, Chung-Ang University, 84 Heukseok-ro, Dongjak-gu, Seoul 156-756 Korea

**Keywords:** N-glycosylation, Evolution, Glycoproteome, Human

## Abstract

**Background:**

N-linked protein glycosylation plays an important role in various biological processes, including protein folding and trafficking, and cell adhesion and signaling. The acquisition of a novel N-glycosylation site may have significant effect on protein structure and function, and therefore, on the phenotype.

**Results:**

We analyzed the human glycoproteome data set (2,534 N-glycosylation sites in 1,027 proteins) and identified 112 novel N-glycosylation sites in 91 proteins that arose in the human lineage since the last common ancestor of Euarchonta (primates and treeshrews). Three of them, Asn-196 in adipocyte plasma membrane-associated protein (APMAP), Asn-91 in cluster of differentiation 166 (CD166/ALCAM), and Asn-76 in thyroglobulin, are human-specific. Molecular evolutionary analysis suggested that these sites were under positive selection during human evolution. Notably, the Asn-76 of thyroglobulin might be involved in the increased production of thyroid hormones in humans, especially thyroxine (T4), because the removal of the glycan moiety from this site was reported to result in a significant decrease in T4 production.

**Conclusions:**

We propose that the novel N-glycosylation sites described in this study may be useful candidates for functional analyses to identify innovative genetic modifications for beneficial phenotypes acquired in the human lineage.

**Electronic supplementary material:**

The online version of this article (doi:10.1186/s12859-015-0468-5) contains supplementary material, which is available to authorized users.

## Background

N-linked glycosylation of the Asn residue in the consensus motif Asn-X-Ser/Thr, where X is any amino acid except Pro, is one of the most well-studied protein posttranslational modifications (PTMs) [1]. N-glycosylation, which mainly occurs in secreted or cell membrane proteins, plays important roles in protein folding, quality control, and trafficking [2], as well as cell adhesion and signalling [3,4]. The emergence of a new N-glycosylation site may alter protein function either positively or negatively. For example, missense mutations in factor VIII created novel N-glycosylation sites that cause severe hemophilia A [5]. Similarly, a missense mutation in the interferon γ receptor 2 induces novel N-glycosylation, which results in a Mendelian susceptibility to mycobacterial disease [6,7]. The abolishment of N-glycosylation sites often causes disrupted protein folding, trafficking, or activity; thus, proper N-glycosylation is crucial for normal protein function [8,9]. A proteome-wide analysis of nonsynonymous single-nucleotide variations in the N-glycosylation motifs of human genes showed that more than 1,000 human proteins had either lost or gained N-glycosylation sites due to missense substitutions, some of which may be implicated in diseases [10].

The gain of new N-glycosylation sites during evolution may affect the structure and molecular function of proteins; when these novel modifications confer beneficial traits, they will be fixed during evolution. Previously, we identified a large variety of genetic changes that could have been involved in the acquisition of human traits, including gene inactivation [11,12], exon evolution [13,14], and gains of phosphorylation or ubiquitylation [15,16]. Therefore, it would be of great interest to collect information on novel N-glycosylation sites that arose during human evolution, as the sites might have been involved in the development of some human phenotypes.

In order to study associations between the acquisition of an N-glycosylation site and its phenotypic outcome, a large amount of N-glycosylation site data and mammalian orthologous protein sequence data are required. Recent developments and advances in various high-throughput proteomics techniques for N-glycoproteome identification using immunoaffinity subtraction, hydrazide chemistry, and mass spectrometry have made it possible to access massive amounts of N-glycosylation site data from human proteomes [17-19]. These data are available at the UniProt database (http://www.uniprot.org), which is a universal protein sequence database, as well as some specialized PTM databases such as PHOSIDA (http://www.phosida.com/) [20].

Since human genome sequences were completed [21,22], a large amount of nucleotide and protein sequence data have become available not only from humans but also from many other organisms. Comparative sequence data, including alignments of mammalian orthologous protein sequences, are available at the University of California Santa Cruz (UCSC) Genome Browser Database (http://genome.ucsc.edu) [23].

In this study, a bioinformatics method was devised to identify novel N-glycosylated Asn residues that are located in the consensus motif Asn-X-Ser/Thr and arose during human evolution after the Euarchonta lineage diverged from the Glires lineage. Both a comprehensive literature survey and extensive data mining were conducted to examine the possible functional implications of novel N-glycosylation sites, especially in cases of human-specific gains.

## Results

### Identification of novel N-glycosylation sites acquired during human evolution and determination of the timing of acquisition

**Figure 1:**
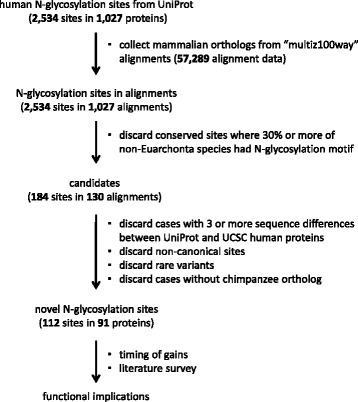
**Overall procedure for identifying gains of novel N-glycosylation sites during human evolution.** Computational screening and manual inspection were employed to identify the acquisition of novel N-glycosylation sites in human proteins during human evolution.

**Figure 2:**
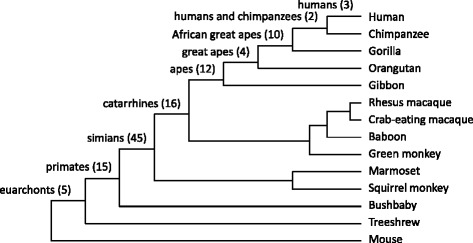
**Timing of acquisition and numbers of novel N-glycosylation sites in the human lineage.** Numbers of novel N-glycosylation sites acquired in the human lineage of the mammalian phylogenetic tree are shown. The number of sites acquired is shown on each branch where the N-glycosylation site consensus motif emerged in the ancestor of the corresponding clade.

We developed a bioinformatics procedure to identify the acquisition by proteins of the N-glycosylation motif Asn-X-Ser/Thr, where the Asn residue was experimentally verified to be N-glycosylated, during human evolution (Figure 1). A novel N-glycosylation site can arise by the emergence of not only an Asn residue but also a Ser or Thr residue to form the consensus motif [24]. The overall procedure devised in this study is similar to that used to identify novel ubiquitylation sites in a previous study [16]. Initially, there were 2,534 experimentally verified human N-glycosylation sites from 1,027 proteins in the UniProt database, and 57,289 orthologous protein sequence alignments from 62 mammalian species, including species from Euarchonta, Glires, Laurasiatheria, Afrotheria, Xenarthra, Marsupialia, and Monotremata, extracted from the UCSC “multiz100way” data [25] (see Additional file 1 for the list of mammalian species). These data were analyzed to collect N-glycosylation sites in human proteins that newly appeared during the evolution from the common ancestor, Euarchonta (primates and treeshrews); as the result, 112 novel N-glycosylation sites from 91 proteins were identified. A summary of the results are presented in Additional file 2, and detailed alignments are provided in Additional file 3. Of the 91 proteins, one protein (CFH) had acquired four N-glycosylation sites (Nos. 28 to 31 in Additional file 2 and Additional file 3; two proteins (PTPRC and PTPRJ) had acquired three sites each (Nos. 85 to 87, and 88 to 90, respectively); 14 proteins had acquired two sites each; and the remaining 74 proteins had acquired one site each. Figure 2 shows the number of the N-glycosylation sites that are shared by each of the Euarchonta clades along the human lineage: humans, three; ancestor of humans and chimpanzees, two; African great apes, 10; great apes, four; apes, 12; catarrhines, 16; simians, 45; primates, 15; and euarchonts, 5.

Most of the novel N-glycosylation sites were generated by the emergence of an Asn residue in an existing X-X-Ser/Thr motif. However, in some cases, the emergence of a Ser or a Thr residue in an Asn-X-X sequence created a novel N-glycosylation site: for example, a change from Asn-Glu-Ile to Asn-Glu-Thr generated a novel N-glycosylation site at Asn-911 in complement factor H (CFH) in apes (No. 30 in Additional file 3).

**Figure 3:**
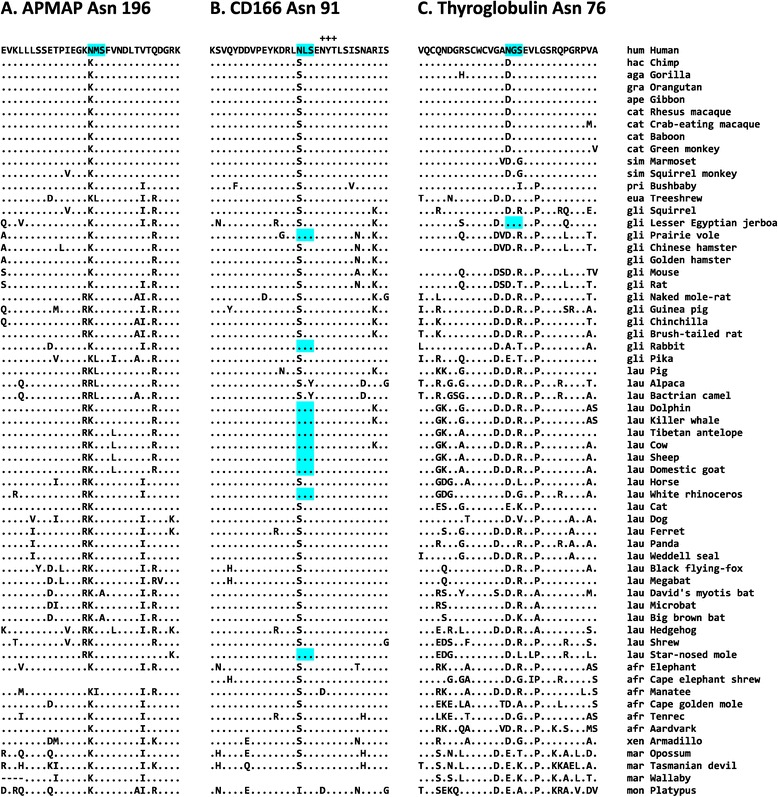
**Multiple sequence alignments of human-specific N-glycosylation sites.** The human-specific N-glycosylation modification sites and the surrounding regions for APMAP **(A)**, CD166 **(B)**, and thyroglobulin **(C)** proteins are presented. The N-glycosylation consensus sequences are highlighted in cyan. An adjacent N-glycosylation site (Asn-95) that is found in CD166 and is well conserved among mammals is indicated by plus signs (+++). The residues that are identical to those in the human sequence are indicated by dots (.). Dashes (−) denote alignment gaps. In some species, sequences were not determined. hum, humans; hac, humans and chimpanzees; aga, African great apes; gra, great apes; ape, apes; cat, catarrhines; sim, simians; pri, primates; eua, Euarchonta; gli, Glires; lau, Laurasiatheria; afr, Afrotheria; xen, Xenarthra; mar, Marsupialia; and mon, Monotremata.

Of the 112 novel N-glycosylation sites, three sites in three proteins were human specific (Table 1 and Figure 3); therefore, these Asn residues subject to the N-glycosylation evolved and were fixed in human proteins after the divergence of humans and chimpanzees. The residues are Asn-196 in adipocyte plasma membrane-associated protein (APMAP), Asn-91 in cluster of differentiation 166 (CD166), and Asn-76 in thyroglobulin.

**Table 1:** **Proteins with human-specific N-glycosylation sites**

**No** ^**a**^	**Gene**	**UniProt ID**	**Position**	**Sequence** ^**b**^	**Protein**
5	*ALCAM*	CD166_HUMAN	91	DDVPEYKDRL**NLS**ENYTLSI	CD166 antigen
8	*APMAP*	APMAP_HUMAN	196	LSSETPIEGK**NMS**FVNDLTV	Adipocyte plasma membrane-associated protein
105	*TG*	THYG_HUMAN	76	DGRSCWCVGA**NGS**EVLGSRQ	Thyroglobulin

^a^The number corresponds to that in Additional files 1 and 2.

^b^The N-glycosylation motif is in bold.

### Human-specific N-glycosylation site Asn-196 in APMAP

The human APMAP (also known as C20orf3) has two N-glycosylation sites: Asn-160 and Asn-196, the latter of which is human specific: almost all the other mammals examined have a Lys residue at this position (Figure 3A). Full-length protein and coding sequences of APMAP orthologs were determined from human, chimpanzee, gorilla, orangutan, gibbon, and rhesus macaque genomes (see Methods section for details). Multiple alignment of these proteins showed that human APMAP protein has two human-specific amino acid changes, Val-100 and Asn-196, where all the other five primates have Ile and Lys, respectively (Additional file 4). The Asn-196 is a human-specific N-glycosylation site.

To test if the APMAP protein has been under positive selection during human evolution, the ratios of nonsynonymous to synonymous rates (dN/dS, ω) across different branches and sites of the selected primate phylogeny were estimated [26-28]. First, we used “branch models”, M0 (one ω ratio for all branches), free ratio (one ω ratio for each branch), and two ratio (ω_1_ for the human branch and ω_0_ for other branches) models (Table 2 and Additional file 4). The likelihood ratio test (LRT) comparing M0 (one ratio) and free ratio model was not significant. However, the LRT comparing M0 and two ratio model was highly significant (P = 0.006943), suggesting the human APMAP has evolved at different rate compared to other primates. The estimated dN and dS rates for the human branch using two ratio model were 0.0024 and 0.0000 (see Additional file 4 for details), respectively, indicating possible accelerated nonsynonymous substitution during human evolution. Next, we used “branch-site models”, model A (ω ratio is left to vary) and null model A (ω ratio is fixed to 1), to infer positively selected sites in human APMAP. The two aforementioned human-specific amino acid positions, Val-100 and Asn-196, were detected to be under positive selection with overall probability of 0.828 and 0.953, respectively, using the Bayes empirical Bayes (BEB) test [29]. However, the LRT comparing model A and null model A was not significant. Although it is statistically insignificant, the acquisition of Asn-196 and its subsequent N-glycosylation might have a significant effect on the structure and function of APMAP in humans.

**Table 2:** **Molecular evolutionary analysis of APMAP, CD166, and thyroglobulin**

**Protein**		**Model**	**ln L**	**ω**	**2Δl**	**P value**	**Positively selected sites** ^**a**^
APMAP	Branch models	M0 (one ratio)	−2042.0968	ω_0_ = 0.07385			
		Free ratio	−2035.7572	See Additional file 4	(M0 vs Free ratio) 12.6792	0.1234	
		Two ratio	−2038.4530	ω_0_ = 0.05733, ω_1_ = 999.000	(M0 vs Two ratio) 7.2876	0.006943**	
	Branch-site models	Model A	−2038.4530	ω_0_ = 0.05732, ω_1_ = 1, ω_2_ = 999.000			Val-100, **Asn-196**
		Null model A	−2039.0253	ω_0_ = 0.05746, ω_1_ = 1, ω_2_ = 1	(Model A vs Null model A) 1.1446	0.2847	
CD166	Branch models	M0 (one ratio)	−2713.8934	ω_0_ = 0.09913			
		Free ratio	−2709.9951	See Additional file 5	(M0 vs Free ratio) 7.7966	0.4536	
		Two ratio	−2713.8904	ω_0_ = 0.09991, ω_1_ = 0.09134	(M0 vs Two ratio) 0.0060	0.9383	
	Branch-site models	Model A	−2713.8934	ω_0_ = 0.09913, ω_1_ = 1, ω_2_ = 1			**Asn-91**
		Null model A	−2713.8934	ω_0_ = 0.09913, ω_1_ = 1, ω_2_ = 1	(Model A vs Null model A) 0.000	1.000	
Thyroglobulin	Branch models	M0 (one ratio)	−15128.0999	ω_0_ = 0.35639			
		Free ratio	−15121.3529	See Additional file 6	(M0 vs Free ratio) 13.4941	0.09595	
		Two ratio	−15124.3983	ω_0_ = 0.33454, ω_1_ = 0.78473	(M0 vs Two ratio) 7.4033	0.006511**	
	Branch-site models	Model A	−15103.5548	ω_0_ = 0.000, ω_1_ = 1, ω_2_ = 3.59382			**Asn-76**, Ser-633, Ser-734, Asn-775, Met-911, Ser-913, Gly-1061, Ser-1140, Thr-1204, Met-1242, Thr-1498, Arg-1646, His-1669, Arg-1691, Asp-1795, His-2486, Arg-2530, Asn-2616, Leu-2632, Glu-2702, Thr-2727, Thr-2765
		Null model A	−15104.1308	ω_0_ = 0.000, ω_1_ = 1, ω_2_ = 1	(Model A vs Null model A) 1.1520	0.2831	

^a^Human-specific N-glycosylation sites are in bold. See Additional files 4, 5, and 6 for details.

**P < 0.05.

APMAP is an adipocyte plasma membrane-associated protein, which is induced during adipocyte differentiation [30]. It is ubiquitously expressed in human embryonic and adult tissues, with the highest levels in liver, placenta, and kidney [31]. APMAP may exhibit calcium-dependent hydrolase activity and is regulated by the peroxisome proliferator activated receptor γ protein that is a master regulator of adipocyte differentiation [32]. Expression of APMAP was reported to be strongly correlated with hepatic-specific metastasis in patients with metastatic colorectal cancer [33]. A recent study demonstrated that APMAP is a negative regulator of amyloid-beta (Aβ) production through its interaction with amyloid precursor protein and γ-secretase [34]. Although APMAP seems to be involved in various biological processes in humans, the molecular function directly associated with the human-specific N-glycosylation site in APMAP is yet to be determined.

### Human-specific N-glycosylation site Asn-91 in CD166

The human CD166, which is also known as activated leukocyte cell adhesion molecule (ALCAM), has 10 N-glycosylation sites. The residue Asn-91 is found in humans but not in other euarchonts; thus, this residue evolved after the divergence of humans and chimpanzees (Figure 3B). Most other mammals have a Ser residue at this position. However, some Glires and Laurasiatheria species, especially whales and ruminants, independently acquired a consensus sequence for N-glycosylation at this position.

Multiple alignment of full-length CD166 proteins from human, chimpanzee, gorilla, orangutan, gibbon, and rhesus macaque genomes revealed that the Asn-91 is only residue that differs between humans and chimpanzees (Additional file 5). Although the Asn-91 is human-specific, all the LRTs (M0 versus free ratio model, M0 versus two ratio model, and model A versus null model A) were insignificant (Table 2 and Additional file 5), implying that there has been no statistically noticeable positive selection on the human CD166. Interestingly, the human-specific N-glycosylation site Asn-91 was still inferred to be under positive selection with overall probability of 0.613, suggesting the acquisition of this site and its N-glycosylation might have an effect on the function of human CD166 proteins.

CD166 binds to the T-cell differentiation antigen CD6 and may play a role in the binding of T and B cells to activated leukocytes, as well as in interactions between cells of the nervous system [35]. CD166 is composed of five extracellular immunoglobulin (Ig)-like domains: two Ig-like V-type domains and three Ig-like C2-type domains. The human-specific N-glycosylation site Asn-91 is located in the first Ig-like V-type domain, which mediates the CD166–CD6 interaction [35-37]. Most functional studies on CD166 have focused on its cancer-related functions such as invasion, migration, and adhesion [37,38]. However, recent studies show that CD166 is also involved in axon growth in neuronal cells such as retinal ganglion cells and dorsal root ganglion cells [39,40]. Therefore, it is possible that the gain of the Asn-91 N-glycosylation site in CD166 might be involved in the evolution of novel phenotypes in nervous system development, as well as in immune response and cell adhesion processes, which must be validated experimentally.

### Human-specific N-glycosylation site Asn-76 in thyroglobulin

Thyroglobulin is the precursor of the thyroid hormones T4 and triiodothyronine (T3), both of which regulate metabolism in humans [41-43]. The human thyroglobulin has 17 N-glycosylation sites; Asn-76, which becomes Asn-57 in the mature form of thyroglobulin, is a human-specific N-glycosylation site: most other mammals have Asp or Glu at this position (Figure 3C). Interestingly, multiple alignment of thyroglobulin orthologs from humans, chimpanzees, gorillas, orangutans, gibbons, and rhesus macaques revealed additional 20 amino acid positions with human-specific substitution (Additional file 6).

To test if the thyroglobulin has been under positive selection during human evolution, ω ratios across different branches and sites of the selected primate phylogeny were estimated (Table 2 and Additional file 6). The LRT comparing M0 and free ratio model was not significant. However, the LRT comparing M0 and two ratio model was highly significant (P = 0.006511), implying the human thyroglobulin has evolved at different rate compared to other primates. The estimated ω ratio for the human branch (ω_1_) using two ratio model was 0.78473, while ω_0_ for other branches was 0.33454 (see Additional file 6 for details), suggesting a slightly accelerated nonsynonymous substitution during human evolution.

Inference of positively selected sites in human thyroglobulin using model A showed that the 21 aforementioned amino acid positions might have been under positive selection although the LRT comparing model A and null model A was not significant. In spite of statistical insignificance, the acquisition of novel N-glycosylation site Asn-76, together with other 20 human-specific amino acid substitutions, might have a significant effect on the thyroid hormone metabolism in humans.

The thyroglobulin protein precursor itself has no biological function but serves as a chemical platform for thyroid hormone production. When the two N-glycosylation sites in its N-terminal region, including the human-specific Asn-76, were deglycosylated by peptide-N^4^-(N-acetyl-β-glucosaminyl)-asparagine amidase, T4 production decreased by half compared to that seen with the normal protein [44]. Therefore, proper N-glycosylation modifications, including those at the human-specific site, in thyroglobulin are crucial for normal T4 production and the control of metabolism. It has been suggested that humans and chimpanzees differ with respect to their thyroid hormone metabolism [45]. Interestingly, compared to chimpanzees, humans have a higher T4 plasma concentration, which may be implicated in the origins of human intelligence [46].

### Novel N-glycosylation sites shared by other animals

**Figure 4:**
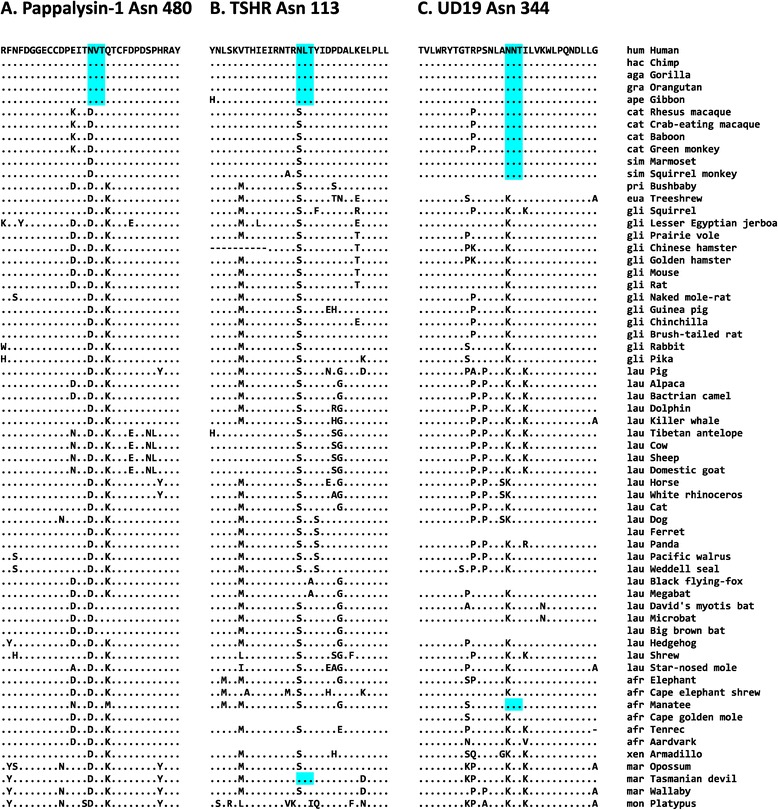
**Multiple sequence alignments of N-glycosylation sites that arose during human evolution.** The N-glycosylation sites and the surrounding regions for pappalysin-1 **(A)**, TSHR **(B)**, and UD19 **(C)** proteins are presented. See Figure 3 for further details.

Of the 112 novel N-glycosylation sites in human proteins, 109 sites were shared by other animals. For example, 12 N-glycosylation sites were shared by all apes, indicating that this site appeared in the common ancestor of apes. The Asn-480 of pappalysin-1 (also known as pregnancy-associated plasma protein-A), which is N-glycosylated in humans [47], is shared by all apes examined (Figure 4A); in contrast, all the other mammals, even Marsupialia and Monotremata species, have an Asp residue at this position. Pappalysin-1 has metalloproteinase activity and specifically cleaves insulin-like growth factor-binding proteins [48,49]; it is present at high concentration in maternal blood during pregnancy and is essential for normal fetal development [50]. The serum pappalysin-1 concentration frequently increases in patients with severe sepsis and appears to be associated with sepsis-related myocardial dysfunction [51]. However, there is no comprehensive study of whether the gain of Asn-480 in apes is associated with these phenotypes.

There are 12 N-glycosylation sites that might have arisen in the common ancestor of apes. One is Asn-113 in the thyrotropin receptor, or thyroid-stimulating hormone receptor (TSHR) (Figure 4B). The THSR responds to thyroid-stimulating hormone (also known as thyrotropin) and stimulates the production of T4 and T3 in the thyroid gland [52]. Human THSR has six N-glycosylation sites: Asn-113 is specific to apes, and the other five are conserved in other mammals. However, a mutated TSHR in which N-glycosylation at Asn-113 had been disrupted had the same expression level and function as the wildtype TSHR; thus, Asn-113 N-glycosylation may not be important for TSHR function [53]. Therefore, gain of N-glycosylation at Asn-113 may be neutral or have a function yet to be determined.

The UDP-glucuronosyltransferase 1–9 (UD19) Asn-344 is one of 45 N-glycosylation sites that are shared by simians (apes and monkeys; Figure 4C). Nonsimian mammals have a Lys residue at this position. UD19, which is also known as UDP glucuronosyltransferase 1 family, polypeptide A9, is involved in the conjugation and elimination of toxic xenobiotic and endogenous compounds [54,55]. Unglycosylation of UD19 resulted in the inhibition of proper protein folding and the impairment of glucuronidation activity; thus, N-glycosylation plays a role in folding the human UD19 protein [56]. UD19 is one of nine functional isoforms produced by the alternative utilization of the first nine exons in the *UGT1A* gene locus [57]. Because Asn-344 is located in the common exon 5, not only UD19 but also eight other isoforms of UDP glucuronosyltransferase 1 enzyme have this novel N-glycosylation site [56].

## Discussion

Previously, it has been suggested that the gain of novel protein PTM sites such as ubiquitylation sites may be associated with the acquisition of novel phenotypes during human evolution by modulating the activity or network of proteins [16]. It is also highly probable that gains of novel N-glycosylation sites may result in functional modification of proteins and phenotypic changes in an organism. In this study, 1,027 human glycoproteins containing experimentally verified N-glycosylation sites and their orthologous mammalian proteins were systematically compared. As a result, 112 novel N-glycosylation sites were identified in 91 proteins that newly appeared during human evolution after the Euarchonta lineage diverged from the Glires lineage. It must be noted that most of these novel N-glycosylation sites were obtained by high-throughput mass spectrometry. The presence of these modifications must be further scrutinized by conventional molecular biology techniques.

Not all the novel N-glycosylation sites described in this study may have resulted in functional innovation. Some of them might have appeared as a result of random genetic drift and be functionally neutral. Nevertheless, some of them could have conferred selective advantage during human evolution and be fixed in the human genome. One such example identified in this study is the novel N-glycosylation site in UD19, which is involved in the elimination of potentially toxic xenobiotics and endogenous compounds. UD19 acquired the novel N-glycosylation site Asn-344 during the evolution of the common ancestor of apes and monkeys (see Figure 4C). When the N-glycosylation at Asn-344 is abolished, folding is inhibited in UD19, and its glucuronidase activity is reduced [54]. Therefore, glycosylation at Asn-344 is required for proper folding and activity of UD19. It is possible that ancestral simian primates required better defense mechanisms against toxic compounds introduced into their systems by environmental or dietary shifts. The acquisition of a new N-glycosylation site in UD19 might have conferred improved xenobiotics metabolism to apes and monkeys, although there is no direct evidence for this hypothesis.

The three human-specific N-glycosylation sites are particularly interesting (see Table 1 and Figure 3). The residue Asn-196 in APMAP is the first of the three human-specific N-glycosylation sites, which was inferred to be positively selected with an extremely high probability in humans (see Table 2 and Additional file 4). The human APMAP has been reported to be involved in a variety of biological processes including adipocyte differentiation, hepatic-specific metastasis in cancer, and inhibition of Aβ production [32-34]. The fact that APMAP is implicated in adipocyte differentiation is particularly interesting because humans and great apes exhibit large differences in adipose tissue and fatty acid storage, and these differences may be associated with the development of subcutaneous fat and even in brain development [58,59]. Therefore, the molecular functional study of human-specific sequence changes in proteins such as APMAP, which are associated with adipose tissue and lipid metabolism, may reveal the molecular mechanisms for the evolution of these traits.

**Figure 5:**
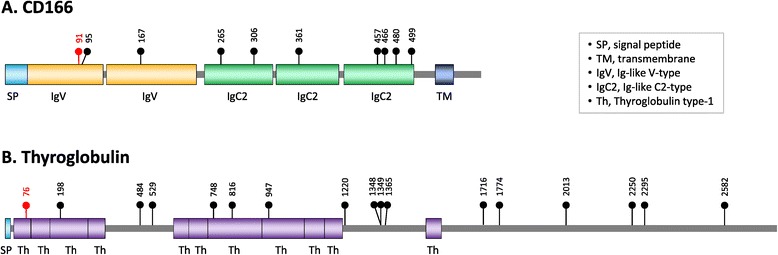
**Schematic domain organizations of CD166 (A) and thyroglobulin (B).** The N-glycosylation sites are indicated with lollipops, and human-specific sites are indicated in red. The domain organizations are derived from the UniProt database; the accession numbers are [Swiss-Prot:Q13740] (CD166) and [Swiss-Prot:P01216] (thyroglobulin).

The human CD166 protein has two Ig-like V-type domains and three Ig-like C2-type domains (Figure 5A) and functions as a cell adhesion molecule. The human-specific N-glycosylation site Asn-91, which was inferred to be positively selected (see Table 2 and Additional file 5), is located within the first Ig-like V-type domain, which is responsible for protein–protein interactions [35-37]. The addition of a bulky glycan moiety to this domain may change its structural profile and thus affect cell–cell adhesion activity or ligand specificity. The most interesting function of CD166 is its involvement in axon growth in neuronal cells [39,40]. Of the 583 amino acid residues in CD166, only the residue at position 91 differs between humans and chimpanzees; therefore, the emergence of Asn-91 and its N-glycosylation might be associated with evolution of human-specific phenotypes, probably in the nervous system, which must be determined experimentally.

The human thyroglobulin, which serves as a precursor molecule for the thyroid hormones T4 and T3, has a human-specific N-glycosylation site Asn-76. The human thyroglobulin contains 11 thyroglobulin type-1 domains, which are involved in the control of proteolytic degradation [60]. The human-specific Asn-76 is located within the first thyroglobulin type-1 domain (Figure 5B). The Asn-76 was inferred to be positively selected during human evolution, along with the other 20 positions (see Table 2 and Additional file 6). Removal of the glycan group from the Asn-76 reduced thyroid hormone production, especially T4 production [44]; thus, the gain of Asn-76 and its N-glycosylation, together with the other 20 putatively adaptive amino acid changes, may be implicated in the increased T4 concentration present in humans as compared to chimpanzees. It is possible that the additional glycan moiety may confer increased resistance to the proteolytic degradation of thyroglobulin proteins and thus lead to increased thyroid hormone production. It is very interesting that the T4 concentration in humans is higher than that in chimpanzees [45], as elevated T4 production may have caused the modification of human physiology in response to selection pressures in a specific environment: specifically, it has been proposed that an altered thyroid hormone metabolism might have been beneficial for early humans in the savannah environment, as they practiced persistence hunting and thus had large energy requirements [46].

Losses of N-glycosylation sites during human evolution are also very interesting. Some human diseases have been reported to be caused by the loss of N-glycosylation sites [61]. To find cases where ancestrally conserved N-glycosylation sites were lost during human evolution, a large amount of N-glycosylation data collected from animals distantly related to humans is required. The N-glycoproteome data obtained from mouse tissues and plasma using high-throughput mass spectrometry would be an ideal dataset for this analysis [62]. With a simple modification, the procedure described in this study could be used to analyze these data for the identification of N-glycosylation sites that were lost during human evolution and their possible phenotypic implications.

## Conclusions

We have devised and applied a bioinformatics method to identify the acquisition of N-glycosylation sites during human evolution. We propose that the acquisition of novel N-glycosylation sites may play a role in the development of lineage-specific phenotypes during evolution. Thus, the cases identified in this study may provide a useful resource for molecular functional analyses in search of human traits acquired during evolution.

## Methods

### Human N-glycosylation site data

The N-glycosylation sites in human proteins were obtained from the UniProt database (as of 13 November, 2013). The feature table of the UniProt records was scanned to collect entries with experimentally identified N-glycosylation sites. Specifically, the lines starting with “FT” followed by the “CARBOHYD” tag were examined for whether they contained a term “N-linked (GlcNAc…)”, which would indicate that the protein was N-glycosylated. Sites without experimental evidence, labeled as “potential”, “by similarity”, “partial”, or “probable”, were excluded. As a result, 2,534 N-glycosylation sites from 1,027 human proteins were obtained.

### Mammalian orthologous proteins

Mammalian orthologs of the human glycosylated proteins were obtained from the UCSC Genome Browser Database (http://genome.ucsc.edu). The “CDS FASTA alignment from multiple alignments” data, derived from the “multiz100way” alignment data prepared from 100 vertebrate genomes [25], were downloaded using the Table Browser tool of the UCSC Genome Browser. Protein sequences from 62 mammalian species were extracted from these alignment datasets. The selected mammalian species include humans, 12 other Euarchonta species (chimpanzees, gorillas, orangutans, gibbons, rhesus macaques, crab-eating macaques, baboons, green monkeys, marmosets, squirrel monkeys, bushbabies, and treeshrews), 13 Glires species (lesser Egyptian jerboas, prairie voles, Chinese hamsters, golden hamsters, mice, rats, naked mole-rats, guinea pigs, chinchillas, brush-tailed rats, rabbits, and pikas), 25 Laurasiatheria species (pigs, alpacas, Bactrian camels, dolphins, killer whales, Tibetan antelopes, cows, sheep, goats, horses, white rhinoceroses, cats, dogs, ferrets, pandas, Pacific walruses, Weddell seals, black flying-foxes, megabats, David’s myotis bats, microbats, big brown bats, hedgehogs, shrews, and star-nosed moles), six Afrotheria species (elephants, cape elephant shrews, manatees, cape golden moles, tenrecs, and aardvarks), one Xenarthra species (armadillos), three Marsupialia species (opossums, Tasmanian devils, and wallabies), and one Monotremata species (platypuses). Additional file 1 contains detailed information on species and genome assemblies.

### Computational screening for candidate novel N-glycosylation sites in human proteins

The total number of experimentally identified N-glycosylation sites collected from human proteins was 2,534. To identify mammalian proteins that were orthologous to each of the human N-glycosylated proteins, the “multiz100way” alignment data containing 57,289 alignment sets were analyzed (see Figure 1 for the overall procedure). There were 1,027 orthologous protein datasets comprising 2,534 human N-glycosylation sites. From each dataset, sequences of 62 mammalian species were extracted and realigned using MUSCLE (http://www.drive5.com/muscle) [63]. Then, each modification site in the alignment was analyzed, and cases where more than 30% of non-Euarchonta species had an N-glycosylation motif, which might represent ancestrally conserved sites, were discarded; cases where only a small number of sequences were aligned were also discarded. A total of 184 sites in 130 protein alignments were retained after this computational screening step and subjected to in-depth semimanual inspection.

### Manual inspection to select novel N-glycosylation sites in human proteins

As the final step, extensive manual inspection and curation on the 184 candidate sites was carried out to identify highly plausible cases of gains of N-glycosylation sites in the human lineage. Datasets showing the following conditions were filtered out: cases where the human sequence of UniProt database was different from that of UCSC in three or more amino acid sequence residues because of a possible paralogous relationship; cases where the N-glycosylated site was different from the consensus Asn-X-Ser/Thr; cases where the human N-glycosylation occurred only in a rare variant or mutant allele; or cases where the chimpanzee protein sequence was not included. In each dataset, sequences containing many gaps in alignment were removed from the dataset to retain only high quality sequences.

As the final result, 112 novel N-glycosylation sites in 91 human proteins were identified. Then, multiple alignments were constructed to determine when the N-glycosylation motifs first appeared. The possible functional consequences of the novel N-glycosylation site were then assessed by comprehensive literature survey and sequence analysis.

### Molecular evolutionary analysis

Full-length protein and coding sequences of APMAP, CD166, and thyroglobulin were collected from humans, chimpanzees, gorillas, orangutans, gibbons, and rhesus macaques. Human cDNA RefSeq sequences were obtained from the National Center for Biotechnology Information (NCBI) (http://www.ncbi.nlm.nih.gov/refseq): accession numbers are [NCBI:NM_020531] (APMAP), [NCBI:NM_001627] (CD166), and [NCBI:NM_003235] (thyroglobulin). For chimpanzee, gorilla, orangutan, gibbon, and rhesus macaque orthologs, genome assemblies (panTro4, gorGor3, ponAbe2, nomLeu3, and rheMac3, respectively) were searched using each of human cDNA sequences at the UCSC Genome Browser Database. Exons, which were predicted from genomic segments, were assembled into a virtual cDNA and then conceptually translated to get a protein sequence. Some exons, which were missing in the current genome assembly, were obtained by assembling whole genome shotgun reads by searching the NCBI Sequence Read Archive (SRA) with SRA-BLAST server (http://www.ncbi.nlm.nih.gov/sra).

The ratio of nonsynonymous to synonymous substitution rates (dN/dS, ω) was estimated by a likelihood method implemented in the codeml program of the PAML package (version 4.8a) [64]. To detect possible accelerated evolution in human proteins, we employed “branch models” that allow the ω ratio to vary among branches in phylogeny [27]; M0 (one ω ratio for all lineages), free ratio (one ω ratio for each branch), and two ratio (ω_1_ for the human branch and ω_0_ for the other branches). To infer positively selected sites in human proteins, we used “branch-site models” that allow the ω ratio to vary among both sites and lineages [26,28]; model A (ω is left to vary) and null model A (ω is fixed to 1). To compare the fit of nested models, the likelihood ratio test (LRT) was performed [27]. P values were obtained using the “chi2” program in the PAML package. Protein and coding sequences, tree files, control files, and major result files for APMAP, CD166, and thyroglobulin are provided in Additional files 4, 5, and 6, respectively.

## Additional files

Additional file 1:
**List of mammalian species and genome assemblies.**
Additional file 2:
**List of novel N-glycosylation sites.**
Additional file 3:
**Sequence alignments of novel N-glycosylation sites.**
Additional file 4:
**Molecular evolutionary analysis of APMAP.**
Additional file 5:
**Molecular evolutionary analysis of CD166.**
Additional file 6:
**Molecular evolutionary analysis of thyroglobulin.**

## Abbreviations

Aβ: Amyloid-beta
APMAP: Adipocyte plasma membrane-associated protein
CD166: Cluster of differentiation 166
CD6: Cluster of differentiation 6
PTM: Posttranslational modification
T3: Triiodothyronine
T4: Thyroxine
TSHR: Thyroid-stimulating hormone receptor
UD19: UDP-glucuronosyltransferase 1–9
