# Supplementary material for: The acquisition of novel N-glycosylation sites in conserved proteins during human evolution
Source: BMC Bioinformatics. 2015 Jan 28;16(1):29. doi: 10.1186/s12859-015-0468-5 (PMC4314935; doi:10.1186/s12859-015-0468-5)
Supplement: Additional file 1: — List of mammalian species and genome assemblies. [file 12859_2015_468_MOESM1_ESM.zip › 12859_2015_468_MOESM1_ESM.html]

## Additional file 1. List of mammalian species and genome assemblies

```
modified from http://hgdownload.cse.ucsc.edu/goldenPath/hg19/multiz100way/README.txt

Common name              Scientific name

== Euarchonta ==
Human                    Homo sapiens                    hg19        Feb 2009  GRCh37
Chimp                    Pan troglodytes                 panTro4     Feb 2011  CSAC 2.1.4
Gorilla                  Gorilla gorilla gorilla         gorGor3     May 2011  gorGor3.1
Orangutan                Pongo pygmaeus abelii           ponAbe2     Jul 2007  WUGSC 2.0.2
Gibbon                   Nomascus leucogenys             nomLeu3     Oct 2012  GGSC Nleu3.0
Rhesus macaque           Macaca mulatta                  rheMac3     Oct 2010  BGI CR_1.0
Crab-eating macaque      Macaca fascicularis             macFas5     Jun 2013  Macaca_fascicularis_5.0
Baboon                   Papio hamadryas                 papHam1     Nov 2008  Baylor Pham_1.0
Green monkey             Chlorocebus sabaeus             chlSab1     Jun 2013  Chlorocebus_sabeus 1.0
Marmoset                 Callithrix jacchus              calJac3     Mar 2009  WUGSC 3.2
Squirrel monkey          Saimiri boliviensis             saiBol1     Oct 2011  Broad
Bushbaby                 Otolemur garnettii              otoGar3     Mar 2011  Broad
Treeshrew                Tupaia chinensis                tupChi1     Jan 2013  TupChi_1.0

== Glires ==
Squirrel                 Spermophilus tridecemlineatus   speTri2     Nov 2011  Broad
Lesser Egyptian jerboa   Jaculus jaculus                 jacJac1     May 2012  JacJac1.0
Prairie vole             Microtus ochrogaster            micOch1     Oct 2012  MicOch1.0
Chinese hamster          Cricetulus griseus              criGri1     Jul 2013  C_griseus_v1.0
Golden hamster           Mesocricetus auratus            mesAur1     Mar 2013  MesAur1.0
Mouse                    Mus musculus                    mm10        Dec 2011  GRCm38
Rat                      Rattus norvegicus               rn5         Mar 2012  RGSC 5.0
Naked mole-rat           Heterocephalus glaber           hetGla2     Jan 2012  Broad HetGla_female_1.0
Guinea pig               Cavia porcellus                 cavPor3     Feb 2008  Broad
Chinchilla               Chinchilla lanigera             chiLan1     May 2012  ChiLan1.0
Brush-tailed rat         Octodon degus                   octDeg1     Apr 2012  OctDeg1.0
Rabbit                   Oryctolagus cuniculus           oryCun2     Apr 2009  Broad
Pika                     Ochotona princeps               ochPri3     May 2012  OchPri3.0

== Laurasiatheria ==
Pig                      Sus scrofa                      susScr3     Aug 2011  SGSC Sscrofa10.2
Alpaca                   Vicugna pacos                   vicPac2     Mar 2013  Vicugna_pacos-2.0.1
Bactrian camel           Camelus ferus                   camFer1     Dec 2011  CB1
Dolphin                  Tursiops truncatus              turTru2     Oct 2011  Baylor Ttru_1.4
Killer whale             Orcinus orca                    orcOrc1     Jan 2013  Oorc_1.1
Tibetan antelope         Pantholops hodgsonii            panHod1     May 2013  PHO1.0
Cow                      Bos taurus                      bosTau7     Oct 2011  Baylor Btau_4.6.1
Sheep                    Ovis aries                      oviAri3     Aug 2012  ISGC Oar_v3.1
Domestic goat            Capra hircus                    capHir1     May 2012  CHIR_1.0
Horse                    Equus caballus                  equCab2     Sep 2007  Broad
White rhinoceros         Ceratotherium simum             cerSim1     May 2012  CerSimSim1.0
Cat                      Felis catus                     felCat5     Sep 2011  ICGSC Felis_catus 6.2
Dog                      Canis lupus familiaris          canFam3     Sep 2011  Broad CanFam3.1
Ferret                   Mustela putorius furo           musFur1     Apr 2011  MusPutFur1.0
Panda                    Ailuropoda melanoleuca          ailMel1     Dec 2009  BGI-Shenzhen 1.0
Pacific walrus           Odobenus rosmarus divergens     odoRosDiv1  Jan 2013  Oros_1.0
Weddell seal             Leptonychotes weddellii         lepWed1     Mar 2013  LepWed1.0
Black flying-fox         Pteropus alecto                 pteAle1     Aug 2012  ASM32557v1
Megabat                  Pteropus vampyrus               pteVam1     Jul 2008  Broad
David's myotis bat       Myotis davidii                  myoDav1     Aug 2012  ASM32734v1
Microbat                 Myotis lucifugus                myoLuc2     Jul 2010  Broad Institute Myoluc2.0
Big brown bat            Eptesicus fuscus                eptFus1     Jul 2012  EptFus1.0
Hedgehog                 Erinaceus europaeus             eriEur2     May 2012  EriEur2.0
Shrew                    Sorex araneus                   sorAra2     Aug 2008  Broad
Star-nosed mole          Condylura cristata              conCri1     Mar 2012  ConCri1.0

== Afrotheria ==
Elephant                 Loxodonta africana              loxAfr3     Jul 2009  Broad
Cape elephant shrew      Elephantulus edwardii           eleEdw1     Aug 2012  EleEdw1.0
Manatee                  Trichechus manatus latirostris  triMan1     Oct 2011  Broad v1.0
Cape golden mole         Chrysochloris asiatica          chrAsi1     Aug 2012  ChrAsi1.0
Tenrec                   Echinops telfairi               echTel2     Nov 2012  Broad
Aardvark                 Orycteropus afer afer           oryAfe1     May 2012  OryAfe1.0

== Xenarthra ==
Armadillo                Dasypus novemcinctus            dasNov3     Dec 2011  Baylor

== Marsupialia ==
Opossum                  Monodelphis domestica           monDom5     Oct 2006  Broad
Tasmanian devil          Sarcophilus harrisii            sarHar1     Feb 2011  WTSI Devil_ref v7.0
Wallaby                  Macropus eugenii                macEug2     Sep 2009  TWGS Meug_1.1

== Monotremata ==
Platypus                 Ornithorhynchus anatinus        ornAna1     Mar 2007  WUGSC 5.0.1
```
