# Supplementary material for: The acquisition of novel N-glycosylation sites in conserved proteins during human evolution
Source: BMC Bioinformatics. 2015 Jan 28;16(1):29. doi: 10.1186/s12859-015-0468-5 (PMC4314935; doi:10.1186/s12859-015-0468-5)
Supplement: Additional file 2: — List of novel N-glycosylation sites. [file 12859_2015_468_MOESM2_ESM.zip › 12859_2015_468_MOESM2_ESM.html]

 List of novel N-glycosylation sites 
 
 Additional file 2. List of novel N-glycosylation sites 
 
  No Gene UniProt ID Position Sequence Clade Protein
  1 ABCA4  ABCA4_HUMAN  444  VGPQIWYFFD N S T QMNMIRD  simians Retinal-specific ATP-binding cassette transporter
  2 ADAMTS13  ATS13_HUMAN  614  VVAGKMSISP N T T YPSLLED  catarrhines A disintegrin and metalloproteinase with thrombospondin motifs 13
  3 ADAMTS13  ATS13_HUMAN  667  VYRRYGEEYG N L T RPDITFT  simians A disintegrin and metalloproteinase with thrombospondin motifs 13
  4 AFM  AFAM_HUMAN  33  PTQPRDIENF N S T QKFIEDN  apes Afamin
  5 ALCAM  CD166_HUMAN  91  DDVPEYKDRL N L S ENYTLSI  humans CD166 antigen
  6 AMBP  AMBP_HUMAN  36  PDNIQVQENF N I S RIYGKWY  simians Protein AMBP
  7 AMY2A  AMYP_HUMAN  476  DVISGDKING N C T GIKIYVS  primates Pancreatic alpha-amylase
  8 APMAP  APMAP_HUMAN  196  LSSETPIEGK N M S FVNDLTV  humans Adipocyte plasma membrane-associated protein
  9 APOB  APOB_HUMAN  3411  LSNKFVEGSH N S T VSLTTKN  simians Apolipoprotein B-100
  10 APOM  APOM_HUMAN  135  SSSCPGGIML N E T GQGYQRF  simians Apolipoprotein M
  11 AREG  AREG_HUMAN  119  KPPQNKTESE N T S DKPKRKK  simians Amphiregulin
  12 ASPH  ASPH_HUMAN  452  TLQRLVQLFP N D T SLKNDLG  primates Aspartyl/asparaginyl beta-hydroxylase
  13 AZGP1  ZA2G_HUMAN  109  ETLKDIVEYY N D S NGSHVLQ  primates Zinc-alpha-2-glycoprotein
  14 AZGP1  ZA2G_HUMAN  112  KDIVEYYNDS N G S HVLQGRF  primates Zinc-alpha-2-glycoprotein
  15 BCAM  BCAM_HUMAN  439  PTVPVLSRTQ N F T LLVQGSP  simians Basal cell adhesion molecule
  16 BTN3A1  BT3A1_HUMAN  115  TAGKAALRIH N V T ASDSGKY  primates Butyrophilin subfamily 3 member A1
  17 C1RL  C1RL_HUMAN  147  LTFRTQPSSE N K T AHLHKGF  simians Complement C1r subcomponent-like protein
  18 C3  CO3_HUMAN  85  VLTPATNHMG N V T FTIPANR  great apes Complement C3
  19 C4BPB  C4BPB_HUMAN  71  LFCNASKEWD N T T TECRLGH  simians C4b-binding protein beta chain
  20 C5  CO5_HUMAN  741  CCVVASQLRA N I S HKDMQLG  simians Complement C5
  21 CD163  C163A_HUMAN  1027  ECGHKEDAAV N C T DISVQKT  primates Scavenger receptor cysteine-rich type 1 protein M130
  22 CD48  CD48_HUMAN  104  LYISKVQKED N S T YIMRVLK  catarrhines CD48 antigen
  23 CD80  CD80_HUMAN  226  CLIKYGHLRV N Q T FNWNTTK  simians T-lymphocyte activation antigen CD80
  24 CD97  CD97_HUMAN  453  SAVNSIFLSH N N T KELNSPI  simians CD97 antigen
  25 CDH5  CADH5_HUMAN  112  VFAIERLDRE N I S EYHLTAV  simians Cadherin-5
  26 CEACAM1  CEAM1_HUMAN  224  ECEIQNPVSA N R S DPVTLNV  euarchonts Carcinoembryonic antigen-related cell adhesion molecule 1
  27 CEACAM1  CEAM1_HUMAN  405  WCEVFNPISK N Q S DPIMLNV  simians Carcinoembryonic antigen-related cell adhesion molecule 1
  28 CFH  CFAH_HUMAN  802  CINGRWDPEV N C S MAQIQLC  African great apes Complement factor H
  29 CFH  CFAH_HUMAN  822  PPPPQIPNSH N M T TTLNYRD  primates Complement factor H
  30 CFH  CFAH_HUMAN  911  CEGGFRISEE N E T TCYMGKW  apes Complement factor H
  31 CFH  CFAH_HUMAN  1029  ATYYKMDGAS N V T CINSRWT  apes Complement factor H
  32 CGB  CGHB_HUMAN  50  KEGCPVCITV N T T ICAGYCP  simians Choriogonadotropin subunit beta
  33 CLCA2  CLCA2_HUMAN  150  HFTPNFLLND N L T AGYGSRG  simians Calcium-activated chloride channel regulator 2
  34 CPN2  CPN2_HUMAN  228  SLQELFLDSN N I S ELPPQVF  African great apes Carboxypeptidase N subunit 2
  35 CRISP1  CRIS1_HUMAN  230  CDIQVHYLGC N H S TTILFCK  primates Cysteine-rich secretory protein 1
  36 CSF1R  CSF1R_HUMAN  353  SDHQPEPKLA N A T TKDTYRH  simians Macrophage colony-stimulating factor 1 receptor
  37 CSF2RB  IL3RB_HUMAN  191  SWEDAAILLS N T S QATLGPE  euarchonts Cytokine receptor common subunit beta
  38 CTSC  CATC_HUMAN  119  EEGSKVTTYC N E T MTGWVHD  primates Dipeptidyl peptidase 1
  39 DMBT1  DMBT1_HUMAN  1712  IVLDDVRCSG N E S YLWSCPH  humans and chimpanzees Deleted in malignant brain tumors 1 protein
  40 DSG2  DSG2_HUMAN  182  SAAHTLVMKI N A T DADEPNT  euarchonts Desmoglein-2
  41 ENPEP  AMPE_HUMAN  773  NASSLFEQWL N G T VSLPVNL  catarrhines Glutamyl aminopeptidase
  42 ENPP1  ENPP1_HUMAN  748  GFLSPPQLNK N S S GIYSEAL  primates Ectonucleotide pyrophosphatase/phosphodiesterase family member 1
  43 ENPP7  ENPP7_HUMAN  168  RKEGIAHNYK N E T EWRANID  primates Ectonucleotide pyrophosphatase/phosphodiesterase family member 7
  44 F7  FA7_HUMAN  382  QQSRKVGDSP N I T EYMFCAG  simians Coagulation factor VII
  45 FCER1A  FCERA_HUMAN  99  GEYKCQHQQV N E S EPVYLEV  African great apes High affinity immunoglobulin epsilon receptor subunit alpha
  46 FCGBP  FCGBP_HUMAN  75  ASVSILSQAD N T S KKVTVRP  apes IgGFc-binding protein
  47 FCGBP  FCGBP_HUMAN  2138  YTRSVTLQIY N H S LTLSARW  simians IgGFc-binding protein
  48 FCGR1A  FCGR1_HUMAN  152  KAFKFFHWNS N L T ILKTNIS  catarrhines High affinity immunoglobulin gamma Fc receptor I
  49 FCGR1A  FCGR1_HUMAN  195  VKELFPAPVL N A S VTSPLLE  catarrhines High affinity immunoglobulin gamma Fc receptor I
  50 GP1BA  GP1BA_HUMAN  37  SHLEVNCDKR N L T ALPPDLP  catarrhines Platelet glycoprotein Ib alpha chain
  51 GP1BA  GP1BA_HUMAN  175  KLEKLSLANN N L T ELPAGLL  African great apes Platelet glycoprotein Ib alpha chain
  52 GUSB  BGLR_HUMAN  272  LLDAENKVVA N G T GTQGQLK  catarrhines Beta-glucuronidase
  53 HEG1  HEG1_HUMAN  520  SSSTSSSESL N S S APRGERS  simians Protein HEG homolog 1
  54 HLA-DMB  DMB_HUMAN  110  HTQPFWGSLT N R T RPPSVQV  apes HLA class II histocompatibility antigen, DM beta chain
  55 HPSE  HPSE_HUMAN  238  SFLKKADIFI N G S QLGEDFI  simians Heparanase
  56 HPX  HEMO_HUMAN  453  NAAKALPQPQ N V T SLLGCTH  apes Hemopexin
  57 HRG  HRG_HUMAN  63  IADAHLDRVE N T T VYYLVLD  catarrhines Histidine-rich glycoprotein
  58 ICAM1  ICAM1_HUMAN  267  QRLNPTVTYG N D S FSAKASV  African great apes Intercellular adhesion molecule 1
  59 ICAM2  ICAM2_HUMAN  153  DSLTLFLFRG N E T LHYETFG  apes Intercellular adhesion molecule 2
  60 ICAM3  ICAM3_HUMAN  84  VASGMGWAAF N L S NVTGNSR  African great apes Intercellular adhesion molecule 3
  61 IFNG  IFNG_HUMAN  48  FNAGHSDVAD N G T LFLGILK  simians Interferon gamma
  62 IFNG  IFNG_HUMAN  120  KKRDDFEKLT N Y S VTDLNVQ  simians Interferon gamma
  63 IL17F  IL17F_HUMAN  83  NIESRSTSPW N Y T VTWDPNR  simians Interleukin-17F
  64 IL6  IL6_HUMAN  73  GISALRKETC N K S NMCESSK  simians Interleukin-6
  65 ITGAM  ITAM_HUMAN  946  TKYLNFTASE N T S RVMQHQY  simians Integrin alpha-M
  66 ITGAX  ITAX_HUMAN  697  SPRATFQETK N R S LSRVRVL  catarrhines Integrin alpha-X
  67 ITGB2  ITB2_HUMAN  212  FAFRHVLKLT N N S NQFQTEV  African great apes Integrin beta-2
  68 KCNK18  KCNKI_HUMAN  70  KFLEELCRIL N C S ETVVEDR  simians Potassium channel subfamily K member 18
  69 LGALS3BP  LG3BP_HUMAN  192  AQALWKEPGS N V T MSVD--A  simians Galectin-3-binding protein
  70 LHB  LSHB_HUMAN  50  KEGCPVCITV N T T ICAGYCP  simians Lutropin subunit beta
  71 MEP1B  MEP1B_HUMAN  370  REYSADNVDG N L T LVEEIKE  catarrhines Meprin A subunit beta
  72 MFGE8  MFGM_HUMAN  238  NGCANPLGLK N N S IPDKQIT  simians Lactadherin
  73 MGAM  MGA_HUMAN  295  IFNRDTTPNG N G T NLYGAQT  simians Maltase-glucoamylase, intestinal
  74 MGAM  MGA_HUMAN  827  GGYIFPTQQP N T T TLASRKN  euarchonts Maltase-glucoamylase, intestinal
  75 MMRN1  MMRN1_HUMAN  114  EKAEGVVKLQ N L T LPTNASI  simians Multimerin-1
  76 MMRN1  MMRN1_HUMAN  120  VKLQNLTLPT N A S IKFNPGA  apes Multimerin-1
  77 MUC5B  MUC5B_HUMAN  5215  QARLPYSLFH N N T EGQCGTC  simians Mucin-5B
  78 NPC2  NPC2_HUMAN  135  VVEWQLQDDK N Q S LFCWEIP  simians Epididymal secretory protein E1
  79 PAPPA  PAPP1_HUMAN  480  GGECCDPEIT N V T QTCFDPD  apes Pappalysin-1
  80 PGLYRP2  PGRP2_HUMAN  77  YHFLLGAWSL N A T ELDPCPL  African great apes N-acetylmuramoyl-L-alanine amidase
  81 PGLYRP2  PGRP2_HUMAN  367  SQEQLAQVAA N A T KEFTEAF  simians N-acetylmuramoyl-L-alanine amidase
  82 PIGR  PIGR_HUMAN  186  VIDSSGYVNP N Y T GRIRLDI  African great apes Polymeric immunoglobulin receptor
  83 PIGR  PIGR_HUMAN  499  SSYEKYWCKW N N T GCQALPS  apes Polymeric immunoglobulin receptor
  84 PRG2  PRG2_HUMAN  86  ISVPDM-VDK N L T CPEEEDT  catarrhines Bone marrow proteoglycan
  85 PTPRC  PTPRC_HUMAN  276  NEVHNLTECK N A S VSISHNS  African great apes Receptor-type tyrosine-protein phosphatase C
  86 PTPRC  PTPRC_HUMAN  335  NIETFTCDTQ N I T YRFQCGN  great apes Receptor-type tyrosine-protein phosphatase C
  87 PTPRC  PTPRC_HUMAN  419  TWNPPQRSFH N F T LCYIKET  simians Receptor-type tyrosine-protein phosphatase C
  88 PTPRJ  PTPRJ_HUMAN  342  LVGLEPGTRY N A T VYSQAAN  simians Receptor-type tyrosine-protein phosphatase eta
  89 PTPRJ  PTPRJ_HUMAN  396  WKVSDNESSS N Y T YKIHVAG  catarrhines Receptor-type tyrosine-protein phosphatase eta
  90 PTPRJ  PTPRJ_HUMAN  413  VAGETDSSNL N V S EPRAVIP  great apes Receptor-type tyrosine-protein phosphatase eta
  91 PVR  PVR_HUMAN  278  ARSNPEPTGY N W S TTMGPLP  catarrhines Poliovirus receptor
  92 RNASE1  RNAS1_HUMAN  104  KNGQGNCYKS N S S MHITDCR  apes Ribonuclease pancreatic
  93 RNASE1  RNAS1_HUMAN  116  SMHITDCRLT N G S RYPNCAY  primates Ribonuclease pancreatic
  94 SERPINA6  CBG_HUMAN  31  QAMDPNAAYV N M S NHHRGLA  simians Corticosteroid-binding globulin
  95 SERPINA6  CBG_HUMAN  369  DTAGSTGVTL N L T SKPIILR  catarrhines Corticosteroid-binding globulin
  96 SLC1A5  AAAT_HUMAN  212  RSYSTTYEER N I T GTRVKVP  simians Neutral amino acid transporter B(0)
  97 SLC3A2  4F2_HUMAN  381  SEDRLLIAGT N S S DLQQILS  simians 4F2 cell-surface antigen heavy chain
  98 SLC4A7  S4A7_HUMAN  791  QWKKDNITAH N I S WRNLTVS  humans and chimpanzees Sodium bicarbonate cotransporter 3
  99 SMPDL3A  ASM3A_HUMAN  263  VPVGYLPSSQ N I T AMREYYN  great apes Acid sphingomyelinase-like phosphodiesterase 3a
  100 SPARCL1  SPRL1_HUMAN  412  GEHQEAKKAE N S S NEEETSS  simians SPARC-like protein 1
  101 SPP1  OSTP_HUMAN  106  HVDSQDSIDS N D S DDVDDTD  simians Osteopontin
  102 SUSD2  SUSD2_HUMAN  522  NSDVVEVRLA N R T GGLEVLL  simians Sushi domain-containing protein 2
  103 TCN1  TCO1_HUMAN  216  QIKADEGSLK N I S IYTKSLV  catarrhines Transcobalamin-1
  104 TFR2  TFR2_HUMAN  754  LLDHLRLLRS N S S GTPGATS  primates Transferrin receptor protein 2
  105 TG  THYG_HUMAN  76  DGRSCWCVGA N G S EVLGSRQ  humans Thyroglobulin
  106 TLR1  TLR1_HUMAN  51  KDLSQKTTIL N I S QNYISEL  primates Toll-like receptor 1
  107 TLR5  TLR5_HUMAN  422  GNKLVTLPKI N L T ANLIHLS  simians Toll-like receptor 5
  108 TNC  TENA_HUMAN  1034  GQWVGVQLPR N T T SYVLRGL  euarchonts Tenascin
  109 TNC  TENA_HUMAN  1275  TEVSWDALRL N W T TPDGTYD  primates Tenascin
  110 TSHR  TSHR_HUMAN  113  VTHIEIRNTR N L T YIDPDAL  apes Thyrotropin receptor
  111 UGT1A9  UD19_HUMAN  344  YTGTRPSNLA N N T ILVKWLP  simians UDP-glucuronosyltransferase 1-9
  112 VNN1  VNN1_HUMAN  283  KMTGSGIYAP N S S RAFHYDM  catarrhines Pantetheinase
 
