# Supplementary material for: The acquisition of novel N-glycosylation sites in conserved proteins during human evolution
Source: BMC Bioinformatics. 2015 Jan 28;16(1):29. doi: 10.1186/s12859-015-0468-5 (PMC4314935; doi:10.1186/s12859-015-0468-5)
Supplement: Additional file 3: — Sequence alignments of novel N-glycosylation sites. [file 12859_2015_468_MOESM3_ESM.zip › 12859_2015_468_MOESM3_ESM.html]

## Additional file 3. Sequence alignments of novel N-glycosylation sites.

```
# NO number
# GN gene symbol
# ID UniProt ID
# MP modified position
# DE description
# CL clade sharing the novel asparagine residue
# SQ number of species
#    part of aligned sequences followed by phylogenetic groups and species
#    parenthesis, the amino acid position where the human asparagine is N-glycosylated
#    ., the same as the human sequence
#    -, alignment gap
#    *, stop codon (either authentic or erroneous)
#    O, N-glycosylation motif N-X-S/T
#    X, no N-glycosylation motif
#    hum, humans
#    hac, humans and chimpanzees
#    aga, African great apes
#    gra, great apes
#    ape, apes
#    cat, catarrhines
#    sim, simians
#    pri, primates
#    eua, Euarchonta
#    gli, Glires
#    lau, Laurasiatheria
#    afr, Afrotheria
#    xen, Xenarthra
#    mar, Marsupialia
#    mon, Monotremata

NO 1
GN ABCA4
ID ABCA4_HUMAN
MP 444
DE Retinal-specific ATP-binding cassette transporter
CL simians
SQ 62
   KAWEEVGPQIWYFFD(NST)QMNMIRDTLGNPT   O   hum   Human
   ...............(...).............   O   hac   Chimp
   ...............(...).............   O   aga   Gorilla
   ...............(...).............   O   gra   Orangutan
   ...............(...).............   O   ape   Gibbon
   ...............(...).......I.....   O   cat   Rhesus macaque
   ...............(...).......I.....   O   cat   Crab-eating macaque
   ...............(...).......I.....   O   cat   Baboon
   ...............(...).......I.....   O   cat   Green monkey
   ...............(...).............   O   sim   Marmoset
   ...............(...).............   O   sim   Squirrel monkey
   .....I.........(K..)..T........SA   X   pri   Bushbaby
   ...............(K..)..T......E...   X   eua   Treeshrew
   ..............E(S..)..T......E-..   X   gli   Squirrel
   ...R......E...E(...)..T.......H..   O   gli   Lesser Egyptian jerboa
   ...............(K..)..T......EH.A   X   gli   Prairie vole
   ...G..........E(K..)..T.......H..   X   gli   Chinese hamster
   ..............E(K..)..T.M.-------   X   gli   Golden hamster
   ..............E(K..)..TV.....QH..   X   gli   Mouse
   ..............E(K..)..AV.....QH..   X   gli   Rat
   ...............(...)..T......EH..   O   gli   Naked mole-rat
   ...K...........(..I)..T...E..EH..   X   gli   Guinea pig
   ...K..........A(...)..T.......H..   O   gli   Chinchilla
   ...K..........N(...)..M.V....EH..   O   gli   Brush-tailed rat
   .....I.........(K.P)..H...N..R..A   X   gli   Rabbit
   ...............(K.P).........Q..A   X   gli   Pika
   R..............(K..)P.A....S.E...   X   lau   Pig
   ........E......(K.A)..T......E...   X   lau   Alpaca
   ........E......(K..)..T....A.E...   X   lau   Bactrian camel
   ...............(K.M)..T......E...   X   lau   Dolphin
   ...............(K.M)..T......E...   X   lau   Killer whale
   .V.............(K..)..T......E...   X   lau   Tibetan antelope
   .V.............(K..)..S......E...   X   lau   Cow
   .V.............(K..)..T......E...   X   lau   Sheep
   .V.............(K..)..T......E...   X   lau   Domestic goat
   ...............(K..)..T..........   X   lau   Horse
   ...............(EG.)..T..........   X   lau   White rhinoceros
   ...............(K..)..T......Q...   X   lau   Cat
   ...............(R..)..T......E...   X   lau   Dog
   ...............(K..)..T......E...   X   lau   Ferret
   ...............(K..).IT......E...   X   lau   Panda
   ...............(K..)..AT.....E...   X   lau   Pacific walrus
   ...............(K..)R.AT.....E...   X   lau   Weddell seal
   ...............(K..)..TT.....A..I   X   lau   Black flying-fox
   ...............(K..)..TT.....A..I   X   lau   Megabat
   ...............(K..)..T......A...   X   lau   David's myotis bat
   ...............(K..)..T......A...   X   lau   Microbat
   ...............(K..)..T...N..A...   X   lau   Big brown bat
   ...............(R..)..T......E...   X   lau   Hedgehog
   ...........D...(K..)..T...N..A...   X   lau   Shrew
   ...........S.L.(K.V)..A..K...Q..A   X   lau   Star-nosed mole
   ...............(K..)..T....I.E...   X   afr   Elephant
   ...............(K..)..A......E...   X   afr   Cape elephant shrew
   .S.............(K..)..T....I.E...   X   afr   Manatee
   ...............(K.I)..T.....QE...   X   afr   Cape golden mole
   ..............E(E..)..T......D...   X   afr   Tenrec
   .S.............(K..)..T......E...   X   afr   Aardvark
   ...............(R..)..AL.....E...   X   xen   Armadillo
   ...........H...(...)..T......E...   O   mar   Opossum
   ...........H...(K..)..A......E...   X   mar   Tasmanian devil
   ...........H...(...).IT....IPE...   O   mar   Wallaby
   .V.........D..H(...)..A...-------   O   mon   Platypus

NO 2
GN ADAMTS13
ID ATS13_HUMAN
MP 614
DE A disintegrin and metalloproteinase with thrombospondin motifs 13
CL catarrhines
SQ 59
   IGGRYVVAGKMSISP(NTT)YPSLLEDGRVEYR   O   hum   Human
   ...............(...).............   O   hac   Chimp
   ...............(...).............   O   aga   Gorilla
   L.........T....(...).........I...   O   gra   Orangutan
   ..........T....(...).............   O   ape   Gibbon
   ..........T...S(...).............   O   cat   Rhesus macaque
   ..........T...S(...).............   O   cat   Crab-eating macaque
   ..........T...S(...).............   O   cat   Baboon
   ..........T....(...).............   O   cat   Green monkey
   ..A.......S....(S..).............   X   sim   Marmoset
   ..A.......S....(S..).............   X   sim   Squirrel monkey
   V.........A....(S.I)F......S.....   X   pri   Bushbaby
   M..GSTGG.NK.L..(S..).............   X   eua   Treeshrew
   .R...I...NG....(S..).......S.....   X   gli   Squirrel
   .Q........T....(S..)...I...S.....   X   gli   Lesser Egyptian jerboa
   TK.H.I....T....(SII).......F.....   X   gli   Prairie vole
   .Q.H.I....T....(SIM).......C.....   X   gli   Chinese hamster
   VQ.H.I....T....(S.M).......C.....   X   gli   Golden hamster
   .Q.H.I....T....(...).......Y.....   O   gli   Mouse
   .QDH.I.S..S.M..(.V.).......Y.....   O   gli   Rat
   .R.H.....EG..A.(S..)H......S.M...   X   gli   Naked mole-rat
   .........EAGLA.(S..)H......S.....   X   gli   Guinea pig
   .Q.......EAG.A.(...)H......S.....   O   gli   Chinchilla
   .Q.......EAG.A.(S..)H..P...S.....   X   gli   Brush-tailed rat
   VR.......NA.L..(S..)H......S.....   X   gli   Rabbit
   VH.......NA...S(S.S)H..P...S.L...   X   gli   Pika
   .R.......NA....(SI.).......S.....   X   lau   Pig
   LR...I...NG....(S.S).......S..Q.K   X   lau   Alpaca
   LR...I...NG....(S.S).......S..QHK   X   lau   Bactrian camel
   .R.......NA....(S..).......S.IQ.K   X   lau   Dolphin
   .R.......NA....(S..).......S.IQ.K   X   lau   Killer whale
   VC...I...NG...A(S.S).......N.....   X   lau   Tibetan antelope
   VR.......NG.A.A(S.S).......N.....   X   lau   Cow
   VR...I...NG...A(S.S).......N.....   X   lau   Domestic goat
   .R.......NS....(S..).......S....K   X   lau   Horse
   .Q.......NS...L(S..).......S....K   X   lau   White rhinoceros
   .....I...NS....(S..).......S.....   X   lau   Cat
   .R.H.I...NS....(S..).......S.....   X   lau   Dog
   .R.......TS....(S..).......S..G..   X   lau   Ferret
   .R.......NS....(S..).......S.....   X   lau   Panda
   .R.H.....NS....(S..).......S.....   X   lau   Pacific walrus
   .R.......NS....(S..).......S.....   X   lau   Weddell seal
   .R...I...NS...A(S..)H......R..Q..   X   lau   Black flying-fox
   .R.......NF....(S.S)H......S.I...   X   lau   David's myotis bat
   .R.H.....NF....(S.S)H......S.I...   X   lau   Microbat
   .R.......NF....(S.S)H..P...S.I...   X   lau   Big brown bat
   .........NS....(S..).......SC...K   X   lau   Hedgehog
   VA.......SARP.G(S.V)...A...E.L...   X   lau   Shrew
   .R.......NG.V..(S..).......S.L...   X   afr   Elephant
   VR.Q.....N.....(S..)...V...S.L...   X   afr   Cape elephant shrew
   .R.......NA....(S..).......S.L...   X   afr   Manatee
   .R.Q.....NT.V..(S..)...I...SHLK.T   X   afr   Cape golden mole
   .....II..NS....(S..)R..V...S.LS..   X   afr   Tenrec
   .R.Q.I...NS....(...).......C.I...   O   afr   Aardvark
   V........ESRP..(S.A).....D.S.....   X   xen   Armadillo
   VR.H...S..RR..S(...)H..V...KQI...   O   mar   Opossum
   VR.Q...S..K...F(...)...V...NQI..K   O   mar   Tasmanian devil
   VR.Q...S..RI..F(...)...V...NQI..K   O   mar   Wallaby
   VR.Q......TR..L(.V.)...V...S.IA.K   O   mon   Platypus

NO 3
GN ADAMTS13
ID ATS13_HUMAN
MP 667
DE A disintegrin and metalloproteinase with thrombospondin motifs 13
CL simians
SQ 60
   DADIQVYRRYGEEYG(NLT)RPDITFTYFQPKP   O   hum   Human
   ...............(...).............   O   hac   Chimp
   ...............(...).............   O   aga   Gorilla
   ..E............(...)C............   O   gra   Orangutan
   ..E............(...)C............   O   ape   Gibbon
   ..E............(...)C............   O   cat   Rhesus macaque
   ..E............(...)C............   O   cat   Crab-eating macaque
   ..E............(...)C............   O   cat   Baboon
   ..E............(...)C...........L   O   cat   Green monkey
   ..E............(S..)...V.........   X   sim   Marmoset
   ..E............(...)C..V.........   O   sim   Squirrel monkey
   AVE.....K.S....(D..)....A.S......   X   pri   Bushbaby
   .ME.......NDQ..(D..)............Q   X   eua   Treeshrew
   .ME......HS..H.(Q..).TNV.......RQ   X   gli   Squirrel
   .ME............(D.A)H...........R   X   gli   Lesser Egyptian jerboa
   .VE.......RGD..(D..)H........E..Q   X   gli   Prairie vole
   .ME.......RAD..(D..)H.........L.Q   X   gli   Chinese hamster
   .ME.......RA...(D..)H...........Q   X   gli   Golden hamster
   .IE........G...(D..)H.....S...L.Q   X   gli   Mouse
   .IE........G...(G..)H.........R.Q   X   gli   Rat
   .VE..........H.(A..)...V........Q   X   gli   Naked mole-rat
   .VEV...........(A..)H...........Q   X   gli   Guinea pig
   .ME............(D..)............Q   X   gli   Chinchilla
   AVE............(A.A)..........S.Q   X   gli   Brush-tailed rat
   .IE........D...(A..)......S.....Q   X   gli   Rabbit
   AME........D...(P.A)H...I....E..Q   X   gli   Pika
   .LE........Q...(R.A)...........EQ   X   lau   Pig
   .ME........A...(A..)...V.......ER   X   lau   Alpaca
   .ME........A...(A.A)...V........R   X   lau   Bactrian camel
   .ME............(S.A)............R   X   lau   Dolphin
   .ME............(S.A)............R   X   lau   Killer whale
   .ME............(SPA)...........EQ   X   lau   Tibetan antelope
   .ME.......S....(SPA)......I....EQ   X   lau   Cow
   ---------------(SPA)...........EQ   X   lau   Sheep
   .ME............(E..)............Q   X   lau   Horse
   .ME............(E..)............Q   X   lau   White rhinoceros
   .VE............(D.A)............Q   X   lau   Cat
   .ME............(D.A).........E..Q   X   lau   Dog
   .VE............(D.A)............R   X   lau   Ferret
   .ME............(D.A)...........QQ   X   lau   Panda
   .VE.....H......(D.V)...........QQ   X   lau   Pacific walrus
   .VE............(D.A)........L..QQ   X   lau   Weddell seal
   .ME............(D..)Q........R..R   X   lau   Black flying-fox
   -----..........(D..)Q........R..R   X   lau   Megabat
   .ME............(.A.)..HV........L   O   lau   David's myotis bat
   .ME............(.A.)..HV........L   O   lau   Microbat
   .VE............(.A.)..H.........L   O   lau   Big brown bat
   HME........K.F.(D..)S...........Q   X   lau   Hedgehog
   PMEV.......D...(A.S)...........EQ   X   lau   Shrew
   .IE.......S....(S.A)H.....S.....Q   X   afr   Elephant
   .VE......H...F.(A.A)..E...S...S.Q   X   afr   Cape elephant shrew
   .IE............(S.A)......S.....Q   X   afr   Manatee
   .IE........D...(SPA)......S...L--   X   afr   Cape golden mole
   .IEV.......D...(S.A)......S.....Q   X   afr   Tenrec
   .IE............(S.A)C.N...S.....Q   X   afr   Aardvark
   VIE........D.H.(H.S)...V..S....V.   X   xen   Armadillo
   .IE.....K..N...(...)..E...S..K..Q   O   mar   Opossum
   .IEV....K..N...(...)N.E...S..K..Q   O   mar   Tasmanian devil
   .IE.....K..N...(...)S.E...S..K..Q   O   mar   Wallaby
   VIE.....K..R...(.V.)N...A.S....TT   O   mon   Platypus

NO 4
GN AFM
ID AFAM_HUMAN
MP 33
DE Afamin
CL apes
SQ 52
   ESLTLPTQPRDIENF(NST)QKFIEDNIEYITI   O   hum   Human
   ...........V...(...).............   O   hac   Chimp
   ...........V...(...).....E.......   O   aga   Gorilla
   .........Q.V...(...).....E.......   O   gra   Orangutan
   ......I..Q.VD..(...).....E.V.....   O   ape   Gibbon
   .........Q.VD..(S..).....E.L.....   X   cat   Rhesus macaque
   .........Q.VD..(S..).....E.L.....   X   cat   Crab-eating macaque
   .....T...Q.VD..(S..).....E.L.....   X   cat   Baboon
   .........Q.VD..(S..).....E.L.....   X   cat   Green monkey
   ...M...ELQ.LD..(.I.).....E.V.....   O   sim   Marmoset
   ...M...ELQ.LD..(.I.).....E.V.....   O   sim   Squirrel monkey
   .......K.E.VDD.(EI.)...V....AF.AT   X   pri   Bushbaby
   ...P...K.Q.ADD.(SVV).....E.V....T   X   eua   Treeshrew
   ...S.L.ETQ.VDS.(GI.).....E.TGS...   X   gli   Squirrel
   ...V...K.Q.VDP.(EAI).....E.TA.L..   X   gli   Prairie vole
   ...A...K.Q.VDP.(YA.)H....E.TA.L..   X   gli   Chinese hamster
   ...A...K.Q..DP.(LD.)H....E.TA.L..   X   gli   Golden hamster
   ...A...K.Q.VDH.(.A.)....DE.TT.LA.   O   gli   Mouse
   ...A...K.Q.VDH.(.A.)....NE.VA.L..   O   gli   Rat
   D........Q..G..(.VN).....E..VPM..   X   gli   Naked mole-rat
   ........LQEVD..(.V.)....NE.TASM..   O   gli   Guinea pig
   .......K.Q.VD.I(.VS)R...DE.LASMA.   O   gli   Brush-tailed rat
   A..I...K.Q.VDD.(SV.).....E.AG.L..   X   gli   Rabbit
   .....T.K.Q.VDD.(SI.).N..DE.TG.L..   X   gli   Pika
   .......K.Q..DDV(RI.)K......VV....   X   lau   Pig
   .......K.Q.VDDV(SV.)K....E.VG....   X   lau   Alpaca
   .......K.Q.VDDV(SV.)K....E.V.....   X   lau   Bactrian camel
   .......K.Q.VDDV(SI.)........G....   X   lau   Dolphin
   .......K.Q.VDDV(SI.)........G....   X   lau   Killer whale
   .........Q.VDDV(RI.)........G....   X   lau   Tibetan antelope
   .........Q.VDDV(RI.)....D...G....   X   lau   Cow
   .........Q.VDDV(RI.).......VG....   X   lau   Sheep
   .........Q.VDDV(RI.).......VG....   X   lau   Domestic goat
   .......KLQ.VDDA(SI.).....E..G...V   X   lau   Horse
   .......KLQ.VDDA(SI.).....K..G....   X   lau   White rhinoceros
   .....S.K.Q.VDDV(SI.).....E.VG....   X   lau   Cat
   .........Q.LDDV(I..).....E.VG....   X   lau   Dog
   .........Q.VDDV(SI.).....E.VG....   X   lau   Ferret
   .........Q.VDDV(.I.).....E.VG....   O   lau   Panda
   .........Q.VDDV(SI.).....E.VG....   X   lau   Pacific walrus
   .........Q.VDDV(SI.).....E.VG....   X   lau   Weddell seal
   .......K.Q.VDD.(RI.).....E.VG....   X   lau   Black flying-fox
   -------------D.(RI.).....E.VG....   X   lau   Megabat
   .C.S...K.Q.VDD.(.I.).......VG....   O   lau   David's myotis bat
   .C.S...K.Q.VDD.(.II).......VG....   X   lau   Microbat
   KC.N...K.Q.VDD.(.I.).......VG....   O   lau   Big brown bat
   .F..P.SKAQ.VDIS(QV.)....L..A..L..   X   afr   Cape elephant shrew
   .......KSQ.VD..(QV.).......VG.L..   X   afr   Aardvark
   ......RA.QTKDYL(.A.)...L.N..RDV.T   O   mar   Opossum
   --------------R(.A.)...L.N.TRDV.T   O   mar   Tasmanian devil
   ......KA.DQEDYL(.V.)...L.N..R----   O   mar   Wallaby
   -------------YL(.A.)NQY.QK.VRSL.T   O   mon   Platypus

NO 5
GN ALCAM
ID CD166_HUMAN
MP 91
DE CD166 antigen
CL humans
SQ 61
   KSVQYDDVPEYKDRL(NLS)ENYTLSISNARIS   O   hum   Human
   ...............(S..).............   X   hac   Chimp
   ...............(S..).............   X   aga   Gorilla
   ...............(S..).............   X   gra   Orangutan
   ...............(S..).............   X   ape   Gibbon
   ...............(S..).............   X   cat   Rhesus macaque
   ...............(S..).............   X   cat   Crab-eating macaque
   ...............(S..).............   X   cat   Baboon
   ...............(S..).............   X   cat   Green monkey
   ...............(S..).............   X   sim   Marmoset
   ...............(S..).............   X   sim   Squirrel monkey
   ....F..........(S..)......V......   X   pri   Bushbaby
   ...............(S..).............   X   eua   Treeshrew
   ...............(S..)..........K..   X   gli   Squirrel
   .N.........R...(S..)..........K..   X   gli   Lesser Egyptian jerboa
   ............G..(...).......N..K..   O   gli   Prairie vole
   ...............(S..).......N..K..   X   gli   Chinese hamster
   ...............(S..).......N..K..   X   gli   Golden hamster
   ...............(S..).......A..K..   X   gli   Mouse
   ...............(S..).......N..K..   X   gli   Rat
   .........D.....(S..)..........K.G   X   gli   Naked mole-rat
   ...Y...........(S..)..........K..   X   gli   Guinea pig
   ...............(S..)..........K..   X   gli   Chinchilla
   ...............(S..)..........K..   X   gli   Brush-tailed rat
   ...............(...).............   O   gli   Rabbit
   ...............(S..).............   X   gli   Pika
   ............N..(S..)..........K..   X   lau   Pig
   ...............(S.Y)........D...G   X   lau   Alpaca
   ...............(S.Y)........D....   X   lau   Bactrian camel
   ...............(...)..........K..   O   lau   Dolphin
   ...............(...)..........K..   O   lau   Killer whale
   ...............(...).............   O   lau   Tibetan antelope
   ...............(...)..........K..   O   lau   Cow
   ...............(...).............   O   lau   Sheep
   ...............(...).............   O   lau   Domestic goat
   ...............(S..).............   X   lau   Horse
   ...............(...).............   O   lau   White rhinoceros
   ...............(S..).............   X   lau   Cat
   ...............(S..).............   X   lau   Dog
   ...........R...(S..).............   X   lau   Ferret
   ...............(S..).............   X   lau   Panda
   ...............(S..).............   X   lau   Pacific walrus
   ...............(S..).............   X   lau   Weddell seal
   ...H...........(S..).............   X   lau   Black flying-fox
   ...H...........(S..).............   X   lau   Megabat
   ...............(S..).............   X   lau   David's myotis bat
   ...............(S..).............   X   lau   Microbat
   ...............(S..).............   X   lau   Big brown bat
   ...........R...(S..).............   X   lau   Hedgehog
   ...............(S..)............G   X   lau   Shrew
   ...............(...).............   O   lau   Star-nosed mole
   .N.............(S..).......T.....   X   afr   Elephant
   ...H...........(S..).............   X   afr   Cape elephant shrew
   ...............(S..).D...........   X   afr   Manatee
   ...............(S..).............   X   afr   Cape golden mole
   ...........R...(S..)........H....   X   afr   Tenrec
   ...............(S..).............   X   afr   Aardvark
   ......E........(S..).......N.....   X   xen   Armadillo
   .H....E........(S..)........H....   X   mar   Opossum
   .H....Q........(S..)........H....   X   mar   Tasmanian devil
   .N....E........(I..).D.....N....G   X   mon   Platypus

NO 6
GN AMBP
ID AMBP_HUMAN
MP 36
DE Protein AMBP
CL simians
SQ 59
   PVPTPPDNIQVQENF(NIS)RIYGKWYNLAIGS   O   hum   Human
   ...............(...).--------....   O   hac   Chimp
   ...............(...).............   O   aga   Gorilla
   ...............(...).............   O   gra   Orangutan
   ...............(.V.).............   O   cat   Rhesus macaque
   ...............(.V.).............   O   cat   Crab-eating macaque
   ...............(.V.).............   O   cat   Baboon
   .......D.......(.V.).............   O   cat   Green monkey
   ......EG.......(.L.).............   O   sim   Marmoset
   ......EG.......(.L.).............   O   sim   Squirrel monkey
   ..L-...D.......(D..).F......V....   X   pri   Bushbaby
   ....T.ID.......(EV.)..F...F.V....   X   eua   Treeshrew
   ..----.D.......(DVT)......F...V..   X   gli   Squirrel
   ....S..D..A....(IEA)Q.....F...V..   X   gli   Lesser Egyptian jerboa
   ..-----D.......(SE.)......F...V..   X   gli   Prairie vole
   ...AL..-.......(SE.)......F.V.V..   X   gli   Chinese hamster
   ...AL..-.......(SE.)......F...V..   X   gli   Golden hamster
   .AS.L..-.......(SE.)..........V..   X   gli   Mouse
   N...L..-.......(.EA)......F...V..   X   gli   Rat
   ...M...........(DE.)......F...T..   X   gli   Naked mole-rat
   ..L.L..D.......(DE.).M.....S.....   X   gli   Guinea pig
   S..M...D.......(DE.)......F......   X   gli   Chinchilla
   .TSM...D.......(DE.)......FTV.M..   X   gli   Brush-tailed rat
   ....L..D.......(EL.)..........V..   X   gli   Rabbit
   ...NV.ET.......(DV.)..........V..   X   gli   Pika
   ..L.L.ND.......(DL.)......FHV.V..   X   lau   Pig
   ....Q.ND.......(DL.)......FLV.V..   X   lau   Alpaca
   ....Q.ND.......(DL.)......FLV.V..   X   lau   Bactrian camel
   ..T.L..D.......(DL.)......FHV.V..   X   lau   Dolphin
   ..T.L..D.......(DL.)......FHV.V..   X   lau   Killer whale
   .M..L..D.......(DL.)......FSV.V..   X   lau   Tibetan antelope
   S...L..D.......(DL.)......F.V.V..   X   lau   Cow
   .L..L..D.......(DL.)......F.V.V..   X   lau   Sheep
   .L..L..D.......(DL.)......F.V.V..   X   lau   Domestic goat
   ......AD.......(DL.)......FLV.M..   X   lau   Horse
   ....L..D.......(D..)......FLV.M..   X   lau   White rhinoceros
   ..L....D.......(D..)......FHV.M..   X   lau   Cat
   ..L....D.......(DV.)......FHV.V..   X   lau   Dog
   ..V....D.......(D..)......FHV.M..   X   lau   Ferret
   ..L....D.......(D..).M....FHV.M..   X   lau   Panda
   ..L....A.......(D..)......FHV.M..   X   lau   Pacific walrus
   ..L....A.......(D..)......FHV.M..   X   lau   Weddell seal
   ....L..D.......(D..).F....FHV.M..   X   lau   Black flying-fox
   ....L..D.......(D..).F....FHV.M..   X   lau   Megabat
   ....L..........(DV.).F....FHV....   X   lau   David's myotis bat
   ......A........(DV.).F....FHV....   X   lau   Microbat
   ....L..........(DV.).F....FHV.M..   X   lau   Big brown bat
   ....S.GS.......(DL.)......FHV....   X   lau   Hedgehog
   ..A.LSED..A....(DLP).V....FHV....   X   lau   Shrew
   ...S.SED.......(DV.)......FHV....   X   lau   Star-nosed mole
   ..S..E.DM......(DLP)......FSI....   X   afr   Elephant
   L------D.......(.L.)......F....A.   O   afr   Cape elephant shrew
   ..K..LND.......(DLP)......F.I....   X   afr   Manatee
   LA..L..D.......(DL.)......F.I....   X   afr   Cape golden mole
   .......D.......(DL.)......F.V....   X   afr   Tenrec
   .LL....D.......(DLP)......F.V....   X   afr   Aardvark
   LA.M...D.......(.L.)G------------   O   xen   Armadillo
   ..-SIQ.D.......(.L.)..F....DI.MA.   O   mar   Opossum
   ...SQ-.D.......(.L.).MF....DV..A.   O   mar   Tasmanian devil

NO 7
GN AMY2A
ID AMYP_HUMAN
MP 476
DE Pancreatic alpha-amylase
CL primates
SQ 54
   AGTYCDVISGDKING(NCT)GIKIYVSDDGKAH   O   hum   Human
   ...............(...).............   O   hac   Chimp
   ...............(...)............N   O   aga   Gorilla
   ...............(...).......N....N   O   gra   Orangutan
   .............D.(...).............   O   ape   Gibbon
   .............D.(...).......N....Q   O   cat   Rhesus macaque
   .............D.(...).......N....Q   O   cat   Crab-eating macaque
   .............D.(...).......N....Q   O   cat   Baboon
   .............D.(...)............Q   O   cat   Green monkey
   ...............(...).......N....D   O   sim   Marmoset
   ...............(...).......G.....   O   sim   Squirrel monkey
   ...............(...)...V...N..N..   O   pri   Bushbaby
   .............GS(D..).......N..N..   X   eua   Treeshrew
   G..............(D..)...V...N....Q   X   gli   Squirrel
   .............D.(...)....Q..G..R..   O   gli   Lesser Egyptian jerboa
   E............D.(Y..)...V...G..T.Q   X   gli   Prairie vole
   ............VD.(...).L.VN.GS..N..   O   gli   Chinese hamster
   ..............D(Y..).A.VN.GS....Y   X   gli   Golden hamster
   ............VD.(...).LRVN.GS.....   O   gli   Mouse
   ............V..(...).L.VN.GS.....   O   gli   Rat
   ...............(...).L..Q.KS..T.Y   O   gli   Guinea pig
   ............VG.(D..).SRV......T.E   X   gli   Chinchilla
   .............D.(Y..)...V..GG....Q   X   gli   Brush-tailed rat
   G...........SGN(S..)...VS..S....Y   X   gli   Rabbit
   G...........SGN(T..)..TVT.NS..T.Y   X   gli   Pika
   G...........VGS(S..)...V...S..T.Q   X   lau   Pig
   ..............N(S..)..Q.Q..S....Y   X   lau   Alpaca
   .............GN(S..)..Q.Q..S..N.Y   X   lau   Bactrian camel
   .............GN(Y..).......G..N.N   X   lau   Dolphin
   .............GN(Y..).......G..N.N   X   lau   Killer whale
   ............SGN(S..)..Q.S..S.....   X   lau   Tibetan antelope
   ............SGN(S..)..Q.S..S.....   X   lau   Cow
   ............SGN(R..)..Q.S..S.....   X   lau   Sheep
   ............SGN(S..)..Q.S..S.....   X   lau   Domestic goat
   ......I......D.(H..).........S..Y   X   lau   White rhinoceros
   .............D.(...).......G..N..   O   lau   Cat
   .............D.(...).....I.G..N..   O   lau   Dog
   .............D.(...).......G..N..   O   lau   Ferret
   ...............(K..)....S..A..S..   X   lau   Pacific walrus
   T............GD(...).......G..N..   O   lau   Black flying-fox
   .............GD(K..).......A....Q   X   lau   David's myotis bat
   .............GD(K..).......A.....   X   lau   Microbat
   .............GD(K..).......A.....   X   lau   Big brown bat
   .............DN(D..).T.....G..N..   X   lau   Hedgehog
   ......I......D.(K..).L.....G..N.Y   X   lau   Star-nosed mole
   ............SD.(...).......S....N   O   afr   Elephant
   ............VGN(S..)..TVN..G..N..   X   afr   Cape elephant shrew
   .............D.(...).......S....N   O   afr   Manatee
   ............VGD(...)....H..G..N.Y   O   afr   Cape golden mole
   .............GD(...).......G....N   O   afr   Tenrec
   .............G.(...).......G....S   O   afr   Aardvark
   .............GD(...).......G..N..   O   xen   Armadillo
   .............DN(...)....T...S.N..   O   mar   Opossum
   .............DN(Q..).......G..R.Y   X   mar   Tasmanian devil

NO 8
GN APMAP
ID APMAP_HUMAN
MP 196
DE Adipocyte plasma membrane-associated protein
CL humans
SQ 61
   EVKLLLSSETPIEGK(NMS)FVNDLTVTQDGRK   O   hum   Human
   ...............(K..).............   X   hac   Chimp
   ...............(K..).............   X   aga   Gorilla
   ...............(K..).............   X   gra   Orangutan
   ...............(K..).............   X   ape   Gibbon
   ...............(K..).............   X   cat   Rhesus macaque
   ...............(K..).............   X   cat   Crab-eating macaque
   ...............(K..).............   X   cat   Baboon
   ...............(K..).............   X   cat   Green monkey
   ...............(K..).............   X   sim   Marmoset
   ...........V...(K..).............   X   sim   Squirrel monkey
   ...............(K..)......I......   X   pri   Bushbaby
   ........D......(KL.)......I.R....   X   eua   Treeshrew
   ...........V...(K..)......I.R....   X   gli   Squirrel
   Q..V...........(K..)......I.R....   X   gli   Lesser Egyptian jerboa
   A..............(K..)........R....   X   gli   Prairie vole
   A.........L....(K..)........R....   X   gli   Chinese hamster
   A..............(K..)........R....   X   gli   Golden hamster
   S..............(K..)........R....   X   gli   Mouse
   S..............(K..)......I.R....   X   gli   Rat
   ..............R(K..).....AI.R....   X   gli   Naked mole-rat
   Q........M....R(K..)......I.R....   X   gli   Guinea pig
   Q.............R(K..).....AI.R....   X   gli   Chinchilla
   ..............R(K..).....AI.R....   X   gli   Brush-tailed rat
   ........D......(K..)......I.R....   X   gli   Rabbit
   .........V.....(KL.).I...A..R....   X   gli   Pika
   ..............R(KL.)........R....   X   lau   Pig
   ...Q..........R(RL.)........R....   X   lau   Alpaca
   ...Q..........R(RL.).....A..R....   X   lau   Bactrian camel
   ..............R(K..)........R....   X   lau   Dolphin
   ..............R(K..)........R....   X   lau   Killer whale
   ..............R(K..).L......R....   X   lau   Tibetan antelope
   ..............R(K..).L......R....   X   lau   Cow
   ..............R(K..).L......R....   X   lau   Sheep
   ..............R(K..).L......R....   X   lau   Domestic goat
   .........I....R(K..)......I......   X   lau   Horse
   ..R...........R(K..)......I......   X   lau   White rhinoceros
   ..............R(K..).............   X   lau   Cat
   .....V...I....R(K..)......I....K.   X   lau   Dog
   .....I........R(K..)......I....K.   X   lau   Ferret
   .....I........R(K..)......I......   X   lau   Panda
   .....I........R(K..)......I......   X   lau   Pacific walrus
   .....I........R(K..)......I......   X   lau   Weddell seal
   ......Y.D.L...R(K..)......I.R....   X   lau   Black flying-fox
   ........D.L...R(K..)......I.RV...   X   lau   Megabat
   ........D.....R(K.A)......I.R....   X   lau   David's myotis bat
   ........DI....R(K..)......I.R....   X   lau   Microbat
   ........D.....R(K.A)......I.R....   X   lau   Big brown bat
   K..........V..R(K..).L....I.R..K.   X   lau   Hedgehog
   ..T........V..R(K..)........R....   X   lau   Shrew
   ..............R(K..)...........K.   X   lau   Star-nosed mole
   ...V...........(K..)......I.R....   X   afr   Elephant
   ...M...........(KI.)......I.R....   X   afr   Manatee
   ........D......(K..)......I......   X   afr   Cape golden mole
   ...I...........(K..)......I......   X   afr   Tenrec
   ...............(K..)......I......   X   afr   Aardvark
   ........DM.....(K..)......I.K....   X   xen   Armadillo
   R..Q....Q......(K..)......I......   X   mar   Opossum
   R..H....KI.....(K..)......I.K....   X   mar   Tasmanian devil
   ----.....I.....(K..)......I......   X   mar   Wallaby
   D.RQ....Q......(K..).....AI.R....   X   mon   Platypus

NO 9
GN APOB
ID APOB_HUMAN
MP 3411
DE Apolipoprotein B-100
CL simians
SQ 62
   ATALSLSNKFVEGSH(NST)VSLTTKNMEVSVA   O   hum   Human
   ...............(...).............   O   hac   Chimp
   ...............(...)......Y..A...   O   aga   Gorilla
   ...............(...)....K....A...   O   gra   Orangutan
   ...............(...)....K....A...   O   ape   Gibbon
   ...............(...)....K....A...   O   cat   Rhesus macaque
   ...............(...)....K....A...   O   cat   Crab-eating macaque
   ...............(...)....K....A...   O   cat   Baboon
   ...............(...)....K....A...   O   cat   Green monkey
   ........R......(...)....K....A..V   O   sim   Marmoset
   ...............(...)....K....A..V   O   sim   Squirrel monkey
   .M....N..Y...N.(D..)I...K....A..T   X   pri   Bushbaby
   .A.............(D..)I...K....A..T   X   eua   Treeshrew
   ...............(D..)I..IE..L.A..T   X   gli   Squirrel
   .............N.(D..)I...K...DA..K   X   gli   Lesser Egyptian jerboa
   ...V..T....K...(D..)I..NK..V.A..K   X   gli   Prairie vole
   ...I..T....K.N.(D..)I...K....AL.K   X   gli   Chinese hamster
   ...I..T....K.N.(D..)F...K....A..K   X   gli   Golden hamster
   ...V..T....K...(D..)I...K....A..R   X   gli   Mouse
   ...V..T...LK...(D..)I...K....A..K   X   gli   Rat
   ...............(D..)I.I.K.I..A.AT   X   gli   Naked mole-rat
   ...............(D..)I.F.K....A.LT   X   gli   Guinea pig
   ...............(D..)..F.K....A.AT   X   gli   Chinchilla
   ........T......(D..)I...K.T..A.AT   X   gli   Brush-tailed rat
   ..T...N...LG.T.(D..)I...K....A.ST   X   gli   Rabbit
   ......N...LG.T.(D..)I...K.S.D..L.   X   gli   Pika
   ..........M..N.(D..)I.F.K..VDA.LT   X   lau   Pig
   ........R..A...(D..)I.F.K..VDA..T   X   lau   Alpaca
   ........R..A...(D..)I.F.K.SVDA..T   X   lau   Bactrian camel
   .........L...N.(DN.)I.F.K...DA.MT   X   lau   Dolphin
   .........L...N.(DN.)I.F.K...DA.MT   X   lau   Killer whale
   ........R....N.(D..)I.F.K...DA..T   X   lau   Tibetan antelope
   ........R....N.(D..)..F.K..VDA..T   X   lau   Cow
   .A......R....N.(D..)I.F.K...DA..T   X   lau   Sheep
   .A......R....N.(D..)I.F.K...DA..T   X   lau   Domestic goat
   .............K.(E.A)I..IK.T..A..T   X   lau   Horse
   .............N.(D..)I.I.K....A..M   X   lau   White rhinoceros
   ..........M..N.(D..)I...K.S..A..T   X   lau   Cat
   .............N.(D..)I...K....A..T   X   lau   Dog
   ..........M..N.(D..)F...K....A..T   X   lau   Ferret
   .............N.(D..)I...K....A..T   X   lau   Panda
   ...F..T......N.(DN.)I...K....A..T   X   lau   Pacific walrus
   ......T......N.(D..)....R....A..T   X   lau   Weddell seal
   ...F.........N.(D..)I...K...DA..T   X   lau   Black flying-fox
   -----........N.(D..)I...K...DA..T   X   lau   Megabat
   .........Y...N.(D..)I...K.T..A..T   X   lau   David's myotis bat
   .........Y...N.(D..)I...K.T..A..T   X   lau   Microbat
   .........H...N.(D..)I...M.TI.AT.T   X   lau   Big brown bat
   ......N......K.(D..)I...K....A..T   X   lau   Hedgehog
   ........EY...K.(D..)I..AK....A...   X   lau   Shrew
   ..T.....E..G...(...)I...K....A..T   O   lau   Star-nosed mole
   ...............(D..)I...K....T..T   X   afr   Elephant
   ........T......(D..)I...K..I.A..T   X   afr   Cape elephant shrew
   ...............(D..)I...KR...A..T   X   afr   Manatee
   ...............(D..)I...K....A..T   X   afr   Cape golden mole
   ...W...........(D..)M...R....A.M.   X   afr   Tenrec
   ...............(D..)L...K....A..T   X   afr   Aardvark
   ..........L....(D..)I..NK....A..T   X   xen   Armadillo
   .M....T.R....N.(D..)....KR...A..T   X   mar   Opossum
   ......NS.....N.(D..)L...KR...A..T   X   mar   Tasmanian devil
   ......N.R....N.(D..)I...K....A..S   X   mar   Wallaby
   ......N......N.(D..)....RR...A..T   X   mon   Platypus

NO 10
GN APOM
ID APOM_HUMAN
MP 135
DE Apolipoprotein M
CL simians
SQ 61
   KTELFSSSCPGGIML(NET)GQGYQRFLLYNRS   O   hum   Human
   ...............(...).............   O   hac   Chimp
   ...............(...).............   O   aga   Gorilla
   ...............(...)........F....   O   gra   Orangutan
   ...............(...).............   O   ape   Gibbon
   ...............(...).R...........   O   cat   Rhesus macaque
   ...............(...).............   O   cat   Crab-eating macaque
   ...............(...).............   O   cat   Baboon
   ...............(...).............   O   sim   Marmoset
   ...............(...).............   O   sim   Squirrel monkey
   ...............(K..).............   X   pri   Bushbaby
   ...............(K..)........F....   X   eua   Treeshrew
   ...............(K..).............   X   gli   Squirrel
   ..........D..V.(K..)........F...L   X   gli   Lesser Egyptian jerboa
   ..D............(K..).E...........   X   gli   Prairie vole
   ..D............(K..).............   X   gli   Chinese hamster
   E.D............(K..).R...........   X   gli   Golden hamster
   ..D............(K..).............   X   gli   Mouse
   ..D...I........(K..).............   X   gli   Rat
   R......P.......(K.R)............A   X   gli   Naked mole-rat
   R....A....R....(K..).............   X   gli   Guinea pig
   .....A.........(R.M).............   X   gli   Chinchilla
   ...I.A.........(K.M).............   X   gli   Brush-tailed rat
   ...............(K..)........F....   X   gli   Rabbit
   ...F...........(K..)........F....   X   gli   Pika
   ..K....T.......(K..).............   X   lau   Pig
   ..K............(K..).............   X   lau   Alpaca
   ..K............(K..).............   X   lau   Bactrian camel
   ..K....A.......(K..).............   X   lau   Dolphin
   ..K....A.......(K..).............   X   lau   Killer whale
   ..K....A.......(K..).............   X   lau   Tibetan antelope
   ..K....A.......(K..).............   X   lau   Cow
   ..K....A.......(K..).............   X   lau   Sheep
   ..K....A.......(K..).............   X   lau   Domestic goat
   ..K............(K..).............   X   lau   Horse
   ..K............(K..)............L   X   lau   White rhinoceros
   ..K............(K.S).............   X   lau   Cat
   R.M............(K.S).H...........   X   lau   Dog
   ..K............(K.S).............   X   lau   Ferret
   ..K..........V.(K.S)......Y......   X   lau   Panda
   ..K............(K.S).............   X   lau   Pacific walrus
   ..K............(K.S).............   X   lau   Weddell seal
   ..K............(K..)............L   X   lau   Black flying-fox
   ..K............(K..)............L   X   lau   Megabat
   ...............(K..).............   X   lau   David's myotis bat
   ...............(K..)........F....   X   lau   Microbat
   ..Q..........L.(K..)....R........   X   lau   Big brown bat
   M.K.......D..I.(K..)........F...L   X   lau   Hedgehog
   M.K.......D....(K..).............   X   lau   Shrew
   ..K..T....D....(K..)........F....   X   lau   Star-nosed mole
   ...............(K.M).............   X   afr   Elephant
   ...............(K.M).H...........   X   afr   Cape elephant shrew
   ...............(K.M).............   X   afr   Manatee
   ...............(K.M).............   X   afr   Cape golden mole
   ...............(K.M).............   X   afr   Tenrec
   ...............(K.M).............   X   afr   Aardvark
   ..K..N....D..T.(K..).............   X   xen   Armadillo
   .......F..D....(R..).............   X   mar   Opossum
   .......F..D....(R..)...N........F   X   mar   Tasmanian devil
   ..........D....(R..).............   X   mar   Wallaby
   ......AP.AES.I.(K..)..D.E...M.---   X   mon   Platypus

NO 11
GN AREG
ID AREG_HUMAN
MP 119
DE Amphiregulin
CL simians
SQ 60
   VEQVVKPPQNKTESE(NTS)DKPKRKKKGGKNG   O   hum   Human
   ........K......(...).............   O   hac   Chimp
   ........K......(...).............   O   gra   Orangutan
   ........K......(...).............   O   ape   Gibbon
   ........K......(...).............   O   cat   Rhesus macaque
   ........K......(...).............   O   cat   Crab-eating macaque
   ........K......(...).............   O   cat   Baboon
   ........K......(...).............   O   cat   Green monkey
   .......QK......(...).............   O   sim   Marmoset
   .......QK......(...).............   O   sim   Squirrel monkey
   .......KK......(K..).............   X   pri   Bushbaby
   .......KK.....K(K..)..T.....E..S.   X   eua   Treeshrew
   ....I..KK......(K..)...........G.   X   gli   Squirrel
   ....I..KK.R..G.(KAT)......R......   X   gli   Lesser Egyptian jerboa
   ....I..KK....EA(K..)...........S.   X   gli   Prairie vole
   ....I..KK....GD(R..).........S.S.   X   gli   Chinese hamster
   ....I..KK..A.GD(K..).....R.....S.   X   gli   Golden hamster
   ....I..KK....G.(KST)E............   X   gli   Mouse
   ....I..KE....G.(KS.)E..........G.   X   gli   Rat
   .......KK....G.(K..).........S...   X   gli   Naked mole-rat
   .......KK...QG.(K..).........N...   X   gli   Guinea pig
   .......KK....G.(K..).........N...   X   gli   Chinchilla
   .......KK......(K..).........N...   X   gli   Brush-tailed rat
   ....I..KK..A...(K..).............   X   gli   Rabbit
   ....I..KK..A...(K..)....K........   X   gli   Pika
   .......KR.R....(...)...........S.   O   lau   Pig
   .......KK......(K..)...-.........   X   lau   Alpaca
   .......KK......(K..).............   X   lau   Bactrian camel
   .......KKD.N...(K..).............   X   lau   Dolphin
   .......KKD.N...(K..).............   X   lau   Killer whale
   .......KK......(K..).............   X   lau   Tibetan antelope
   .......KK......(K..).............   X   lau   Cow
   .......KK......(K..).............   X   lau   Sheep
   .......KK......(K..).............   X   lau   Domestic goat
   .......KK......(K..).............   X   lau   Horse
   .......KK......(K..).R...........   X   lau   White rhinoceros
   .K*.IM.MK.D.QD.(EI.)VE...........   X   lau   Cat
   .......MK......(K..)...........S.   X   lau   Dog
   .......MK......(K..)...........S.   X   lau   Ferret
   ....I..MK....G.(K..)...........S.   X   lau   Panda
   .......MK.R....(K..)...........S.   X   lau   Pacific walrus
   .......MK.R....(K..)...........S.   X   lau   Weddell seal
   .......KK......(K..)..S......S...   X   lau   Black flying-fox
   .......KK......(K..)..S...N..S...   X   lau   Megabat
   ....F..KE......(..T).........P..R   O   lau   David's myotis bat
   .......KE......(..T).........Q..R   O   lau   Microbat
   .......KG....N.(..T).........H.GR   O   lau   Big brown bat
   .......KK......(K..)EN..........R   X   lau   Hedgehog
   ....I..KK....G.(K..).............   X   lau   Shrew
   ....I..KK..A..K(K..)..T...R....D.   X   lau   Star-nosed mole
   ........H......(Q..)S.......A....   X   afr   Elephant
   ........R......(K..)N.......S....   X   afr   Cape elephant shrew
   .......T......G(K..)N............   X   afr   Manatee
   ...............(K..)N.....R......   X   afr   Cape golden mole
   ....I...R.....G(K..)N.......D....   X   afr   Tenrec
   ....I...K......(K..)N......NS....   X   afr   Aardvark
   ....I..EK...K..(K..)G...........R   X   xen   Armadillo
   .GRL...KK.R....(KN.)..TR...N-----   X   mar   Opossum
   .GRL...KK.R....(KN.)S.TG...F-----   X   mar   Tasmanian devil
   ..RLE..RR...D..(KNP).R.....NR....   X   mon   Platypus

NO 12
GN ASPH
ID ASPH_HUMAN
MP 452
DE Aspartyl/asparaginyl beta-hydroxylase
CL primates
SQ 61
   RGSLLTLQRLVQLFP(NDT)SLKNDLGVGYLLI   O   hum   Human
   ...............(...).............   O   hac   Chimp
   ...............(...).............   O   aga   Gorilla
   ...............(...).............   O   gra   Orangutan
   ...............(...).............   O   ape   Gibbon
   ...............(...).............   O   cat   Rhesus macaque
   ...............(...).............   O   cat   Crab-eating macaque
   ...............(...).............   O   cat   Baboon
   ...............(...).............   O   cat   Green monkey
   ...........H...(...).............   O   sim   Marmoset
   ...........H...(...).............   O   sim   Squirrel monkey
   ...............(...).............   O   pri   Bushbaby
   ........K......(S..).............   X   eua   Treeshrew
   ...............(S..).............   X   gli   Squirrel
   ...............(S..)............M   X   gli   Lesser Egyptian jerboa
   ...............(G..)A...........M   X   gli   Prairie vole
   ...............(S..)T...........M   X   gli   Chinese hamster
   ...............(G..)T...........M   X   gli   Golden hamster
   ...............(S..)T...........L   X   gli   Mouse
   ...............(S..)T...........M   X   gli   Rat
   ...............(S..)............M   X   gli   Naked mole-rat
   ...............(S..).............   X   gli   Guinea pig
   ...............(S..).............   X   gli   Chinchilla
   ...............(S..).............   X   gli   Brush-tailed rat
   ...............(...).............   O   gli   Rabbit
   ...............(...).............   O   gli   Pika
   ........K......(D..).............   X   lau   Pig
   ........K......(D..)A............   X   lau   Alpaca
   ........K......(D..)A...........M   X   lau   Bactrian camel
   ........K......(D..).............   X   lau   Dolphin
   ........K......(D..).............   X   lau   Killer whale
   ........K......(D..).............   X   lau   Tibetan antelope
   ........K......(D..)A............   X   lau   Cow
   ........K......(D..).............   X   lau   Sheep
   ........K......(D..).............   X   lau   Domestic goat
   ........K......(D..).............   X   lau   Horse
   ........K......(D..).............   X   lau   White rhinoceros
   ....I...K......(D..).............   X   lau   Cat
   ....I...K......(D.M).............   X   lau   Dog
   ....I...K......(D.M).............   X   lau   Ferret
   ....M...K......(D.M).............   X   lau   Panda
   ....I...K......(D.M).............   X   lau   Pacific walrus
   ....I...K......(D.M).............   X   lau   Weddell seal
   ....V...K......(D..).............   X   lau   Black flying-fox
   ....V...K......(D..).............   X   lau   Megabat
   ....A...K......(D..).............   X   lau   David's myotis bat
   ....A...K......(D..).............   X   lau   Microbat
   ....A...K......(D..)............M   X   lau   Big brown bat
   ...............(D..)............M   X   lau   Hedgehog
   ...............(DE.)............L   X   lau   Shrew
   ...............(E..)............L   X   lau   Star-nosed mole
   ....M..K.......(...).............   O   afr   Elephant
   ...............(S..)............M   X   afr   Cape elephant shrew
   ....M..K.......(...).............   O   afr   Manatee
   ...............(...).............   O   afr   Cape golden mole
   K...M..........(...).............   O   afr   Tenrec
   ...............(..S).............   O   afr   Aardvark
   ....H..........(S..).............   X   xen   Armadillo
   ....V...K..H...(.E.).F...........   O   mar   Opossum
   ....V...K..H...(.E.).F...........   O   mar   Tasmanian devil
   .R.I...........(...).F..........V   O   mon   Platypus

NO 13
GN AZGP1
ID ZA2G_HUMAN
MP 109
DE Zinc-alpha-2-glycoprotein
CL primates
SQ 50
   EDIFMETLKDIVEYY(NDS)NGSHVLQGRFGCE   O   hum   Human
   ...............(...).............   O   hac   Chimp
   ...............(...).............   O   aga   Gorilla
   ...............(.A.).............   O   gra   Orangutan
   ........NN.M...(...)....I........   O   ape   Gibbon
   ........N......(...)....N.H......   O   cat   Rhesus macaque
   ........N......(...)....N.H......   O   cat   Crab-eating macaque
   ........N......(...)....N.H......   O   cat   Baboon
   ........N......(...)....N.H......   O   cat   Green monkey
   .....Q..N..T...(...)....T........   O   sim   Marmoset
   ........N..MD..(...)....T........   O   sim   Squirrel monkey
   .S.YL...Q..MAF.(...)....I..E.L...   O   pri   Bushbaby
   .NL.....G..LD..(..N)..F....EA....   X   eua   Treeshrew
   .E..LV.....MD.H(K..)R...TF..M....   X   gli   Squirrel
   .E..L......MD..(K..)Q...TF..M....   X   gli   Lesser Egyptian jerboa
   .E..LV....VMD..(E..)A...TF..M....   X   gli   Prairie vole
   .E..LV.....MD..(K.R)E...T...M.S..   X   gli   Naked mole-rat
   QE..LV.....MD..(K..)E...TF..M....   X   gli   Guinea pig
   .E..LV.....MD..(K.R)E...TF..M....   X   gli   Chinchilla
   .E..LVA....MD..(K.R)E...TF..M....   X   gli   Brush-tailed rat
   .E..L.....AMN..(KE.)T...TF..M....   X   gli   Rabbit
   .K..L...T.AMN..(QA.)K...TF..M....   X   gli   Pika
   .....A..R..MD..(K.R)E...TF.......   X   lau   Pig
   .....V.....MDH.(K.A)E...TF..M....   X   lau   Alpaca
   .....V.....MDH.(K.G)E...TF..M....   X   lau   Bactrian camel
   ........S..MD..(K.R)E...TF..A....   X   lau   Tibetan antelope
   ........S..MD..(K.R)E...TF..A....   X   lau   Cow
   ........S..MD..(K.R)E...TF..A....   X   lau   Sheep
   ........S..MD..(K.R)E...TF..A....   X   lau   Domestic goat
   .....V..E..MD..(K.R)E...TF..L....   X   lau   Horse
   .....V.....MD..(K.R)E...T...L....   X   lau   White rhinoceros
   .S......Q..MD..(K.R)E...TF.......   X   lau   Cat
   .....V.....M...(K.K)E...TF..M....   X   lau   Dog
   .K...A..T..MD..(K.K)E...TF..M....   X   lau   Ferret
   .....V.....MD..(K.T)E.A.TF..M....   X   lau   Pacific walrus
   G.F.LKN....MD.K(K.R).--.TM.EK...K   X   lau   Black flying-fox
   G.F.LKN....MG..(K.T).--.TM.EKY..K   X   lau   David's myotis bat
   Q.........VMG..(R.R).--.TFK.T....   X   lau   Microbat
   Q....K....VMG..(R.R).--.TFK.T....   X   lau   Big brown bat
   G.F.L.A...A.N..(K.P)EAP.TF.......   X   lau   Shrew
   .........N.MD..(K.G)K...TF..V....   X   lau   Star-nosed mole
   .E...V.....MD..(K.G)E...TF..M....   X   afr   Elephant
   .E...V..Q..MS..(K.K)E...TF..M....   X   afr   Cape elephant shrew
   .E...V..N..MD..(E.R)E...TF..M....   X   afr   Manatee
   .....V..N..MD..(K.R)E...TF..M....   X   afr   Tenrec
   .E..LV.....MD..(K.G)E...TF..M....   X   afr   Aardvark
   .E...A..D...D..(K..)R...TF..M...A   X   xen   Armadillo
   ..FVLDNMQN.LD..(..G)....IF......K   X   mar   Opossum
   ..FVL.NMQN.TD..(..G)K...IF......Q   X   mar   Tasmanian devil
   DHM.IID.WT.MDNH(SQG)Q...I..VLL...   X   mar   Wallaby

NO 14
GN AZGP1
ID ZA2G_HUMAN
MP 112
DE Zinc-alpha-2-glycoprotein
CL primates
SQ 46
   FMETLKDIVEYYNDS(NGS)HVLQGRFGCEIEN   O   hum   Human
   ...............(...).............   O   hac   Chimp
   ...............(...).............   O   aga   Gorilla
   .............A.(...).............   O   gra   Orangutan
   .....NN.M......(...).I...........   O   ape   Gibbon
   .....N.........(...).N.H.........   O   cat   Rhesus macaque
   .....N.........(...).N.H.........   O   cat   Crab-eating macaque
   .....N.........(...).N.H.........   O   cat   Baboon
   .....N.........(...).N.H.........   O   cat   Green monkey
   ..Q..N..T......(...).T.........Q.   O   sim   Marmoset
   .....N..MD.....(...).T.........Q.   O   sim   Squirrel monkey
   YL...Q..MAF....(...).I..E.L...VH.   O   pri   Bushbaby
   .....G..LD....N(..F)....EA....LQ.   X   eua   Treeshrew
   .LV.....MD.HK..(R..).TF..M.....Q.   X   gli   Squirrel
   .L......MD..K..(Q..).TF..M....LGS   X   gli   Lesser Egyptian jerboa
   .LV....VMD..E..(A..).TF..M....LS.   X   gli   Prairie vole
   .LV.....MD..K.R(E..).T...M.S...Q.   X   gli   Naked mole-rat
   .LV.....MD..K..(E..).TF..M.....Q.   X   gli   Guinea pig
   .LV.....MD..K.R(E..).TF..M.....Q.   X   gli   Chinchilla
   .LVA....MD..K.R(E..).TF..M.....Q.   X   gli   Brush-tailed rat
   .L.....AMN..KE.(T..).TF..M....LQ.   X   gli   Rabbit
   .L...T.AMN..QA.(K..).TF..M....LHR   X   gli   Pika
   ..A..R..MD..K.R(E..).TF.......LR.   X   lau   Pig
   ..V.....MDH.K.A(E..).TF..M....LRR   X   lau   Alpaca
   ..V.....MDH.K.G(E..).TF..M....LRR   X   lau   Bactrian camel
   .....S..MD..K.R(E..).TF..A....LR.   X   lau   Tibetan antelope
   .....S..MD..K.R(E..).TF..A....LR.   X   lau   Cow
   .....S..MD..K.R(E..).TF..A....LR.   X   lau   Sheep
   .....S..MD..K.R(E..).TF..A....LR.   X   lau   Domestic goat
   ..V..E..MD..K.R(E..).TF..L....LR.   X   lau   Horse
   ..V.....MD..K.R(E..).T...L....LR.   X   lau   White rhinoceros
   .....Q..MD..K.R(E..).TF.......LL.   X   lau   Cat
   ..V.....M...K.K(E..).TF..M....LQ.   X   lau   Dog
   ..A..T..MD..K.K(E..).TF..M....LR.   X   lau   Ferret
   ..V.....MD..K.T(E.A).TF..M....LW.   X   lau   Pacific walrus
   .L.A...A.N..K.P(EAP).TF.......LQ.   X   lau   Shrew
   ......N.MD..K.G(K..).TF..V....LRS   X   lau   Star-nosed mole
   ..V.....MD..K.G(E..).TF..M....LW.   X   afr   Elephant
   ..V..Q..MS..K.K(E..).TF..M....LR.   X   afr   Cape elephant shrew
   ..V..N..MD..E.R(E..).TF..M....LR.   X   afr   Manatee
   ..V..N..MD..K.R(E..).TF..M....VR.   X   afr   Tenrec
   .LV.....MD..K.G(E..).TF..M....LR.   X   afr   Aardvark
   ..A..D...D..K..(R..).TF..M...ALW.   X   xen   Armadillo
   VLDNMQN.LD....G(...).IF......KLCG   O   mar   Opossum
   VL.NMQN.TD....G(K..).IF......QLCG   X   mar   Tasmanian devil
   .IID.WT.MDNHSQG(Q..).I..VLL...LGD   X   mar   Wallaby

NO 15
GN BCAM
ID BCAM_HUMAN
MP 439
DE Basal cell adhesion molecule
CL simians
SQ 58
   CEASLPTVPVLSRTQ(NFT)LLVQGSPELKTAE   O   hum   Human
   ...............(...).............   O   hac   Chimp
   ...............(...).............   O   aga   Gorilla
   ...............(...)....---------   O   gra   Orangutan
   ...-.....-.....(...).........R...   O   ape   Gibbon
   ...............(...).....L...RA..   O   cat   Rhesus macaque
   ...............(...).....L...RA..   O   cat   Crab-eating macaque
   ...............(...).....L...RA..   O   cat   Baboon
   ...............(...).....L...RA..   O   cat   Green monkey
   ..............R(...)..........ALD   O   sim   Marmoset
   ..............R(...)..........A.D   O   sim   Squirrel monkey
   ...FM....L.....(S.E).........RAE.   X   pri   Bushbaby
   ....MS...L..Q..(S.K).........RPE.   X   eua   Treeshrew
   ....MS...F...VR(S.K).........RAE.   X   gli   Squirrel
   ....T....L....E(S.Q).M.......SPQ.   X   gli   Lesser Egyptian jerboa
   ....T....L.....(S.Q).V...T....PN.   X   gli   Prairie vole
   ....T.I..L.....(S.Q)...E.P..I.PN.   X   gli   Chinese hamster
   ...DT.I..L.....(S.Q).V...P..IRPN.   X   gli   Golden hamster
   ....T....L.....(S.Q).I...A....PN.   X   gli   Mouse
   ....T....L.....(S.Q).V...A....PN.   X   gli   Rat
   ....M.S..LV....(S.R).........RAK.   X   gli   Naked mole-rat
   ....V.S..L.....(S.K).........RAK.   X   gli   Guinea pig
   ....V.G..L.....(S.R).........RAK.   X   gli   Chinchilla
   ....V.S..L.....(S.R).V........AK.   X   gli   Brush-tailed rat
   ....V..M.L.T..R(A.Q).....T...RAE.   X   gli   Pika
   ....M....L....R(S.K).....P...RAE.   X   lau   Pig
   ...FM....L....R(S.K).....P...RAE.   X   lau   Alpaca
   ...FMS...L....R(S.K).....P...RAE.   X   lau   Bactrian camel
   ....M.........R(S.E).....P...RAE.   X   lau   Dolphin
   ....M.........R(S.E).....P...RAE.   X   lau   Killer whale
   ...YM.KI.L....R(S.R).....T....AK.   X   lau   Tibetan antelope
   ...YM.RI.L....R(S.R).....T....AK.   X   lau   Cow
   ...YM.NI.L....R(S.R).....T....AK.   X   lau   Sheep
   ...YM.NI.L....R(S.R).....T....AK.   X   lau   Domestic goat
   ....M.R..L....R(S.K)...E.P..V.PE.   X   lau   Horse
   ....M....L...A.(S.K).....P...RAE.   X   lau   Cat
   ....M....L.....(S.R).....P.Q.RAE.   X   lau   Dog
   ....M....L.....(S.K).....P.Q.SAE.   X   lau   Ferret
   ....M....L.....(S.N).....P.Q.SAE.   X   lau   Panda
   ....M....L.....(S.K).....P.Q.SPE.   X   lau   Pacific walrus
   ....M....L.....(S.K).....P.Q.SPE.   X   lau   Weddell seal
   ....M....L...NR(ILR)...E.....RPE.   X   lau   Black flying-fox
   ....M....L...NR(ILR)...E.....RPE.   X   lau   Megabat
   ...YT....L....R(VLK)...E.P.D..PE.   X   lau   David's myotis bat
   ...YT....L....R(ALK)...E.V.D..PEK   X   lau   Big brown bat
   ....V....L....R(S.R).....L...RPE.   X   lau   Hedgehog
   ....M....L....R(S.K).........RPE.   X   lau   Shrew
   ....M....L....R(GVK).....P...RQE.   X   lau   Star-nosed mole
   ....MSM..L.....(S.K).....L...RAD.   X   afr   Elephant
   ....MS...L.....(S.K)........VREV.   X   afr   Cape elephant shrew
   ....MS...L.....(S.K)..........AE.   X   afr   Manatee
   ....MS...L.....(S.R).........WAE.   X   afr   Cape golden mole
   ....VS...L.....(A.K).H.......MAE.   X   afr   Tenrec
   ....MS...L..H.G(S.K).........RAE.   X   afr   Aardvark
   --------------.(S.Q).....L...RPET   X   xen   Armadillo
   ....V.KL.G.T..R(QLQ).F...E....PD.   X   mar   Tasmanian devil
   ....V.KL.G.T..R(.LQ).....K....PE.   X   mar   Wallaby
   ....V.A..R.N...(TLQ)...T.K..I.EEA   X   mon   Platypus

NO 16
GN BTN3A1
ID BT3A1_HUMAN
MP 115
DE Butyrophilin subfamily 3 member A1
CL primates
SQ 50
   LRDGITAGKAALRIH(NVT)ASDSGKYLCYFQD   O   hum   Human
   ...............(...).............   O   hac   Chimp
   ...............(...).............   O   aga   Gorilla
   ...............(...).............   O   gra   Orangutan
   ..........T....(...).............   O   ape   Gibbon
   ...............(...).............   O   cat   Rhesus macaque
   ...............(...).............   O   cat   Crab-eating macaque
   ...............(...).............   O   cat   Green monkey
   .....A.........(...).............   O   sim   Marmoset
   .....A.........(...).............   O   sim   Squirrel monkey
   ...D......T....(...).....N.......   O   pri   Bushbaby
   ....LA.........(G.R)...N.........   X   gli   Squirrel
   VTH.LR..R.....R(G.R)...D.E.R.F.R.   X   gli   Lesser Egyptian jerboa
   VT..LPD.R.T...R(G.R)V..Q.E.R.F.K.   X   gli   Prairie vole
   VSTD.SK.RV..I..(...).Y.N.I.Y....E   O   gli   Chinese hamster
   MSTD.SK.RV..I..(...).Y.N.I.C....E   O   gli   Golden hamster
   ATA.LLD.R.T.L.R(D.R)V..Q.E.R.L.K.   X   gli   Mouse
   VTARLLD.L.T...R(G.R)V..Q.Q.R.FLK.   X   gli   Rat
   VTEN....H.MV...(GIR)TF.E.V.G.S.R.   X   gli   Naked mole-rat
   VT.N....H......(DIR).F.E.V.GAFL.-   X   gli   Guinea pig
   VA..MAQ.R..A...(A.R).A.D.D.G.L.R.   X   gli   Chinchilla
   VT.N....H......(GIR)VF.E.V.G.S.K.   X   gli   Brush-tailed rat
   VPAVLAG.R.....R(A.R)...D.E.R.I.RK   X   gli   Rabbit
   VPEELAE.I.....R(GIR)...D.E.R.V.RE   X   gli   Pika
   ...D.....V....R(..R).....N.......   X   lau   Alpaca
   ...D.....V....R(..R).....N.......   X   lau   Bactrian camel
   ............Q..(S.R).....N....V..   X   lau   Killer whale
   VE.H.AE.SV.....(..R).....N.......   X   lau   Tibetan antelope
   ...D......V....(..R).....N.......   X   lau   Cow
   VE.H.AE.SV.....(..R).....N.......   X   lau   Sheep
   VE.H.AE.SV.....(..R).....N.-.....   X   lau   Domestic goat
   ...DV.E...T...Y(D.R)...R.S.R....G   X   lau   Horse
   ......E...T...Y(D.R).....S.......   X   lau   White rhinoceros
   .YGD.A..RV.V...(R.R)...D.E.R.L...   X   lau   Cat
   VDAD.A..RV.V.L.(R.R)...D.E.R.S...   X   lau   Dog
   VDRD.A..RV.V...(Q.R)...D.E.R.F...   X   lau   Ferret
   VARD.A..RV.V...(R.R)...D.E.R.F...   X   lau   Pacific walrus
   VQ.D.AQ.RV....R(P.Q)...D.E.R.F..N   X   lau   Black flying-fox
   ..ED.AE........(K.R)T....T.Q...-.   X   lau   Microbat
   VE.D.NV.RV.MK..(G.K)VA.G.E.R.F..N   X   lau   Hedgehog
   V..NLSQ.RM.V...(GIK).K.A.E.R.F.EK   X   lau   Shrew
   VDRHVPS.C..V...(G.K)...D.E.R.F.EN   X   lau   Star-nosed mole
   VK.N....RV.V...(P.R)...D.E.R.F.RR   X   afr   Elephant
   VN.N....RV.V...(G.R)...D.E.R.F.RE   X   afr   Manatee
   V.Y....E.V.V-.Y(..R).....N-MH.L..   X   afr   Cape golden mole
   AK.N....H..V...(G.R)P..D.E.R.F.RE   X   afr   Tenrec
   VK.N....HV.V...(G.R)V..D.E.W.F.RE   X   afr   Aardvark
   ...SVAER.V....S(..R).....H.......   X   xen   Armadillo
   IK.A..N.NV..K.R(.IR)V..G.Q.Q...EK   X   mar   Opossum
   VQ.N..E.NV.VT..(..R)...D.Q.R.F.K.   X   mar   Tasmanian devil

NO 17
GN C1RL
ID C1RL_HUMAN
MP 147
DE Complement C1r subcomponent-like protein
CL simians
SQ 60
   GRSLRLTFRTQPSSE(NKT)AHLHKGFLALYQT   O   hum   Human
   ......I........(...).............   O   hac   Chimp
   ...............(...)............S   O   aga   Gorilla
   ........C......(...).............   O   gra   Orangutan
   ...............(.R.).............   O   ape   Gibbon
   ...............(...)V............   O   cat   Rhesus macaque
   ...............(...)V............   O   cat   Crab-eating macaque
   ........C......(...)V............   O   cat   Baboon
   ...............(...)I............   O   cat   Green monkey
   ...............(...)............A   O   sim   Marmoset
   .............M.(...)............A   O   sim   Squirrel monkey
   ....Q.....HAV..(D.P)I.H.........A   X   pri   Bushbaby
   ..T.....HAHS..K(D.S)TP..........A   X   eua   Treeshrew
   ..RF.....AHT..K(D..)T.......V.F.A   X   gli   Squirrel
   ....Q.I.Q.HS..K(S.A)T...R....V..A   X   gli   Lesser Egyptian jerboa
   .........AHS.PQ(..M)T...........A   X   gli   Prairie vole
   .........AHSP.Q(..I)T.S.........A   X   gli   Chinese hamster
   .........AHSP.Q(..I)T.V.........A   X   gli   Golden hamster
   ........QAHS..K(S.I)T...........A   X   gli   Mouse
   .........AH--.K(..V)T...........A   X   gli   Rat
   ....Q....ARA..Q(DQP)T...R....V..A   X   gli   Naked mole-rat
   .........AHS..Q(EQP)T........V..A   X   gli   Guinea pig
   ....Q....AHD..Q(ELP)T........V..A   X   gli   Chinchilla
   ..H.K...WAHN..Q(EQP)N........I..A   X   gli   Brush-tailed rat
   ..R.H.....RSTL.(..S)....R....V..A   O   gli   Rabbit
   ..R.Q.S.H.RG.CK(DQG)..P.........A   X   gli   Pika
   .N.......ASA...(DRP)PS..........A   X   lau   Pig
   .N.......APA...(DG.)PG..........A   X   lau   Alpaca
   .N.......APA...(DG.)PG..........A   X   lau   Bactrian camel
   .N.......SPA--.(DR.)PG..........A   X   lau   Dolphin
   .N.......SPA--.(DR.)PG..........A   X   lau   Killer whale
   .N......SARA--.(D..)PG..........A   X   lau   Tibetan antelope
   .N......SAPA--.(D..)PGF.........A   X   lau   Cow
   .N......SARA--.(D..)PG..........A   X   lau   Sheep
   .N......SARA--.(D..)PG..........A   X   lau   Domestic goat
   --.......APA...(DR.)T...........A   X   lau   Horse
   .S.......APA...(DR.)T...R.......A   X   lau   White rhinoceros
   .NR......APA..Q(DR.)TG..R.......A   X   lau   Cat
   .NR......APA..R(DR.)TG..........A   X   lau   Dog
   .NR......APA..Q(DR.)TD..........A   X   lau   Ferret
   .NR......APA..Q(DG.)TG..........A   X   lau   Panda
   .NR......APA..Q(DR.)TG.......F..A   X   lau   Pacific walrus
   .NR......APA..Q(DR.)TG..........A   X   lau   Weddell seal
   .S..Q...CAPA...(...)TL..........A   O   lau   Black flying-fox
   .S..Q...CAPA...(...)TL..........A   O   lau   Megabat
   .N......PAPA..V(DR.)TG......V...A   X   lau   David's myotis bat
   .N......PAPA..A(DR.)TG......V...A   X   lau   Microbat
   .N.......APA...(DR.)TG......V...A   X   lau   Big brown bat
   ..T.....HAPA..A(.S.)T........F..A   O   lau   Hedgehog
   .NKML...--HDF..(.G.)TMFY.....Y..A   O   lau   Shrew
   .S.......VPA...(DR.)TS..........A   X   lau   Star-nosed mole
   ..R.Q.....DG.L.(.R.)TC..R.......A   O   afr   Elephant
   .KR.Q.....DV.L.(DR.)T...........A   X   afr   Cape elephant shrew
   ..R.Q.N.H.DA.L.(.R.)TC..R.......A   O   afr   Manatee
   ..R.Q...C.GA...(DS.)IR..R.......A   X   afr   Cape golden mole
   ..R...A.H.DA...(DRA)TGPYR....I..A   X   afr   Tenrec
   ....Q.....DA...(DRI)P........F..A   X   afr   Aardvark
   DGR.Q...H.GA...(DRA)ST..........A   X   xen   Armadillo
   ------------.P.(.G.)TIFY.....YF.A   O   mar   Opossum
   QKKMK.S.-..S.P.(.W.).IFF....VY..V   O   mar   Tasmanian devil

NO 18
GN C3
ID CO3_HUMAN
MP 85
DE Complement C3
CL great apes
SQ 55
   SSEKTVLTPATNHMG(NVT)FTIPANREFKSEK   O   hum   Human
   ...............(.F.)CM...E-------   O   hac   Chimp
   ...............(...).....E-------   O   aga   Gorilla
   ...........S...(...).....E-------   O   gra   Orangutan
   ...........S...(S..)IR...E-------   X   cat   Rhesus macaque
   ...........S...(S..)IR...E-------   X   cat   Crab-eating macaque
   ...........S...(S..)IR...E-------   X   cat   Baboon
   ...........S...(S..)IR...E-------   X   cat   Green monkey
   ...........S.I.(...)IR...D-------   O   sim   Squirrel monkey
   AN..IT..R..G.L.(...)IKVE.G-------   O   pri   Bushbaby
   .....M..A.NG.LS(T..)VK...E-------   X   eua   Treeshrew
   .......SG..G.L.(...)IK-----------   O   gli   Squirrel
   .......MG.MG.L.(...)IK....-------   O   gli   Lesser Egyptian jerboa
   T.......G..R.L.(T..)IK....-------   X   gli   Prairie vole
   T.......S..G.LS(SI.)IK....-------   X   gli   Chinese hamster
   T.......S.AGYLS(SI.)VK....-------   X   gli   Golden hamster
   T.......G.SG.LR(S.S)IK...D-------   X   gli   Mouse
   T.......G..G.LN(R.S)IK...D-------   X   gli   Rat
   ........S..G.L.(T..)IK..SD-------   X   gli   Naked mole-rat
   ........S..GYL.(T..)IK-----------   X   gli   Guinea pig
   C.......S.RG.L.(T..)IK...D-------   X   gli   Chinchilla
   ........S..GYL.(T.S)IK...D-------   X   gli   Brush-tailed rat
   F...L...S..GYL.(...).K..NG-------   O   gli   Pika
   ...T.T.NN.N.YLS(T.N)IK...E-------   X   lau   Pig
   ...N...NR.NSYL.(H.N)IK.QEV-------   X   lau   Alpaca
   ...N...NR.NSYL.(H.N)IK.QEV-------   X   lau   Bactrian camel
   ...N.E.NS.NGYL.(T..)IK---E-------   X   lau   Dolphin
   ...N.E.NS.NGYL.(T..)IK...E-------   X   lau   Killer whale
   .N.N.Q.NSNNGYLS(T..)IK...D-------   X   lau   Tibetan antelope
   .N.N.Q.NSNNGYLS(T..)IK...D-------   X   lau   Cow
   .K.N.Q.NSNNGYLS(T..)IK...E-------   X   lau   Domestic goat
   .....I..K.NGYL.(T..)IK...A-------   X   lau   Horse
   ........K.AG.L.(T..)VK...A-------   X   lau   White rhinoceros
   .RGN.Y..KDND..S(T.N)IK...G-------   X   lau   Cat
   FR...D..S.NQY.S(T..)IQM..E-------   X   lau   Dog
   FN...E.NS.NEYLS(A..)I...NE-------   X   lau   Ferret
   AN...D..SNNDY.S(T..)IS..TE-------   X   lau   Panda
   .N...D..S.NDY.S(T..)IR..TE-------   X   lau   Pacific walrus
   .N...D..S.NDY.S(T..)IR...E-------   X   lau   Weddell seal
   ...N.L..K.NGF.D(T..)VK...E-------   X   lau   Black flying-fox
   ...N.L..K.NGF.D(T..)VK...E-------   X   lau   Megabat
   ...D.I.NS.NG.LS(T..)IK...T-------   X   lau   Microbat
   ..KD.I.NS.NGYLS(T..)IK...Q-------   X   lau   Big brown bat
   F.......K.N..LS(AIP)VKV.VG-------   X   lau   Shrew
   T....M..S.NG.LD(T..)I...VS-------   X   lau   Star-nosed mole
   V.......S.NS.V.(T.N)IK...D-------   X   afr   Elephant
   L....E...ENS..S(S..)IK...S-------   X   afr   Cape elephant shrew
   F..D.Q..A.NS.LS(ALP)IK...D-------   X   afr   Manatee
   ...N....A.SG.L.(T..)IK...T-------   X   afr   Cape golden mole
   ...N......NG.L.(TI.)MK...T-------   X   afr   Tenrec
   ...N....D.NG.LS(T..)VK-----------   X   xen   Armadillo
   FL.QLT.NN.N.YLT(T.N).K....-------   X   mar   Opossum
   Y...IT.NN.N.YLS(TI.)VK...K-------   X   mar   Tasmanian devil
   Y.Q.IT.NN.N.YL.(TI.)VKV..K-------   X   mar   Wallaby
   YN..IM.SSDNDYL.(IIK)IKL.GK-------   X   mon   Platypus

NO 19
GN C4BPB
ID C4BPB_HUMAN
MP 71
DE C4b-binding protein beta chain
CL simians
SQ 56
   VGKKTLFCNASKEWD(NTT)TECRLGHCPDPVL   O   hum   Human
   ...............(...).............   O   hac   Chimp
   ...............(...).............   O   aga   Gorilla
   ...............(...)A............   O   gra   Orangutan
   ...............(...)...H.........   O   ape   Gibbon
   .....V.........(...).............   O   cat   Rhesus macaque
   .....V.........(...).............   O   cat   Crab-eating macaque
   .....V.........(...).............   O   cat   Baboon
   ...........E...(...).............   O   cat   Green monkey
   ...............(...)...C.........   O   sim   Marmoset
   ............G..(...)...C.........   O   sim   Squirrel monkey
   ..E...L...T...S(AP.).K...........   X   pri   Bushbaby
   ...G......FD..T(VP.)AT.H.....N...   X   eua   Treeshrew
   .....FL....EG.N(DF.).............   X   gli   Squirrel
   ...E.FV....E...(VSP).............   X   gli   Lesser Egyptian jerboa
   ...QS.VL.I.E...(VSP)P........E...   X   gli   Prairie vole
   ...QS.VL.L.E...(DSP)P..H.....E...   X   gli   Chinese hamster
   ...QF.VL.L.E...(DSP)P........E...   X   gli   Golden hamster
   ...QS.VFDP....N(ASL)P..Q*........   X   gli   Mouse
   ....S.VFDP....N(A.L)P..L.........   X   gli   Rat
   Q...SFV....E..V(ASA).Q...........   X   gli   Naked mole-rat
   E...VF.S...E...(AS.).Q...........   X   gli   Guinea pig
   E.TRVFVSD..E...(ASA).Q...........   X   gli   Chinchilla
   K...FFVSD.IE..N(VSP)A..H.........   X   gli   Brush-tailed rat
   ..E......S.Q..N(APA)P............   X   lau   Pig
   ..E..I..S...G.N(A.S)PQ.QW......F.   X   lau   Alpaca
   ..E..I......G.N(A.S)PQ.QW......F.   X   lau   Bactrian camel
   ..E.........G.N(ASA)P..C.......L.   X   lau   Dolphin
   ..E.........G.N(ASA)PA.........L.   X   lau   Killer whale
   ------.FD...G.N(AL.)P..C.......L.   X   lau   Tibetan antelope
   ------.FD...G.N(AL.)P..C.......L.   X   lau   Cow
   ------.FD...G.N(AL.)P..C---------   X   lau   Sheep
   ------.FD...G.N(AL.)P..C.......L.   X   lau   Domestic goat
   ..E........L..N(GSP)P...F....N...   X   lau   White rhinoceros
   ..E..FI....G..N(.P.)PT.Q.........   X   lau   Cat
   .....F.....L..N(APV)PT.Q.....V...   X   lau   Dog
   ..E........V..N(APV)PT...........   X   lau   Ferret
   ..E.IR.....M..N(APV)PT...........   X   lau   Panda
   ..E..FV....T..N(APV)PT..P........   X   lau   Pacific walrus
   ..E..F.....T..N(APV)PT..P........   X   lau   Weddell seal
   ..E..FY....E..N(TP.)PK.H.........   X   lau   Black flying-fox
   ..E..FY....E..N(TP.)PK.H.........   X   lau   Megabat
   ..ES..........N(AGP)PR...........   X   lau   David's myotis bat
   ..ES..........N(AR.)PR...........   X   lau   Microbat
   ..ES.......E..N(AP.)PR...........   X   lau   Big brown bat
   ..RQ..I..T.QG.S(GPA)PT.QV........   X   lau   Hedgehog
   I...S.S.VT.QG..(AP.)PR.HV........   X   lau   Shrew
   ......S...FQD..(TP.)PT.H.........   X   afr   Elephant
   E.STS.......D..(APP)PS.......E...   X   afr   Cape elephant shrew
   ...............(AP.)PT.L.........   X   afr   Manatee
   ...D...........(AP.)PT.........E.   X   afr   Cape golden mole
   ...R.IH....MV..(AP.)PT.H.........   X   afr   Tenrec
   ......V........(APA)PR.H.........   X   afr   Aardvark
   ..E.AF...TP...N(API)PK.Y.--.L..I.   X   xen   Armadillo
   ....E.LY.T.L...(SPA)PI.H.....I...   X   mar   Opossum
   ...........HK..(RPA)PS.L---------   X   mon   Platypus

NO 20
GN C5
ID CO5_HUMAN
MP 741
DE Complement C5
CL simians
SQ 61
   KAFTECCVVASQLRA(NIS)HKDMQLGRLHMKT   O   hum   Human
   ...............(...).............   O   hac   Chimp
   ...............(.N.).............   O   aga   Gorilla
   ...............(.N.).............   O   gra   Orangutan
   ............H..(.S.)Y..I.........   O   ape   Gibbon
   ...............(.N.)...L.........   O   cat   Rhesus macaque
   ...............(.N.)...L.........   O   cat   Crab-eating macaque
   ...............(.N.)...L.........   O   cat   Baboon
   ...............(.D.)L..LH........   O   cat   Green monkey
   ........A.AEHC.(.C.)F.H......G..I   O   sim   Marmoset
   ........E.NRH.D(.ST)Y.PIS....D..I   O   sim   Squirrel monkey
   ...N...TI.N.V.S(SH.)..NI....I...S   X   pri   Bushbaby
   A..NA..IL.IK...(EL.)..PI......IQA   X   eua   Treeshrew
   R..N...II.K.Y.D(ADP)..NI.....NI..   X   gli   Squirrel
   ...N...IL.HKI.T(EQG)..N........M.   X   gli   Lesser Egyptian jerboa
   R..N...II.TKI.Q(EA.)L.LV....M.I..   X   gli   Prairie vole
   R..N...II.TKI.Q(EGF)I.P.....I.I..   X   gli   Chinese hamster
   R..N...II.TKM.Q(ET.)Y.PV....I.I..   X   gli   Golden hamster
   R..N...TI.NKI.K(ESP)..PV....I.I..   X   gli   Mouse
   R..N...TI.DKI.K(ESH)..G.L...IQI.A   X   gli   Rat
   Q..N...IL.NKI..(ES.)..PI......I..   X   gli   Naked mole-rat
   R..N...TL.NKI..(EDT)L.PIP.....V..   X   gli   Guinea pig
   R..N...IL.TK...(EHT)..PN......I..   X   gli   Chinchilla
   R..N...IL.KKI.D(QDP)..PI......I..   X   gli   Brush-tailed rat
   R..N...SI.R.F.D(KH.)..NI......T..   X   gli   Rabbit
   ...S...II.TEF..(.M.)..NI......TMP   O   gli   Pika
   ...KD..YI.N.V..(EE.)..NI......I..   X   lau   Pig
   ...K...AI.Q.H.F(.E.)Y.NI.....QI.A   O   lau   Alpaca
   ...KD..AI.Q.H.F(.E.)Y.N......QI.A   O   lau   Bactrian camel
   .L.KK..AI...I..(AE.)G.II......I..   X   lau   Dolphin
   .V.KN..AI...I..(AE.)G.II......I..   X   lau   Killer whale
   ...K...AI..EF..(DET)Y.NK......V.S   X   lau   Tibetan antelope
   ...KS..AI...F..(DEH)..N.......I.S   X   lau   Cow
   ...K...AI..EF..(.KT)Y.NI......V.S   O   lau   Sheep
   ...K...AI..EF..(.KT)Y.NI......V.S   O   lau   Domestic goat
   QV.KD..AI.E....(.E.)..HI......I..   O   lau   Horse
   Q..KI..T..K.I..(.ET)..GV.....QI..   O   lau   White rhinoceros
   RI.K...LI.KKH.D(EDP)L.NI......I..   X   lau   Cat
   .I.KD..II...I.D(KE.)...I....M.I..   X   lau   Ferret
   RI.K...II.C...D(KE.)..NL......I..   X   lau   Panda
   .I.K...NI.....S(EM.)..NIH.....I..   X   lau   Pacific walrus
   .I.KD..NI...H.S(KEY)R..I......I..   X   lau   Weddell seal
   T..K...AI.NKF.S(EE.)..E.R.....I..   X   lau   Black flying-fox
   T..K...AI.NKF.S(EE.)..EVR.....I..   X   lau   Megabat
   Q..MN..TI.HLF..(EE.)..H.L.....IRP   X   lau   David's myotis bat
   Q..MN..TI.HLF..(EE.)..LI......IRP   X   lau   Microbat
   Q..MN..TI.HLF..(EE.)..L.......IRP   X   lau   Big brown bat
   ...K....A.NKF.D(EM.)...IR.....I..   X   lau   Hedgehog
   M..NA..KI.HHHH.(TMP).RVL....H.I.A   X   lau   Shrew
   Q..MS..AI..EF..(.F.)..FL.....GI..   O   lau   Star-nosed mole
   A..N...I..KKF.D(ENP)..NI.....QL.S   X   afr   Elephant
   R..N...II.....N(EH.)..PL......I..   X   afr   Cape elephant shrew
   A..N...SI.KKF.D(DN.)...I.....QI..   X   afr   Manatee
   A..NV..TI.NKF.D(END)N.IL.....EI.S   X   afr   Cape golden mole
   Q..NS..TI.TNF.D(GNP)K.N......Q..N   X   afr   Tenrec
   A..K...NI.KKF.D(ENP)I.PI......I.S   X   afr   Aardvark
   TI.K...NI.K...D(ED.)V.N......QI..   X   xen   Armadillo
   E..KS..EL.F.I.S(KSN)..PVI...NYI..   X   mar   Opossum
   E..RT..LF..KM.I(ESE)R.HLI...IYI.A   X   mar   Tasmanian devil
   E..KN..LL.T.I.D(KFG)I.PIL...IY---   X   mar   Wallaby
   RV.K...EL.TEV..(.CT)..H.L...IGFE.   O   mon   Platypus

NO 21
GN CD163
ID C163A_HUMAN
MP 1027
DE Scavenger receptor cysteine-rich type 1 protein M130
CL primates
SQ 61
   RWGHSECGHKEDAAV(NCT)DISVQKTPQKATT   O   hum   Human
   ...............(...)...........-.   O   hac   Chimp
   ....N..........(...)H............   O   aga   Gorilla
   .....D.........(...)..P.H........   O   gra   Orangutan
   ...............(...)....H........   O   ape   Gibbon
   ....N..........(...)...AH......P.   O   cat   Rhesus macaque
   ....N..........(...)...AH......P.   O   cat   Crab-eating macaque
   ...............(...)S..AHE.S....R   O   cat   Baboon
   ...............(...)E..TH........   O   cat   Green monkey
   ...............(...)N..EH.NA.....   O   sim   Marmoset
   ...............(...)N..EH.N......   O   sim   Squirrel monkey
   ...............(..S)E...SGKSSE.KG   O   pri   Bushbaby
   P....D.........(R.S)E.PEI.E.LNT-A   X   eua   Treeshrew
   P...TD.........(Q.P)GTLSANA-----.   X   gli   Squirrel
   A.H..D.........(Q.P)GT.SRHN-----.   X   gli   Lesser Egyptian jerboa
   P.S..D.......S.(M.P)GTPSERG-----.   X   gli   Prairie vole
   P.S..D.......SI(Q.P)GAPSEQ-------   X   gli   Chinese hamster
   P.S..D........I(R.P)GAPSEH-------   X   gli   Golden hamster
   P.S..D.......SI(Q.P)KT.SHHG-----.   X   gli   Mouse
   ...N.D.........(R.P)GT.SSTA.-----   X   gli   Naked mole-rat
   Q..Y.D.........(Q.P)GT.SM-------P   X   gli   Guinea pig
   Q....D.........(Q.P)GMEY*QI.-----   X   gli   Chinchilla
   E....D.........(Q.P)GT.SPPP.-----   X   gli   Brush-tailed rat
   P....D.W.......(K.P)GY.DT.G.LRN-.   X   gli   Rabbit
   P....D.........(K.P)R..DTQGSLRN-.   X   gli   Pika
   P..Q.D.K.E...G.(R.S)-------------   X   lau   Pig
   S....D.......S.(R.S)EVAEN.G.L..-.   X   lau   Alpaca
   S....D.......S.(R.S)EVAEN.G.L..-.   X   lau   Bactrian camel
   S....D.......S.(K.S)E.AKNEGSLN.-.   X   lau   Dolphin
   S....D.......S.(K.S)E.AKNEGSLN.-.   X   lau   Killer whale
   Y....D.......S.(K.S)E.AES.GSL..-A   X   lau   Tibetan antelope
   S....D.......S.(K.S)E.AES.GSV..-A   X   lau   Cow
   Y....D.......S.(K.S)E.AES.GSL..-A   X   lau   Sheep
   Y....D.......S.(K.S)E.AES.GSL..-A   X   lau   Domestic goat
   S....D.........(R.S)ELVESSNA----.   X   lau   Horse
   S....D.........(R.S)---.PEESSN.-.   X   lau   White rhinoceros
   A....D.........(R.S)E.A.AQRSST.-A   X   lau   Cat
   P....D.........(R.S)E.AMAQRSSNP-R   X   lau   Dog
   P....D.........(R.S)E.AMAQRSSN.-G   X   lau   Ferret
   P....D.........(R.S)ELAMAQRSSN.-G   X   lau   Panda
   P....D.........(R.S)E.AMAQRSSN.-G   X   lau   Pacific walrus
   P....D.........(R.S)E.AMTQRSSN.-G   X   lau   Weddell seal
   S....D.........(I.S)E--STNGLSD.-.   X   lau   Black flying-fox
   S....D.........(I.S)E--STNGLSD.-.   X   lau   Megabat
   S....D.........(I.S)G--TA.E.SS.-K   X   lau   David's myotis bat
   S....D.........(I.S)G--NA.ESSH.-K   X   lau   Microbat
   S....D.........(I.S)G--NA.ESSS.-K   X   lau   Big brown bat
   T....D.........(R.L)EV.RTLESSSS-.   X   lau   Hedgehog
   T....D.........(R.L)ETP.--K.PS.-.   X   lau   Shrew
   I....D.........(R.S)E..TPIK.SD.-.   X   lau   Star-nosed mole
   P....D.......G.(R.S)G.A.T.QSPNS-.   X   afr   Elephant
   S....D.........(E.S)GTAI---.PNS-.   X   afr   Cape elephant shrew
   P....D.......G.(R..)G.VET.ESPNS-.   X   afr   Manatee
   P....D.......G.(E.L)G.T.N.ASPDS-.   X   afr   Cape golden mole
   P....D.......G.(E.L)G.A.TR.SPVS-.   X   afr   Tenrec
   S.R.ND.......G.(E.L)GPA.TRESRNS-.   X   afr   Aardvark
   P....N.......G.(R.S)GAAAT.KSSS.-.   X   xen   Armadillo
   H.KQTD.E.....G.(R.S)GVLMPTE.HE.S-   X   mar   Opossum
   P..QAD.......G.(R.S)-------------   X   mar   Tasmanian devil
   P..QND.K.....G.(K.S)-------------   X   mar   Wallaby
   PM.Q.D.........(K.S)-------------   X   mon   Platypus

NO 22
GN CD48
ID CD48_HUMAN
MP 104
DE CD48 antigen
CL catarrhines
SQ 53
   PQSGALYISKVQKED(NST)YIMRVLKKTGNEQ   O   hum   Human
   ...............(..I).............   X   hac   Chimp
   ...............(...).............   O   aga   Gorilla
   ...............(...).............   O   gra   Orangutan
   .........N.....(..I).............   X   ape   Gibbon
   ...............(...).V......D.Y..   O   cat   Rhesus macaque
   ...............(...).V......D.Y..   O   cat   Crab-eating macaque
   ...............(...)........D.Y..   O   cat   Baboon
   ...............(...)........N....   O   cat   Green monkey
   A...V..M.N.....(ANI)..F....E.MF..   X   sim   Marmoset
   S..........E...(ANI).......E.LF..   X   sim   Squirrel monkey
   ......N..NT..D.(S..)FRIKM.NAS.VA.   X   pri   Bushbaby
   R.....H.TN..R..(S..).F.....TD.KL.   X   eua   Treeshrew
   ...P..H.HQ.....(S..)..L..S.DD.K..   X   gli   Squirrel
   S.TP..H.YN.R...(SGP).YL...RR.--.E   X   gli   Lesser Egyptian jerboa
   NIT...Q.YNIR...(RGD).Y.....T---.E   X   gli   Prairie vole
   HTN...R.YN.R...(KGH).Y....ET---.K   X   gli   Chinese hamster
   LT....H.YN.S...(KGD).YV...EM---..   X   gli   Golden hamster
   ENN...H..N.R...(KG.).Y....ET---.N   X   gli   Mouse
   KTN...R.YN.S...(RGD).Y..M.ET---.D   X   gli   Rat
   N.....H.YNI..S.(S..)..L..SEEDED.R   X   gli   Naked mole-rat
   ...A..H.YN.E.S.(S..).LL..SNES.S.Y   X   gli   Guinea pig
   .R.T..H.YN.....(S..).LL..SNEG.S.Y   X   gli   Chinchilla
   ...H..H.YN.E.Q.(S..).LL.LSNEG.S.A   X   gli   Brush-tailed rat
   ......N..D.R...(S.D).LV...MAS.T.R   X   gli   Rabbit
   ...S..N..D.....(SN.).LV.I..EG...H   X   gli   Pika
   L.....H.Q..K...(E..)..LK.NGD.R..K   X   lau   Pig
   I.T...N.Y....D.(S..).LLKI.YNA...N   X   lau   Alpaca
   I.T...N.Y....D.(S..).LLKM.YNA...N   X   lau   Bactrian camel
   C...T.H.YN.....(S..).LL....DS.Y.D   X   lau   Dolphin
   L...T.H.HN.....(S..).LLK...DP.Y.D   X   lau   Killer whale
   ......F.RNI....(S..).LL....DN.D.K   X   lau   Cow
   --..T.H.......N(S..).LL....DS.H.E   X   lau   Domestic goat
   LTN...D.HN.....(S..).LL..V.V....E   X   lau   Horse
   LST...N.Y......(S..).LL..V.AA...E   X   lau   White rhinoceros
   --DHT.F.YN.R...(S..)..LK...ES.K.I   X   lau   Cat
   --.Y..C.Y......(S..)..L....DS.K.E   X   lau   Dog
   N.N--.C.HN...N.(S..)..L...TDS.T.K   X   lau   Ferret
   --NQ..S.FN.....(S..)..L....ES.K.E   X   lau   Panda
   --.H..C.YN.....(S..)..L.M.DES.T.K   X   lau   Pacific walrus
   --NH....YN.....(S..)..L...EEY.R.K   X   lau   Weddell seal
   H.N.T.H.Y..R...(S..).LLKI..EA.T.E   X   lau   Black flying-fox
   --N.T.H.CN.....(S..)..LK..L.S.F.E   X   lau   David's myotis bat
   H.D...H.YN..E..(S..)..LK..LDNRI.E   X   lau   Microbat
   L.D.T.H.YN..E..(S.I).TLK.VL.S.F.E   X   lau   Big brown bat
   --N.V.T.YN...N.(SGI).TLKRWNYE.K.E   X   lau   Star-nosed mole
   -DH.T.H.YT..ES.(SG.)..LQ..DI..V.R   X   afr   Elephant
   -SN.I.T.CN.TAK.(SN.).YLK.---*.KME   X   afr   Cape elephant shrew
   -NN.T.N.YR...N.(STE).LLQA.ETS.V..   X   afr   Manatee
   -NN.T.H.YR..EQ.(SGN).SLQ.VNTD.IV.   X   afr   Cape golden mole
   -DH.T.F.N....N.(S..).TLQ..WIG.E..   X   afr   Tenrec
   -DN.T.H.YN.REN.(S..).LLQ.FTTE.H.H   X   afr   Aardvark
   SS..S.S.FD.K...(S.I).LLK..HEA...D   X   xen   Armadillo

NO 23
GN CD80
ID CD80_HUMAN
MP 226
DE T-lymphocyte activation antigen CD80
CL simians
SQ 59
   NHSFMCLIKYGHLRV(NQT)FNWNTTKQEHFPD   O   hum   Human
   ...............(...).............   O   hac   Chimp
   ...............(...).............   O   aga   Gorilla
   ...............(...).....P.......   O   gra   Orangutan
   ...............(...).....P.......   O   ape   Gibbon
   ...............(...).....P.......   O   cat   Rhesus macaque
   ...............(...).....P.......   O   cat   Crab-eating macaque
   ....V..........(...).....P.......   O   cat   Baboon
   ...............(...).....P.......   O   cat   Green monkey
   .............K.(..I)...R.S.P.....   X   sim   Marmoset
   .............K.(...)...R.P.......   O   sim   Squirrel monkey
   ....T......D.T.(S..)...Q.S...PL.-   X   pri   Bushbaby
   D...V..V...DSTI(S..).H.QKSRN.PYIL   X   eua   Treeshrew
   ....V.RV...D.S.(S.I)...EKPEDP--..   X   gli   Squirrel
   .Q..V.VVQ..EHT.(SSV)YD.IKPEEP--..   X   gli   Lesser Egyptian jerboa
   ...IV.H.A..DSQ.(SKN)YT.EKPEAP--..   X   gli   Prairie vole
   ...IV.H.A..DSQ.(SKN).T.EKPEAP--..   X   gli   Chinese hamster
   ...IV.QVS..DSQ.(SKN).T.EKPEAP--..   X   gli   Golden hamster
   ..TIK......DAH.(SED).T.EKPEDPPDSK   X   gli   Mouse
   D.FID.F.E..DAH.(S.N).T.EKPEDP--..   X   gli   Rat
   ....K......DTT.(SE.).S.QKPQ.DDN.N   X   gli   Naked mole-rat
   S.V.K..V...D*A.(SE.).I.QRPQPNDT.G   X   gli   Guinea pig
   ..I.K..V...DKT.(SE.).I.QKPQPNDT..   X   gli   Chinchilla
   ....Q..V...NTE.(SE.).T.QKPQPNET.G   X   gli   Brush-tailed rat
   ...IV......E.S.(S.I).P.SKPQEP--.V   X   gli   Rabbit
   ...I.......DS..(S..).T.HKPPEP--.I   X   gli   Pika
   .......V...G.T.(S..)...QKSQTS--SA   X   lau   Pig
   .......V...D.T.(S..)...QKSPAP--SA   X   lau   Alpaca
   .......V...D.T.(S..)...QKSPAP--SA   X   lau   Bactrian camel
   ....V..V...G.T.(S..)...QKSPAP--SA   X   lau   Dolphin
   ....V..V...G.T.(S..)...QKSPAP--SA   X   lau   Killer whale
   ....L..V...D.T.(S..).Y.QESPTP--SA   X   lau   Tibetan antelope
   ....L..V...D.T.(S..).Y.QESPTP--SA   X   lau   Cow
   ....L..V...D.T.(S..).Y.QESPTP--SA   X   lau   Sheep
   ....L..V...D.T.(S..).Y.QESPTP--SA   X   lau   Domestic goat
   ....V..V...D.T.(S..).K.QKPLDS--.T   X   lau   Horse
   ....V..V...D.T.(S..).K.Q.PPDS-..N   X   lau   White rhinoceros
   ....L..V...N.L.(S.I)...QKSP.P--SN   X   lau   Cat
   ....V..V...D.T.(S.I)...QKFPHP--.N   X   lau   Dog
   ....V..V...D.T.(S.I).S.QKFPPH--T.   X   lau   Ferret
   ....V..V...D.T.(S.I).D.QKFPPP--L.   X   lau   Panda
   ....V..V...D.A.(S.I)...QKFPPP--..   X   lau   Pacific walrus
   ....V..V...D.A.(S.I)...QKFPTP--..   X   lau   Weddell seal
   ....I..V...N.T.(S..)...QK.PPPS.N-   X   lau   Black flying-fox
   ....I..V...N.T.(S..)...QK.PPPS.N-   X   lau   Megabat
   ..T.I..V...N.T.(S.I)...QKSPNS--.F   X   lau   David's myotis bat
   ..T.I..V...N.T.(S.I)...QKYPTS--.F   X   lau   Microbat
   S.N.T.FV...NST.(SHN)YH.QKITDS----   X   lau   Hedgehog
   .......V...N.Q.(S.N).T.QKIPDP.PS-   X   lau   Shrew
   ....V..V...N.T.(SKN)...QKNSDRRSS-   X   lau   Star-nosed mole
   ..T.V......DQE.(S..).P.IIPPSP--.V   X   afr   Elephant
   ..TLV...N..DKQ.(SKN)ISLVMNPT.--.I   X   afr   Cape elephant shrew
   ..T.V......DQQ.(S..).P.IISPSP--LV   X   afr   Manatee
   ..T.I.V....DKE.(S.N).S.IISPL---.E   X   afr   Cape golden mole
   .RT.I.V...RDKE.(S..).R.IIPPP---..   X   afr   Tenrec
   ..T.I......DKQ.(S..).S.IIPPLP--T.   X   afr   Aardvark
   ..T.V......DKE.(S.I).H.RISQNHSLSQ   X   xen   Armadillo
   .SL.I.S.D..DS..(.V.)-----------..   O   mar   Opossum
   .F.IT......DFH.(.R.)-------------   O   mar   Wallaby

NO 24
GN CD97
ID CD97_HUMAN
MP 453
DE CD97 antigen
CL simians
SQ 57
   QLRRLSAVNSIFLSH(NNT)KELNSPILFAFSH   O   hum   Human
   ...............(...).............   O   hac   Chimp
   .F........V....(...).............   O   gra   Orangutan
   R.........V....(...)-------------   O   ape   Gibbon
   R.........V....(...)....F........   O   cat   Rhesus macaque
   R.........V....(...)....F........   O   cat   Crab-eating macaque
   R.........V....(...)....F........   O   cat   Baboon
   R.........V....(...)....F........   O   cat   Green monkey
   ..TC......V....(...)...S.........   O   sim   Marmoset
   ..TC......V....(...)...S.........   O   sim   Squirrel monkey
   .VQLY.....V...N(T..)EK.A...T.....   X   pri   Bushbaby
   R..L..P...V...N(K..).R.D.LVT.....   X   eua   Treeshrew
   RIQI..P...V..TN(.D.)EK.A..VT.....   O   gli   Squirrel
   .VMV..N...V...N(TE.)QA.T..VN.T..Y   X   gli   Lesser Egyptian jerboa
   S.TL..N.S....TN(TD.)GK.T.NVT.K..Y   X   gli   Prairie vole
   S.TL..NI.....TN(TD.)RK.A.NVT.N...   X   gli   Chinese hamster
   S.TL..N......TN(TD.)RK.A.NVT.N..Y   X   gli   Golden hamster
   S.KL..NI..V..TN(T..)EK.A.NVT.K.DF   X   gli   Mouse
   T.TF..NI.A...TN(TD.)EK.A.DVT.K.NL   X   gli   Rat
   .GTL......V...N(TD.)DK.A..VT.....   X   gli   Naked mole-rat
   RATL...I..V.V.N(AD.)DK.A..VT.....   X   gli   Guinea pig
   RATL......V...N(T..)DQ.A..VT.....   X   gli   Chinchilla
   RATL......V...N(T..)DK.A..VT.....   X   gli   Brush-tailed rat
   RPQL....S.V.V.N(RK.)ER.S..VT.....   X   gli   Pika
   ...L......V...N(T..)DK.D..VT.....   X   lau   Pig
   KVSLFT..S.V...N(R..)DK.D.NVT.....   X   lau   Alpaca
   KVSLFT..S.V...N(R..)DK.DFNVT.....   X   lau   Bactrian camel
   ...L.....LV...N(T..)DK...KVT.....   X   lau   Dolphin
   ...L.....LV...N(T..)DK...KVT.....   X   lau   Killer whale
   KVTL....S.V...N(T..)EK.D.NVS...TL   X   lau   Tibetan antelope
   KVTL....S.V...N(T..)EK.D.NVS...AL   X   lau   Cow
   KVTL....S.V...N(P..)EK.D.NVS...TL   X   lau   Sheep
   KVTL....S.V...N(P..)EK.D.NVS...TL   X   lau   Domestic goat
   ...L......A...N(T..)TA.DT.VT.....   X   lau   White rhinoceros
   .YKL.....LV...N(T..)EH.D..VT.....   X   lau   Cat
   ..QL.....VV...N(T..)EK.D..VT.....   X   lau   Dog
   .AEIV....LV...N(T..)EK.D..VT.....   X   lau   Ferret
   .PLL..P..LV...N(T..)EK.D..VI....Y   X   lau   Panda
   KP.L.....LV...N(TK.)EK....VT.....   X   lau   Pacific walrus
   EP.L.....LV...N(T..)EK.D..VT.....   X   lau   Weddell seal
   .F.F...I..A...N(TK.)EK.D..VT.....   X   lau   Black flying-fox
   .F.F...I..A...N(TK.)EK.D..VT.....   X   lau   Megabat
   .AKI..T..TV...N(K..)EK.DP.VT.S..Y   X   lau   David's myotis bat
   .SKL..T..TV...N(K..)EK.DP.VT.S..Y   X   lau   Microbat
   EIKL......V...N(RD.)R..AT.V....A.   X   lau   Hedgehog
   R.QL......V...N(PH.)ES.DY.VY.....   X   lau   Shrew
   YP.LI..I..V...N(EK.)EK.DF.VT.....   X   lau   Star-nosed mole
   ..KV......V...N(T..)EK.D..VT.....   X   afr   Elephant
   R.TV...I..V...N(K..)EK.E.NVT.S.AY   X   afr   Cape elephant shrew
   ..KV.....TV...N(R..).N.SL.VT.....   X   afr   Manatee
   .FKI...I..A...N(T..)EQ.EP.VT.....   X   afr   Cape golden mole
   RVEV........V.N(...)ET.-P.VT.T..Y   O   afr   Tenrec
   ..SV......A...N(T..)EK.D...T.....   X   afr   Aardvark
   IPIL....STA...N(RD.)QN.S..VT.....   X   mar   Opossum
   IPVL....STA...N(K..)AY..P.VT...T.   X   mar   Tasmanian devil
   VPVL....STA...N(ED.)GN.S..VT.T...   X   mar   Wallaby
   .PVL..T.S...V.N(RD.)QM.S...TL...K   X   mon   Platypus

NO 25
GN CDH5
ID CADH5_HUMAN
MP 112
DE Cadherin-5
CL simians
SQ 56
   AETGDVFAIERLDRE(NIS)EYHLTAVIVDKDT   O   hum   Human
   ...............(...).............   O   hac   Chimp
   ...............(...).............   O   aga   Gorilla
   ...............(...).............   O   gra   Orangutan
   ...............(...).............   O   cat   Rhesus macaque
   ...............(...).............   O   cat   Crab-eating macaque
   ...............(...).............   O   cat   Baboon
   ...............(...).............   O   cat   Green monkey
   ...............(...)....I...A....   O   sim   Marmoset
   ...............(...)........M....   O   sim   Squirrel monkey
   PQ....Y.F......(K..)..Q...L......   X   pri   Bushbaby
   .D....Y.F......(K..)....V.L.....S   X   eua   Treeshrew
   ......Y.F......(KVT)..Q...L....E.   X   gli   Squirrel
   ......Y.F......(K..)......L......   X   gli   Lesser Egyptian jerboa
   .D......F......(KKV)..F...L....E.   X   gli   Prairie vole
   SDS...Y.F......(KVP)..Y...L....ES   X   gli   Chinese hamster
   SD....Y.F......(KVP)..L...L....E.   X   gli   Golden hamster
   .N..N.L.Y......(KV.)..F...L....N.   X   gli   Mouse
   .D....L.Y......(KV.)..F...L....K.   X   gli   Rat
   .D....Y.FA.....(KV.)..S.V.L......   X   gli   Naked mole-rat
   PD......FA.....(KV.)S.P.V.L......   X   gli   Guinea pig
   PD....Y.FA.....(KV.)S.P.V.L.....S   X   gli   Chinchilla
   P.....Y.FA.....(KV.)S.P.V.L......   X   gli   Brush-tailed rat
   .D....Y.F......(K..)....V.L......   X   gli   Rabbit
   S.S.T.S.F......(KV.)....V.L......   X   gli   Pika
   ENS...Y.F......(K..)....I.LV.....   X   lau   Alpaca
   ENS...Y.F......(K..)....I.LV.....   X   lau   Bactrian camel
   ES....Y.F......(K..)....I.LV...N.   X   lau   Dolphin
   ES....Y.F......(K..)....I.LV...N.   X   lau   Killer whale
   KN....Y.L......(K..)......LV.....   X   lau   Tibetan antelope
   KN....Y.L......(K..)......LV....S   X   lau   Cow
   KN....Y.L......(K..)......LV.....   X   lau   Sheep
   KN....Y.L......(K..)......LV.....   X   lau   Domestic goat
   ......Y.F......(K..)..Q.V.LV.....   X   lau   Horse
   ........F......(K..)..N...LV.....   X   lau   White rhinoceros
   EDS...Y.F......(K.P)......LV.....   X   lau   Cat
   ED....Y.F......(K..)......LV.....   X   lau   Dog
   ED....Y.F......(K.P)......LV...N.   X   lau   Ferret
   ED....Y.F......(K.P)......LV...N.   X   lau   Panda
   ED....Y.F......(K.P)......LV...N.   X   lau   Pacific walrus
   ED....Y.F......(K.P)......LV...N.   X   lau   Weddell seal
   ED....Y.F......(K..)....V.L......   X   lau   Black flying-fox
   ED....Y.F......(K..)....V.L......   X   lau   Megabat
   EV......F......(KT.)Q.....L....N.   X   lau   David's myotis bat
   .D....Y.F......(K..)......LV.....   X   lau   Hedgehog
   .N....Y.F......(KV.)......L......   X   lau   Shrew
   K...N...H......(K.P)......LV.N.M.   X   lau   Star-nosed mole
   ED....Y.W......(KK.)..R...F......   X   afr   Elephant
   .DS.M.Y.F......(K.A)Q.....F......   X   afr   Cape elephant shrew
   EK....Y.F......(K..)....V.L....N.   X   afr   Manatee
   ED....Y.Y......(KT.)......L......   X   afr   Cape golden mole
   P.....Y.F......(K..)....V.L......   X   afr   Aardvark
   .D....Y.F......(K..)....I.L....N.   X   xen   Armadillo
   EH....Y.F......(KK.)..Y...Y......   X   mar   Opossum
   .QN...Y.F......(KK.)..Y...Y......   X   mar   Tasmanian devil
   ED...IY.Y.K....(K.A)......L.I..Y.   X   mon   Platypus

NO 26
GN CEACAM1
ID CEAM1_HUMAN
MP 224
DE Carcinoembryonic antigen-related cell adhesion molecule 1
CL euarchonts
SQ 52
   DTGPYECEIQNPVSA(NRS)DPVTLNVTYGPDT   O   hum   Human
   ...............(...).............   O   hac   Chimp
   .A...Q...RDRYGG(I..)Y......L.....   X   aga   Gorilla
   .A.............(...).............   O   gra   Orangutan
   ...............(...).............   O   ape   Gibbon
   ...............(...).............   O   cat   Rhesus macaque
   ...............(...).............   O   cat   Crab-eating macaque
   ...............(...).T...........   O   cat   Baboon
   ...............(...)............A   O   cat   Green monkey
   ..............V(...).............   O   sim   Marmoset
   ...............(...)......I......   O   sim   Squirrel monkey
   .S.HFV....T.E.T(.H.)..F....L....A   O   pri   Bushbaby
   ........T.....T(...)...F...S....P   O   eua   Treeshrew
   ...............(S..)..L...IS....A   X   gli   Squirrel
   .....Q..TW.....(...).S.S...L.....   O   gli   Lesser Egyptian jerboa
   ........TR.L..D(S..)..F...I.....V   X   gli   Chinese hamster
   FR.H....AK..L..(FH.)..F..D.F.....   X   gli   Mouse
   FR.R....AK..LG.(FH.)..F..D.F.....   X   gli   Rat
   G.......TR.L..G(I..)......IS....A   X   gli   Naked mole-rat
   ........TR...AF(...)...S..IS....A   O   gli   Guinea pig
   ...S....MW.EANS(...)..L....S....A   O   gli   Chinchilla
   ........T..I..G(S..)..F..DIY....D   X   gli   Brush-tailed rat
   .......GVS..GNT(I..)..L.M..S....A   X   gli   Rabbit
   ...I.....R.STIV(IFR)NAL...IF.....   X   gli   Pika
   ........T......(T..)..F....IVA---   X   lau   Pig
   .....M..AR....V(S..)..F....IVA---   X   lau   Dolphin
   .....V..AR....V(S..)..F....LVA---   X   lau   Tibetan antelope
   .....VS.AG..ENI(THG)..F...GL.....   X   lau   Cow
   .....V..AR..A.V(S..)..F..DAL.....   X   lau   Sheep
   .....V..AR....V(S..)..F..D.L.....   X   lau   Domestic goat
   ........TR.....(R..)..FY...L.----   X   lau   Horse
   ........T......(H..).SFY...L....A   X   lau   White rhinoceros
   ........TW.....(GH.)..F....L....A   X   lau   Cat
   ........TR.....(D..)..F....L.....   X   lau   Dog
   .....V.ATW....D(GC.)..F....L....A   X   lau   Ferret
   ....*...T......(SC.)..F....L....A   X   lau   Panda
   ........TR.....(GH.)..F....L....A   X   lau   Pacific walrus
   .......ITW..G..(SC.)..FH...L...E.   X   lau   Megabat
   ........T..----(G..)..F....V.....   X   lau   David's myotis bat
   .M......TH.....(G..)..F....VC....   X   lau   Microbat
   ........V..L...(H..)..L....L....A   X   lau   Hedgehog
   N..A.V..NW....S(KT.).QFN.D.L....P   X   lau   Shrew
   .....V..TR.....(R..)..F....L.....   X   lau   Star-nosed mole
   .......RTW.Q.N.(R..)..FI...L.....   X   afr   Elephant
   ...Q.Q.RAW....V(AD.)..LH.D.L.....   X   afr   Cape elephant shrew
   ........TR...N.(R..)..FI...L.....   X   afr   Manatee
   ES.L...KTW.....(R..)G.F....L.....   X   afr   Cape golden mole
   ES.F...RTR....S(.H.)..FF...S.....   O   afr   Tenrec
   .K.....ATW.....(...)..F...IS.....   O   afr   Aardvark
   .......WTR....V(R..)..F....F.....   X   xen   Armadillo
   HE.S.V...K..FYS(...)..F....Y....N   O   mar   Opossum
   .K.......W..I.R(...)..F..DFA.....   O   mar   Wallaby

NO 27
GN CEACAM1
ID CEAM1_HUMAN
MP 405
DE Carcinoembryonic antigen-related cell adhesion molecule 1
CL simians
SQ 53
   DAGTYWCEVFNPISK(NQS)DPIMLNVNYNALP   O   hum   Human
   ...............(...).............   O   hac   Chimp
   ...............(...).............   O   aga   Gorilla
   ...K.......S...(...).....T.K..YPS   O   gra   Orangutan
   ...K...........(...).....T....DPS   O   ape   Gibbon
   ...N.S.....L...(.R.)...V.I....NPA   O   cat   Rhesus macaque
   ...N.S.....L...(.R.)...V.I....NPA   O   cat   Crab-eating macaque
   ...K.S.....L...(.R.)...V.I...-ND.   O   cat   Baboon
   ...K.S.....L...(.R.)...V.I....DPA   O   cat   Green monkey
   ...Q.Y...S....V(...)...R.T.K.----   O   sim   Marmoset
   ...Q.Y...S....V(...).....I.K.----   O   sim   Squirrel monkey
   .E.E.Q.A.S.SV.F(.P.)..VK.Y.RS--PK   X   eua   Treeshrew
   .E.E.H.Q.S.QVFS(EK.)N..T.T.IA--PG   X   gli   Squirrel
   ...L.Q...S..V.S(RK.)GLFR.V.LF----   X   gli   Lesser Egyptian jerboa
   .T.K.Q...S..V.S(KR.)...Q.DIIA--PT   X   gli   Prairie vole
   .S.E.Q...S..V.T(KR.)N..Q.DIIA--PT   X   gli   Chinese hamster
   ...A.Q...S..LNT(R..)..VK.A.L.----   X   gli   Mouse
   ...A.Q...S..LNT(RL.)..VK.A.L.----   X   gli   Rat
   ...E.Q...S..V.S(GR.)AT.K.K.E.----   X   gli   Naked mole-rat
   ...Q.Q...S..V.S(.R.).ALW.D.FH--PE   O   gli   Guinea pig
   ...E.Q...S..V.S(RR.)..LS.Q...--PT   X   gli   Chinchilla
   ...E.R...S.AV.S(SR.)H.LR.E.I.----   X   gli   Brush-tailed rat
   .E.E.Q..A.HLNCS(SK.)..LWMT.KD--PE   X   gli   Rabbit
   .E.E.Q....YLNCS(SK.).SVSIT.KG----   X   gli   Pika
   ...N.Q..AS.LGNS(.K.)..LR.D.KF---S   O   lau   Pig
   ...D.Q...S.QGNS(SR.)..LG.D.KF----   X   lau   Dolphin
   ...D.Q...S.QGNS(SR.)..LR.D.KF---S   X   lau   Killer whale
   .T.D.Q..A..RGNS(.R.)..LR.H.K.---L   O   lau   Tibetan antelope
   ...G.Q...S.RGNS(SR.)G.LR.R.T.---L   X   lau   Cow
   .T.D.Q..A.DRGNS(.R.)N.LR.H.K.---S   O   lau   Sheep
   ...D.Q..A..RGNS(SR.)..LR.R.T.---L   X   lau   Domestic goat
   ..RD.Q...S.LV.S(RKR)NILR-..K.---.   X   lau   Horse
   ...D.Q...S..V.S(SR.).LLR.D.K.---.   X   lau   White rhinoceros
   ...D.Q...S..V.S(SK.).LVK...E.---F   X   lau   Cat
   ...K.Q...S..V.S(.K.)..VR.D.ED---S   O   lau   Dog
   ...N.R...S..GDS(SK.)..VR.V.KG---L   X   lau   Ferret
   ..RN.Q...S..GGS(SK.)E.VRIA.EG---L   X   lau   Panda
   ....*QY..S..G.S(SK.)..VR.A.ED---R   X   lau   Pacific walrus
   ...N.Q...S.RSNS(SK.)A..R.D.SC--FT   X   lau   Weddell seal
   .D.D.Q.....LA.S(DI.)E.LR.H.Q.---.   X   lau   David's myotis bat
   .T.G.Q..I.....S(SK.)..LE.D.Q.---.   X   lau   Microbat
   ...D.Q......V.S(GK.)..FR.D.LD---.   X   lau   Big brown bat
   .T.D.H.M.T..V.F(.K.).TLK.V.KI----   O   lau   Hedgehog
   ...D.H..IS....S(LK.)P.VK.E.TL----   X   lau   Shrew
   ...E.Q..AS..A.S(RR.)..FT.K.LC----   X   lau   Star-nosed mole
   .T.E.Q...Y..V.S(.K.)..LN.T.T.---.   O   afr   Elephant
   .T.N.Q.....NA.S(RR.)...T.T.K.---.   X   afr   Cape elephant shrew
   ...V.L.K.Y.LV.S(ST.)..LN.T.K.---.   X   afr   Manatee
   .N.D.Q......V.Y(RK.)E.LI.T.TV----   X   afr   Cape golden mole
   ...K.Q...S..GNA(QK.).SLT.T.EN----   X   afr   Tenrec
   ...A.Q...S..V.H(.R.).LFR.T...---S   O   afr   Aardvark
   .S.A.R..IWD.FLV(YR.)PLFN.V.C-----   X   mar   Opossum
   N..S.E..IR....S(.R.)...T.T..*----   O   mar   Tasmanian devil

NO 28
GN CFH
ID CFAH_HUMAN
MP 802
DE Complement factor H
CL African great apes
SQ 51
   WIHTVCINGRWDPEV(NCS)MAQIQLCPPPPQI   O   hum   Human
   ...............(...).............   O   hac   Chimp
   ...............(...).............   O   aga   Gorilla
   Q.RS.....I.....(T..).............   X   gra   Orangutan
   H.QA...........(T..)...M.........   X   ape   Gibbon
   R........K.....(T..)..-..........   X   cat   Rhesus macaque
   RL.A...........(T..).............   X   cat   Crab-eating macaque
   RL.A...........(T..).............   X   cat   Baboon
   RL.A...........(T..)....K........   X   cat   Green monkey
   QK.S...........(T.P).............   X   sim   Marmoset
   QK.S...........(...)K............   O   sim   Squirrel monkey
   YR.S..V......AL(R.T)-V...........   X   pri   Bushbaby
   SK.S...........(T.T)EVPV.........   X   eua   Treeshrew
   YE.ST..H.T.I..P(T.K)KIEKKF.......   X   gli   Lesser Egyptian jerboa
   YQQSI.........P(T.T)RVEKKS.......   X   gli   Prairie vole
   YK.SI.........P(T.T)-------------   X   gli   Chinese hamster
   YE.S..........P(T.T)RVEKES.......   X   gli   Golden hamster
   YERSI....K....P(..T)RKGKTS.......   O   gli   Mouse
   YE.SI.........P(..T)RNEKRF.......   O   gli   Rat
   LQ.SI........QP(S.T)KVEKIS.......   X   gli   Naked mole-rat
   LQ..T.V....E..P(T.T)KVEKP..A.....   X   gli   Guinea pig
   LQ.AI......A..P(T.T)KVEKAS.A.....   X   gli   Chinchilla
   PQ.S..........I(T.T)KEEKVS.......   X   gli   Brush-tailed rat
   NK.SI........KL(T.T)EVK..........   X   gli   Rabbit
   YK.ST....K.K.KL(..T)-------------   O   gli   Pika
   SKYS.....V...R.(S.K)--EVNS.......   X   lau   Pig
   HKYS..........L(T.E)E..V........F   X   lau   Bactrian camel
   YK.SI.........A(T.K)E....S.......   X   lau   Dolphin
   YK.SI........KA(T.K)E....S.......   X   lau   Killer whale
   HKYSI....I.....(T.R)G...R--S..AV.   X   lau   Tibetan antelope
   YK.ST.V....E...(T.T)EV.M.........   X   lau   Horse
   YK.SI.........I(P.T)EL.M.SG......   X   lau   Cat
   YK.SI..........(A.T)EL.T.S......-   X   lau   Dog
   SKLST......N..L(S.T)EVIT.P.......   X   lau   Ferret
   QKYS..........L(S.A)EVRT.S.......   X   lau   Panda
   QK.ST.........L(..T)EV.T.S.......   O   lau   Weddell seal
   QKYS..........I(T.T)--EV.R.....R.   X   lau   Megabat
   QKRS..VH.K.E...(T.T)-------------   X   lau   David's myotis bat
   QK.S..V..K.E...(..T)E.PR--.......   O   lau   Microbat
   NKQST....N.E..I(..M)EV.T.P.A.....   X   lau   Hedgehog
   IKFST....N....I(..K)GQT..V.......   X   lau   Shrew
   RKNST....K.N..I(..N)EVSK.........   X   lau   Star-nosed mole
   NKQ.T.........L(T.T)EEIKKP.....H.   X   afr   Elephant
   YKQST......F..P(T.T)EILKTS.....H.   X   afr   Cape elephant shrew
   HKQST........KL(T.T)EEKK.......H.   X   afr   Manatee
   TRQST.K..M...TL(T.E)GQMK.......L.   X   afr   Cape golden mole
   -KKSI....V...KI(T.A)EEKT.V.....L.   X   afr   Tenrec
   FKQST.M......PL(..T)V..KKF.......   O   afr   Aardvark
   FKS.K......S.SL(S..)G---KS.A.....   X   mar   Opossum
   F.S.K.T....L..L(Q.P).---K..A.....   X   mar   Tasmanian devil
   TRIP-....K.QQLP(T.L)RERRKS.S.....   X   mon   Platypus

NO 29
GN CFH
ID CFAH_HUMAN
MP 822
DE Complement factor H
CL primates
SQ 50
   QIQLCPPPPQIPNSH(NMT)TTLNYRDGEKVSV   O   hum   Human
   ...............(...).............   O   hac   Chimp
   ...............(...).............   O   aga   Gorilla
   ...............(...).............   O   gra   Orangutan
   .M.............(...).............   O   ape   Gibbon
   -.............R(...)..M..Q.....A.   O   cat   Rhesus macaque
   ..............R(...).....Q......L   O   cat   Crab-eating macaque
   ..............W(T..).............   X   cat   Baboon
   ..K...........W(T.K).............   X   cat   Green monkey
   ..............L(.I.).....Q....I..   O   sim   Marmoset
   ..............V(.I.).....Q......L   O   sim   Squirrel monkey
   .............AW(...)..V..Q.......   O   pri   Bushbaby
   PV...........AQ(D.K)..V..Q.......   X   eua   Treeshrew
   EKKF.........AQ(D.R)..VK.Q....I..   X   gli   Lesser Egyptian jerboa
   EKKS.........AQ(V.E)..VK.W.......   X   gli   Prairie vole
   EKES.........AQ(VIE)..VK.L.......   X   gli   Golden hamster
   GKTS.........TQ(VIE)..VK.L....L..   X   gli   Mouse
   EKRF.........AQ(VIE)..VK.L.......   X   gli   Rat
   EKIS........KAQ(..I)..VK.D....I.I   X   gli   Naked mole-rat
   EKP..A......KAQ(..I)..VK.K...R..I   X   gli   Guinea pig
   EKAS.A.......AQ(..I)..VK.K......I   X   gli   Chinchilla
   EKVS........KAQ(..M)..VK.E...T..I   X   gli   Brush-tailed rat
   K...........Y.Q(.V.)..V..Q....M.I   O   gli   Rabbit
   EVNS.........AQ(D..)..V..K....I.I   X   lau   Pig
   .V........F..TR(S..)..V..QN......   X   lau   Bactrian camel
   ...S..........R(...)..VT.Q....I.I   O   lau   Dolphin
   ...S..........R(...)..VT.Q....I.I   O   lau   Killer whale
   ..R--S..AV.S.TA(D..)...KCH....I.I   X   lau   Tibetan antelope
   .M...........AQ(...)..V..Q....IFI   O   lau   Horse
   EV.S.........AQ(D..)..V.....G.I..   X   lau   White rhinoceros
   .M.SG........AR(D..)..VK......I..   X   lau   Cat
   .T.S......-..AQ(D..)..V..Q..G.I.I   X   lau   Dog
   IT.P.........AQ(..A)..V..Q.......   X   lau   Ferret
   RT.S.........AQ(...)..V..Q.......   O   lau   Panda
   .T.S.........AQ(..A)..V..Q.......   X   lau   Weddell seal
   EV.R.....R..RAR(...)..V..Q..D.I.I   O   lau   Megabat
   PR--..........Q(...)L.V..QE...I.I   O   lau   Microbat
   .T.P.A.......AE(A..)..V..Q..Q.I.I   X   lau   Hedgehog
   T..V..........Q(I..)..V......RI.I   X   lau   Shrew
   SK.........LHAE(A..)..V..N...RI.L   X   lau   Star-nosed mole
   IKKP.....H...TQ(...)M.V..QE...I.I   O   afr   Elephant
   LKTS.....H...AR(D..)..V..QN.....L   X   afr   Cape elephant shrew
   KK.......H...TR(D..)..V..Q....I.I   X   afr   Manatee
   MK.......L...TR(...)V.V..K....I.I   O   afr   Cape golden mole
   KT.V.....L...AQ(T..)..V..QN..T..I   X   afr   Tenrec
   .KKF..........E(S..)V.V..Q....I.I   X   afr   Aardvark
   --KS.A........Q(D.N)I.I..E....IGI   X   mar   Opossum
   --K..A.......AQ(D..)..VS.Q....IVI   X   mar   Tasmanian devil
   -MKP.A.......AQ(D.S).MV..E....IG.   X   mar   Wallaby
   RRKS.S......QAL(TVS).SVSFLN..S.T.   X   mon   Platypus

NO 30
GN CFH
ID CFAH_HUMAN
MP 911
DE Complement factor H
CL apes
SQ 51
   KLSYTCEGGFRISEE(NET)TCYMGKWSSPPQC   O   hum   Human
   ...........L...(...).............   O   hac   Chimp
   ........S..L...(...).............   O   aga   Gorilla
   ...........L...(...).............   O   gra   Orangutan
   ...........L...(...).............   O   ape   Gibbon
   .......Y..WL...(..I)...........H.   X   cat   Rhesus macaque
   .......Y..WL...(..I).............   X   cat   Crab-eating macaque
   .......D...L...(..I)...........H.   X   cat   Baboon
   .......D...L.K.(..I)...........H.   X   cat   Green monkey
   ...F...D...L...(..I)..S.....L....   X   sim   Marmoset
   ...F...D.......(..I)..F..........   X   sim   Squirrel monkey
   ....I..D......D(..I)...L.........   X   pri   Bushbaby
   ....V.DD...M..G(D.I).........-...   X   eua   Treeshrew
   ..N.I..D..TA...(D.I)........P..R.   X   gli   Squirrel
   ..G.I..H..TL.HA(HG.)M.H..........   X   gli   Lesser Egyptian jerboa
   T...V.DD...MA..(HGV)..HL....A..R.   X   gli   Prairie vole
   T.N.V.DD..MMA.D(HGV)..H........R.   X   gli   Chinese hamster
   T...V.DD...MA.D(HGV)..H........H.   X   gli   Golden hamster
   TF..V.DD....P..(.RI)........T..R.   X   gli   Mouse
   TF..V.DD.......(.RV)..N......L.R.   X   gli   Rat
   ..N....D..VL...(GGI)..H.....L....   X   gli   Naked mole-rat
   ..N....D..VL.SG(QGI)..HL....L..E.   X   gli   Guinea pig
   ..N....D..VL...(YGI)..HL....L..E.   X   gli   Chinchilla
   ..N....D..VL.AQ(DGI)..H........E.   X   gli   Brush-tailed rat
   ....V..D..S..GG(DGI)..H........L.   X   gli   Rabbit
   ..N....D..TL...(DG.)...........R.   X   lau   Bactrian camel
   .......D..S....(.VI)..H........H.   X   lau   Dolphin
   .......D..S....(.VI)..H........R.   X   lau   Killer whale
   ....I..E....F..(DGI).........S...   X   lau   Tibetan antelope
   R.N.I..D..SL..G(DGI)R.H..........   X   lau   Horse
   R...I.DD.....G.(DRI)..HL.........   X   lau   White rhinoceros
   ....I..D.Y...A.(DKI)..H..........   X   lau   Cat
   ....I.KD.....G.(DGI).............   X   lau   Dog
   ......KD..L..GK(E.I)..E......L.R.   X   lau   Ferret
   .......E......N(D.I)..Q......L.W.   X   lau   Panda
   ..N....D......K(D.I)..Q......L.R.   X   lau   Weddell seal
   ....I..D...L...(DGI)..N..........   X   lau   Megabat
   T...I.KD..KL.K.(DGI)..HL....AS...   X   lau   David's myotis bat
   T...I.KD..KL.K.(DGI)..HL.Q..AS...   X   lau   Microbat
   ....I..E.....ND(DGI)..H..V.......   X   lau   Hedgehog
   I.N...QA..K....(DKI)K.F..E..L..R.   X   lau   Shrew
   .F..I.DD..KT.A.(E.I)..H......L...   X   lau   Star-nosed mole
   ..T.V..E..KL...(DGI)..HL.........   X   afr   Elephant
   ..T.I..D...V...(..I)R.D.....T....   X   afr   Cape elephant shrew
   T.T.I..D..KL...(.GV)..H........H.   X   afr   Cape golden mole
   T.R....E..KL...(KGI)..H..........   X   afr   Tenrec
   .IT.V..E..MH.DD(GGI).............   X   afr   Aardvark
   I.....QQ..MMTG.(E.I)K.N........H.   X   mar   Opossum
   TIN.M.QK..KMVGG(E.I)..N......S.H.   X   mar   Tasmanian devil
   IVN.S..E..KMTGK(E.I)..N......T...   X   mar   Wallaby
   V.K...MD..TLDGT(A.I)..T.....P..K.   X   mon   Platypus

NO 31
GN CFH
ID CFAH_HUMAN
MP 1029
DE Complement factor H
CL apes
SQ 52
   VTYTCATYYKMDGAS(NVT)CINSRWTGRPTCR   O   hum   Human
   ...............(...).............   O   hac   Chimp
   ...............(...).............   O   aga   Gorilla
   .............P.(...).............   O   gra   Orangutan
   .......H.....P.(...).............   O   ape   Gibbon
   .......N.....P.(T..).............   X   cat   Rhesus macaque
   .......N.....P.(T..).............   X   cat   Crab-eating macaque
   .............P.(T..).............   X   cat   Baboon
   .......N.....P.(T..).............   X   cat   Green monkey
   ......P..Q...P.(T..)...R.........   X   sim   Marmoset
   ......P..Q...P.(T..)...RK........   X   sim   Squirrel monkey
   L..E.VAD.QL..PN(T..)..KRT.M.NI..K   X   pri   Bushbaby
   AI.K.P.N.QLV.SN(VI.)...G..V.T...K   X   eua   Treeshrew
   ...R.PKF.QV.RSN(TI.)...GK.I.K.M.K   X   gli   Squirrel
   ...E.TAS.QL..PN(A..).V.GK.I.K.V..   X   gli   Lesser Egyptian jerboa
   ..FK..PF.H.N.SN(I..).V.RT.I.KLV.K   X   gli   Prairie vole
   ..FK..PS.Q.N.SN(I..).V.RT.I.ELV.K   X   gli   Chinese hamster
   ..FR.QSP.Q.N.SD(T..).V....I.Q.V.K   X   gli   Mouse
   ..FR.PPP.R...SD(I..).V.TK.I.Q.V.K   X   gli   Rat
   -..K.PRS.QL..S.(I..).V.GK.I.E.K.K   X   gli   Naked mole-rat
   ...R.PSS.HLE.SD(T..).V.GK.I.E.K.K   X   gli   Guinea pig
   ...G.PQFHYL..SN(T..).A.GK.I.E.K.K   X   gli   Chinchilla
   .....P.F.Y...SN(A..).V.GK.I.E.K.K   X   gli   Brush-tailed rat
   .S.K.PEN.RL..PG(VI.)......I.K...K   X   gli   Rabbit
   .....PEN.R.T.S.(V..).V....I.A...Q   X   gli   Pika
   L.FK.QL...L..SN(T.E)....K.I...A..   X   lau   Bactrian camel
   .VFK.LPS.RLE..N(TIQ)..K.K.I...A..   X   lau   Dolphin
   .VFK.LPS.QLE..N(TIQ)..K.K.I...A..   X   lau   Killer whale
   .AFK.WPC.QL..YN(IIQ)...RK*....A..   X   lau   Tibetan antelope
   .AFK.RP..QLN.SN(TIQ).VK.K.I...V..   X   lau   Cow
   .AFK.QP..QL..SN(TIQ).VK.K.I...V..   X   lau   Sheep
   .AFK.QP..QL..SN(TIQ).VK.K.I...V..   X   lau   Domestic goat
   .A.K.PE..QL..S.(F.Q)...GK.I......   X   lau   Horse
   .I.K.PK..QL..SN(I.Q)Y.KNH.I...I..   X   lau   Dog
   ..FV.PKI.QL..PN(FIQ)..KGQ.I.E.K.K   X   lau   Ferret
   .....PK..EL..PN(FIQ)..K.Q.I.K.I.K   X   lau   Panda
   ...K.PK..E...PN(F.Q)..K.Q.I.K...K   X   lau   Weddell seal
   .I.R.EQ..Q.E.PK(TIR).LKG..I......   X   lau   Megabat
   ...Q.PD..Q...SD(V.Q).RDGI.I.T.K.K   X   lau   David's myotis bat
   ...R.PE..Q...SN(V.Q).KDGI.I.T...K   X   lau   Microbat
   .S.A.PEFHQ...SN(I.Q)...GN...N....   X   lau   Hedgehog
   ..FK.SEN.QL..P.(YAE).F..K.I.G.I..   X   lau   Shrew
   .....PK.HQI..SD(T.K)..K.K.I......   X   lau   Star-nosed mole
   .E.E.EKDFHL..SN(I.K)..K...I.S....   X   afr   Elephant
   LK.E.KPNFRL..SN(I.Q)..MAK.K.N...K   X   afr   Cape elephant shrew
   .E.K---NFQL.ESN(T.E)..KGK.I.S...K   X   afr   Manatee
   .E.E.EKNFQLN.SN(IIK)..K.K.V.T...K   X   afr   Cape golden mole
   .E.E.EKSFQL..SN(I.K)..K.K.I.T...K   X   afr   Tenrec
   .E.E.EKSFQLE.SN(I.K).FK.K.V.S.I.K   X   afr   Aardvark
   L..K.EQNFQL..SN(I..).V.GK.I.G...K   X   xen   Armadillo
   .V.Q.LSNFQPE.ST(E.I).TGPN.K.D.Q.K   X   mar   Opossum
   LL.K.LDGFQSE.ST(L..).SGTT.I.D.K.T   X   mon   Platypus

NO 32
GN CGB
ID CGHB_HUMAN
MP 50
DE Choriogonadotropin subunit beta
CL simians
SQ 51
   TLAVEKEGCPVCITV(NTT)ICAGYCPTMTRVL   O   hum   Human
   ...............(...)....-----....   O   hac   Chimp
   ...............(...).............   O   aga   Gorilla
   ............V..(...).............   O   gra   Orangutan
   ..PI.....S.....(...)......A.K....   O   ape   Gibbon
   ...A...A.......(...).........M...   O   cat   Rhesus macaque
   ...A...A.......(...).........M...   O   cat   Crab-eating macaque
   ...A...A.......(...).........M...   O   cat   Green monkey
   I..A........VAF(...)......SS.----   O   sim   Marmoset
   I...........VPF(...)......SS.SS.P   O   sim   Squirrel monkey
   ...A.N.A......F(T.S).......S.V...   X   pri   Bushbaby
   ..TA.N.A......F(T.S).......S.V...   X   eua   Treeshrew
   ...A.H.A......F(T.S).......S.V...   X   gli   Squirrel
   ...A.N.A.......(T.S).......S.V...   X   gli   Lesser Egyptian jerboa
   ...A...A......F(T.S).......S.E..V   X   gli   Prairie vole
   ...A.N.V......F(T.S).......S.V...   X   gli   Chinese hamster
   ...A.NDV......F(T.S).......S.V...   X   gli   Golden hamster
   ...A.N.F......F(T.S).......S.V...   X   gli   Mouse
   ...A.N.F......F(T.S).......S.V...   X   gli   Rat
   ...A...A..I.V.F(T.S).......S.M...   X   gli   Guinea pig
   ...A.N.A....V.F(T.S).......S.M...   X   gli   Chinchilla
   ...A...A....M.F(T.S).......S.V...   X   gli   Brush-tailed rat
   ...A.N.A......F(T.S).......S.V...   X   gli   Rabbit
   ...A...A.......(T.S).......S.V...   X   gli   Pika
   ...A.N.A......F(T.S).......S.V...   X   lau   Pig
   ...A.N.A......F(T.S).......S.V...   X   lau   Alpaca
   ...A.N.A......F(T.S).......S.V...   X   lau   Bactrian camel
   ...A.N.A......F(T.S).......S.V...   X   lau   Killer whale
   ...A...A......F(T.S).......S.K...   X   lau   Tibetan antelope
   ...A...A......F(T.S).......S.K...   X   lau   Cow
   ...A...A......F(T.S).......S.K...   X   lau   Sheep
   ...A...A......F(T.S).......S.----   X   lau   Domestic goat
   ...A...A..I...F(T.S).......S.V..M   X   lau   Horse
   ...A.N.A....V.F(T..).......S.M...   X   lau   Cat
   ...A.N.A......F(T..).......S.V...   X   lau   Dog
   ...A.N.A....V.F(T.S).......S.V...   X   lau   Ferret
   ...A.N.A......F(T..).......SLV...   X   lau   Pacific walrus
   ...A.N.A....V.F(T.S).......S.V...   X   lau   Black flying-fox
   ...A.N.A......F(T.S).......S.V...   X   lau   Big brown bat
   ...A.S.A..F...F(T.S).......S.V...   X   lau   Hedgehog
   ...A.N.A..F..AF(T.S).......S.V...   X   lau   Shrew
   ...A.N.A......F(T.S)......SSKM...   X   lau   Star-nosed mole
   ..TA...A......F(T.S)....C..LTV...   X   afr   Elephant
   ..GI.....S....L(T..).......S.....   X   afr   Cape elephant shrew
   ...A...A......F(T.S).......S.V...   X   afr   Manatee
   ..VA.N.A......F(T.S).......S.V...   X   afr   Cape golden mole
   ..VA.N.A..F...F(T.S).......S.V...   X   afr   Tenrec
   ...A.N.A......F(T.R).......S.----   X   afr   Aardvark
   ...A.N.A....V.F(T..).......S.V...   X   xen   Armadillo
   ...A.SDA....V.F(T..).......S.V...   X   mar   Opossum
   ...A.SDD....V.F(T..).......S.V...   X   mar   Tasmanian devil

NO 33
GN CLCA2
ID CLCA2_HUMAN
MP 150
DE Calcium-activated chloride channel regulator 2
CL simians
SQ 59
   EGKYIHFTPNFLLND(NLT)AGYGSRGRVFVHE   O   hum   Human
   ...............(...).............   O   hac   Chimp
   ...............(...).............   O   aga   Gorilla
   ...............(...).............   O   gra   Orangutan
   ...............(...).............   O   ape   Gibbon
   ...............(...).............   O   cat   Rhesus macaque
   ...............(...).............   O   cat   Crab-eating macaque
   ...............(...).............   O   cat   Baboon
   ...............(...).............   O   cat   Green monkey
   ...............(...)S............   O   sim   Marmoset
   .....R.........(...).............   O   sim   Squirrel monkey
   ........S....S.(..I).............   X   pri   Bushbaby
   ...............(...).............   O   eua   Treeshrew
   ..R............(D..).............   X   gli   Squirrel
   .............S.(D..)........A....   X   gli   Lesser Egyptian jerboa
   ...............(E.I).............   X   gli   Prairie vole
   ...H...........(E.A).............   X   gli   Chinese hamster
   ...............(E.A).............   X   gli   Golden hamster
   ..R......S.....(E.A)....A........   X   gli   Mouse
   ...............(E.A).............   X   gli   Rat
   ........S......(D..)TA...........   X   gli   Naked mole-rat
   ........S......(D.I)T............   X   gli   Guinea pig
   ........S......(D..)T............   X   gli   Chinchilla
   ........S......(D..)..........A..   X   gli   Brush-tailed rat
   .........A....E(KF.)....A........   X   gli   Rabbit
   .........A...SE(K..).............   X   gli   Pika
   .........D.....(A..).............   X   lau   Pig
   ...F.....D.....(D..).............   X   lau   Alpaca
   ...F..L..D.....(D..).............   X   lau   Bactrian camel
   ........LD.....(D..).V...........   X   lau   Dolphin
   ........LD.....(D..).V...........   X   lau   Killer whale
   ........SK.....(G..).............   X   lau   Tibetan antelope
   ........SK.....(G..).............   X   lau   Cow
   ......L.SE.....(G..).............   X   lau   Sheep
   ........SE.....(G..).............   X   lau   Domestic goat
   .........D.....(D..).......K.....   X   lau   Horse
   ........SD.....(D..).............   X   lau   White rhinoceros
   ........S......(D..)....P........   X   lau   Cat
   ........S......(D..)....P........   X   lau   Dog
   ........S......(D.I)....P........   X   lau   Ferret
   ........S...Q..(D..)....P...L....   X   lau   Panda
   ........S...I..(D..)....P........   X   lau   Pacific walrus
   ........S...V..(D..)....P........   X   lau   Weddell seal
   K.R..........TN(..I)--...........   X   lau   Black flying-fox
   ...H.Y...D.....(D..).............   X   lau   David's myotis bat
   ...H.Y...D.....(D..).............   X   lau   Microbat
   ...H.Y...D.....(D..)....P.....I..   X   lau   Big brown bat
   .........S.....(D.I)P............   X   lau   Hedgehog
   ........S....D.(D.I)..........A..   X   lau   Shrew
   ........SK.....(G..).............   X   lau   Star-nosed mole
   ..............E(..I)....P........   X   afr   Elephant
   ...........M...(...).A..P........   O   afr   Cape elephant shrew
   ..............S(DF.)....P........   X   afr   Cape golden mole
   .........S.....(...)....P........   O   afr   Tenrec
   ...............(...)T...P........   O   afr   Aardvark
   ...............(...).............   O   xen   Armadillo
   ...............(...).I..P........   O   mar   Opossum
   ..Q............(...).I...........   O   mar   Tasmanian devil
   ............V..(...).V...........   O   mon   Platypus

NO 34
GN CPN2
ID CPN2_HUMAN
MP 228
DE Carboxypeptidase N subunit 2
CL African great apes
SQ 60
   FGKLGSLQELFLDSN(NIS)ELPPQVFSQLFCL   O   hum   Human
   ...............(...).............   O   hac   Chimp
   ...............(...).............   O   aga   Gorilla
   .....N.K.......(K..).............   X   gra   Orangutan
   .S.............(K..)..-..........   X   ape   Gibbon
   .AR............(K..)....R......R.   X   cat   Rhesus macaque
   .AR............(K..)....R......R.   X   cat   Crab-eating macaque
   ..R............(K..)....R......R.   X   cat   Baboon
   ..R............(K..)....R......R.   X   cat   Green monkey
   .............G.(K..)...LE......R.   X   sim   Marmoset
   .............G.(K..)...LA......R.   X   sim   Squirrel monkey
   ..S............(RL.)....DA.......   X   pri   Bushbaby
   .RG..........G.(A..)....A.....L..   X   eua   Treeshrew
   ..N.CR.......G.(A.R)..S.N...G....   X   gli   Squirrel
   .SR.......Y....(V..)..S.R...E....   X   gli   Lesser Egyptian jerboa
   LDN..R.........(G.T)..S..A....LS.   X   gli   Prairie vole
   LAD............(A.E)..S.......LS.   X   gli   Chinese hamster
   LAD.V........N.(A.T)..S.R.....LS.   X   gli   Golden hamster
   L.S.S........G.(A.T)..S.HL.....S.   X   gli   Mouse
   LSN............(A.T)..S.HL..H.LS.   X   gli   Rat
   .AH..........G.(A.R)....RL..G..H.   X   gli   Naked mole-rat
   .AT....E.....N.(GLR)..S.ELL.G..R.   X   gli   Guinea pig
   .AA.R..E.......(A.C)....EL..G.SR.   X   gli   Chinchilla
   .AT.R..E......S(ALR)....EL..G..R.   X   gli   Brush-tailed rat
   ..R....R.......(AL.)....A...R.LS.   X   gli   Rabbit
   .DG..N.......G.(A..)..A.A...R..R.   X   gli   Pika
   ....S........G.(S..)....E..A..PH.   X   lau   Pig
   .SN.......S..G.(S..)...SE..A..SR.   X   lau   Alpaca
   .SN..R....S..G.(S..)...SE..A..SR.   X   lau   Bactrian camel
   .SH..G.R.....G.(S..)...A.A.AG.SR.   X   lau   Dolphin
   .SH..G.R.....G.(S..)...A.A.AG.SR.   X   lau   Killer whale
   .SR..........G.(S..)....E..A..S..   X   lau   Tibetan antelope
   .SR..........G.(S..)....E..A..S..   X   lau   Cow
   .SR..........G.(S..)..S.E..A..S..   X   lau   Sheep
   .SR..........G.(S..)K.S.E..A..S..   X   lau   Domestic goat
   .......R.....G.(S.K)...SE......R.   X   lau   Horse
   .............G.(S.A)...SE......R.   X   lau   White rhinoceros
   .D...C.R.....G.(S..)....T...G..R.   X   lau   Cat
   ..G.H..R.....G.(S..)....G...R..R.   X   lau   Dog
   .SQ..C.R.....G.(A.R)....G..AG..Q.   X   lau   Ferret
   .....G.R.....G.(S..)....GL..G.VR.   X   lau   Panda
   .S.............(L..)....G...G..Q.   X   lau   Pacific walrus
   .......R.......(L..)....G...G..Q.   X   lau   Weddell seal
   .A.....K.....G.(S..)...SE......N.   X   lau   Black flying-fox
   .A.....K.....G.(S..)...SE......N.   X   lau   Megabat
   .DG.SN....S.-.K(TR.)..S.GL..*---.   X   lau   David's myotis bat
   .EG..R.R.....G.(S..)....A......R.   X   lau   Microbat
   .AG.R..R.....G.(S..)....SL.....R.   X   lau   Big brown bat
   .SG....R.......(SLA)...EG.....PR.   X   lau   Hedgehog
   ..G..G....S....(AL.)...AGL..P.RG.   X   lau   Shrew
   .......R.....G.(A.L)D..QE.....VH.   X   lau   Star-nosed mole
   .D...........G.(S..)Q.H.....E.P..   X   afr   Elephant
   .D...........N.(V..)K..ARL..R.RS.   X   afr   Cape elephant shrew
   .............G.(L..)Q.H.K...E.V..   X   afr   Manatee
   .............G.(SL.)Q..SE........   X   afr   Cape golden mole
   ..R..R.R.....G.(AL.)R..AEL.AP.RR.   X   afr   Tenrec
   .S...........G.(S..)Q.S.E........   X   afr   Aardvark
   .A..R..R.......(S..)..S.E........   X   xen   Armadillo
   -ST.......A.QE.(QLR)R.A.GL.QGTRQ.   X   mar   Opossum
   .WT.AR.........(G.L)..S.DI..K.SQ.   X   mon   Platypus

NO 35
GN CRISP1
ID CRIS1_HUMAN
MP 230
DE Cysteine-rich secretory protein 1
CL primates
SQ 56
   DEYFDCDIQVHYLGC(NHS)TTILFCKATCLCD   O   hum   Human
   .........--....(...).............   O   hac   Chimp
   .......V.--....(...)..V..........   O   aga   Gorilla
   .........--....(...).............   O   gra   Orangutan
   .........--....(...).............   O   ape   Gibbon
   ...T..SLE--F...(...).PRM.........   O   cat   Rhesus macaque
   ...T..SLE--F...(...).PRM.........   O   cat   Crab-eating macaque
   ...T..SLE--F...(...).PRM.........   O   cat   Baboon
   ...T..GLE--F...(...).PRM..Q.S.R..   O   cat   Green monkey
   ...Y...E.--....(...).............   O   sim   Marmoset
   ...Y...V.--F...(...)............N   O   sim   Squirrel monkey
   ...TY.EQH--HF..(.NT)ANE.........N   O   pri   Bushbaby
   N..R..L.Y--GAK.(SQM)PFK.........G   X   eua   Treeshrew
   ...N..ETK--I...(K.P)SVK.........K   X   gli   Squirrel
   ...NN..K.--V...(E.P)SVK.L...S...T   X   gli   Lesser Egyptian jerboa
   ...NN..K.--LI..(S..)SVLK........K   X   gli   Prairie vole
   ...NN..K.--TK..(S.P)AVL.....S...K   X   gli   Chinese hamster
   ...NN..K.--LR..(L.Q)SVL.....S...R   X   gli   Golden hamster
   ...NN..T.--LY..(S.P)AVQP....S...T   X   gli   Mouse
   ...NN..K.--L...(S.P)AVRP....F...K   X   gli   Rat
   K..NNPE.R--VFE.(AT*)L-----------.   X   gli   Chinchilla
   ..RTN.E..--H...(S.P)SVQ.....S.N..   X   gli   Rabbit
   ..LTN.ETR--A...(S..)SLK.L.N...N..   X   gli   Pika
   ...SN.KA.--GP..(S.V)SVQQL...S...H   X   lau   Pig
   ...IN.K..--G...(S.L)SVQ.L...S...H   X   lau   Alpaca
   ...IN.K..--G...(S.L)SVQ.L...S...H   X   lau   Bactrian camel
   ...SN.KT.--GPA.(S.L)SVQRL...S..YP   X   lau   Dolphin
   .G.SN.KT.--GPA.(S.L)SVQRL...S...P   X   lau   Killer whale
   ...NN.NT.--R...(..L)SVQRL...S.T.Q   X   lau   Tibetan antelope
   ...NN.NT.--R...(..L)SVQRL...S.M.Q   X   lau   Cow
   ..HNN.NT.--H...(..L)SVQRL...S.M.Q   X   lau   Sheep
   ...NN.NT.--R...(..L)SVQRL...S.M.Q   X   lau   Domestic goat
   ..SNK.KT.--A...(S.L)SVK..........   X   lau   Horse
   ...SN.TT.--V...(R.V)SVR.L...S....   X   lau   White rhinoceros
   ...NN.KT.--SF..(SRQ)WVQQ....S....   X   lau   Cat
   ...SN.Q..--SF..(SPQ)SVQQ....S....   X   lau   Dog
   ...SN.KA.--SF..(GSQ)SVQQ....S....   X   lau   Ferret
   ...SN.QT.--SF..(RSQ)SVQQ....S....   X   lau   Panda
   ...SN.KT.--SF..(RAQ)SVQQ....S....   X   lau   Pacific walrus
   ...SN.KT.--SF..(RSQ)SVQQ....S....   X   lau   Weddell seal
   ...T..KL.--V...(S.P)SAQ.LYI.S....   X   lau   Black flying-fox
   ...T..KL.--V...(S.P)SAQ.LYI.S....   X   lau   Megabat
   ...T.....--VP..(RAL)SVT.Q.......T   X   lau   David's myotis bat
   ...I..H.R--VP..(SAL)SVT.Q.....F.T   X   lau   Microbat
   ...TN.H..--FP..(SAL)SVT.Q.....F.T   X   lau   Big brown bat
   ..HDK.KSH--NI..(..L)SVK.M...S...E   X   lau   Hedgehog
   ..NT...EL--SG..(D.P)VYYQL.....V.E   X   lau   Shrew
   ..QTN.KAK--HQE.(SQL)SVQQL...S.Q..   X   lau   Star-nosed mole
   ..LIN.KT.--R...(S.K)MVERY...S.F.T   X   afr   Elephant
   ..VIN.KTR--RF..(S.K)VVQQY...S...T   X   afr   Manatee
   ..LIN.QL*--KI..(S.V)MVRTY...S...T   X   afr   Cape golden mole
   .AIII.KKK--K...(.DK)LVGQY...S...T   X   afr   Aardvark
   ..VIN.KER--A...(S.K)SVE.L...S...T   X   xen   Armadillo
   .DAGN.QML--S...(H.K)LVAKS...S.K.T   X   mar   Opossum
   .DLVN.QML--L.H.(..K)LVAIK...S.R.T   X   mar   Tasmanian devil
   .NASN.RL*---...(K.E)L.EAY...S.K.T   X   mon   Platypus

NO 36
GN CSF1R
ID CSF1R_HUMAN
MP 353
DE Macrophage colony-stimulating factor 1 receptor
CL simians
SQ 60
   YLGPFSDHQPEPKLA(NAT)TKDTYRHTFTLSL   O   hum   Human
   ...............(...).............   O   hac   Chimp
   ...............(...).............   O   aga   Gorilla
   ...............(...).........P...   O   gra   Orangutan
   ...............(...).............   O   ape   Gibbon
   ...............(...).............   O   cat   Rhesus macaque
   ...............(...).............   O   cat   Crab-eating macaque
   ...............(...).............   O   cat   Baboon
   ...............(...).............   O   cat   Green monkey
   ...........L...(.V.).NS..........   O   sim   Marmoset
   .........L.....(.V.)SNS..........   O   sim   Squirrel monkey
   .Q....E..---..D(F..)......Y.S....   X   pri   Bushbaby
   .......Y.---..D(FV.)N.E...Y.SS...   X   gli   Squirrel
   .......Y.---T.D(FV.)N..R..Y.SV...   X   gli   Lesser Egyptian jerboa
   .....FGD.---N.D(FI.)QRT..SY.S..I.   X   gli   Prairie vole
   .....FED.---NPD(FTI)QRT...Y.SK.T.   X   gli   Chinese hamster
   .....FED.---..D(FT.)QRT.C.Y.SK.I.   X   gli   Golden hamster
   .....FED.---..E(FI.)QRAI..Y..K.F.   X   gli   Mouse
   .....FEDP---N.E(FR.)QWT..SYS.K.H.   X   gli   Rat
   ........E---..N(FN.)I.S...YSSS.F.   X   gli   Naked mole-rat
   ........K---..N(FNI)..G...Y.SS.I.   X   gli   Guinea pig
   ........K---.PN(FN.)I.S...Y.SS.F.   X   gli   Chinchilla
   ........K---..N(FN.)I.S...Y.SS.F.   X   gli   Brush-tailed rat
   .......YE---..E(FI.)N..M..Y.SS...   X   gli   Rabbit
   .........---..D(FV.)I.K...Y.SS...   X   gli   Pika
   .Q.....Q.---E.S(FE.)S.....YAS..T.   X   lau   Pig
   .Q...FG..---..N(FV.)I.....Y.SI.T.   X   lau   Alpaca
   .Q...FG..---..N(FV.)I.....Y.SV.T.   X   lau   Bactrian camel
   .R...LG..---..N(FV.)N.G...Y.S..T.   X   lau   Dolphin
   .R...LG..---..N(FV.)N.G...Y.S..T.   X   lau   Killer whale
   .E...FGS.---..N(FV.).S....YISK.T.   X   lau   Tibetan antelope
   .E...FGS.---..N(FE.).NN...Y.SK.T.   X   lau   Cow
   .E...FGS.---..N(FV.).S....Y.SK.T.   X   lau   Sheep
   .E...FGS.---..N(FV.).S....Y.SK.T.   X   lau   Domestic goat
   .W......P---..T(FT.)VE....YSS....   X   lau   Horse
   .Q.......---..N(FV.)I.....YAS....   X   lau   White rhinoceros
   .......Y.---..D(FV.)I.....Y.S....   X   lau   Cat
   .......Q.---..K(FVI)......Y.S....   X   lau   Dog
   .........---..N(FVM)M.....Y.SI...   X   lau   Ferret
   ......V..---..N(FV.)M.....Y.S....   X   lau   Panda
   .........---..N(FVM)L.....Y.SI...   X   lau   Pacific walrus
   .........---..N(FVM)M.....Y.SI...   X   lau   Weddell seal
   ......N..---..N(FV.)I.....Y.S..Y.   X   lau   Black flying-fox
   ......N..---..N(FV.)I.....Y.S..Y.   X   lau   Megabat
   .G.......---..S(FN.)R.E...Y.S....   X   lau   David's myotis bat
   .G.......---..N(FDA)H.E...Y.S....   X   lau   Microbat
   .R.......---..N(FV.)..E...Y.S....   X   lau   Big brown bat
   .M......H---N.E(FT.)N.TP..Y.S.VL.   X   lau   Hedgehog
   .R...P...---..N(FI.)K..A..YSS..L.   X   lau   Shrew
   .......Q.---..N(FDI)I.....Y.S....   X   afr   Elephant
   .....PGR.---..S(FD.)VR....GMS..Y.   X   afr   Cape elephant shrew
   .......Q.---S.N(FDI)IE....Y.S....   X   afr   Manatee
   ......S..---EFD(FVI)IN....Y.SI...   X   afr   Cape golden mole
   .Q...FNQ.---..D(YIL)...K..Y.SI...   X   afr   Tenrec
   .......-----..N(F..).NN...Y.S....   X   afr   Aardvark
   .......P.---..N(FDI).EC...C.S....   X   xen   Armadillo
   .D...LNS.---S.P(.SI)SET...FNT..P.   X   mar   Opossum
   .D...P.SH---R.Q(TSL)SGK...FNTI.T.   X   mar   Tasmanian devil
   .D...L.SH---G.L(TSI)SGE...YNTI.T.   X   mar   Wallaby
   -RR.VP.RR---.FP(AVL).GE...YDT..A.   X   mon   Platypus

NO 37
GN CSF2RB
ID IL3RB_HUMAN
MP 191
DE Cytokine receptor common subunit beta
CL euarchonts
SQ 57
   KRLQDSWEDAAILLS(NTS)QATLGPEHLMPSS   O   hum   Human
   ...............(...).............   O   hac   Chimp
   ...............(...).............   O   aga   Gorilla
   ...........T...(...)..A.R........   O   gra   Orangutan
   ...........T...(.I.).............   O   ape   Gibbon
   ...........T...(.A.).............   O   cat   Rhesus macaque
   ...........T...(.A.).............   O   cat   Crab-eating macaque
   ...........TF..(.A.).............   O   cat   Baboon
   ...........TF..(...).............   O   cat   Green monkey
   ...........S...(.A.).....L.......   O   sim   Marmoset
   ...........T...(.A.)H............   O   sim   Squirrel monkey
   R....P....PS.R.(...).VV...S......   O   pri   Bushbaby
   R..H......TTIY.(.S.)..V........H.   O   eua   Treeshrew
   R..S....E.SS.Y.(RS.)P.V..A.L-----   X   gli   Squirrel
   R..........HVRV(SA.)STL...PRVL...   X   gli   Lesser Egyptian jerboa
   .....T....PR.NT(SDL)WVS.ASKLFL..N   X   gli   Prairie vole
   ..........PS.HT(SNL)W...E.KL.L..N   X   gli   Chinese hamster
   ..........YS.HT(SKF).VNFE.KLFL.N.   X   gli   Mouse
   ..........SS.HT(SNL)WV..E.KLFL.N.   X   gli   Rat
   ...HE...N.ST.H.(SS.).V.....I.L...   X   gli   Naked mole-rat
   ...HEP..S.ST.H.(.S.)..A....LFL...   O   gli   Guinea pig
   ...HE...S.TT.H.(.S.).VA...KL.L.N.   O   gli   Chinchilla
   ...DE...N.TS.H.(SS.).VS...DL.LSD.   X   gli   Brush-tailed rat
   R..H....E.TS...(.S.)..I......T.N.   O   gli   Rabbit
   ...H....E.SS.VA(.S.).VI.D....W...   O   gli   Pika
   R.........HTIH.(GSP).V.......L...   X   lau   Pig
   R.........PTIY.(SA.)R.I......I...   X   lau   Dolphin
   R.........PTIY.(SA.)R.I..........   X   lau   Killer whale
   ..........H.IH.(TSP)..I.ELKY.L...   X   lau   Tibetan antelope
   ..........LTIH.(KSP)..I.ELKD.Q...   X   lau   Cow
   ..........HTIH.(TSP)..I.ELKY.L...   X   lau   Sheep
   ..........HTIH.(TSP)..I.ELKY.L...   X   lau   Domestic goat
   R.........PT...(DSF)..I......L...   X   lau   Horse
   RQ........PT...(.S.)..V......L...   O   lau   White rhinoceros
   ..........PT.YC(.S.)..V......L...   O   lau   Cat
   ........E.ST.HL(.S.)R.V......V...   O   lau   Dog
   ........E.ST.YL(.S.)..I.ES...I.G.   O   lau   Ferret
   ........E.ST.QV(.S.)..I.E....I...   O   lau   Panda
   ........E.ST.YF(KS.)..V.A....I...   X   lau   Pacific walrus
   ........E.ST.YF(KS.)..V.A....I...   X   lau   Weddell seal
   R.......E.PT.H.(TSP)K.I......IA..   X   lau   Black flying-fox
   R.......E.PT.H.(TSP)K.I......IA..   X   lau   Megabat
   ---------.PR.P.(TAP)..H...G..I...   X   lau   David's myotis bat
   ..........SS.N.(TAP)..H...G..I.G.   X   lau   Big brown bat
   R.........HTIHT(...)R.....QQ.L...   O   lau   Hedgehog
   R........TSTVYA(SSP)RVV.ARQL.P.--   X   lau   Shrew
   R.......E.VTVH.(SSQ)......P..L..T   X   lau   Star-nosed mole
   R.......E.ST.Y.(SS.)..V......V...   X   afr   Elephant
   R.......E.ST.SI(.S.)R.I......I...   O   afr   Cape elephant shrew
   R.H.......TT.Y.(.S.)..S......V.N.   O   afr   Cape golden mole
   R.......E.ST.Y.(.S.).....TGQ.V...   O   afr   Tenrec
   R.......E..T.Y.(.S.)..V.E..D.I...   O   afr   Aardvark
   R.......E.PT...(SSP)..M....L.V...   X   xen   Armadillo
   ..QWE...FFS.HP.(S..)HV.F.HD..I.DN   X   mar   Opossum
   ..RWE...LFS.HH.(SEA)HVL.EYD..I.ND   X   mar   Tasmanian devil
   --------------.(SM.)PVS..HD..ISDN   X   mar   Wallaby
   ..QR......SSRYL(.RT).MI.--R.PI.G.   O   mon   Platypus

NO 38
GN CTSC
ID CATC_HUMAN
MP 119
DE Dipeptidyl peptidase 1
CL primates
SQ 61
   FFKYKEEGSKVTTYC(NET)MTGWVHDVLGRNW   O   hum   Human
   ...............(...).............   O   hac   Chimp
   ...............(...).............   O   aga   Gorilla
   ...............(...).............   O   gra   Orangutan
   ...............(...).............   O   ape   Gibbon
   ........I...I..(...).............   O   cat   Rhesus macaque
   ........I...I..(...).............   O   cat   Crab-eating macaque
   ........I...I..(...).............   O   cat   Baboon
   ........I...I..(...).............   O   cat   Green monkey
   ............S..(...).............   O   sim   Marmoset
   ........G...S..(...).............   O   sim   Squirrel monkey
   ........R...S..(...).P...........   O   pri   Bushbaby
   ........T...S..(H..).............   X   eua   Treeshrew
   ....EAK..R.IS..(H..).P...........   X   gli   Squirrel
   ....EVK.RQ.IS..(H..).............   X   gli   Lesser Egyptian jerboa
   ....EVK..TAIS..(H..).............   X   gli   Prairie vole
   ....ETK....IS..(H..).............   X   gli   Chinese hamster
   ....ETK....IS..(H..).............   X   gli   Golden hamster
   ....EVR.HTAIS..(H..).............   X   gli   Mouse
   ....EVK..RAIS..(H..).............   X   gli   Rat
   ....ETK....IS..(Q..).............   X   gli   Naked mole-rat
   ....ETK.I..IS..(H..).I...........   X   gli   Guinea pig
   ....ETK..E.IS..(H..).............   X   gli   Chinchilla
   ....ETQ..T.VS..(H..).............   X   gli   Brush-tailed rat
   ........R...S..(H..).P...........   X   gli   Rabbit
   .......SGM..S..(...).P...........   O   gli   Pika
   ........R...S..(...).............   O   lau   Pig
   ....E...G...S..(H..).............   X   lau   Alpaca
   ....E...G...S..(H..).............   X   lau   Bactrian camel
   ........G...S..(H..).............   X   lau   Dolphin
   ........G...S..(H..).............   X   lau   Killer whale
   ........GE..S..(H..).............   X   lau   Tibetan antelope
   ........G...S..(H..).............   X   lau   Cow
   ........G...S..(H..).............   X   lau   Sheep
   ........G...S..(H..).............   X   lau   Domestic goat
   ....E...IQ..S..(...).P...........   O   lau   Horse
   ....E...T...S..(...).P...........   O   lau   White rhinoceros
   ....E..CG...S..(Q..).P...........   X   lau   Cat
   ........H...S..(...).............   O   lau   Dog
   ........HT..S..(...)LP...........   O   lau   Ferret
   ........HT..S..(...)LP...........   O   lau   Panda
   ........HT..S..(...)LP...........   O   lau   Pacific walrus
   ........HT..S..(...)LP...........   O   lau   Weddell seal
   ........G...S..(...).V...........   O   lau   Black flying-fox
   ........G...S..(...).V...........   O   lau   Megabat
   .....Q......S..(D..).A...........   X   lau   David's myotis bat
   SVM..Q......S..(D..).............   X   lau   Microbat
   .....K..G...S..(D..).A...........   X   lau   Big brown bat
   .....Q......S..(H..).............   X   lau   Hedgehog
   ........G...S..(H..).P...........   X   lau   Shrew
   .....Q...Q.KS..(H..).P...........   X   lau   Star-nosed mole
   .....Q...R..S..(H..).P...........   X   afr   Elephant
   ........G...S..(Y..).P...........   X   afr   Cape elephant shrew
   .....Q...R..S..(Q..).P...........   X   afr   Manatee
   .....Q......S..(H..)LP...........   X   afr   Cape golden mole
   .....Q......S..(H..).P........Q..   X   afr   Aardvark
   ............S..(H..).P...........   X   xen   Armadillo
   .....S.AGN..S..(H..)FP........K..   X   mar   Opossum
   ........HN..S..(H..)FP........K..   X   mar   Tasmanian devil
   .....K.AHN..S..(H..)LP........K..   X   mar   Wallaby
   .....Q..TN..S..(H..)LP...........   X   mon   Platypus

NO 39
GN DMBT1
ID DMBT1_HUMAN
MP 1712
DE Deleted in malignant brain tumors 1 protein
CL humans and chimpanzees
SQ 52
   QGSGPIVLDDVRCSG(NES)YLWSCPHKGWLTH   O   hum   Human
   ...............(...).......N.....   O   hac   Chimp
   ...............(H..).......N.....   X   aga   Gorilla
   ...............(R..).......N.....   X   gra   Orangutan
   ...............(H..).......N.....   X   ape   Gibbon
   ...............(Q..)...N...R...S.   X   cat   Rhesus macaque
   ...............(Q..)...N...R...S.   X   cat   Crab-eating macaque
   ...............(Q..)...N...R...S.   X   cat   Green monkey
   ...............(Y..).......S...S.   X   sim   Marmoset
   ....L......A...(H..)....YR.N...L.   X   pri   Bushbaby
   ....Q....N.....(H..)...N...N...S.   X   eua   Treeshrew
   R.V...A....E.M.(T.A)R..Q.L.S..F..   X   gli   Squirrel
   .......M...A.N.(H..)...N.N.R...N.   X   gli   Lesser Egyptian jerboa
   ...........D.R.(Y.Y)H.A..S.R...S.   X   gli   Prairie vole
   P...S......A.T.(H.D)...R.S.R...S.   X   gli   Mouse
   ....L......S...(Y..)H..N.R.P...V.   X   gli   Rat
   ...........A...(H..)...N...R...S.   X   gli   Naked mole-rat
   ...........A...(H..)...N.S.R...S.   X   gli   Guinea pig
   ..N........A...(H..)...D...R...S.   X   gli   Chinchilla
   S..........A...(H..)...N.R.R...S.   X   gli   Brush-tailed rat
   ....SV...Y.GRL.(R..)...D.S.R...SC   X   gli   Rabbit
   ..T........S...(Q..)F....K.S.....   X   gli   Pika
   ......L..ELG...(..T)F.....NS..N..   O   lau   Alpaca
   .........N.G...(H..).......N..N..   X   lau   Killer whale
   ..........GG...(H.T)S....S.NP.N..   X   lau   Tibetan antelope
   ...........G...(Y.T).....S.SP.N..   X   lau   Cow
   .........N.G...(H.T).....S.SP.N..   X   lau   Sheep
   ...........G...(H.T).......NP.N..   X   lau   Domestic goat
   ...............(Q..).....T.N..NS.   X   lau   Horse
   .........N.....(Q..)...R...N..NS.   X   lau   Cat
   ..........L....(...)...N...N..NL.   O   lau   Dog
   .........N.....(H..).......N..NS.   X   lau   Ferret
   ...............(H..)...R...N..NL.   X   lau   Pacific walrus
   R.L...A....E.V.(T.A)R..K.L.G..FS.   X   lau   Weddell seal
   ...............(Q..).......R..NS.   X   lau   Black flying-fox
   ...............(Q..).......R..NS.   X   lau   Megabat
   ...........D...(Q..)F.....NR..NS.   X   lau   David's myotis bat
   ...........G...(H..)F..N...R..NS.   X   lau   Microbat
   ....S......A...(Q..).......R..KS.   X   lau   Big brown bat
   ...............(H..)...H...S..NS.   X   lau   Hedgehog
   -.T.R......Q.T.(K.A)R..Q.L.S..FS.   X   lau   Star-nosed mole
   R...Q....N.....(H..).......G...S.   X   afr   Elephant
   E.V.S.L......Q.(S.R)T.GQ.E.L.FSI.   X   afr   Cape elephant shrew
   ....Q....N.S...(H..).....R.S.L.S.   X   afr   Manatee
   ..T.Q....EL....(H..)...N...N...S.   X   afr   Cape golden mole
   ....Q......N.T.(Y..)...N...G...S.   X   afr   Tenrec
   ......I........(Q..).......N...S.   X   afr   Aardvark
   ....Q........L.(...)...N.S.N...S.   O   xen   Armadillo
   P...N.L..NMQ.V.(A.R)..GQ.S.S..SN.   X   mar   Opossum
   ..T.R..........(F.F)...Y...N...S.   X   mar   Tasmanian devil
   P...S.W..N.Q.K.(I..)S..DLA-SP.-GQ   X   mar   Wallaby
   P...R.L....N...(...)S.ED.A.N..G..   O   mon   Platypus

NO 40
GN DSG2
ID DSG2_HUMAN
MP 182
DE Desmoglein-2
CL euarchonts
SQ 59
   SVEELSAAHTLVMKI(NAT)DADEPNTLNSKIS   O   hum   Human
   ...............(...).............   O   hac   Chimp
   ...............(...).............   O   aga   Gorilla
   ...............(...).............   O   ape   Gibbon
   ...............(...).............   O   cat   Rhesus macaque
   ...............(...).............   O   cat   Crab-eating macaque
   ...............(...).............   O   cat   Baboon
   ...............(...).............   O   cat   Green monkey
   ......V.N......(...).............   O   sim   Marmoset
   ......V.N......(...).............   O   sim   Squirrel monkey
   ..A...S........(...).......M.....   O   pri   Bushbaby
   .I.............(...).......M.....   O   eua   Treeshrew
   ...............(T.I).......M...V.   X   gli   Squirrel
   ...............(T..).......M.....   X   gli   Lesser Egyptian jerboa
   .I............V(I..)...D.T.R.AE.A   X   gli   Prairie vole
   ..............V(I..).............   X   gli   Golden hamster
   .I.............(T..)...D.E...A.V.   X   gli   Mouse
   .I.............(T..)...D.Q.......   X   gli   Rat
   ...............(I..).......M.....   X   gli   Naked mole-rat
   .......PY......(T..).......I.....   X   gli   Guinea pig
   .I.............(T..).......M.....   X   gli   Chinchilla
   ...............(T.E).......M.....   X   gli   Brush-tailed rat
   .I.............(...).............   O   gli   Rabbit
   .I.....LN.F..R.(...).......M...V.   O   gli   Pika
   .I......N......(T..).............   X   lau   Pig
   ........N......(...).............   O   lau   Alpaca
   ........N......(...).............   O   lau   Bactrian camel
   ........N...I..(T..).............   X   lau   Dolphin
   ........N......(T..).............   X   lau   Killer whale
   ........N......(T..).............   X   lau   Tibetan antelope
   ........N......(...).............   O   lau   Cow
   ........N......(T..).............   X   lau   Sheep
   ........N......(T..).............   X   lau   Domestic goat
   .I...........TV(I..).......M.....   X   lau   Horse
   .I.............(I..).............   X   lau   White rhinoceros
   ........D.F....(S..).............   X   lau   Cat
   .I......D......(S..).............   X   lau   Dog
   ........D......(S..).............   X   lau   Ferret
   .......SD......(S..).............   X   lau   Panda
   .I......D.F....(S..).............   X   lau   Pacific walrus
   .I......D......(S..).............   X   lau   Weddell seal
   ...............(T..).............   X   lau   Black flying-fox
   ...............(T..).............   X   lau   Megabat
   ...............(...)......SM.....   O   lau   David's myotis bat
   ...............(...)......SM.....   O   lau   Microbat
   ...............(...)......SM.....   O   lau   Big brown bat
   ...............(...).......R.....   O   lau   Hedgehog
   ............T.V(...).......V..EL.   O   lau   Shrew
   Y.......R.F....(T..).......R..E..   X   lau   Star-nosed mole
   .....C.RG......(...).............   O   afr   Elephant
   AI....T.G.....V(S..).............   X   afr   Cape elephant shrew
   A....L.RD......(S..).......M.....   X   afr   Manatee
   ...............(T..).......M.....   X   afr   Cape golden mole
   .I......D.I....(...).......I.....   O   afr   Tenrec
   .I..M.....F....(...).............   O   afr   Aardvark
   .I.....VD......(S..).......P.....   X   xen   Armadillo
   ......P.N......(S..)....K.HI..QV.   X   mar   Opossum
   ......P.N......(S..)....EGHI.....   X   mar   Tasmanian devil
   Q...S.PPG.S..Q.(S..)..........R.T   X   mon   Platypus

NO 41
GN ENPEP
ID AMPE_HUMAN
MP 773
DE Glutamyl aminopeptidase
CL catarrhines
SQ 61
   REALNNASSLFEQWL(NGT)VSLPVNLRLLVYR   O   hum   Human
   ...............(...).............   O   hac   Chimp
   ...............(...)I............   O   aga   Gorilla
   ...............(S..).............   X   gra   Orangutan
   ........L......(...).............   O   ape   Gibbon
   ........F......(...).............   O   cat   Rhesus macaque
   ........F......(...).............   O   cat   Crab-eating macaque
   ........F......(...).............   O   cat   Baboon
   .D......F......(...).............   O   cat   Green monkey
   ....E...L......(T..)ERV..........   X   sim   Marmoset
   ....D...L...R..(T..)ERI..........   X   sim   Squirrel monkey
   ........Q......(S.N).R...........   X   pri   Bushbaby
   PD...S..Q..QE..(...)R............   O   eua   Treeshrew
   P...K...Q...D.I(KTG)K.I.......G..   X   gli   Squirrel
   KQ..D...R...E..(S..)..I..........   X   gli   Lesser Egyptian jerboa
   KD..AK..E...D.V(Q.G)K.I..........   X   gli   Prairie vole
   ........Q..DN..(QKN)ENI.......A..   X   gli   Chinese hamster
   ........K..DN.V(QKN)E.I..........   X   gli   Golden hamster
   ....G...Q..DS..(K.S)A.I..........   X   gli   Mouse
   G...G...Q...A..(K.N)E.I..........   X   gli   Rat
   T.......Q...L..(T..)..I..........   X   gli   Naked mole-rat
   K....S..Q..Q...(A.A)D.I.......A..   X   gli   Guinea pig
   K........Y..D.V(A.K)I.I..........   X   gli   Chinchilla
   KD.........KN.V(D.A)I.I..........   X   gli   Brush-tailed rat
   T.......Q..QE.I(..R)Q............   X   gli   Rabbit
   E...R...Q.....V(SAA)..P..........   X   gli   Pika
   SD......Q..QK..(T..)L............   X   lau   Alpaca
   SD......Q..QK..(T..).............   X   lau   Bactrian camel
   SD......Q..QA..(T..).............   X   lau   Dolphin
   SD......Q..QT..(T..).............   X   lau   Killer whale
   .D......Q..QE..(T..).............   X   lau   Tibetan antelope
   SD......Q..QE..(T..).............   X   lau   Cow
   SD......Q..Q...(T..).............   X   lau   Sheep
   SD......R..QE..(T..).............   X   lau   Domestic goat
   ....G...Q..QE..(S..)AR...........   X   lau   Horse
   A.......Q..Q...(S..).............   X   lau   White rhinoceros
   ....D..TQ..Q...(S..).R...........   X   lau   Cat
   Q......TQ..Q...(S..).R...........   X   lau   Dog
   Q...D..TQ..Q...(S..).R...........   X   lau   Ferret
   Q...E..TQ..Q...(S..)LR...........   X   lau   Panda
   Q...D..TQ..Q...(S..).R..T........   X   lau   Pacific walrus
   Q...D..TQ..Q...(S..).R..T........   X   lau   Weddell seal
   Q...G...E..Q...(S..).RI..........   X   lau   Black flying-fox
   Q...G...T..Q...(S..).RI..........   X   lau   Megabat
   K...G...Q..Q...(T..).RI..........   X   lau   David's myotis bat
   Q...G...Q..Q...(T..).RI..........   X   lau   Microbat
   K...G...Q..Q...(S..)..I..........   X   lau   Big brown bat
   Q....S..Q...E..(K.N)L.V..........   X   lau   Hedgehog
   PN..KS..EE.QK.I(ADP)Y.I.......A..   X   lau   Shrew
   Q...D...Q..QN..(SER)K............   X   lau   Star-nosed mole
   ........Q......(S..).............   X   afr   Elephant
   .D......Q...E..(K.N).............   X   afr   Cape elephant shrew
   SD......Q......(S.S).............   X   afr   Manatee
   KA..ED..Q...K..(S.S).............   X   afr   Cape golden mole
   QD......Q......(R.S)G............   X   afr   Tenrec
   ....G...H...E..(S.N)G............   X   afr   Aardvark
   TD...S..QR.....(..A)G............   X   xen   Armadillo
   PD......E..KK.Q(..R)Q--..........   X   mar   Opossum
   TD......E..K..Q(..S)Q--..........   O   mar   Tasmanian devil
   TD.....TD..KK.Q(K..)Q--..........   X   mar   Wallaby
   E...Q.......K..(T.S)Q--..........   X   mon   Platypus

NO 42
GN ENPP1
ID ENPP1_HUMAN
MP 748
DE Ectonucleotide pyrophosphatase/phosphodiesterase family member 1
CL primates
SQ 59
   TKVSYGFLSPPQLNK(NSS)GIYSEALLTTNIV   O   hum   Human
   ...............(...).............   O   hac   Chimp
   ------------...(...).............   O   aga   Gorilla
   ...............(...)R..........V.   O   gra   Orangutan
   ...............(...).............   O   ape   Gibbon
   ...............(...).............   O   cat   Rhesus macaque
   ...............(...).............   O   cat   Crab-eating macaque
   ...............(..R).............   X   cat   Baboon
   ...............(...).............   O   cat   Green monkey
   ...............(...).............   O   sim   Marmoset
   ...........H...(...).............   O   sim   Squirrel monkey
   AR......A..R.S.(...)Q.......F....   O   pri   Bushbaby
   .RL...........I(D.N)Q.N..........   X   eua   Treeshrew
   A.L.....A...I.R(D.N)Q......I.....   X   gli   Lesser Egyptian jerboa
   L.LT....A..R...(..N)Q........S.M.   X   gli   Prairie vole
   S.L.....A..R..T(IEN)Q........S...   X   gli   Chinese hamster
   S.L.....A..R...(L.N)K........S...   X   gli   Golden hamster
   S.L.....T..R..R(V.N)H........S...   X   gli   Mouse
   S.L.....T..R..R(V.R)Q........S...   X   gli   Rat
   A.M.....T....S.(D..)Q............   X   gli   Naked mole-rat
   A.L.....A....S.(YA.)Q............   X   gli   Guinea pig
   NRL.....T....S.(Y..)Q.........-..   X   gli   Chinchilla
   A.L.....T....S.(Y..)Q............   X   gli   Brush-tailed rat
   A.L..........S.(D..)Q........S...   X   gli   Rabbit
   A.L............(D..)Q........S...   X   gli   Pika
   A.L............(G..)QV.........M.   X   lau   Pig
   A.L............(G..)QV...........   X   lau   Alpaca
   A.L............(G..)QV...........   X   lau   Bactrian camel
   ..L............(G..)QV.........T.   X   lau   Dolphin
   ..L............(G..)QV...........   X   lau   Killer whale
   A.L..........H.(G..)QV...........   X   lau   Tibetan antelope
   A.L...L......H.(G..)QV...........   X   lau   Cow
   A.L..........H.(G..)QV...........   X   lau   Sheep
   A.L..........H.(G..)QV...........   X   lau   Domestic goat
   A.L............(G..)K.N..........   X   lau   Horse
   AQL.....A......(G..)K............   X   lau   White rhinoceros
   AEL.....Y......(G..)QL......N....   X   lau   Cat
   AEL.....Y......(G.N)QL......S....   X   lau   Dog
   AEL..E..Y......(G.D)QL......N....   X   lau   Ferret
   AEL.....Y......(G.N)QL......N....   X   lau   Panda
   AEL.....Y......(G.N)QL......N....   X   lau   Pacific walrus
   AEL.....Y......(G.N)QL......N....   X   lau   Weddell seal
   S.L............(G..)K............   X   lau   Black flying-fox
   S.L............(G..)K............   X   lau   Megabat
   A.M............(G.N)K............   X   lau   David's myotis bat
   A.M............(G.N)K............   X   lau   Microbat
   A.I............(G.N)K............   X   lau   Big brown bat
   A.H.....Y..G...(AL.)L......I.S...   X   lau   Hedgehog
   ..LN...........(V.N)K............   X   lau   Shrew
   V.L.....F.....N(V.N)KT...........   X   lau   Star-nosed mole
   ..F............(G.R)Q........S.V.   X   afr   Elephant
   V.L............(D..)Q.H..........   X   afr   Cape elephant shrew
   ..L............(G..)QT......S..L.   X   afr   Manatee
   V.L..........S.(D..)Q..........V.   X   afr   Cape golden mole
   A.L.........I..(G..)Q..........V.   X   afr   Aardvark
   P.L.....Y....T.(G.N)NR.Q...I.S..I   X   mar   Opossum
   P.LN.........T.(GTN)SRHH...I.S..I   X   mar   Tasmanian devil
   P.L..........T.(GTN)DRHH.....S.VI   X   mar   Wallaby
   PQLNP...F....R.(SLD)EVPF...I.S.V.   X   mon   Platypus

NO 43
GN ENPP7
ID ENPP7_HUMAN
MP 168
DE Ectonucleotide pyrophosphatase/phosphodiesterase family member 7
CL primates
SQ 58
   AVTRSRKEGIAHNYK(NET)EWRANIDTVMAWF   O   hum   Human
   ...............(...).............   O   hac   Chimp
   ...............(...).......A.....   O   aga   Gorilla
   ...............(...).............   O   gra   Orangutan
   ...............(...).............   O   ape   Gibbon
   ...............(...).............   O   cat   Rhesus macaque
   ...............(...).............   O   cat   Crab-eating macaque
   ...............(...).............   O   cat   Baboon
   ...............(...).............   O   cat   Green monkey
   .........VL....(...).....V.......   O   sim   Marmoset
   ...Q.....VL....(...).....V.......   O   sim   Squirrel monkey
   .L.L......L....(...)..........L..   O   pri   Bushbaby
   ...L.....VL.D..(..Q)..........R..   X   eua   Treeshrew
   ...L......L....(D.K)..........R..   X   gli   Squirrel
   ..AL.....VL....(D.K)...E.V....R..   X   gli   Lesser Egyptian jerboa
   ...M......F....(...)...E.V....K..   O   gli   Prairie vole
   ...M......F....(D.K)...E.V....R..   X   gli   Chinese hamster
   ...M......F....(D.K)...E.V....R..   X   gli   Golden hamster
   ...M.....VL....(...)...G.V....K..   O   gli   Mouse
   ...M.....VL....(...).....V....K..   O   gli   Rat
   .M.L.Q.........(D.Q).....V....K.L   X   gli   Naked mole-rat
   .L.LG.E..-S.S*R(D.Q).G.VSVH..LS..   X   gli   Guinea pig
   ...L.....PV....(D.Q).....V....R..   X   gli   Chinchilla
   ..SL.....AL....(D.E)...T.V....R.L   X   gli   Brush-tailed rat
   ...L.....AL...G(..S).....V....T..   O   gli   Pika
   ...L.....VL....(D.A)..........K..   X   lau   Pig
   ...L.....FL....(D.A)..........E..   X   lau   Alpaca
   ...L.....FL....(D.A)..........E..   X   lau   Bactrian camel
   ...L......L....(D.K)..........K..   X   lau   Dolphin
   ...L......L....(D.K)..........K..   X   lau   Killer whale
   ...L...Q..L....(D.A)..........K..   X   lau   Tibetan antelope
   ...L......L..F.(D.A)..........T..   X   lau   Cow
   ...L......L....(D.A)..........K..   X   lau   Sheep
   ...L...Q..L....(D.A).......M..K..   X   lau   Domestic goat
   ...L.....VL....(D.E)..........T..   X   lau   Horse
   .MML..E.S.L....(D.E).R.......LK..   X   lau   White rhinoceros
   ...L.....AL...G(..A)..K.......T..   X   lau   Cat
   ...L.....AL....(D.V)..K.......K..   X   lau   Dog
   ...L......L....(D.Q)...T......K..   X   lau   Ferret
   ...L.....VL....(...)R...D.....K..   O   lau   Pacific walrus
   ...L.....VL....(D.A)..........K..   X   lau   Weddell seal
   ...P.W..SVL..CR(D.K).G.PRT....TRL   X   lau   Black flying-fox
   D..P.W..SVL..CR(D.K).G.SHT....TRL   X   lau   Megabat
   ...L......L....(D.A).....V....R..   X   lau   Microbat
   ...L.....AL....(D.A).....V....R.L   X   lau   Big brown bat
   V..L..I.SVL....(...)..........R..   O   lau   Hedgehog
   ...Q..R..AL....(D.Q).........LG..   X   lau   Shrew
   R..Q..R..AL....(...)..........R..   O   lau   Star-nosed mole
   ..SQ.....VF....(D.Q)...R......N..   X   afr   Cape elephant shrew
   ...Q..R..VL.S..(D.Q)..........R..   X   afr   Manatee
   ...Q......F....(D.Q)..........R..   X   afr   Cape golden mole
   ...Q......F...S(K.Q)..........R..   X   afr   Tenrec
   ...Q.....VL....(D.Q).........IS..   X   afr   Aardvark
   .L.L.....VL....(...).....V....R.L   O   xen   Armadillo
   S..V..........G(..K)...R......K..   X   mar   Opossum
   Y..M......I.K.N(D.K)...Q......K..   X   mar   Tasmanian devil
   F.NV....SVW.K.N(D.I)...Q......T..   X   mar   Wallaby
   P..Q....PVG.K.N(...)...Q.....LR..   O   mon   Platypus

NO 44
GN F7
ID FA7_HUMAN
MP 382
DE Coagulation factor VII
CL simians
SQ 55
   TQDCLQQSRKVGDSP(NIT)EYMFCAGYSDGSK   O   hum   Human
   ......--.......(...).............   O   hac   Chimp
   ......--.......(...).............   O   aga   Gorilla
   ......--.......(...).............   O   gra   Orangutan
   ......--.......(...).............   O   ape   Gibbon
   ......--Q.AEA..(...)............R   O   cat   Rhesus macaque
   ......--..AEA..(...)............R   O   cat   Crab-eating macaque
   ......--..AEA..(...)............R   O   cat   Baboon
   ......--..AEA..(...)............R   O   cat   Green monkey
   .....E--E.TRGY.(.V.)........R....   O   sim   Marmoset
   .....E--E.T.G..(.V.)........R....   O   sim   Squirrel monkey
   ....E.--.RRAG.R(RL.).N......L....   X   pri   Bushbaby
   .....E--KQRP...(V..).N......L....   X   gli   Squirrel
   .....E--K.NP.T.(T..)DN......M..K.   X   gli   Lesser Egyptian jerboa
   .....E--KHSPST.(Q..)DN......M..T.   X   gli   Prairie vole
   .....E--KHSSNT.(K..)DN......V..T.   X   gli   Chinese hamster
   .....E--KHSSNT.(K..)DN......V..T.   X   gli   Golden hamster
   .....E--KHSSNT.(K..).N......M..T.   X   gli   Mouse
   .....E--KHSANT.(R..).N......M..T.   X   gli   Rat
   .....E--.RTPH..(V..)TN......L....   X   gli   Naked mole-rat
   N....E--QRTNS..(V..)KN......L..T.   X   gli   Guinea pig
   N....E--QWRNS..(A..)KN......L....   X   gli   Chinchilla
   N....E--QWRNT..(M..)KN......L....   X   gli   Brush-tailed rat
   ....VE--EHKPG..(EV.)GN......L....   X   gli   Rabbit
   .....E--DRKPG..(E..).N......L...Q   X   gli   Pika
   .....E--.RRPG..(S..)DN......L....   X   lau   Pig
   .....E--.LRPG..(AL.)DN......L....   X   lau   Alpaca
   .....E--.RRLG..(AL.)DN......L....   X   lau   Bactrian camel
   ....R.--.RRPSG.(V..)DN......T....   X   lau   Dolphin
   ....R.--.RRPGG.(VV.)DN......T....   X   lau   Killer whale
   ......--.QRPGG.(VV.)DN...........   X   lau   Cow
   ......--.QRPSG.(AV.)DN.....HT....   X   lau   Sheep
   .....E--HRMEG..(AL.).N......V..TQ   X   lau   Horse
   .....E--.RTAG..(AL.).N......L..TQ   X   lau   White rhinoceros
   ....QE--HRKAG..(A..).N......L....   X   lau   Cat
   ....QE--.RRSG..(A..).N......L....   X   lau   Dog
   ....QE--.QWEGL.(AV.).N......L....   X   lau   Ferret
   ....Q.--..WVG..(V..).N......L....   X   lau   Panda
   ....QE--.RREG..(T..).N......L....   X   lau   Pacific walrus
   ....QE--.RREG..(T..).N......L....   X   lau   Weddell seal
   .....E--TREQT..(K..).N......L....   X   lau   Black flying-fox
   .....E--TREQT..(K..).N......L....   X   lau   Megabat
   .....E--K.EAG..(VL.).N......R....   X   lau   David's myotis bat
   .....E--K.EAG..(E..).N......R....   X   lau   Microbat
   .....E--K.KAAA.(KL.).N......L....   X   lau   Big brown bat
   .....E--HRQPGA.(G..).N......L....   X   lau   Shrew
   ....NE--QRTAN..(VV.).N......L..T.   X   afr   Elephant
   ....NE--V.SSNT.(VL.).N......L..T.   X   afr   Cape elephant shrew
   ....KE--QRTAH..(VL.).N......L..T.   X   afr   Manatee
   ....KK--K.SINA.(E..).N......L..T.   X   afr   Cape golden mole
   ....KE--KRSINA.(EL.).N.....DL..H.   X   afr   Tenrec
   ....KE--QR.AN..(VM.).N......L..T.   X   afr   Aardvark
   ....KE--QRSANA.(VL.).N......V..T.   X   xen   Armadillo
   .....E---NEE.A.(.T.).N.....FL..T.   O   mar   Opossum
   ......---NKENI.(...).N.....FLN.T.   O   mar   Tasmanian devil

NO 45
GN FCER1A
ID FCERA_HUMAN
MP 99
DE High affinity immunoglobulin epsilon receptor subunit alpha
CL African great apes
SQ 58
   KFEDSGEYKCQHQQV(NES)EPVYLEVFSDWLL   O   hum   Human
   ...............(...).............   O   hac   Chimp
   ...............(...).............   O   aga   Gorilla
   ...............(K..).............   X   gra   Orangutan
   S..............(K..).............   X   ape   Gibbon
   D.............F(DD.)...H.........   X   cat   Rhesus macaque
   D.............F(DD.)...H.........   X   cat   Crab-eating macaque
   D..........Y..F(DN.)...H.........   X   cat   Baboon
   D.............F(DD.)...H.........   X   cat   Green monkey
   D.....Q....SPKS(...).....G.......   O   sim   Marmoset
   D...........PKF(...)D............   O   sim   Squirrel monkey
   S.H........Y.NF(K..)K.AH.........   X   pri   Bushbaby
   TVN.....R....KH(.K.)P.....I......   O   eua   Treeshrew
   TYQH..K.T.HGRNF(YK.)K......I...M.   X   gli   Squirrel
   N.Q...K....NKES(YK.)KAA....TR....   X   gli   Lesser Egyptian jerboa
   TIQ...K.V..N.GL(YK.)K......TR....   X   gli   Prairie vole
   TIQ...K.V..K.GL(YK.)K......MR....   X   gli   Chinese hamster
   TIQ...K.I..K.GF(YK.)K......TK....   X   gli   Golden hamster
   TVQ...K.I..K.GL(FK.)K....N.TQ....   X   gli   Mouse
   TIQ...K.I..K.GF(YK.)K....N.MQE...   X   gli   Rat
   NLQ.N.T.E..N.NL(PR.)D.M...L.R....   X   gli   Naked mole-rat
   SSQ.....R.CS.NL(YW.)NR.H...LK....   X   gli   Guinea pig
   STQH..K.E..N.NL(LR.)K..H....K....   X   gli   Chinchilla
   TLQ...K.E..Y.NL(RR.)Q..H...CKN...   X   gli   Brush-tailed rat
   T.QH..Q.Q..SPSF(.Q.)D............   O   gli   Rabbit
   TTG.Q...Q..SSGF(.K.)D..N.........   O   gli   Pika
   .PG...K.R..SKDF(TM.)...H...I.....   X   lau   Pig
   .A..G.Q.R.KS.TR(AI.).......I.....   X   lau   Alpaca
   .A....Q.R.KS.KR(TI.).......I.....   X   lau   Bactrian camel
   SIQ...D.R..SKGF(IM.).....D.I.....   X   lau   Dolphin
   SIQ...D.R..SKGF(FM.).....D.I.....   X   lau   Killer whale
   RMQ.....Q.RIKGF(AI.)D....N.I....I   X   lau   Tibetan antelope
   RMQ.....Q.RIKGF(AI.).....N.I....I   X   lau   Cow
   RVQ...K.Q.RIKGF(AI.).....N.I....I   X   lau   Sheep
   RMQ.....Q.RIKGF(AI.).....N.I....I   X   lau   Domestic goat
   SHRS....R.RNNDL(.L.).A.H.........   O   lau   Horse
   .SQNT...R.RNKKF(SL.).......S.....   X   lau   White rhinoceros
   QIR.....T..NKGS(ML.)K..S.K..RE...   X   lau   Cat
   QIQ.....R.RENRS(IL.)D....T..TE..I   X   lau   Dog
   HPQ.....R.RDKES(.V.)D........E...   O   lau   Ferret
   RIQ.....R..NKES(IP.).....G.VAG...   X   lau   Panda
   QIR.....R..NNGS(TP.).....R..AE...   X   lau   Pacific walrus
   QIQ.....R..NNGS(TP.).....S..AE...   X   lau   Weddell seal
   T.Q.....R.WNKNL(IP.)Q............   X   lau   Black flying-fox
   T.Q.....R.WNKNL(IP.)Q............   X   lau   Megabat
   ..Q.....R..SKNL(IP.)Q..H.........   X   lau   David's myotis bat
   T.Q.....R..SNNL(IP.)Q............   X   lau   Microbat
   T.Q.....R..SKNL(IP.)Q............   X   lau   Big brown bat
   SIR.....R..IGSS(EK.)N..S.Y.Y.G...   X   lau   Hedgehog
   NDLN....R..NENF(.Q.)D....RI......   O   lau   Star-nosed mole
   SIQ.....L..NKKR(.L.)K............   O   afr   Elephant
   NISN.......D.KH(TQ.)N..V...L--...   X   afr   Cape elephant shrew
   GMQ.....I..N.RH(.P.)K..F.........   X   afr   Manatee
   NSNN..K.T..SIES(EV.)K..W.K.......   X   afr   Cape golden mole
   .IQ...K.T..NKEN(KQ.)N....Q.......   X   afr   Tenrec
   GQQ.....T..NGKY(.L.)K..F.........   O   afr   Aardvark
   TI.N....R..NKNL(KQ.)K..H..I......   X   xen   Armadillo
   TMNM....M..NGDS(AF.)D....GIY.....   X   mar   Opossum

NO 46
GN FCGBP
ID FCGBP_HUMAN
MP 75
DE IgGFc-binding protein
CL apes
SQ 58
   LSESPASVSILSQAD(NTS)KKVTVRPGESVMV   O   hum   Human
   ---............(...).............   O   hac   Chimp
   ---............(...).............   O   aga   Gorilla
   ---............(...).............   O   gra   Orangutan
   ---............(...).N...........   O   ape   Gibbon
   ---.....F......(K..)HN...........   X   cat   Rhesus macaque
   ---.....F......(K..)HN...........   X   cat   Crab-eating macaque
   ---.....F......(K..)HN...........   X   cat   Baboon
   ---.....F......(K..)HN...........   X   cat   Green monkey
   ---.....V...H..(...)QE.....R.....   O   sim   Marmoset
   ---.....V...H..(...)QQ.....R...R.   O   sim   Squirrel monkey
   ---.........L..(D..)H....I.EV....   X   pri   Bushbaby
   ---..TL........(G.L)QN...........   X   eua   Treeshrew
   ---.I.T...I.H..(K.L)Q....K..QT.T.   X   gli   Squirrel
   ---.TT..T......(G..)Q....K.......   X   gli   Prairie vole
   ---.TT..T......(D..)Q....K.....T.   X   gli   Chinese hamster
   ---.T...T......(AS.)Q..A.K.......   X   gli   Golden hamster
   ----STC......VN(D..)Q...----.....   X   gli   Mouse
   ---.T....L....N(D..)Q.--GE....---   X   gli   Rat
   ---...L.....H..(G.L).............   X   gli   Naked mole-rat
   ---...L........(D..).T.......T...   X   gli   Guinea pig
   ---...L.....R..(...).............   O   gli   Chinchilla
   ---.T.L.....R..(D.L).............   X   gli   Brush-tailed rat
   ---...L..V.....(KA.)QT...K.....V.   X   gli   Rabbit
   ---...A..VV..V.(K.L)Q..IA.L.Q.AV.   X   gli   Pika
   ---..T...V..RV.(G..)Q....G..Q....   X   lau   Alpaca
   ---..T...V..RV.(G..)Q....G..Q....   X   lau   Bactrian camel
   ---.LT.......V.(G..)Q.......Q....   X   lau   Dolphin
   ---.LT.......V.(G..)Q.......Q....   X   lau   Killer whale
   ---.LT......R..(G.T)Q.......Q..L.   X   lau   Tibetan antelope
   ---.LT......R..(G.T)Q.......Q..L.   X   lau   Cow
   ---.LT......R..(G.T)Q.......Q..L.   X   lau   Sheep
   ---.LT.........(G.T)Q.......Q..L.   X   lau   Domestic goat
   ---..........V.(S..)QD......Q....   X   lau   Horse
   ---............(S..)QN.........I.   X   lau   White rhinoceros
   ---......V.....(G..)Q....K..R..T.   X   lau   Cat
   ---..T....HCH..(G.V)QN.I.N..Q..T.   X   lau   Dog
   ---.S.....HCH..(G.E)QN...N..Q..L.   X   lau   Ferret
   ---.......HCH..(G.R)QN...N..Q..T.   X   lau   Panda
   ---.......HCH..(G.R)QN...N..Q....   X   lau   Pacific walrus
   ---.......HCH..(G.R)QN...N..Q....   X   lau   Weddell seal
   ---.....A......(G..)QN......Q....   X   lau   Black flying-fox
   ----....A......(G..)RD......Q....   X   lau   Megabat
   ---.....TV.....(G..)QN.P.K..Q....   X   lau   David's myotis bat
   ---.S...T......(G..)QN.P.K..Q....   X   lau   Microbat
   ---.....A......(G..)QN...K..Q....   X   lau   Big brown bat
   ---.A.T..V.....(D..)Q.......L....   X   lau   Hedgehog
   ---..TT......V.(G.L)QN...G..Q.I.I   X   lau   Shrew
   ---............(G..)Q.......Q..LA   X   lau   Star-nosed mole
   ---.........RT.(..L)QN...K..Q..V.   X   afr   Elephant
   ---......V..RV.(EA.)Q....K.....I.   X   afr   Cape elephant shrew
   ---..........R.(...)Q....KT.Q..L.   O   afr   Manatee
   ---........NR..(...)..I..K..Q....   O   afr   Cape golden mole
   ---......V..RT.(.S.)Q.I..K.......   O   afr   Tenrec
   ---...A..V..RT.(.SL)Q....K..Q....   X   afr   Aardvark
   ---......V..R..(G..)QTL..K..Q.AV.   X   xen   Armadillo
   ----------AEGSQ(GQV)TT...GA.PPDRS   X   mar   Opossum
   ---..TT.T....VT(K..)ET...GT.QT.T.   X   mar   Tasmanian devil

NO 47
GN FCGBP
ID FCGBP_HUMAN
MP 2138
DE IgGFc-binding protein
CL simians
SQ 49
   SQAVSYTRSVTLQIY(NHS)LTLSARWPRKLQV   O   hum   Human
   ...............(...).I...........   O   hac   Chimp
   ...............(...)............-   O   aga   Gorilla
   ...............(...).........Q...   O   gra   Orangutan
   ...............(...).........Q...   O   ape   Gibbon
   ...............(...).........Q...   O   cat   Crab-eating macaque
   ............H..(...).........Q...   O   sim   Marmoset
   ............H..(...).........Q...   O   sim   Squirrel monkey
   ............H..(..N)..M..........   X   pri   Bushbaby
   ............H..(...)........QR..L   O   eua   Treeshrew
   ............HV.(G..).............   X   gli   Squirrel
   ............N..(G..).............   X   gli   Lesser Egyptian jerboa
   ............N..(GL.).....S...Q...   X   gli   Prairie vole
   ..........S.N..(GL.).....Q...R...   X   gli   Chinese hamster
   ...............(GL.).....Q.......   X   gli   Mouse
   ............H..(GL.).....Q...Q...   X   gli   Rat
   ........T...YV.(..V).....L..QR.K.   X   gli   Naked mole-rat
   ............YV.(G.A).....SQ..QI..   X   gli   Guinea pig
   ............HV.(G.A).......K.Q.R.   X   gli   Chinchilla
   .......H....HV.(D.V).....S..QQ...   X   gli   Brush-tailed rat
   ............Y..(D.R).........Q...   X   gli   Rabbit
   ........T...H..(G..)F........Q...   X   lau   Pig
   ........T...H..(G..)F....G...Q...   X   lau   Alpaca
   ........T...H..(G..)F....G..WQ...   X   lau   Bactrian camel
   ...I....T...H..(G..)F.....R..Q...   X   lau   Killer whale
   ........T...H..(G..)F....G...Q...   X   lau   Tibetan antelope
   ........T...H..(G..)F....G...Q...   X   lau   Cow
   ........T...H..(G..)F....G...Q...   X   lau   Sheep
   ........T...H..(G..)F....G...Q...   X   lau   Domestic goat
   ............H..(G.R).....Q.......   X   lau   Horse
   ............HL.(...).....Q...Q...   O   lau   White rhinoceros
   ............HM.(..R).........Q...   X   lau   Cat
   ............H..(S..).....Q...Q...   X   lau   Dog
   ............H..(K..).....Q...Q...   X   lau   Ferret
   ......P.....H..(K..).....Q...Q...   X   lau   Pacific walrus
   ............H..(.R.).........Q..S   O   lau   Black flying-fox
   ............H..(..N).........Q..L   X   lau   David's myotis bat
   ............H..(..N).........Q...   X   lau   Microbat
   ............H..(..R).........Q...   X   lau   Big brown bat
   ..........S.Y.H(..V)....TQ.......   X   lau   Hedgehog
   .........AS.Y..(..N)..V......Q.L.   X   lau   Shrew
   ..........S.T..(..I).V...Q.......   X   lau   Star-nosed mole
   ............H..(...).....Q...Q...   O   afr   Elephant
   ............H..(G..)........KQ...   X   afr   Cape elephant shrew
   .........I..S..(..N).....Q.......   X   afr   Cape golden mole
   ............HM.(K..).....Q...Q...   X   afr   Tenrec
   ..V.........H..(D..).....Q...Q...   X   afr   Aardvark
   ............H..(G..).........Q...   X   xen   Armadillo
   NK..T...D...HV.(D..)FS..SSF....K.   X   mar   Tasmanian devil

NO 48
GN FCGR1A
ID FCGR1_HUMAN
MP 152
DE High affinity immunoglobulin gamma Fc receptor I
CL catarrhines
SQ 54
   YYRNGKAFKFFHWNS(NLT)ILKTNISHNGTYH   O   hum   Human
   ...............(...).............   O   hac   Chimp
   ..Q............(...).............   O   aga   Gorilla
   ...............(...).............   O   ape   Gibbon
   ..Q........YR..(Q..)..........A..   X   cat   Rhesus macaque
   ..Q........YR..(Q..)..........A..   X   cat   Crab-eating macaque
   ..Q........YR..(K..)..........A..   X   cat   Baboon
   ..Q........YR..(...)..........A..   O   cat   Green monkey
   ..Q.....RS..R..(D..)..N.......I..   X   sim   Marmoset
   ..Q...VI.S.NR..(D..).P....N...I..   X   sim   Squirrel monkey
   F.Q...T...LP...(EF.)....SVN...I..   X   pri   Bushbaby
   F.....T...SPHS.(KF.).P...V....I.R   X   eua   Treeshrew
   F.Q.....Q.--R..(EF.).Q...T....V..   X   gli   Squirrel
   FH....P.Q.--QDP(E..)..R..V..S.V..   X   gli   Lesser Egyptian jerboa
   F.Q...S.N.--RG.(EVK).....L....V..   X   gli   Prairie vole
   F..D..--H.YSQD.(EVI)..QA.P..T.I..   X   gli   Chinese hamster
   F..D..--Y.HSQD.(EV.)..Q..LT.S.V..   X   gli   Golden hamster
   F.....S.Q.S--D.(EVA).....L..S.I..   X   gli   Mouse
   F....ESLD.--QG.(EV.).....L..S.I..   X   gli   Rat
   F...E.FI..SR...(E..).....TN.S.L..   X   gli   Naked mole-rat
   F.Q...PV..SYQD.(A..)...A.MN.S.V..   X   gli   Guinea pig
   F.Q...PI..SYRD.(E..)L....MN.SSI..   X   gli   Chinchilla
   F.Q...PIR.TYQ..(A..)....SMN.S.V..   X   gli   Brush-tailed rat
   F.Q.......SSQD.(E..).P...VN...I..   X   gli   Rabbit
   F.Q.DRT...LTQP.(E..).PNV.M......Y   X   gli   Pika
   F.Q...T...SPR..(EF.).....L....I..   X   lau   Pig
   F.QD......SPRD.(EF.).....L..S.I..   X   lau   Alpaca
   F..D......SPRD.(EF.).....L..S.V..   X   lau   Bactrian camel
   F.KD..S.W.S.RD.(EF.).....L....I..   X   lau   Tibetan antelope
   F.KD..P.R.SSQD.(EF.)..Q..L....I..   X   lau   Cow
   F.KD.TS.W.SNRD.(EF.).....L....I..   X   lau   Sheep
   F.KD..S.W.SNRD.(EF.).....L....I..   X   lau   Domestic goat
   F.Q.......SPQD.(EF.).....L....I..   X   lau   Horse
   F.QD......SP...(EF.).R...V....I..   X   lau   White rhinoceros
   F.Q.D.....SPQ..(EF.)....SL....I..   X   lau   Cat
   F.Q..TVL..SPQ..(EF.)....TLH...I..   X   lau   Dog
   F.Q...V...STG..(EF.).....L....I.R   X   lau   Ferret
   F.Q...VL.Y.IQ..(EF.).....L....I..   X   lau   Panda
   F.Q...V.R.SSQ..(EV.).....L....I..   X   lau   Pacific walrus
   F.Q...V.R.SSQ..(EV.).....L....I..   X   lau   Weddell seal
   F.Q...I.H.SPK..(EY.).....L....I..   X   lau   Black flying-fox
   F.Q...I.H.SPT..(EY.).....L....I..   X   lau   Megabat
   F.H.S.T.Q.SYE.P(EV.).P...L....I..   X   lau   David's myotis bat
   F.H.N.T.Q.SYR..(EF.).....L..S.I..   X   lau   Microbat
   F.H.N.I...SS...(EF.).P...L..S.I..   X   lau   Big brown bat
   F.KD......S.KT.(GF.).P...V..S.L..   X   lau   Star-nosed mole
   FFKDERGL..SSCSC(E.S)T....V....I.R   X   afr   Elephant
   F.Q...S...S.H.P(EF.).V...L.......   X   afr   Cape elephant shrew
   F.Q.....M.SPYK.(E..).Q...L..S.V..   X   afr   Manatee
   F.Q.....N.S.F.P(.IS).S..SLD...I..   O   afr   Cape golden mole
   F.Q.D......PH..(Q..)....DL.......   X   afr   Aardvark
   F.L...G.N.SYSD.(EY.).P.IDL....N..   X   xen   Armadillo
   ..H.D..L.YAYECF(DYI)VPQV.YT.S.S.Y   X   mar   Opossum
   ..H.N..L.YEYESF(.YV)VSQV.YT.S...F   X   mar   Tasmanian devil

NO 49
GN FCGR1A
ID FCGR1_HUMAN
MP 195
DE High affinity immunoglobulin gamma Fc receptor I
CL catarrhines
SQ 53
   GISVTVKELFPAPVL(NAS)VTSPLLEGNLVTL   O   hum   Human
   ...............(...).............   O   hac   Chimp
   ...............(...).............   O   aga   Gorilla
   .V.............(...).............   O   cat   Rhesus macaque
   .V.............(...).............   O   cat   Crab-eating macaque
   .V.............(...).............   O   cat   Baboon
   .V.............(...)...........I.   O   cat   Green monkey
   .V.............(S..).............   X   sim   Marmoset
   .V.............(K..).............   X   sim   Squirrel monkey
   .V........TP...(T..)LV......TQ.N.   X   pri   Bushbaby
   .V........TV...(T..)SP..F...SP.N.   X   eua   Treeshrew
   .VT.......SV...(R..)LSL.V........   X   gli   Squirrel
   .V....R...A....(K..)LSL.AP..S....   X   gli   Lesser Egyptian jerboa
   .MA.......A....(R..)LS..F...SP...   X   gli   Prairie vole
   .M........A....(T..)LS..FP..SP...   X   gli   Chinese hamster
   .M........AT...(T..)LS..FP..SP...   X   gli   Golden hamster
   .V.I......TT...(R..).S..FP..S....   X   gli   Mouse
   .V........AT...(RV.)LS..FP..S..I.   X   gli   Rat
   RTA...E........(SS.)LS.H.R.....N.   X   gli   Naked mole-rat
   RVP.M..K.......(TS.)LG...QQRK..K.   X   gli   Guinea pig
   .VA............(SS.)LS...W.EK....   X   gli   Chinchilla
   .VA............(RSP)LS...WKKQ..K.   X   gli   Brush-tailed rat
   .V....Q...RV...(R..)SPL..Q..SA...   X   gli   Rabbit
   TM.........V...(R..)LPF.I...SPL..   X   gli   Pika
   .V.I.I.........(R..)LSF.I......N.   X   lau   Pig
   .V.I......TE...(R..)LPL......M.N.   X   lau   Alpaca
   .V.I......TE...(R..)LPF......T.N.   X   lau   Bactrian camel
   .V.I.I.........(RT.)FS..HQ.....N.   X   lau   Tibetan antelope
   .V.I.I.........(RT.)FS..HQ.....N.   X   lau   Cow
   .V.I.I.........(RT.)FS..HQ.....N.   X   lau   Sheep
   .V.I.I.........(RT.)FS..HQ.....N.   X   lau   Domestic goat
   .V.I.I.........(RS.)LSF........N.   X   lau   Horse
   .VPI...........(KV.)SSL.I......N.   X   lau   White rhinoceros
   .V.I......L....(K..)LSL.....H..N.   X   lau   Cat
   .V.I.I.........(K..)LS..I...HV.N.   X   lau   Dog
   .VNI...........(K..)LS..I...H..N.   X   lau   Ferret
   .VPI...........(K..)LS..I...Q..N.   X   lau   Panda
   .V.I...........(K..)LS..I...H..N.   X   lau   Pacific walrus
   .V.I.I.........(K..)LS..I...H..N.   X   lau   Weddell seal
   .V.I...........(R..)FS--I...SP.N.   X   lau   Black flying-fox
   .V.I...........(ST.)FS--I...SP.N.   X   lau   Megabat
   .V.I...........(R..)PP.S....DP.K.   X   lau   David's myotis bat
   .V....E........(R..)SS..FP..DP.N.   X   lau   Microbat
   .V.I...........(R..)SS.......P.S.   X   lau   Big brown bat
   .V....R....T.L.(KT.)FP...A..SS.H.   X   lau   Star-nosed mole
   .V.I...D...P...(T.T)LS--.....P.N.   X   afr   Elephant
   .VDI..Q........(R..)LSG..PA.HP.N.   X   afr   Cape elephant shrew
   .V.IS..........(R.F)FS.......P.N.   X   afr   Manatee
   .VHII..........(K..)LSF......P.N.   X   afr   Cape golden mole
   .V.IA..........(R..)LSF.....KP.N.   X   afr   Aardvark
   RV.I.......P.E.(R..)LPL.....SS.K.   X   xen   Armadillo
   SVMI..Q....P.TI(I.T)PSTKPQ..SQM..   X   mar   Opossum
   VVIIN.H....P.TM(T.T)TSTQPQ..SQM..   X   mar   Tasmanian devil

NO 50
GN GP1BA
ID GP1BA_HUMAN
MP 37
DE Platelet glycoprotein Ib alpha chain
CL catarrhines
SQ 59
   VSKVASHLEVNCDKR(NLT)ALPPDLPKDTTIL   O   hum   Human
   ...............(...).............   O   hac   Chimp
   ...............(...)V............   O   aga   Gorilla
   ...............(...).............   O   gra   Orangutan
   ...............(...).............   O   ape   Gibbon
   ..............S(..R).............   X   cat   Rhesus macaque
   ..............S(..R).............   X   cat   Crab-eating macaque
   ..............S(...).............   O   cat   Baboon
   ...............(..I).........M...   X   cat   Green monkey
   ...............(..R)V........A...   X   sim   Marmoset
   .......I.......(..R)V......R.A...   X   sim   Squirrel monkey
   .TQ...Q.......Q(Q..)......Q.E.A..   X   pri   Bushbaby
   IF.G.NKV....E.Q(K..)K..LG..S.ANTI   X   eua   Treeshrew
   I..P..Q.....E.Q(...)M..A...A..A..   O   gli   Squirrel
   ....T.Q......N.(K..).V.A...A..G..   X   gli   Lesser Egyptian jerboa
   I.Q.T.L.....ENQ(K..)...A...A..G..   X   gli   Prairie vole
   I.Q.T.FR....ENQ(K..)...A...A..G..   X   gli   Chinese hamster
   I.Q.T.LR....ENQ(K..)...A...A..G..   X   gli   Golden hamster
   I...T.L.....ENK(K..)...A...A..G..   X   gli   Mouse
   ....T.L.....EDK(K.K)K..T...A..G..   X   gli   Rat
   .GNLV.---...ESM(..K)G..T..RA..A..   X   gli   Naked mole-rat
   ..RAGKRVI...ENM(..N)M..A...A..AT.   X   gli   Guinea pig
   ..N.G.RVIA..ENM(..K)G..A..RA..AV.   X   gli   Chinchilla
   ..N.DKR.IM..EDM(..K)G..AG.RT..A..   X   gli   Brush-tailed rat
   ..E...QV.....Q.(K.K)....G..ANM...   X   gli   Rabbit
   ......QV.....RL(R.K)....G..A.M.S.   X   gli   Pika
   .T....QV.M..ENK(T.K).P....EAE..N.   X   lau   Pig
   ..I..KRM.L...NL(G.K).P.....AE.A..   X   lau   Alpaca
   ..I..KRM.L...NL(G.K).P.....AE.A..   X   lau   Bactrian camel
   I.....EV.M..ENK(G.K).P....QAE.N..   X   lau   Dolphin
   I.....EV.M..ENK(G.K).P....QAE.N..   X   lau   Killer whale
   .GSKD.QV....ENK(G.K).P..N.QA..A..   X   lau   Tibetan antelope
   .GNKD.QV....ENK(G.K).P.....A..A..   X   lau   Cow
   .GNKD.QV....ENK(G.K).P..N.QA..A..   X   lau   Sheep
   .GNKD.QV....ENK(G.K).P..N.QA..A..   X   lau   Domestic goat
   ..E..--V.......(G.K)K.....RN..D..   X   lau   White rhinoceros
   ..Q.T.QV.....N.(G.K).P.....A..A..   X   lau   Cat
   ..Q.TTLM.....N.(G.K)V..T...G..A..   X   lau   Ferret
   ..Q.T.QV.....N.(G.R).V..G..E..A..   X   lau   Panda
   ....TRL.....EN.(GQK)V......R..A..   X   lau   Pacific walrus
   ....T.Q......N.(G.K)V......R.MA..   X   lau   Weddell seal
   .T....QV.....NL(G.K).......A..N..   X   lau   Black flying-fox
   ..R...QV.....NQ(G.K).M.S...A...S.   X   lau   David's myotis bat
   ..R...QV.....NQ(G.K).M.....A.....   X   lau   Microbat
   ..T...QV.....N.(G.K).M.S...PG....   X   lau   Big brown bat
   F...INK.H...ENL(G.K)...T.RLT.S...   X   lau   Hedgehog
   ..E..NQV.T..E..(G.K).V.SG.AA..A..   X   lau   Shrew
   ..R...QV....EN.(G.K)V..R...A.....   X   lau   Star-nosed mole
   ..E.D.KAL...Q..(G.K)V......T..A..   X   afr   Elephant
   I.....KV.A..ETQ(G.K)T......A..A..   X   afr   Cape elephant shrew
   ....D.KV.M..E.H(G.K)VP.L...V..A..   X   afr   Manatee
   ...ETNQV....ENL(G.K).....M.A..V..   X   afr   Cape golden mole
   G.RE..QMD...EAL(G.K)V..S...A..S..   X   afr   Tenrec
   I.RETTEV....EN.(R.K)G......P..V..   X   afr   Aardvark
   FY...NQV....ENL(Q.K)...A...A.....   X   xen   Armadillo
   IH....K..T..ESL(G.K).V.SK.SP..A..   X   mar   Opossum
   TQ.G..KM.T..ESL(G.K)SV.LK.SP..A..   X   mar   Tasmanian devil
   TH..D.K..T..ESL(G.K)SV.SK..P..N..   X   mar   Wallaby
   IHH--.D..TT.RGL(G.R).V.TN..AA.G..   X   mon   Platypus

NO 51
GN GP1BA
ID GP1BA_HUMAN
MP 175
DE Platelet glycoprotein Ib alpha chain
CL African great apes
SQ 61
   LTPTPKLEKLSLANN(NLT)ELPAGLLNGLENL   O   hum   Human
   ...............(...).............   O   hac   Chimp
   ...............(...).............   O   aga   Gorilla
   ...............(D..)..........G..   X   gra   Orangutan
   ...............(...)..........G..   O   ape   Gibbon
   ...............(H..)Q......D..G..   X   cat   Rhesus macaque
   ...............(H..)Q......D..G..   X   cat   Crab-eating macaque
   ...............(H..)Q......D..G..   X   cat   Baboon
   ...............(H..).......D..G..   X   cat   Green monkey
   .M.............(...)...PR........   O   sim   Marmoset
   .M.............(...)...P.........   O   sim   Squirrel monkey
   .....E.K..N..E.(RM.)D..S...A..SS.   X   pri   Bushbaby
   .A..TR.K.....D.(K..)...P...D..DK.   X   eua   Treeshrew
   .ET.TQ.Q..N....(K.S)...P.......E.   X   gli   Squirrel
   .AS.T..R..N..K.(..G)...P...D..GE.   X   gli   Lesser Egyptian jerboa
   .VS.TN.K..N....(H.R)...H...D...D.   X   gli   Prairie vole
   .VSVTD.K..N..Y.(K.R)...P..IV..T..   X   gli   Chinese hamster
   .VSVTN.K..N....(K.R)...P...D...M.   X   gli   Golden hamster
   .L..T..K..N....(K.R)...S...D...D.   X   gli   Mouse
   .M..T..L.......(K.R)...P...D...D.   X   gli   Rat
   .AS.SQ.K..N....(Q.N)...P.......E.   X   gli   Naked mole-rat
   .AS.SQ.K..N....(G.A).V.P....E..D.   X   gli   Guinea pig
   .A..SG.K..N....(G.A).VSP....E..E.   X   gli   Chinchilla
   .AS.SQ.K..N....(R..)NV.P...SNM.D.   X   gli   Brush-tailed rat
   .A.MRQ.R..N..E.(Q.H)...PQ..A..GY.   X   gli   Rabbit
   .A..TQ.R..N..E.(K.N)D..S...D..TE.   X   gli   Pika
   .A.....K..N..E.(Q.K)...P...D...EP   X   lau   Pig
   .AT.SQ.Q..N..D.(R.S)...P.F.H..DE.   X   lau   Alpaca
   .AT.SQ.Q..N..D.(R.S)...P.F.H..DE.   X   lau   Bactrian camel
   .Q.....K..N..E.(G.S)...P.F.D...E.   X   lau   Dolphin
   .Q.....K..N..E.(G.S)...P.F.D...E.   X   lau   Killer whale
   .V...R.R..N..E.(D.Q)...TE..K...E.   X   lau   Tibetan antelope
   .V...H.K.....E.(D.Q)...PE..K...E.   X   lau   Cow
   .V...R.R..N..E.(D.Q)...TE..K...E.   X   lau   Sheep
   .V...R.R..N..E.(D.Q)...SE..K...E.   X   lau   Domestic goat
   .R..AQ.K..N..E.(K.Q)...P...D...D.   X   lau   Horse
   .G..AQ.KQ.N..D.(E.Q)...PE......E.   X   lau   White rhinoceros
   .A...R.R..N..D.(K.N)...P...E...E.   X   lau   Cat
   .Q.V.Q.......D.(K.N)...L...D...K.   X   lau   Ferret
   .A.V.Q.R..N..D.(K.S)...P...D..GE.   X   lau   Panda
   .R.VAQ.R..N..D.(K.N)...P...D..GE.   X   lau   Pacific walrus
   .R.VAQ.R..N..D.(K.N)...P...D..AD.   X   lau   Weddell seal
   .A..TR.R..N..D.(K.E)D..L.F.D...E.   X   lau   Black flying-fox
   .A..TR.R..N..D.(K.E)D..L.F.D...E.   X   lau   Megabat
   .AS..Q.K..N..Q.(Q.M)...LE..E...E.   X   lau   David's myotis bat
   .A...Q.K..N..E.(R..)...LE..D...E.   X   lau   Microbat
   .AS.VQ.R..N..D.(E.Q)...LE..D...E.   X   lau   Big brown bat
   .AL..Q.K.....D.(H.E)...P...D..D..   X   lau   Hedgehog
   .V..AN.K..N..D.(K.G)...P...D..GQ.   X   lau   Shrew
   .A...Q.R..N..S.(K.K)...P...D..DE.   X   lau   Star-nosed mole
   .AS..RVK..N....(E.D)...P......DD.   X   afr   Elephant
   .A..R..K..N.SE.(..E)...P...D...D.   X   afr   Cape elephant shrew
   .A...R.K..N..Y.(K.S)...Q...D...D.   X   afr   Manatee
   .A.....Q..N....(K.A)K..P...D..DD.   X   afr   Cape golden mole
   .V.....Q..N....(Q.R)...P...D..TE.   X   afr   Tenrec
   .QA....Q..N....(K.E)...P...H...D.   X   afr   Aardvark
   .A...Q.K..N..D.(E.N)...P...D.VDE.   X   xen   Armadillo
   .QA.R.....D....(Y.E)H.S.D..AN.S..   X   mar   Opossum
   .KAAF..Q..D..K.(R.E)H.SVD..AD.G..   X   mar   Tasmanian devil
   .KA....Q..N....(Y.E)H.S.D..K.....   X   mar   Wallaby
   .R.....KT.D..H.(Q.E)A..GL..ES.RD.   X   mon   Platypus

NO 52
GN GUSB
ID BGLR_HUMAN
MP 272
DE Beta-glucuronidase
CL catarrhines
SQ 57
   KLEVRLLDAENKVVA(NGT)GTQGQLKVPGVSL   O   hum   Human
   ...............(...).............   O   hac   Chimp
   E..A...........(...)..........A..   O   gra   Orangutan
   E..............(..S)V.........A..   O   ape   Gibbon
   E...........L..(...)..........AR.   O   cat   Crab-eating macaque
   E...........L..(...)..........AR.   O   cat   Baboon
   E.........H.L..(...)..........AR.   O   cat   Green monkey
   E..............(D..)..........AN.   X   sim   Squirrel monkey
   Q...F.W.E.GRI..(K..).SR...Q..SAH.   X   pri   Bushbaby
   Q.......E.G....(E..).GR...Q...AH.   X   eua   Treeshrew
   Q.D.H....DGR...(D.A).GR...Q..AAH.   X   gli   Squirrel
   H...C...EDG....(K..).N....Q..NAQ.   X   gli   Lesser Egyptian jerboa
   Q.......EDG....(QE.).E....R..SAN.   X   gli   Prairie vole
   Q.......EDG....(QE.).D....R...AN.   X   gli   Chinese hamster
   Q.......EDGQ...(QE.).D....Q...AN.   X   gli   Golden hamster
   Q...Q...EGG....(H..).N....Q..SAN.   X   gli   Mouse
   Q.......EDG.I..(R..).NE......RAH.   X   gli   Rat
   ..Q.Q...V.GHI..(Q..).G....Q..TAR.   X   gli   Naked mole-rat
   E.Q.....M.GN...(Q..).DE...Q..NAH.   X   gli   Guinea pig
   E.........GN...(Q..).GR...Q..NAR.   X   gli   Chinchilla
   E.Q.W.....GS...(R..).GE...Q..NAH.   X   gli   Brush-tailed rat
   Q...S...E.G....(Q..).GW...L..SAN.   X   gli   Rabbit
   L.......K.S....(Q..).DR...Q..SAN.   X   gli   Pika
   Q...H...E.GR...(K..).G....Q..SAH.   X   lau   Pig
   Q...F...EDGRA..(Q..).G....Q..RAH.   X   lau   Alpaca
   Q...F....DGRT..(Q..).G....Q..RAH.   X   lau   Bactrian camel
   Q...C...E.GT...(K..).GR...Q..SAH.   X   lau   Dolphin
   Q...C...E.GT...(K..).GR...Q..SAH.   X   lau   Killer whale
   QVD.S...EDG....(K.A).AE...Q..SAH.   X   lau   Tibetan antelope
   QVD.S...E.G..M.(K.A).AE...Q..SAH.   X   lau   Cow
   Q.D.C...E.G..L.(K.A).V....Q..SAH.   X   lau   Domestic goat
   Q.......E.G....(Q..).D....Q..SAY.   X   lau   Horse
   Q.......E.G....(Q..).GR...Q...AH.   X   lau   White rhinoceros
   Q.......E.G....(Q..).GR...Q..NAH.   X   lau   Cat
   Q...Y...E.G....(Q..).S..R.Q..N.H.   X   lau   Dog
   Q.......Q.S....(Q..).CR...Q..NAH.   X   lau   Ferret
   Q...Y...Q.G....(Q..).GR...Q..NAH.   X   lau   Panda
   Q...C...Q.G....(Q..).GR...Q..NAH.   X   lau   Pacific walrus
   Q.......Q.G....(Q..).GR...Q..NAH.   X   lau   Weddell seal
   Q...Y...E.G....(Q.M).G....Q..SAH.   X   lau   Black flying-fox
   Q...Y...E.G....(Q.M).G....Q..SAH.   X   lau   Megabat
   Q...Y.H.E.GNI..(R..).SR...Q..NAH.   X   lau   David's myotis bat
   Q...Y.Q.E.GNI..(R..).AR...Q..NAH.   X   lau   Microbat
   Q...Y.Q.E.GNI..(R..).GH...Q..NAH.   X   lau   Big brown bat
   E...F...EDS....(K..).GR...R...AH.   X   lau   Hedgehog
   Q.......EDG....(Q.E).GR...R..AAH.   X   lau   Shrew
   Q...C...E.G....(Q..).GR...Q..SAH.   X   lau   Star-nosed mole
   E.........GQI..(HSM).A....Q..SAH.   X   afr   Elephant
   Q.....Q.E.GQ...(Q.S).AR...Q..SAH.   X   afr   Cape elephant shrew
   E.A.......GQ...(HH.).A....Q..SAH.   X   afr   Manatee
   E..T......GQ...(H.E).A....R..NAH.   X   afr   Cape golden mole
   Q.QT......GQ...(Q.P).A....Q..SAH.   X   afr   Tenrec
   D.K.......GQQ..(E..).A....Q..RAH.   X   afr   Aardvark
   E.K.....EDGG...(Q..).A....Q..SAH.   X   xen   Armadillo
   E.D.L.W.K.GRA..(K..).AR...Q..DAQ.   X   mar   Opossum
   ------------...(K.A).........NAQ.   X   mar   Tasmanian devil
   E..LL.W.K.GR...(K..).VR...A..NAQF   X   mar   Wallaby

NO 53
GN HEG1
ID HEG1_HUMAN
MP 520
DE Protein HEG homolog 1
CL simians
SQ 58
   RSYSESSSTSSSESL(NSS)APRGERSIAGISY   O   hum   Human
   ...............(...)........T....   O   hac   Chimp
   ..........F....(...)........T....   O   gra   Orangutan
   ...............(...)........T....   O   ape   Gibbon
   .N.............(...)........T....   O   cat   Rhesus macaque
   .N.............(...)........T....   O   cat   Crab-eating macaque
   .N.............(...)........T....   O   cat   Baboon
   .N.............(...)........T....   O   cat   Green monkey
   ...............(...)P..E....T....   O   sim   Marmoset
   ...............(...)P..E....T....   O   sim   Squirrel monkey
   SG....L........(D..)........T....   X   pri   Bushbaby
   ......L........(D..)........T....   X   eua   Treeshrew
   S......FI......(D..)TLH..N..T.L..   X   gli   Squirrel
   .GF....A.......(D..)V.L.....T.L..   X   gli   Lesser Egyptian jerboa
   KNF.....SA.....(D..).T...H..TELNQ   X   gli   Prairie vole
   K..............(D..)..H.GH..T.L.D   X   gli   Chinese hamster
   K.....A...A....(D..)..QRGH..T.L..   X   gli   Golden hamster
   ...A...........(D..)..LR.H.LT.L..   X   gli   Mouse
   ...A...........(D.P)..GRAH..T.L..   X   gli   Rat
   ......-----.QR.(E..).Q...H..T.F..   X   gli   Naked mole-rat
   K.....L.P.F.Q..(E.L).Q...H..T.L--   X   gli   Guinea pig
   N..L..L.....QR.(E.V).Q..KH..T.L--   X   gli   Chinchilla
   N.....L....LQ..(E..).QH..H..T.L--   X   gli   Brush-tailed rat
   T.F...P........(...)........T.F..   O   gli   Rabbit
   KRF........T...(D..)........T.L..   X   gli   Pika
   G.--....S...T..(D.W)..H..HL.T.T.S   X   lau   Pig
   ..S...L.S......(D.W)..H...W.T..G.   X   lau   Alpaca
   ..S...L.S......(D.W)......W.T..G.   X   lau   Bactrian camel
   ......L.S......(G..)........T.M..   X   lau   Dolphin
   ......L.S......(G..)........T.M..   X   lau   Killer whale
   M.....L.S......(GF.)........P....   X   lau   Tibetan antelope
   M....AL.S......(GF.).......MT....   X   lau   Cow
   M.....L.S......(GF.)........P.V..   X   lau   Sheep
   M.....L.S......(GF.)........P....   X   lau   Domestic goat
   .G...TL.SPA.KNQ(...)S.......T..GW   O   lau   Horse
   ......L.SP....P(...)........T.D..   O   lau   White rhinoceros
   ......L.S......(D..).A...C..T....   X   lau   Cat
   ..HAG.L.S......(AA.)...D....T.V.D   X   lau   Dog
   ...A..L.S......(D..)S.......T....   X   lau   Ferret
   ...A..L.S......(D..)........T.T..   X   lau   Panda
   ..SA..L.SP.....(D..)........T....   X   lau   Pacific walrus
   ..SA..L.S......(D..)........T.M..   X   lau   Weddell seal
   ......L.S......(E..)..L.....T.T..   X   lau   Black flying-fox
   ......L.S......(E..)..L..H..T.T..   X   lau   Megabat
   G.DA.P..S......(E..)........T..I.   X   lau   David's myotis bat
   G.DA....S......(E..)........T.VI.   X   lau   Microbat
   G.DA....S......(E..)........T..I.   X   lau   Big brown bat
   H.H...L.-......(D..)..H.KHL.T.M.K   X   lau   Hedgehog
   SRP.K.W.V......(EA.).LQR..AVT.V.L   X   lau   Shrew
   ..S..FL.S......(DF.)......F.T.V.R   X   lau   Star-nosed mole
   .N....L.V......(DP.).S...H..T.VG.   X   afr   Elephant
   .PDLGALF.......(DPL).S......T.V..   X   afr   Cape elephant shrew
   .NS...L........(DP.)VS......T....   X   afr   Manatee
   .N....L......N.(DF.).S......T....   X   afr   Cape golden mole
   .N..D.L..F..R..(D..).S......T....   X   afr   Aardvark
   ....K.L........(D..).S.D....T.F..   X   xen   Armadillo
   THL.....A....MS(D.L)--HHHPLPT.SNH   X   mar   Tasmanian devil
   AHLPG...A....IW(D..)--Q.RPLST..NH   X   mar   Wallaby

NO 54
GN HLA-DMB
ID DMB_HUMAN
MP 110
DE HLA class II histocompatibility antigen, DM beta chain
CL apes
SQ 58
   QNCATHTQPFWGSLT(NRT)RPPSVQVAKTTPF   O   hum   Human
   ...............(...).............   O   hac   Chimp
   ...............(...).............   O   aga   Gorilla
   ...............(...).............   O   ape   Gibbon
   ...T...........(H..)........Q....   X   cat   Rhesus macaque
   ...T...........(H..)........Q....   X   cat   Crab-eating macaque
   ...T...........(H..)........Q....   X   cat   Baboon
   ...T...........(H..)........Q....   X   cat   Green monkey
   .D.............(H..)........R....   X   sim   Marmoset
   .D.............(...).Q......R....   O   sim   Squirrel monkey
   .D..V..........(H..).A......Q....   X   pri   Bushbaby
   .D.S........P..(H..)...L....Q....   X   eua   Treeshrew
   .D.........K...(...)........QA...   O   gli   Squirrel
   .D...R......T..(H..)........Q....   X   gli   Lesser Egyptian jerboa
   .D..M..K...NA..(H..)........Q.A.V   X   gli   Prairie vole
   .D..M......DA..(H..)........R.A.I   X   gli   Chinese hamster
   RD.........DA..(Q..)........Q.A.I   X   gli   Golden hamster
   LD..S......NA..(H..).A...R..Q....   X   gli   Mouse
   .D.S.......NA..(H..).T......Q.A..   X   gli   Rat
   .D.........E...(H.R)K.......Q....   X   gli   Naked mole-rat
   .D.............(Q.K)K..T....Q....   X   gli   Guinea pig
   .D.............(H..)K.......Q....   X   gli   Chinchilla
   .D.V...K.......(H..)K.......Q....   X   gli   Brush-tailed rat
   K..G.......T...(H..)...T....E....   X   gli   Rabbit
   .......N...DK..(R.A)...T....PV...   X   gli   Pika
   ...........K...(H..)Q............   X   lau   Pig
   .D.........K...(H..).............   X   lau   Alpaca
   .D.........K...(H..).............   X   lau   Bactrian camel
   .D.........R...(H..).........A...   X   lau   Dolphin
   .D.........R...(H..).......V.A...   X   lau   Killer whale
   .D.VV......S...(H..)...T....R....   X   lau   Tibetan antelope
   ....A......S...(H..)...T.........   X   lau   Cow
   .D..V......S...(H..)...T....R....   X   lau   Sheep
   .D..V......S...(H..)...T....R....   X   lau   Domestic goat
   .D..N..........(...)WA...........   O   lau   Horse
   .D...L.........(...).............   O   lau   White rhinoceros
   .D......S......(H..)...T.........   X   lau   Cat
   .D......S......(H..)...T.....S...   X   lau   Dog
   .D......S......(H..)...T.........   X   lau   Ferret
   .D..AR..S......(H..)...T.........   X   lau   Panda
   .D......S......(H..)...T.........   X   lau   Pacific walrus
   .D......S......(H..)...T.........   X   lau   Weddell seal
   .HXX...K.......(R..).............   X   lau   Black flying-fox
   .D...R.....E...(Q..).........A..V   X   lau   David's myotis bat
   .D...R.....E...(Q..).........A..V   X   lau   Microbat
   .D.............(Q..).........A...   X   lau   Big brown bat
   .D.S....A......(H..)...I..I.....I   X   lau   Hedgehog
   .D.SS.......A..(K.I)Q.......E....   X   lau   Shrew
   KD..I...D..E...(H.I)......I......   X   lau   Star-nosed mole
   .D.......Y.K...(H..).A...K..PV...   X   afr   Cape elephant shrew
   .D..M..........(H..).L...K..PV...   X   afr   Manatee
   .D.........RT..(Q..).....K.GPV...   X   afr   Cape golden mole
   .D.............(Q..).....K..PIA..   X   afr   Tenrec
   .D..........P..(Q..)K....K..PV...   X   afr   Aardvark
   .D.............(S.A)........R....   X   xen   Armadillo
   .D..S..K....A..(Q..).L....I.QVK..   X   mar   Opossum
   .D..S..K.......(Q..).S....I.Q....   X   mar   Tasmanian devil
   ....S..K.......(H..)........QV...   X   mar   Wallaby

NO 55
GN HPSE
ID HPSE_HUMAN
MP 238
DE Heparanase
CL simians
SQ 60
   GNEPNSFLKKADIFI(NGS)QLGEDFIQLHKLL   O   hum   Human
   ...............(...).............   O   hac   Chimp
   ...............(...).............   O   aga   Gorilla
   ...............(...).............   O   gra   Orangutan
   ...............(...).............   O   ape   Gibbon
   ...............(...).............   O   cat   Rhesus macaque
   ...............(...).............   O   cat   Crab-eating macaque
   ...............(...).............   O   cat   Baboon
   ...............(...).............   O   cat   Green monkey
   ...............(...).............   O   sim   Marmoset
   ...............(...).............   O   sim   Squirrel monkey
   .......R...G...(D.L).......E..N..   X   pri   Bushbaby
   .......RI.SG...(D.L)...R..VR.R...   X   eua   Treeshrew
   .......W..SG.Y.(D.F).......E.....   X   gli   Squirrel
   .......R...G.S.(D.L)......VE.....   X   gli   Lesser Egyptian jerboa
   .......W...HV..(D.L)......VE.S...   X   gli   Prairie vole
   .......W...H...(D.L)......V.FR...   X   gli   Chinese hamster
   .......W..GH...(D.L)......VEFR...   X   gli   Golden hamster
   .......W...H.L.(D.L)......VE.....   X   gli   Mouse
   .......W...Q.S.(D.L)..-----------   X   gli   Rat
   .......R...G...(D.L)...K...E.....   X   gli   Naked mole-rat
   .....N.R...G...(D.W)...K..VA.....   X   gli   Guinea pig
   .......R...G...(D.W)...K...E.....   X   gli   Chinchilla
   ......YE...G...(D.W)...K..VK.....   X   gli   Brush-tailed rat
   .......W...G...(D.L)...K...E.R...   X   gli   Rabbit
   .......R...G...(D.L).......E.....   X   gli   Pika
   .......RM..G.Y.(D.F).......D.....   X   lau   Pig
   .......R...G...(D.L).......E.....   X   lau   Alpaca
   .......R...G...(D.L).......E.....   X   lau   Bactrian camel
   .......R...G...(D.L)......ME.....   X   lau   Dolphin
   .......R...G...(D.L)......ME.....   X   lau   Killer whale
   .......QR..G...(..R).......EFR...   X   lau   Tibetan antelope
   .......QR..G...(..R).......EFR...   X   lau   Cow
   .......QR..G...(S.W).......E.R...   X   lau   Sheep
   .......QR..G...(..R).......E.....   X   lau   Domestic goat
   .......R...G...(D.L).......E.R...   X   lau   Horse
   .......W...G...(D.L).......E.....   X   lau   White rhinoceros
   .......R...G...(D.L)......VK.....   X   lau   Cat
   .......R...G...(D.L)...K..VK.....   X   lau   Dog
   .......R...G...(D.L)......VK.....   X   lau   Ferret
   .......W...G...(D.L)......VK.....   X   lau   Panda
   .......R...G...(D.L)......VK.....   X   lau   Pacific walrus
   .......R...G...(D.L)......VK.....   X   lau   Weddell seal
   .......Q...G...(D.L).......E.....   X   lau   Black flying-fox
   .......Q...G...(D.L).......E.....   X   lau   Megabat
   .......Q...G...(D.L)......VK.....   X   lau   David's myotis bat
   .......Q...G...(D.L)......VK.....   X   lau   Microbat
   .......Q...G...(D.L)......VK.....   X   lau   Big brown bat
   .......R...G...(D.L)...K.........   X   lau   Hedgehog
   .......R...G...(D.L).......E.....   X   lau   Star-nosed mole
   ..........SG.Y.(D.F).......E.....   X   afr   Elephant
   ...........G.YV(D..)......VE.....   X   afr   Cape elephant shrew
   ..........SG.Y.(D.F).......E.....   X   afr   Manatee
   .......R..SG.Y.(D.F)......VE..T..   X   afr   Cape golden mole
   ..........SGVY.(D.F)...T...E.R...   X   afr   Tenrec
   ...........GVY.(D.F).......E.....   X   afr   Aardvark
   .......R...G.Y.(D.F).......E.....   X   xen   Armadillo
   .......R..SG.Y.(E..)...K...K..S..   X   mar   Opossum
   .......R..SG.Y.(...)...K...K..S..   O   mar   Tasmanian devil
   .......R..SG.Y.(...)...K...KF.S..   O   mar   Wallaby

NO 56
GN HPX
ID HEMO_HUMAN
MP 453
DE Hemopexin
CL apes
SQ 62
   DVEKLNAAKALPQPQ(NVT)SLLGCTH-   O   hum   Human
   ...............(...).......*   O   hac   Chimp
   .........T.....(...).......*   O   aga   Gorilla
   ...............(...).......*   O   gra   Orangutan
   ...............(.M.).......*   O   ape   Gibbon
   .........SP....(K..)......Y*   X   cat   Rhesus macaque
   .........SP....(K..)......Y*   X   cat   Crab-eating macaque
   .........SP....(K..).......*   X   cat   Baboon
   .........SP....(K..).......*   X   cat   Green monkey
   .........T.....(K.A).......*   X   sim   Marmoset
   .........TR....(K.A).......*   X   sim   Squirrel monkey
   .........N.....(K..).......*   X   pri   Bushbaby
   ..D............(R.A)D....S.*   X   eua   Treeshrew
   .....T.........(R.N).....S.*   X   gli   Squirrel
   N....I...T.....(R.N).....S.*   X   gli   Lesser Egyptian jerboa
   SID......VA....(R.N).....SQ*   X   gli   Prairie vole
   SID......IVS...(K.N).....SQ*   X   gli   Chinese hamster
   SID......TVSK..(K.N).....SQ*   X   gli   Golden hamster
   SID......S.....(K.N).I...SQ*   X   gli   Mouse
   SID......S.....(K.N).I...SQ*   X   gli   Rat
   .....S...T.....(R.A).....S.*   X   gli   Naked mole-rat
   .........T.....(K.A).....G.*   X   gli   Guinea pig
   ..D..S...S.....(K.A)T....S.*   X   gli   Chinchilla
   .....S...S.....(RTA).....S.*   X   gli   Brush-tailed rat
   ..D......N.....(R.S)R......*   X   gli   Rabbit
   .........S.....(T.L)R.....Q*   X   gli   Pika
   .....VS........(S.N).....HRS   X   lau   Pig
   .....T..ENP....(R.N).....LPS   X   lau   Alpaca
   .....T...NP....(R.N).....LPS   X   lau   Bactrian camel
   ......V........(K.N).....P.*   X   lau   Dolphin
   ......V........(K.N).....P.*   X   lau   Killer whale
   ...E.SKT.D...A.(RMN).....APS   X   lau   Tibetan antelope
   ...E.SKT.D...A.(RMN).....APH   X   lau   Cow
   ...E.SKT.D...A.(RMN).....APS   X   lau   Sheep
   ...E.SKT.D...A.(RMN).....APS   X   lau   Domestic goat
   ............K..(RMN).....HN*   X   lau   Horse
   .L.......T.....(R.N).....N.*   X   lau   White rhinoceros
   ......T..T....L(R.D).....S.*   X   lau   Cat
   .....ST..S.....(R.K).....N.*   X   lau   Dog
   .....ST..T.....(R.E)T....G.*   X   lau   Ferret
   .....ST..T.....(R.E).....N.*   X   lau   Panda
   .A...ST..T.....(K.G).....N.*   X   lau   Pacific walrus
   .A...ST..T.....(K.G).I...N.*   X   lau   Weddell seal
   .....S.......A.(RMN).....S.*   X   lau   Black flying-fox
   .....S.......A.(RMN).....S.*   X   lau   Megabat
   N..E.S........L(RMN).....S.*   X   lau   David's myotis bat
   N..E.S........L(RMN).....S.*   X   lau   Microbat
   N..E.S.S..F...L(RTN).....S.*   X   lau   Big brown bat
   ......S........(R..)D....R.*   X   lau   Hedgehog
   .....T........L(R.A)D....S.*   X   lau   Shrew
   .....S...V...A.(R.G).....R.*   X   lau   Star-nosed mole
   ..D............(K.A)D....S.S   X   afr   Elephant
   ..D......V.....(K.A)AF...N.T   X   afr   Cape elephant shrew
   ..D.........K..(S.A)N....S.S   X   afr   Manatee
   ..D..S...V.....(R.A)D....N.S   X   afr   Cape golden mole
   ..D......T.....(K.I)H.FS.H.I   X   afr   Tenrec
   ..D.....EV.....(T.A).....N.V   X   afr   Aardvark
   ..D..KT..T...A.(R..).....N.G   X   xen   Armadillo
   .P.E.KTT.IP.P..(S.A)R..N.RQ*   X   mar   Opossum
   ...E.KT..TF.P.R(S.A).......*   X   mar   Tasmanian devil
   ...E.RT..NP.S.L(..A).F...RR*   X   mar   Wallaby
   GP.E.TPSGIP.K.H(SLA)T....A.*   X   mon   Platypus

NO 57
GN HRG
ID HRG_HUMAN
MP 63
DE Histidine-rich glycoprotein
CL catarrhines
SQ 59
   FQLLRIADAHLDRVE(NTT)VYYLVLDVQESDC   O   hum   Human
   ...............(...).............   O   hac   Chimp
   ....L..........(...).............   O   aga   Gorilla
   ...............(...)....A...R....   O   gra   Orangutan
   .....V.........(...)....A........   O   ape   Gibbon
   .....V.........(.A.)....A........   O   cat   Rhesus macaque
   .....V.........(.A.)....A........   O   cat   Crab-eating macaque
   .....V.........(.A.)....A........   O   cat   Baboon
   .....V.........(.A.)....A........   O   cat   Green monkey
   .....V.........(TA.)I...A......N.   X   sim   Marmoset
   .....V.........(TA.)I...A......N.   X   sim   Squirrel monkey
   --------------G(SA.)....L..IK..E.   X   pri   Bushbaby
   .E.W.V......KAD(SA.)......E.K....   X   eua   Treeshrew
   .....V......T.G(SA.)........K....   X   gli   Squirrel
   .....V......T.G(SA.)....A...K....   X   gli   Lesser Egyptian jerboa
   .....VS.....T.R(.A.)I..F....K....   O   gli   Prairie vole
   .E...V........G(.A.)........K....   O   gli   Chinese hamster
   .E...VS.......R(...)........K....   O   gli   Golden hamster
   .E...VS......AG(TA.)....A...I....   X   gli   Mouse
   .....VS........(TA.)I.......V....   X   gli   Rat
   .....V........G(PA.).......M.....   X   gli   Naked mole-rat
   .....V......TMG(PA.)....A...R....   X   gli   Guinea pig
   .L...V.......A.(PA.).............   X   gli   Chinchilla
   .H...V.......TG(P.A).............   X   gli   Brush-tailed rat
   .....V......GA.(SA.)........K.T..   X   gli   Rabbit
   .....V......GA.(SA.)........K.T..   X   gli   Pika
   .E..QV......KS.(S.A)......H.K....   X   lau   Pig
   .....V......KA.(SVA)........K....   X   lau   Alpaca
   .....V......KA.(SIA)........K....   X   lau   Bactrian camel
   ....WV..V...KMG(S.A).*......K....   X   lau   Dolphin
   ....WV..V...KMG(S.A).*......K....   X   lau   Killer whale
   .....V....-E...(S.A)........K....   X   lau   Tibetan antelope
   .....V......K..(SIA)........K....   X   lau   Cow
   .....V.....E...(S.A)........K....   X   lau   Sheep
   .....V.....E...(S.A)........K....   X   lau   Domestic goat
   .E.V......V.KA.(SAA)........E....   X   lau   Horse
   ......T.....KT.(S.A)........K..N.   X   lau   White rhinoceros
   .....V......K..(SAA)........K....   X   lau   Cat
   ....QV....V.ES.(SVA)........K....   X   lau   Dog
   .....V....V.QA.(SAA)........K....   X   lau   Ferret
   .....V......K..(SAA)........K....   X   lau   Panda
   ....QV....V.KA.(SVA).H......K....   X   lau   Pacific walrus
   .....V....V.KA.(SVA)........K....   X   lau   Weddell seal
   .....V.....NNL.(FA.)........K....   X   lau   Black flying-fox
   .....V......K..(SRD)........K....   X   lau   David's myotis bat
   .....V......KA.(S.D)........K....   X   lau   Microbat
   .....V......KG.(FAA)........K....   X   lau   Hedgehog
   .....V......KG.(SS.)........I....   X   lau   Shrew
   .....V......KA.(S..)........K....   X   lau   Star-nosed mole
   .....V......EA.(SAA)........K....   X   afr   Elephant
   ...V.V....V.QAD(SVA).......IK.F..   X   afr   Cape elephant shrew
   .....VT.....K..(SAD)........K....   X   afr   Manatee
   .....V......KA.(SA.)........V....   X   afr   Cape golden mole
   .E...V......K..(S.V)....A...S.T..   X   afr   Tenrec
   .....V......KA.(SVA)....A...K....   X   afr   Aardvark
   ......T.....KA.(SPA)........K....   X   xen   Armadillo
   .D...V....THEE.(SAV)IH......M.T..   X   mar   Opossum
   .....V....T.EEK(SAI)IH......I....   X   mar   Tasmanian devil
   .E...V....VHQT.(YAL).S......I....   X   mon   Platypus

NO 58
GN ICAM1
ID ICAM1_HUMAN
MP 267
DE Intercellular adhesion molecule 1
CL African great apes
SQ 59
   LALGDQRLNPTVTYG(NDS)FSAKASVSVTAED   O   hum   Human
   ...............(...).............   O   hac   Chimp
   ...............(...).............   O   aga   Gorilla
   ...............(V..)L...........E   X   gra   Orangutan
   ........D......(V..)L...........E   X   ape   Gibbon
   ......K....I...(.N.)L......K....E   O   cat   Rhesus macaque
   .E....K..T.I...(.N.)L......K....K   O   cat   Crab-eating macaque
   ......K....I...(DN.)L......K....E   X   cat   Baboon
   ......K....I...(.N.)L......K....E   O   cat   Green monkey
   ......K..H.I...(I..)L......KG.T..   X   sim   Squirrel monkey
   ..........KI..K(.E.)LL...F.KAN..E   O   pri   Bushbaby
   .T.R.L...S...RR(K.T)L..R..GM....E   X   eua   Treeshrew
   .....H...T....N(E..)L..T.W.EG...N   X   gli   Squirrel
   .E..E...HSLT.NI(E.F)A....L.E..K.Q   X   gli   Lesser Egyptian jerboa
   .E..GYT.TSKS.NH(R.L)V..M.L.E....L   X   gli   Prairie vole
   .E..GHT.TSKS.NH(R.L)V..T.---....M   X   gli   Chinese hamster
   .E..GRT.TSES.NH(G..)V..T.---.SS.L   X   gli   Golden hamster
   .E..G.MPTQES.NS(S..)V..T.L.E..E.F   X   gli   Mouse
   .EM.G.M.TLES.NS(R.F)V..T...E..EKL   X   gli   Rat
   .....R..SA.....(K..)L..T..LE...QE   X   gli   Naked mole-rat
   .....LQ..A....H(D..)LT.S.WLEA..QE   X   gli   Guinea pig
   .....RT..A....Q(...)L..S.WLE...QE   O   gli   Chinchilla
   .....HK..T....R(D..)L..S.PLE...QE   X   gli   Brush-tailed rat
   .E..GRK.DA..SH.(K.A)V..M...RG...E   X   gli   Rabbit
   .Q..GRE..T..SHA(E..)VR.T...E..V.E   X   gli   Pika
   ..R..H.PPL.I.HN(G..)LL..TWING.EKE   X   lau   Pig
   M.....K.E.KIMNH(G..)LL.T.W.KSN-.E   X   lau   Alpaca
   M.....N.E.KIVNH(G..)LL.T.W.KSN-.E   X   lau   Bactrian camel
   .....R..QT.I..N(EY.)LL...L.EGKV.E   X   lau   Dolphin
   .....R..QT.I..N(EY.)LL...L.EGKV.E   X   lau   Killer whale
   .V..E.K.ESNI..N(G..)VL.E.WTEEN--E   X   lau   Tibetan antelope
   .V....K.ESNI..D(G..)VL...WMEEN--E   X   lau   Cow
   VQ....K.ESNI..N(G..)VL.E.WTEEN--E   X   lau   Sheep
   .V....K.ESNI..N(S..)VL...WTEEN--E   X   lau   Domestic goat
   ...E.H...A.I..N(G..)LV.R.PMQGL.GE   X   lau   Horse
   .G...R..Q.....K(G..)...T.L.KAH..E   X   lau   White rhinoceros
   .E.SGRN.H....HN(...)LL.T.HDKANM.E   O   lau   Cat
   ...AEE..HS..L.K(K..)LL.T.N.KANP..   X   lau   Dog
   .T.AEKK.DS.SL..(K..)VL.T.N.KANSQK   X   lau   Ferret
   .T.AE.K.HS.PL..(K..)VL.T.I.KAN..E   X   lau   Panda
   .T.AE.K.PS.SL.S(K..)VL.T.N.KAN.AQ   X   lau   Pacific walrus
   .M.AE.K.HS.SL.S(K..)VL.M.N.EAN.AE   X   lau   Weddell seal
   .V..E.K.E...K.N(D.F)LW.S.W.EAKS.E   X   lau   Black flying-fox
   .V..E.K.E...K.N(D.F)LW.S.W.EAKS.E   X   lau   Megabat
   ...E....E.RIK.S(K..)L..T.L.EMAPR.   X   lau   David's myotis bat
   ...E....K..IK.S(E..)L..T.L.E.APR.   X   lau   Microbat
   ...E....K..IK.S(K..)LL.T.V.EG.PGE   X   lau   Big brown bat
   .EV.G.Q...KI.PR(T.A)VWQV.---TKW.Q   X   lau   Hedgehog
   .V...DP.RTVI..R(E.F)I.RAMAMAA---E   X   lau   Shrew
   .I..N.S.H...KHK(K.A)LM---W.Q.KV.E   X   lau   Star-nosed mole
   M....R..S.MIKSN(ARA)LL.T.T.TADSAE   X   afr   Elephant
   M....HQ...VTVRN(MSA)LE.T.Q.TADT..   X   afr   Cape elephant shrew
   .S..GR..S..IKDS(D.L)LL.T.M.MADLDQ   X   afr   Manatee
   .T...TP.S.VMVSN(AAP)E.VM.TAVANKYE   X   afr   Cape golden mole
   MT........VTVPD(AAP)L.VT...VADSDE   X   afr   Tenrec
   M.......S.VI.SR(GSM)L..T.V.RADNDE   X   afr   Aardvark
   ......P..A..RHN(A.A)L..T.T.TALPDE   X   xen   Armadillo
   .TW.G.V.KVN.IQH(A.M)LR.T.TIMTSQ.V   X   mar   Opossum
   .SW.G.I.KNST.RQ(GNL)LK.T.TITAQ---   X   mar   Tasmanian devil

NO 59
GN ICAM2
ID ICAM2_HUMAN
MP 153
DE Intercellular adhesion molecule 2
CL apes
SQ 60
   TVEPLDSLTLFLFRG(NET)LHYETFGKAAPAP   O   hum   Human
   ...............(...).............   O   hac   Chimp
   ...............(...)..NQ........L   O   aga   Gorilla
   ...............(...)..NQ........L   O   gra   Orangutan
   ..K............(...)..SQ........L   O   ape   Gibbon
   A.........S.L..(S..)..SQ........L   X   cat   Rhesus macaque
   A.........S.L..(S..)..SQ........L   X   cat   Crab-eating macaque
   A.........S.L..(S..)..SQ........L   X   cat   Baboon
   A.........S.L..(S..)..SQ........L   X   cat   Green monkey
   A..............(...)..SQ....G...L   O   sim   Marmoset
   A..............(...)..SQ....G...M   O   sim   Squirrel monkey
   A.K..EN...T....(S..).YNQ..ERQT...   X   pri   Bushbaby
   A.K..EN...T..H.(K.I)..HQ..AGGTLD.   X   eua   Treeshrew
   A.K..E....T....(K..)..SQ..SGG.TD.   X   gli   Squirrel
   D.K..E....T.L..(K..).QNQ...G..L..   X   gli   Lesser Egyptian jerboa
   A.K..E....S.LHE(G..).QNQ..EG.----   X   gli   Prairie vole
   A.K..E....T.L..(R..).QNQ..EGTEL--   X   gli   Chinese hamster
   A.K..E....T.L..(R..).QNQ...GT----   X   gli   Golden hamster
   P.Q..ER...S.L..(R..).KNQ...G.ETV.   X   gli   Mouse
   S.K..E....S.LC.(R..).KNQ...G.ETV.   X   gli   Rat
   A....E....T.L..(Q..)..NQ..VG.....   X   gli   Naked mole-rat
   A....E....TV...(Q..)V.NQ..VG...V.   X   gli   Guinea pig
   A.K..S....S....(Q..)..NQ..AG..A.A   X   gli   Chinchilla
   A.K..EG...T.Q..(Q..).QNW.SVE....R   X   gli   Brush-tailed rat
   A....E....I.LH.(.K.)..NQ..EGR.AV.   O   gli   Rabbit
   S.A.FET..IT.L..(S.I).YN..LRGTT.S.   X   lau   Pig
   S.A..EG..VT.L..(S.V)..SQ..EGT.LS.   X   lau   Alpaca
   S.A..EG..VT.L..(S.V)..SQ..EGT.LS.   X   lau   Bactrian camel
   S.A..EG..VT.LH.(S.I)VYSQ..VGTTLS.   X   lau   Dolphin
   S.A..EG..VT.LH.(S.I)VYSQ..VGTTLS.   X   lau   Killer whale
   A.A..EG..VT.L..(T.I).YNQ..VGT..SL   X   lau   Tibetan antelope
   A.A..EG..VT.L..(T.I)..NQ..EET..F.   X   lau   Cow
   A.A..EG..VT.L..(T.I).YIQ..VGT..S.   X   lau   Sheep
   A.A..EG..VT.L..(T.I).YNH..VGT..S.   X   lau   Domestic goat
   A..AFE....I.L..(K..)..RK..E.-T.G.   X   lau   Horse
   A.A..ER...T.L..(KK.)..NK..E.TM.D.   X   lau   White rhinoceros
   N.A..E....T.L..(K.A)M.NK..RTM.L..   X   lau   Cat
   A.K..E....M.L..(Q.V)..NQ..WGTTDGT   X   lau   Dog
   D.K..E....T.L..(K..).CNK..TGEKNDT   X   lau   Ferret
   A.R..E....T.LH.(K.A).CNK..TGEKNGT   X   lau   Panda
   A.K..E....T.LH.(Q.A).CNK..ARGDDSI   X   lau   Pacific walrus
   A.K..EG...T.L..(Q.A).CNK..ARGDSGT   X   lau   Weddell seal
   A.A..EK...T.LH.(KKP)..TQ...SGKDD.   X   lau   Black flying-fox
   A.A..EN...T.L..(Q.P)..MQR.EGETA..   X   lau   David's myotis bat
   A.A..EN...T.L..(Q.P)..VQR.EG.TA..   X   lau   Microbat
   A.A..EN...T.L..(Q.P)..MH..VG.TA..   X   lau   Big brown bat
   A.A..S....S.L..(E.I)..NQ..QET.AD.   X   lau   Hedgehog
   A.A..E....T.LH.(HQP)..SQ..RQG..G.   X   lau   Shrew
   A.A..EN...T.LQ.(SQE)R.GKNV--S.V.T   X   lau   Star-nosed mole
   ..A..R....T.L..(..M)..NQ..EQ.TA..   X   afr   Elephant
   N.A..EN...T...D(R.I).GTQ..QN.TSD.   X   afr   Cape elephant shrew
   G.A..E....T.L..(R..).YNQ..EG.TVV.   X   afr   Manatee
   ..A..E....T.L..(K.A)..NQ..KR.V.S.   X   afr   Cape golden mole
   A.A..E....T.L..(RDP)..NQ...R.TS..   X   afr   Tenrec
   A.A..K....T.LH.(RKM).YNQ..E..T.D.   X   afr   Aardvark
   A.A..E....T.L..(Q..).YN...ER.T...   X   xen   Armadillo
   N.G..EN...T.LQ.(T.M)..RQ..KTVSKVL   X   mar   Opossum
   N.V..EN..VT.LQ.(T.K)..RQ..VTESK.L   X   mar   Tasmanian devil
   N.V..EK...T..Q.(T.K).YHQ..VTVSA.L   X   mar   Wallaby
   N.M..EN..MA.LQ.(TQR)VGLKD.PGKLTGL   X   mon   Platypus

NO 60
GN ICAM3
ID ICAM3_HUMAN
MP 84
DE Intercellular adhesion molecule 3
CL African great apes
SQ 56
   LSKELVASGMGWAAF(NLS)NVTGNSRILCSVY   O   hum   Human
   ...............(...).............   O   hac   Chimp
   ...............(...)........F....   O   aga   Gorilla
   ......DN.......(Y..).............   X   gra   Orangutan
   ......DN.T.....(Q..)...........G.   X   cat   Rhesus macaque
   ......DN.T.....(Q..)...........G.   X   cat   Crab-eating macaque
   ......GN.T.....(Q..)...........G.   X   cat   Baboon
   ......GN.T.....(Q..)...........G.   X   cat   Green monkey
   ......DN.T....H(RV.)...........G.   X   sim   Marmoset
   ......DN.T.....(WI.)K...DN.....G.   X   sim   Squirrel monkey
   .L....GNSL.....(R..)...S...V...GF   X   pri   Bushbaby
   ....SAG..L.....(Q..)....D.Q.F...F   X   eua   Treeshrew
   .P..VIGEDQ.....(R..)....D.K.I..AF   X   gli   Squirrel
   ....PIGE.V.....(Q.I)...VD.QL...G.   X   gli   Lesser Egyptian jerboa
   ..*KPIS--L.....(Y.H)P..S..QL-----   X   gli   Rat
   .P.Q.NG.SP...T.(W..).L.EDT..M...L   X   gli   Naked mole-rat
   .P.Q.NG..P.*.T.(W..).L.ED.T.I..AL   X   gli   Guinea pig
   .L.QPNG..P...T.(W..).M.ED.T.I..AL   X   gli   Chinchilla
   .P.Q.NG..P...T.(W..).L.ED.T.I...L   X   gli   Brush-tailed rat
   ......GQ.Q..V..(L..)GASDD.QV...G.   X   gli   Rabbit
   F..RQAGQ.P..T..(W.D)K..S.NP.F..GF   X   gli   Pika
   .LW.P.G..R.....(Q.N)....DTQFF.FGL   X   lau   Pig
   .L..T.GR.L.....(Q..)....DTMV...GF   X   lau   Alpaca
   .L..T.GR.L.....(H..)....DTTV...GF   X   lau   Bactrian camel
   .L..S.GR.L.....(Q..)....DTQL...GF   X   lau   Dolphin
   .L..S.GR.L.....(Q..)....DTQL...GF   X   lau   Killer whale
   ....HHSR.L.....(R.T)....DMEL...GL   X   lau   Tibetan antelope
   ....PHSR.L.....(R.T)....DME....GI   X   lau   Cow
   ....HHSR.L.....(R.T)....DTEL...GL   X   lau   Sheep
   ....HHSR.L.....(R.T)....DMEL...GL   X   lau   Domestic goat
   .P..PLS..L.....(R..)...DDREV...SF   X   lau   Horse
   .P.KSLGQ.L.....(Q..)...ND.Q....TF   X   lau   White rhinoceros
   .P.KP.GN.L.....(Q..)S..SD.QV...GF   X   lau   Cat
   .A.KP.GN.L.....(L..)...SD.QV...GF   X   lau   Dog
   ....P.GN.P.....(Q.F).L..DTQV...GF   X   lau   Ferret
   ....P.GN.L.....(Q..).A.TD.QV...GF   X   lau   Panda
   .F..A.GN.L.....(Q..)...SD.QVF..GF   X   lau   Pacific walrus
   ....P.GN.L.....(Q..)...S..QV...GF   X   lau   Weddell seal
   .F..T.G..L..E..(Q.N)....D.KV....F   X   lau   Black flying-fox
   .F..T.G..L..E..(Q.N)....D.KV....L   X   lau   Megabat
   .F..EAGE.L..R.Y(W..)...SD.E.F..GI   X   lau   David's myotis bat
   .F..EAGE.L..K.Y(R..)S..SD.E.F..GI   X   lau   Microbat
   .L..E.GT.R..K..(Q..)...SD.K.F..AI   X   lau   Big brown bat
   F..DTLGK.R...T.(R..)...SDHKL...S.   X   lau   Hedgehog
   ...KM...SQ..ET.(E.T)...EDAVVI.ATR   X   lau   Shrew
   ...QP.G..LS.S..(Q..)....D.D.I.AGF   X   afr   Elephant
   ....PAGR.PN.S..(W.T)..SADDDAI..AF   X   afr   Cape elephant shrew
   ......D..LS.S..(Q..)...SD.K.I..SF   X   afr   Manatee
   ....S.GR.PS.VTY(R.L)....D.EVI.AGF   X   afr   Cape golden mole
   .L..PAGR.PS.V..(R..)...ADKE.F.AGF   X   afr   Tenrec
   .F.K..DN.PS.S..(L..)...SD.DVI.VGL   X   afr   Aardvark
   .....RGRSN..V..(Q.H)..ADD.QV..A.T   X   xen   Armadillo
   .N.TQ..N.TR.KE.(L.R)..IKDTEL..FAN   X   mar   Opossum
   .D.LPENN.TR.R..(R.K).I.QD.LL..FAN   X   mar   Tasmanian devil
   .D.TQEDK.VR.ST.(L.R).I.QDTLL..F.N   X   mar   Wallaby
   .H.AVN...TH.RTY(VVK)AGADQG.V..FAN   X   mon   Platypus

NO 61
GN IFNG
ID IFNG_HUMAN
MP 48
DE Interferon gamma
CL simians
SQ 59
   NLKKYFNAGHSDVAD(NGT)LFLGILKNWKEES   O   hum   Human
   ...............(...).............   O   hac   Chimp
   ...............(...).............   O   aga   Gorilla
   .........D.....(...).............   O   gra   Orangutan
   .........D....N(...).............   O   ape   Gibbon
   .........DP....(...)...D..R......   O   cat   Rhesus macaque
   .........DP....(...)...D..R......   O   cat   Crab-eating macaque
   ........VDP....(...)...D..R......   O   cat   Baboon
   .........DP....(...)...D..R......   O   cat   Green monkey
   .........D.....(...)...D..RT.R..G   O   sim   Marmoset
   .........D.....(...)...N..RT.R..G   O   sim   Squirrel monkey
   Q..E...TSST.T.G(G.I)...D.....EK..   X   pri   Bushbaby
   ...N....TDPG.G.(T.R)..VD.....T...   X   eua   Treeshrew
   A..E....SN.N.S.(G.S)...D..DR.E...   X   gli   Squirrel
   K..E....SK....N(GEI)...N.S.K.Q..K   X   gli   Lesser Egyptian jerboa
   T......STS...GE(G.D).LFE.....Q.DG   X   gli   Prairie vole
   .......SSSE..GN(G.D).VFNT.M..QKDG   X   gli   Chinese hamster
   .......SSSL..VN(G.D).VFN..T..QKAG   X   gli   Golden hamster
   S..N...SSSM.AME(GKS).L.D.WR..QKDG   X   gli   Rat
   I..D....SS...G.(...)..V.....CQ...   O   gli   Naked mole-rat
   I..N....DN...G.(...)..V.....CQ...   O   gli   Guinea pig
   F.....K.DN...G.(...)..VD....CQ...   O   gli   Chinchilla
   V.AD..E.QG.EAGA(K.H)..ID....CQN..   X   gli   Brush-tailed rat
   H..A.LK.NT....N(G.P)...N..R......   X   gli   Rabbit
   QI.E..K.NT....T(G.P)...N..R......   X   gli   Pika
   I..D....ST...PN(G.P)...E.........   X   lau   Pig
   ........S.P....(G.P)...E.........   X   lau   Alpaca
   ........SNP....(G.P)...E.........   X   lau   Bactrian camel
   ...E....SNP...G(G.P)...E..E...D..   X   lau   Dolphin
   ...E....SNP...G(G.P)...E..E...D..   X   lau   Killer whale
   ...E....SNP...K(G.P)..SE.........   X   lau   Tibetan antelope
   ...E....SSP...K(G.P)..SE......D..   X   lau   Cow
   ...E....SNP...K(G.P)..SE.........   X   lau   Sheep
   ...E....SNP...K(G.P)..SE.........   X   lau   Domestic goat
   ...E....SNP..G.(G.P)...D.......D.   X   lau   Horse
   ...E....SNP....(G.S)...D.........   X   lau   White rhinoceros
   E..G....SNP....(G.S)..VD.........   X   lau   Cat
   ...E....SNP..S.(G.S)..VD...K.R...   X   lau   Dog
   D..E....SNP....(G.P)...D.....R...   X   lau   Ferret
   ...E....SNP....(G.P)...D.....R...   X   lau   Panda
   ...E....SNP....(G.P)...D.....R...   X   lau   Pacific walrus
   ...E....SNP....(G.P)...D.....R...   X   lau   Weddell seal
   ...E....SN.N...(G.N)...D.....R...   X   lau   Black flying-fox
   ...E....SN.N..N(G.N)...D.....R...   X   lau   Megabat
   K..R....TS...GN(G.H)...D..M...G..   X   lau   David's myotis bat
   K..R....TS...GN(G.H)...D..T...G..   X   lau   Microbat
   K..R....TSP.I.N(G.R)...D..T..EG..   X   lau   Big brown bat
   K..E....TS.S..S(G..)...DTM.K.....   X   lau   Hedgehog
   ....H.S.NY...S.(..P)...N...K.NK..   X   lau   Shrew
   ...E....SS.....(GKP)...E.........   X   lau   Star-nosed mole
   ...E.L..TD.....(G.P)..ID.......D.   X   afr   Elephant
   I..E.L..TD.S...(G.P)....F.R.LT...   X   afr   Cape elephant shrew
   .....L..TG.....(G.P)...D.........   X   afr   Manatee
   ...E.L..TD.....(GRP)..SE...T....G   X   afr   Cape golden mole
   ...E....SDA....(GKP)..SD..R..SH.T   X   afr   Tenrec
   T..E.L..TN....T(D.P)...N..E......   X   afr   Aardvark
   T..E....SD.....(G.P)...D.........   X   xen   Armadillo
   I.MD...GST..ISE(...)...NMMDR...D.   O   mar   Wallaby
   ...E.Y..SEP...E(D.P)..VMM..DAQQD-   X   mon   Platypus

NO 62
GN IFNG
ID IFNG_HUMAN
MP 120
DE Interferon gamma
CL simians
SQ 60
   FNSNKKKRDDFEKLT(NYS)VTDLNVQRKAIHE   O   hum   Human
   ...............(...).............   O   hac   Chimp
   ...............(...).............   O   aga   Gorilla
   ...............(...).............   O   gra   Orangutan
   ...............(...).............   O   ape   Gibbon
   ...............(...)...S......V..   O   cat   Rhesus macaque
   ...............(...)...S......V..   O   cat   Crab-eating macaque
   .......W.......(...)..........V..   O   cat   Baboon
   ...............(...)..........V..   O   cat   Green monkey
   .....R.Q....R..(...).N...........   O   sim   Marmoset
   .....R.Q....R..(...).............   O   sim   Squirrel monkey
   ...SYS.AEA.QN.L(RI.).H..Q......S.   X   pri   Bushbaby
   ...SPT.L.V.LN..(RI.).N.VQ......Y.   X   eua   Treeshrew
   ....CI..E..L.VS(QFQ).N..KI....VR.   X   gli   Squirrel
   ..NSVD.LW..LNI.(QI.).NNQL......N.   X   gli   Lesser Egyptian jerboa
   ..NSEA.VN....IA(KIP).D.PQ.....VN.   X   gli   Prairie vole
   ...SME.LN..VRI.(KIP).N.VQ.....VN.   X   gli   Chinese hamster
   ...SME.LN..V...(KIP).N..Q.....VN.   X   gli   Golden hamster
   .SNS.A.K.A.MSIA(KFE).NNPQ...Q.FN.   X   gli   Mouse
   .SNS.A.K.A.MSIA(KFE).NNPQI.H..VN.   X   gli   Rat
   .H..SS.L.V.QS..(RI.).DNVDF..R..I.   X   gli   Naked mole-rat
   .KD.SSNKEA.KN.I(QI.).N.EH...Q..I.   X   gli   Guinea pig
   .SN.YYTLEA.QN..(RI.).K.VH...Q..I.   X   gli   Chinchilla
   LR-.QSNLEI.QN..(QL.).R.K....Q..I.   X   gli   Brush-tailed rat
   ....LT.M...QN..(RI.).D.RL.....VS.   X   gli   Rabbit
   ...SLT.LQ..QN..(QI.).D.QQ......S.   X   gli   Pika
   L.GSSG.LN.....I(KIP).DN.QI.....S.   X   lau   Pig
   L.GSSE.LE..K..I(QIP).DN.K......S.   X   lau   Alpaca
   L.GSSE.LE..K..I(QIP).DN.K......S.   X   lau   Bactrian camel
   L.GSSE.L...K..I(QIP).D..QI.....S.   X   lau   Dolphin
   L.GSSE.L...K..I(QIP).D..QI.....S.   X   lau   Killer whale
   L.GSSE.LE..K..I(QIP).D..QI.....N.   X   lau   Tibetan antelope
   L.GSSE.LE..K..I(QIP).D..QI.....N.   X   lau   Cow
   L.GSSE.LE..KR.I(QIP).D..QI.....N.   X   lau   Sheep
   L.GSSE.LE..K..I(QIP).D..QI.....N.   X   lau   Domestic goat
   ...STS.LE..Q..I(QIP).N..K......S.   X   lau   Horse
   ...STS.L...K..I(QIP).D..Q......S.   X   lau   White rhinoceros
   L.TSSS.....L..I(QIP).N..Q......N.   X   lau   Cat
   L..STS..E..L..I(QIP).N..Q......N.   X   lau   Dog
   ..NSSS.LE..L..I(RIP).N..Q......N.   X   lau   Ferret
   ...STS..S..L..I(QIP).N.MQ......N.   X   lau   Panda
   ...SNS.....L..I(RIP).N..Q......N.   X   lau   Pacific walrus
   ...SNS.....L..I(RIP).N..Q......N.   X   lau   Weddell seal
   ....NS.LE..K.VI(QIP).NNQT......S.   X   lau   Black flying-fox
   ....NS.LE..K.VI(QIP).NNQT......S.   X   lau   Megabat
   ...SNS.ME..K..I(QTP).N.QR......S.   X   lau   David's myotis bat
   ...SNS.ME..K..I(QTP).N.QR......Y.   X   lau   Microbat
   ...SNS.ME..K..I(QIP).N..R......S.   X   lau   Big brown bat
   ..G.LS..E.....A(.I.)MN.GM...R..S.   O   lau   Hedgehog
   ..KS.E.L......M(GIQ).N.IK..K...N.   X   lau   Shrew
   ...SDS.KE..KT.I(KIQ).N.M.......S.   X   lau   Star-nosed mole
   ...SSS.....L.VM(QTP).N.R.I.....S.   X   afr   Elephant
   ...D.T.ISN.TE.I(KTP).D.RM.....VS.   X   afr   Cape elephant shrew
   ....SS.....L.VM(QTP).N.R.......S.   X   afr   Manatee
   ...SIN.WNE.L..A(KTP)AN.RKI.....N.   X   afr   Cape golden mole
   ...SST.QE..LR.I(KTP).N.QK......N.   X   afr   Tenrec
   ...SES.QK..LD.L(KIQ)ANERK......S.   X   afr   Aardvark
   ...SSS.LN..L..I(RTP).N..K.....VN.   X   xen   Armadillo
   ..NTAS.VN...AVI(.TQ)-------------   X   mar   Wallaby
   QTHGF..LG.LQ..I(KT.).S.AKI....V..   X   mon   Platypus

NO 63
GN IL17F
ID IL17F_HUMAN
MP 83
DE Interleukin-17F
CL simians
SQ 61
   VSMSRNIESRSTSPW(NYT)VTWDPNRYPSEVV   O   hum   Human
   ...............(...)..R..........   O   hac   Chimp
   ...............(...).............   O   aga   Gorilla
   ...............(...).............   O   gra   Orangutan
   ...............(...).............   O   ape   Gibbon
   ...............(...).............   O   cat   Rhesus macaque
   ...............(...).............   O   cat   Crab-eating macaque
   ...............(...).............   O   cat   Baboon
   ...............(...).............   O   cat   Green monkey
   .PL.....R......(...).............   O   sim   Marmoset
   .PL.....R......(...).........L.I.   O   sim   Squirrel monkey
   ..K.Q..QN..F...(D..)ADR......FVIY   X   pri   Bushbaby
   FLV.H.VHN......(D.N)I.R.SE.F...IA   X   eua   Treeshrew
   IAI.HDFQN..I...(D.N)I.R..H.F...IA   X   gli   Squirrel
   I.V.HDLQN..S...(D.N)I.R..H.F.A.IA   X   gli   Lesser Egyptian jerboa
   ..V.HGFQN..S...(D.N)I.R..H.F...IA   X   gli   Prairie vole
   .FV.HDFQN..S...(D.N)I.R..H.F...IA   X   gli   Chinese hamster
   IYV.QDFQN..S...(D.N)I.R..H.F...IA   X   gli   Golden hamster
   I.VP.EFQN..S...(D.N)I.R..H.F...IA   X   gli   Mouse
   I.VP.DFQN..S...(D.N)I.R..D.F...IA   X   gli   Rat
   ILITHDFRN..S...(D.N)I.R..H.F.T.I.   X   gli   Naked mole-rat
   TPITHDFRN..S...(D.N)..R..H.F.P.I.   X   gli   Guinea pig
   TPIPHDFRN..S...(D.N)I.R..H.F.T.I.   X   gli   Chinchilla
   MPVTHDFRN..S...(D.N)I.R..H.F.T.I.   X   gli   Brush-tailed rat
   .PITHDFQN..I...(D.N)I.R..H.F...IA   X   gli   Rabbit
   .PITHDFQN..I...(D.N)I.Q..H.F...IA   X   gli   Pika
   .PL.HDFQN..S...(D.N)I.R..H.F...IA   X   lau   Pig
   .PL.HVFQN..S...(D.N)I.R....F...IA   X   lau   Alpaca
   .PL.HDFQN..S...(D.N)I.R....F...IA   X   lau   Bactrian camel
   IPL.HGFQN..ST..(D.N)..R..H.....IA   X   lau   Dolphin
   IPL.HGFQN..ST..(D.N)..R..H.....IA   X   lau   Killer whale
   ..F...LQN..I...(D.N)I.R....F...IS   X   lau   Tibetan antelope
   .FF...LQN..I...(D.N)I.R.A..F...IA   X   lau   Cow
   ..F...LQN..I...(D.N)I.R....F...IA   X   lau   Sheep
   ..F...LQN..I...(D.N)I.R....F...IA   X   lau   Domestic goat
   I.SLHD.HN..S...(V..).SR....F...IA   X   lau   Horse
   F.S..D.QN..S...(V.N)I.R....F...I.   X   lau   White rhinoceros
   I.I.NDFQN..S...(D.N)..R....F...I.   X   lau   Cat
   I.I.NDFQN..S...(D.N)I.R..H.F...IA   X   lau   Dog
   I.I.-.FQN..I...(D.N)I.Q..H.F...IA   X   lau   Ferret
   IPI.NDFQN..S...(D.N)I.R..H.F...IA   X   lau   Panda
   I.I.NDFQN..S...(D.N)I.R..H.F...IA   X   lau   Pacific walrus
   I.I.NDFQN..S...(D.N)I.R..H.F...IA   X   lau   Weddell seal
   ILI.HDFQN..S...(D.N)..Q..H.F...IA   X   lau   Black flying-fox
   ILI.HDFQN..S...(D.N)..Q..H.L...IA   X   lau   Megabat
   ..I.HDLQN..S...(D.N)I.R..H.F...IA   X   lau   David's myotis bat
   ..I.HDLQN..S...(D.N)I.R..H.F...IA   X   lau   Microbat
   .TI.HDFQN..S...(D.N)I.R..H.F...IA   X   lau   Big brown bat
   ..I.SD.QN..I...(..N)I.Q....F...IA   X   lau   Hedgehog
   .PV.Q.FQN..I...(D.N).....H.I..SIA   X   lau   Shrew
   ...AQ.FQN..I...(D.N)RNE..E...PVIW   X   lau   Star-nosed mole
   GPTLPS.NN......(D.N)I.R.L..F..SIA   X   afr   Elephant
   SPT.P..NN......(D.N)I.Q.I..F..SIA   X   afr   Cape elephant shrew
   GHTLP..NN......(D.N)IMR.M..F..SIA   X   afr   Manatee
   N.I.S..NN......(D.S)I.E..G.F..PIS   X   afr   Cape golden mole
   GPILS..NN......(D.K)L.K.L..I.FSIA   X   afr   Tenrec
   SPILP..SN......(Y.N)I.Q.L..F..SIA   X   afr   Aardvark
   .HF.HDFQN..S...(D.N)I.R..D.F...IA   X   xen   Armadillo
   IHSA.AFQN..I...(D.N)I.K..D.F...IA   X   mar   Opossum
   IRFAHTFQN..I...(D.N)I.K....F...IA   X   mar   Tasmanian devil
   APVFHDYRN..V..R(D.S)INR..H.I.Q.LA   X   mon   Platypus

NO 64
GN IL6
ID IL6_HUMAN
MP 73
DE Interleukin-6
CL simians
SQ 59
   RYILDGISALRKETC(NKS)NMCESSKEALAEN   O   hum   Human
   ...............(...).............   O   hac   Chimp
   ...............(...).............   O   aga   Gorilla
   ...............(...).............   O   gra   Orangutan
   ...............(.R.)..........V..   O   ape   Gibbon
   ...............(.R.).............   O   cat   Rhesus macaque
   ...............(.R.).............   O   cat   Crab-eating macaque
   ........V......(.R.).............   O   cat   Baboon
   ...............(.R.).............   O   cat   Green monkey
   .............I.(...).............   O   sim   Marmoset
   W............I.(...).............   O   sim   Squirrel monkey
   TH.IME.ND.NGKM.(S.G)IK..GDSHVMEN.   X   pri   Bushbaby
   K...EE.NV.K-QA.(DSI)YK----RV...K.   X   eua   Treeshrew
   TF..RR.LD..T.L.(DND)ED.LENE...S..   X   gli   Lesser Egyptian jerboa
   S...REVFEM...L.(DN.)PD.MANDD..S..   X   gli   Prairie vole
   THV.RE.FE....L.(.NN)PD.MNYDD..L..   X   gli   Chinese hamster
   T.V.RE.YE....L.(.NN)PG.MDNDYV.L..   X   gli   Golden hamster
   THV.WE.VEM...L.(.GN)SD.MNNDD.....   X   gli   Mouse
   T.V.RE.LEM...L.(.GN)SD.MN.DD..S..   X   gli   Rat
   TLV.HDVQE.KS...(KHN)VN.LEEEK.M--.   X   gli   Naked mole-rat
   MRVYQAVKE.KN.MN(KHN)VE-------K.I.   X   gli   Guinea pig
   --VYKDVKE.KD.MS(EHN)VET-------VTD   X   gli   Brush-tailed rat
   .S..ET.KE....M.(DHD)VN.MNR......V   X   gli   Rabbit
   S...MK..DI..KM.(.YD)AN.LDC.QV.SDV   X   gli   Pika
   K...GK...M...M.(E.Y)EK..N...V....   X   lau   Pig
   K...GR...M...M.(E.Y)DK..N.....S..   X   lau   Alpaca
   K...GR...M...M.(E.Y)DK..N.....S..   X   lau   Bactrian camel
   K...GK...M...M.(E.Y)DK..N........   X   lau   Dolphin
   K...GK...M...M.(E.Y)DK..N........   X   lau   Killer whale
   KH.V.K.......I.(E.N)DE..N...T....   X   lau   Tibetan antelope
   KRMV.K...M...I.(E.N)DE......T....   X   lau   Cow
   KH.V.K...I...I.(E.N)DE..N...T....   X   lau   Sheep
   KH.V.K...I...I.(E.N)DE..N...T....   X   lau   Domestic goat
   K...GK....KN.M.(.NF)SK..N...V....   X   lau   Horse
   K....K....K.-M.(.NF)SK..N...I....   X   lau   White rhinoceros
   K...GK....K..M.(DNY).K..D........   X   lau   Cat
   K...GK.......M.(D.F).K..D........   X   lau   Dog
   .F..GK....K..M.(E.Y).K..D........   X   lau   Ferret
   K...GR....K..M.(D.Y).K..D........   X   lau   Panda
   K...GK.......M.(D.Y).K..D........   X   lau   Pacific walrus
   K...GK.......M.(D.Y).K..D........   X   lau   Weddell seal
   KS.FLE..EV.NKM.(GND)DS.KN...V.T..   X   lau   Black flying-fox
   KS..LE..DVKNKM.(DNH)ES.KN...V.T..   X   lau   David's myotis bat
   KS..LE..DVKNKM.(DNH)ES.KN...V.T..   X   lau   Microbat
   KS..LE..DMKNKM.(DNH)ES.KN.....T..   X   lau   Big brown bat
   ....GK..E.KQ.I.(ENH)S..RNGMV..ED.   X   lau   Hedgehog
   K...GK..L.K..L.(G.Y)GK..NV.......   X   lau   Shrew
   K...IK....KN.M.(K.Y)DK.DNN.......   X   lau   Star-nosed mole
   .F..AE..V...KM.(D.Y)DK..N.R....G.   X   afr   Elephant
   KF.IEQ....K..V.(E.F)DK...IS....G.   X   afr   Cape elephant shrew
   NF..AKV.V....M.(D.Y)DK.DN.R....G.   X   afr   Manatee
   SF..SQVVE.KN.M.(D.Y)DK..NT-.V..G.   X   afr   Cape golden mole
   MF..SQVEE....I.(D.Y)DK..N.R......   X   afr   Tenrec
   TT.RFQVTE....M.(D.Y).K..NTTV...R.   X   afr   Aardvark
   MF..SK..E....I.(E.Y)GK.GKTP......   X   xen   Armadillo
   K.LEKTA.D.KE.I.(RIH).L.DN.N......   X   mar   Opossum
   -------------NG(ENQ)S..DN.STI....   X   mar   Tasmanian devil
   -------------M.(LTQ)S..DD.N......   X   mar   Wallaby
   KTLWKQANG.KDKI.(DDY)SL..EN.VV....   X   mon   Platypus

NO 65
GN ITGAM
ID ITAM_HUMAN
MP 946
DE Integrin alpha-M
CL simians
SQ 55
   SHGVSTKYLNFTASE(NTS)RVMQHQYQVSNLG   O   hum   Human
   ...............(...).............   O   hac   Chimp
   ..E............(...).............   O   gra   Orangutan
   ..E......S.....(...)Q............   O   ape   Gibbon
   ..E.....I......(...).........K...   O   cat   Rhesus macaque
   ..E.....I......(...).........K...   O   cat   Crab-eating macaque
   ..E.....I......(...).........K...   O   cat   Baboon
   ..E............(...).........K...   O   cat   Green monkey
   ..E............(...).............   O   sim   Marmoset
   ..E............(...).............   O   sim   Squirrel monkey
   .DE..........F.(K..)K.I..H..FN...   X   pri   Bushbaby
   .QEF...........(KA.)K....H..FN...   X   eua   Treeshrew
   .TE............(K..)H.I.....FN...   X   gli   Squirrel
   .SEI..R........(MS.)QFI....EFN...   X   gli   Lesser Egyptian jerboa
   .DEN...........(M..)QDI.....FN...   X   gli   Prairie vole
   .DES....F......(M..)Q.I.....FN...   X   gli   Chinese hamster
   .DES....F......(M..)Q.I.....FN...   X   gli   Golden hamster
   .DES.IR........(M..)K.I.....FN...   X   gli   Mouse
   .GES.I.........(M..)K.I.....FN...   X   gli   Rat
   .GEA.....S.....(KA.)QA.E....F....   X   gli   Naked mole-rat
   .GEA..T.....T..(K.I)QT.K...KFT...   X   gli   Guinea pig
   .GEA...........(K..)QI.E....F....   X   gli   Brush-tailed rat
   .GE............(KST)..L..E..FN...   X   gli   Rabbit
   .GE............(KSR)H.I..E..IN...   X   gli   Pika
   .LE.....F......(K.R)H.IE....FN...   X   lau   Pig
   .LE............(..R)HIIE....FN...   X   lau   Alpaca
   .LE............(K.R)HIIE....FN...   X   lau   Bactrian camel
   .LE............(K.H)HIIE....FN...   X   lau   Dolphin
   .LE.....-...T..(K.R)HIIE....FN...   X   lau   Killer whale
   .LEA...........(K.R)H..E....FN...   X   lau   Tibetan antelope
   .LEA...........(K.V)HA.E....FN...   X   lau   Cow
   .LEA...........(K.R)H..K....FN...   X   lau   Sheep
   .LEA...........(K.R)H..K....FN...   X   lau   Domestic goat
   .GEA...........(K..).IIK.E..FK...   X   lau   Horse
   .RE...........K(DI.).IIN.E..FK...   X   lau   White rhinoceros
   ..E......S.I...(K..)HSIK...E.N...   X   lau   Cat
   ..EG.........A.(K..)HIIE.Y.EIN...   X   lau   Dog
   ..E......S...A.(K..).IIE.K.EFN...   X   lau   Ferret
   ..ET.......I.E.(K..)HSTE.K.ELN...   X   lau   Panda
   ..E........I.E.(K..)HTIE.K.EFN...   X   lau   Pacific walrus
   ..E..........A.(K..)HTIE.K.EFN...   X   lau   Weddell seal
   ..E............(K..)H.IK....FN...   X   lau   Black flying-fox
   .RE...........K(K..)H.IN....F....   X   lau   David's myotis bat
   .REG.V...S.P..K(K..)H.IN....FT...   X   lau   Big brown bat
   .QEL....F......(KSN)QTIL...EFH...   X   lau   Hedgehog
   ..EA....F......(I..)E.IM.R..FN...   X   lau   Shrew
   .ND...N.F......(K..)..I.....FK...   X   lau   Star-nosed mole
   .LEH...........(K..)LI.E....FN...   X   afr   Elephant
   .REN..N......L.(K.T)..LV.Y..FN...   X   afr   Cape elephant shrew
   .LEH...........(S..)..VE....F....   X   afr   Manatee
   ..EN..........D(K.I)Q.VE....FN...   X   afr   Cape golden mole
   ..EN..........N(K..)...E....FN...   X   afr   Tenrec
   .LEN...........(KS.).LIE.H...N...   X   xen   Armadillo
   ..EG..RF...S...(DKI)H.LK.R...N...   X   mar   Opossum
   ..EE.......ST..(ESI)SDLK.K...N...   X   mar   Tasmanian devil

NO 66
GN ITGAX
ID ITAX_HUMAN
MP 697
DE Integrin alpha-X
CL catarrhines
SQ 58
   DPGRLSPRATFQETK(NRS)LSRVRVLGLKAHC   O   hum   Human
   ...............(...).............   O   hac   Chimp
   ...............(...).............   O   aga   Gorilla
   .........N.....(...).........E...   O   gra   Orangutan
   ...............(...).........ET..   O   ape   Gibbon
   ...............(...)....Q....ETY.   O   cat   Rhesus macaque
   ...............(...)....Q....ETY.   O   cat   Crab-eating macaque
   ...............(...)....Q....ETY.   O   cat   Baboon
   ...............(...)....Q....ETY.   O   cat   Green monkey
   ...............(S..)....Q....QT..   X   sim   Marmoset
   ...............(S..)....Q.....T..   X   sim   Squirrel monkey
   .........I.K..H(AHN).T...D...GK..   X   pri   Bushbaby
   ....P.S..V.K..N(T.T)...A.D...TQY.   X   eua   Treeshrew
   ...H.....V.K..N(T.I).T..KD...NKY.   X   gli   Squirrel
   .........I.K...(T.T).I.DK....NR..   X   gli   Lesser Egyptian jerboa
   .........I.E...(TQA).....I...SK..   X   gli   Prairie vole
   .........I.K...(TQA).T..KT...NKY.   X   gli   Chinese hamster
   .........V.K..N(TQA).T...S...GEY.   X   gli   Golden hamster
   .H....T..I.K...(T.A).T..KT...NK..   X   gli   Mouse
   .........I.K...(TQA).TK..T...SS..   X   gli   Rat
   .........I.K..N(T.T).TQTQ.FR.SRR.   X   gli   Naked mole-rat
   ...........K..N(T.T).T..Q.FE.SR..   X   gli   Guinea pig
   .........I.ET.N(T.T)..QK..FE.GK..   X   gli   Chinchilla
   .....N...V.KK.N(T.T).TQ.Q....RQ..   X   gli   Brush-tailed rat
   .....N...V.K...(T..)...EQE...DPY.   X   gli   Rabbit
   .........V.K..S(LWT).R.QQ.....Q..   X   gli   Pika
   .........I.E..N(TWN).T.......TE..   X   lau   Alpaca
   .........I.E..N(TWN)MT.......TE..   X   lau   Bactrian camel
   .........V.E..G(T.N).T...D...TQ..   X   lau   Dolphin
   ....M....V.E..G(T.N).T...D...TQ..   X   lau   Killer whale
   .........V....G(T.N).T...E....Q..   X   lau   Tibetan antelope
   .........I....R(T.N).T..QE....Q..   X   lau   Sheep
   .........I....R(T.N).T..QE....Q..   X   lau   Domestic goat
   ....MN...I.E...(TWN).T.......NQ..   X   lau   Horse
   .........I.E...(TWN).T........Q..   X   lau   White rhinoceros
   ....QN...I.E...(A.N).TH......RQY.   X   lau   Cat
   ....Q....I.E...(T.N).T..Q....RQY.   X   lau   Dog
   ....Q....I.E...(T.N).T.......SQ..   X   lau   Ferret
   ....QN...I.E...(TWN).T...D...RQ..   X   lau   Panda
   ....Q....I.E...(TWN).T.......RQ..   X   lau   Pacific walrus
   ....Q....I.E...(TWN).T.......GQY.   X   lau   Weddell seal
   .........I.E...(TWN).T.......RQ..   X   lau   Black flying-fox
   ...H.....I.K..N(T.H).........RQ..   X   lau   David's myotis bat
   .........I.K..N(T.H).T...L.D.RQ..   X   lau   Big brown bat
   .........I.K..N(TWN).T..Q....RY..   X   lau   Hedgehog
   .....N...I.E...(T.N).T..Q....GR..   X   lau   Shrew
   .....N...V.EK..(M.N).TL.QD...GQ..   X   lau   Star-nosed mole
   .........I.K...(T.N).TQ.QDI..GQ..   X   afr   Elephant
   .....N...I.D...(TWN).T..QL...GQ..   X   afr   Cape elephant shrew
   .........I.K...(TWN).TV.QD...GQ..   X   afr   Manatee
   .....N...I.D...(T.N).NQ.Q....GQ..   X   afr   Cape golden mole
   .........I.EA.Q(A.N).TQ.Q....GR..   X   afr   Tenrec
   K..C...C.I.S...(TWN).T*.Q..E.GQY.   X   afr   Aardvark
   .....K...I.S..M(T.N).T..Q....SQF.   X   xen   Armadillo
   .........I.D...(..I).K..KT.K.GD..   X   mar   Opossum
   .....K...I.D.K.(K.T).N..KI...GN..   X   mar   Tasmanian devil
   .....N...V.D...(S.I)IK..EI...GKR.   X   mar   Wallaby
   .....N...V.DQ..(..T).RAQQTI..GEN.   O   mon   Platypus

NO 67
GN ITGB2
ID ITB2_HUMAN
MP 212
DE Integrin beta-2
CL African great apes
SQ 55
   ECQPPFAFRHVLKLT(NNS)NQFQTEVGKQLIS   O   hum   Human
   ...............(...)S............   O   hac   Chimp
   ...............(...).............   O   aga   Gorilla
   ...............(D..).............   X   gra   Orangutan
   ...............(...)H...........-   O   ape   Gibbon
   ...A...........(S..)....R........   X   cat   Rhesus macaque
   ...A...........(S..)....R........   X   cat   Crab-eating macaque
   ...A...........(S..)....K........   X   cat   Baboon
   ...A...........(S..)....R........   X   cat   Green monkey
   ...A...........(...).............   O   sim   Marmoset
   ...A...........(...).............   O   sim   Squirrel monkey
   G..A...........(D.A).............   X   pri   Bushbaby
   ...............(D..).............   X   eua   Treeshrew
   Q..............(S..)....A........   X   gli   Squirrel
   A..............(D..).............   X   gli   Lesser Egyptian jerboa
   A..............(D..).............   X   gli   Prairie vole
   A..............(D..).............   X   gli   Chinese hamster
   A..............(D..).............   X   gli   Mouse
   A..............(D..).............   X   gli   Rat
   ...............(...)...R.........   O   gli   Naked mole-rat
   ......S.....R..(D..)...R.........   X   gli   Guinea pig
   ...............(...).............   O   gli   Chinchilla
   ...............(D..)...R.........   X   gli   Brush-tailed rat
   ...A...........(S..)E..R.........   X   gli   Rabbit
   ...A...........(D..).............   X   lau   Pig
   ......T........(D..)....R........   X   lau   Alpaca
   ......T........(D..)....R........   X   lau   Bactrian camel
   ...A...........(D..)S..R.........   X   lau   Dolphin
   ...A...........(D..)S..R.........   X   lau   Killer whale
   ...............(...)K..E.........   O   lau   Tibetan antelope
   ...............(D..)K..E.........   X   lau   Cow
   ...............(D..)K..E.........   X   lau   Sheep
   Q..............(D..)K..E.........   X   lau   Domestic goat
   ...............(D..).............   X   lau   Horse
   ...............(D..)K............   X   lau   White rhinoceros
   ...A...........(...)H............   O   lau   Cat
   ...A...........(...).K...........   O   lau   Dog
   ...............(...)....R........   O   lau   Ferret
   ............R..(...).............   O   lau   Panda
   ...............(...).............   O   lau   Pacific walrus
   ...............(...).............   O   lau   Weddell seal
   ...............(D..).............   X   lau   Black flying-fox
   ...............(D..).............   X   lau   Megabat
   ........K......(D..).............   X   lau   David's myotis bat
   ........K......(D..).............   X   lau   Microbat
   ........K......(D..).............   X   lau   Big brown bat
   ...A...........(D..)....K........   X   afr   Elephant
   Q..A...........(S..)DK...........   X   afr   Cape elephant shrew
   A..............(D..)..........S..   X   afr   Manatee
   D..............(...).............   O   afr   Cape golden mole
   ...A...........(...).............   O   afr   Tenrec
   ...A...........(...)...K.........   O   afr   Aardvark
   Q...........R..(...)....S........   O   mar   Opossum
   Q.......K......(D..).............   X   mar   Tasmanian devil
   Q.......K......(D..)....S........   X   mar   Wallaby

NO 68
GN KCNK18
ID KCNKI_HUMAN
MP 70
DE Potassium channel subfamily K member 18
CL simians
SQ 58
   DGEFEKFLEELCRIL(NCS)ETVVEDRKQDLQG   O   hum   Human
   ...............(...)....Q........   O   hac   Chimp
   ...............(...)....Q........   O   aga   Gorilla
   ...............(...).............   O   gra   Orangutan
   ...............(...).............   O   ape   Gibbon
   ...............(...).............   O   cat   Rhesus macaque
   ...............(...).............   O   cat   Crab-eating macaque
   ...............(...).............   O   cat   Baboon
   ...............(...).............   O   cat   Green monkey
   .....E.........(...).............   O   sim   Marmoset
   .....E.........(...).............   O   sim   Squirrel monkey
   --...E...G..S..(E.N)G.AMRG..EKV.E   X   pri   Bushbaby
   .QG..M...K..S..(E.N)K..M.HS..A.RE   X   eua   Treeshrew
   EEV.KA..H...G..(E.N)G..A.GKT.HVRK   X   gli   Squirrel
   EP.L....T...S..(E.N)R..T.G.DK..LR   X   gli   Lesser Egyptian jerboa
   NP.LK...DK..S..(K.N)R...DGNRKV.CE   X   gli   Prairie vole
   NP.LK...DD..S..(K.N)R.E..GNRKA.CE   X   gli   Chinese hamster
   NPDLKN..DD..S..(E.N)R....GNRKA.CK   X   gli   Golden hamster
   NP.LK...DD..N..(K.N)L....GSRKN.CE   X   gli   Mouse
   NP.LK...DK..N..(K.N)L....GSRK..CE   X   gli   Rat
   NP..Q...S......(.YT)T.AS..E.LEVLK   O   gli   Naked mole-rat
   .PD.QR..N....V.(..T)KAAS..E.LKVL.   O   gli   Guinea pig
   NA..QS..N....V.(..T)..AS.EE.LEVLK   O   gli   Chinchilla
   NP..QI..T....V.(..T).IASK.E..EVLR   O   gli   Brush-tailed rat
   NR...E...T.YG..(D.N)R.A..G..EAVRV   X   gli   Rabbit
   NQ...A...Q..G..(E.N)T..AKG.QAAVRT   X   gli   Pika
   .P...V...K..GF.(G.N)T..E.G......E   X   lau   Pig
   .P...E...K..D..(K.N)R..E.S..R..GK   X   lau   Alpaca
   .P...E...K..D..(K.N)R..E.S..R..GK   X   lau   Bactrian camel
   .P...E...K..D..(K.N)R..E.G..RG.GK   X   lau   Dolphin
   .P...E...K..D..(K.N)R..E.G..RG.GK   X   lau   Killer whale
   .P...E..KK..D..(K.N)S..E.G..R..EK   X   lau   Tibetan antelope
   .P...E...K..G..(K.N)S..E.G..R..EK   X   lau   Cow
   .P...E..KK..D..(K.N)SS.E.G..R..EK   X   lau   Sheep
   .P...E..KK..D..(K.N)S..E.G..R..EK   X   lau   Domestic goat
   ..GL.E...K..G..(K.N)R..E.G.....GK   X   lau   Horse
   .R...E...K..S..(K.N)R..E..G.R..RK   X   lau   White rhinoceros
   .P...E..G...G..(K.N)R..E.G.....RA   X   lau   Cat
   .PD..E......G..(K.N)R..E.G.....GT   X   lau   Dog
   .PD..E..V...G..(K.N)R..E.G..R..GV   X   lau   Ferret
   .P...E......G..(K.N)R..E.G..R..GA   X   lau   Panda
   .P...E..V...G.S(K.N)R..E.G.....GA   X   lau   Pacific walrus
   .P...E..V...G..(K.N)R..E.G..R..GA   X   lau   Weddell seal
   .Q...E..GK..ST.(T.N)R..M.GK.R..GE   X   lau   Black flying-fox
   .Q...E..GK..ST.(T.N)R..M.GK....RE   X   lau   Megabat
   .P...D...Q..GL.(K.N)R.GM....M..RQ   X   lau   David's myotis bat
   .P...D...Q..GL.(K.N)R.GM....M..RQ   X   lau   Microbat
   .P...D...K..DL.(E.N)R.GMK...LG.RQ   X   lau   Big brown bat
   .P...T...K.RGV.(G.N)R..E.....E.GE   X   lau   Hedgehog
   .P...D.....RGL.(R.N)G.--DGW..K.GN   X   lau   Shrew
   .L..KA..KN..S..(K.N)K..E.......GL   X   lau   Star-nosed mole
   .R...A...KIGNV.(K.N)G.AEGS...S.WS   X   afr   Elephant
   .Q...F..QN.ST..(G.N)K..E.GP..R.RE   X   afr   Cape elephant shrew
   .*...E...K..N..(K..)R..E.S...S.WR   X   afr   Manatee
   .Q...R...N..N..(K.N)R..EASS.LN.RK   X   afr   Cape golden mole
   .Q...E..KA..N..(Q.N)R..E.S.RRA.RV   X   afr   Tenrec
   GQK..E...K..D..(K.N)R..E.GS..T.W-   X   afr   Aardvark
   QQ...E..AN..S..(K.N)G..EDVE..SVRE   X   xen   Armadillo

NO 69
GN LGALS3BP
ID LG3BP_HUMAN
MP 192
DE Galectin-3-binding protein
CL simians
SQ 54
   TANLEAQALWKEPGS(NVT)MSVD--AECVPMV   O   hum   Human
   ...............(...).............   O   hac   Chimp
   ...............(...).............   O   aga   Gorilla
   ...P...........(...)...H.........   O   gra   Orangutan
   ...............(...).............   O   ape   Gibbon
   ...............(...).............   O   cat   Rhesus macaque
   ...............(...).............   O   cat   Crab-eating macaque
   ...P...........(...).G...........   O   cat   Baboon
   ...P...........(...)......V......   O   cat   Green monkey
   ..........E....(...).H...........   O   sim   Marmoset
   ...............(...).........M...   O   sim   Squirrel monkey
   .T.P.......A.SK(S.D)...........L.   X   pri   Bushbaby
   S..P.......K...(K..).Y.........VI   X   eua   Treeshrew
   ...P.....SGA...(.L.)......S..M.V.   O   gli   Squirrel
   NT.P......EK...(G.V).R.......LHV.   X   gli   Lesser Egyptian jerboa
   NT.P.....QQAV.R(S.L).R.......M.V.   X   gli   Prairie vole
   NT.S......HAE..(S.I).R....VD.M.V.   X   gli   Chinese hamster
   NT.P......QVV..(S.I).R.......M.V.   X   gli   Golden hamster
   RT.P......QVV..(S.I).R.......M.V.   X   gli   Mouse
   RT.P......QVV..(S.I).R.......M.V.   X   gli   Rat
   ...P......E....(SMV).R....S..MFVA   X   gli   Naked mole-rat
   R..P..E...EK...(SII).R....S...FVA   X   gli   Guinea pig
   ...P......E....(KII).R....S..MSV.   X   gli   Chinchilla
   ...P..E...EK...(KII).R....S..MFV.   X   gli   Brush-tailed rat
   A..P.....QQ....(...).N....S..M.Q.   O   gli   Pika
   ST.P..RS.......(R..).E.....G.M.V.   X   lau   Alpaca
   ST.P..RS.......(R..).E.....G...V.   X   lau   Bactrian camel
   ST.P..HG.....S.(R..).E....T....V.   X   lau   Dolphin
   ST.P..HG.....S.(R..).E....T....V.   X   lau   Killer whale
   ST.P..HG.......(R..).E.........T.   X   lau   Tibetan antelope
   ST.P..HG.......(R..).E.........V.   X   lau   Cow
   ST.P..HG.......(R..).E.........T.   X   lau   Sheep
   ST.P..HG.......(R..).E.........T.   X   lau   Domestic goat
   ST.P.........D.(T..).EL........V.   X   lau   Horse
   S..P.....LR....(A..).E.........V.   X   lau   White rhinoceros
   ST.P..E....G..P(T..).E.......L.V.   X   lau   Cat
   A..P.....C.A...(T..).E.......L.V.   X   lau   Dog
   SS.P..........R(T..).E.......L.V.   X   lau   Ferret
   ST.P..........P(T..).E.......L.V.   X   lau   Panda
   S..P..........A(T..).E.......L.V.   X   lau   Pacific walrus
   ST.P..........P(T..).E.......L.V.   X   lau   Weddell seal
   ST.P......R....(T..)LE.......L.L.   X   lau   Black flying-fox
   ST.P......R....(T..)LE.......L.L.   X   lau   Megabat
   .S.P......S....(T..).E.......L.V.   X   lau   David's myotis bat
   .S.P......T....(T..).E.......L.V.   X   lau   Microbat
   .S.P......T....(S..).E.......L.V.   X   lau   Big brown bat
   S..P.....LRG..N(VI.)LQ....S..M.FI   X   lau   Hedgehog
   AT.P.....LR.SDG(T..)LE....P..L.V.   X   lau   Shrew
   ST.P.....LRG..G(T..).E.E.......V.   X   afr   Cape elephant shrew
   ST.P..RD.LR.S..(...).E.........V.   O   afr   Manatee
   SS.P...V.LRGT..(S..)IA....T..L.V.   X   afr   Cape golden mole
   SS.P...T.L.D.S.(SII)IE....T..L.V.   X   afr   Tenrec
   ST.P.....LR...H(SI.).E.........V.   X   afr   Aardvark
   VVTP*T.S.R.P.R.(QIS).ATEPK.DLH.RP   X   mar   Opossum

NO 70
GN LHB
ID LSHB_HUMAN
MP 50
DE Lutropin subunit beta
CL simians
SQ 58
   ILAVEKEGCPVCITV(NTT)ICAGYCPTMMRVL   O   hum   Human
   T..............(...).............   O   hac   Chimp
   T..............(...).........T...   O   aga   Gorilla
   T...........V..(...).........T...   O   gra   Orangutan
   T..............(...)......A......   O   ape   Gibbon
   T..A...A.......(...).............   O   cat   Rhesus macaque
   T..A...A.......(..S).............   O   cat   Crab-eating macaque
   T..A...A....V..(...).............   O   cat   Baboon
   T..A...A.......(...).............   O   cat   Green monkey
   ...A........VAF(...)......SS.V...   O   sim   Marmoset
   ............VPF(...)......SS.V..M   O   sim   Squirrel monkey
   T..A.N.A......F(T.S).......S.V...   X   pri   Bushbaby
   T.TA.N.A......F(T.S).......S.V...   X   eua   Treeshrew
   T..A.H.A......F(T.S).......S.V...   X   gli   Squirrel
   T..A.N.A.......(T.S).......S.V...   X   gli   Lesser Egyptian jerboa
   T..A...A......F(T.S).......S.E..V   X   gli   Prairie vole
   T..A.N.V......F(T.S).......S.V...   X   gli   Chinese hamster
   T..A.NDV......F(T.S).......S.V...   X   gli   Golden hamster
   T..A.N.F......F(T.S).......S.V...   X   gli   Mouse
   T..A.N.F......F(T.S).......S.V...   X   gli   Rat
   T..A.N.A....V.F(T.S).......S.....   X   gli   Naked mole-rat
   T..A...A..I.V.F(T.S).......S.....   X   gli   Guinea pig
   T..A.N.A....V.F(T.S).......S.....   X   gli   Chinchilla
   T..A...A....M.F(T.S).......S.V...   X   gli   Brush-tailed rat
   T..A.N.A......F(T.S).......S.V...   X   gli   Rabbit
   T..A...A.......(T.S).......S.V...   X   gli   Pika
   T..A.N.A......F(T.S).......S.V...   X   lau   Pig
   T..A.N.A......F(T.S).......S.V...   X   lau   Alpaca
   T..A.N.A......F(T.S).......S.V...   X   lau   Bactrian camel
   T..A.N.A......I(T.S).......S.V...   X   lau   Dolphin
   T..A.N.A......F(T.S).......S.V...   X   lau   Killer whale
   T..A...A......F(T.S).......S.K...   X   lau   Tibetan antelope
   T..A...A......F(T.S).......S.K...   X   lau   Cow
   T..A...A......F(T.S).......S.K...   X   lau   Sheep
   T..A...A......F(T.S).......S.K...   X   lau   Domestic goat
   T..A...A..I...F(T.S).......S.V..M   X   lau   Horse
   T..A.N.A......F(T.S).......S.V...   X   lau   White rhinoceros
   T..A.N.A....V.F(T..).......S.....   X   lau   Cat
   T..A.N.A......F(T..).......S.V...   X   lau   Dog
   T..A.N.A....V.F(T.S).......S.V...   X   lau   Ferret
   T..A.N.A......F(T..).......S.V...   X   lau   Panda
   T..A.N.A......F(T..).......SLV...   X   lau   Pacific walrus
   T..A.N.A......F(T..).......SLV...   X   lau   Weddell seal
   T..A.N.A....V.F(T.S).......S.V...   X   lau   Black flying-fox
   T..A.N.A....V.F(T.S).......S.V...   X   lau   Megabat
   T..A.N.A......F(T.S).......S.V...   X   lau   Big brown bat
   T..A.S.A..F...F(T.S).......S.V...   X   lau   Hedgehog
   T..A.N.A..F..AF(T.S).......S.V...   X   lau   Shrew
   T..A.N.A......F(T.S)......SSK....   X   lau   Star-nosed mole
   T.TA...A......F(T.S)....C----V...   X   afr   Elephant
   T.GI.....S....L(T..).......S.T...   X   afr   Cape elephant shrew
   T..A...A......F(T.S).......S.V...   X   afr   Manatee
   T.VA.N.A......F(T.S).......S.V...   X   afr   Cape golden mole
   T.VA.N.A..F...F(T.S).......S.V...   X   afr   Tenrec
   T..A.N.A......F(T.R).......S.V...   X   afr   Aardvark
   T..A.N.A....V.F(T..).......S.V...   X   xen   Armadillo
   T..A.SDD....V.F(T..).......S.V...   X   mar   Tasmanian devil
   T..A.SDA....V.F(T..).......S.V...   X   mar   Wallaby

NO 71
GN MEP1B
ID MEP1B_HUMAN
MP 370
DE Meprin A subunit beta
CL catarrhines
SQ 59
   LNIYIREYSADNVDG(NLT)LVEEIKEIPTGSW   O   hum   Human
   ...............(...).............   O   hac   Chimp
   ...............(...).............   O   gra   Orangutan
   ...............(...).............   O   ape   Gibbon
   .........T.....(...).........I...   O   cat   Rhesus macaque
   .........T.....(...).........I...   O   cat   Crab-eating macaque
   .........T.....(...).........I...   O   cat   Baboon
   .........T..M..(...).........I...   O   cat   Green monkey
   ....T..........(..I).........I...   X   sim   Marmoset
   .............NS(..I).............   X   sim   Squirrel monkey
   ...........S.NS(S..)......G..S.N.   X   pri   Bushbaby
   ..........G..N.(S..)......G..I.N.   X   eua   Treeshrew
   ....V.N.T..D.S.(HR.)..KQ.....I.I.   X   gli   Squirrel
   ......K...GKLE.(...)..K......I.N.   O   gli   Lesser Egyptian jerboa
   VK..T...T.G.PN.(V..).QR..QD..M...   X   gli   Prairie vole
   V...T.V.T.G.P..(V..).QR........R.   X   gli   Chinese hamster
   V...T.V.T.S.PE.(V..).QR......I...   X   gli   Golden hamster
   ....T...TTGQQG.(V..).QRQ...V.I...   X   gli   Mouse
   ..V.T...T.GHQ..(V..).QR..RD......   X   gli   Rat
   ....V....VNDL..(K..)..K..........   X   gli   Naked mole-rat
   ....V...AVNDTN.(K..).........M...   X   gli   Guinea pig
   ....V....DNDAH.(K..).........I...   X   gli   Chinchilla
   ....V....DND.R.(K..).........I...   X   gli   Brush-tailed rat
   ....V...P.G....(S..)....L....V...   X   gli   Rabbit
   ....V....TG..N.(S..).............   X   gli   Pika
   .D.......G...N.(I.I)......D..L...   X   lau   Pig
   ...FV.....AS.N.(T..)......D..I...   X   lau   Alpaca
   ....V.....AS.N.(T..)......D..I...   X   lau   Bactrian camel
   ....V.....AS.NR(T..)......DV.I...   X   lau   Dolphin
   ....V.....AS.NR(T..)......DV.I...   X   lau   Killer whale
   .........VAHLSR(T.S)...Q..D..S...   X   lau   Tibetan antelope
   .........TAHLSR(T..)...Q..D..S...   X   lau   Cow
   ..........AHPSR(TVS)...Q..D..S...   X   lau   Sheep
   ..........AHPSR(T.S)...Q..D..S...   X   lau   Domestic goat
   ...........D...(S..)......D..I...   X   lau   White rhinoceros
   .............N.(T..)......D..I...   X   lau   Cat
   ....T...T..H.N.(T..)......D..I...   X   lau   Dog
   ....T......H.T.(T..)..K......L...   X   lau   Ferret
   ..V.T........A.(T..).............   X   lau   Panda
   ....T...P.G..V.(T.M).........I...   X   lau   Pacific walrus
   ....T...P....V.(T..).........I...   X   lau   Weddell seal
   ....V........N.(T..).L....D..I.N.   X   lau   Black flying-fox
   ....V........N.(T..).L....D..I.N.   X   lau   Megabat
   ..........GAPN.(TVV)......D......   X   lau   David's myotis bat
   ..........GAPN.(TVV)......D......   X   lau   Microbat
   ..........GSEN.(TV.).....RD......   X   lau   Big brown bat
   ..........SH.N.(S..)..K...D..I.R.   X   lau   Hedgehog
   ..........AH.N.(S.I)..K...D..M.T.   X   lau   Shrew
   MK........S..N.(I..)...T..D..V...   X   lau   Star-nosed mole
   ..........GS.N.(T..).........I...   X   afr   Elephant
   ...FV...TTE..N.(T..)...Q..D......   X   afr   Cape elephant shrew
   ..........G..N.(T..).........I...   X   afr   Manatee
   ....V.....G..N.(T.I)..Q..........   X   afr   Cape golden mole
   .AL.V.....GH.N.(T.A)..K..R.......   X   afr   Tenrec
   .....K....G..N.(S..).............   X   xen   Armadillo
   .K......TSV..N.(S.S)..H.V.DV...I.   X   mar   Opossum
   .K..V.K.TSE.DN.(S..)..D.V.DVSI.T.   X   mar   Tasmanian devil
   .K......VDNYDNN(L.K)..D.V.DQTI...   X   mar   Wallaby
   .K.W.....S.H.N.(T.N).....IGA.EDT.   X   mon   Platypus

NO 72
GN MFGE8
ID MFGM_HUMAN
MP 238
DE Lactadherin
CL simians
SQ 59
   LGCELNGCANPLGLK(NNS)IPDKQITASSSYK   O   hum   Human
   ...............(...).............   O   hac   Chimp
   ...............(...).............   O   aga   Gorilla
   .........S.-...(.SI)--...........   X   gra   Orangutan
   ........T......(...).............   O   ape   Gibbon
   .....D..F......(...).............   O   cat   Rhesus macaque
   .....D..F......(...).............   O   cat   Crab-eating macaque
   .....D..F......(...).............   O   cat   Baboon
   .....D..F......(...).............   O   cat   Green monkey
   ........HH.....(...).............   O   sim   Marmoset
   ........HH.....(...)............R   O   sim   Squirrel monkey
   ........SE.....(D.I).L...VK...T..   X   pri   Bushbaby
   ........SE.....(DRI)..........Y..   X   eua   Treeshrew
   ........SE.....(D.T)...........F.   X   gli   Squirrel
   ....IH..SE.....(D.R)...RR.....T..   X   gli   Lesser Egyptian jerboa
   .....H..SE.....(..T)...S.........   O   gli   Prairie vole
   ........SE.....(...)...SHM....T..   O   gli   Chinese hamster
   ........SE.....(...)...N.M.......   O   gli   Golden hamster
   .....H..SE.....(..T)...S.MS......   O   gli   Mouse
   .....H..SE.....(..T)...S.........   O   gli   Rat
   V.......SE.....(DGT)...R.........   X   gli   Naked mole-rat
   ........SE.....(DG.)..NR.........   X   gli   Guinea pig
   ........SE.....(DHK)...R.........   X   gli   Chinchilla
   ........SE.....(DHT)...R.........   X   gli   Brush-tailed rat
   ........SE.....(D.T).............   X   gli   Rabbit
   .....S...E.....(D.T)..N.......F.R   X   lau   Pig
   .........E.....(DST)..........F..   X   lau   Alpaca
   .........E.....(DST)..........F..   X   lau   Bactrian camel
   .........E.....(..T)..S.......F..   O   lau   Dolphin
   .........E.....(D.T)..S.......F..   X   lau   Killer whale
   .....D..TE.....(D.T)..N.......Y..   X   lau   Tibetan antelope
   ........TE.....(D.T)..N.......Y..   X   lau   Cow
   .....D..TE.....(H.T)..........Y..   X   lau   Sheep
   .....D..TE.....(D.T)..N.......Y..   X   lau   Domestic goat
   ....V....E....E(D..)...R......T.R   X   lau   Horse
   ....V....E.....(D..)..........T.R   X   lau   White rhinoceros
   .........E...M.(D.T)...R......I.R   X   lau   Cat
   .........E...M.(D.T)..........I.R   X   lau   Dog
   ....V....E...M.(D..)...R......I.R   X   lau   Ferret
   .........E...M.(DGT)....R.....I.R   X   lau   Panda
   .........E...M.(D..).....V....I.R   X   lau   Pacific walrus
   .........E...M.(D..)..........I.R   X   lau   Weddell seal
   .........G.....(D.T)..........T..   X   lau   Black flying-fox
   .........G.....(D.T)..........T..   X   lau   Megabat
   ....V...DE...M.(.RT)..........T.R   O   lau   David's myotis bat
   ........SE...M.(D.I).L........F.R   X   lau   Hedgehog
   ........SD...M.(D.T)..........VFR   X   lau   Shrew
   -------..E...MR(D.V)....R.....T..   X   lau   Star-nosed mole
   ........SE.....(D.T)..........MF.   X   afr   Elephant
   .....S..SE...M.(D.T)..........F.N   X   afr   Cape elephant shrew
   ........SD.....(..T).R.Q......VF.   O   afr   Manatee
   ........SE.....(D..).L........YF.   X   afr   Cape golden mole
   ........S.S..M.(DGT).L.R..S...FF.   X   afr   Tenrec
   ........SE.....(D.T).L........IF.   X   afr   Aardvark
   .....H..SA.....(D.R)...RY.....TFR   X   xen   Armadillo
   Y...VE..SS...M.(SMA)....K.....TF.   X   mar   Opossum
   F...VE..SE...M.(SEI)...SK.....TF.   X   mar   Tasmanian devil
   -------.SE...M.(SEI)...SR.....TF.   X   mar   Wallaby
   -------.SE...M.(SHL).R........T..   X   mon   Platypus

NO 73
GN MGAM
ID MGA_HUMAN
MP 295
DE Maltase-glucoamylase, intestinal
CL simians
SQ 61
   WKTWPIFNRDTTPNG(NGT)NLYGAQTFFLCLE   O   hum   Human
   ...............(...).............   O   hac   Chimp
   ...............(...).............   O   aga   Gorilla
   ...............(...).............   O   gra   Orangutan
   ...............(...).............   O   ape   Gibbon
   ...............(...).............   O   cat   Rhesus macaque
   ...............(...).............   O   cat   Crab-eating macaque
   ...............(...).............   O   cat   Baboon
   ...............(...).............   O   cat   Green monkey
   ...............(...).............   O   sim   Marmoset
   ...............(...).............   O   sim   Squirrel monkey
   ..............A(D..)....T........   X   pri   Bushbaby
   ...............(D..).............   X   eua   Treeshrew
   .......T......K(D.N).............   X   gli   Squirrel
   .......T......E(D..).............   X   gli   Lesser Egyptian jerboa
   ....AL.A...F..A(..H)....V........   X   gli   Prairie vole
   .....L.S......K(D.N)....V........   X   gli   Chinese hamster
   ....SL.S...V..K(D.H)....V........   X   gli   Golden hamster
   .....M.S......E(D..)....V........   X   gli   Mouse
   .....M.A......E(D.N)....V........   X   gli   Rat
   .......T...I..R(D..).............   X   gli   Naked mole-rat
   .R.....T...I..A(D..)......P......   X   gli   Guinea pig
   .......T...A...(D..).............   X   gli   Chinchilla
   .R.....T.......(A..).............   X   gli   Brush-tailed rat
   .......T.....SE(D..).............   X   gli   Rabbit
   .......T...A..E(I.S).............   X   gli   Pika
   .R.....T..A..TQ(GMI).....HP......   X   lau   Pig
   .R.....A...A.T.(D..).............   X   lau   Alpaca
   .R.....A...A.T.(D..).............   X   lau   Bactrian camel
   .......A...I.K.(D..).............   X   lau   Dolphin
   .......A...I.K.(D..)...S.........   X   lau   Killer whale
   .......A.......(D..).............   X   lau   Tibetan antelope
   .......A.......(D..).............   X   lau   Cow
   .......A.......(D..).............   X   lau   Sheep
   .......A.......(D..).............   X   lau   Domestic goat
   ...............(D..).............   X   lau   Horse
   .......A.......(D..)....S........   X   lau   White rhinoceros
   ....SM.A...I...(D..)....T........   X   lau   Cat
   .......A.......(D..).............   X   lau   Dog
   ....AM.A.......(D..)....T........   X   lau   Ferret
   ....SM.A.......(D..)....T........   X   lau   Panda
   ....SM.A...A...(D..)....T........   X   lau   Pacific walrus
   ....SL.A.....S.(D..)....T........   X   lau   Weddell seal
   ....S..A.......(D..)....T........   X   lau   Black flying-fox
   ....S..A.......(D..)....T........   X   lau   Megabat
   .......A.......(D.N).............   X   lau   David's myotis bat
   .......A.......(D.N).............   X   lau   Microbat
   .......A.......(D.N).............   X   lau   Big brown bat
   ---------------(D..)....T........   X   lau   Hedgehog
   ....TL.S...V..R(DR.)....V........   X   lau   Shrew
   .......T.....T.(D.N).............   X   lau   Star-nosed mole
   .......A..AF..E(DVK)....T........   X   afr   Elephant
   ....S..A.......(D..).............   X   afr   Cape elephant shrew
   .......A...I.TE(DAK)....S........   X   afr   Manatee
   .......A.......(D..).............   X   afr   Cape golden mole
   .......T.......(D..).............   X   afr   Tenrec
   .......A......R(D..).............   X   afr   Aardvark
   .......S.......(D..)....V........   X   xen   Armadillo
   .......S......E(DM.).............   X   mar   Opossum
   .......S..N...D(GM.).............   X   mar   Tasmanian devil
   ..I....S..N...D(GM.).............   X   mar   Wallaby

NO 74
GN MGAM
ID MGA_HUMAN
MP 827
DE Maltase-glucoamylase, intestinal
CL euarchonts
SQ 60
   GLHLRGGYIFPTQQP(NTT)TLASRKNPLGLII   O   hum   Human
   ...............(...).............   O   hac   Chimp
   ...............(...)...----------   O   aga   Gorilla
   ...............(...).............   O   gra   Orangutan
   ...............(...).............   O   ape   Gibbon
   ...............(...).............   O   cat   Rhesus macaque
   ...............(...).............   O   cat   Crab-eating macaque
   ...............(...).............   O   cat   Baboon
   ...............(...).............   O   cat   Green monkey
   ...............(...).............   O   sim   Marmoset
   ...............(...).............   O   sim   Squirrel monkey
   ...............(...).E...........   O   pri   Bushbaby
   ...............(...).M.----------   O   eua   Treeshrew
   ...............(S..).M...Q.......   X   gli   Squirrel
   ........V......(S..).T..........V   X   gli   Lesser Egyptian jerboa
   ...............(S..).T.........L.   X   gli   Prairie vole
   ...............(D..).AD..........   X   gli   Chinese hamster
   ...............(D..).T...........   X   gli   Golden hamster
   ...............(A..).E..........V   X   gli   Mouse
   ...............(A..).E...........   X   gli   Rat
   ...............(S..).M...R.......   X   gli   Naked mole-rat
   ...........I...(S..).V...Q.......   X   gli   Guinea pig
   ...............(S..).M...Q.......   X   gli   Chinchilla
   ...............(S..).K....R....LV   X   gli   Brush-tailed rat
   ...............(D..).T...L.A.....   X   gli   Pika
   ...............(...).E...R.S.....   O   lau   Pig
   ...............(A..).E...R.......   X   lau   Alpaca
   ...............(A..).E...R.......   X   lau   Bactrian camel
   .........L.....(A..).V...R.....T.   X   lau   Dolphin
   ...............(A..).V...R.......   X   lau   Killer whale
   ...............(A..).V...Q.......   X   lau   Tibetan antelope
   ...............(A..).V...R.......   X   lau   Cow
   ...............(A..).V...R.......   X   lau   Sheep
   ...............(A..).V...R.......   X   lau   Domestic goat
   ...............(A..).V...R.......   X   lau   Horse
   ...............(A..).V...Q.......   X   lau   White rhinoceros
   .......HV......(A..).VV..R.......   X   lau   Cat
   .......H.......(A..).V...R.......   X   lau   Dog
   .......H.......(A..).V...R.......   X   lau   Ferret
   .......H.......(A..).V...Q.......   X   lau   Panda
   .......H.......(A..).A...R.......   X   lau   Pacific walrus
   .......H.......(A..).V...R.......   X   lau   Weddell seal
   ...............(AI.).A...R.......   X   lau   Black flying-fox
   ...............(A..).A...R.......   X   lau   Megabat
   ...............(A..).V...R.......   X   lau   David's myotis bat
   ...............(A..).V...R.......   X   lau   Microbat
   ...........I...(A..).V...R.......   X   lau   Big brown bat
   ...F.......I.E.(AM.).V...R.......   X   lau   Hedgehog
   .............E.(A..).V...R...A...   X   lau   Star-nosed mole
   .............E.(S..).E...R.......   X   afr   Elephant
   .......H.......(S..).V...Q.......   X   afr   Cape elephant shrew
   ...............(S..).E...R.......   X   afr   Manatee
   ........V......(...).D...R.......   O   afr   Cape golden mole
   .......F...I...(S..).A...R.......   X   afr   Tenrec
   ...............(S..).E...R.......   X   afr   Aardvark
   ...............(A..).A...R.......   X   xen   Armadillo
   ...........I...(AI.).V...........   X   mar   Opossum
   ...............(AI.).V...........   X   mar   Tasmanian devil
   ...............(AI.).V...Q.......   X   mar   Wallaby
   .......H.L...K.(A..).TT..TK......   X   mon   Platypus

NO 75
GN MMRN1
ID MMRN1_HUMAN
MP 114
DE Multimerin-1
CL simians
SQ 58
   TLTSTEKAEGVVKLQ(NLT)LPTNASIKFNPGA   O   hum   Human
   ...............(...).............   O   hac   Chimp
   ...............(...).............   O   aga   Gorilla
   .F.............(...).............   O   gra   Orangutan
   .........R...S.(...).............   O   ape   Gibbon
   .P....R........(...)F..K.N.E.....   O   cat   Rhesus macaque
   .P....R........(...)...K.N.E.....   O   cat   Crab-eating macaque
   .P....R........(...)...K.N.E..S..   O   cat   Baboon
   .P....R--......(...)...K.N.E.....   O   cat   Green monkey
   .........EA..F.(...)...KS.T......   O   sim   Marmoset
   .....G.......F.(...)...KP........   O   sim   Squirrel monkey
   .V.P.R.E......R(S.A)....STV..S..E   X   pri   Bushbaby
   N..P...T.---ERK(K.A)FS.PPT..L...P   X   eua   Treeshrew
   ...P.G.T.E..G..(...)...EP...VS...   O   gli   Squirrel
   ..KLEG.T..G.QF.(TPA)...QST..RSS..   X   gli   Lesser Egyptian jerboa
   .P.PL..T.A.MAPL(P.A).QSKG-----...   X   gli   Prairie vole
   ...PL..TGA.MGPL(P..).QSKGI.QPS...   X   gli   Chinese hamster
   .P.PL.TT.A.RTPL(P.A)HQSKGI.QPS...   X   gli   Golden hamster
   --..L..TGTA.VSL(P.S).QDKP...PST..   X   gli   Mouse
   .P.P...TGTALASL(P.S).QGKP...PSS..   X   gli   Rat
   .P.A.G.TKE.....(K.A)F..KPT.NIK...   X   gli   Naked mole-rat
   .A.A.G.T..E.ESP(K.A)F.NR.NAS.K.E.   X   gli   Guinea pig
   MAPA.G.T..E...P(R.A)I..K..NS.K...   X   gli   Chinchilla
   LAPGAR.T..E...P(E.A)F..Q...SSQA..   X   gli   Brush-tailed rat
   .PAP..MT.EG..S.(.PA).R.QT.T......   X   gli   Rabbit
   .PVP..AIKE-....(.PS).QKQL.A..K...   X   gli   Pika
   M..P..ET.M.L...(T.D)...QS.TQ.S.K.   X   lau   Pig
   ...P...P.R.L...(TPA).LISS..T.S.K.   X   lau   Alpaca
   ..AP...P.R.L...(TPA).LISS....S.K.   X   lau   Bactrian camel
   ...P.G.T.V.L...(T.A)...KS..R.S.K.   X   lau   Dolphin
   ...P.G.T.V.L...(T.A)...KS.TR.S.K.   X   lau   Killer whale
   I..P...T..ML..R(T.A).Q.ESTL..S.K.   X   lau   Tibetan antelope
   ...L...T..ML...(T.A).Q.EPTL..S.K.   X   lau   Cow
   ...P...T..MLN.R(T.A).Q.ESTL..S.K.   X   lau   Sheep
   ...P...T..ML...(T.A).Q.ESTL..S.K.   X   lau   Domestic goat
   ...P..ET..PL.S.(TPA).LPKS..Q.S.R.   X   lau   Horse
   .R....TT...L..H(TPA).R.KS...SS...   X   lau   White rhinoceros
   ...P.G.T...L...(T.A)...KP.L..S.R.   X   lau   Cat
   .....G.T..LL...(TPA)..V.P.LQL..R.   X   lau   Dog
   ...PP..T..AL...(P.A)...KP.L..S.R.   X   lau   Ferret
   ...PPG.T...L...(P.A)...KL.L..S.R.   X   lau   Panda
   ...PPG.I...L..P(P.A)...KP.L..S.R.   X   lau   Pacific walrus
   ...PPG.I...L...(P.A)...KP.L..S.R.   X   lau   Weddell seal
   S......T..EL.F.(T.A)...ES....S.R.   X   lau   Black flying-fox
   S......T..EL.F.(T.A)...ES....S.R.   X   lau   Megabat
   A.....EPGR.L...(T.A)PL.QS.S..S...   X   lau   David's myotis bat
   A.....EP.R.L...(T.A)PL.KS.S..S...   X   lau   Microbat
   A.----ET.REL...(T.A)PLARSGVG.G...   X   lau   Big brown bat
   .FSPP.RTQR.LG.A(T.S)PLGEPPTQPS.R.   X   lau   Hedgehog
   .V.P...T.EMLT..(AFA)..SKP....S.K.   X   lau   Star-nosed mole
   .V........L..S.(T.A)...QS.S.VS..E   X   afr   Elephant
   ....KAN..--..FH(T.A).LS.S.TRL....   X   afr   Cape elephant shrew
   .PA........L.S.(T.A)...QPN.R.S..E   X   afr   Manatee
   ....K.....A..S.(...)...KSN...S..E   O   afr   Cape golden mole
   ....K..T..MIRS.(KIV)....PGL.IS...   X   afr   Tenrec
   ....K..........(S.A)...ES..R.S...   X   afr   Aardvark
   .PLF......MM..E(T.A)F..KS.L..SS..   X   xen   Armadillo
   ..PPS..VA.ML...(PFL)P..QSRLEPS.A.   X   mon   Platypus

NO 76
GN MMRN1
ID MMRN1_HUMAN
MP 120
DE Multimerin-1
CL apes
SQ 57
   KAEGVVKLQNLTLPT(NAS)IKFNPGAESVVLS   O   hum   Human
   ...............(...).............   O   hac   Chimp
   ...............(...).............   O   aga   Gorilla
   ...............(...).............   O   gra   Orangutan
   ...R...S.......(...).............   O   ape   Gibbon
   R...........F..(K.N).E......P....   X   cat   Rhesus macaque
   R..............(K.N).E......P....   X   cat   Crab-eating macaque
   R..............(K.N).E..S...P....   X   cat   Baboon
   R--............(K.N).E......P....   X   cat   Green monkey
   ...EA..F.......(KS.)T........A...   X   sim   Marmoset
   .......F.......(KP.).........A...   X   sim   Squirrel monkey
   .E......RS.A...(.ST)V..S..E......   O   pri   Bushbaby
   .T.---ERKK.AFS.(PPT)..L...PK.....   X   eua   Treeshrew
   .T.E..G........(EP.)..VS....M....   X   gli   Squirrel
   .T..G.QF.TPA...(QST)..RSS...A....   X   gli   Lesser Egyptian jerboa
   .TGA.MGPLP...QS(KGI).QPS....R...A   X   gli   Chinese hamster
   TT.A.RTPLP.AHQS(KGI).QPS....R...A   X   gli   Golden hamster
   .TGTA.VSLP.S.QD(KP.)..PST..GT.M.A   X   gli   Mouse
   .TGTALASLP.S.QG(KP.)..PSS...T.M.A   X   gli   Rat
   .TKE.....K.AF..(KPT).NIK....T....   X   gli   Naked mole-rat
   .T..E.ESPK.AF.N(R.N)AS.K.E..T.E..   X   gli   Guinea pig
   .T..E...PR.AI..(K..)NS.K....AA...   X   gli   Chinchilla
   .T..E...PE.AF..(Q..).SSQA...T.L..   X   gli   Brush-tailed rat
   MT.EG..S..PA.R.(QT.)T......K.....   X   gli   Rabbit
   AIKE-.....PS.QK(QL.)A..K......A..   X   gli   Pika
   ET.M.L...T.D...(QS.)TQ.S.K.......   X   lau   Pig
   .P.R.L...TPA.LI(SS.).T.S.K......P   X   lau   Alpaca
   .P.R.L...TPA.LI(SS.)...S.K......P   X   lau   Bactrian camel
   .T.V.L...T.A...(KS.).R.S.K.......   X   lau   Dolphin
   .T.V.L...T.A...(KS.)TR.S.K.......   X   lau   Killer whale
   .T..ML..RT.A.Q.(EST)L..S.K....I..   X   lau   Tibetan antelope
   .T..ML...T.A.Q.(EPT)L..S.K....I..   X   lau   Cow
   .T..MLN.RT.A.Q.(EST)L..S.K....I..   X   lau   Sheep
   .T..ML...T.A.Q.(EST)L..S.K....I..   X   lau   Domestic goat
   ET..PL.S.TPA.LP(KS.).Q.S.R.A.A..P   X   lau   Horse
   TT...L..HTPA.R.(KS.)..SS.........   X   lau   White rhinoceros
   .T...L...T.A...(KP.)L..S.R.......   X   lau   Cat
   .T..LL...TPA..V(.P.)LQL..R......T   X   lau   Dog
   .T..AL...P.A...(KP.)L..S.R.......   X   lau   Ferret
   .T...L...P.A...(KL.)L..S.R.......   X   lau   Panda
   .I...L..PP.A...(KP.)L..S.R.......   X   lau   Pacific walrus
   .I...L...P.A...(KP.)L..S.R.......   X   lau   Weddell seal
   .T..EL.F.T.A...(ES.)...S.R.......   X   lau   Black flying-fox
   .T..EL.F.T.A...(ES.)...S.R.......   X   lau   Megabat
   EPGR.L...T.APL.(QS.)S..S........A   X   lau   David's myotis bat
   EP.R.L...T.APL.(KS.)S..S.........   X   lau   Microbat
   ET.REL...T.APLA(RSG)VG.G.........   X   lau   Big brown bat
   RTQR.LG.AT.SPLG(EPP)TQPS.R..LG...   X   lau   Hedgehog
   .T.EMLT..AFA..S(KP.)...S.K.KP....   X   lau   Star-nosed mole
   ....L..S.T.A...(QS.)S.VS..EG.....   X   afr   Elephant
   N..--..FHT.A.LS(.S.)TRL..........   O   afr   Cape elephant shrew
   .....L.S.T.A...(QPN).R.S..E......   X   afr   Manatee
   ....A..S.......(KSN)...S..E......   X   afr   Cape golden mole
   .T..MIRS.KIV...(.PG)L.IS.........   X   afr   Tenrec
   .........S.A...(ES.).R.S...G.....   X   afr   Aardvark
   ....MM..ET.AF..(KS.)L..SS....A...   X   xen   Armadillo
   .VA.ML...PFLP..(QSR)LEPS.A..AAG..   X   mon   Platypus

NO 77
GN MUC5B
ID MUC5B_HUMAN
MP 5215
DE Mucin-5B
CL simians
SQ 54
   NGQVFQARLPYSLFH(NNT)EGQCGTCTNNQRD   O   hum   Human
   ...............(...).............   O   hac   Chimp
   ...............(...).............   O   aga   Gorilla
   D.H.....M...H..(...).............   O   gra   Orangutan
   D.H.......H....(...)....---------   O   ape   Gibbon
   D.HI...........(...).............   O   cat   Rhesus macaque
   D.HI...........(...).............   O   cat   Crab-eating macaque
   D.HI...........(...).............   O   cat   Baboon
   D.H............(...).............   O   cat   Green monkey
   ----...........(...).........S...   O   sim   Marmoset
   D.N............(...).........S...   O   sim   Squirrel monkey
   D.HT..V..SFGR.S(H..)..........KE.   X   pri   Bushbaby
   D.HIL..Q.S..H.G(H..).........D...   X   eua   Treeshrew
   ..HT..IQ.S.GY.G(H..).........S.S.   X   gli   Squirrel
   D.H...I..S.G..K(Y..).........S.I.   X   gli   Lesser Egyptian jerboa
   D.HI..IW...RY.S(...).........S.T.   O   gli   Prairie vole
   D.HI..IW...RY.S(...)..R......S.T.   O   gli   Chinese hamster
   D.HI..IQ...RY.S(...)..R......S.T.   O   gli   Golden hamster
   D.NI..IW...RY.S(S..).........S.I.   X   gli   Mouse
   D.NI..IW...RY.S(...).........S.T.   O   gli   Rat
   S.R...V..S..H.S(H..).........S.S.   X   gli   Naked mole-rat
   TRST..VW....H.S(H..).........S.S.   X   gli   Guinea pig
   T.SI..LW.S..H.S(H..).........S.S.   X   gli   Chinchilla
   T.SM..VW.S..R.G(H..).........S.G.   X   gli   Brush-tailed rat
   D..L...Q..F.R.S(H..)..L........K.   X   gli   Pika
   ..R...V..S..R.S(H..)....---------   X   lau   Alpaca
   ..R......S..R.S(Y..).........SR..   X   lau   Dolphin
   ..R......S..R.S(Y..).........SR..   X   lau   Killer whale
   ..R......S..R.S(H..)..........R..   X   lau   Cow
   ..R......S..H.S(H..)..........R..   X   lau   Domestic goat
   S.LI.SVEV.F.K.A(...).........DPKE   O   lau   Horse
   D.R....Q.S..H.N(H..)..L......S.T.   X   lau   White rhinoceros
   D.H...V....TR.S(H..).........S.T.   X   lau   Cat
   D.HL..I..S..H.S(H..)...........N.   X   lau   Dog
   D....HIQ.S..R.S(H..).........SRT.   X   lau   Ferret
   ..D..HIQ.S..H.S(H..).........S.T.   X   lau   Panda
   ..HI.HIQ.S.NR.S(H..).........S.T.   X   lau   Pacific walrus
   ..HL.HIQ.S..R.S(H..).........S.T.   X   lau   Weddell seal
   H..A..IL.S..H.S(Y..)..........KT.   X   lau   Black flying-fox
   T.LI.SVEV.F.K.A(...).........DPK.   O   lau   David's myotis bat
   D.....VQ.S..H.S(H..)....TQSE-----   X   lau   Microbat
   D..A..VQ.S..R.S(H..).........SKT.   X   lau   Big brown bat
   ..HG..IQ...AN.S(H..).........SRA.   X   lau   Hedgehog
   D..A..IQ......S(H..).........S.A.   X   lau   Shrew
   S.LI.SVE..F.K.A(...).......S..PK.   O   afr   Elephant
   D.RL..I..S..Q.N(H..).........TRV.   X   afr   Cape elephant shrew
   ..NI.....S..Q.R(H..).........S.V.   X   afr   Manatee
   D.SI...Q.S..H.S(H..).........S.V.   X   afr   Cape golden mole
   D.AIV.VQ.S.AH..(H..).........S.V.   X   afr   Tenrec
   D.SI...Q.S....S(H..)..........WA.   X   afr   Aardvark
   D.HI.....A..Q.S(H..).........SRT.   X   xen   Armadillo
   S.LL.SVEV.FTK.A(...).........DK..   O   mar   Opossum
   S.ML.SVEV.FTK.A(...).........DK..   O   mar   Tasmanian devil
   Q.KDS.V*RLHI.LP(DRP)G.LGS..SVPA.P   X   mon   Platypus

NO 78
GN NPC2
ID NPC2_HUMAN
MP 135
DE Epididymal secretory protein E1
CL simians
SQ 62
   PSIKLVVEWQLQDDK(NQS)LFCWEIPVQIVSH   O   hum   Human
   ...............(...).............   O   hac   Chimp
   ...............(...).............   O   aga   Gorilla
   ...............(...).............   O   gra   Orangutan
   ...............(...).............   O   ape   Gibbon
   ...............(...).............   O   cat   Rhesus macaque
   ...............(...).............   O   cat   Crab-eating macaque
   ...............(...).............   O   cat   Baboon
   ...............(...).............   O   cat   Green monkey
   ..............A(...)....R......T.   O   sim   Marmoset
   ..............A(...)...........T.   O   sim   Squirrel monkey
   .....T.Q.E.H...(D..)....K..I...T.   X   pri   Bushbaby
   ..V....K.E.K..Q(..N).......IE.AP.   X   eua   Treeshrew
   .......K.E.V..S(HK.)F......I...PP   X   gli   Squirrel
   .....L...K.L..Q(RKI)I...R..L..FP.   X   gli   Lesser Egyptian jerboa
   .......K.E.E...(K.K)........E.---   X   gli   Prairie vole
   ..L....K.E.....(E.N)........E..PR   X   gli   Chinese hamster
   ..L....K.E.....(K.N)........E..PR   X   gli   Golden hamster
   .........K.E...(KNN)..........F*-   X   gli   Mouse
   ..L......K.....(KDN)........E.Y*-   X   gli   Rat
   .DV....K.E.L...(K.D)........E..PQ   X   gli   Naked mole-rat
   .D.....K.E.I...(K.N)........A..PQ   X   gli   Guinea pig
   .D.....K.E.L...(..N).....V..A..PQ   X   gli   Chinchilla
   .D.....K.A.L...(S.N)........A..PP   X   gli   Brush-tailed rat
   .......K.E.K...(QRN).......IA.APV   X   gli   Rabbit
   .......K.E.K.E.(QK.).......IE..PL   X   gli   Pika
   .........K....N(D.C)....Q......PP   X   lau   Pig
   .......Q.E.....(D.R)....Q......P.   X   lau   Alpaca
   .......Q.E.....(D.R)....Q......P.   X   lau   Bactrian camel
   .........E.T..N(D.R)....Q......P.   X   lau   Dolphin
   .........E.T..N(D.R)....Q......P.   X   lau   Killer whale
   ....V....E.T...(..R)F...Q..IEV.PC   X   lau   Tibetan antelope
   ....V....E.T...(..R)F...Q..IEV.PR   X   lau   Cow
   ....V....E.T...(..R)F...Q..IEV.PC   X   lau   Sheep
   ....V....E.T...(..R)F...Q..IEV.PC   X   lau   Domestic goat
   .......K.E.....(G..)...........R.   X   lau   Horse
   .......Q.E.....(G.R)....K.....---   X   lau   White rhinoceros
   ....VM.K...LG..(E.N)...........P.   X   lau   Cat
   .......Q.M.LG.N(..H)...........P.   X   lau   Dog
   .......Q.E.LG..(G.H)...........PR   X   lau   Ferret
   .......Q.E..G.N(D.R)...........P.   X   lau   Panda
   .T.....Q.E.LG..(D.R)...........--   X   lau   Pacific walrus
   .......Q.E.LG..(D.R)...........P.   X   lau   Weddell seal
   .......K.E.....(D.Y)..........LQ-   X   lau   Black flying-fox
   .......K.E.....(D.Y)..........LQ-   X   lau   Megabat
   .......K.E.R...(D.C).........LLQ-   X   lau   David's myotis bat
   .......K.E.R...(D.C).........LLQ-   X   lau   Microbat
   .......K.E.R...(D.C).........L---   X   lau   Big brown bat
   .....L.....K..R(D..)F...Q......P-   X   lau   Hedgehog
   .......K.E.....(H..)...........A-   X   lau   Shrew
   .......K.E..A.G(GHR)........E.AP-   X   lau   Star-nosed mole
   .T.......E.L...(G.H)....K..LE.IL.   X   afr   Elephant
   .Q.....K.L.K...(GKY).......LE.ILC   X   afr   Cape elephant shrew
   .........E.L...(G.R)....K..LE.IL.   X   afr   Manatee
   .T...L.K.E.R...(D.C).......L..ILQ   X   afr   Cape golden mole
   .......K.E.V...(K..)I......LE.ILQ   X   afr   Tenrec
   .T.....K.E.L..N(KKR)........E.IL.   X   afr   Aardvark
   ..V....K.E.K...(K..)....K..IE..PY   X   xen   Armadillo
   .KM....K.T.L...(KNT).......IE.AFT   X   mar   Opossum
   .N.....Q.E.L...(SNI)V...K..IE..F-   X   mar   Tasmanian devil
   .NM......KFW...(STF)....K..IETAFT   X   mar   Wallaby
   .......Q.E.A...(KNR)....Q.....---   X   mon   Platypus

NO 79
GN PAPPA
ID PAPP1_HUMAN
MP 480
DE Pappalysin-1
CL apes
SQ 62
   RFNFDGGECCDPEIT(NVT)QTCFDPDSPHRAY   O   hum   Human
   ...............(...).............   O   hac   Chimp
   ...............(...).............   O   aga   Gorilla
   ...............(...).............   O   gra   Orangutan
   ...............(...).............   O   ape   Gibbon
   ............K..(D..).............   X   cat   Rhesus macaque
   ............K..(D..).............   X   cat   Crab-eating macaque
   ............K..(D..).............   X   cat   Baboon
   ............K..(D..).............   X   cat   Green monkey
   ...............(D..).............   X   sim   Marmoset
   ...............(D..).............   X   sim   Squirrel monkey
   ............D..(D..)K............   X   pri   Bushbaby
   ...............(D..)K............   X   eua   Treeshrew
   ...............(D..)K............   X   gli   Squirrel
   K..Y........D..(D..)K...E........   X   gli   Lesser Egyptian jerboa
   ............D..(D..)K............   X   gli   Prairie vole
   ............D..(D..)K............   X   gli   Chinese hamster
   ............D..(D..)K............   X   gli   Golden hamster
   ............D..(D..)K............   X   gli   Mouse
   ............D..(D..)K............   X   gli   Rat
   ..S............(D..)K............   X   gli   Naked mole-rat
   ...............(D..)K............   X   gli   Guinea pig
   ...............(D..)K............   X   gli   Chinchilla
   ...............(D..)K............   X   gli   Brush-tailed rat
   W..............(D..)K............   X   gli   Rabbit
   H..............(D..)K............   X   gli   Pika
   ...............(D..)K........Y...   X   lau   Pig
   ............D..(D..)K............   X   lau   Alpaca
   ............D..(D..)K............   X   lau   Bactrian camel
   ...............(D..)K............   X   lau   Dolphin
   ...............(D..)K............   X   lau   Killer whale
   ............N..(D..)K...E..NL....   X   lau   Tibetan antelope
   ............N..(D..)K...E..NL....   X   lau   Cow
   ............N..(D..)K...E..NL....   X   lau   Sheep
   ............N..(D..)K...E..NL....   X   lau   Domestic goat
   ...............(D..)K........Y...   X   lau   Horse
   ...............(D..)K........Y...   X   lau   White rhinoceros
   ...............(D..)K............   X   lau   Cat
   ..........N....(D..)K............   X   lau   Dog
   ...............(D..)K............   X   lau   Ferret
   ...............(D..)K............   X   lau   Panda
   ..S............(D..)K............   X   lau   Pacific walrus
   ..S............(D..)K............   X   lau   Weddell seal
   ............D..(D..)K............   X   lau   Black flying-fox
   ............D..(D..)K............   X   lau   Megabat
   ............D..(D..).............   X   lau   David's myotis bat
   ............D..(D..).............   X   lau   Microbat
   ............D..(D..).............   X   lau   Big brown bat
   .Y..........D..(D..)K............   X   lau   Hedgehog
   ..H............(D..)K............   X   lau   Shrew
   ............A..(D..)K............   X   lau   Star-nosed mole
   ............D..(D..)K............   X   afr   Elephant
   ............D..(D..)K............   X   afr   Cape elephant shrew
   ............N..(D..)M............   X   afr   Manatee
   ............D..(D..)K............   X   afr   Cape golden mole
   ............D..(D..)K............   X   afr   Tenrec
   ............D..(D..)K............   X   afr   Aardvark
   ...............(D..)K............   X   xen   Armadillo
   .YS.......N....(D..)K........Y...   X   mar   Opossum
   .Y.............(D..)K........Y...   X   mar   Tasmanian devil
   .Y........N....(D..)K........Y...   X   mar   Wallaby
   .Y........N...S(D..)K........Y...   X   mon   Platypus

NO 80
GN PGLYRP2
ID PGRP2_HUMAN
MP 77
DE N-acetylmuramoyl-L-alanine amidase
CL African great apes
SQ 56
   PHNRLYHFLLGAWSL(NAT)ELDPCPLSPELLG   O   hum   Human
   ............R..(...).............   O   hac   Chimp
   ............R..(...).............   O   aga   Gorilla
   ............Q..(D.A).............   X   gra   Orangutan
   ...........VQ..(D.A)....Y......Q.   X   cat   Rhesus macaque
   ...........VQ..(D.A)....Y......Q.   X   cat   Crab-eating macaque
   ...........VQ..(D.A)....Y......Q.   X   cat   Baboon
   L.T........VQN.(D.A)..H.Y......Q.   X   cat   Green monkey
   ...H......E....(D.A)....YS.....Q.   X   sim   Marmoset
   ...H..R...E.Q..(D.A).W..YS.....Q.   X   sim   Squirrel monkey
   ...H.H......Q..(.TD)K..TYT...G.QF   X   pri   Bushbaby
   TS.P.H....ETP.H(KTA)D..AH......Q.   X   gli   Squirrel
   ...S.H.L.RK.P.H(.M.).SE.HS.....QT   O   gli   Prairie vole
   ...S.H.L.RKTP.H(.TM).P..HS.....QA   X   gli   Chinese hamster
   ...S.H.L.RK.P.H(.TM).T.LHS.....QA   X   gli   Golden hamster
   T..S.H.L..K.P.H(.T.).P..HS.....QA   O   gli   Mouse
   S..S.H.L..K.P.Q(.T.).P.THS.....QA   O   gli   Rat
   .RHP.H.L..E.P..(KI.)..E.P..N...QD   X   gli   Naked mole-rat
   .LHP.H.L..Q.P..(KV.)K...P......RD   X   gli   Guinea pig
   .VHP.H.L..TMPN.(KV.)..Q.P..N...QD   X   gli   Chinchilla
   .LHP.H.L..NVP.R(KV.)..K.R..N...QD   X   gli   Brush-tailed rat
   HR.P.RG...E.PEF(K.A)ARE.R.......S   X   gli   Rabbit
   ..SP.HPL..LIPVF(Q..)AR.H......F.N   X   gli   Pika
   A..P.PR...EGQ..(KTA)K.A.PS....FQ.   X   lau   Pig
   S..P.RR...EGQT.(KTI)K..LPS.....Q.   X   lau   Alpaca
   S.DP.RR...EGQT.(KTI)K..LPS.....Q.   X   lau   Bactrian camel
   A..P.Q....EEQ..(KT.)M...PS.....Q.   X   lau   Dolphin
   A..P.Q....EEQ..(KT.)M...PS.....Q.   X   lau   Killer whale
   T.SP.H....EGKR.(.I.)...LPS.....R.   O   lau   Tibetan antelope
   T.SP......EGK..(.T.)....PS.....R.   O   lau   Cow
   T.SP.H....EGKC.(.I.)....PS.....R.   O   lau   Sheep
   T.SP.H....EGKR.(.I.)....PS.....R.   O   lau   Domestic goat
   ..DA.H....EGRR.(K.M)....P......R.   X   lau   Horse
   .YDP.H....RGR.P(K..)D...P...S..Q.   X   lau   White rhinoceros
   .RDLVH....EG...(K..)R...HQ.....R.   X   lau   Cat
   .RDL.H....ERQN.(K..)N...HQV....RA   X   lau   Dog
   ..DV.HR...EQ.NI(KD.)R...QQ..S.FQ.   X   lau   Ferret
   .QDL.H....EGRT.(Q..)K...QQ....V..   X   lau   Panda
   R..L.H.V..EGQN.(...)K...QQ.....Q.   O   lau   Pacific walrus
   H.DL.H....EGQN.(K..)K...QQ.....R.   X   lau   Weddell seal
   ..DP.H....QS...(Q.I)....IT.....Q.   X   lau   Black flying-fox
   .QDP.H....QS...(Q.I)....TT.....Q.   X   lau   Megabat
   .D----------PL.(KV.)K...PA.....Q.   X   lau   David's myotis bat
   .QDP.H....KER.P(KV.)K...SA.....Q.   X   lau   Microbat
   ..DP.H....KGR..(KV.)K..LSA.....Q.   X   lau   Big brown bat
   .QDPMFS..HKDRG.(G.P)KTEAPR.....EQ   X   lau   Hedgehog
   LLDP..V...DGQH.(LTA)K.GAPE.....QA   X   lau   Shrew
   ..ESF.R...EG.P.(EPV)K.E.L......GD   X   lau   Star-nosed mole
   ...P..R...KEQL.(K.I)K...HR.....R.   X   afr   Elephant
   ..EP.H...MEGQGF(RDS)..N.HL.....R.   X   afr   Cape elephant shrew
   ..DP.RR...KKEG.(K.I)K...HL...K.Q.   X   afr   Manatee
   .SDLFHS...EGQG.(KDI)K...LL.....QD   X   afr   Cape golden mole
   A.DLFHS..FERQD.(KVI)K.G.DS.....Q.   X   afr   Tenrec
   ..DS.HR.V.YGQG.(K.M)...RHL..T..RD   X   afr   Aardvark
   SIDSFQ.L..QG--.(EEL)K...--.G...QA   X   mar   Opossum
   .RDAFLQL..EME.P(PPD).--.D....VQRE   X   mon   Platypus

NO 81
GN PGLYRP2
ID PGRP2_HUMAN
MP 367
DE N-acetylmuramoyl-L-alanine amidase
CL simians
SQ 59
   QLQCMSQEQLAQVAA(NAT)KEFTEAFLGCPAI   O   hum   Human
   ...............(...).............   O   hac   Chimp
   ...............(...).............   O   aga   Gorilla
   ....T..........(...).............   O   gra   Orangutan
   ..R............(...).............   O   cat   Rhesus macaque
   ..R............(...).............   O   cat   Crab-eating macaque
   ..R.I..........(...).............   O   cat   Baboon
   ...............(...).............   O   cat   Green monkey
   ...H....H.....T(...).............   O   sim   Marmoset
   ...H.T..H.....T(...)...M.........   O   sim   Squirrel monkey
   ...D..........T(Y..)Q....V.......   X   pri   Bushbaby
   ...DV.PQE..H..T(R..).............   X   eua   Treeshrew
   ..RNV.........T(Y.A).............   X   gli   Squirrel
   ...N.T..E.....S(L..).....T.......   X   gli   Prairie vole
   ...N....E..R..T(F..).....T.......   X   gli   Chinese hamster
   ...NK...E...G.T(F..).....T.......   X   gli   Golden hamster
   ...NI.........T(L..).............   X   gli   Mouse
   R..N...K......T(F..).............   X   gli   Rat
   ...NI..........(H.A)R...KD.......   X   gli   Naked mole-rat
   ..HNI..DH......(Q.A)...V.........   X   gli   Guinea pig
   ...NI..........(H.A)R..M.........   X   gli   Chinchilla
   ...NI.....G...S(H.A)T..M.........   X   gli   Brush-tailed rat
   ...N..........T(H..)E............   X   gli   Rabbit
   ..WNV..D......T(R.A)......Y......   X   gli   Pika
   ...G..........T(H.A).............   X   lau   Pig
   ...G.N........T(H..).............   X   lau   Alpaca
   ...G..........T(H..).............   X   lau   Bactrian camel
   ...G..........T(H..).............   X   lau   Dolphin
   ...G..........T(H..).............   X   lau   Killer whale
   H..G....E.....T(H..).............   X   lau   Tibetan antelope
   H..G....E.....T(H..).............   X   lau   Cow
   H..G....E.....T(H..).............   X   lau   Sheep
   H..G....E.....T(H..).............   X   lau   Domestic goat
   ...GL.P...V...T(H.S)T.......-----   X   lau   Horse
   ...GI.........T(H.S).............   X   lau   White rhinoceros
   ...G..........T(...).............   O   lau   Cat
   ..RG.......E..T(...).............   O   lau   Dog
   ...G.......E..T(...).............   O   lau   Ferret
   ..RG.......E..T(...).............   O   lau   Panda
   ..RG......FE..T(...).............   O   lau   Pacific walrus
   ..RG....R.FE..T(...).............   O   lau   Weddell seal
   ...G......VL..T(H..)....K........   X   lau   Black flying-fox
   ...G......VL..T(H..)........-....   X   lau   Megabat
   ...GL...K.E.A.T(H.A).............   X   lau   David's myotis bat
   ...GL...E...A.T(R.A).............   X   lau   Microbat
   ...G........A.T(H..).............   X   lau   Big brown bat
   EITGT...R..AA.S(Q.A)E..M....-----   X   lau   Hedgehog
   ...GIG.....K..T(H.A)T............   X   lau   Shrew
   ...G...G..EKI.T(H..)....D........   X   lau   Star-nosed mole
   ...G...D......S(H..).............   X   afr   Elephant
   .V.G..HD......S(K..)........E....   X   afr   Cape elephant shrew
   ...G...D......S(H..)E...K...D....   X   afr   Manatee
   ..HG..GD......S(H..).....V..E....   X   afr   Cape golden mole
   ..KDTNLG.MTR..R(H.S)........E....   X   afr   Tenrec
   ...G...D....M.N(H..)....K...E....   X   afr   Aardvark
   .MANLL.T...E...(R.A)........E....   X   mar   Opossum
   ..KNL..D...E..T(Q.A)........E....   X   mar   Tasmanian devil
   ..ANL..G...E..T(Q.A)........E....   X   mar   Wallaby
   ..APLARTE..H...(R.A)E....S..K....   X   mon   Platypus

NO 82
GN PIGR
ID PIGR_HUMAN
MP 186
DE Polymeric immunoglobulin receptor
CL African great apes
SQ 61
   LYPVLVIDSSGYVNP(NYT)GRIRLDIQGTGQL   O   hum   Human
   .........ND....(...)...H.........   O   hac   Chimp
   ...............(...).............   O   aga   Gorilla
   .S.......T.....(..M)...H..V......   X   gra   Orangutan
   .S.............(S.R).............   X   ape   Gibbon
   QTS......RN....(..K)D....R.P.....   X   cat   Rhesus macaque
   QTS......RN....(..K)D....R.P.....   X   cat   Crab-eating macaque
   QTS......RN....(..K)D....R.P.....   X   cat   Baboon
   QSS......RS....(..Q).....R.P.....   X   cat   Green monkey
   EN...IT.TTS..S.(..K)...H...H..D..   X   sim   Marmoset
   SSHMV....TK..S.(P.R)..T....H.....   X   sim   Squirrel monkey
   SSCL.L...NK.TH.(..L)...S.I....S.S   X   pri   Bushbaby
   NGEM..T.TN....E(Y.K).....TPT..S..   X   eua   Treeshrew
   E---...NTN-....(.FK)..ASIV....N..   X   gli   Squirrel
   QSCE.....AEN.S.(SFN)..A..I....S.Q   X   gli   Lesser Egyptian jerboa
   QTCE.....NKN...(..K)..VN.NLG..SAS   X   gli   Prairie vole
   QTCE.....TE....(K.K)D.AI.FMK..S.E   X   gli   Chinese hamster
   QTCE.....TE....(K.K)..TT.FMK..NPE   X   gli   Golden hamster
   QSCE.....TEK...(S.I)..AK.FMK..DLT   X   gli   Mouse
   EACEV....TE..D.(S.K)D.AI.FMK..SRD   X   gli   Rat
   QSCE.....DKNE..(SFA)..V..V....D.E   X   gli   Naked mole-rat
   ESC......NNN.H.(SFI)..V..VV......   X   gli   Guinea pig
   QSCE...N.N.DIS.(SFQ)..V..V....S.D   X   gli   Chinchilla
   QSCE...N.NRE.G.(SFQ)..VS.A....D.S   X   gli   Brush-tailed rat
   EDGE.I....RQ-R.(R.K)...T.Q..S.TAK   X   gli   Rabbit
   QIR......N..L.N(.F.)N.AH.S....N..   O   lau   Pig
   QSC..IA.TNN.M..(S.K)..AHIS....S..   X   lau   Alpaca
   QSC..IA.TNN.M..(S.E)..AHIS....S..   X   lau   Bactrian camel
   QSC...T..T...S.(S.S)N.VH.N....NE.   X   lau   Dolphin
   QSC...T..T...S.(S.S)H.VH.N....NE.   X   lau   Killer whale
   QDCF.I...N..M.E(..K)D..H.S.L..ST.   X   lau   Tibetan antelope
   QDCFQ....NN..SD(K.K)D..H.S.P..NT.   X   lau   Cow
   QDCF.I...T...SG(S..).....N.A..NT.   X   lau   Sheep
   QGCF.I...T..K.E(..E)D....N.A..DT.   X   lau   Domestic goat
   KHCIR....A..KG.(..E)..AK.I....T.T   X   lau   Horse
   QDCIP..........(S.S)N.AQ.S....N..   X   lau   White rhinoceros
   QLC......N.....(..S)N.AVI..D..S.V   O   lau   Cat
   KGC.T....T.E..S(D.G)...E.E.M..D..   X   lau   Dog
   QACLT....N...SS(..A)D.AEIK.A..S..   X   lau   Ferret
   QSC...T..NK....(..K)D.VE.V.D..N..   X   lau   Panda
   QSC......TEN.S.(..K)D.AEII....N..   X   lau   Pacific walrus
   QVC...A..KEE.H.(D.E)..AEIF.SD.N..   X   lau   Weddell seal
   ETCI.........S.(S.C)D.V..T....S..   X   lau   Black flying-fox
   ETC..........S.(S.C)D.V..S....S..   X   lau   Megabat
   ETC...T..T...S.(D.S)N.AHIT..D.S..   X   lau   David's myotis bat
   KQC...T..T...S.(D.S)N.AEIT..D.S..   X   lau   Microbat
   GQC.....TN.....(E.S)N.ASII..D.S..   X   lau   Big brown bat
   QGC....N....I..(..E)N.AQ.F.YD.SAA   X   lau   Hedgehog
   QECIP..N---.K..(DFG)N..S.YPG..SAK   X   lau   Shrew
   SE-I.LFN.N..LDS(YFK)D.LEFPFS..SES   X   lau   Star-nosed mole
   QDC......T...S.(..K)D.AHFKL...S..   X   afr   Elephant
   QSFYII...G..K.N(..K)D.AD.N.D..SGQ   X   afr   Cape elephant shrew
   QDC..IT........(K.K)D.A.FEQYS.N..   X   afr   Manatee
   QNC.........IS.(I.K)..TD.KL...S..   X   afr   Cape golden mole
   DHS..I...N--T..(L.K)..VN.HVPD.S..   X   afr   Tenrec
   QNC.T....A.S.SA(D.V)..ASFNL...SK.   X   afr   Aardvark
   QSCS.....FENKG.(S.N)E.TKFSLK.....   X   xen   Armadillo
   KNCE.....QNQ.G.(D..)..V.FR.S..SSK   X   mar   Opossum
   ESCS.....LNQKG.(D.E)E.V.FK.S...SE   X   mar   Tasmanian devil
   KSCA.....QNN.GS(D..)E.V.FR.S..SSK   X   mar   Wallaby
   TSCTD....I.....(EFQ)..VS.TVHD.MRK   X   mon   Platypus

NO 83
GN PIGR
ID PIGR_HUMAN
MP 499
DE Polymeric immunoglobulin receptor
CL apes
SQ 62
   FPCKFSSYEKYWCKW(NNT)GCQALPSQDEGPS   O   hum   Human
   ...............(...).............   O   hac   Chimp
   ...............(...).............   O   aga   Gorilla
   ...............(...).........K...   O   gra   Orangutan
   ...............(...).............   O   ape   Gibbon
   ...............(S..)...T.........   X   cat   Rhesus macaque
   ...............(S..)...T.........   X   cat   Crab-eating macaque
   ...............(S..)...T.........   X   cat   Baboon
   L..............(S..)...T.........   X   cat   Green monkey
   .....Y.........(S.K).............   X   sim   Marmoset
   .....Y.........(SEK)..R..........   X   sim   Squirrel monkey
   .....Y.H.......(S.K)...V.........   X   pri   Bushbaby
   .....Y.........(S.K).......E.....   X   eua   Treeshrew
   ....YYTF.......(S.G)E.RE.....KS..   X   gli   Squirrel
   .....Y.........(S.R)..RL......SSR   X   gli   Lesser Egyptian jerboa
   Y...LY.HD......(S.G)..HT.S.----AR   X   gli   Prairie vole
   Y...YY.H.......(S.K)..HI...HN.AAR   X   gli   Chinese hamster
   Y...YY.H.......(S.K)..RI...H..AAR   X   gli   Golden hamster
   Y....Y.Q.......(S.K)..HI...H...AR   X   gli   Mouse
   Y....Y.Q.......(S.D)..HI...H...AR   X   gli   Rat
   Y....YA.K......(S.Q)..R..........   X   gli   Naked mole-rat
   Y....Y.........(SKQ).............   X   gli   Guinea pig
   ....YY....F....(S.Q).............   X   gli   Chinchilla
   .....Y.........(S.Q).............   X   gli   Brush-tailed rat
   ....YF.S.......(.DH)..ED..TK-LSS.   X   gli   Rabbit
   ....YN.......R.(SSQ)D.ET.TT.E.DA.   X   gli   Pika
   .....Y..Q......(S..)..R........Q.   X   lau   Pig
   .....Y.S.......(S..)..RT.....Q.A.   X   lau   Alpaca
   .....Y.S.......(S..)..RT.....Q.A.   X   lau   Bactrian camel
   .....Y.........(S.K)..RT.........   X   lau   Dolphin
   .....Y.........(S.K)..RT.........   X   lau   Killer whale
   .....Y.F.......(S.K)..SP..T.ND...   X   lau   Tibetan antelope
   .....Y.F.......(S.R)..S...T.ND...   X   lau   Cow
   .....Y.F.......(S.E)..SP..T.ND...   X   lau   Sheep
   .....Y.F.......(S.E)..SP..T.ND...   X   lau   Domestic goat
   S....Y..K.F....(TDQ)..S........SG   X   lau   Horse
   S...YY..K.F....(T.E)..R........S.   X   lau   White rhinoceros
   ....YY.........(S.K)..KT..T..D...   X   lau   Cat
   ....YY.........(S.K)D.KI....G....   X   lau   Dog
   ....YY.FQ......(SKK)..TT.........   X   lau   Ferret
   ....YY.........(S.K)..KT.......H.   X   lau   Panda
   ....YY.........(S.K)..KT....---S.   X   lau   Pacific walrus
   ....YY.........(S.K)V.KT....A....   X   lau   Weddell seal
   L....A....F....(SSK)..K....HN..SG   X   lau   Black flying-fox
   L....A....F....(SSK)..K....HN..SG   X   lau   Megabat
   S....F.........(S.K)..KV....N.ASR   X   lau   David's myotis bat
   S....F.........(S.K)..KV....N.ASR   X   lau   Microbat
   S....F.........(S.E)S.KV......ASR   X   lau   Big brown bat
   V....Y..D......(T.K)..RTF........   X   lau   Hedgehog
   V....Y.........(T.K)..K...T..P.F.   X   lau   Shrew
   VS.Q.Y.S.......(TDR)..K......K..N   X   lau   Star-nosed mole
   .....Y.H.......(S.R)S.LT.........   X   afr   Elephant
   .....YPH.......(S..).............   X   afr   Cape elephant shrew
   ....YY.........(S.R)S..N.L..E....   X   afr   Manatee
   Y....YTQ.......(S.K)...T.....D...   X   afr   Cape golden mole
   .S.HYY..T......(SD.)..RT....E..S.   X   afr   Tenrec
   .....Y.........(S.K).............   X   afr   Aardvark
   .....Y..V......(SEQ)..RT.........   X   xen   Armadillo
   .....Y.......R.(S.H)..ET.ST..G.N.   X   mar   Opossum
   .....Y.........(S.Q)..KT.A..E..N.   X   mar   Tasmanian devil
   .A..LY.........(S.Q)..EN..T.E..S.   X   mar   Wallaby
   ....Y.....F....(SRQ)...PI....Q...   X   mon   Platypus

NO 84
GN PRG2
ID PRG2_HUMAN
MP 86
DE Bone marrow proteoglycan
CL catarrhines
SQ 59
   GAVESISVPDM-VDK(NLT)CPEEEDTVKVVGI   O   hum   Human
   ...............(...).............   O   hac   Chimp
   .......L.......(...).............   O   aga   Gorilla
   ...............(...).............   O   gra   Orangutan
   ......L........(...).............   O   ape   Gibbon
   ...KL..........(...).............   O   cat   Rhesus macaque
   ...KL..........(...).............   O   cat   Crab-eating macaque
   ...AL..........(...).............   O   cat   Baboon
   ....L..........(...).............   O   cat   Green monkey
   R.........V...R(..L)..K.....QL...   X   sim   Marmoset
   ..........L....(..P)........QL..V   X   sim   Squirrel monkey
   T.A....AL.V....(DVQ)..K......IT..   X   pri   Bushbaby
   ....PV.AL.V..E.(D.Q)..K.D.V.TL..S   X   eua   Treeshrew
   EPM..D.AL.V....(D.Q)..K......LE.S   X   gli   Squirrel
   E....D.AL.V.L.R(D.Q)..K......LE.S   X   gli   Lesser Egyptian jerboa
   ..----.GQ...A.E(DFQ)..K....ISLK.N   X   gli   Prairie vole
   .SD.GA.GQ...T.E(D.Q)..RA...MSLP.N   X   gli   Chinese hamster
   ..----.GQ.V.S.E(D.Q)..K....MSLM..   X   gli   Golden hamster
   ..----.GQ.V.T.V(D.Q)..K....TSLM.D   X   gli   Mouse
   ..----.GQ.V.T.E(..Q)..K....TSLM.D   X   gli   Rat
   ...A.GAEL...GRE(GVQ)..K......F..S   X   gli   Naked mole-rat
   EE...S.EL...GPE(DVQ)..K...I..FE.S   X   gli   Guinea pig
   .E...S.EL.V.GHE(DIQ)..T....I.F..S   X   gli   Chinchilla
   ER..PS.EL...GHM(DIQ)..K...A..L..S   X   gli   Brush-tailed rat
   ..A..G.DL.V...T(A.Q)..K.D....LAIS   X   gli   Rabbit
   EPM..-.DLVI.L.D(K.Q)..K.....L.Q.N   X   gli   Pika
   E.M.DQAAL.E.---(.VE)..R...R.LMQAS   X   lau   Pig
   .T.....ALEE..G.(DIQ)..K......LE..   X   lau   Alpaca
   .T.....ALEE..G.(DFQ)..K.......E..   X   lau   Bactrian camel
   .......AL.E...R(D.Q)..KD.....LE..   X   lau   Dolphin
   .......AL.E...R(D.Q)..KD.....LE..   X   lau   Killer whale
   .PA..V.SLGE..AE(D.*)H.R.G.A.GLE.V   X   lau   Tibetan antelope
   .PA....GW.E..A.(D.*)HSQ..--------   X   lau   Cow
   .PA....GLGE..AE(D.*)H.Q.GEA.GLE.V   X   lau   Sheep
   .PA....GLGE..AE(D.*)H.Q.G.A.GLE.V   X   lau   Domestic goat
   ...K.V.ALEE....(DFQ)..K......Q...   X   lau   Horse
   .P.K.D.ALSE....(DIQ)..K..E...L...   X   lau   White rhinoceros
   ....LA.AL.E....(GFQ)..K......LE..   X   lau   Cat
   ....CD.SL.K.EEE(DFQ)..K.*.I.RLE.S   X   lau   Dog
   .S...SLAL.A.E.E(EFQ)..K......LEDS   X   lau   Ferret
   ..A..V.AL.Q.E.E(DFQ)..K......LGDS   X   lau   Panda
   .....A.AL.E.E..(DFQ)..K......LENS   X   lau   Pacific walrus
   R..D.A.AL.K.E.E(DFQ)..K......LENS   X   lau   Weddell seal
   E....DPAL.E.---(D.Q)..R......MS.G   X   lau   Black flying-fox
   K.M..D.AL.E.---(..Q)..RA...EEML.S   X   lau   David's myotis bat
   K.M..DPALEE.---(..Q)..RA...EEML.S   X   lau   Microbat
   K.M..HPA---.L..(.FQ)..RA....EIS.S   X   lau   Big brown bat
   .------ALEE.E..(DFQ)..K....AQLE.N   X   lau   Hedgehog
   .GRAFVLHVNLGLSQ(HPI)V.QA-----LP..   X   lau   Shrew
   R..KA..DLGT....(SFQ)..KK....EL..S   X   lau   Star-nosed mole
   EPM..V.ALAE...N(DFQ)..K......LE.S   X   afr   Elephant
   ELM..V.ALAE...H(YFQ)Y.G...SKLDS.-   X   afr   Cape elephant shrew
   EP..LV.ALAE...N(DFQ)..K......L..S   X   afr   Manatee
   EPMDLDAPL.-.--.(D.Q).SGD..L.H.L.S   X   afr   Tenrec
   EPT..V.ALAE...S(.FQ)..K......L..S   X   afr   Aardvark
   .DA..V.ALAE.A..(DFQ)..K......L..S   X   xen   Armadillo
   E.T.LVP..ST.KED(INV)..K...VIHLK.S   X   mar   Opossum
   E.TDFVP..AT.KEN(DSV)SLK.G.I.NLT.T   X   mar   Tasmanian devil
   E.P.LVP..VT.KED(DNV)..K...I.HLT.S   X   mar   Wallaby

NO 85
GN PTPRC
ID PTPRC_HUMAN
MP 276
DE Receptor-type tyrosine-protein phosphatase C
CL African great apes
SQ 55
   NTCTNNEVHNLTECK(NAS)VSISHNSCTAPDK   O   hum   Human
   ...............(.M.).............   O   hac   Chimp
   ...............(.M.).............   O   aga   Gorilla
   .N.I...L...K..E(QI.)L..F.......YR   X   gra   Orangutan
   .N.......K.A...(EIN)........S--Y.   X   ape   Gibbon
   .N......L..P...(EMN).FV......DRH.   X   cat   Rhesus macaque
   .N......L..P...(EMN).FV......DRH.   X   cat   Crab-eating macaque
   .N......L..P...(EMN).FV......DRH.   X   cat   Baboon
   .N......L..P...(EMI)..V......DRH.   X   cat   Green monkey
   FD....RLQ..K..E(IRT).T.FG......N.   X   sim   Marmoset
   FN....TL....D..(IKT).T..DD.......   X   sim   Squirrel monkey
   FN.....F...QQ.T(SFT).N.T.HT.AG.P.   X   pri   Bushbaby
   -E.I..T..G.K..E(.ID)I....T..AF.N.   X   eua   Treeshrew
   KD.---KLQG.RG.H(.DI)FNL..T...-.F.   X   gli   Squirrel
   EE.-------KL..S(MSN)ITLVDS...-SN.   X   gli   Lesser Egyptian jerboa
   VD.---KLKY.P..S(KKN)FNV.DG...-.A.   X   gli   Prairie vole
   AD.---.LK..Q..S(QSN)FTV.NG..I-.H.   X   gli   Chinese hamster
   VD.---.LK..Q..S(QRN)FTV.NG..I-...   X   gli   Golden hamster
   ED.-E.VLN..E..S(QKN)I.V.ND..A-.AT   X   gli   Mouse
   TD.-EK.LK..P..S(QKN).TL.NG...-...   X   gli   Rat
   VK.--EDLE..R..S(HP.)IE.ADD..H-.PS   X   gli   Naked mole-rat
   PE.-EKTLQG.H..S(HVT).L.GDGN.SS.I-   X   gli   Guinea pig
   ED.-RKVLQ..KG.S(.LT)IH.ADG..S-.PT   O   gli   Chinchilla
   KN.-EEVLQR..G.S(EHV)IQ.TDD..S-RH.   X   gli   Brush-tailed rat
   LI.K..SL.-.QG.E(QI.)IN......VS.P.   X   gli   Rabbit
   -N...KSI.-.KA.D(DRT).R..--L..G.PR   X   gli   Pika
   -D.-AKMLQ..Q..E(HRT).NL..S..VP.F.   X   lau   Pig
   -E.R.KQLFG.ET.T(DHT)IE.K.E..GD.P.   X   lau   Alpaca
   -E.RQ.QL.G.ET.T(.HT).E.K.D..GD.P.   O   lau   Bactrian camel
   ----SD.L.G.KA.E(KKN)IN.T.P..YP.F.   X   lau   Dolphin
   ----SD.L.G.K..E(KKN)IN.T.P..YP.F.   X   lau   Killer whale
   -G.-EEQHRG.SA.E(AKV)INM..P..EP.FE   X   lau   Tibetan antelope
   -G.-EK.HRG.SA.Q(TKN)I.M..P..EP.FE   X   lau   Cow
   -G.-EKQHRG.PA.G(AKA)IN...P..EP.LA   X   lau   Sheep
   -G.-EQQH.D.ST.D(IKV)IN...P..EP.LE   X   lau   Domestic goat
   IS.V..MF.G.P...(IFT).R.Y....S--Y.   X   lau   Horse
   I..V..TLKG.Q..E(IFA).D...D..ST.H.   X   lau   White rhinoceros
   AI.KM..IL..PA.E(.KN).T...T..DP.N.   X   lau   Cat
   -Q.KK..IS..K..E(.LN)IT...S..DS.N.   X   lau   Dog
   V..E..MIY.....S(H..)FT...E..VG.N.   X   lau   Ferret
   A..SK..IL.....E(.KN).T......GT.N.   X   lau   Panda
   AS.P.K.IND.P..E(.TN).T......HI.N.   X   lau   Pacific walrus
   A..VE..IQT.P..A(.IN).T......HT.N.   X   lau   Weddell seal
   TP.PEEKLS..SA.D(TIT).NLTGD..E-SA.   X   lau   Black flying-fox
   TP.PEKKLS..SA.D(TIT).NLTGD..E-.A.   X   lau   Megabat
   Q..G.KR.DG.P..Q(TIN).K..DD..AD.P.   X   lau   David's myotis bat
   -N.KCTKIK..SS.Y(E..).A.HSD...Q.Y-   X   lau   Hedgehog
   -..RCDDIG...A.Q(SV*)IYF----------   X   lau   Star-nosed mole
   -P.-----A..S..N(SI.).N..DD..VP.F.   X   afr   Elephant
   -S.-----E..E..S(SPL).T.TN...VD.A.   X   afr   Cape elephant shrew
   -S.-----Q..IA.S(LT.).N..GD..VS.S.   X   afr   Manatee
   -G.-----E..K..T(.SN).TVFRD...R.F.   X   afr   Cape golden mole
   -S.-----K...A.T(YI.).N.YDD..V..F.   X   afr   Tenrec
   -S.-----E..KG..(PT.).....D..VP.L.   X   afr   Aardvark
   TSY-----G.....S(SIN)IC---.P.--.NE   X   mar   Opossum

NO 86
GN PTPRC
ID PTPRC_HUMAN
MP 335
DE Receptor-type tyrosine-protein phosphatase C
CL great apes
SQ 57
   CLKWKNIETFTCDTQ(NIT)YRFQCGNMIFDNK   O   hum   Human
   ...............(...).......TTS...   O   hac   Chimp
   ...............(...)........T..K.   O   aga   Gorilla
   .......KN....PR(...).......TT.NK.   O   gra   Orangutan
   ....EQ....D.G.N(K..).......TT..K.   X   ape   Gibbon
   .....I....A..KS(K..).......KTYNKE   X   cat   Rhesus macaque
   .....I....A..KS(K..).......KTYNKE   X   cat   Crab-eating macaque
   .....I..P.A..KS(K..).S.....KTYNKG   X   cat   Baboon
   .....I--..A..KS(K..).......ETYNKE   X   cat   Green monkey
   Y...E--.K....KN(KF.)......EITSEK.   X   sim   Marmoset
   K....--.N.A..IN(K.A).......ITS.KE   X   sim   Squirrel monkey
   .V..EKN..ID..EN(KF.).S.....ETYKE.   X   pri   Bushbaby
   ..E.I---NSA.E.E(AFV).L.K.T.ATVYKQ   X   eua   Treeshrew
   ....R---GPA.GEE(KFR).EY..D.LTSSG.   X   gli   Squirrel
   ..E.TVKRDY...EK(K.S)FT....KQT--D.   X   gli   Lesser Egyptian jerboa
   ..E.N---RSR.NNT(K..).K.H.NSQEYSQ-   X   gli   Prairie vole
   ..R.NFTHRPD..NK(K.S)...Y..TQT--.S   X   gli   Chinese hamster
   ..E.DFDNR.G...R(K.S)..IR..DPT--DS   X   gli   Golden hamster
   ..E..TKNR.K.NSD(..S).VLH.E.NTNALE   O   gli   Mouse
   ..E..IKNK....I.(K.S).N.R.KMKT.ALE   X   gli   Rat
   H.C.E--.H.--N.E(.FR).E.....FQ.Y.Q   X   gli   Naked mole-rat
   V.Q.EKT.N.K--NE(SF.).E....TSVSHSE   X   gli   Guinea pig
   ..Q...T..LRI--K(EFQ).IYK.DISESP.E   X   gli   Chinchilla
   ..E.I.THKM----K(EF.).K.T...STSTD.   X   gli   Brush-tailed rat
   ..R.DVNARPP.EEN(K.K).K.E.DGKKCEDN   X   gli   Rabbit
   ..Q.NITNN.R.Y.N(K.K).S.K.D.IGS.KN   X   gli   Pika
   ..R.E.SGN.S..EG(KLR).S....KP.PTKE   X   lau   Pig
   ..I.RES.SSA..AN(K..).K....DYTQNSS   X   lau   Alpaca
   ..I.RKNGSSA..AN(K..).K....DYPQNSS   X   lau   Bactrian camel
   ..R.EISKNV...KK(...).K.K.DGLSYNES   O   lau   Dolphin
   ..R.EISKN....KK(...).K.K.DGLSYNES   O   lau   Killer whale
   ..H.QH.-D.M..EN(E.E).K...DGHLNGSE   X   lau   Tibetan antelope
   ..H.Q-NKD.K.NEN(K.E).K.K.DGGSSY..   X   lau   Cow
   ..R.Q-NKD.M..EN(K.E).K...DGR*N.S.   X   lau   Sheep
   ..R.Q-N.N.K..EN(K.E).K...DGR*NVS.   X   lau   Domestic goat
   .---RLEKD...EKD(...).K...DISTSTEA   O   lau   Horse
   .---TMEQGA..EKD(K.A).K...DHSPSNGT   X   lau   White rhinoceros
   ..RLEKK.N....EE(K..)HVLS.A.FSSNKT   X   lau   Cat
   .VRVEEKKN....KS(K.K)..L..D.RTSKDM   X   lau   Dog
   H..V.IQNN....KN(K..).....E.CTSTGE   X   lau   Ferret
   ..TVEEKKS....ET(K..)H.L..D.RTSNDI   X   lau   Panda
   Y..VEENKN....KN(K.R).I.....CTNNK.   X   lau   Pacific walrus
   ...VEKN......NN(K.S).T....IYTVSKT   X   lau   Weddell seal
   ..T.VQTRD.N.EE.(D.R).L.....IR--EG   X   lau   Black flying-fox
   ..N.EQTRG.N.KEE(D.R).......IR--EG   X   lau   Megabat
   ..E.NI.GRMN.L.N(..K)..H....FT--T.   X   lau   David's myotis bat
   ..E..-ENN.N.TRH(K.Y).K...--------   X   lau   Hedgehog
   ..NIT-SHN....EK(K.S).I...D.KG--KT   X   lau   Shrew
   L.I.----N.NGCEE(...).S...A.TSTSEE   O   lau   Star-nosed mole
   ..E..KTSD.S.VEN(...).K.....TSSNES   O   afr   Elephant
   ..E..HTLPLN.EQS(...).VVK..HNKYQC-   O   afr   Cape elephant shrew
   ..E.NI.GKIS.KEE(G..).K.N...ESSSHY   X   afr   Manatee
   ..E..ITRYMN..Y.(...)F..T.D.KTVNDC   O   afr   Cape golden mole
   ..V.EYS-NS..--K(.A.).S.R...ESSHE.   O   afr   Tenrec
   ..I.ATAVS.A.--G(...).KIT...SSSN.S   O   afr   Aardvark
   S.R.TYNRN.D.-..(PLI)FS.EKDPYTQKK-   X   mar   Opossum
   T.E.AYWRN.N.TKN(.LS).N.E---------   O   mar   Wallaby

NO 87
GN PTPRC
ID PTPRC_HUMAN
MP 419
DE Receptor-type tyrosine-protein phosphatase C
CL simians
SQ 54
   HQGVITWNPPQRSFH(NFT)LCYIKETEKDCLN   O   hum   Human
   ...............(...)...M.....N...   O   hac   Chimp
   ...............(...)....N....K...   O   aga   Gorilla
   ..............N(...)...VNK...K..I   O   gra   Orangutan
   .....A.I.......(...)...ENK.G.K...   O   ape   Gibbon
   ...............(...)...VSK.A.K..S   O   cat   Rhesus macaque
   ...............(...)...VNKPA.K..I   O   cat   Crab-eating macaque
   ...I...........(...)...VNK.A.I...   O   cat   Baboon
   ........H......(Y.I)...VNK.A.N...   X   cat   Green monkey
   .K......H.HGL..(...)..FQNKP.DN..K   O   sim   Marmoset
   .K......H.HSL..(SY.)..FQNKP.DN.FK   X   sim   Squirrel monkey
   ESVH.S.S.L.NK.D(G.I)...QNKS..N.FD   X   pri   Bushbaby
   TNVN...QA.GNA..(..S)F.NKMKQ.DV.HD   O   eua   Treeshrew
   NK...S.KS.ESTPS(G.V)..--DNSVEK.AT   X   gli   Squirrel
   NT.IL..S..PTL..(G.I)..FE.IQVGN.V.   X   gli   Lesser Egyptian jerboa
   TKTLVI.DR.STFLN(GYY)..SNNSSVEN.EE   X   gli   Prairie vole
   NTTLV..TH.SPLP.(GYF)S..NNNSVEI.DP   X   gli   Chinese hamster
   NTTSV..TH.SPFP.(GYY)..LNNNPVGE.KT   X   gli   Golden hamster
   RKTLVS.PE.ASKP.(GYV)...KNNSVEK.KS   X   gli   Mouse
   STTLVS.AE.ASKH.(GYI)...KNPSVEK.E.   X   gli   Rat
   T-.E.V.L..SNV--(G.Y).S.LETSVGKEK.   X   gli   Naked mole-rat
   TTAQ.H..SLPNL--(G.Y).S.RNNSAGKDI.   X   gli   Guinea pig
   STAEVI.A..K--YS(G.C)FS.LNISVGKT..   X   gli   Chinchilla
   NEVK.S.A.QPN.--(G.Y)FA.WANSAGKEVV   X   gli   Brush-tailed rat
   VK.S...T...NI..(GY.)V.HQAKSV.N.S.   X   gli   Rabbit
   .E.MM..TA..NV..(GYK)..NTND----.ID   X   gli   Pika
   QE.HV..K..NSV..(K.F)...QSDGDPL.VP   X   lau   Pig
   TDAQLS.E..E.TS.(G..)...RNASDRV..-   X   lau   Alpaca
   TDAQLS.K..E.TS.(G..)...WNASDRV..-   X   lau   Bactrian camel
   DK.WV..E..ANF..(T..)...RNSS..R.HT   X   lau   Dolphin
   .K.WV..E..ENF..(T..).R.RNHS.ER.HT   X   lau   Killer whale
   TE.K...T...NY.D(R.S)...WITS..N.TT   X   lau   Tibetan antelope
   TE.KC..T...SY.D(RIS)...WITPGRN.IP   X   lau   Cow
   TE.K...T...NY.D(R.S)...RITS.PN.TT   X   lau   Sheep
   TE.K...T...NY.D(R.S)...RITS.PN.TT   X   lau   Domestic goat
   KNRT.S.D..ENF..(G..)...QNVSDRN.SL   X   lau   Horse
   D..E...K...N..D(...)...QNTSDQK.FH   O   lau   White rhinoceros
   TE.R...ES.EDY.D(.YV)..LPHAS.EKFIL   X   lau   Cat
   NSV..I.E..INT..(GYN)V..HIN--EM.QE   X   lau   Dog
   TSILL..K..TDT..(.Y.)...--K.D.E.I.   O   lau   Ferret
   NDILL..KH.KDF..(KYH)...ETAQDCE.ED   X   lau   Panda
   NDITL..K...DF..(.YS)...K.DADPK.DE   O   lau   Pacific walrus
   TDI.L..K...DF..(.YN)...KEDEDIT.CD   X   lau   Weddell seal
   NSAL...S..PSLY.(...)..--SSSGSE.Y.   O   lau   Black flying-fox
   NSAL...S..PSLY.(...)...SSGS.--.YD   O   lau   Megabat
   TH.RA..ES.P.D..(...)...LSGP.--.I.   O   lau   David's myotis bat
   N.TT...PH----.S(S.Y)I..LPSDNREVF*   X   lau   Hedgehog
   T.EE.S.E...SVV.(..L)F..WNDS.NA.TI   X   afr   Elephant
   TKN.V..R..KNY.T(G.D)F..EEKSG.N.EK   X   afr   Cape elephant shrew
   TEEE...K...TV..(...)F..KNNS.SN.KC   O   afr   Manatee
   NDEK...K..EDD.Y(M.Y)F..ST.SVEN.TK   X   afr   Cape golden mole
   KKEE...ED.ESD..(K.K)F..SNASANI.MS   X   afr   Tenrec
   D.EE...T...T...(..Y)F..SNDS--Y.E.   X   afr   Aardvark
   REL..N.TH.EGF..(E.C)...EHG.DPN.IY   X   xen   Armadillo

NO 88
GN PTPRJ
ID PTPRJ_HUMAN
MP 342
DE Receptor-type tyrosine-protein phosphatase eta
CL simians
SQ 62
   DTEVLLVGLEPGTRY(NAT)VYSQAANGTEGQP   O   hum   Human
   .........R.....(...).............   O   hac   Chimp
   ...............(...).............   O   aga   Gorilla
   ...............(...).............   O   gra   Orangutan
   ...............(...)...........E.   O   ape   Gibbon
   .......E.K.....(...).............   O   cat   Rhesus macaque
   .......E.K.....(...).............   O   cat   Crab-eating macaque
   .......E.K.....(...).....V.......   O   cat   Baboon
   .......E.KL....(...).............   O   cat   Green monkey
   V........K.....(...)........M....   O   sim   Marmoset
   V........K.....(..I)........M....   X   sim   Squirrel monkey
   .MH......K...Q.(KT.)......DD...K.   X   pri   Bushbaby
   N....ID..K..AK.(Q..)......D......   X   eua   Treeshrew
   HID.....VKSN.Q.(K..)......D..K...   X   gli   Squirrel
   ......T..Q.D.H.(Q..)..P..VD......   X   gli   Lesser Egyptian jerboa
   -S....T..K.D.D.(S.V)......D......   X   gli   Prairie vole
   L.Q...T..K...N.(T.K)..PR..DD.....   X   gli   Chinese hamster
   L.K...L..K.D.N.(T.K)...R..D......   X   gli   Golden hamster
   L..I..TD.K.D.Q.(...)I............   O   gli   Mouse
   L.....TE.K.D.Q.(KV.)I.....D......   X   gli   Rat
   .....IS..KLD.Q.(S..)..PR..D......   X   gli   Naked mole-rat
   .....IS..KTNVQ.(K..)..PR..D....W.   X   gli   Guinea pig
   .....I...KTD.Q.(...)..P...D......   O   gli   Chinchilla
   .....IT..KRDAQ.(R..)..P...D....A.   X   gli   Brush-tailed rat
   --Q...T..D.....(R.S).FP...D......   X   gli   Rabbit
   --K........S.L.(R..).FP...D.....S   X   gli   Pika
   .....VW..KSDAG.(K..)..PR..D.P..H.   X   lau   Pig
   ---...W..KSD.L.(E..)..P..ED....K.   X   lau   Alpaca
   ---.Q.L..KSD.L.(K..)..P..ED......   X   lau   Bactrian camel
   ......F..KSD.Q.(K..)..PR....A....   X   lau   Dolphin
   ......F..KSD.Q.(K..)..PR....A....   X   lau   Killer whale
   ......LR.K.D.Q.(K..)..PR.V..A....   X   lau   Tibetan antelope
   H.....LR.K...Q.(K..)..P..DK.A....   X   lau   Cow
   ......LR.K.D.Q.(K..)..PR.V..A....   X   lau   Sheep
   ......LR.K.D.Q.(K..)..PR.V..A....   X   lau   Domestic goat
   ......L..KSD.Q.(...)..CR.EG......   O   lau   Horse
   G.Q...L..K.D.Q.(K..)...R.TD....K.   X   lau   White rhinoceros
   .....IT..V.D.Q.(C.A)......G.A..G.   X   lau   Cat
   ......F..KSN.Q.(K..)......D......   X   lau   Dog
   N...W.F..K.N.Q.(K..)..P...G.E....   X   lau   Ferret
   ......F..KSN.Q.(K..)......D......   X   lau   Panda
   ......F..KSN.Q.(R..)......D......   X   lau   Pacific walrus
   ......F..KSN.Q.(K..)......D......   X   lau   Weddell seal
   ..K..ILR.KSD.E.(...)...R.PD.K....   O   lau   Black flying-fox
   ..K..ILR.KSD.E.(...)...R.PD.K....   O   lau   Megabat
   ......L..KS..S.(...)..PR.PT.P..E.   O   lau   David's myotis bat
   ......L..KSDMS.(...)..P..PT.L..E.   O   lau   Microbat
   Y.....L..KSD...(K..)..PR..T.Q..L.   X   lau   Big brown bat
   QIK.Q....K.D.Q.(.V.)......D.KK.EA   O   lau   Hedgehog
   .IS.Q.L..S...E.(KV.)...........S.   X   lau   Shrew
   ....T.R..KAD.P.(.VS)....T.G.K..P.   O   lau   Star-nosed mole
   G...H....K...L.(K..)I.....D......   X   afr   Elephant
   A...R....K..MP.(K..).F....D.....A   X   afr   Cape elephant shrew
   G...Q....K...Q.(K..)......D......   X   afr   Manatee
   ....W.A..KS.S..(K..).........K...   X   afr   Cape golden mole
   ..R.WI...Q.S.A.(K..)..P..EG......   X   afr   Tenrec
   -.Q.Q....KS.MK.(K..).............   X   afr   Aardvark
   A..I.IS..KL.AQ.(...).F....D......   O   xen   Armadillo
   S.RIHIT..NS..L.(QV.).F..TT..VK.D.   X   mar   Opossum
   N.R.HVT..NS..S.(Q..).F.........D.   X   mar   Tasmanian devil
   N.R.NVI..NS..S.(QV.).F.....E...D.   X   mar   Wallaby
   L.R.NV...K.S.Q.(T..)IF..E......N.   X   mon   Platypus

NO 89
GN PTPRJ
ID PTPRJ_HUMAN
MP 396
DE Receptor-type tyrosine-protein phosphatase eta
CL catarrhines
SQ 60
   SLTLIWKVSDNESSS(NYT)YKIHVAGETDSSN   O   hum   Human
   ...............(...).............   O   hac   Chimp
   ...............(...).............   O   aga   Gorilla
   ...............(...).............   O   gra   Orangutan
   ...............(...).....V.....T.   O   ape   Gibbon
   ....T..........(...)...........L.   O   cat   Rhesus macaque
   ....T..........(...).....V....PL.   O   cat   Crab-eating macaque
   ....T..I.......(...)...........F.   O   cat   Baboon
   ....T..........(...)...........P.   O   cat   Green monkey
   ..I.T..IN.SDP..(V..)...D.M..MH.L.   X   sim   Marmoset
   T...T..IN.SDP..(V..)...D.V..MH.F.   X   sim   Squirrel monkey
   ....T..IT......(V..).E.V..T..SFLS   X   pri   Bushbaby
   .MI.T.R..H.G.C.(LC.).G.Q.S.D.G.F.   X   eua   Treeshrew
   .M..T..AVY.G...(A.S)...Q....ASVL.   X   gli   Squirrel
   .MS.T...RYGG..W(A..).E...D.GPA.LI   X   gli   Lesser Egyptian jerboa
   NM..T..IKY....Y(P..).......G.N.I.   X   gli   Prairie vole
   NM..T..INN....F(C..).......GSSAF.   X   gli   Chinese hamster
   N.A.T..INY....F(C..).......GSIAI.   X   gli   Golden hamster
   .M..T..SNYDG.RT(SIV).......G.H.V.   X   gli   Mouse
   NM..T..SNN...HA(SF.)...Y...GS..I.   X   gli   Rat
   ..S.T..MRH.G...(A..).E.....D.E.F.   X   gli   Naked mole-rat
   ..S.T.T.RH.G...(A..).E.....HDK.LT   X   gli   Guinea pig
   ..S.T.EM.H.G...(A..).E.....D.E.FD   X   gli   Chinchilla
   ..S.T.NM.HSG...(P..).E.Y.V.N.E.L.   X   gli   Brush-tailed rat
   .M..T..I.H.G.H.(A.A).EVQ.....YFF.   X   gli   Rabbit
   .M..T.NF.H.G.Y.(A..).EV...S.MEFF.   X   gli   Pika
   ....T..IN.H.P..(V..).E.Q.......F.   X   lau   Pig
   ....T..I.......(V.A)...Q...R...F.   X   lau   Alpaca
   ....T..I.......(V.S)...Q...R...F.   X   lau   Bactrian camel
   ....T..INE..P..(V..)...Q......PF.   X   lau   Dolphin
   ....T..INE..P..(V..)...Q......PF.   X   lau   Killer whale
   ...MT..IN......(V..)...Q.V.....F.   X   lau   Tibetan antelope
   ...MT..IN......(V..)...Q.VD....F.   X   lau   Cow
   ...MT..IN......(V..)...Q.V.....F.   X   lau   Sheep
   ...MT..IN......(V..)...Q.V.....F.   X   lau   Domestic goat
   NM..T..IN......(V.M).E.Q..T..SVL.   X   lau   Horse
   .M..T..IN..K...(V..).E.E..T..S.L.   X   lau   White rhinoceros
   TM..T..IN......(V..)...Q.T.....FT   X   lau   Cat
   .MN.T..IN......(L.S).E.Q.....G.L.   X   lau   Dog
   NM..T..IN.D.A..(V..).V.Q...AM..F.   X   lau   Ferret
   NM..S..IN......(V..).E.Q...A...FT   X   lau   Panda
   HM..T..INND....(L..)..VQ...P...FV   X   lau   Pacific walrus
   HM..T..IN......(L..).E.Q..WP...FV   X   lau   Weddell seal
   GM..T..IN.D.P..(F..)F..Q...Q.S.L.   X   lau   Black flying-fox
   GM..T..IN.D.P..(F..)F..Q...Q.S.L.   X   lau   Megabat
   .MI.T..IN.--.CF(V..).E.D.--.PA.L.   X   lau   David's myotis bat
   .MI.T..IN.--.C.(V..).....--.PT.LS   X   lau   Microbat
   .MI.T..IN.SD...(V..).E.R....PA.R.   X   lau   Big brown bat
   NM..T.NI.G.T.YF(V..).E.....G.SNLS   X   lau   Hedgehog
   N...A..LRH.A...(T.S).Q.L.VSP.GNRS   X   lau   Shrew
   .MN.T.EI...V...(D..).Q.Q.V...R.F.   X   afr   Elephant
   .MK.T..IKNRV.T.(I..)F..Q.V...Q.F.   X   afr   Cape elephant shrew
   .MN.T.EIRSPV.FP(LFI).R.Q.V..NS.FS   X   afr   Cape golden mole
   .IM.T..INETLQ..(G.S)...Q..EQ.M.F.   X   afr   Tenrec
   .MN.T.EIR.TVPP.(V..)...Q.V..NQ.F.   X   afr   Aardvark
   .MH.T.E...SV...(V..)..VQ.DR.M..F.   X   xen   Armadillo
   GVN.T.E--NSD...(S..).R.....DGH.FD   X   mar   Opossum
   GVK.F.---E.TD..(S..).R.Q...DGH.FD   X   mar   Tasmanian devil
   TVM.T.E--NSD...(...).R.....DAG.FD   O   mar   Wallaby
   EVS.T.E--NKD...(A.S).R.L.V.NET.F.   X   mon   Platypus

NO 90
GN PTPRJ
ID PTPRJ_HUMAN
MP 413
DE Receptor-type tyrosine-protein phosphatase eta
CL great apes
SQ 61
   TYKIHVAGETDSSNL(NVS)EPRAVIPGLRSST   O   hum   Human
   ...............(...).............   O   hac   Chimp
   ..............F(...).............   O   aga   Gorilla
   ...............(...)......L......   O   gra   Orangutan
   ......V.....T..(T.R).......E.....   X   ape   Gibbon
   ............L..(T..).A....S......   X   cat   Rhesus macaque
   ......V....PL..(T..).A....S......   X   cat   Crab-eating macaque
   ............F..(T..).A....S......   X   cat   Baboon
   ............P..(T..).A....S......   X   cat   Green monkey
   ....D.M..MH.L.G(TFG)....I.L......   X   sim   Marmoset
   ....D.V..MH.F.V(T..)G.H.T.S..C...   X   sim   Squirrel monkey
   ..E.V..T..SFLSV(.I.).MQ......T...   O   pri   Bushbaby
   ..G.Q.S.D.G.F..(T..).TS.D.S..N..S   X   eua   Treeshrew
   S...Q....ASVL..(T.K).TH.TVS..N...   X   gli   Squirrel
   ..E...D.GPA.LI.(TA.).T..T.S..S.G.   X   gli   Lesser Egyptian jerboa
   ........G.N.I.Q(T..)KTE.I.Y..S...   X   gli   Prairie vole
   ........GSSAF.Q(T.N)KTE.I.R..T...   X   gli   Chinese hamster
   ........GSIAI.Q(T.N)KTE.I.H..T...   X   gli   Golden hamster
   V.......G.H.V.Q(T.N)KTE.I.L..S...   X   gli   Mouse
   ....Y...GS..I.E(T.N).TQ...R..S...   X   gli   Rat
   ..E.....D.E.F..(TTD).T..D.L..N.R.   X   gli   Naked mole-rat
   ..E.....HDK.LT.(S.N).TY.N.S......   X   gli   Guinea pig
   ..E.....D.E.FDV(S.N).TS.D.R..N...   X   gli   Chinchilla
   ..E.Y.V.N.E.L.V(SAN).TW.D.H..N..S   X   gli   Brush-tailed rat
   A.EVQ.....YFF.F(T.N)DTH...S..SP..   X   gli   Rabbit
   ..EV...S.MEFF.V(T.N)DT....S..S...   X   gli   Pika
   ..E.Q.......F..(T.N).TQ..LTP....S   X   lau   Pig
   A...Q...R...F..(T..).TH...TA.S...   X   lau   Alpaca
   S...Q...R...F..(T..).TH...AA.S...   X   lau   Bactrian camel
   ....Q......PF..(T.H).T....TP.N...   X   lau   Dolphin
   ....Q......PF..(T.H).T....TP.N...   X   lau   Killer whale
   ....Q.V.....F..(T.N).TY...TA.S.G.   X   lau   Tibetan antelope
   ....Q.VD....F..(I.N).TH...TE.S.G.   X   lau   Cow
   ....Q.V.....F..(T.N).TY...TA.S.G.   X   lau   Sheep
   ....Q.V.....F..(T.N).TY...TA.S.G.   X   lau   Domestic goat
   M.E.Q..T..SVL.V(T.N).T..L.SQ.SP..   X   lau   Horse
   ..E.E..T..S.L..(T.N).T..L.AP.S.R.   X   lau   White rhinoceros
   ....Q.T.....FT.(H.N).TQVA.TS.S...   X   lau   Cat
   S.E.Q.....G.L..(S.N)TKWVF.TS.N...   X   lau   Dog
   ..V.Q...AM..F..(.T.).TWVN..S.K...   O   lau   Ferret
   ..E.Q...A...FT.(.T.).TGVI.TS.N...   O   lau   Panda
   ...VQ...P...FV.(...)DTWVN.SS.S...   O   lau   Pacific walrus
   ..E.Q..WP...FV.(...)DTWVN..S.N...   O   lau   Weddell seal
   .F..Q...Q.S.L..(T.N).TQ...TS.SP..   X   lau   Black flying-fox
   .F..Q...Q.S.L..(T.N).TQ...TS.SP.-   X   lau   Megabat
   ..E.D.--.PA.L..(TIN).TQ...T..S...   X   lau   David's myotis bat
   ......--.PT.LS.(TIN).TQ...A..S...   X   lau   Microbat
   ..E.....G.SNLSV(T.N)KTV...AP.Q..A   X   lau   Hedgehog
   S.Q.L.VSP.GNRSF(TTN).TLT..T..Q.C.   X   lau   Shrew
   ---.Q......VL.F(TTD)VTQ.ILAP.Q...   X   lau   Star-nosed mole
   ..Q.Q.V...R.F..(TAH)VTHV.....T...   X   afr   Elephant
   .F..Q.V...Q.F..(TAN).TQVL.A..T..S   X   afr   Cape elephant shrew
   -.Q.L.VR..TFF..(TAN).MHV.....T...   X   afr   Manatee
   I.R.Q.V..NS.FSH(TAL)..HV..S..T...   X   afr   Cape golden mole
   S...Q..EQ.M.F..(TAN).T.VA....TPC.   X   afr   Tenrec
   ....Q.V..NQ.F.F(TAN).THV.....T...   X   afr   Aardvark
   ...VQ.DR.M..F.F(T.N).T....T..S...   X   xen   Armadillo
   ..R.....DGH.FDQ(.SN)VTHVA.TE.Y...   X   mar   Opossum
   ..R.Q...DGH.FDQ(.SN)DTHVA.T..N...   X   mar   Tasmanian devil
   ..R.....DAG.FDQ(.SN)STHVT.T..N...   X   mar   Wallaby
   S.R.L.V.NET.F.A(SYN)LR....T..N...   X   mon   Platypus

NO 91
GN PVR
ID PVR_HUMAN
MP 278
DE Poliovirus receptor
CL catarrhines
SQ 52
   TLTCDARSNPEPTGY(NWS)TTMGPLPPFAVAQ   O   hum   Human
   ...............(...).............   O   hac   Chimp
   I..............(...).............   O   aga   Gorilla
   ...............(...).............   O   gra   Orangutan
   ...............(...).............   O   cat   Crab-eating macaque
   ...............(...).............   O   cat   Baboon
   ...............(...).............   O   cat   Green monkey
   S..............(E..)........S.M..   X   sim   Marmoset
   S......G.......(E..)........S....   X   sim   Squirrel monkey
   N...NV.........(...)..N.S..HS..P.   O   pri   Bushbaby
   ....NV.......V.(Y.N)..V.......K..   X   eua   Treeshrew
   ..S.E........D.(A.D)..T....S.....   X   gli   Squirrel
   A.S.....K....D.(E..)..T....NNTE..   X   gli   Prairie vole
   V.....Q.K....S.(K..).AT....NTTEP.   X   gli   Chinese hamster
   ....E.H.K.A.DR.(...).NT.DF.NSVKR.   O   gli   Mouse
   N...E...K.P..N.(S..).AT....NSTHF.   X   gli   Rat
   S.N..VQ.....QDV(V.K)..T.T..LS.EP.   X   gli   Naked mole-rat
   N.S..IY.....VTV(V.N)..T.T..RS.EV.   X   gli   Guinea pig
   ..S.EVH.....ENV(V.K)..T.T..RS.E..   X   gli   Chinchilla
   ....EVD....DVNV(V.N)..R.T..LS.K..   X   gli   Brush-tailed rat
   A....V.........(...)..T.....S....   O   gli   Rabbit
   ..S..V....A....(E..)..T.....ST...   X   gli   Pika
   ..N..I..K......(...)........S....   O   lau   Pig
   ..H..V..K......(...).ST.....S....   O   lau   Alpaca
   ..H..V..K......(...).ST.....S....   O   lau   Bactrian camel
   S.H..V..K......(D.N).I......SV...   X   lau   Dolphin
   S.H..V..K......(D.N).I......SV...   X   lau   Killer whale
   A.N..VH.K......(..T)..K.T..S.....   O   lau   Tibetan antelope
   A.N..IH.K......(...).PN.T..VS..V.   O   lau   Cow
   A.N..VH.K......(..T)..R.T..S.....   O   lau   Sheep
   A.N..V..K......(..T)..K.T..S.....   O   lau   Domestic goat
   ..S..V.........(D..)..R.....S....   X   lau   Horse
   ..N..V....A....(D.N)..K.....S....   X   lau   White rhinoceros
   ..N..V..K......(D..)..R.....S.E..   X   lau   Cat
   ..S..V..K......(...)..V.....S....   O   lau   Dog
   A.S..V..K.D....(D..)..N.T...S....   X   lau   Ferret
   ..S..V..K......(D..)..S.S...S.A..   X   lau   Panda
   ..S..V..K......(D..)..K.....S....   X   lau   Pacific walrus
   ..S..V..K......(D..)..K.....S....   X   lau   Weddell seal
   ..N..V.........(K.N).S..L..SS...R   X   lau   Black flying-fox
   ..N..V.........(K.N).S..L..SS...H   X   lau   Megabat
   .RN..VH........(G..)..S.S..AS.KV.   X   lau   David's myotis bat
   ..N..V.......S.(V..)..S.S..AS.KD.   X   lau   Big brown bat
   V.S..V.......S.(...)..E.S..A..E..   O   lau   Hedgehog
   ..N..V.......N.(V..)..T.S...S..T.   X   lau   Star-nosed mole
   N.S.......K..A.(M.N)....R...S..P.   X   afr   Elephant
   ..S.........ID.(I.N)..V.S...S....   X   afr   Manatee
   ..N.N........D.(M.N)..L.L...ST...   X   afr   Cape golden mole
   ..S..VH......V.(..R).AA.....S...R   X   xen   Armadillo
   S.N...Q......S.(...).IS.S..ST..S.   O   mar   Opossum
   S.D.A.Q.H.P.LA.(R..).AS.S...T..SE   X   mar   Tasmanian devil
   I.N...Q.Y....S.(E..).LS.F..STVK..   X   mon   Platypus

NO 92
GN RNASE1
ID RNAS1_HUMAN
MP 104
DE Ribonuclease pancreatic
CL apes
SQ 57
   EKVTCKNGQGNCYKS(NSS)MHITDCRLTNGSR   O   hum   Human
   ...............(...).............   O   hac   Chimp
   ...............(...).............   O   aga   Gorilla
   ...............(...).........H...   O   gra   Orangutan
   .........A.....(...).............   O   ape   Gibbon
   .........T..F..(K..).............   X   cat   Rhesus macaque
   .........T..F..(K..).............   X   cat   Crab-eating macaque
   .........T..F..(K..).............   X   cat   Baboon
   .........T..F..(K..).............   X   cat   Green monkey
   .........P.....(S..).R...........   X   sim   Marmoset
   .........A.....(S..).............   X   sim   Squirrel monkey
   .R.N.....T.....(S..).D..........K   X   pri   Bushbaby
   .N......NT.....(T..).........GS.K   X   eua   Treeshrew
   .N.......T..FQ.(R.N).........G..K   X   gli   Squirrel
   .N......NS.....(H.A)L.......KGN.K   X   gli   Prairie vole
   .N.K....KS.....(H.A)L.......KGNAK   X   gli   Chinese hamster
   .N.....RKS.....(S.A)L.....H.KGN.K   X   gli   Mouse
   GQ......RN..H..(S.T)LR......KGS.K   X   gli   Rat
   KN.A.....T...Q.(S..).........SN.K   X   gli   Naked mole-rat
   RN.S.....T...Q.(Y..)....E....S..K   X   gli   Guinea pig
   KN.P.....S...Q.(..N).........SN.K   X   gli   Chinchilla
   KN.P.....T...Q.(I.N).........SN.K   X   gli   Brush-tailed rat
   ......D.KT...R.(T.K)..T...S.LDT.K   X   gli   Rabbit
   IN.N.....TXXXQ.(..T).......Q.GS.K   O   lau   Pig
   .S.......T..HQ.(T.T).......E.GS.K   X   lau   Alpaca
   KNI......Y..HQ.(..T)VN.....Q.GS..   O   lau   Bactrian camel
   KNIH....KT...E.(..T).Y..E..E.GS.K   O   lau   Dolphin
   KNIH....KT...E.(..T).Y..E..E.GS.K   O   lau   Killer whale
   KN.A.....T...Q.(Y.T).S.....E.GS.K   X   lau   Tibetan antelope
   KN.A.....T...Q.(Y.T).S.....E.GS.K   X   lau   Cow
   KN.A.....T...Q.(Y.T).S.....E.GS.K   X   lau   Sheep
   KN.A.....T...Q.(Y.T).S.....E.GS.K   X   lau   Domestic goat
   KNI......S...Q.(S..).........S..K   X   lau   Horse
   .N.I.....S...Q.(S..).R..E.Q..S..K   X   lau   White rhinoceros
   GN.......P..HR.(V.K).N....H.RR...   X   lau   Cat
   KD.L.....S..HQ.(R.Q).N......K...K   X   lau   Dog
   GN.P.....P...Q.(S.K).R......K...K   X   lau   Ferret
   KN.......PD.HQ.(S.R)........KR..K   X   lau   Panda
   GN.I.....P..HQ.(S.R).N..N...R...K   X   lau   Pacific walrus
   GN.V.....PS.HQ.(S.R)........R...K   X   lau   Weddell seal
   ......D..S...Q.(S..).S..........K   X   lau   Black flying-fox
   ......D..SD..Q.(S..).S..........K   X   lau   Megabat
   ---C....KP..H..(S..).K.....VKSS.K   X   lau   David's myotis bat
   GNII....KP..H..(S..).K.....VKSS.E   X   lau   Microbat
   GNI.....RP..H..(S..).K.....V.S..K   X   lau   Big brown bat
   GGIS...KKNK.HQ.(S..).S..T.S.FRH..   X   lau   Hedgehog
   QTIP...E.PH.H..(RDI).DL.....KRS..   X   lau   Shrew
   RNI......P...Q.(R..).R.......G...   X   lau   Star-nosed mole
   KNI......P..HQ.(...)IP.......GS.K   O   afr   Elephant
   RNM.Y.SKESS.HQ.(Y..).YN.E.H.YSSFT   X   afr   Cape elephant shrew
   .NI......PH.HQ.(..N)I........GS..   X   afr   Manatee
   GNI......S..HQ.(S.A)............K   X   afr   Cape golden mole
   GSI......S..HR.(G.R)V.......KG.AK   X   afr   Tenrec
   KS.....K.P..HQ.(...).........S..K   O   afr   Aardvark
   TNI......S...Q.(SQP).SL.H..Q.GA.K   X   mar   Opossum
   T.AP..TENS..HR.(RKL).QVIE...KGN.K   X   mar   Tasmanian devil
   KNI......PS.HQ.(SLP).IL.H..Q.GA.K   X   mar   Wallaby

NO 93
GN RNASE1
ID RNAS1_HUMAN
MP 116
DE Ribonuclease pancreatic
CL primates
SQ 58
   YKSNSSMHITDCRLT(NGS)RYPNCAYRTSPKE   O   hum   Human
   ...............(...).............   O   hac   Chimp
   ...............(...).............   O   aga   Gorilla
   ...............(H..).............   X   gra   Orangutan
   ...............(...).............   O   ape   Gibbon
   F..K...........(...).............   O   cat   Rhesus macaque
   F..K...........(...).............   O   cat   Crab-eating macaque
   F..K...........(...).............   O   cat   Baboon
   F..K...........(...).............   O   cat   Green monkey
   ...S...R.......(...)..........Q..   O   sim   Marmoset
   ...S...........(...)..........Q..   O   sim   Squirrel monkey
   ...S...D.......(...)K....S....Q.K   O   pri   Bushbaby
   ...T...........(GS.)K.........Q.Q   X   eua   Treeshrew
   FQ.R.N.........(G..)K....S.K..Q..   X   gli   Squirrel
   ...H.AL.......K(GN.)K....D.Q.NQLQ   X   gli   Prairie vole
   ...H.AL.......K(GNA)K....D.Q..QHQ   X   gli   Chinese hamster
   ...S.AL.....H.K(GN.)K....D.K.TQYQ   X   gli   Mouse
   H..S.TLR......K(GS.)K....D.T.TDSQ   X   gli   Rat
   .Q.S...........(SN.)K..T.S....QM.   X   gli   Naked mole-rat
   .Q.Y......E....(S..)KF...S....QAQ   X   gli   Guinea pig
   .Q...N.........(SN.)K....S....REN   X   gli   Chinchilla
   .Q.I.N.........(SN.)K..D.S...RQE.   X   gli   Brush-tailed rat
   .R.T.K..T...S.L(DT.)K..D.K.Q.VQ..   X   gli   Rabbit
   XQ...T.......Q.(GS.)K......KA.QQ.   X   lau   Pig
   HQ.T.T.......E.(GS.)K......KA.NLQ   X   lau   Alpaca
   HQ...TVN.....Q.(GS.).....V.K.TKLQ   X   lau   Bactrian camel
   .E...T.Y..E..E.(GS.)K......K..Q..   X   lau   Dolphin
   .E...T.Y..E..E.(GS.)K......K..Q..   X   lau   Killer whale
   .Q.Y.T.S.....E.(GS.)K......K.TQAK   X   lau   Tibetan antelope
   .Q.Y.T.S.....E.(GS.)K......K.TQAN   X   lau   Cow
   .Q.Y.T.S.....E.(GS.)K......K.TQA.   X   lau   Sheep
   .Q.Y.T.S.....E.(GS.)K......K.TQA.   X   lau   Domestic goat
   .Q.S...........(S..)K.........Q..   X   lau   Horse
   .Q.S...R..E.Q..(S..)K..S......QM.   X   lau   White rhinoceros
   HR.V.K.N....H.R(R..)...K.T.E.TQED   X   lau   Cat
   HQ.R.Q.N......K(...)KF.K.V.T.TQ..   O   lau   Dog
   .Q.S.K.R......K(...)K..K.D.Q.QQLQ   O   lau   Ferret
   HQ.S.R........K(R..)K..K.E.Q.EQ..   X   lau   Panda
   HQ.S.R.N..N...R(...)K....K.Q.TQE.   O   lau   Pacific walrus
   HQ.S.R........R(...)K..K.Q.Q.MQ..   O   lau   Weddell seal
   .Q.S...S.......(...)K.........Q.K   O   lau   Black flying-fox
   .Q.S...S.......(...)K.........Q.K   O   lau   Megabat
   H..S...K.....VK(SS.)K..D.D.HI.H..   X   lau   David's myotis bat
   H..S...K.....VK(SS.)E..F.D.E..H..   X   lau   Microbat
   H..S...K.....V.(S..)K.........Q..   X   lau   Big brown bat
   HQ.S...S..T.S.F(RH.)...K.I.K.TR..   X   lau   Hedgehog
   H..RDI.DL.....K(RS.).DG....Q.TRRQ   X   lau   Shrew
   .Q.R...R.......(G..)..........Q..   X   lau   Star-nosed mole
   HQ....IP.......(GS.)K.........N.M   X   afr   Elephant
   HQ.Y...YN.E.H.Y(SSF)T..K.G.WA.-.M   X   afr   Cape elephant shrew
   HQ...NI........(GS.)...S...WP.N.M   X   afr   Manatee
   HQ.S.A.........(...)K.......ATN.M   O   afr   Cape golden mole
   HR.G.RV.......K(G.A)K..K.E...TT.M   X   afr   Tenrec
   HQ.............(S..)K......Q..NMM   X   afr   Aardvark
   HEG---VKV...KE.(GS.).T...R..AMS--   X   xen   Armadillo
   .Q.SQP.SL.H..Q.(GA.)K....Q..G.DT-   X   mar   Opossum
   HR.RKL.QVIE...K(GN.)KF.H.K.Q.MN.-   X   mar   Tasmanian devil
   HQ.SLP.IL.H..Q.(GA.)K....Q..A.-YN   X   mar   Wallaby

NO 94
GN SERPINA6
ID CBG_HUMAN
MP 31
DE Corticosteroid-binding globulin
CL simians
SQ 57
   GLWTVQAMDPNAAYV(NMS)NHHRGLASANVDF   O   hum   Human
   ...............(...).............   O   hac   Chimp
   ...............(.T.).............   O   aga   Gorilla
   ...........T...(...).............   O   gra   Orangutan
   ..............M(...)........T....   O   ape   Gibbon
   ..............R(...).......AV.A..   O   cat   Rhesus macaque
   ..............R(...).......AV.A..   O   cat   Crab-eating macaque
   ..............R(...)S......AV.A..   O   cat   Baboon
   ..............R(...).......AV....   O   cat   Green monkey
   ...N..........M(...)R.--D...V....   O   sim   Marmoset
   ..............M(.T.)R...V...V.A..   O   sim   Squirrel monkey
   ....I..EHSS.--.(.AK).......P.....   X   pri   Bushbaby
   C...I..Q.SA.N-.(.IR)SP.....PT....   X   eua   Treeshrew
   ....I..E.....-I(STK)SP..D..PI.T..   X   gli   Squirrel
   ..........S.V-.(SAR)SP.....PT....   X   gli   Lesser Egyptian jerboa
   ..V.ANP.------S(HTW).P.....PT....   X   gli   Prairie vole
   ..VST.N..-----R(SAM)SS.....PT....   X   gli   Chinese hamster
   ....A..-------T(.E.)SS.....PT....   O   gli   Rat
   ...AI..ETSS.V-S(GID)SP..D..PT.L..   X   gli   Naked mole-rat
   ....A..E..G.I-S(STE)SP.....PT....   X   gli   Guinea pig
   ...AI..E.SS.V-S(STE)S......PT.A..   X   gli   Chinchilla
   S..AN..E.ASTV-S(GTE)S.S....PT....   X   gli   Brush-tailed rat
   .H.A...A..PGGDI(CTR)SP.....P.....   X   gli   Rabbit
   ....T..E..SDDGI(YTR)SP.....P.....   X   gli   Pika
   ....S..-..DSD-L(STR)SR..N..PN....   X   lau   Pig
   ....I..-.SD.G-.(SER).P..D..PS.T..   X   lau   Alpaca
   ....I..-.SD.V-.(SKR).P..D..PS.T..   X   lau   Bactrian camel
   ....I..-..DTD-M(S.R).P..H..PN....   X   lau   Dolphin
   ....I..-..DTD-M(S.R).P..H..PN....   X   lau   Killer whale
   .......-GSD.D-.(STR).S..D..PN....   X   lau   Tibetan antelope
   .......-GSDTD-M(STR).P..D..PN....   X   lau   Cow
   .......-GTDTD-.(STR).P..D..PN....   X   lau   Sheep
   ...A...-GSGTD-.(STR).P..D..PN....   X   lau   Domestic goat
   ....I..-..DGD-M(SIR)IP..D..PS....   X   lau   Horse
   ....I..-..D.D-M(STR)SP..E..PG....   X   lau   White rhinoceros
   D......-..DTD-.(STR)SP..D..PN....   X   lau   Cat
   Y.L....-..DTD-.(ST.)SS..D..PK....   X   lau   Dog
   D......-..DTD-.(STR)SP..D..PN....   X   lau   Ferret
   D......-..DTD-.(SPR)TP..D..PS....   X   lau   Panda
   D..A...-..DTD-L(STR)SP..D..PK....   X   lau   Pacific walrus
   D..A...-..DTD-.(STR)SL..D..PK.I..   X   lau   Weddell seal
   .......-...VN-.(S.R)SY..D..PS....   X   lau   Black flying-fox
   ....A..-.SE.D-M(SVR)SQ..D..PN....   X   lau   David's myotis bat
   ....A..-.SD.D-M(SVR)SQ..D..PN....   X   lau   Microbat
   ...A...-.SD.D-T(SLR)SQ..V..PN....   X   lau   Big brown bat
   SPQ.A..-------L(DT.)RDE.Q..PN.A..   X   lau   Hedgehog
   ..Q.I..-.SHEN-M(STR)SPY.N..PN.A..   X   lau   Shrew
   .....HSEVSDSG--(STR)GP..NI.PN....   X   lau   Star-nosed mole
   .F.GTR.-..SLD-.(DVR)SP.....P.....   X   afr   Elephant
   ...A...-..ISD-N(DQR)TP.....P.....   X   afr   Cape elephant shrew
   .FRGI..-N.SHD-.(..R)SP.....P.....   X   afr   Manatee
   ...GI..-...PE-.(DTR)RP..S..PT....   X   afr   Cape golden mole
   -..GIR.-..DPD-M(SLR)D--....L.....   X   afr   Tenrec
   S.RAI..-.S.VD-.(S..)RP.WD..L..T..   X   xen   Armadillo
   .SCVP.P-.FSHSLR(SKK)IL..R..PV....   X   mar   Opossum
   SFCVS.T-.LQNPLM(RKK)ILY.K..PV....   X   mar   Tasmanian devil
   SFCVSHP-..KNPLL(RKK)IL..R..PV.A..   X   mar   Wallaby

NO 95
GN SERPINA6
ID CBG_HUMAN
MP 369
DE Corticosteroid-binding globulin
CL catarrhines
SQ 60
   NEEGVDTAGSTGVTL(NLT)SKPIILRFNQPFI   O   hum   Human
   ...............(...).............   O   hac   Chimp
   ...............(...).............   O   aga   Gorilla
   ...............(...).............   O   gra   Orangutan
   T..............(...)..T.V........   O   ape   Gibbon
   S............S.(...).............   O   cat   Rhesus macaque
   S............S.(...).............   O   cat   Crab-eating macaque
   S............S.(...).............   O   cat   Baboon
   S............S.(...).............   O   cat   Green monkey
   S......T..S....(.VM).RR..M......L   X   sim   Marmoset
   S....N.T.......(.PM).....M......L   X   sim   Squirrel monkey
   D....QASA.RV..P(Y..).S..NVS..R..L   X   pri   Bushbaby
   D.R.MKA.AH.E.SP(EQS).E.HTIL.....V   X   eua   Treeshrew
   D..DMKP.AT--G..(E.E).E.LTIN..R..L   X   gli   Squirrel
   D.R.MAPTAP.KNA.(P..).E.VT...DR..L   X   gli   Lesser Egyptian jerboa
   D.T.EPIRPTR.AP.(EV.).E.LTVK..K...   X   gli   Prairie vole
   D.TDELPVAI.KAPQ(QQ.).D.LT.T..E...   X   gli   Chinese hamster
   D.KDEPPVTT.EAPP(QT.).E.LT.T..K...   X   gli   Golden hamster
   D.GN.LP.ATN.PPV(H.P).ESFT.KY.R...   X   gli   Mouse
   D.GN.LPNSTN.AP.(H.R).E.LDIK..K...   X   gli   Rat
   DQDR.--.ANP.S.P(K.E).E.VT..L.W..L   X   gli   Naked mole-rat
   D.SNAMKPAPA-.II(KQE)YEAVTFE..H..F   X   gli   Guinea pig
   D.NN.KKVADP...V(KQE).E.VTFN..H..L   X   gli   Chinchilla
   D.R.RKE.ADP...V(KQE).E.VTFA.DH..L   X   gli   Brush-tailed rat
   D.Q..EV.A--.GP.(Q.V).E.LT.N..R..L   X   gli   Rabbit
   D.K.AEA.V.P.R..(Q.V).E.LTIS..R..L   X   gli   Pika
   D.K.MEA.AP.T.S.(HAA)P..VTVH..R...   X   lau   Pig
   D.K.LEAVAP.K.S.(QGA)PD.LTIH..R...   X   lau   Alpaca
   D.K.LEAVAY.K.G.(QGA)PD.LTIH..R...   X   lau   Bactrian camel
   D.R.LES.AR.Q.HR(.SA)PE.LTI..DR..V   X   lau   Dolphin
   D.R.LES.AR.Q.HR(.SA)PE.LTI..DR..V   X   lau   Killer whale
   D.K.LEA.AP.R.SV(SAA)PG.LT....R...   X   lau   Tibetan antelope
   D.K.LEADAP.R.S.(SAA)PG.LT....R...   X   lau   Cow
   D.K.LEA.AP.R.SV(SAA)PG.LT....R...   X   lau   Domestic goat
   D.K.EES.AP..IPP(.VG)...LTI...R...   X   lau   Horse
   D.K.ARANAPAAI.A(HAV).E.LTV..DR..L   X   lau   White rhinoceros
   S.S.LEA.APS.LMP(.AA)...LAVHI.R...   X   lau   Cat
   H.K.LEA..PA..MP(.VK).E.LAFH..R...   X   lau   Dog
   D.K.LEG.D.PR.M.(HTA)PE.LTF...R...   X   lau   Ferret
   D.K.LEA.TCPR.M.(EGA).E.LTF..DR..L   X   lau   Panda
   D.KSFMAPEFPM.M.(DMA).E.LTFH..R...   X   lau   Pacific walrus
   D.KSFKA.EFPM.M.(DMA).E.LTFH..R...   X   lau   Weddell seal
   D.K..QE.AP...S.(KVE).E..TIH..R...   X   lau   Black flying-fox
   D.Q..QE.AP...S.(KVE).E.LTIH..R...   X   lau   Megabat
   D.K..EE.AP....Q(.EA).E.LTIH..R...   X   lau   David's myotis bat
   D.K..EE.AP.R.SR(.EA).E.LTIH..R...   X   lau   Microbat
   D.K.AEE.AR..A.Q(.VA).E.LSI...R..V   X   lau   Big brown bat
   D.Q.AEP.PP.--SK(KSD)PE..TVQ.....M   X   lau   Hedgehog
   T.K.KEA.V..RE.-(RG.)AQLTKIQ..R...   X   lau   Shrew
   A....KD.AP.Q.PK(DMA)AE.LTVI..R...   X   lau   Star-nosed mole
   D.T..KA.IAP.GG.(K.M)TL.HSIK..R...   X   afr   Elephant
   D.T.IKG.VASEGGQ(K.M)NS.YYFKL.R...   X   afr   Cape elephant shrew
   E.T..KA.AAPMGG.(K.M)VR.RSIK..R...   X   afr   Manatee
   D.T.TKP.TTP.GAP(..R)AL.P.IK..R..V   X   afr   Cape golden mole
   D.A..GP.AAPAEA.(..R)PL.P.IK....LL   X   afr   Tenrec
   D.K..KA.AASEDGR(R.M)AMSGSVK..R..V   X   afr   Aardvark
   H.K..MP.AT..GAQ(R.E)VP.LVIK..R..L   X   xen   Armadillo
   D...TEA.AG..MRF(FFK).ALPVVK..K..L   X   mar   Opossum
   D...MEA.AG.ALSI(RVK).YRPVIK.DR..L   X   mar   Tasmanian devil
   D...TEE.AG..SNV(VFD).YHPVIK.DR..L   X   mar   Wallaby

NO 96
GN SLC1A5
ID AAAT_HUMAN
MP 212
DE Neutral amino acid transporter B(0)
CL simians
SQ 50
   VSAAFRSYSTTYEER(NIT)GTRVKVPVGQEVE   O   hum   Human
   ...............(...).............   O   hac   Chimp
   ...............(...).............   O   aga   Gorilla
   ...............(...).............   O   gra   Orangutan
   ...............(...)..T..........   O   ape   Gibbon
   ..........S....(...).............   O   cat   Rhesus macaque
   ..........S....(...).............   O   cat   Baboon
   ..........S....(.V.).............   O   cat   Green monkey
   ........A.S....(...)..L..--------   O   sim   Marmoset
   ........A.S.K..(.T.)..L....M.E...   O   sim   Squirrel monkey
   ..........S.RTI(.VN)..T..I...E...   X   pri   Bushbaby
   ..........S..HI(HVN)..K...VL.....   X   eua   Treeshrew
   ...T......S.QWI(K.N)..L..E.N.K...   X   gli   Squirrel
   ...T....A.K....(RLN)TAMAQ..MES...   X   gli   Lesser Egyptian jerboa
   .......QTVPCTK.(E.N)A.M.Q---.C...   X   gli   Prairie vole
   .......LT.DCAH.(ETN)A.L.Q---.G...   X   gli   Chinese hamster
   ......TQT.SCSW.(EYN)A.MIQ---.C.E.   X   gli   Golden hamster
   ........T....DG(LCN)H.KG..--.K.E.   X   gli   Naked mole-rat
   .....K..T.V.QH.(PFN)H.M....L.M..D   X   gli   Guinea pig
   .....S..T.I..D.(PFS)N.T......K..D   X   gli   Chinchilla
   .....S..A.A..D.(PFN)H.I......K..D   X   gli   Brush-tailed rat
   ..........S...K(.FN)..L......H.E.   X   gli   Rabbit
   .........SYCDVQ(DFN)E.MG.M.E.C...   X   gli   Pika
   ........A.S.S..(PFN)..V......A...   X   lau   Pig
   ........A.S.K.I(PFD)..L......G...   X   lau   Alpaca
   ........A.S.K.I(PFD)E.L......G...   X   lau   Bactrian camel
   ........A.S.K..(LFN)..Q....M.G...   X   lau   Dolphin
   ........A.S.K..(LFN)..Q....M.G...   X   lau   Killer whale
   ........T.S.K.M(LFN).SL....T.G...   X   lau   Tibetan antelope
   ........T.S.K..(LFN)..L....T.G...   X   lau   Cow
   ........T.S.K.I(LFN).SP....T.G...   X   lau   Sheep
   ........T.S.K.I(LFN).SP....T.G...   X   lau   Domestic goat
   ........A.S..QS(S.N)..Q......G...   X   lau   Horse
   ........A.S..QI(W.N)..Q......G...   X   lau   White rhinoceros
   ..........V..S.(W.N)..K......D..D   X   lau   Cat
   .............H.(W.N)..M....I.E...   X   lau   Dog
   ..........S.DT.(S.N)..K......T...   X   lau   Ferret
   ..........S..N.(W.N)..K....I.R...   X   lau   Panda
   ..........S..N.(W.N)..K....I.D...   X   lau   Pacific walrus
   ..........S..N.(W.N)..K....I.D...   X   lau   Weddell seal
   ..........S.KVI(RNN)E.Q......D...   X   lau   Black flying-fox
   ..........S.KMI(RNN)E.Q......D...   X   lau   Megabat
   ....SP...AS.--K(S.N)..L....K.D...   X   lau   Hedgehog
   ..........S.VPQ(..S)..LM...Q.K...   O   lau   Shrew
   ..........S...K(E.S).KL....IET...   X   lau   Star-nosed mole
   ........T.S..M.(R.N)..L....L.G...   X   afr   Elephant
   ........A.H..I.(KVN)E.L....L.G...   X   afr   Manatee
   ........A.S..MK(SVN)S.L......D...   X   afr   Cape golden mole
   ........T....MK(A.N)..L....W.G...   X   afr   Aardvark
   ..........QW.MK(WVN)R.S..---TG...   X   xen   Armadillo

NO 97
GN SLC3A2
ID 4F2_HUMAN
MP 381
DE 4F2 cell-surface antigen heavy chain
CL simians
SQ 55
   ITKGFSEDRLLIAGT(NSS)DLQQILSLLESNK   O   hum   Human
   ...............(...).............   O   hac   Chimp
   ...............(...).............   O   aga   Gorilla
   ...............(...).............   O   gra   Orangutan
   ...............(...).............   O   ape   Gibbon
   ...............(...).....V.......   O   cat   Rhesus macaque
   ...............(...).....V.......   O   cat   Crab-eating macaque
   ...............(...).....V.......   O   cat   Baboon
   ...............(...).....V.......   O   cat   Green monkey
   ...............(...).............   O   sim   Marmoset
   ...............(...).............   O   sim   Squirrel monkey
   ...S.G.........(DY.).....S...K.T.   X   pri   Bushbaby
   ...S...A.......(...)...........T.   O   gli   Lesser Egyptian jerboa
   ...S...........(D..).......I...TS   X   gli   Prairie vole
   ...N...........(D..).....VTI...TS   X   gli   Chinese hamster
   ...NL..........(E..).....VNI...TS   X   gli   Mouse
   ...N...........(A..).....VNI...TS   X   gli   Rat
   ...SS.K...F....(...)NM..L......TQ   O   gli   Naked mole-rat
   ...S..K.K......(...)....L......T.   O   gli   Guinea pig
   VI.SS.T....V...(..F)....LV...N.TQ   X   gli   Chinchilla
   ..RSS.K........(...)....L......TQ   O   gli   Brush-tailed rat
   M..S.K.....LV..(G..)G.RPT.D....T.   X   gli   Pika
   L..S...........(D..)..........PT.   X   lau   Pig
   ...S...........(D..)...........T.   X   lau   Alpaca
   ...S...........(D..)...........T.   X   lau   Bactrian camel
   ...SV..........(D..)......R...PT.   X   lau   Tibetan antelope
   ...SV..........(D..)......R...PTR   X   lau   Cow
   ...SV..........(D..)......R...PT.   X   lau   Sheep
   ...SV..........(D..)......R...PT.   X   lau   Domestic goat
   M..NI..........(E..)..H..R.....T.   X   lau   Horse
   ...S...........(E..).........D.T.   X   lau   White rhinoceros
   ...S....---....(E..).........G.T.   X   lau   Cat
   V..S...........(E..).........G.T.   X   lau   Dog
   M..S....----...(E..)..R......G.T.   X   lau   Ferret
   V..S...........(E..).........G.T.   X   lau   Panda
   M..S...........(EF.).........G.A.   X   lau   Pacific walrus
   M..S...........(EF.).........G.T.   X   lau   Weddell seal
   ..GSV.G.K......(GF.)T.E......KFT.   X   lau   Black flying-fox
   ..GSV.G.K......(GF.)T.E......KFT.   X   lau   Megabat
   ...S.G.....M...(K..)S..E.V......N   X   lau   David's myotis bat
   ...S.G..---L...(K..)N..E.V...D..S   X   lau   Microbat
   ...S...........(G..)...E......PA.   X   lau   Big brown bat
   ...N.....I....M(K.E).P...........   X   lau   Hedgehog
   ...N....S......(...)..A...H..SVSN   O   lau   Shrew
   ...S.N.........(G.A)N.SV.QE..NH--   X   lau   Star-nosed mole
   ..Q............(D..)......K..D.TR   X   afr   Elephant
   ..QRV...K.....M(...)...K..Q..N.--   O   afr   Cape elephant shrew
   ..QN...........(D..)......K..N.TE   X   afr   Manatee
   ..QSV..E.......(D..)N..E..E..N.TS   X   afr   Cape golden mole
   ..QS.N.EK..M...(D..)......K..N.T.   X   afr   Tenrec
   ..QSS..........(GF.)......K..D.T.   X   afr   Aardvark
   ..QS.N..------.(...).........G.T.   O   xen   Armadillo
   L.HSI...NV..V..(EA.)E.S.LM...NDTD   X   mar   Opossum
   F.H.IG...VF....(EA.)E.SA.M...NDTD   X   mar   Tasmanian devil
   L.HSI.D.KV..V..(GA.)..SE.....NDTD   X   mar   Wallaby

NO 98
GN SLC4A7
ID S4A7_HUMAN
MP 791
DE Sodium bicarbonate cotransporter 3
CL humans and chimpanzees
SQ 62
   NETLAQWKKDNITAH(NIS)WRNLTVSECKKLR   O   hum   Human
   ..............D(...)............H   O   hac   Chimp
   ...............(S..)............H   X   aga   Gorilla
   .........E.....(...)............H   O   gra   Orangutan
   .........E.....(...)............H   O   ape   Gibbon
   .........E.....(...)............H   O   cat   Rhesus macaque
   .........E.....(...)............H   O   cat   Crab-eating macaque
   .........E.....(...)............H   O   cat   Baboon
   .........E.....(...)............H   O   cat   Green monkey
   .........E.....(..P)...........VH   X   sim   Marmoset
   .........E.....(..P).S.........VH   X   sim   Squirrel monkey
   ....EL.N.E.....(S..).G........TFH   X   pri   Bushbaby
   ....QL...E.K...(T..).G........SFH   X   eua   Treeshrew
   ....EL...E.....(S..).G........TFH   X   gli   Squirrel
   .D.VEL..RE.M...(DV.).G.....K..AYH   X   gli   Lesser Egyptian jerboa
   ...IEL..RK.V..E(S.V).G........TFH   X   gli   Prairie vole
   ...IEL..RK.V..D(S.L).G........AFH   X   gli   Chinese hamster
   ...IEL..RK.V.S.(S.F).G........AFH   X   gli   Golden hamster
   ....EL..RK....Y(SV.).G........TFH   X   gli   Mouse
   ...VEL.ERK.V..A(S..).A........TFH   X   gli   Rat
   .....L...K.L...(S..)......ID..TFH   X   gli   Naked mole-rat
   .D..VL.*NKYLS..(DT.)..........TFH   X   gli   Guinea pig
   .....L..NK.L.S.(G..)....S.....SFH   X   gli   Chinchilla
   S....L..EK.L.V.(G..).P....P...T.H   X   gli   Brush-tailed rat
   ....EL...E.....(...).G....P...TFH   O   gli   Rabbit
   ....EF.Q.E.....(S..).G.......AA..   X   gli   Pika
   ....KV.EER.V..R(D.P).G........SFH   X   lau   Pig
   ....KV.EER.V..R(D.P).G........TFH   X   lau   Alpaca
   ....KV.EER...VR(D.P).G........TFH   X   lau   Bactrian camel
   ....KV.EER.V...(..P).G........IFH   X   lau   Dolphin
   ....KV.EER.V...(..P).G........IFH   X   lau   Killer whale
   ....KM.EEE.V..R(D.P).G........VYH   X   lau   Tibetan antelope
   ....KM.E.N.V..R(D.P).G........VYH   X   lau   Cow
   ....KM.EEK.V..R(D.P).G.......RAY.   X   lau   Sheep
   ....KM.EEK.V..R(D.P).G........VYH   X   lau   Domestic goat
   ....KM..EK.V...(D..).G........TFH   X   lau   Horse
   ....KV..EK.V...(E..).G........TFH   X   lau   White rhinoceros
   ....KV..EK.L...(D..).E........TFH   X   lau   Cat
   ....KI..EM.L...(D..).G........TFH   X   lau   Dog
   ....KI..EK.L..D(D.F).G.......RTFH   X   lau   Ferret
   ....KI.QEK.L..D(D.F).G........TFH   X   lau   Panda
   ....KI..EK.L..D(D.F).G........TFH   X   lau   Pacific walrus
   ....KI..EK.L..D(D.F).G....P...TFH   X   lau   Weddell seal
   ....NA..EK.L...(D..).G........TFH   X   lau   Black flying-fox
   ....NV..EK.L...(D..).G........TFH   X   lau   Megabat
   ....KV.TEK.....(D..).G........TFH   X   lau   David's myotis bat
   ....KV.TEK.....(D..).G........TFH   X   lau   Microbat
   ....KV.TEN.....(D..).G........TFH   X   lau   Big brown bat
   .V..KM.R.N...TN(D.N).S........AFH   X   lau   Hedgehog
   ....MV..EK....D(GV.).E........AI.   X   lau   Shrew
   .A..ML.EEK.V..S(D.L).G........AFH   X   lau   Star-nosed mole
   ....EL..EKHV..R(D..).G.......QAFH   X   afr   Elephant
   ....KL..EKH..TD(..T).E....T...E.H   O   afr   Cape elephant shrew
   ....EL..EKHV..R(...).G........TFH   O   afr   Manatee
   ....EL..QK....N(S..).E.....K..TYH   X   afr   Cape golden mole
   ....EL..ERHL..E(...).G.....Q..TFH   O   afr   Tenrec
   ....EL..AKH.A..(...).G........SFH   O   afr   Aardvark
   ....EL..EK.....(...).G.....D..LYH   O   xen   Armadillo
   ....RK.MEM.HS.Q(T.P).G.....K..TFH   X   mar   Opossum
   ....KR.IEM.KS.Q(T..).G........T.H   X   mar   Tasmanian devil
   ....KK.REM.QST.(T..).G....K...EFH   X   mar   Wallaby
   ...IEM...I.K.GD(T..).G...I....TYH   X   mon   Platypus

NO 99
GN SMPDL3A
ID ASM3A_HUMAN
MP 263
DE Acid sphingomyelinase-like phosphodiesterase 3a
CL great apes
SQ 62
   YIIAHVPVGYLPSSQ(NIT)AMREYYNEKLIDI   O   hum   Human
   ..V............(...).............   O   hac   Chimp
   ...............(...).............   O   aga   Gorilla
   ...............(...).............   O   gra   Orangutan
   ...............(H..).V...........   X   ape   Gibbon
   ............Y..(...).I...........   O   cat   Rhesus macaque
   ............Y..(...).I...........   O   cat   Crab-eating macaque
   ............Y..(...).I...........   O   cat   Baboon
   ............Y.K(...).I...........   O   cat   Green monkey
   ............Y.K(S..).I...........   X   sim   Marmoset
   ............Y.K(S..).............   X   sim   Squirrel monkey
   ............QVK(.T.).I..A......N.   O   pri   Bushbaby
   ............FPG(SN.).I.G.........   X   eua   Treeshrew
   .....I......F.S(G..)...Q.........   X   gli   Squirrel
   ............Y.S(G.P)...QC.....VN.   X   gli   Lesser Egyptian jerboa
   ............YAT(ATP).V.Q......VE.   X   gli   Prairie vole
   ............YAT(DTP).V.Q......V..   X   gli   Chinese hamster
   ............YAT(DTP).V.Q......V..   X   gli   Golden hamster
   ............YAT(DTP).I.Q......L..   X   gli   Mouse
   .V..........YAT(KTP)...Q......V..   X   gli   Rat
   FL..........Q.T(G..).V.Q....R.VAL   X   gli   Naked mole-rat
   ............Y.S(G..)...Q......VEL   X   gli   Guinea pig
   ............Y.S(G..).I.Q......VEL   X   gli   Chinchilla
   .......L....F.S(G..)...Q....R.VEL   X   gli   Brush-tailed rat
   ............Y.N(.T.).I........V..   O   gli   Rabbit
   ............F.R(S..).V........V..   X   gli   Pika
   ............Y.K(ST.).............   X   lau   Pig
   ............F.R(S..)...........E.   X   lau   Alpaca
   ............F.R(S..)...........E.   X   lau   Bactrian camel
   ............Y.R(S..).............   X   lau   Dolphin
   ............Y.R(S..).............   X   lau   Killer whale
   ............FAK(G.P)...KCH....T..   X   lau   Tibetan antelope
   ............YAR(G.S)...K.H.......   X   lau   Cow
   ............FAK(G.P)...KCH.......   X   lau   Sheep
   ............FAK(G.P)...QCH.......   X   lau   Domestic goat
   .....I......YTV(GT.).............   X   lau   Horse
   .....I......H.M(.T.)...........E.   O   lau   White rhinoceros
   .....I....V.H.R(...)S...L........   O   lau   Cat
   .....I......Y.G(GTM)....F......E.   X   lau   Dog
   .....I......Y.S(ST.)....FH.....N.   X   lau   Ferret
   .....I......YLS(ST.)....FH.......   X   lau   Panda
   .....I......Y.S(ST.)....FH....V..   X   lau   Pacific walrus
   .....I......Y.G(ST.)....VH.......   X   lau   Weddell seal
   .....I.M....Y.M(ST.).L......R.V..   X   lau   Black flying-fox
   .....I.M....Y.M(ST.).L......R.V..   X   lau   Megabat
   .....I......Y.M(GT.)....L......G.   X   lau   David's myotis bat
   .....I......Y.M(GT.)....L........   X   lau   Microbat
   .....I......F.M(GT.)....F......G.   X   lau   Big brown bat
   ............YAN(T..)...........N.   X   lau   Hedgehog
   ............Y.K(GT.)...........E.   X   lau   Shrew
   .....I......FTR(GT.)...........N.   X   lau   Star-nosed mole
   .L..........F.R(ST.).............   X   afr   Elephant
   .L..........Y.R(ST.)...........N.   X   afr   Cape elephant shrew
   .L...I......F.R(.T.).............   O   afr   Manatee
   ............FVR(ST.)V......K...G.   X   afr   Cape golden mole
   .L..........FT.(.T.).L...........   O   afr   Tenrec
   .L...I......Y.R(ST.).............   X   afr   Aardvark
   .....I........K(S..)..........L..   X   xen   Armadillo
   .V..........Y.R(.T.)...........G.   O   mar   Opossum
   .V.G........Y.R(.T.).I.........G.   O   mar   Tasmanian devil
   .V..........C.R(.T.).............   O   mar   Wallaby
   .V..........YVS(.T.)........R.VST   O   mon   Platypus

NO 100
GN SPARCL1
ID SPRL1_HUMAN
MP 412
DE SPARC-like protein 1
CL simians
SQ 59
   GTTEPGEHQEAKKAE(NSS)NEEETSSEGNMRV   O   hum   Human
   ..S............(...).............   O   hac   Chimp
   ..S............(...).............   O   aga   Gorilla
   D.S.....E......(...).............   O   gra   Orangutan
   D.S............(...).D..M........   O   ape   Gibbon
   D.S............(...).D...........   O   cat   Rhesus macaque
   D.S............(...).D...........   O   cat   Crab-eating macaque
   D.S............(...).D...........   O   cat   Baboon
   D.S............(...).D...........   O   cat   Green monkey
   D.S....Y......D(...).DD..........   O   sim   Marmoset
   D.S....Y......D(...).DD..........   O   sim   Squirrel monkey
   .ASG.A..P.V....(R..).GDDM.....T..   X   pri   Bushbaby
   D.SD..HY.G.....(S..)..--.A...STQL   X   eua   Treeshrew
   D.S.H.DY.G...VG(S..)..DD..T...VE.   X   gli   Squirrel
   D.S.AEDY.G.....(SP.)T.D...K..SSG.   X   gli   Lesser Egyptian jerboa
   DKS.A.DN.R.....(S.P)..--P.D...S.G   X   gli   Prairie vole
   DKS.A.DN.G.....(SLP).G--P.D...S.G   X   gli   Chinese hamster
   DKS.A.DD.G...VK(SLP).G--P.D...S.G   X   gli   Golden hamster
   SENQAKIQGR.....(S.P)..--P.D...S.E   X   gli   Mouse
   .DNQ------.....(S.P)..--P.D...S.G   X   gli   Rat
   D.S...D..G....Q(S..).....W..DHE..   X   gli   Naked mole-rat
   DAS..AD...D.N.Q(S..)....A....H..A   X   gli   Guinea pig
   DAH.S.D.......Q(S..).....A...HV..   X   gli   Chinchilla
   DAS...D.....Q.Q(S..)....SA...HVWM   X   gli   Brush-tailed rat
   D.S...DY.G.....(S..)..D.......V..   X   gli   Rabbit
   D.S...DRHG.....(S..)..D.A...D.V..   X   gli   Pika
   D.G.AA.N.G.....(G.P)..DDN.T..TT.G   X   lau   Pig
   D.SA.V.N.G...V.(S.L)..DDS.T.....M   X   lau   Alpaca
   D.SA.V.N.G...V.(S.L)..DDS.T.....M   X   lau   Bactrian camel
   D.S..V.NRG.....(S.L)..DDR.T...T..   X   lau   Dolphin
   D.S..V.NRG.....(S.L)..DDH.T...T..   X   lau   Killer whale
   D.S..V.N.R.....(T.T)K.--S.T...R..   X   lau   Tibetan antelope
   DSS.TV.N.Q.....(S.L)K.D.H.T...T..   X   lau   Cow
   D.S..V.N.R.....(S.P)K.--S.T...R..   X   lau   Sheep
   D.S..V.N.R.....(S.P)K.--S.A...R..   X   lau   Domestic goat
   D.N.........T..(S.P)I.D.S........   X   lau   Horse
   D.SK.....GS...D(SLP)..D.S........   X   lau   White rhinoceros
   DAS....Y.G...T.(SLP)..G.S.Y.D.RM.   X   lau   Cat
   DAS....Y.G.....(S.P)..D.S.Y.N.RM.   X   lau   Dog
   DAS....Y.......(S.P)K.D.S.Y.D.RM.   X   lau   Ferret
   DAG......G.G...(S.L)K.D.R.Y.D.RM.   X   lau   Panda
   ..S...AY.G...T.(S.P)K.D.S.Y.D.RM.   X   lau   Pacific walrus
   D.S....Y.G...T.(S.P)K.D.S.Y.D.RM.   X   lau   Weddell seal
   ..S....Y.G.....(S.L)..D.S......GL   X   lau   Black flying-fox
   ----------.....(S.L)..D.S......GL   X   lau   Megabat
   DAS....S.G.....(S.L)..D.S.T...I..   X   lau   David's myotis bat
   DAS....S.G.....(S.L)..D.S.T...F..   X   lau   Microbat
   DVS....SEG.....(T.L)..D.S.T...I..   X   lau   Big brown bat
   DAR..E...G...P.(..P)..DGS....SA.Q   X   lau   Hedgehog
   -SS.A.Q.HG.....(S.A)P.DGG....DS.L   X   lau   Shrew
   D.S...K...V....(G.P)R.DGG.....SK.   X   lau   Star-nosed mole
   DRSG...S.G.....(S.P).DD.S...D.V..   X   afr   Elephant
   DSNG.R.S..V..I.(SLP)..D.N........   X   afr   Cape elephant shrew
   D--G...S.G.Q...(S.P)..D.S........   X   afr   Manatee
   DRS....S.G.....(S.L)H.D.N.......L   X   afr   Cape golden mole
   DSPGA..S.A.....(S.P).AD.N........   X   afr   Tenrec
   NSSG.R.S.RG....(S.P)..D.N........   X   afr   Aardvark
   D.SA...N.G.....(S.P)..D.SLRR..R..   X   xen   Armadillo
   --------TAV.AS.(..P)PKDDS..--.WKQ   X   mon   Platypus

NO 101
GN SPP1
ID OSTP_HUMAN
MP 106
DE Osteopontin
CL simians
SQ 50
   EDDDDHVDSQDSIDS(NDS)DDVDDTDDSHQSD   O   hum   Human
   ...............(...).............   O   hac   Chimp
   ...............(...).............   O   aga   Gorilla
   ...............(...).............   O   ape   Gibbon
   ...............(...).E...........   O   cat   Rhesus macaque
   ...............(...).E...........   O   cat   Crab-eating macaque
   ...............(...).E...........   O   cat   Baboon
   ...............(...).E...........   O   cat   Green monkey
   D.........---..(...).E..H........   O   sim   Marmoset
   ............V..(K..).E..H........   X   sim   Squirrel monkey
   .........----E.(D..)..T.NP....H..   X   pri   Bushbaby
   ...GN.....---..(D..)..T.HP....H..   X   eua   Treeshrew
   D..G...NN...T..(D..)-------EDDH..   X   gli   Squirrel
   D..G.....H..V..(D..)E.T.H--.DYH..   X   gli   Lesser Egyptian jerboa
   D..G..A.....V..(DE.)-------.DD.P.   X   gli   Prairie vole
   N..G..AN....V..(DE.)-----E..--HP.   X   gli   Chinese hamster
   D..G..AN....V..(.E.)..D.PD..--HP.   O   gli   Golden hamster
   D..G..AE.E..V..(DE.)-------------   X   gli   Mouse
   D..G..AE.E..VN.(DE.)-------------   X   gli   Rat
   ...G.LT.-----.P(..E)------..A.H.N   X   gli   Guinea pig
   D..G.Q--TA..A.T(D.A)-------...H..   X   gli   Chinchilla
   D.R.E-----..V.T(D.P)---------.H..   X   gli   Brush-tailed rat
   DE......N...NE.(D.P)-------...H..   X   gli   Rabbit
   DE.E..N..HSF...(D.A)-------...H..   X   gli   Pika
   D....T.....-V..(...).----...TDR..   O   lau   Alpaca
   D....T.....-V..(...).----...TDR..   O   lau   Bactrian camel
   .E..SQ--DT.AS..(S.T)----------H..   X   lau   Dolphin
   .E..SQDN-----..(..T)----------H..   O   lau   Killer whale
   D..NSQ-----EVN.(D..)..A--P...DH.N   X   lau   Tibetan antelope
   D.NSQD.------N.(...)..AET...PDH..   O   lau   Cow
   D..G..E.D......(D..).ET.P...PDN..   X   lau   Horse
   D..G...........(D..).ET.P...PD..E   X   lau   White rhinoceros
   ...E.D......V..(H.T)------------.   X   lau   Cat
   ...G.D......V..(..L)------------.   X   lau   Dog
   D..R.D......V..(D.V)------------.   X   lau   Ferret
   ...R.D......D..(..G)------------.   X   lau   Panda
   ...G.D.........(..V)------------.   X   lau   Pacific walrus
   ...G.D.........(..V)------------.   X   lau   Weddell seal
   ...E.......H...(D..)..A.H....DN..   X   lau   Black flying-fox
   ...E.......H...(D..)..A.H....DN..   X   lau   Megabat
   D..E...........(...)..D.H...PDN..   O   lau   David's myotis bat
   D..E.R.........(...)..D.R...PDN..   O   lau   Microbat
   D..E...........(...)..D.H...PDT..   O   lau   Big brown bat
   DR.G.AL..H---N.(DED)..D.EAE.TDS..   X   lau   Hedgehog
   ...G........V..(D.L)..D.H...PYH..   X   afr   Elephant
   ...I.....K..TE.(D..)E--.H...PYH..   X   afr   Cape elephant shrew
   ...GEQ.....P...(D.T)HED.H....NH.N   X   afr   Manatee
   ...S...NN...VE.(D..)E.H.....PDR..   X   afr   Cape golden mole
   A..S..SK....VE.(D..)..D.HA.EPDH..   X   afr   Tenrec
   ...G......---E.(D..)E.H.H...PYH..   X   afr   Aardvark

NO 102
GN SUSD2
ID SUSD2_HUMAN
MP 522
DE Sushi domain-containing protein 2
CL simians
SQ 52
   AVQEGNSDVVEVRLA(NRT)GGLEVLLNQEVLS   O   hum   Human
   ...............(...).............   O   hac   Chimp
   ...............(...)R............   O   aga   Gorilla
   ...............(...)R............   O   gra   Orangutan
   ...............(...)R........D...   O   ape   Gibbon
   ...............(.G.)R............   O   cat   Crab-eating macaque
   ...............(.G.)R............   O   cat   Baboon
   ...............(.G.)R............   O   cat   Green monkey
   ...............(...)R............   O   sim   Marmoset
   ...............(...).........G...   O   sim   Squirrel monkey
   ...............(D.A).V...........   X   pri   Bushbaby
   ....D..........(AGA).A.........Q.   X   eua   Treeshrew
   ...............(Q.S).V...........   X   gli   Squirrel
   .L......IL.....(DGS)RA..........N   X   gli   Lesser Egyptian jerboa
   ...............(GSP)QA.......KA..   X   gli   Prairie vole
   ....D..........(.GP)QA.Q.....KA..   X   gli   Chinese hamster
   ....D.........T(D.P)QA.Q.....KA..   X   gli   Golden hamster
   ....D....I.....(GGS)RV.......K...   X   gli   Mouse
   ....DK...I...V.(DGS)QV.......KL..   X   gli   Rat
   V....S.........(GGS).V......S....   X   gli   Naked mole-rat
   V...DS.....A...(H.P).V......S....   X   gli   Guinea pig
   V...D..........(SGS).V......S....   X   gli   Chinchilla
   VI..D..........(DGS).V......S....   X   gli   Brush-tailed rat
   ...............(DGS).V...........   X   gli   Rabbit
   ....HD.........(EG.).V.Q.........   X   gli   Pika
   ...............(S.D)RV.Q.........   X   lau   Alpaca
   ...............(R.D).V.Q.........   X   lau   Bactrian camel
   ..............R(DGA).A.Q.........   X   lau   Dolphin
   ..............R(DGA).A.Q.........   X   lau   Killer whale
   .I..A.........G(DGA).V.Q.---.....   X   lau   Tibetan antelope
   ....A.........G(DGA).V.Q.........   X   lau   Cow
   .I..A.........G(DGA).V.Q.........   X   lau   Domestic goat
   .....D.........(GGA).V.Q.........   X   lau   Horse
   ...............(DGV).V.Q.........   X   lau   White rhinoceros
   ...............(GGA).V.Q.........   X   lau   Cat
   ...............(GGA).V.Q.........   X   lau   Dog
   .........L.....(GGE).V.Q.........   X   lau   Panda
   ...............(GEG)RV.Q........R   X   lau   Pacific walrus
   ...............(GEG)RV.Q.........   X   lau   Weddell seal
   ...............(GE.).V.Q.........   X   lau   Black flying-fox
   ...............(GE.).V.Q.........   X   lau   Megabat
   V....T...L....T(DGA)KM.Q.....H...   X   afr   Elephant
   ..R..T..IL.....(.EN)RK.......R...   X   afr   Cape elephant shrew
   .....T...L.....(DGA)RM.Q.....R...   X   afr   Manatee
   .....T...L....S(DES)RT.Q.....R...   X   afr   Cape golden mole
   ....DSL..L.....(SGS)AT.Q.....R...   X   afr   Tenrec
   .....T.........(GEA)KTPQ...K.R...   X   afr   Aardvark
   .....A...L.....(GGA)..........S..   X   xen   Armadillo
   ..K..T...L.A..G(DPA)RP.......RL.N   X   mar   Opossum
   ..K.NV........G(GSG)KT........L.N   X   mar   Tasmanian devil
   V.K.NT...L....G(DNG)RAF.......M.N   X   mar   Wallaby
   .M...S...I...RL(EG.)A...........D   X   mon   Platypus

NO 103
GN TCN1
ID TCO1_HUMAN
MP 216
DE Transcobalamin-1
CL catarrhines
SQ 55
   SLINGQIKADEGSLK(NIS)IYTKSLVEKILSE   O   hum   Human
   ......V........(...).............   O   hac   Chimp
   ......V.....T..(...).............   O   aga   Gorilla
   ......V.....NS.(...)..I..........   O   gra   Orangutan
   ......V........(...).............   O   ape   Gibbon
   ......V.T..SN..(...)..I..........   O   cat   Rhesus macaque
   ......V.T..SN..(...)..I..........   O   cat   Crab-eating macaque
   ......V.T..SN..(...)..I..........   O   cat   Baboon
   ......V.T..SN..(...)..I..........   O   cat   Green monkey
   ......V...G.D.M(K..)N......D....K   X   sim   Marmoset
   ....A.V...G.D.M(K.N)N.K.........K   X   sim   Squirrel monkey
   R.M........KAVE(R..)NH.E...K.....   X   pri   Bushbaby
   N.VD.K..T.TED.R(S..)NHIEL..K.....   X   eua   Treeshrew
   T.T.E....-.KN.T(K.D)E-IRF..K.....   X   gli   Squirrel
   ..TK..T....EN..(S.E)NHIQ...K.....   X   gli   Lesser Egyptian jerboa
   -.EK..KE.N.N.IY(T.A)FSLV..EHRTVVN   X   gli   Prairie vole
   -.KK..K....NYTY(TVD)FFLM...HRAMVN   X   gli   Mouse
   P-KG..KR.N.NYAY(S.D)FFLM...HRVMVN   X   gli   Rat
   G.....T.T.KKD.R(S.D)DHIQ...K.....   X   gli   Naked mole-rat
   VVTC..TET.TED.S(SLY)NHIG...KM....   X   gli   Guinea pig
   G.A...T.TEKED.R(S.D)NHIQ...D...L.   X   gli   Chinchilla
   D..K..T.V.SED.R(S.D)NHIR...K.....   X   gli   Brush-tailed rat
   .....N...G.ED..(E.N)NHIA...K....Q   X   gli   Rabbit
   .IS..K...AIKDSD(T.Q)K.IE...H..Q..   X   lau   Pig
   .IMS.K.E...KD.N(K.N)H.RE...N.T...   X   lau   Alpaca
   .ITS.K.E...KD.N(K.N)H.RE...N.T...   X   lau   Bactrian camel
   RIEKEE.E..IKD.H(KTN)V.IT...N....A   X   lau   Dolphin
   TIEKEE.E..IKD.N(KTN)V.IT...N....A   X   lau   Killer whale
   ---DT.K.L.ANTKR(K..)G.IA..AN..QA.   X   lau   Tibetan antelope
   ---.T.KNM.MNAEG(K..)N.VA..AN..QA.   X   lau   Cow
   ---DT.R.MGASTQR(K..)G.IA..AN..QA.   X   lau   Sheep
   ---DT.K.MNANTKR(K..)G.IA..AN..QA.   X   lau   Domestic goat
   K----..ET.GEY.N(H.K)KHK....K.....   X   lau   Horse
   NVT.....I.SKD.E(C.N)NHI....K.....   X   lau   White rhinoceros
   -IT..KK.T.VEN.N(..N)NHI...I.....Q   X   lau   Cat
   DKT.S.NIMYRQD.E(I.G)GHIE..IR....Q   X   lau   Dog
   T--RR.KQI.KKDIE(..N)N..E..IN....Q   X   lau   Ferret
   KITRR.KQTQRKAM.(..V)NH.....NE...Q   X   lau   Panda
   .ITS..N.I.TKD.E(T.D)NHME..IA....Q   X   lau   Pacific walrus
   T--R..NQI.TKD.N(..D)NHI.L.IR....Q   X   lau   Weddell seal
   RI..EK.E...ED.N(R.N)NH.EL..K.....   X   lau   Black flying-fox
   RI..EK.E...ED.N(R.N)NH.EL..K.....   X   lau   Megabat
   .QN..E------D.Q(E.N)HHIE...K.....   X   lau   David's myotis bat
   .QN..E------D.Q(D..)H.IE...K.....   X   lau   Microbat
   .IM.R..QTNGED.Q(H.N)N..E...K.....   X   lau   Big brown bat
   .VV.K..QT----..(V.N)NNIQL..K...A.   X   lau   Shrew
   .VV...LE.GTEH.D(S..)KHI.....I....   X   lau   Star-nosed mole
   .IEKQK.E..TTISV(T.D)NHI...IK.....   X   afr   Elephant
   .IK.Q......NTSL(K..)NNIEL..K.....   X   afr   Cape elephant shrew
   .I..R.--E..KISM(S.N)NH.EL.IK.....   X   afr   Manatee
   .KV.LS-.I.TNIAT(I..)SHIET.I......   X   afr   Cape golden mole
   .I..Q...T.QSVTQ(S.D)SHIGI..K.....   X   afr   Tenrec
   ---.Q...T.Q.N.E(R.D)N..E...K...L.   X   xen   Armadillo
   -----------KPD.(E.R)NDIRM...R..K.   X   mar   Tasmanian devil
   ------------QPD(M.R)SNILW......K.   X   mar   Wallaby

NO 104
GN TFR2
ID TFR2_HUMAN
MP 754
DE Transferrin receptor protein 2
CL primates
SQ 55
   HTLGALLDHLRLLRS(NSS)GTPGATSSTGFQE   O   hum   Human
   ...............(...).............   O   hac   Chimp
   ...............(...)...R.........   O   aga   Gorilla
   ...............(...).............   O   gra   Orangutan
   ...............(.C.).............   O   ape   Gibbon
   ...............(...)........AV...   O   cat   Rhesus macaque
   ...............(...)........AV...   O   cat   Crab-eating macaque
   ...............(...)........AV...   O   cat   Baboon
   ...............(...)........AV...   O   cat   Green monkey
   ...............(...)R.......A....   O   sim   Marmoset
   ...............(...)R....P.AA....   O   sim   Squirrel monkey
   ..........Q...T(.GT)EA.R....A....   O   pri   Bushbaby
   ..............T(G..).S...S..I....   X   eua   Treeshrew
   ..........Q....(...).S.-----P....   O   gli   Squirrel
   ...D...E.......(GAA)S----AAPP....   X   gli   Lesser Egyptian jerboa
   ...........M...(DGF)K----A.......   X   gli   Prairie vole
   ...........M...(DG.)K-------AS...   X   gli   Chinese hamster
   ...D.......M...(DG.)K----A.......   X   gli   Golden hamster
   ......V....M..A(DG.).----A..S....   X   gli   Mouse
   ......VE...M...(DG.).----A..P....   X   gli   Rat
   ...DG.........P(DG.)RASK.S...S...   X   gli   Naked mole-rat
   ..............P(EA.)QS.--K..AS...   X   gli   Guinea pig
   ..............P(EA.)RA.--K..A....   X   gli   Chinchilla
   ..............P(EA.)RA.---K.AS...   X   gli   Brush-tailed rat
   ..............T(GN.)D------.D....   X   gli   Pika
   ...T.....V.....(GGP).AL.....-....   X   lau   Pig
   .........V.....(GG.).AL..V..S....   X   lau   Alpaca
   .........V.....(GG.).AL..V..S....   X   lau   Bactrian camel
   ......V..V.....(SGP).-----A.A....   X   lau   Dolphin
   ......V..V.....(SGP).-----A.A....   X   lau   Killer whale
   ...H.....A....A(GGA).---....K....   X   lau   Tibetan antelope
   ...H.....V....A(GGA).---....K....   X   lau   Sheep
   ...D.....V.....(HG.).D.R.A..S....   X   lau   Cat
   .........V.....(DG.).D...SP.S....   X   lau   Dog
   .........V.....(GG.).D....A.S....   X   lau   Ferret
   .........V.....(GG.).D...A..S....   X   lau   Panda
   .........V.....(GG.).DA..A..S....   X   lau   Pacific walrus
   .........V.....(GG.)ED...A..S....   X   lau   Weddell seal
   .........V.....(SG.).ALEVA.......   X   lau   Black flying-fox
   .........V.....(SG.).ALEVA.......   X   lau   Megabat
   .......N.V.....(S..)E....AT.A....   X   lau   David's myotis bat
   .......N.V.....(S..)E.L--AT.A....   X   lau   Microbat
   .......N.V.....(S..)E.L..AT.A....   X   lau   Big brown bat
   .........A.....(GGA)RD.KTA..AD...   X   lau   Hedgehog
   .........V....G(QG.).G.E.A..P....   X   lau   Shrew
   .......E.V....F(GN.)RD.E.S..I....   X   lau   Star-nosed mole
   ..............K(GD.)RA...S.P.....   X   afr   Elephant
   ...S..........K(GD.).A..VS.P.....   X   afr   Manatee
   ..........Q...K(GD.)RV...S.PI....   X   afr   Cape golden mole
   ...D..........K(GD.)TR..DS.P.....   X   afr   Tenrec
   ..............K(SD.).AL.TS.P.....   X   afr   Aardvark
   ..............P(RAP)---------....   X   xen   Armadillo
   ...R...E..Q...D(EGT).----DAPSH.E.   X   mar   Opossum
   ...R...E..Q...G(KKA).----AAPSH.E.   X   mar   Tasmanian devil
   ...K...E..Q....(KEA)E----NAPSH.E.   X   mar   Wallaby

NO 105
GN TG
ID THYG_HUMAN
MP 76
DE Thyroglobulin
CL humans
SQ 61
   VQCQNDGRSCWCVGA(NGS)EVLGSRQPGRPVA   O   hum   Human
   ...............(D..).............   X   hac   Chimp
   .......H.......(D..).............   X   aga   Gorilla
   ...............(D..).............   X   gra   Orangutan
   ...............(D..).............   X   ape   Gibbon
   ...............(D..).............   X   cat   Rhesus macaque
   ...............(D..)...........M.   X   cat   Crab-eating macaque
   ...............(D..).............   X   cat   Baboon
   ...............(D..)............V   X   cat   Green monkey
   ..............V(D.G).............   X   sim   Marmoset
   ...............(D.G).............   X   sim   Squirrel monkey
   ...............(..I)..P..........   X   pri   Bushbaby
   T....N.......D.(D..)..P..........   X   eua   Treeshrew
   ...R...........(D.R)..P...RQ...E.   X   gli   Squirrel
   .......S.....D.(...)..P....Q.....   O   gli   Lesser Egyptian jerboa
   .......Q.....DV(D.R)..P....L...T.   X   gli   Prairie vole
   .............DV(D.R)..P....L...T.   X   gli   Chinese hamster
   .......Q.....DS(D.R)..P....L...TV   X   gli   Mouse
   .......Q.....DS(D.T)..P....L...T.   X   gli   Rat
   I..L.........D.(D.R)..P........T.   X   gli   Naked mole-rat
   I..R.........D.(D.R)..P....SR..A.   X   gli   Guinea pig
   I..K.........D.(D.R)..P........T.   X   gli   Chinchilla
   T..K.........D.(D.R)..P........A.   X   gli   Brush-tailed rat
   L............D.(A.T)..P........A.   X   gli   Rabbit
   I..R...Q.....D.(E.T)..P..........   X   gli   Pika
   ...KK..G.....D.(D.R)..P..........   X   lau   Pig
   T..R.G.G.....D.(D.R)..P...R....T.   X   lau   Alpaca
   T..R.GSG.....D.(D.R)..P...R....A.   X   lau   Bactrian camel
   ...GK..G.....D.(D.R)..P........AS   X   lau   Dolphin
   ...GK..G.....D.(D.R)..P........AS   X   lau   Killer whale
   ...GK..A.....D.(D.R)..P..........   X   lau   Tibetan antelope
   ...GK..A.....D.(D.R)..P........A.   X   lau   Cow
   ...GK..A.....D.(D.R)..P........A.   X   lau   Sheep
   ...GK..A.....D.(D.R)..P........A.   X   lau   Domestic goat
   ...GDG..A......(D.L)..P........A.   X   lau   Horse
   ...GDG.........(D.G)..P...R....A.   X   lau   White rhinoceros
   ...ES..G.......(E.K)..P..........   X   lau   Cat
   ........T......(D.V)..P.....A..A.   X   lau   Dog
   ....S..G.....D.(D.R)..P.....A....   X   lau   Ferret
   ...R...G.....D.(..R)..P...R.A..A.   X   lau   Panda
   I......G.....D.(D.R)..P.....A....   X   lau   Pacific walrus
   I......G.....D.(D.R)..P.....A....   X   lau   Weddell seal
   ....Q..........(D.R)..P........A.   X   lau   Black flying-fox
   ....Q..........(D.R)..P..........   X   lau   Megabat
   ...RS..Y.....S.(D.R)..A........M.   X   lau   David's myotis bat
   ...RS..........(D.R)..A..........   X   lau   Microbat
   ....S..........(D.K)..A..........   X   lau   Big brown bat
   ...E.R.L.....D.(D.R)..A........S.   X   lau   Hedgehog
   ...EDS..F......(D.R)..P....R...S.   X   lau   Shrew
   ...EDG.........(D.L).LP....R...L.   X   lau   Star-nosed mole
   ...RK...A......(D.R)..P........AS   X   afr   Elephant
   .....G.GA......(D.G).IP...R.....S   X   afr   Cape elephant shrew
   ...RK...A....D.(D.R)..P.......L.S   X   afr   Manatee
   ...EKE.LA.....T(D.A)..P...L.....S   X   afr   Cape golden mole
   ...LKE..T......(D.G)..P........AS   X   afr   Tenrec
   ...RK..QA.....V(D.R)..P..K.S...MS   X   afr   Aardvark
   ...R....A......(D.G)..P........A.   X   xen   Armadillo
   ...S.N.L.....D.(E.T)..P..K.A.L.D.   X   mar   Opossum
   T..S.N.L.....D.(E.K)..P..KKAEL.A.   X   mar   Tasmanian devil
   ...S.S.L.....D.(E.K)..P..K.A.L.T.   X   mar   Wallaby
   T..SEKQ......D.(E.A)..P..KRA.V.DV   X   mon   Platypus

NO 106
GN TLR1
ID TLR1_HUMAN
MP 51
DE Toll-like receptor 1
CL primates
SQ 60
   LIHVPKDLSQKTTIL(NIS)QNYISELWTSDIL   O   hum   Human
   ...............(...).............   O   hac   Chimp
   ..R............(...).............   O   aga   Gorilla
   .T...........V.(...).............   O   gra   Orangutan
   ..Y............(...).............   O   ape   Gibbon
   ...............(...).............   O   cat   Rhesus macaque
   ...............(...).............   O   cat   Crab-eating macaque
   ...............(...).............   O   cat   Baboon
   ...............(...).............   O   cat   Green monkey
   ...I.........V.(...).......Q.....   O   sim   Marmoset
   ...I.........V.(...).......Q.....   O   sim   Squirrel monkey
   .T.......L.....(...)H.F...IQ..E.S   O   pri   Bushbaby
   .T.I.Q..PLE..T.(DM.)H......Q..VL.   X   eua   Treeshrew
   .T.I..E.ALE..A.(DV.).......Q.....   X   gli   Squirrel
   .T...R...L..AT.(...)H.N..Q.QI...S   O   gli   Lesser Egyptian jerboa
   .T......PLT..T.(DL.)..N....Q.....   X   gli   Prairie vole
   .T......PLT..T.(DL.)..N....Q...V.   X   gli   Chinese hamster
   .A......PLT..T.(DL.)..N....Q...V.   X   gli   Golden hamster
   .TR.....PLQ..T.(DL.)..N....Q.....   X   gli   Mouse
   .TR.....PLQ..T.(DV.)..N....Q.....   X   gli   Rat
   .T.......L...T.(...)......VQS....   O   gli   Naked mole-rat
   .T.......L...T.(D..)..N...IQS..V.   X   gli   Guinea pig
   .T.......L...T.(DL.)......IQS..M.   X   gli   Chinchilla
   .T.F.....P...V.(DM.)....A.IQS.N..   X   gli   Brush-tailed rat
   .L......PLQ..V.(D..)..S..A.Q.A.VQ   X   gli   Rabbit
   .F.F.....L...V.(D..)..SL...Q..NV.   X   gli   Pika
   .T.......LE....(DL.)..S....Q.....   X   lau   Pig
   ..C.....PL...V.(D..)..S....QA....   X   lau   Alpaca
   ..C....IPL...V.(D..)..S....QAP...   X   lau   Bactrian camel
   .T.......LN....(D..)............I   X   lau   Dolphin
   .T.......LN....(D..)............I   X   lau   Killer whale
   .TY......LE....(D..)Y......Q.P...   X   lau   Tibetan antelope
   .TY...N..LE....(D..)Y......QMP...   X   lau   Cow
   .TY..R...LE....(...)Y......QMP...   O   lau   Sheep
   .TY......LE....(D..)Y......Q.P...   X   lau   Domestic goat
   .T.......L.....(D..).......R....Q   X   lau   Horse
   .T.....V.P.....(D..).......R.....   X   lau   White rhinoceros
   .F....N..L.....(...).......R....I   O   lau   Cat
   .F.I.....L.....(D..).......Q.....   X   lau   Dog
   .F.......L.....(D..).......H.....   X   lau   Ferret
   .F.......L.....(D..).......Q.....   X   lau   Panda
   .F.......L.....(D..).......Q.....   X   lau   Pacific walrus
   .F.......L.....(D..).......Q.....   X   lau   Weddell seal
   .........L.....(...).......R.....   O   lau   Black flying-fox
   .........L.....(...).......R.....   O   lau   Megabat
   .T.......LE....(DL.)..D....QA....   X   lau   David's myotis bat
   .T.......L.....(DL.)..D....QA....   X   lau   Microbat
   .T......PL.....(D..)..D....QA.G..   X   lau   Big brown bat
   ...I....PPS....(.V.)H.......A....   O   lau   Hedgehog
   .N......PVE....(EL.)..S....H...F.   X   lau   Shrew
   .L....E..L.....(D..)..A...IR....R   X   lau   Star-nosed mole
   .T......TL.....(DA.).......QA....   X   afr   Elephant
   .TQI..N..L...V.(DL.)..S..S.Q...F.   X   afr   Cape elephant shrew
   .T.......L.....(DV.)..S....QA....   X   afr   Manatee
   .TY......L..MV.(DV.)..F..K.Q.....   X   afr   Cape golden mole
   YTD....I.L.....(DA.)..CF.Q.QA..FS   X   afr   Tenrec
   .T.I.....L...S.(DL.)....Y..Q....S   X   afr   Aardvark
   .TQI.....LR..V.(.V.).....K.Q....S   O   xen   Armadillo
   .S...HH..P...V.(DL.)L.N.T.IQIE.FK   X   mar   Opossum
   .S...SH..S.....(DL.)L.N.IK.QIE.FK   X   mar   Tasmanian devil

NO 107
GN TLR5
ID TLR5_HUMAN
MP 422
DE Toll-like receptor 5
CL simians
SQ 60
   DIFLSGNKLVTLPKI(NLT)ANLIHLSENRLEN   O   hum   Human
   ...............(...)....Y........   O   hac   Chimp
   ...............(...).............   O   aga   Gorilla
   ...............(...)..F..........   O   gra   Orangutan
   ...............(...)..F..........   O   ape   Gibbon
   ............SE.(...)..F..........   O   cat   Rhesus macaque
   ............SE.(...)..F..........   O   cat   Crab-eating macaque
   ............SE.(...)..F..........   O   cat   Baboon
   ............SE.(...)..F..........   O   cat   Green monkey
   N...G....A.....(...)..F..........   O   sim   Marmoset
   N...G....A.....(...)..F..........   O   sim   Squirrel monkey
   T...GS.........(..R).TF.S........   X   pri   Bushbaby
   ....G........H.(S..)..F.Q.......K   X   eua   Treeshrew
   TV..G........NV(.FK)..F......K...   X   gli   Squirrel
   T...GN...T...N.(S.A)..F.........S   X   gli   Lesser Egyptian jerboa
   TVL.G.....D..RV(RF.)..F.E....K..D   X   gli   Prairie vole
   AV..G....AD.TP.(RF.).SY.Q........   X   gli   Chinese hamster
   TVY.G....AD..H.(RFM)V.F.Q........   X   gli   Golden hamster
   MVL.G.....H..H.(HF.)..FLE........   X   gli   Mouse
   MVL.GS...TH..HV(RF.)..F.E....G...   X   gli   Rat
   TV..G....G...H.(..S)VDFMQ..A.....   O   gli   Naked mole-rat
   TV...........N.(D.R)V.F.Q.......K   X   gli   Chinchilla
   TVL.......S..DV(R.S)VHFLQ........   X   gli   Brush-tailed rat
   T.L.GN....S..N.(R..).KF.Q.......H   X   gli   Rabbit
   TLL.AN...AS.SDT(H..).KFLQ.T......   X   gli   Pika
   TL..G........N.(R..)..F..........   X   lau   Pig
   T...G........N.(T..)..F..........   X   lau   Alpaca
   TV..G........N.(T..)..F..........   X   lau   Bactrian camel
   N........A...N.(A..)..F.T........   X   lau   Dolphin
   N........A...N.(A..)..F.T........   X   lau   Killer whale
   N...G........N.(P..)....Q........   X   lau   Tibetan antelope
   N........M...N.(P..)..F.Q........   X   lau   Cow
   N...G........N.(P..)....Q........   X   lau   Sheep
   N...G........N.(P..)..F.Q........   X   lau   Domestic goat
   N...G........N.(Q..)..F..........   X   lau   White rhinoceros
   T...GS.......NV(R..)..F.........D   X   lau   Cat
   T...GN...E.VSHM(D..).SFLE..D....D   X   lau   Dog
   T...GS...AA..DM(E.K).SF.........D   X   lau   Ferret
   T....S......QNM(R.K)..FL........D   X   lau   Panda
   T...GS...AA..NM(R.K).KF.........D   X   lau   Pacific walrus
   T...GS...AA..S.(R.K).KF...P.....D   X   lau   Weddell seal
   N...G........T.(RF.)..F.........S   X   lau   Black flying-fox
   N...G....G..AT.(KFP)..F.P.......S   X   lau   Megabat
   TL..G....A...NT(RF.)..FVQ.......R   X   lau   David's myotis bat
   TL..G........NT(RF.).SFVQ.......R   X   lau   Microbat
   TLY.G........NT(KF.)..FVQ.......R   X   lau   Big brown bat
   MVL.G....TA..NV(Q..)..FLQ....K..H   X   lau   Hedgehog
   T...G........T.(KF.).SF.Q.......K   X   lau   Shrew
   T.L.G..N.....T.(TF.)..F...A.....T   X   lau   Star-nosed mole
   N...G........N.(K..)....Q........   X   afr   Elephant
   AL..G.......SN.(R.R)..F.Q......K.   X   afr   Cape elephant shrew
   N...G........N.(...)SDF.Q........   O   afr   Manatee
   T...G........N.(..V)T.F.Q........   X   afr   Cape golden mole
   T.Y.G.....K.SH.(S.V).DI...A......   X   afr   Tenrec
   N...G........N.(S.R)..F.Q........   X   afr   Aardvark
   T...G..R.K...DV(D..)..F.Q........   X   xen   Armadillo
   TA........HIEN.(.I.).GFLD.A....A.   O   mar   Opossum
   TV........SFERR(GI.).EFLD.A.....T   X   mar   Tasmanian devil
   MS........SFQN.(QII).EFLD.AQ....D   X   mar   Wallaby
   SA..A.....H.KGR(INS).EFL..AG....D   X   mon   Platypus

NO 108
GN TNC
ID TENA_HUMAN
MP 1034
DE Tenascin
CL euarchonts
SQ 61
   YSLPTGQWVGVQLPR(NTT)SYVLRGLEPGQEY   O   hum   Human
   ...............(...).............   O   hac   Chimp
   ...............(...).............   O   aga   Gorilla
   ...............(...).....D.......   O   gra   Orangutan
   ...............(...).............   O   ape   Gibbon
   ...............(...).............   O   cat   Rhesus macaque
   ...............(...).............   O   cat   Crab-eating macaque
   ...............(...).............   O   cat   Baboon
   ...............(...).............   O   cat   Green monkey
   .......R.E.....(...).......G.....   O   sim   Marmoset
   .......R.E.....(...).......G.....   O   sim   Squirrel monkey
   .......L.ET....(...).....D.Q.....   O   pri   Bushbaby
   .......P.E....K(...)..I..........   O   eua   Treeshrew
   ....A..SEE....K(D..).H..T........   X   gli   Lesser Egyptian jerboa
   .......SKE....K(DA.).H...D.......   X   gli   Prairie vole
   .......SIE....K(DA.).H..TD....K..   X   gli   Chinese hamster
   .......SIE....K(DA.).H..TD....K..   X   gli   Golden hamster
   .......SME....K(DA.).H..TD.......   X   gli   Mouse
   .......SIEI...K(DA.).H..TD.......   X   gli   Rat
   .......S.ED....(D..).............   X   gli   Naked mole-rat
   .......SMEE....(D..).H...........   X   gli   Guinea pig
   .......S.ED....(D..).............   X   gli   Chinchilla
   .......S.EE....(D..).H...........   X   gli   Brush-tailed rat
   ..S.M..PAE.L...(DS.)..I.T........   X   gli   Rabbit
   ..S....SAE.....(D..)..P....Q.....   X   gli   Pika
   .G..S..P.E.....(.A.)..I..........   O   lau   Pig
   ....S..P.E....K(DS.).......A.....   X   lau   Alpaca
   ....S..P.E....K(DS.).......A.....   X   lau   Bactrian camel
   ....S..P.E.....(G..)....K........   X   lau   Dolphin
   ....L..P.E.....(G..)....K........   X   lau   Killer whale
   ....S..P.E.....(D..)..........K..   X   lau   Tibetan antelope
   ....S..PKE...T.(D..).F...........   X   lau   Cow
   ....S..P.E.....(DA.).............   X   lau   Sheep
   ....S..P.E.....(D..).............   X   lau   Domestic goat
   ....S..P.E.R..K(A..).............   X   lau   Horse
   ....L..P.EMR..K(G..).H...........   X   lau   White rhinoceros
   ....S..P.E.K...(DA.).H...........   X   lau   Cat
   ....S..P.E.K...(D..)..D.......KG.   X   lau   Dog
   ....S..P.D.K...(D..)..........K..   X   lau   Ferret
   ....S..P.E.K...(D..)..........K..   X   lau   Panda
   ....S..P.E.K...(D..)..........K..   X   lau   Pacific walrus
   ....S..P.E.K...(D..)..........T..   X   lau   Weddell seal
   ....A..P.EM....(D..).T.....N.....   X   lau   Black flying-fox
   ....A..P.EM....(D..).T.....K.....   X   lau   Megabat
   ...AS..P.E.....(D..).............   X   lau   David's myotis bat
   ...AS..P.E.....(D..).............   X   lau   Microbat
   ...AS..PME.....(D..).............   X   lau   Big brown bat
   .....R.P.E.....(D..)..I..D.......   X   lau   Hedgehog
   ....A..SME.....(DI.)....KD....K..   X   lau   Shrew
   ...AA..P.E.....(D..)....HD.A.....   X   lau   Star-nosed mole
   ....M..QMQ.....(D..).H...........   X   afr   Elephant
   .......QME.....(D..).H..K........   X   afr   Cape elephant shrew
   ......KQME.K...(D..).H...........   X   afr   Manatee
   .......QTE.....(D..).H...D.......   X   afr   Cape golden mole
   ..V....SME.....(D..).H.....Q...P.   X   afr   Tenrec
   ..F....QMQ.....(G..).HI..........   X   afr   Aardvark
   ......RP.EK...K(D..).............   X   xen   Armadillo
   ..SS...QKE....A(D..)..I....N..T..   X   mar   Opossum
   ..SS...HKE....A(D..).....D.N..T..   X   mar   Tasmanian devil
   ..SSK..QKE....A(D..).....D.D..T..   X   mar   Wallaby
   .NSSS.KHHEME..G(G.S).......N..ID.   X   mon   Platypus

NO 109
GN TNC
ID TENA_HUMAN
MP 1275
DE Tenascin
CL primates
SQ 58
   GNLTVTEVSWDALRL(NWT)TPDGTYDQFTIQV   O   hum   Human
   ...............(...).............   O   hac   Chimp
   ............V..(...).............   O   aga   Gorilla
   ...............(...).............   O   gra   Orangutan
   ...............(...).............   O   ape   Gibbon
   ...............(...).............   O   cat   Rhesus macaque
   ...............(...).............   O   cat   Crab-eating macaque
   ...............(...).............   O   cat   Baboon
   ...............(...).............   O   cat   Green monkey
   ...............(...).........I...   O   sim   Marmoset
   ...............(...).........I...   O   sim   Squirrel monkey
   T.............M(...)...D.....I...   O   pri   Bushbaby
   ...........T.K.(D..)A............   X   eua   Treeshrew
   .......I...T...(D..)...DI....I...   X   gli   Squirrel
   ......KLN......(D..)...E.....I...   X   gli   Lesser Egyptian jerboa
   ......-L...V...(D..)...R..V..ITW.   X   gli   Prairie vole
   ....M.-L.S.S...(D..).T.RS....IT*.   X   gli   Chinese hamster
   .TF.MA-L.S.S...(D*G).T.RS.E..ITWI   X   gli   Golden hamster
   ..F...GLR..V.K.(D.N)I..R.....IT*I   X   gli   Mouse
   ..F...GLR*.V.T.(D.A)...RI....IT*I   X   gli   Rat
   .........S..FK.(D..)A........V.E.   X   gli   Naked mole-rat
   ......D.....FK.(D..)...EI....V.E.   X   gli   Guinea pig
   ....L.T....T.T.(D..)...D.....V.E.   X   gli   Brush-tailed rat
   ......K........(D..).........V...   X   gli   Rabbit
   ..........N....(D..)..E......V...   X   gli   Pika
   ...............(D..)S.E.I.E.YV.E.   X   lau   Pig
   .....S.........(S..)S...I.E..V.E.   X   lau   Alpaca
   .....S.........(S..)S...I.E..V.E.   X   lau   Bactrian camel
   ...............(D..)S...I.E..V.EI   X   lau   Dolphin
   ...............(D..)S...I.E..V.EI   X   lau   Killer whale
   ...............(H..)S...I.E..V.KI   X   lau   Tibetan antelope
   ......R........(H..)S...I.ER.V.KI   X   lau   Cow
   ........N......(Q..)S...I.E..V.KI   X   lau   Sheep
   ........N......(H..)S...I.E..V.KI   X   lau   Domestic goat
   ...............(D..)...EI.E..V.E.   X   lau   Horse
   .....A.........(D.I)....I.E..V.E.   X   lau   White rhinoceros
   ........R..G.S.(D..)A.E.F.E..V.A.   X   lau   Cat
   .....P..R..G.T.(D..)A...F.E..V.EI   X   lau   Dog
   ......K.G.HG.S.(D.M)A...I.E..I.EI   X   lau   Ferret
   ........H..G.T.(D..)A...I.E..V.EI   X   lau   Panda
   ......A.H..G.S.(D..)A...I.E..I.EI   X   lau   Pacific walrus
   ......A.H..G.S.(D..)A...I.E..I.EI   X   lau   Weddell seal
   ...............(...)....I.E..V.E.   O   lau   Black flying-fox
   ...............(H..)....I.E..V.E.   X   lau   David's myotis bat
   ...............(H..)....I.E..V.E.   X   lau   Microbat
   ...............(H..)S.A.I.E..V.E.   X   lau   Big brown bat
   ...........T...(D..)I...I.E..ILE.   X   lau   Hedgehog
   ..F....A..NT.T.(D..)S.E.I.E..V.E.   X   lau   Shrew
   ...............(D..)....I.E..V.EA   X   lau   Star-nosed mole
   ...........T...(D..)A.H...E..V...   X   afr   Elephant
   R........L.T...(D..)..EE..E..V...   X   afr   Cape elephant shrew
   ......K....T...(D..)......E..V.R.   X   afr   Manatee
   ..I.GNS....V..M(D.I)--H.NF..IV..A   X   afr   Cape golden mole
   ......N..R.T...(D..)S.....E..V...   X   afr   Aardvark
   ......K.G....K.(D..)....P.E..K...   X   xen   Armadillo
   .....AK....SFK.(...)AA..A.ET.V.K.   O   mar   Opossum
   .....AK....S.K.(...)AA..A.EI.V.K.   O   mar   Tasmanian devil
   .R...SDI..E..K.(..S)--.VPFEN.V...   O   mon   Platypus

NO 110
GN TSHR
ID TSHR_HUMAN
MP 113
DE Thyrotropin receptor
CL apes
SQ 60
   YNLSKVTHIEIRNTR(NLT)YIDPDALKELPLL   O   hum   Human
   ...............(...).............   O   hac   Chimp
   ...............(...).............   O   aga   Gorilla
   ...............(...).............   O   gra   Orangutan
   H..............(...).............   O   ape   Gibbon
   ...............(S..).............   X   cat   Rhesus macaque
   ...............(S..).............   X   cat   Crab-eating macaque
   ...............(S..).............   X   cat   Baboon
   ...............(S..).............   X   cat   Green monkey
   ...............(S..).............   X   sim   Marmoset
   .............A.(S..).............   X   sim   Squirrel monkey
   .....M.........(S..)...S.........   X   pri   Bushbaby
   .....M.........(S..)...TN..E.....   X   eua   Treeshrew
   .....M.........(S..)F......R.....   X   gli   Squirrel
   .....M..L......(S..).......E.....   X   gli   Lesser Egyptian jerboa
   .....M.........(S..).......T.....   X   gli   Prairie vole
   ----------.....(S..).......T.....   X   gli   Chinese hamster
   .....M.........(S..).......T.....   X   gli   Golden hamster
   .....M.........(S..).......T.....   X   gli   Mouse
   .....M.........(S..).......T.....   X   gli   Rat
   .....M.........(S..).............   X   gli   Naked mole-rat
   .....M.........(S..)..EH.........   X   gli   Guinea pig
   .....M.........(S..).......E.....   X   gli   Chinchilla
   .....M.........(S..).............   X   gli   Brush-tailed rat
   .....M.........(S..).............   X   gli   Rabbit
   .....M.........(S..)........K....   X   gli   Pika
   .....M.........(S..)..N.G...D....   X   lau   Pig
   .....M.........(S..)....G........   X   lau   Alpaca
   .....M.........(S..)....G........   X   lau   Bactrian camel
   .....M.........(S..)...RG........   X   lau   Dolphin
   .....M.........(S..)...HG........   X   lau   Killer whale
   H..............(S..)...SG........   X   lau   Tibetan antelope
   ...............(S..)...SG........   X   lau   Cow
   ...............(S..)...SG........   X   lau   Sheep
   ...............(S..)...SG........   X   lau   Domestic goat
   .....M.........(S..)..E.G........   X   lau   Horse
   .....M.........(S..)...AG........   X   lau   White rhinoceros
   .....M.........(S..)....G........   X   lau   Cat
   .....M.........(S..)S............   X   lau   Dog
   .....M.........(S..)S............   X   lau   Ferret
   .....M.........(S..)S............   X   lau   Panda
   .....M.........(S..)S............   X   lau   Pacific walrus
   .....M.........(S..)S............   X   lau   Weddell seal
   .....M.........(..A)....G........   X   lau   Black flying-fox
   .....M.........(..A)....G........   X   lau   Megabat
   .....M.........(S..)....G........   X   lau   David's myotis bat
   .....M.........(S..)....G........   X   lau   Microbat
   .....M.........(S..)....G........   X   lau   Big brown bat
   .....M.........(S..)....G........   X   lau   Hedgehog
   .....L.........(S..)...SG.F......   X   lau   Shrew
   .....I.........(S..)..EAG........   X   lau   Star-nosed mole
   ..M..M.........(S..)....G........   X   afr   Elephant
   ..M...A......M.(S..)..H.....K....   X   afr   Cape elephant shrew
   ..M..M.........(S..)....G........   X   afr   Manatee
   .....M.........(S..)....E........   X   afr   Tenrec
   .....M.........(S..)...H.........   X   xen   Armadillo
   .....M.........(S..).............   X   mar   Opossum
   .....M.........(...)........D....   O   mar   Tasmanian devil
   .....M.........(S..)........D....   X   mar   Wallaby
   .S.R.L.......VK(..I)Q.....F.N....   X   mon   Platypus

NO 111
GN UGT1A9
ID UD19_HUMAN
MP 344
DE UDP-glucuronosyltransferase 1-9
CL simians
SQ 58
   TVLWRYTGTRPSNLA(NNT)ILVKWLPQNDLLG   O   hum   Human
   ...............(...).............   O   hac   Chimp
   ...............(...).............   O   aga   Gorilla
   ...............(...).............   O   gra   Orangutan
   ...............(...).............   O   ape   Gibbon
   .........P.....(...).............   O   cat   Rhesus macaque
   .........P.....(...).............   O   cat   Crab-eating macaque
   .........P.....(...).............   O   cat   Baboon
   .........P.....(...).............   O   cat   Green monkey
   ...............(...).............   O   sim   Marmoset
   ...............(...).............   O   sim   Squirrel monkey
   ........S......(K..)............A   X   eua   Treeshrew
   .........P.....(K..)K............   X   gli   Squirrel
   ...............(K..).............   X   gli   Lesser Egyptian jerboa
   ........P......(K..).............   X   gli   Prairie vole
   ........PK.....(K..).............   X   gli   Chinese hamster
   ........PK.....(K..).............   X   gli   Golden hamster
   ...............(K..).............   X   gli   Mouse
   ...............(K..).............   X   gli   Rat
   .........P.....(K..).............   X   gli   Naked mole-rat
   .........P.....(K..).............   X   gli   Guinea pig
   .........P.....(K..).............   X   gli   Chinchilla
   .........P.....(K..).............   X   gli   Brush-tailed rat
   ........S......(K..).............   X   gli   Rabbit
   ........S......(K..).............   X   gli   Pika
   ........PA.P...(K..)K............   X   lau   Pig
   .........P.P...(K..)K............   X   lau   Alpaca
   .........P.P...(K..)K............   X   lau   Bactrian camel
   .........P.P...(K..)K............   X   lau   Dolphin
   .........P.P...(K..)K...........A   X   lau   Killer whale
   .........P.P...(K..)K............   X   lau   Tibetan antelope
   .........P.P...(K..)K............   X   lau   Cow
   .........P.P...(K..)K............   X   lau   Sheep
   .........P.P...(K..)K............   X   lau   Domestic goat
   .........P.P..S(K..).............   X   lau   Horse
   .........P.P..S(K..).............   X   lau   White rhinoceros
   .........P.P...(K..).............   X   lau   Cat
   .........P.P..S(K..).............   X   lau   Dog
   .........P.P...(K..)R............   X   lau   Panda
   .........P.P...(K..).............   X   lau   Pacific walrus
   .......S.P.P...(K..).............   X   lau   Weddell seal
   ........P......(K..).............   X   lau   Megabat
   ........A......(K..)...N.........   X   lau   David's myotis bat
   ...............(K..)...N.........   X   lau   Microbat
   ........P......(K..).............   X   lau   Hedgehog
   .........P.....(K..)K............   X   lau   Shrew
   ........P......(K..)............A   X   lau   Star-nosed mole
   ........SP.....(K..).............   X   afr   Elephant
   ...............(K..).............   X   afr   Cape elephant shrew
   ........S......(...).............   O   afr   Manatee
   ........S......(K..).............   X   afr   Cape golden mole
   ........P......(K..)K...........-   X   afr   Tenrec
   ........N......(K..)V............   X   afr   Aardvark
   ........SQ....G(K..)K............   X   xen   Armadillo
   ........KP.....(K..)K...........A   X   mar   Opossum
   ........KP.....(K..)K............   X   mar   Tasmanian devil
   .........P.P...(K..)K...........A   X   mar   Wallaby
   ........KP.A...(K..)K...........A   X   mon   Platypus

NO 112
GN VNN1
ID VNN1_HUMAN
MP 283
DE Pantetheinase
CL catarrhines
SQ 61
   HYPSKKMTGSGIYAP(NSS)RAFHYDMKTEEGK   O   hum   Human
   ...............(...).............   O   hac   Chimp
   ..............S(...).............   O   aga   Gorilla
   ...............(...)............R   O   gra   Orangutan
   ...............(...).............   O   ape   Gibbon
   .....N.........(S..).............   X   cat   Rhesus macaque
   .....N.........(S..).............   X   cat   Crab-eating macaque
   .....N.........(S..)............R   X   cat   Baboon
   ...............(...)............R   O   cat   Green monkey
   ...............(..P).........Q...   X   sim   Marmoset
   ....M..........(..P).........Q...   X   sim   Squirrel monkey
   .N..M..........(D.A).............   X   pri   Bushbaby
   ...............(G.P).V...........   X   eua   Treeshrew
   .N..NR.........(DFP).........P...   X   gli   Squirrel
   ...T.R.........(E.P)KS.....R.K...   X   gli   Lesser Egyptian jerboa
   .N.LRR.........(D.P)......R..D...   X   gli   Prairie vole
   .S.LRR.........(..P)K.....R..N...   X   gli   Chinese hamster
   ---------......(D.P)K.....R..N...   X   gli   Golden hamster
   .N..RR.........(D.P).V....R..Q...   X   gli   Mouse
   .I.LRR.........(D.P)......R..Q...   X   gli   Rat
   .N.LRR.........(DFP).T.......Q...   X   gli   Naked mole-rat
   .N.LLR.........(D.P).K.....E.Q...   X   gli   Guinea pig
   .N.LLR.........(DFP)KT.......K...   X   gli   Chinchilla
   .N.QQR.........(DFP).T.......K...   X   gli   Brush-tailed rat
   .C.............(D.P).............   X   gli   Rabbit
   .....N.........(D.P).............   X   gli   Pika
   .F.LR..........(D.P).........K...   X   lau   Pig
   ..RIT..........(D.P)K............   X   lau   Alpaca
   ...I...........(D.P)K............   X   lau   Bactrian camel
   ..R............(D.P).............   X   lau   Dolphin
   ..R............(D.P).............   X   lau   Killer whale
   ...L...........(D.P)Q............   X   lau   Tibetan antelope
   ...L...........(D.P).............   X   lau   Cow
   ...L...........(D.P).V...........   X   lau   Sheep
   ...L...........(D.P).V...........   X   lau   Domestic goat
   ....M..........(D.P).............   X   lau   Horse
   ....NR.........(D.P).............   X   lau   White rhinoceros
   .H.H.R.........(D.P).........K...   X   lau   Cat
   .H...R.........(D.P).........K...   X   lau   Dog
   .H..NR.........(D.P)QE.......KK..   X   lau   Ferret
   .H...R.........(D.P)KT.......K...   X   lau   Panda
   .D...R.........(D.P).........K...   X   lau   Pacific walrus
   .H...R.........(D.P).........K...   X   lau   Weddell seal
   .H.............(D.P).............   X   lau   Black flying-fox
   .H.............(D.P).............   X   lau   Megabat
   .H.............(D.P)..........K..   X   lau   David's myotis bat
   .H.............(D.P)..........K..   X   lau   Microbat
   .H.............(D.P)..........K..   X   lau   Big brown bat
   .R.............(D.P).........R...   X   lau   Hedgehog
   .N..RR.........(D.P)K........N...   X   lau   Shrew
   ...............(D.P).........K...   X   lau   Star-nosed mole
   .H.FMH.........(D.P).G...........   X   afr   Elephant
   .Q.L.R.........(...).........NK..   O   afr   Cape elephant shrew
   .N.QM...E.S....(D.P).............   X   afr   Manatee
   .H.MM..........(D.P)P........K...   X   afr   Cape golden mole
   .N.LM..........(D.P)K............   X   afr   Tenrec
   .H.L.E.........(D.P).............   X   afr   Aardvark
   ...IY..........(D.P).............   X   xen   Armadillo
   .SI.NQ.........(D.V)K.YYF.K.SDD..   X   mar   Opossum
   RNTVNQ.S.......(D.P)KV.YF.INSKN.T   X   mar   Tasmanian devil
   .NT.NR.........(D.P)KVY......DN..   X   mon   Platypus
```
