# Supplementary material for: The acquisition of novel N-glycosylation sites in conserved proteins during human evolution
Source: BMC Bioinformatics. 2015 Jan 28;16(1):29. doi: 10.1186/s12859-015-0468-5 (PMC4314935; doi:10.1186/s12859-015-0468-5)
Supplement: Additional file 4: — Molecular evolutionary analysis of APMAP. [file 12859_2015_468_MOESM4_ESM.zip › 12859_2015_468_MOESM4_ESM.html]

## Additional file 4. Molecular evolutionary analysis of APMAP.

**1. Multiple sequence alignment of selected primate orthologs (positive sites by Model A are in magenta background)**

```
human       MSEADGLRQRRPLRPQVVTDDDGQAPEAKDGSSFSGRVFRVTFLMLAVSLTVPLLGAMMLLESPIDPQPLSFKEPPLLLGVLHPNTKLRQAERLFENQLV  100
chimpanzee  ...................................................................................................I  100
gorilla     ........................................................................................Q..........I  100
orangutan   ...................................................................................................I  100
gibbon      ......................R..............................................F.............................I  100
rhesus      ...................................................................................................I  100


human       GPESIAHIGDVMFTGTADGRVVKLENGEIETIARFGSGPCKTRDDEPVCGRPLGIRAGPNGTLFVADAYKGLFEVNPWKREVKLLLSSETPIEGKNMSFV  200
chimpanzee  ............................................................R..................................K....  200
gorilla     ...............................................................................................K....  200
orangutan   ...............................................................................................K....  200
gibbon      ...............................................................................................K....  200
rhesus      ...............................................................................................K....  200


human       NDLTVTQDGRKIYFTDSSSKWQRRDYLLLVMEGTDDGRLLEYDTVTREVKVLLDQLRFPNGVQLSPAEDFVLVAETTMARIRRVYVSGLMKGGADLFVEN  300
chimpanzee  ....................................................................................................  300
gorilla     ....................................................................................................  300
orangutan   ....................................................................................................  300
gibbon      ....................................................................................................  300
rhesus      ....................................................................................................  300


human       MPGFPDNIRPSSSGGYWVGMSTIRPNPGFSMLDFLSERPWIKRMIFKLFSQETVMKFVPRYSLVLELSDSGAFRRSLHDPDGLVATYISEVHEHDGHLYL  400
chimpanzee  ....................................................................................................  400
gorilla     ....................................................................................................  400
orangutan   ....................................................................................................  400
gibbon      ....................................................................................................  400
rhesus      ............F........................................................................A.V............  400


human       GSFRSPFLCRLSLQAV  416
chimpanzee  ................  416
gorilla     ................  416
orangutan   ................  416
gibbon      ................  416
rhesus      ................  416
```

---

**2. Sequence data file "apmap.phy"**

```
6 1248

human
ATG AGC GAG GCG GAC GGG CTG CGA CAG CGC CGG CCC CTG CGG CCG CAG GTC GTC ACA GAC GAT GAT
GGC CAG GCC CCG GAG GCT AAG GAC GGC AGC TCC TTT AGC GGC AGA GTT TTC CGA GTG ACC TTC TTG
ATG CTG GCT GTT TCT CTC ACC GTT CCC CTG CTT GGA GCC ATG ATG CTG CTG GAA TCT CCT ATA GAT
CCA CAG CCT CTC AGC TTC AAA GAA CCC CCG CTC TTG CTT GGT GTT CTG CAT CCA AAT ACG AAG CTG
CGA CAG GCA GAA AGG CTG TTT GAA AAT CAA CTT GTT GGA CCG GAG TCC ATA GCA CAT ATT GGG GAT
GTG ATG TTT ACT GGG ACA GCA GAT GGC CGG GTC GTA AAA CTT GAA AAT GGT GAA ATA GAG ACC ATT
GCC CGG TTT GGT TCG GGC CCT TGC AAA ACC CGA GAT GAT GAG CCT GTG TGT GGG AGA CCC CTG GGT
ATC CGT GCA GGG CCC AAT GGG ACT CTC TTT GTG GCC GAT GCA TAC AAG GGA CTA TTT GAA GTA AAT
CCC TGG AAA CGT GAA GTG AAA CTG CTG CTG TCC TCC GAG ACA CCC ATT GAG GGG AAG AAC ATG TCC
TTT GTG AAT GAT CTT ACA GTC ACT CAG GAT GGG AGG AAG ATT TAT TTC ACC GAT TCT AGC AGC AAA
TGG CAA AGA CGA GAC TAC CTG CTT CTG GTG ATG GAG GGC ACA GAT GAC GGG CGC CTG CTG GAG TAT
GAT ACT GTG ACC AGG GAA GTA AAA GTT TTA TTG GAC CAG CTG CGG TTC CCG AAT GGA GTC CAG CTG
TCT CCT GCA GAA GAC TTT GTC CTG GTG GCA GAA ACA ACC ATG GCC AGG ATA CGA AGA GTC TAC GTT
TCT GGC CTG ATG AAG GGC GGG GCT GAT CTG TTT GTG GAG AAC ATG CCT GGA TTT CCA GAC AAC ATC
CGG CCC AGC AGC TCT GGG GGG TAC TGG GTG GGC ATG TCG ACC ATC CGC CCT AAC CCT GGG TTT TCC
ATG CTG GAT TTC TTA TCT GAG AGA CCC TGG ATT AAA AGG ATG ATT TTT AAG CTC TTT AGT CAA GAG
ACG GTG ATG AAG TTT GTG CCG CGG TAC AGC CTC GTC CTA GAA CTC AGC GAC AGC GGT GCC TTC CGG
AGA AGC CTG CAT GAT CCC GAT GGG CTG GTG GCC ACC TAC ATC AGC GAG GTG CAC GAA CAC GAT GGG
CAC CTG TAC CTG GGC TCT TTC AGG TCC CCC TTC CTC TGC AGA CTC AGC CTC CAG GCT GTT
chimpanzee
... ... ... ... ... ... ... ... ... ... ... ..T ... ... ... ... ... ... ... ... ... ...
... ... ... ... ... ... ... ... ... ... ... ... ... ... ... ... ... ... ... ... ... ...
... ... ... ... ... ... ... ... ... ... ... ... ... ... ... ... ... ... ... ... ... ...
... ... ... ... ... ... ... ... ... ... ... ... ... ... ... ... ... ... ... ... ... ...
... ... ... ... ... ... ... ... ... ... ... A.. ... ... ... ... ... ... ... ... ... ...
... ... ... ... ... ... ... ... ... ... ... ... ... ... ... ... ... ... ... ... ... ...
... ... ... ... ... ... ... ... ... ... ... ... ... ... ... ... ... ... ... ... ... ...
... ... ... ... ... ... A.. ... ... ... ... ..T ... ... ... ... ... ... ... ... ... ...
... ... ... ... ... ... ... ... ... ... ... ... ... ... ... ... ... ... ... ..A ... ...
... ... ... ... ... ... ... ... ... ... ... ... ... ... ... ... ... ... ... ... ... ...
... ... ... ... ... ... ... ... ... ... ... ... ... ... ... ... ... ... ... ... ... ...
... ... ... ... ... ... ... ... ... ... ... ... ... ... ... ... ... ... ... ... ... ...
... ... ... ... ... ... ... ... ... ... ... ... ... ... ... ... ... ... ... ... ... ...
... ... ... ... ... ... ... ... ... ... ... ... ... ... ... ... ... ... ... ... ... ...
... ... ... ... ... ... ... ... ... ... ... ... ... ... ... ... ... ... ... ... ... ...
... ... ... ... ... ... ... ... ... ... ... ... ... ... ... ... ... ... ... ... ... ...
... ... ... ... ... ... ... ... ... ... ... ... ... ... ... ... ... ... ... ... ... ...
... ... ... ... ... ..T ... ... ... ... ... ... ... ... ... ... ... ... ... ... ... ...
... ... ... ... ... ... ... ... ... ... ... ... ... ... ... ... ... ... ... ...
gorilla
... ... ... ... ... ... ... ... ... ... ... ... ... ... ... ... ... ... ... ... ... ...
... ... ... ... ... ... ... ... ... ... ... ... ... ... ... ... ... ... ... ... ... ...
... ... ... ... ... ... ... ... ... ... ... ... ... ... ... ... ... ... ... ... ... ...
... ... ... ... ... ... ... ... ... ... ..G ... ... ... ... ... ... ... ... ... ... T..
.A. ... ... ... ... ... ... ... ... ... ... A.. ... ... ... ... ... ... ... ... ... ...
... ... ... ... ... ... ... ... ... ... ... ... ... ... ... ... ... ... ... ... ... ...
... ... ... ... ..A ... ... ... ... ... ... ... ... ... ... ... ... ... ... ... ... ...
... ... ... ... ... ... ... ... ... ... ... ... ... ... ... ... ... ... ... ... ... ...
... ... ... ... ... ... ... ... ... ... ... ... ... ... ... ... ... ... ... ..A ... ...
... ... ... ... ... ... ... ... ... ... ... ... ... ... ... ... ..T ... ... ... ... ...
... ... ... ... ... ... ... ... ... ... ... ... ... ... ... ... ... ... ... ... ... ...
... ... ... ... ... ... ... ... ... ... ... ... ... ... ... ... ... ... ... ... ... ...
... ... ... ... ... ... ... ... ... ... ... ... ... ... ... ... ... ... ... ... ... ...
... ... ... ... ... ... ... ... ... ... ... ... ... ... ... ... ... ... ... ... ... ...
... ... ... ... ... ... ... ... ... ... ... ... ... ... ... ... ... ... ... ... ... ...
... ... ... ... ... ... ... ... ... ... ... ... ... ... ... ... ... ... ... ... ... ...
... ... ... ... ... ... ... ... ... ... ... ... ... ... ... ... ... ... ... ... ... ...
... ... ... ... ... ... ... ... ... ... ... ... ... ... ... ... ... ... ... ..T ... ...
... ... ... ... ... ... ... ... ... ... ... ... ... ... ... ... ... ... ... ...
orangutan
... ... ... ... ... ... ... ... ... ... ... ... ... ... ... ... ... ... ... ... ... ...
... ... ... ... ... ... ... ... ... ... ... ... ... ... ... ... ... ... ... ... ... ...
... ... ... ... ... ... ... ... ... ... ... ... ... ... ... ... ... ... ... ... ... ...
... ... ... ... ... ... ... ... ... ... ... ... ... ... ... ... ... ... ... ... ... T..
... ... ... ... ... ... ... ... ... ... ... A.. ... ... ... ... ... ... ... ... ... ...
... ... ... ..C ... ... ... ... ... ... ... ... ... ... ... ... ... ... ... ... ... ...
... ... ... ... ... ... ... ... ... ... ... ... ... ... ... ... ... ... ... ... ... ...
... ... ... ... ... ... ... ... ... ... ... ..T ... ... ... ... ... ... ... ... ... ...
... ... ... ... ... ... ... ... ... ... ... ... ... ... ... ... ... ... ... ..A ... ...
... ... ... ... ... ... ... ... ... ... ... ... ... ... ... ... ..T ... ... ... ... ..G
... ... ... ... ... ... ... ... ... ... ... ... ... ..T ... ... ... ... ... ... ... ..C
... ... ... ... ... ... ... ... ... ... ... ... ... ... ... ... ... ... ... ... ... ...
... ... ..G ... ... ... ... ... ... ... ... ... ... ... ... ... ... ... ... ... ... ...
... ... ... ... ... ... ... ... ... ... ... ... ... ... ... ... ... ... ... ... ... ...
... ... ... ... ... ... ... ... ... ... ... ... ..A ... ... ... ... ... ... ... ... ...
... ... ... ... ... ... ... ... ... ... ... ... ... ... ... ... ... ... ... ... ... ...
... ... ... ... ... ... ... ... ... ... ... ... ... ... ... ..T ... ... ... ... ... ...
... ... ... ... ... ... ..C ... ... ..A ... ... ... ... ..T ... ... ... ..G ... ... ...
... ... ... ... ... ... ... ... ... ... ... ... ... ... ... ... ... ... ... ...
gibbon
... ... ... ... ... ... ... ... ... ..G ... ... ... ... ... ... ..T ..T ... ... ... ...
C.. ... ... ... ... ... ... ... ... ... ... ... ... ... ... ... ... ... ... ... ... ...
... ... ... ... ... ... ... ... ... ... ... ... ... ... ... ..A ... ... ... ... ... ...
... ... ... T.. ... ... ... ... ... ... ... ... ... ..C ... ... ... ... ... ... ... T..
... ... ... ... ... ... ... ... ... ... ... A.. ... ... ... ... ... ... ... ... ... ..C
... ... ... ... ... ... ... ... ... ... ... ... ... ... ... ... ... ... ... ... ... ...
... ... ... ... ... ... ... ... ... ... ... ... ... ... ... ... ... ... ... ... ... ...
... ... ... ... ... ... ... ... ... ... ... ..T ... ... ... ... ... ... ... ... ... ..C
... ... ... ... ... ... ... ... ... ... ... ... ... ... ... ... ... ... ... ..A ... ...
... ... ... ... ... ..G ... ... ... ... ... ... ... ... ... ... ... ... ... ... ... ...
... ... ... ... ... ... ... ... ... ... ... ... ... ... ... ..T ... ... ... ... ... ..C
... ... ... ... ... ... ... ... ... ... ... ... ... ... ... ... ... ... ... ... ... ...
... ... ... ... ... ... ... T.. ... ... ... ... ..G ... ... ... ... ... ... ... ... ...
... ... ..A ... ... ... ... ... ... ... ... ... ... ... ... ... ... ... ... ... ... ...
... ... ... ... ... ... ... ... ... ..T ... ... ... ... ... ... ... ... ... ... ... ...
... ... ... ... ... ... ... ... ... ... ... ... ... ... ... ... ... ... ... ... ... ...
... ... ... ... ... ... ..A ... ... ... ... ... ... ... ... ... ... ... ... ... ... ...
... ... ... ... ... ... ..C ... ... ... ... ... ... ... ... ... ... ... ..G ... ... ...
... ... ... ... ... ... ... ... ..A ... ... ... ... ... ... ... ... ... ... ...
rhesus
... ... ... ... ... ... ... ... ... ... ... ..T ... ... ... ... ... ... ..G ... ... ...
... ... ... ... ... ... ... ... ... ... ... ... ... ... ... ... ... ... ... ... ... ...
... ... ... ... ... ... ..T ... ... ... ... ... ... ... ... ... ... ... ... ... ... ...
... ... ... ... ... ... ... ... ... ... ... ... ... ..C ... ... ... ... ... ..A ... T..
... ... ... ... ... ... ... ... ... ..G ... A.. ... ... ... ... ... ... ... ..C ... ...
... ... ... ... ... ... ... ... ... ... ..T ... ... ... ... ... ... ... ... ... ... ...
... ... ... ... ... ..T ..G ... ... ... ... ... ... ... ... ... ... ... ... ... ... ...
... ... ... ... ... ... ... ... ... ... ... ..T ... ... ... ... ... ... ... ... ... ...
... ... ... ... ... ... ... ... ... ... ... ... ... ... ... ... ... ... ... ..A ... ...
... ... ... ... ... ..G ... ... ... ... ... ... ... ... ... ... ..T ... ... ... ... ...
... ... ... ... ... ... ... ... ... ... ... ... ... ... ... ... ... ... ... ... ... ..C
... ... ... ... ... ... ... ... ... ... ... ... ... ... ... ... ... ... ... ... ... ...
... ... ... ... ... ... ... ... ... ... ... ..G ... ... ... ... ... ... ... ... ..T ...
... ... ... ... ... ... ... ... ... ... ... ... ... ... ... ... ... ... ... ... ... ...
... ... ... ... .T. ... ... ... ... ..C ... ... ..A ... ... ... ... ... ... ... ... ...
... ... ... ... ... ... ... ... ... ... ..C ... ... ... ... ... ... ... ... ... ..G ...
... ... ... ... ... ... ... ... ... ... ... ... ... ... ... ... ... ... ... ... ... ...
... ... ... ... ... ... ..C ... ... ... ... G.. ... G.. ..T ... ... ... ..G ... ..C ...
... ... ... ... ... ... ... ... ... ... ... ... ... ... ... ... ... ... ... ...
```

---

**3. Tree file "apmap.tree"**

```
((((human, chimpanzee), gorilla), orangutan), gibbon, rhesus);
```

**4. Tree file "apmap-human.tree"**

```
((((human #1, chimpanzee), gorilla), orangutan), gibbon, rhesus);
```

---

**5. Control file for "M0"**

```
      seqfile = apmap.phy
     treefile = apmap.tree
      outfile = apmap-M0-one-ratio.mlc

        noisy = 9  * 0,1,2,3,9: how much rubbish on the screen
      verbose = 0  * 0: concise; 1: detailed, 2: too much
      runmode = 0  * 0: user tree;  1: semi-automatic;  2: automatic
                   * 3: StepwiseAddition; (4,5):PerturbationNNI; -2: pairwise

      seqtype = 1  * 1:codons; 2:AAs; 3:codons-->AAs
    CodonFreq = 2  * 0:1/61 each, 1:F1X4, 2:F3X4, 3:codon table
        clock = 0  * 0:no clock, 1:clock; 2:local clock; 3:CombinedAnalysis
        model = 0
                   * models for codons:
                       * 0:one, 1:b, 2:2 or more dN/dS ratios for branches

      NSsites = 0  * 0:one w; 1:neutral; 2:selection; 3:discrete; 4:freqs;
                   * 5:gamma; 6:2gamma; 7:beta; 8:beta&w 9:betaγ
                   * 10:betaγ+1; 11:beta&normal>1; 12:0&2normal>1;
                   * 13:3normal>0
        icode = 0  * 0:universal code; 1:mammalian mt; 2-10:see below

    fix_kappa = 0  * 1: kappa fixed, 0: kappa to be estimated
        kappa = 2  * initial or fixed kappa
    fix_omega = 0  * 1: omega or omega_1 fixed, 0: estimate 
        omega = 1  * initial or fixed omega, for codons or codon-based AAs

    fix_alpha = 1  * 0: estimate gamma shape parameter; 1: fix it at alpha
        alpha = .0 * initial or fixed alpha, 0:infinity (constant rate)
       Malpha = 0  * different alphas for genes
        ncatG = 4  * # of categories in dG of NSsites models

        getSE = 0  * 0: don't want them, 1: want S.E.s of estimates
 RateAncestor = 0  * (0,1,2): rates (alpha>0) or ancestral states (1 or 2)
       method = 0  * 0: simultaneous; 1: one branch at a time
```

---

**6. Control file for "Free ratio"**

```
      seqfile = apmap.phy
     treefile = apmap.tree
      outfile = apmap-free-ratio.mlc

        noisy = 9  * 0,1,2,3,9: how much rubbish on the screen
      verbose = 0  * 0: concise; 1: detailed, 2: too much
      runmode = 0  * 0: user tree;  1: semi-automatic;  2: automatic
                   * 3: StepwiseAddition; (4,5):PerturbationNNI; -2: pairwise

      seqtype = 1  * 1:codons; 2:AAs; 3:codons-->AAs
    CodonFreq = 2  * 0:1/61 each, 1:F1X4, 2:F3X4, 3:codon table
        clock = 0  * 0:no clock, 1:clock; 2:local clock; 3:CombinedAnalysis
        model = 1
                   * models for codons:
                       * 0:one, 1:b, 2:2 or more dN/dS ratios for branches

      NSsites = 0  * 0:one w; 1:neutral; 2:selection; 3:discrete; 4:freqs;
                   * 5:gamma; 6:2gamma; 7:beta; 8:beta&w 9:betaγ
                   * 10:betaγ+1; 11:beta&normal>1; 12:0&2normal>1;
                   * 13:3normal>0
        icode = 0  * 0:universal code; 1:mammalian mt; 2-10:see below

    fix_kappa = 0  * 1: kappa fixed, 0: kappa to be estimated
        kappa = 2  * initial or fixed kappa
    fix_omega = 0  * 1: omega or omega_1 fixed, 0: estimate 
        omega = 1  * initial or fixed omega, for codons or codon-based AAs

    fix_alpha = 1  * 0: estimate gamma shape parameter; 1: fix it at alpha
        alpha = .0 * initial or fixed alpha, 0:infinity (constant rate)
       Malpha = 0  * different alphas for genes
        ncatG = 4  * # of categories in dG of NSsites models

        getSE = 0  * 0: don't want them, 1: want S.E.s of estimates
 RateAncestor = 0  * (0,1,2): rates (alpha>0) or ancestral states (1 or 2)
       method = 0  * 0: simultaneous; 1: one branch at a time
```

---

**7. Control file for "Two ratio"**

```
      seqfile = apmap.phy
     treefile = apmap-human.tree
      outfile = apmap-two-ratio.mlc

        noisy = 9  * 0,1,2,3,9: how much rubbish on the screen
      verbose = 0  * 0: concise; 1: detailed, 2: too much
      runmode = 0  * 0: user tree;  1: semi-automatic;  2: automatic
                   * 3: StepwiseAddition; (4,5):PerturbationNNI; -2: pairwise

      seqtype = 1  * 1:codons; 2:AAs; 3:codons-->AAs
    CodonFreq = 2  * 0:1/61 each, 1:F1X4, 2:F3X4, 3:codon table
        clock = 0  * 0:no clock, 1:clock; 2:local clock; 3:CombinedAnalysis
        model = 2
                   * models for codons:
                       * 0:one, 1:b, 2:2 or more dN/dS ratios for branches

      NSsites = 0  * 0:one w; 1:neutral; 2:selection; 3:discrete; 4:freqs;
                   * 5:gamma; 6:2gamma; 7:beta; 8:beta&w 9:betaγ
                   * 10:betaγ+1; 11:beta&normal>1; 12:0&2normal>1;
                   * 13:3normal>0
        icode = 0  * 0:universal code; 1:mammalian mt; 2-10:see below

    fix_kappa = 0  * 1: kappa fixed, 0: kappa to be estimated
        kappa = 2  * initial or fixed kappa
    fix_omega = 0  * 1: omega or omega_1 fixed, 0: estimate 
        omega = 1  * initial or fixed omega, for codons or codon-based AAs

    fix_alpha = 1  * 0: estimate gamma shape parameter; 1: fix it at alpha
        alpha = .0 * initial or fixed alpha, 0:infinity (constant rate)
       Malpha = 0  * different alphas for genes
        ncatG = 4  * # of categories in dG of NSsites models

        getSE = 0  * 0: don't want them, 1: want S.E.s of estimates
 RateAncestor = 0  * (0,1,2): rates (alpha>0) or ancestral states (1 or 2)
       method = 0  * 0: simultaneous; 1: one branch at a time
```

---

**8. Control file for "Model A"**

```
      seqfile = apmap.phy
     treefile = apmap-human.tree
      outfile = apmap-model-A.mlc

        noisy = 9  * 0,1,2,3,9: how much rubbish on the screen
      verbose = 0  * 0: concise; 1: detailed, 2: too much
      runmode = 0  * 0: user tree;  1: semi-automatic;  2: automatic
                   * 3: StepwiseAddition; (4,5):PerturbationNNI; -2: pairwise

      seqtype = 1  * 1:codons; 2:AAs; 3:codons-->AAs
    CodonFreq = 2  * 0:1/61 each, 1:F1X4, 2:F3X4, 3:codon table
        clock = 0  * 0:no clock, 1:clock; 2:local clock; 3:CombinedAnalysis
        model = 2
                   * models for codons:
                       * 0:one, 1:b, 2:2 or more dN/dS ratios for branches

      NSsites = 2  * 0:one w; 1:neutral; 2:selection; 3:discrete; 4:freqs;
                   * 5:gamma; 6:2gamma; 7:beta; 8:beta&w 9:betaγ
                   * 10:betaγ+1; 11:beta&normal>1; 12:0&2normal>1;
                   * 13:3normal>0
        icode = 0  * 0:universal code; 1:mammalian mt; 2-10:see below

    fix_kappa = 0  * 1: kappa fixed, 0: kappa to be estimated
        kappa = 2  * initial or fixed kappa
    fix_omega = 0  * 1: omega or omega_1 fixed, 0: estimate 
        omega = 1  * initial or fixed omega, for codons or codon-based AAs

    fix_alpha = 1  * 0: estimate gamma shape parameter; 1: fix it at alpha
        alpha = .0 * initial or fixed alpha, 0:infinity (constant rate)
       Malpha = 0  * different alphas for genes
        ncatG = 4  * # of categories in dG of NSsites models

        getSE = 0  * 0: don't want them, 1: want S.E.s of estimates
 RateAncestor = 0  * (0,1,2): rates (alpha>0) or ancestral states (1 or 2)
       method = 0  * 0: simultaneous; 1: one branch at a time
```

---

**9. Control file for "Null model A"**

```
      seqfile = apmap.phy
     treefile = apmap-human.tree
      outfile = apmap-null-model-A.mlc

        noisy = 9  * 0,1,2,3,9: how much rubbish on the screen
      verbose = 0  * 0: concise; 1: detailed, 2: too much
      runmode = 0  * 0: user tree;  1: semi-automatic;  2: automatic
                   * 3: StepwiseAddition; (4,5):PerturbationNNI; -2: pairwise

      seqtype = 1  * 1:codons; 2:AAs; 3:codons-->AAs
    CodonFreq = 2  * 0:1/61 each, 1:F1X4, 2:F3X4, 3:codon table
        clock = 0  * 0:no clock, 1:clock; 2:local clock; 3:CombinedAnalysis
        model = 2
                   * models for codons:
                       * 0:one, 1:b, 2:2 or more dN/dS ratios for branches

      NSsites = 2  * 0:one w; 1:neutral; 2:selection; 3:discrete; 4:freqs;
                   * 5:gamma; 6:2gamma; 7:beta; 8:beta&w 9:betaγ
                   * 10:betaγ+1; 11:beta&normal>1; 12:0&2normal>1;
                   * 13:3normal>0
        icode = 0  * 0:universal code; 1:mammalian mt; 2-10:see below

    fix_kappa = 0  * 1: kappa fixed, 0: kappa to be estimated
        kappa = 2  * initial or fixed kappa
    fix_omega = 1  * 1: omega or omega_1 fixed, 0: estimate 
        omega = 1  * initial or fixed omega, for codons or codon-based AAs

    fix_alpha = 1  * 0: estimate gamma shape parameter; 1: fix it at alpha
        alpha = .0 * initial or fixed alpha, 0:infinity (constant rate)
       Malpha = 0  * different alphas for genes
        ncatG = 4  * # of categories in dG of NSsites models

        getSE = 0  * 0: don't want them, 1: want S.E.s of estimates
 RateAncestor = 0  * (0,1,2): rates (alpha>0) or ancestral states (1 or 2)
       method = 0  * 0: simultaneous; 1: one branch at a time
```

---

**10. Main result file for "M0"**

```
CODONML (in paml version 4.8a, July 2014)  apmap.phy
Model: One dN/dS ratio for branches, 
Codon frequency model: F3x4
ns =   6  ls = 416

Codon usage in sequences
--------------------------------------------------------------------------------------------------------------------------------------
Phe TTT  14  14  14  14  14  15 | Ser TCT   8   8   8   8   8   7 | Tyr TAT   2   2   2   1   1   2 | Cys TGT   1   1   1   1   1   1
    TTC   9   9   9   9  10   9 |     TCC   7   7   7   7   6   7 |     TAC   7   7   7   8   8   7 |     TGC   2   2   2   2   2   2
Leu TTA   2   2   2   2   2   2 |     TCA   0   0   1   1   1   1 | *** TAA   0   0   0   0   0   0 | *** TGA   0   0   0   0   0   0
    TTG   3   3   4   4   5   4 |     TCG   2   2   1   1   2   1 |     TAG   0   0   0   0   0   0 | Trp TGG   4   4   4   4   4   4
--------------------------------------------------------------------------------------------------------------------------------------
Leu CTT   6   6   6   6   6   6 | Pro CCT   8  10   8   8   8   8 | His CAT   3   3   4   3   3   3 | Arg CGT   2   2   2   2   2   2
    CTC  10  10   9  10   9  10 |     CCC  11   9  11  11  11  10 |     CAC   3   3   2   3   3   3 |     CGC   3   3   3   3   3   3
    CTA   2   2   2   2   4   2 |     CCA   3   3   3   3   4   3 | Gln CAA   3   3   4   3   3   1 |     CGA   6   6   5   6   6   6
    CTG  27  27  27  26  23  26 |     CCG   6   6   6   6   5   7 |     CAG   9   9   9   9   9  11 |     CGG   8   8   8   8   9   8
--------------------------------------------------------------------------------------------------------------------------------------
Ile ATT   6   7   7   7   7   5 | Thr ACT   4   4   5   5   4   6 | Asn AAT   7   7   7   7   6   7 | Ser AGT   1   1   1   3   1   2
    ATC   4   4   4   4   4   5 |     ACC   9   9   8   9   8   6 |     AAC   4   3   3   3   4   3 |     AGC  14  14  14  12  14  13
    ATA   4   4   4   4   4   4 |     ACA   6   6   6   5   5   4 | Lys AAA   8   9   9   8   9   9 | Arg AGA   7   7   7   7   7   7
Met ATG  14  14  14  14  14  14 |     ACG   2   2   2   2   4   4 |     AAG   8   8   8   9   8   8 |     AGG   6   7   6   6   6   6
--------------------------------------------------------------------------------------------------------------------------------------
Val GTT   8   7   7   7  10   8 | Ala GCT   4   5   4   5   5   5 | Asp GAT  18  18  18  17  17  16 | Gly GGT   5   5   5   5   4   5
    GTC   8   8   8   8   6   9 |     GCC   7   6   7   6   6   7 |     GAC   9   9   9  10  10  11 |     GGC  10  10  10  10  10  10
    GTA   3   3   3   4   3   3 |     GCA   7   7   7   6   7   7 | Glu GAA  13  13  13  12  12  12 |     GGA   5   5   5   5   5   5
    GTG  15  15  15  14  14  14 |     GCG   1   1   1   2   1   1 |     GAG  13  13  13  14  14  14 |     GGG  15  14  15  15  15  15
--------------------------------------------------------------------------------------------------------------------------------------

Codon position x base (3x4) table for each sequence.

#1: human          
position  1:    T:0.14663    C:0.26442    A:0.25000    G:0.33894
position  2:    T:0.32452    C:0.20433    A:0.25721    G:0.21394
position  3:    T:0.23317    C:0.28125    A:0.16587    G:0.31971
Average         T:0.23478    C:0.25000    A:0.22436    G:0.29087

#2: chimpanzee     
position  1:    T:0.14663    C:0.26442    A:0.25481    G:0.33413
position  2:    T:0.32452    C:0.20433    A:0.25721    G:0.21394
position  3:    T:0.24038    C:0.27163    A:0.16827    G:0.31971
Average         T:0.23718    C:0.24679    A:0.22676    G:0.28926

#3: gorilla        
position  1:    T:0.14904    C:0.26202    A:0.25240    G:0.33654
position  2:    T:0.32452    C:0.20433    A:0.25962    G:0.21154
position  3:    T:0.23798    C:0.27163    A:0.17067    G:0.31971
Average         T:0.23718    C:0.24599    A:0.22756    G:0.28926

#4: orangutan      
position  1:    T:0.14904    C:0.26202    A:0.25240    G:0.33654
position  2:    T:0.32452    C:0.20433    A:0.25721    G:0.21394
position  3:    T:0.23798    C:0.27644    A:0.16346    G:0.32212
Average         T:0.23718    C:0.24760    A:0.22436    G:0.29087

#5: gibbon         
position  1:    T:0.15385    C:0.25962    A:0.25240    G:0.33413
position  2:    T:0.32452    C:0.20433    A:0.25721    G:0.21394
position  3:    T:0.23317    C:0.27404    A:0.17308    G:0.31971
Average         T:0.23718    C:0.24599    A:0.22756    G:0.28926

#6: rhesus         
position  1:    T:0.14904    C:0.26202    A:0.24760    G:0.34135
position  2:    T:0.32692    C:0.20192    A:0.25721    G:0.21394
position  3:    T:0.23558    C:0.27644    A:0.15865    G:0.32933
Average         T:0.23718    C:0.24679    A:0.22115    G:0.29487

Sums of codon usage counts
------------------------------------------------------------------------------
Phe F TTT      85 | Ser S TCT      47 | Tyr Y TAT      10 | Cys C TGT       6
      TTC      55 |       TCC      41 |       TAC      44 |       TGC      12
Leu L TTA      12 |       TCA       4 | *** * TAA       0 | *** * TGA       0
      TTG      23 |       TCG       9 |       TAG       0 | Trp W TGG      24
------------------------------------------------------------------------------
Leu L CTT      36 | Pro P CCT      50 | His H CAT      19 | Arg R CGT      12
      CTC      58 |       CCC      63 |       CAC      17 |       CGC      18
      CTA      14 |       CCA      19 | Gln Q CAA      17 |       CGA      35
      CTG     156 |       CCG      36 |       CAG      56 |       CGG      49
------------------------------------------------------------------------------
Ile I ATT      39 | Thr T ACT      28 | Asn N AAT      41 | Ser S AGT       9
      ATC      25 |       ACC      49 |       AAC      20 |       AGC      81
      ATA      24 |       ACA      32 | Lys K AAA      52 | Arg R AGA      42
Met M ATG      84 |       ACG      16 |       AAG      49 |       AGG      37
------------------------------------------------------------------------------
Val V GTT      47 | Ala A GCT      28 | Asp D GAT     104 | Gly G GGT      29
      GTC      47 |       GCC      39 |       GAC      58 |       GGC      60
      GTA      19 |       GCA      41 | Glu E GAA      75 |       GGA      30
      GTG      87 |       GCG       7 |       GAG      81 |       GGG      89
------------------------------------------------------------------------------


Codon position x base (3x4) table, overall

position  1:    T:0.14904    C:0.26242    A:0.25160    G:0.33694
position  2:    T:0.32492    C:0.20393    A:0.25761    G:0.21354
position  3:    T:0.23638    C:0.27524    A:0.16667    G:0.32171
Average         T:0.23678    C:0.24720    A:0.22529    G:0.29073


Nei & Gojobori 1986. dN/dS (dN, dS)
(Note: This matrix is not used in later ML. analysis.
Use runmode = -2 for ML pairwise comparison.)

human               
chimpanzee           0.3317 (0.0032 0.0097)
gorilla              0.1978 (0.0032 0.0162) 0.0817 (0.0021 0.0262)
orangutan            0.0462 (0.0021 0.0463) 0.0215 (0.0011 0.0498) 0.0214 (0.0011 0.0498)
gibbon               0.0638 (0.0043 0.0672) 0.0454 (0.0032 0.0707) 0.0411 (0.0032 0.0780) 0.0262 (0.0021 0.0814)
rhesus               0.0632 (0.0054 0.0849) 0.0526 (0.0043 0.0813) 0.0483 (0.0043 0.0887) 0.0413 (0.0032 0.0778) 0.0518 (0.0054 0.1033)


TREE #  1:  ((((1, 2), 3), 4), 5, 6);   MP score: 62
lnL(ntime:  9  np: 11):  -2042.096834      +0.000000
   7..8     8..9     9..10   10..1    10..2     9..3     8..4     7..5     7..6  
 0.005750 0.011081 0.004799 0.006063 0.008783 0.011089 0.017802 0.042870 0.048848 7.895736 0.073847

Note: Branch length is defined as number of nucleotide substitutions per codon (not per neucleotide site).

tree length =   0.15709

((((1: 0.006063, 2: 0.008783): 0.004799, 3: 0.011089): 0.011081, 4: 0.017802): 0.005750, 5: 0.042870, 6: 0.048848);

((((human: 0.006063, chimpanzee: 0.008783): 0.004799, gorilla: 0.011089): 0.011081, orangutan: 0.017802): 0.005750, gibbon: 0.042870, rhesus: 0.048848);

Detailed output identifying parameters

kappa (ts/tv) =  7.89574

omega (dN/dS) =  0.07385

dN & dS for each branch

 branch          t       N       S   dN/dS      dN      dS  N*dN  S*dS

   7..8      0.006   857.8   390.2  0.0738  0.0004  0.0053   0.3   2.1
   8..9      0.011   857.8   390.2  0.0738  0.0008  0.0102   0.6   4.0
   9..10     0.005   857.8   390.2  0.0738  0.0003  0.0044   0.3   1.7
  10..1      0.006   857.8   390.2  0.0738  0.0004  0.0056   0.4   2.2
  10..2      0.009   857.8   390.2  0.0738  0.0006  0.0081   0.5   3.1
   9..3      0.011   857.8   390.2  0.0738  0.0008  0.0102   0.6   4.0
   8..4      0.018   857.8   390.2  0.0738  0.0012  0.0163   1.0   6.4
   7..5      0.043   857.8   390.2  0.0738  0.0029  0.0393   2.5  15.3
   7..6      0.049   857.8   390.2  0.0738  0.0033  0.0448   2.8  17.5

tree length for dN:       0.0106
tree length for dS:       0.1441


Time used:  0:04
```

---

**11. Main result file for "Free ratio"**

```
CODONML (in paml version 4.8a, July 2014)  apmap.phy
Model: free dN/dS Ratios for branches for branches, 
Codon frequency model: F3x4
ns =   6  ls = 416

Codon usage in sequences
--------------------------------------------------------------------------------------------------------------------------------------
Phe TTT  14  14  14  14  14  15 | Ser TCT   8   8   8   8   8   7 | Tyr TAT   2   2   2   1   1   2 | Cys TGT   1   1   1   1   1   1
    TTC   9   9   9   9  10   9 |     TCC   7   7   7   7   6   7 |     TAC   7   7   7   8   8   7 |     TGC   2   2   2   2   2   2
Leu TTA   2   2   2   2   2   2 |     TCA   0   0   1   1   1   1 | *** TAA   0   0   0   0   0   0 | *** TGA   0   0   0   0   0   0
    TTG   3   3   4   4   5   4 |     TCG   2   2   1   1   2   1 |     TAG   0   0   0   0   0   0 | Trp TGG   4   4   4   4   4   4
--------------------------------------------------------------------------------------------------------------------------------------
Leu CTT   6   6   6   6   6   6 | Pro CCT   8  10   8   8   8   8 | His CAT   3   3   4   3   3   3 | Arg CGT   2   2   2   2   2   2
    CTC  10  10   9  10   9  10 |     CCC  11   9  11  11  11  10 |     CAC   3   3   2   3   3   3 |     CGC   3   3   3   3   3   3
    CTA   2   2   2   2   4   2 |     CCA   3   3   3   3   4   3 | Gln CAA   3   3   4   3   3   1 |     CGA   6   6   5   6   6   6
    CTG  27  27  27  26  23  26 |     CCG   6   6   6   6   5   7 |     CAG   9   9   9   9   9  11 |     CGG   8   8   8   8   9   8
--------------------------------------------------------------------------------------------------------------------------------------
Ile ATT   6   7   7   7   7   5 | Thr ACT   4   4   5   5   4   6 | Asn AAT   7   7   7   7   6   7 | Ser AGT   1   1   1   3   1   2
    ATC   4   4   4   4   4   5 |     ACC   9   9   8   9   8   6 |     AAC   4   3   3   3   4   3 |     AGC  14  14  14  12  14  13
    ATA   4   4   4   4   4   4 |     ACA   6   6   6   5   5   4 | Lys AAA   8   9   9   8   9   9 | Arg AGA   7   7   7   7   7   7
Met ATG  14  14  14  14  14  14 |     ACG   2   2   2   2   4   4 |     AAG   8   8   8   9   8   8 |     AGG   6   7   6   6   6   6
--------------------------------------------------------------------------------------------------------------------------------------
Val GTT   8   7   7   7  10   8 | Ala GCT   4   5   4   5   5   5 | Asp GAT  18  18  18  17  17  16 | Gly GGT   5   5   5   5   4   5
    GTC   8   8   8   8   6   9 |     GCC   7   6   7   6   6   7 |     GAC   9   9   9  10  10  11 |     GGC  10  10  10  10  10  10
    GTA   3   3   3   4   3   3 |     GCA   7   7   7   6   7   7 | Glu GAA  13  13  13  12  12  12 |     GGA   5   5   5   5   5   5
    GTG  15  15  15  14  14  14 |     GCG   1   1   1   2   1   1 |     GAG  13  13  13  14  14  14 |     GGG  15  14  15  15  15  15
--------------------------------------------------------------------------------------------------------------------------------------

Codon position x base (3x4) table for each sequence.

#1: human          
position  1:    T:0.14663    C:0.26442    A:0.25000    G:0.33894
position  2:    T:0.32452    C:0.20433    A:0.25721    G:0.21394
position  3:    T:0.23317    C:0.28125    A:0.16587    G:0.31971
Average         T:0.23478    C:0.25000    A:0.22436    G:0.29087

#2: chimpanzee     
position  1:    T:0.14663    C:0.26442    A:0.25481    G:0.33413
position  2:    T:0.32452    C:0.20433    A:0.25721    G:0.21394
position  3:    T:0.24038    C:0.27163    A:0.16827    G:0.31971
Average         T:0.23718    C:0.24679    A:0.22676    G:0.28926

#3: gorilla        
position  1:    T:0.14904    C:0.26202    A:0.25240    G:0.33654
position  2:    T:0.32452    C:0.20433    A:0.25962    G:0.21154
position  3:    T:0.23798    C:0.27163    A:0.17067    G:0.31971
Average         T:0.23718    C:0.24599    A:0.22756    G:0.28926

#4: orangutan      
position  1:    T:0.14904    C:0.26202    A:0.25240    G:0.33654
position  2:    T:0.32452    C:0.20433    A:0.25721    G:0.21394
position  3:    T:0.23798    C:0.27644    A:0.16346    G:0.32212
Average         T:0.23718    C:0.24760    A:0.22436    G:0.29087

#5: gibbon         
position  1:    T:0.15385    C:0.25962    A:0.25240    G:0.33413
position  2:    T:0.32452    C:0.20433    A:0.25721    G:0.21394
position  3:    T:0.23317    C:0.27404    A:0.17308    G:0.31971
Average         T:0.23718    C:0.24599    A:0.22756    G:0.28926

#6: rhesus         
position  1:    T:0.14904    C:0.26202    A:0.24760    G:0.34135
position  2:    T:0.32692    C:0.20192    A:0.25721    G:0.21394
position  3:    T:0.23558    C:0.27644    A:0.15865    G:0.32933
Average         T:0.23718    C:0.24679    A:0.22115    G:0.29487

Sums of codon usage counts
------------------------------------------------------------------------------
Phe F TTT      85 | Ser S TCT      47 | Tyr Y TAT      10 | Cys C TGT       6
      TTC      55 |       TCC      41 |       TAC      44 |       TGC      12
Leu L TTA      12 |       TCA       4 | *** * TAA       0 | *** * TGA       0
      TTG      23 |       TCG       9 |       TAG       0 | Trp W TGG      24
------------------------------------------------------------------------------
Leu L CTT      36 | Pro P CCT      50 | His H CAT      19 | Arg R CGT      12
      CTC      58 |       CCC      63 |       CAC      17 |       CGC      18
      CTA      14 |       CCA      19 | Gln Q CAA      17 |       CGA      35
      CTG     156 |       CCG      36 |       CAG      56 |       CGG      49
------------------------------------------------------------------------------
Ile I ATT      39 | Thr T ACT      28 | Asn N AAT      41 | Ser S AGT       9
      ATC      25 |       ACC      49 |       AAC      20 |       AGC      81
      ATA      24 |       ACA      32 | Lys K AAA      52 | Arg R AGA      42
Met M ATG      84 |       ACG      16 |       AAG      49 |       AGG      37
------------------------------------------------------------------------------
Val V GTT      47 | Ala A GCT      28 | Asp D GAT     104 | Gly G GGT      29
      GTC      47 |       GCC      39 |       GAC      58 |       GGC      60
      GTA      19 |       GCA      41 | Glu E GAA      75 |       GGA      30
      GTG      87 |       GCG       7 |       GAG      81 |       GGG      89
------------------------------------------------------------------------------


Codon position x base (3x4) table, overall

position  1:    T:0.14904    C:0.26242    A:0.25160    G:0.33694
position  2:    T:0.32492    C:0.20393    A:0.25761    G:0.21354
position  3:    T:0.23638    C:0.27524    A:0.16667    G:0.32171
Average         T:0.23678    C:0.24720    A:0.22529    G:0.29073


Nei & Gojobori 1986. dN/dS (dN, dS)
(Note: This matrix is not used in later ML. analysis.
Use runmode = -2 for ML pairwise comparison.)

human               
chimpanzee           0.3317 (0.0032 0.0097)
gorilla              0.1978 (0.0032 0.0162) 0.0817 (0.0021 0.0262)
orangutan            0.0462 (0.0021 0.0463) 0.0215 (0.0011 0.0498) 0.0214 (0.0011 0.0498)
gibbon               0.0638 (0.0043 0.0672) 0.0454 (0.0032 0.0707) 0.0411 (0.0032 0.0780) 0.0262 (0.0021 0.0814)
rhesus               0.0632 (0.0054 0.0849) 0.0526 (0.0043 0.0813) 0.0483 (0.0043 0.0887) 0.0413 (0.0032 0.0778) 0.0518 (0.0054 0.1033)


TREE #  1:  ((((1, 2), 3), 4), 5, 6);   MP score: 62
lnL(ntime:  9  np: 19):  -2035.757177      +0.000000
   7..8     8..9     9..10   10..1    10..2     9..3     8..4     7..5     7..6  
 0.006117 0.012484 0.004870 0.004876 0.009823 0.009933 0.017685 0.042984 0.048288 7.936822 0.000100 0.000100 0.000100 999.000000 0.150258 0.148282 0.000100 0.058333 0.081088

Note: Branch length is defined as number of nucleotide substitutions per codon (not per neucleotide site).

tree length =   0.15706

((((1: 0.004876, 2: 0.009823): 0.004870, 3: 0.009933): 0.012484, 4: 0.017685): 0.006117, 5: 0.042984, 6: 0.048288);

((((human: 0.004876, chimpanzee: 0.009823): 0.004870, gorilla: 0.009933): 0.012484, orangutan: 0.017685): 0.006117, gibbon: 0.042984, rhesus: 0.048288);

Detailed output identifying parameters

kappa (ts/tv) =  7.93682

w (dN/dS) for branches:  0.00010 0.00010 0.00010 999.00000 0.15026 0.14828 0.00010 0.05833 0.08109

dN & dS for each branch

 branch          t       N       S   dN/dS      dN      dS  N*dN  S*dS

   7..8      0.006   857.6   390.4  0.0001  0.0000  0.0065   0.0   2.5
   8..9      0.012   857.6   390.4  0.0001  0.0000  0.0133   0.0   5.2
   9..10     0.005   857.6   390.4  0.0001  0.0000  0.0052   0.0   2.0
  10..1      0.005   857.6   390.4 999.0000  0.0024  0.0000   2.0   0.0
  10..2      0.010   857.6   390.4  0.1503  0.0012  0.0079   1.0   3.1
   9..3      0.010   857.6   390.4  0.1483  0.0012  0.0080   1.0   3.1
   8..4      0.018   857.6   390.4  0.0001  0.0000  0.0188   0.0   7.4
   7..5      0.043   857.6   390.4  0.0583  0.0024  0.0406   2.0  15.8
   7..6      0.048   857.6   390.4  0.0811  0.0035  0.0437   3.0  17.1

tree length for dN:       0.0106
tree length for dS:       0.1440

dS tree:
((((human: 0.000002, chimpanzee: 0.007870): 0.005188, gorilla: 0.007984): 0.013300, orangutan: 0.018842): 0.006517, gibbon: 0.040602, rhesus: 0.043677);
dN tree:
((((human: 0.002364, chimpanzee: 0.001183): 0.000001, gorilla: 0.001184): 0.000001, orangutan: 0.000002): 0.000001, gibbon: 0.002368, rhesus: 0.003542);

w ratios as labels for TreeView:
((((human #999.0000 , chimpanzee #0.1503 ) #0.0001 , gorilla #0.1483 ) #0.0001 , orangutan #0.0001 ) #0.0001 , gibbon #0.0583 , rhesus #0.0811 );


Time used:  0:32
```

---

**12. Main result file for "Two ratio"**

```
CODONML (in paml version 4.8a, July 2014)  apmap.phy
Model: several dN/dS ratios for branches for branches, 
Codon frequency model: F3x4
ns =   6  ls = 416

Codon usage in sequences
--------------------------------------------------------------------------------------------------------------------------------------
Phe TTT  14  14  14  14  14  15 | Ser TCT   8   8   8   8   8   7 | Tyr TAT   2   2   2   1   1   2 | Cys TGT   1   1   1   1   1   1
    TTC   9   9   9   9  10   9 |     TCC   7   7   7   7   6   7 |     TAC   7   7   7   8   8   7 |     TGC   2   2   2   2   2   2
Leu TTA   2   2   2   2   2   2 |     TCA   0   0   1   1   1   1 | *** TAA   0   0   0   0   0   0 | *** TGA   0   0   0   0   0   0
    TTG   3   3   4   4   5   4 |     TCG   2   2   1   1   2   1 |     TAG   0   0   0   0   0   0 | Trp TGG   4   4   4   4   4   4
--------------------------------------------------------------------------------------------------------------------------------------
Leu CTT   6   6   6   6   6   6 | Pro CCT   8  10   8   8   8   8 | His CAT   3   3   4   3   3   3 | Arg CGT   2   2   2   2   2   2
    CTC  10  10   9  10   9  10 |     CCC  11   9  11  11  11  10 |     CAC   3   3   2   3   3   3 |     CGC   3   3   3   3   3   3
    CTA   2   2   2   2   4   2 |     CCA   3   3   3   3   4   3 | Gln CAA   3   3   4   3   3   1 |     CGA   6   6   5   6   6   6
    CTG  27  27  27  26  23  26 |     CCG   6   6   6   6   5   7 |     CAG   9   9   9   9   9  11 |     CGG   8   8   8   8   9   8
--------------------------------------------------------------------------------------------------------------------------------------
Ile ATT   6   7   7   7   7   5 | Thr ACT   4   4   5   5   4   6 | Asn AAT   7   7   7   7   6   7 | Ser AGT   1   1   1   3   1   2
    ATC   4   4   4   4   4   5 |     ACC   9   9   8   9   8   6 |     AAC   4   3   3   3   4   3 |     AGC  14  14  14  12  14  13
    ATA   4   4   4   4   4   4 |     ACA   6   6   6   5   5   4 | Lys AAA   8   9   9   8   9   9 | Arg AGA   7   7   7   7   7   7
Met ATG  14  14  14  14  14  14 |     ACG   2   2   2   2   4   4 |     AAG   8   8   8   9   8   8 |     AGG   6   7   6   6   6   6
--------------------------------------------------------------------------------------------------------------------------------------
Val GTT   8   7   7   7  10   8 | Ala GCT   4   5   4   5   5   5 | Asp GAT  18  18  18  17  17  16 | Gly GGT   5   5   5   5   4   5
    GTC   8   8   8   8   6   9 |     GCC   7   6   7   6   6   7 |     GAC   9   9   9  10  10  11 |     GGC  10  10  10  10  10  10
    GTA   3   3   3   4   3   3 |     GCA   7   7   7   6   7   7 | Glu GAA  13  13  13  12  12  12 |     GGA   5   5   5   5   5   5
    GTG  15  15  15  14  14  14 |     GCG   1   1   1   2   1   1 |     GAG  13  13  13  14  14  14 |     GGG  15  14  15  15  15  15
--------------------------------------------------------------------------------------------------------------------------------------

Codon position x base (3x4) table for each sequence.

#1: human          
position  1:    T:0.14663    C:0.26442    A:0.25000    G:0.33894
position  2:    T:0.32452    C:0.20433    A:0.25721    G:0.21394
position  3:    T:0.23317    C:0.28125    A:0.16587    G:0.31971
Average         T:0.23478    C:0.25000    A:0.22436    G:0.29087

#2: chimpanzee     
position  1:    T:0.14663    C:0.26442    A:0.25481    G:0.33413
position  2:    T:0.32452    C:0.20433    A:0.25721    G:0.21394
position  3:    T:0.24038    C:0.27163    A:0.16827    G:0.31971
Average         T:0.23718    C:0.24679    A:0.22676    G:0.28926

#3: gorilla        
position  1:    T:0.14904    C:0.26202    A:0.25240    G:0.33654
position  2:    T:0.32452    C:0.20433    A:0.25962    G:0.21154
position  3:    T:0.23798    C:0.27163    A:0.17067    G:0.31971
Average         T:0.23718    C:0.24599    A:0.22756    G:0.28926

#4: orangutan      
position  1:    T:0.14904    C:0.26202    A:0.25240    G:0.33654
position  2:    T:0.32452    C:0.20433    A:0.25721    G:0.21394
position  3:    T:0.23798    C:0.27644    A:0.16346    G:0.32212
Average         T:0.23718    C:0.24760    A:0.22436    G:0.29087

#5: gibbon         
position  1:    T:0.15385    C:0.25962    A:0.25240    G:0.33413
position  2:    T:0.32452    C:0.20433    A:0.25721    G:0.21394
position  3:    T:0.23317    C:0.27404    A:0.17308    G:0.31971
Average         T:0.23718    C:0.24599    A:0.22756    G:0.28926

#6: rhesus         
position  1:    T:0.14904    C:0.26202    A:0.24760    G:0.34135
position  2:    T:0.32692    C:0.20192    A:0.25721    G:0.21394
position  3:    T:0.23558    C:0.27644    A:0.15865    G:0.32933
Average         T:0.23718    C:0.24679    A:0.22115    G:0.29487

Sums of codon usage counts
------------------------------------------------------------------------------
Phe F TTT      85 | Ser S TCT      47 | Tyr Y TAT      10 | Cys C TGT       6
      TTC      55 |       TCC      41 |       TAC      44 |       TGC      12
Leu L TTA      12 |       TCA       4 | *** * TAA       0 | *** * TGA       0
      TTG      23 |       TCG       9 |       TAG       0 | Trp W TGG      24
------------------------------------------------------------------------------
Leu L CTT      36 | Pro P CCT      50 | His H CAT      19 | Arg R CGT      12
      CTC      58 |       CCC      63 |       CAC      17 |       CGC      18
      CTA      14 |       CCA      19 | Gln Q CAA      17 |       CGA      35
      CTG     156 |       CCG      36 |       CAG      56 |       CGG      49
------------------------------------------------------------------------------
Ile I ATT      39 | Thr T ACT      28 | Asn N AAT      41 | Ser S AGT       9
      ATC      25 |       ACC      49 |       AAC      20 |       AGC      81
      ATA      24 |       ACA      32 | Lys K AAA      52 | Arg R AGA      42
Met M ATG      84 |       ACG      16 |       AAG      49 |       AGG      37
------------------------------------------------------------------------------
Val V GTT      47 | Ala A GCT      28 | Asp D GAT     104 | Gly G GGT      29
      GTC      47 |       GCC      39 |       GAC      58 |       GGC      60
      GTA      19 |       GCA      41 | Glu E GAA      75 |       GGA      30
      GTG      87 |       GCG       7 |       GAG      81 |       GGG      89
------------------------------------------------------------------------------


Codon position x base (3x4) table, overall

position  1:    T:0.14904    C:0.26242    A:0.25160    G:0.33694
position  2:    T:0.32492    C:0.20393    A:0.25761    G:0.21354
position  3:    T:0.23638    C:0.27524    A:0.16667    G:0.32171
Average         T:0.23678    C:0.24720    A:0.22529    G:0.29073


Nei & Gojobori 1986. dN/dS (dN, dS)
(Note: This matrix is not used in later ML. analysis.
Use runmode = -2 for ML pairwise comparison.)

human               
chimpanzee           0.3317 (0.0032 0.0097)
gorilla              0.1978 (0.0032 0.0162) 0.0817 (0.0021 0.0262)
orangutan            0.0462 (0.0021 0.0463) 0.0215 (0.0011 0.0498) 0.0214 (0.0011 0.0498)
gibbon               0.0638 (0.0043 0.0672) 0.0454 (0.0032 0.0707) 0.0411 (0.0032 0.0780) 0.0262 (0.0021 0.0814)
rhesus               0.0632 (0.0054 0.0849) 0.0526 (0.0043 0.0813) 0.0483 (0.0043 0.0887) 0.0413 (0.0032 0.0778) 0.0518 (0.0054 0.1033)


TREE #  1:  ((((1, 2), 3), 4), 5, 6);   MP score: 62
lnL(ntime:  9  np: 12):  -2038.453022      +0.000000
   7..8     8..9     9..10   10..1    10..2     9..3     8..4     7..5     7..6  
 0.005711 0.012363 0.004841 0.004879 0.009843 0.009992 0.017672 0.043165 0.048845 7.899027 0.057326 999.000000

Note: Branch length is defined as number of nucleotide substitutions per codon (not per neucleotide site).

tree length =   0.15731

((((1: 0.004879, 2: 0.009843): 0.004841, 3: 0.009992): 0.012363, 4: 0.017672): 0.005711, 5: 0.043165, 6: 0.048845);

((((human: 0.004879, chimpanzee: 0.009843): 0.004841, gorilla: 0.009992): 0.012363, orangutan: 0.017672): 0.005711, gibbon: 0.043165, rhesus: 0.048845);

Detailed output identifying parameters

kappa (ts/tv) =  7.89903

w (dN/dS) for branches:  0.05733 999.00000

dN & dS for each branch

 branch          t       N       S   dN/dS      dN      dS  N*dN  S*dS

   7..8      0.006   857.7   390.3  0.0573  0.0003  0.0054   0.3   2.1
   8..9      0.012   857.7   390.3  0.0573  0.0007  0.0117   0.6   4.6
   9..10     0.005   857.7   390.3  0.0573  0.0003  0.0046   0.2   1.8
  10..1      0.005   857.7   390.3 999.0000  0.0024  0.0000   2.0   0.0
  10..2      0.010   857.7   390.3  0.0573  0.0005  0.0093   0.5   3.6
   9..3      0.010   857.7   390.3  0.0573  0.0005  0.0095   0.5   3.7
   8..4      0.018   857.7   390.3  0.0573  0.0010  0.0167   0.8   6.5
   7..5      0.043   857.7   390.3  0.0573  0.0023  0.0409   2.0  15.9
   7..6      0.049   857.7   390.3  0.0573  0.0027  0.0462   2.3  18.0

tree length for dN:       0.0106
tree length for dS:       0.1443

dS tree:
((((human: 0.000002, chimpanzee: 0.009319): 0.004583, gorilla: 0.009459): 0.011704, orangutan: 0.016730): 0.005406, gibbon: 0.040864, rhesus: 0.046241);
dN tree:
((((human: 0.002365, chimpanzee: 0.000534): 0.000263, gorilla: 0.000542): 0.000671, orangutan: 0.000959): 0.000310, gibbon: 0.002343, rhesus: 0.002651);

w ratios as labels for TreeView:
((((human #999.0000 , chimpanzee #0.0573 ) #0.0573 , gorilla #0.0573 ) #0.0573 , orangutan #0.0573 ) #0.0573 , gibbon #0.0573 , rhesus #0.0573 );


Time used:  0:06
```

---

**13. Main result file for "Model A"**

```
CODONML (in paml version 4.8a, July 2014)  apmap.phy
Model: several dN/dS ratios for branches for branches, 
Codon frequency model: F3x4
Site-class models:  PositiveSelection
ns =   6  ls = 416

Codon usage in sequences
--------------------------------------------------------------------------------------------------------------------------------------
Phe TTT  14  14  14  14  14  15 | Ser TCT   8   8   8   8   8   7 | Tyr TAT   2   2   2   1   1   2 | Cys TGT   1   1   1   1   1   1
    TTC   9   9   9   9  10   9 |     TCC   7   7   7   7   6   7 |     TAC   7   7   7   8   8   7 |     TGC   2   2   2   2   2   2
Leu TTA   2   2   2   2   2   2 |     TCA   0   0   1   1   1   1 | *** TAA   0   0   0   0   0   0 | *** TGA   0   0   0   0   0   0
    TTG   3   3   4   4   5   4 |     TCG   2   2   1   1   2   1 |     TAG   0   0   0   0   0   0 | Trp TGG   4   4   4   4   4   4
--------------------------------------------------------------------------------------------------------------------------------------
Leu CTT   6   6   6   6   6   6 | Pro CCT   8  10   8   8   8   8 | His CAT   3   3   4   3   3   3 | Arg CGT   2   2   2   2   2   2
    CTC  10  10   9  10   9  10 |     CCC  11   9  11  11  11  10 |     CAC   3   3   2   3   3   3 |     CGC   3   3   3   3   3   3
    CTA   2   2   2   2   4   2 |     CCA   3   3   3   3   4   3 | Gln CAA   3   3   4   3   3   1 |     CGA   6   6   5   6   6   6
    CTG  27  27  27  26  23  26 |     CCG   6   6   6   6   5   7 |     CAG   9   9   9   9   9  11 |     CGG   8   8   8   8   9   8
--------------------------------------------------------------------------------------------------------------------------------------
Ile ATT   6   7   7   7   7   5 | Thr ACT   4   4   5   5   4   6 | Asn AAT   7   7   7   7   6   7 | Ser AGT   1   1   1   3   1   2
    ATC   4   4   4   4   4   5 |     ACC   9   9   8   9   8   6 |     AAC   4   3   3   3   4   3 |     AGC  14  14  14  12  14  13
    ATA   4   4   4   4   4   4 |     ACA   6   6   6   5   5   4 | Lys AAA   8   9   9   8   9   9 | Arg AGA   7   7   7   7   7   7
Met ATG  14  14  14  14  14  14 |     ACG   2   2   2   2   4   4 |     AAG   8   8   8   9   8   8 |     AGG   6   7   6   6   6   6
--------------------------------------------------------------------------------------------------------------------------------------
Val GTT   8   7   7   7  10   8 | Ala GCT   4   5   4   5   5   5 | Asp GAT  18  18  18  17  17  16 | Gly GGT   5   5   5   5   4   5
    GTC   8   8   8   8   6   9 |     GCC   7   6   7   6   6   7 |     GAC   9   9   9  10  10  11 |     GGC  10  10  10  10  10  10
    GTA   3   3   3   4   3   3 |     GCA   7   7   7   6   7   7 | Glu GAA  13  13  13  12  12  12 |     GGA   5   5   5   5   5   5
    GTG  15  15  15  14  14  14 |     GCG   1   1   1   2   1   1 |     GAG  13  13  13  14  14  14 |     GGG  15  14  15  15  15  15
--------------------------------------------------------------------------------------------------------------------------------------

Codon position x base (3x4) table for each sequence.

#1: human          
position  1:    T:0.14663    C:0.26442    A:0.25000    G:0.33894
position  2:    T:0.32452    C:0.20433    A:0.25721    G:0.21394
position  3:    T:0.23317    C:0.28125    A:0.16587    G:0.31971
Average         T:0.23478    C:0.25000    A:0.22436    G:0.29087

#2: chimpanzee     
position  1:    T:0.14663    C:0.26442    A:0.25481    G:0.33413
position  2:    T:0.32452    C:0.20433    A:0.25721    G:0.21394
position  3:    T:0.24038    C:0.27163    A:0.16827    G:0.31971
Average         T:0.23718    C:0.24679    A:0.22676    G:0.28926

#3: gorilla        
position  1:    T:0.14904    C:0.26202    A:0.25240    G:0.33654
position  2:    T:0.32452    C:0.20433    A:0.25962    G:0.21154
position  3:    T:0.23798    C:0.27163    A:0.17067    G:0.31971
Average         T:0.23718    C:0.24599    A:0.22756    G:0.28926

#4: orangutan      
position  1:    T:0.14904    C:0.26202    A:0.25240    G:0.33654
position  2:    T:0.32452    C:0.20433    A:0.25721    G:0.21394
position  3:    T:0.23798    C:0.27644    A:0.16346    G:0.32212
Average         T:0.23718    C:0.24760    A:0.22436    G:0.29087

#5: gibbon         
position  1:    T:0.15385    C:0.25962    A:0.25240    G:0.33413
position  2:    T:0.32452    C:0.20433    A:0.25721    G:0.21394
position  3:    T:0.23317    C:0.27404    A:0.17308    G:0.31971
Average         T:0.23718    C:0.24599    A:0.22756    G:0.28926

#6: rhesus         
position  1:    T:0.14904    C:0.26202    A:0.24760    G:0.34135
position  2:    T:0.32692    C:0.20192    A:0.25721    G:0.21394
position  3:    T:0.23558    C:0.27644    A:0.15865    G:0.32933
Average         T:0.23718    C:0.24679    A:0.22115    G:0.29487

Sums of codon usage counts
------------------------------------------------------------------------------
Phe F TTT      85 | Ser S TCT      47 | Tyr Y TAT      10 | Cys C TGT       6
      TTC      55 |       TCC      41 |       TAC      44 |       TGC      12
Leu L TTA      12 |       TCA       4 | *** * TAA       0 | *** * TGA       0
      TTG      23 |       TCG       9 |       TAG       0 | Trp W TGG      24
------------------------------------------------------------------------------
Leu L CTT      36 | Pro P CCT      50 | His H CAT      19 | Arg R CGT      12
      CTC      58 |       CCC      63 |       CAC      17 |       CGC      18
      CTA      14 |       CCA      19 | Gln Q CAA      17 |       CGA      35
      CTG     156 |       CCG      36 |       CAG      56 |       CGG      49
------------------------------------------------------------------------------
Ile I ATT      39 | Thr T ACT      28 | Asn N AAT      41 | Ser S AGT       9
      ATC      25 |       ACC      49 |       AAC      20 |       AGC      81
      ATA      24 |       ACA      32 | Lys K AAA      52 | Arg R AGA      42
Met M ATG      84 |       ACG      16 |       AAG      49 |       AGG      37
------------------------------------------------------------------------------
Val V GTT      47 | Ala A GCT      28 | Asp D GAT     104 | Gly G GGT      29
      GTC      47 |       GCC      39 |       GAC      58 |       GGC      60
      GTA      19 |       GCA      41 | Glu E GAA      75 |       GGA      30
      GTG      87 |       GCG       7 |       GAG      81 |       GGG      89
------------------------------------------------------------------------------


Codon position x base (3x4) table, overall

position  1:    T:0.14904    C:0.26242    A:0.25160    G:0.33694
position  2:    T:0.32492    C:0.20393    A:0.25761    G:0.21354
position  3:    T:0.23638    C:0.27524    A:0.16667    G:0.32171
Average         T:0.23678    C:0.24720    A:0.22529    G:0.29073


Nei & Gojobori 1986. dN/dS (dN, dS)
(Note: This matrix is not used in later ML. analysis.
Use runmode = -2 for ML pairwise comparison.)

human               
chimpanzee           0.3317 (0.0032 0.0097)
gorilla              0.1978 (0.0032 0.0162) 0.0817 (0.0021 0.0262)
orangutan            0.0462 (0.0021 0.0463) 0.0215 (0.0011 0.0498) 0.0214 (0.0011 0.0498)
gibbon               0.0638 (0.0043 0.0672) 0.0454 (0.0032 0.0707) 0.0411 (0.0032 0.0780) 0.0262 (0.0021 0.0814)
rhesus               0.0632 (0.0054 0.0849) 0.0526 (0.0043 0.0813) 0.0483 (0.0043 0.0887) 0.0413 (0.0032 0.0778) 0.0518 (0.0054 0.1033)


TREE #  1:  ((((1, 2), 3), 4), 5, 6);   MP score: 62
lnL(ntime:  9  np: 14):  -2038.453022      +0.000000
   7..8     8..9     9..10   10..1    10..2     9..3     8..4     7..5     7..6  
 0.005711 0.012362 0.004841 0.004879 0.009843 0.009992 0.017672 0.043165 0.048845 7.898971 0.000000 0.000000 0.057325 999.000000

Note: Branch length is defined as number of nucleotide substitutions per codon (not per neucleotide site).

tree length =   0.15731

((((1: 0.004879, 2: 0.009843): 0.004841, 3: 0.009992): 0.012362, 4: 0.017672): 0.005711, 5: 0.043165, 6: 0.048845);

((((human: 0.004879, chimpanzee: 0.009843): 0.004841, gorilla: 0.009992): 0.012362, orangutan: 0.017672): 0.005711, gibbon: 0.043165, rhesus: 0.048845);

Detailed output identifying parameters

kappa (ts/tv) =  7.89897


dN/dS (w) for site classes (K=4)

site class             0        1       2a       2b
proportion       0.00000  0.00000  1.00000  0.00000
background w     0.05732  1.00000  0.05732  1.00000
foreground w     0.05732  1.00000 999.00000 999.00000


Bayes Empirical Bayes (BEB) analysis (Yang, Wong & Nielsen 2005. Mol. Biol. Evol. 22:1107-1118)
Positive sites for foreground lineages Prob(w>1):
   100 V 0.828
   196 N 0.953*


The grid (see ternary graph for p0-p1)

w0:   0.050  0.150  0.250  0.350  0.450  0.550  0.650  0.750  0.850  0.950
w2:   1.500  2.500  3.500  4.500  5.500  6.500  7.500  8.500  9.500 10.500


Posterior on the grid

w0:   0.998  0.002  0.000  0.000  0.000  0.000  0.000  0.000  0.000  0.000
w2:   0.012  0.023  0.038  0.055  0.075  0.099  0.126  0.156  0.189  0.226

Posterior for p0-p1 (see the ternary graph)

 0.000
 0.000 0.000 0.000
 0.000 0.000 0.000 0.000 0.000
 0.000 0.000 0.000 0.000 0.000 0.000 0.000
 0.000 0.000 0.000 0.000 0.000 0.000 0.000 0.000 0.000
 0.000 0.000 0.000 0.000 0.000 0.000 0.000 0.000 0.000 0.000 0.000
 0.000 0.000 0.000 0.000 0.000 0.000 0.000 0.000 0.000 0.000 0.000 0.000 0.000
 0.000 0.000 0.000 0.000 0.000 0.000 0.000 0.000 0.000 0.000 0.000 0.000 0.000 0.000 0.000
 0.000 0.000 0.000 0.000 0.000 0.000 0.000 0.000 0.000 0.000 0.000 0.000 0.000 0.000 0.000 0.000 0.000
 0.000 0.000 0.000 0.000 0.016 0.000 0.092 0.002 0.182 0.008 0.221 0.017 0.198 0.023 0.135 0.020 0.065 0.010 0.013

sum of density on p0-p1 =   1.000000

Time used:  0:30
```

---

**14. Main result file for "Null model A"**

```
CODONML (in paml version 4.8a, July 2014)  apmap.phy
Model: several dN/dS ratios for branches for branches,  omega = 1.000 fixed

Codon frequency model: F3x4
Site-class models:  PositiveSelection
ns =   6  ls = 416

Codon usage in sequences
--------------------------------------------------------------------------------------------------------------------------------------
Phe TTT  14  14  14  14  14  15 | Ser TCT   8   8   8   8   8   7 | Tyr TAT   2   2   2   1   1   2 | Cys TGT   1   1   1   1   1   1
    TTC   9   9   9   9  10   9 |     TCC   7   7   7   7   6   7 |     TAC   7   7   7   8   8   7 |     TGC   2   2   2   2   2   2
Leu TTA   2   2   2   2   2   2 |     TCA   0   0   1   1   1   1 | *** TAA   0   0   0   0   0   0 | *** TGA   0   0   0   0   0   0
    TTG   3   3   4   4   5   4 |     TCG   2   2   1   1   2   1 |     TAG   0   0   0   0   0   0 | Trp TGG   4   4   4   4   4   4
--------------------------------------------------------------------------------------------------------------------------------------
Leu CTT   6   6   6   6   6   6 | Pro CCT   8  10   8   8   8   8 | His CAT   3   3   4   3   3   3 | Arg CGT   2   2   2   2   2   2
    CTC  10  10   9  10   9  10 |     CCC  11   9  11  11  11  10 |     CAC   3   3   2   3   3   3 |     CGC   3   3   3   3   3   3
    CTA   2   2   2   2   4   2 |     CCA   3   3   3   3   4   3 | Gln CAA   3   3   4   3   3   1 |     CGA   6   6   5   6   6   6
    CTG  27  27  27  26  23  26 |     CCG   6   6   6   6   5   7 |     CAG   9   9   9   9   9  11 |     CGG   8   8   8   8   9   8
--------------------------------------------------------------------------------------------------------------------------------------
Ile ATT   6   7   7   7   7   5 | Thr ACT   4   4   5   5   4   6 | Asn AAT   7   7   7   7   6   7 | Ser AGT   1   1   1   3   1   2
    ATC   4   4   4   4   4   5 |     ACC   9   9   8   9   8   6 |     AAC   4   3   3   3   4   3 |     AGC  14  14  14  12  14  13
    ATA   4   4   4   4   4   4 |     ACA   6   6   6   5   5   4 | Lys AAA   8   9   9   8   9   9 | Arg AGA   7   7   7   7   7   7
Met ATG  14  14  14  14  14  14 |     ACG   2   2   2   2   4   4 |     AAG   8   8   8   9   8   8 |     AGG   6   7   6   6   6   6
--------------------------------------------------------------------------------------------------------------------------------------
Val GTT   8   7   7   7  10   8 | Ala GCT   4   5   4   5   5   5 | Asp GAT  18  18  18  17  17  16 | Gly GGT   5   5   5   5   4   5
    GTC   8   8   8   8   6   9 |     GCC   7   6   7   6   6   7 |     GAC   9   9   9  10  10  11 |     GGC  10  10  10  10  10  10
    GTA   3   3   3   4   3   3 |     GCA   7   7   7   6   7   7 | Glu GAA  13  13  13  12  12  12 |     GGA   5   5   5   5   5   5
    GTG  15  15  15  14  14  14 |     GCG   1   1   1   2   1   1 |     GAG  13  13  13  14  14  14 |     GGG  15  14  15  15  15  15
--------------------------------------------------------------------------------------------------------------------------------------

Codon position x base (3x4) table for each sequence.

#1: human          
position  1:    T:0.14663    C:0.26442    A:0.25000    G:0.33894
position  2:    T:0.32452    C:0.20433    A:0.25721    G:0.21394
position  3:    T:0.23317    C:0.28125    A:0.16587    G:0.31971
Average         T:0.23478    C:0.25000    A:0.22436    G:0.29087

#2: chimpanzee     
position  1:    T:0.14663    C:0.26442    A:0.25481    G:0.33413
position  2:    T:0.32452    C:0.20433    A:0.25721    G:0.21394
position  3:    T:0.24038    C:0.27163    A:0.16827    G:0.31971
Average         T:0.23718    C:0.24679    A:0.22676    G:0.28926

#3: gorilla        
position  1:    T:0.14904    C:0.26202    A:0.25240    G:0.33654
position  2:    T:0.32452    C:0.20433    A:0.25962    G:0.21154
position  3:    T:0.23798    C:0.27163    A:0.17067    G:0.31971
Average         T:0.23718    C:0.24599    A:0.22756    G:0.28926

#4: orangutan      
position  1:    T:0.14904    C:0.26202    A:0.25240    G:0.33654
position  2:    T:0.32452    C:0.20433    A:0.25721    G:0.21394
position  3:    T:0.23798    C:0.27644    A:0.16346    G:0.32212
Average         T:0.23718    C:0.24760    A:0.22436    G:0.29087

#5: gibbon         
position  1:    T:0.15385    C:0.25962    A:0.25240    G:0.33413
position  2:    T:0.32452    C:0.20433    A:0.25721    G:0.21394
position  3:    T:0.23317    C:0.27404    A:0.17308    G:0.31971
Average         T:0.23718    C:0.24599    A:0.22756    G:0.28926

#6: rhesus         
position  1:    T:0.14904    C:0.26202    A:0.24760    G:0.34135
position  2:    T:0.32692    C:0.20192    A:0.25721    G:0.21394
position  3:    T:0.23558    C:0.27644    A:0.15865    G:0.32933
Average         T:0.23718    C:0.24679    A:0.22115    G:0.29487

Sums of codon usage counts
------------------------------------------------------------------------------
Phe F TTT      85 | Ser S TCT      47 | Tyr Y TAT      10 | Cys C TGT       6
      TTC      55 |       TCC      41 |       TAC      44 |       TGC      12
Leu L TTA      12 |       TCA       4 | *** * TAA       0 | *** * TGA       0
      TTG      23 |       TCG       9 |       TAG       0 | Trp W TGG      24
------------------------------------------------------------------------------
Leu L CTT      36 | Pro P CCT      50 | His H CAT      19 | Arg R CGT      12
      CTC      58 |       CCC      63 |       CAC      17 |       CGC      18
      CTA      14 |       CCA      19 | Gln Q CAA      17 |       CGA      35
      CTG     156 |       CCG      36 |       CAG      56 |       CGG      49
------------------------------------------------------------------------------
Ile I ATT      39 | Thr T ACT      28 | Asn N AAT      41 | Ser S AGT       9
      ATC      25 |       ACC      49 |       AAC      20 |       AGC      81
      ATA      24 |       ACA      32 | Lys K AAA      52 | Arg R AGA      42
Met M ATG      84 |       ACG      16 |       AAG      49 |       AGG      37
------------------------------------------------------------------------------
Val V GTT      47 | Ala A GCT      28 | Asp D GAT     104 | Gly G GGT      29
      GTC      47 |       GCC      39 |       GAC      58 |       GGC      60
      GTA      19 |       GCA      41 | Glu E GAA      75 |       GGA      30
      GTG      87 |       GCG       7 |       GAG      81 |       GGG      89
------------------------------------------------------------------------------


Codon position x base (3x4) table, overall

position  1:    T:0.14904    C:0.26242    A:0.25160    G:0.33694
position  2:    T:0.32492    C:0.20393    A:0.25761    G:0.21354
position  3:    T:0.23638    C:0.27524    A:0.16667    G:0.32171
Average         T:0.23678    C:0.24720    A:0.22529    G:0.29073


Nei & Gojobori 1986. dN/dS (dN, dS)
(Note: This matrix is not used in later ML. analysis.
Use runmode = -2 for ML pairwise comparison.)

human               
chimpanzee           0.3317 (0.0032 0.0097)
gorilla              0.1978 (0.0032 0.0162) 0.0817 (0.0021 0.0262)
orangutan            0.0462 (0.0021 0.0463) 0.0215 (0.0011 0.0498) 0.0214 (0.0011 0.0498)
gibbon               0.0638 (0.0043 0.0672) 0.0454 (0.0032 0.0707) 0.0411 (0.0032 0.0780) 0.0262 (0.0021 0.0814)
rhesus               0.0632 (0.0054 0.0849) 0.0526 (0.0043 0.0813) 0.0483 (0.0043 0.0887) 0.0413 (0.0032 0.0778) 0.0518 (0.0054 0.1033)


TREE #  1:  ((((1, 2), 3), 4), 5, 6);   MP score: 62
lnL(ntime:  9  np: 13):  -2039.025313      +0.000000
   7..8     8..9     9..10   10..1    10..2     9..3     8..4     7..5     7..6  
 0.005709 0.011886 0.004826 0.005313 0.009445 0.010408 0.017729 0.043105 0.048907 7.843518 0.000000 0.000000 0.057461

Note: Branch length is defined as number of nucleotide substitutions per codon (not per neucleotide site).

tree length =   0.15733

((((1: 0.005313, 2: 0.009445): 0.004826, 3: 0.010408): 0.011886, 4: 0.017729): 0.005709, 5: 0.043105, 6: 0.048907);

((((human: 0.005313, chimpanzee: 0.009445): 0.004826, gorilla: 0.010408): 0.011886, orangutan: 0.017729): 0.005709, gibbon: 0.043105, rhesus: 0.048907);

Detailed output identifying parameters

kappa (ts/tv) =  7.84352


dN/dS (w) for site classes (K=4)

site class             0        1       2a       2b
proportion       0.00000  0.00000  1.00000  0.00000
background w     0.05746  1.00000  0.05746  1.00000
foreground w     0.05746  1.00000  1.00000  1.00000


Time used:  0:25
```

---
