# Supplementary material for: The acquisition of novel N-glycosylation sites in conserved proteins during human evolution
Source: BMC Bioinformatics. 2015 Jan 28;16(1):29. doi: 10.1186/s12859-015-0468-5 (PMC4314935; doi:10.1186/s12859-015-0468-5)
Supplement: Additional file 5: — Molecular evolutionary analysis of CD166. [file 12859_2015_468_MOESM5_ESM.zip › 12859_2015_468_MOESM5_ESM.html]

## Additional file 5. Molecular evolutionary analysis of CD166.

**1. Multiple sequence alignment of selected primate orthologs (positive sites by Model A are in magenta background)**

```
human       MESKGASSCRLLFCLLISATVFRPGLGWYTVNSAYGDTIIIPCRLDVPQNLMFGKWKYEKPDGSPVFIAFRSSTKKSVQYDDVPEYKDRLNLSENYTLSI  100
chimpanzee  ..........................................................................................S.........  100
gorilla     ........................................M.................................................S.........  100
orangutan   ................V.S.....................M.................................................S.........  100
gibbon      ........................................M.................................................S.........  100
rhesus      .....................L..................M.................................................S.........  100


human       SNARISDEKRFVCMLVTEDNVFEAPTIVKVFKQPSKPEIVSKALFLETEQLKKLGDCISEDSYPDGNITWYRNGKVLHPLEGAVVIIFKKEMDPVTQLYT  200
chimpanzee  ....................................................................................................  200
gorilla     ....................................................................................................  200
orangutan   ....................................................................................................  200
gibbon      ....................................................................................................  200
rhesus      ..........................V..............................V..........................................  200


human       MTSTLEYKTTKADIQMPFTCSVTYYGPSGQKTIHSEQAVFDIYYPTEQVTIQVLPPKNAIKEGDNITLKCLGNGNPPPEEFLFYLPGQPEGIRSSNTYTL  300
chimpanzee  ....................................................................................................  300
gorilla     ....................................................................................................  300
orangutan   ....................................................................................................  300
gibbon      ....................................R...............................................................  300
rhesus      ................................V........................T.......V..................................  300


human       TDVRRNATGDYKCSLIDKKSMIASTAITVHYLDLSLNPSGEVTRQIGDALPVSCTISASRNATVVWMKDNIRLRSSPSFSSLHYQDAGNYVCETALQEVE  400
chimpanzee  ....................................................................................................  400
gorilla     ....................................................................................................  400
orangutan   ....................................................................................................  400
gibbon      ....................................................................................................  400
rhesus      ....................................................................................................  400


human       GLKKRESLTLIVEGKPQIKMTKKTDPSGLSKTIICHVEGFPKPAIQWTITGSGSVINQTEESPYINGRYYSKIIISPEENVTLTCTAENQLERTVNSLNV  500
chimpanzee  ....................................................................................................  500
gorilla     ....................................................................................................  500
orangutan   ....................................................................................................  500
gibbon      ....................................................................................................  500
rhesus      ....................................................................................................  500


human       SAISIPEHDEADEISDENREKVNDQAKLIVGIVVGLLLAALVAGVVYWLYMKKSKTASKHVNKDLGNMEENKKLEENNHKTEA  583
chimpanzee  ...................................................................................  583
gorilla     ...................................................................................  583
orangutan   ...................................................................................  583
gibbon      .......................................................A...........................  583
rhesus      ...................................................................................  583
```

---

**2. Sequence data file "cd166.phy"**

```
6 1749

human
ATG GAA TCC AAG GGG GCC AGT TCC TGC CGT CTG CTC TTC TGC CTC TTG ATC TCC GCC ACC GTC TTC
AGG CCA GGC CTT GGA TGG TAT ACT GTA AAT TCA GCA TAT GGA GAT ACC ATT ATC ATA CCT TGC CGA
CTT GAC GTA CCT CAG AAT CTC ATG TTT GGC AAA TGG AAA TAT GAA AAG CCC GAT GGC TCC CCA GTA
TTT ATT GCC TTC AGA TCC TCT ACA AAG AAA AGT GTG CAG TAC GAC GAT GTA CCA GAA TAC AAA GAC
AGA TTG AAC CTC TCA GAA AAC TAC ACT TTG TCT ATC AGT AAT GCA AGG ATC AGT GAT GAA AAG AGA
TTT GTG TGC ATG CTA GTA ACT GAG GAC AAC GTG TTT GAG GCA CCT ACA ATA GTC AAG GTG TTC AAG
CAA CCA TCT AAA CCT GAA ATT GTA AGC AAA GCA CTG TTT CTC GAA ACA GAG CAG CTA AAA AAG TTG
GGT GAC TGC ATT TCA GAA GAC AGT TAT CCA GAT GGC AAT ATC ACA TGG TAC AGG AAT GGA AAA GTG
CTA CAT CCC CTT GAA GGA GCG GTG GTC ATA ATT TTT AAA AAG GAA ATG GAC CCA GTG ACT CAG CTC
TAT ACC ATG ACT TCC ACC CTG GAG TAC AAG ACA ACC AAG GCT GAC ATA CAA ATG CCA TTC ACC TGC
TCG GTG ACA TAT TAT GGA CCA TCT GGC CAG AAA ACA ATT CAT TCT GAA CAG GCA GTA TTT GAT ATT
TAC TAT CCT ACA GAG CAG GTG ACA ATA CAA GTG CTG CCA CCA AAA AAT GCC ATC AAA GAA GGG GAT
AAC ATC ACT CTT AAA TGC TTA GGG AAT GGC AAC CCT CCC CCA GAG GAA TTT TTG TTT TAC TTA CCA
GGA CAG CCC GAA GGA ATA AGA AGC TCA AAT ACT TAC ACA CTG ACG GAT GTG AGG CGC AAT GCA ACA
GGA GAC TAC AAG TGT TCC CTG ATA GAC AAA AAA AGC ATG ATT GCT TCA ACA GCT ATC ACA GTT CAC
TAT TTG GAT TTG TCC TTA AAC CCA AGT GGA GAA GTG ACT AGA CAG ATT GGT GAT GCC CTA CCC GTG
TCA TGC ACA ATA TCT GCT AGC AGG AAT GCA ACT GTG GTA TGG ATG AAA GAT AAC ATC AGG CTT CGA
TCT AGC CCG TCA TTT TCT AGT CTT CAT TAT CAG GAT GCT GGA AAC TAT GTC TGC GAA ACT GCT CTG
CAG GAG GTT GAA GGA CTA AAG AAA AGA GAG TCA TTG ACT CTC ATT GTA GAA GGC AAA CCT CAA ATA
AAA ATG ACA AAG AAA ACT GAT CCC AGT GGA CTA TCT AAA ACA ATA ATC TGC CAT GTG GAA GGT TTT
CCA AAG CCA GCC ATT CAA TGG ACA ATT ACT GGC AGT GGA AGC GTC ATA AAC CAA ACA GAG GAA TCT
CCT TAT ATT AAT GGC AGG TAT TAT AGT AAA ATT ATC ATT TCC CCT GAA GAG AAT GTT ACA TTA ACT
TGC ACA GCA GAA AAC CAA CTG GAG AGA ACA GTA AAC TCC TTG AAT GTC TCT GCT ATA AGT ATT CCA
GAA CAC GAT GAG GCA GAC GAG ATA AGT GAT GAA AAC AGA GAA AAG GTG AAT GAC CAG GCA AAA CTA
ATT GTG GGA ATC GTT GTT GGT CTC CTC CTT GCT GCC CTT GTT GCT GGT GTC GTC TAC TGG CTG TAC
ATG AAG AAG TCA AAG ACT GCA TCA AAA CAT GTA AAC AAG GAC CTC GGT AAT ATG GAA GAA AAC AAA
AAG TTA GAA GAA AAC AAT CAC AAA ACT GAA GCC
chimpanzee
... ... ... ... ... ... ... ... ... ... ... ... ... ... ... ... ... ... ... ... ... ...
... ... ... ... ... ... ... ... ... ... ... ... ... ... ... ... ... ... ... ... ... ...
... ..T ... ... ... ... ... ... ... ... ... ... ... ... ... ... ... ... ... ... ... ...
... ... ... ... ... ... ... ... ... ... ... ... ... ... ... ... ... ... ... ... ... ...
... ... .G. ... ... ... ... ... ..G ... ... ... ... ... ... ... ... ... ..C ... ... ...
... ... ... ... ... ... ... ... ... ... ... ... ... ... ... ... ... ... ... ... ... ...
... ... ... ... ... ... ... ... ... ... ... ... ... ... ... ... ... ... ... ... ... ...
... ... ... ... ... ... ... ... ... ... ... ... ... ... ... ... ... ... ... ... ... ...
... ... ... ... ... ... ... ... ... ... ... ... ... ... ... ... ... ... ... ... ... ...
... ... ... ... ... ... ... ... ... ... ... ... ... ... ... ... ... ... ... ... ... ...
... ... ... ... ... ... ... ... ... ... ... ... ... ... ... ... ... ... ... ... ... ...
... ... ... ... ... ... ... ... ... ... ... ... ... ... ... ... ... ... ... ... ... ...
... ... ... ... ... ... ... ... ... ... ... ... ... ... ... ... ... ... ... ... ... ...
... ... ..T ... ... ... ... ... ... ... ... ... ... ... ..A ... ... ... ... ... ... ...
... ... ... ... ... ... ... ... ... ... ... ... ... ... ... ... ... ..C ... ... ... ...
... ... ... ... ... ... ... ..C ... ... ... ... ..C ... ... ... ... ... ... ... ... ...
... ... ... ... ..C ... ... ... ... ... ... ... ... ... ... ... ... ... ... ... ... ...
... ... ... ... ... ... ... ... ... ... ... ... ... ... ... ... ... ... ... ... ... ...
... ... ... ... ... ... ... ... ... ... ... ... ... ... ... ... ... ... ... ... ... ...
... ... ... ... ... ... ... ... ... ... ... ... ... ... ... ... ... ... ... ... ... ...
... ... ... ... ... ... ... ... ... ... ... ... ... ... ... ... ... ... ... ... ... ...
... ... ... ... ... ... ... ... ... ... ... ... ... ... ... ... ... ... ... ... ... ...
... ... ... ... ... ... ... ... ... ... ... ... ... C.. ... ... ... ... ... ... ... ...
... ... ... ... ... ... ... ... ... ... ... ... ... ... ... ... ... ... ... ... ... ...
... ... ... ... ... ... ... ... ... ... ... ... ... ... ... ... ... ... ... ... ... ...
... ... ... ... ... ... ... ... ... ... ... ... ..A ... ... ... ... ... ... ... ... ...
... ... ... ... ... ... ... ... ... ... ...
gorilla
... ... ... ... ... ... ... ... ... ... ... ... ... ... ... ... ... ... ... ..T ... ...
... ... ... ... ... ... ... ... ... ... ... ... ... ... ... ... ... ... ..G ... ... ...
... ... ... ... ... ... ... ... ... ... ... ... ... ... ... ... ... ... ... ... ... ...
... ... ... ... ... ... ... ... ... ... ... ... ... ... ... ... ... ... ... ... ... ...
... ... .G. ... ... ... ... ... ... ... ... ... ... ... ... ... ... ... ..C ... ... ...
... ... ... ... ... ... ... ... ... ... ... ... ... ... ... ... ... ... ... ... ... ...
... ... ... ... ... ... ... ... ... ... ... ... ... ... ... ... ... ... ... ... ... ...
... ... ... ... ... ... ... ... ... ... ... ... ... ... ... ... ... ... ... ... ... ...
... ... ... ... ... ... ... ... ... ... ... ... ... ... ... ... ... ... ... ... ... ...
... ... ... ... ... ... ... ... ... ... ... ... ... ... ... ... ... ... ... ... ... ...
... ... ... ... ... ... ... ... ... ... ... ... ... ... ... ... ... ... ... ... ... ...
... ... ... ... ... ... ... ... ... ... ... ... ... ... ... ... ... ... ... ... ... ...
... ... ... ... ... ... ... ... ... ... ... ... ... ... ... ... ... ... ... ... ... ...
... ... ... ... ... ... ... ... ... ... ... ... ... ... ..A ... ... ... ... ... ... ...
... ... ... ... ... ... ... ... ... ... ... ... ... ... ... ... ... ..C ... ... ... ...
... ... ... ... ... ... ... ... ... ... ... ... ..C ... ... ... ... ... ... ... ... ...
... ... ... ... ..C ... ... ..A ... ... ... ... ... ... ... ... ... ... ... ... ... ...
... ... ... ... ... ... ... ... ... ... ... ... ... ... ... ... ... ... ... ... ... ...
... ... ... ... ... ... ... ... ... ... ... ... ... ... ... ... ... ... ... ... ... ...
... ... ... ... ... ... ... ... ... ... ... ... ... ... ... ... ... ... ... ... ... ...
... ... ... ... ... ... ... ... ... ... ... ... ... ... ... ... ... ... ... ... ... ...
... ... ..C ... ... ... ... ... ... ... ... ... ... ... ... ... ... ... ... ... ... ...
... ... ... ... ... ... ... ... ... ... ... ... ... ... ... ... ... ... ... ... ... ...
... ... ... ... ... ... ... ... ... ... ... ... ... ... ... ... ... ... ... ... ... ...
... ... ... ... ... ... ... ... ... ... ... ... ... ... ... ... ... ... ... ... ... ...
... ... ... ... ... ... ... ... ... ... ... ... ... ... ... ... ... ... ... ... ... ...
... ... ... ... ... ... ... ... ... ... ...
orangutan
... ... ... ... ... ... ... ... ... ... ... ... ... ... ... ... G.. ... T.. ... ... ...
... ... ... ... ... ... ... ... ... ... ... ... ... ... ... ... ... ... ..G ... ... ...
... ... ... ... ... ... ... ... ... ... ... ... ... ... ... ... ... ... ... ... ... ...
... ... ... ... ..G ... ... ... ... ... ... ... ... ... ... ... ... ... ... ... ... ...
... ... .G. ... ... ... ... ... ... ... ... ... ... ... ... ... ... ... ... ... ... ...
... ... ... ... ... ... ... ... ... ... ... ... ... ... ... ... ... ... ... ... ... ...
... ... ... ... ... ... ... ... ... ... ... ... ... ..A ... ... ... ... ... ... ... ...
... ... ... ... ... ... ... ... ... ... ... ... ... ... ... ... ... ... ... ... ... ...
... ... ... ... ... ... ... ... ... ... ... ... ... ... ... ... ... ... ... ... ... ...
... ... ... ... ... ... ... ... ... ... ... ... ... ... ... ... ... ... ... ... ... ...
... ... ... ... ... ... ... ... ... ... ... ... ... ... ... ... ... ... ... ... ... ...
... ... ... ... ... ... ... ... ... ... ... ... ... ... ... ... ... ... ... ... ... ...
... ... ... ... ... ... ... ... ... ... ... ... ... ... ... ... ... ... ... ... ... ...
... ... ... ... ... ... ... ... ... ... ... ... ... ... ..A ... ... ... ... ... ... ...
... ... ... ... ... ... ... ... ... ... ... ... ... ... ... ... ... ..C ... ... ... ...
... ... ... ... ... ... ... ... ... ... ... ... ..C ... ... ... ... ... ... ... ..T ...
... ... ... ... ..C ... ... ... ..C ... ... ... ... ... ... ... ... ... ... ... ... ...
... ... ... ... ... ... ... ... ... ... ... ... ... ... ... ..C ... ... ... ... ... ...
... ... ... ... ... ... ... ... ... ... ... ... ... ... ... ... ... ... ... ... ... ...
... ... ... ... ... ... ... ... ... ... ... ... ... ... ... ... ... ... ... ... ... ...
... ... ... ... ... ... ... ... ... ... ... ... ... ... ... ... ... ... ... ... ... ...
... ... ..C ... ... ... ... ... ... ... ... ... ... ... ... ... ... ... ... ... ... ...
... ... ... ... ... ... ... ... ... ... ... ... ... ... ... ... ... ... ... ... ... ...
... ... ... ... ... ... ... ... ... ... ... ... ... ... ... ... ... ... ... ... ... ...
... ... ... ... ..C ... ... ... ... ... ... ... ... ... ... ... ... ... ... ... ... ...
... ... ... ... ... ... ... ... ... ... ... ... ... ... ... ... ... ... ... ... ... ...
... ... ... ... ... ... ... ... ... ... ...
gibbon
... ... ... ... ... ... ... ... ... ... ... ... ... ... ... ... ... ... ... ... ... ...
... ... ... ... ... ... ... ... ... ... ... ... ... ... ... ... ..C ... ..G ... ... ...
... ..T ... ... ... ... ... ... ... ... ... ... ... ... ... ... ... ... ... ... ... ...
... ... ... ... ..G ... ... ... ... ... ... ... ... ... ... ... ... ... ... ... ... ...
... ... .G. ... ... ... ... ... ... ... ... ... ... ... ... ... ... ... ... ... ... ...
... ... ... ... ... ... ... ... ... ... ... ... ... ... ... ... ... ... ... ... ... ..A
... ... ... ... ... ... ... ... ... ... ... ..C ... ... ... ... ... ... ... ... ... ...
... ... ... ... ... ... ... ... ... ... ... ... ..C ... ... ... ... ... ... ... ... ...
... ... ... ... ... ... ... ... ... ... ... ... ... ... ... ... ... ... ... ... ... ...
... ... ... ... ... ... ... ... ... ... ... ... ... ... ... ... ... ... ... ... ... ...
..T ... ... ... ... ..G ... ... ... ... ... ... ... ... ... ... .G. ... ... ... ... ...
... ... ... ... ... ... ... ... ... ... ... ... ... ... ... ... ... ... ... ... ... ...
... ... ... ... ... ... ... ... ... ... ... ... ... ... ... ... ... ... ... ... ... ...
... ... ... ... ... ... ... ... ... ... ... ... ... ... ..A ... ... ... ... ... ... ...
... ... ... ... ... ... ... ... ... ... ... ... ... ... ... ... ... ..C ... ... ... ...
... ... ... ... ... ... ... ... ... ... ... ... ..C ... ... ... ... ... ... ... ... ...
... ... ... ... ..C ... ... ... ... ... ... ... ... ... ... ... ... ... ... ... ... ...
... ... ..A ... ... ... ... ... ... ... ... ... ... ... ... ... ... ..T ... ... ... ...
... ... ... ... ... ... ... ... ... ... ... ... ... ... ... ... ... ... ... ... ... ...
... ... ... ... ... ... ... ... ... ... ... ... ... ... ... ... ... ... ... ... ... ...
... ... ... ... ... ... ... ... ... ... ... ... ... ... ... ... ... ... ... ... ... ...
... ... ..C ... ... ... ... ... ... ... ... ... ... ... ... ... ... ... ... ... ... ...
... ... ... ... ... ... ... ... ... ... ... ... ... ... ... ... ... ... ... ... ... ...
... ... ... ... ... ... ... ... ... ... ... ... ... ... ... ... ... ... ... ... ... ...
... ... ... ... ... ... ... ... ... ... ... ... ... ... ... ... ... ... ... ... ... ...
... ... ... ... ... G.. ... ... ... ... ... ... ... ... ... ... ... ... ... ... ... ...
... ... ... ... ... ... ... ... ... ... ...
rhesus
... ... ... ... ..A ... ..C ... ... ... ... ... ... ... ... ... ... ... ... ... ... C..
... ... ..T ... ... ... ..C ... ... ... ... ... ... ... ... ... ... ... ..G ... ... ...
... ..T ... ... ... ... ... ... ... ... ... ... ... ... ... ... ... ... ... ... ..T ...
... ... ... ... ..G ... ... ... ... ... ... ... ... ... ... ... ... ... ... ... ... ...
... ... .G. ... ... ... ... ... ... ... ... ... ... ... ... ... ... ... ... ..G ... ...
... ... ... ... ... ... ... ... ... ... ... ... ... ... ... ... G.. ... ..A ... ... ...
... ... ... ... ... ... ... ... ... ... ... ... ... ... ... ... ... ... ... ... ... ...
... ... ..T G.. ... ... ... ... ... ... ... ... ..C ... ... ... ... ... ... ... ... ...
... ... ... ... ... ... ... ... ... ... ... ... ... ... ... ... ... ... ... ... ... ...
... ... ... ... ... ... ... ... ... ... ... ... ... ... ... ... ... ... ... ... ... ...
..T ..C ... ... ... ... ... ... ... ... ... ... G.. ... ... ... ... ... ... ... ... ...
... ... ... ... ... ... ... ... ... ... ... ... ... ... ... .C. ... ... ... ... ... ...
... G.. ... ... ... ... ... ..A ... ... ..T ... ... ... ... ..G ... ... ... ... ... ...
... ... ... ... ... ... ... ... ... ... ... ... ... ... ..A ... ... ... ... ... ... ...
... ... ... ... ..C ... ... ... ... ... ... ... ... ... ... ... ... ..C ... ... ... ...
... ... ... ... ... ... ... ... ... ... ... ... ..C ... ... ... ... ... ... ... ... ...
... ... ... ... ..C ... ... ... ... ... ... ... ... ... ... ... ... ... ... ... ... ...
... ... ... ... ... ... ... ... ... ... ... ... ... ... ... ... ... ... ... ... ... ...
... ... ... ... ... ... ... ... ... ... ... ... ... ... ... ... ... ... ... ... ... ...
... ... ... ... ... ... ... ... ... ... ... ... ... ... ..C ... ... ..C ... ... ... ...
... ... ... ... ... ... ... ... ... ... ... ... ... ... ... ... ... ... ... ... ... ...
... ... ..C ... ... ... ... ... ... ... ... ... ... ... ... ... ... ... ... ... ... ...
... ... ... ... ... ... ... ... ... ... ... ... ... ... ... ... ... ... ... ... ... ...
... ... ... ... ... ... ... ... ... ... ... ... ... ..G ... ... ... ... ... ... ... ..C
... ... ... ... ... ... ... ... ... ... ... ... ... ... ... ... ... ... ... ... ... ...
... ... ... ... ... ... ... ... ... ... ... ... ... ... ... ... ... ... ... ... ... ...
... ... ... ... ... ... ... ... ... ... ...
```

---

**3. Tree file "cd166.tree"**

```
((((human, chimpanzee), gorilla), orangutan), gibbon, rhesus);
```

**4. Tree file "cd166-human.tree"**

```
((((human #1, chimpanzee), gorilla), orangutan), gibbon, rhesus);
```

---

**5. Control file for "M0"**

```
      seqfile = cd166.phy
     treefile = cd166.tree
      outfile = cd166-M0-one-ratio.mlc

        noisy = 9  * 0,1,2,3,9: how much rubbish on the screen
      verbose = 0  * 0: concise; 1: detailed, 2: too much
      runmode = 0  * 0: user tree;  1: semi-automatic;  2: automatic
                   * 3: StepwiseAddition; (4,5):PerturbationNNI; -2: pairwise

      seqtype = 1  * 1:codons; 2:AAs; 3:codons-->AAs
    CodonFreq = 2  * 0:1/61 each, 1:F1X4, 2:F3X4, 3:codon table
        clock = 0  * 0:no clock, 1:clock; 2:local clock; 3:CombinedAnalysis
        model = 0
                   * models for codons:
                       * 0:one, 1:b, 2:2 or more dN/dS ratios for branches

      NSsites = 0  * 0:one w; 1:neutral; 2:selection; 3:discrete; 4:freqs;
                   * 5:gamma; 6:2gamma; 7:beta; 8:beta&w 9:betaγ
                   * 10:betaγ+1; 11:beta&normal>1; 12:0&2normal>1;
                   * 13:3normal>0
        icode = 0  * 0:universal code; 1:mammalian mt; 2-10:see below

    fix_kappa = 0  * 1: kappa fixed, 0: kappa to be estimated
        kappa = 2  * initial or fixed kappa
    fix_omega = 0  * 1: omega or omega_1 fixed, 0: estimate 
        omega = 1  * initial or fixed omega, for codons or codon-based AAs

    fix_alpha = 1  * 0: estimate gamma shape parameter; 1: fix it at alpha
        alpha = .0 * initial or fixed alpha, 0:infinity (constant rate)
       Malpha = 0  * different alphas for genes
        ncatG = 4  * # of categories in dG of NSsites models

        getSE = 0  * 0: don't want them, 1: want S.E.s of estimates
 RateAncestor = 0  * (0,1,2): rates (alpha>0) or ancestral states (1 or 2)
       method = 0  * 0: simultaneous; 1: one branch at a time
```

---

**6. Control file for "Free ratio"**

```
      seqfile = cd166.phy
     treefile = cd166.tree
      outfile = cd166-free-ratio.mlc

        noisy = 9  * 0,1,2,3,9: how much rubbish on the screen
      verbose = 0  * 0: concise; 1: detailed, 2: too much
      runmode = 0  * 0: user tree;  1: semi-automatic;  2: automatic
                   * 3: StepwiseAddition; (4,5):PerturbationNNI; -2: pairwise

      seqtype = 1  * 1:codons; 2:AAs; 3:codons-->AAs
    CodonFreq = 2  * 0:1/61 each, 1:F1X4, 2:F3X4, 3:codon table
        clock = 0  * 0:no clock, 1:clock; 2:local clock; 3:CombinedAnalysis
        model = 1
                   * models for codons:
                       * 0:one, 1:b, 2:2 or more dN/dS ratios for branches

      NSsites = 0  * 0:one w; 1:neutral; 2:selection; 3:discrete; 4:freqs;
                   * 5:gamma; 6:2gamma; 7:beta; 8:beta&w 9:betaγ
                   * 10:betaγ+1; 11:beta&normal>1; 12:0&2normal>1;
                   * 13:3normal>0
        icode = 0  * 0:universal code; 1:mammalian mt; 2-10:see below

    fix_kappa = 0  * 1: kappa fixed, 0: kappa to be estimated
        kappa = 2  * initial or fixed kappa
    fix_omega = 0  * 1: omega or omega_1 fixed, 0: estimate 
        omega = 1  * initial or fixed omega, for codons or codon-based AAs

    fix_alpha = 1  * 0: estimate gamma shape parameter; 1: fix it at alpha
        alpha = .0 * initial or fixed alpha, 0:infinity (constant rate)
       Malpha = 0  * different alphas for genes
        ncatG = 4  * # of categories in dG of NSsites models

        getSE = 0  * 0: don't want them, 1: want S.E.s of estimates
 RateAncestor = 0  * (0,1,2): rates (alpha>0) or ancestral states (1 or 2)
       method = 0  * 0: simultaneous; 1: one branch at a time
```

---

**7. Control file for "Two ratio"**

```
      seqfile = cd166.phy
     treefile = cd166-human.tree
      outfile = cd166-two-ratio.mlc

        noisy = 9  * 0,1,2,3,9: how much rubbish on the screen
      verbose = 0  * 0: concise; 1: detailed, 2: too much
      runmode = 0  * 0: user tree;  1: semi-automatic;  2: automatic
                   * 3: StepwiseAddition; (4,5):PerturbationNNI; -2: pairwise

      seqtype = 1  * 1:codons; 2:AAs; 3:codons-->AAs
    CodonFreq = 2  * 0:1/61 each, 1:F1X4, 2:F3X4, 3:codon table
        clock = 0  * 0:no clock, 1:clock; 2:local clock; 3:CombinedAnalysis
        model = 2
                   * models for codons:
                       * 0:one, 1:b, 2:2 or more dN/dS ratios for branches

      NSsites = 0  * 0:one w; 1:neutral; 2:selection; 3:discrete; 4:freqs;
                   * 5:gamma; 6:2gamma; 7:beta; 8:beta&w 9:betaγ
                   * 10:betaγ+1; 11:beta&normal>1; 12:0&2normal>1;
                   * 13:3normal>0
        icode = 0  * 0:universal code; 1:mammalian mt; 2-10:see below

    fix_kappa = 0  * 1: kappa fixed, 0: kappa to be estimated
        kappa = 2  * initial or fixed kappa
    fix_omega = 0  * 1: omega or omega_1 fixed, 0: estimate 
        omega = 1  * initial or fixed omega, for codons or codon-based AAs

    fix_alpha = 1  * 0: estimate gamma shape parameter; 1: fix it at alpha
        alpha = .0 * initial or fixed alpha, 0:infinity (constant rate)
       Malpha = 0  * different alphas for genes
        ncatG = 4  * # of categories in dG of NSsites models

        getSE = 0  * 0: don't want them, 1: want S.E.s of estimates
 RateAncestor = 0  * (0,1,2): rates (alpha>0) or ancestral states (1 or 2)
       method = 0  * 0: simultaneous; 1: one branch at a time
```

---

**8. Control file for "Model A"**

```
      seqfile = cd166.phy
     treefile = cd166-human.tree
      outfile = cd166-model-A.mlc

        noisy = 9  * 0,1,2,3,9: how much rubbish on the screen
      verbose = 0  * 0: concise; 1: detailed, 2: too much
      runmode = 0  * 0: user tree;  1: semi-automatic;  2: automatic
                   * 3: StepwiseAddition; (4,5):PerturbationNNI; -2: pairwise

      seqtype = 1  * 1:codons; 2:AAs; 3:codons-->AAs
    CodonFreq = 2  * 0:1/61 each, 1:F1X4, 2:F3X4, 3:codon table
        clock = 0  * 0:no clock, 1:clock; 2:local clock; 3:CombinedAnalysis
        model = 2
                   * models for codons:
                       * 0:one, 1:b, 2:2 or more dN/dS ratios for branches

      NSsites = 2  * 0:one w; 1:neutral; 2:selection; 3:discrete; 4:freqs;
                   * 5:gamma; 6:2gamma; 7:beta; 8:beta&w 9:betaγ
                   * 10:betaγ+1; 11:beta&normal>1; 12:0&2normal>1;
                   * 13:3normal>0
        icode = 0  * 0:universal code; 1:mammalian mt; 2-10:see below

    fix_kappa = 0  * 1: kappa fixed, 0: kappa to be estimated
        kappa = 2  * initial or fixed kappa
    fix_omega = 0  * 1: omega or omega_1 fixed, 0: estimate 
        omega = 1  * initial or fixed omega, for codons or codon-based AAs

    fix_alpha = 1  * 0: estimate gamma shape parameter; 1: fix it at alpha
        alpha = .0 * initial or fixed alpha, 0:infinity (constant rate)
       Malpha = 0  * different alphas for genes
        ncatG = 4  * # of categories in dG of NSsites models

        getSE = 0  * 0: don't want them, 1: want S.E.s of estimates
 RateAncestor = 0  * (0,1,2): rates (alpha>0) or ancestral states (1 or 2)
       method = 0  * 0: simultaneous; 1: one branch at a time
```

---

**9. Control file for "Null model A"**

```
      seqfile = cd166.phy
     treefile = cd166-human.tree
      outfile = cd166-null-model-A.mlc

        noisy = 9  * 0,1,2,3,9: how much rubbish on the screen
      verbose = 0  * 0: concise; 1: detailed, 2: too much
      runmode = 0  * 0: user tree;  1: semi-automatic;  2: automatic
                   * 3: StepwiseAddition; (4,5):PerturbationNNI; -2: pairwise

      seqtype = 1  * 1:codons; 2:AAs; 3:codons-->AAs
    CodonFreq = 2  * 0:1/61 each, 1:F1X4, 2:F3X4, 3:codon table
        clock = 0  * 0:no clock, 1:clock; 2:local clock; 3:CombinedAnalysis
        model = 2
                   * models for codons:
                       * 0:one, 1:b, 2:2 or more dN/dS ratios for branches

      NSsites = 2  * 0:one w; 1:neutral; 2:selection; 3:discrete; 4:freqs;
                   * 5:gamma; 6:2gamma; 7:beta; 8:beta&w 9:betaγ
                   * 10:betaγ+1; 11:beta&normal>1; 12:0&2normal>1;
                   * 13:3normal>0
        icode = 0  * 0:universal code; 1:mammalian mt; 2-10:see below

    fix_kappa = 0  * 1: kappa fixed, 0: kappa to be estimated
        kappa = 2  * initial or fixed kappa
    fix_omega = 1  * 1: omega or omega_1 fixed, 0: estimate 
        omega = 1  * initial or fixed omega, for codons or codon-based AAs

    fix_alpha = 1  * 0: estimate gamma shape parameter; 1: fix it at alpha
        alpha = .0 * initial or fixed alpha, 0:infinity (constant rate)
       Malpha = 0  * different alphas for genes
        ncatG = 4  * # of categories in dG of NSsites models

        getSE = 0  * 0: don't want them, 1: want S.E.s of estimates
 RateAncestor = 0  * (0,1,2): rates (alpha>0) or ancestral states (1 or 2)
       method = 0  * 0: simultaneous; 1: one branch at a time
```

---

**10. Main result file for "M0"**

```
CODONML (in paml version 4.8a, July 2014)  cd166.phy
Model: One dN/dS ratio for branches, 
Codon frequency model: F3x4
ns =   6  ls = 583

Codon usage in sequences
--------------------------------------------------------------------------------------------------------------------------------------
Phe TTT  11  11  11  11  11  11 | Ser TCT  11  10  10  10  11  11 | Tyr TAT  14  14  14  13  14  13 | Cys TGT   1   1   1   1   2   1
    TTC   5   5   5   5   5   4 |     TCC  10  11  11  12  11  11 |     TAC  11  11  11  12  11  12 |     TGC  11  11  11  11  10  11
Leu TTA   5   5   5   5   5   5 |     TCA  10  10  10  10  10  10 | *** TAA   0   0   0   0   0   0 | *** TGA   0   0   0   0   0   0
    TTG   9   8   9   9   9   9 |     TCG   1   1   1   1   0   0 |     TAG   0   0   0   0   0   0 | Trp TGG   6   6   6   6   6   6
--------------------------------------------------------------------------------------------------------------------------------------
Leu CTT   8   8   8   8   8   8 | Pro CCT   9  10   9  10   9  10 | His CAT   5   5   5   5   5   4 | Arg CGT   1   1   1   1   1   1
    CTC  10  10  10   9  11  12 |     CCC   6   6   6   5   6   6 |     CAC   3   3   3   3   3   4 |     CGC   1   1   1   1   1   1
    CTA   7   7   7   8   7   6 |     CCA  16  15  16  16  17  15 | Gln CAA   7   7   7   7   7   7 |     CGA   2   2   2   2   2   2
    CTG   9  10   9   9   8   9 |     CCG   1   1   1   1   0   1 |     CAG  12  12  12  12  11  12 |     CGG   0   0   0   0   1   0
--------------------------------------------------------------------------------------------------------------------------------------
Ile ATT  17  17  16  16  15  14 | Thr ACT  16  14  16  15  14  16 | Asn AAT  16  16  16  15  15  15 | Ser AGT  12  12  12  12  12  11
    ATC  12  12  13  12  14  13 |     ACC   6   7   6   7   7   7 |     AAC  15  14  14  15  15  14 |     AGC   6   7   7   7   7   8
    ATA  13  13  12  12  12  10 |     ACA  21  22  22  22  22  22 | Lys AAA  26  27  26  26  27  27 | Arg AGA   8   8   9   7   7   7
Met ATG  11  11  12  12  12  12 |     ACG   1   1   0   0   0   0 |     AAG  20  19  20  20  19  19 |     AGG   7   7   6   8   8   8
--------------------------------------------------------------------------------------------------------------------------------------
Val GTT   6   6   6   5   6   8 | Ala GCT   9   8   8   8   9   8 | Asp GAT  15  15  14  15  16  16 | Gly GGT   6   6   6   6   6   7
    GTC   8   8   8  10   8  10 |     GCC   8   9   9   8   9   9 |     GAC  13  13  14  13  12  12 |     GGC   9   9   9   9   9   8
    GTA  11  11  11  11  11  12 |     GCA  11  11  11  11  11  11 | Glu GAA  30  30  30  30  30  27 |     GGA  14  14  14  14  13  16
    GTG  17  17  17  17  17  16 |     GCG   1   1   1   1   1   1 |     GAG  13  13  13  13  13  16 |     GGG   3   3   3   3   4   1
--------------------------------------------------------------------------------------------------------------------------------------

Codon position x base (3x4) table for each sequence.

#1: human          
position  1:    T:0.18010    C:0.16638    A:0.35506    G:0.29846
position  2:    T:0.27273    C:0.23499    A:0.34305    G:0.14923
position  3:    T:0.26930    C:0.22985    A:0.31046    G:0.19039
Average         T:0.24071    C:0.21041    A:0.33619    G:0.21269

#2: chimpanzee     
position  1:    T:0.17839    C:0.16810    A:0.35506    G:0.29846
position  2:    T:0.27273    C:0.23499    A:0.34134    G:0.15094
position  3:    T:0.26415    C:0.23499    A:0.31218    G:0.18868
Average         T:0.23842    C:0.21269    A:0.33619    G:0.21269

#3: gorilla        
position  1:    T:0.18010    C:0.16638    A:0.35506    G:0.29846
position  2:    T:0.27273    C:0.23499    A:0.34134    G:0.15094
position  3:    T:0.26244    C:0.23671    A:0.31218    G:0.18868
Average         T:0.23842    C:0.21269    A:0.33619    G:0.21269

#4: orangutan      
position  1:    T:0.18182    C:0.16638    A:0.35334    G:0.29846
position  2:    T:0.27273    C:0.23499    A:0.34134    G:0.15094
position  3:    T:0.25901    C:0.23842    A:0.31046    G:0.19211
Average         T:0.23785    C:0.21326    A:0.33505    G:0.21384

#5: gibbon         
position  1:    T:0.18010    C:0.16638    A:0.35334    G:0.30017
position  2:    T:0.27273    C:0.23499    A:0.33962    G:0.15266
position  3:    T:0.26415    C:0.23842    A:0.31046    G:0.18696
Average         T:0.23899    C:0.21326    A:0.33448    G:0.21326

#6: rhesus         
position  1:    T:0.17839    C:0.16810    A:0.34820    G:0.30532
position  2:    T:0.27273    C:0.23671    A:0.33962    G:0.15094
position  3:    T:0.26415    C:0.24357    A:0.30360    G:0.18868
Average         T:0.23842    C:0.21612    A:0.33047    G:0.21498

Sums of codon usage counts
------------------------------------------------------------------------------
Phe F TTT      66 | Ser S TCT      63 | Tyr Y TAT      82 | Cys C TGT       7
      TTC      29 |       TCC      66 |       TAC      68 |       TGC      65
Leu L TTA      30 |       TCA      60 | *** * TAA       0 | *** * TGA       0
      TTG      53 |       TCG       4 |       TAG       0 | Trp W TGG      36
------------------------------------------------------------------------------
Leu L CTT      48 | Pro P CCT      57 | His H CAT      29 | Arg R CGT       6
      CTC      62 |       CCC      35 |       CAC      19 |       CGC       6
      CTA      42 |       CCA      95 | Gln Q CAA      42 |       CGA      12
      CTG      54 |       CCG       5 |       CAG      71 |       CGG       1
------------------------------------------------------------------------------
Ile I ATT      95 | Thr T ACT      91 | Asn N AAT      93 | Ser S AGT      71
      ATC      76 |       ACC      40 |       AAC      87 |       AGC      42
      ATA      72 |       ACA     131 | Lys K AAA     159 | Arg R AGA      46
Met M ATG      70 |       ACG       2 |       AAG     117 |       AGG      44
------------------------------------------------------------------------------
Val V GTT      37 | Ala A GCT      50 | Asp D GAT      91 | Gly G GGT      37
      GTC      52 |       GCC      52 |       GAC      77 |       GGC      53
      GTA      67 |       GCA      66 | Glu E GAA     177 |       GGA      85
      GTG     101 |       GCG       6 |       GAG      81 |       GGG      17
------------------------------------------------------------------------------


Codon position x base (3x4) table, overall

position  1:    T:0.17982    C:0.16695    A:0.35334    G:0.29989
position  2:    T:0.27273    C:0.23528    A:0.34105    G:0.15094
position  3:    T:0.26387    C:0.23699    A:0.30989    G:0.18925
Average         T:0.23880    C:0.21307    A:0.33476    G:0.21336


Nei & Gojobori 1986. dN/dS (dN, dS)
(Note: This matrix is not used in later ML. analysis.
Use runmode = -2 for ML pairwise comparison.)

human               
chimpanzee           0.0268 (0.0007 0.0278)
gorilla              0.0739 (0.0015 0.0201) 0.0328 (0.0007 0.0227)
orangutan            0.1072 (0.0030 0.0278) 0.0629 (0.0022 0.0355) 0.0656 (0.0015 0.0227)
gibbon               0.0780 (0.0030 0.0382) 0.0548 (0.0022 0.0408) 0.0451 (0.0015 0.0330) 0.0837 (0.0030 0.0356)
rhesus               0.0888 (0.0060 0.0673) 0.0747 (0.0052 0.0699) 0.0722 (0.0045 0.0619) 0.0925 (0.0060 0.0646) 0.1008 (0.0060 0.0592)


TREE #  1:  ((((1, 2), 3), 4), 5, 6);   MP score: 59
lnL(ntime:  9  np: 11):  -2713.893402      +0.000000
   7..8     8..9     9..10   10..1    10..2     9..3     8..4     7..5     7..6  
 0.004965 0.002274 0.003496 0.009384 0.011876 0.004757 0.012407 0.014523 0.041830 5.049256 0.099135

Note: Branch length is defined as number of nucleotide substitutions per codon (not per neucleotide site).

tree length =   0.10551

((((1: 0.009384, 2: 0.011876): 0.003496, 3: 0.004757): 0.002274, 4: 0.012407): 0.004965, 5: 0.014523, 6: 0.041830);

((((human: 0.009384, chimpanzee: 0.011876): 0.003496, gorilla: 0.004757): 0.002274, orangutan: 0.012407): 0.004965, gibbon: 0.014523, rhesus: 0.041830);

Detailed output identifying parameters

kappa (ts/tv) =  5.04926

omega (dN/dS) =  0.09913

dN & dS for each branch

 branch          t       N       S   dN/dS      dN      dS  N*dN  S*dS

   7..8      0.005  1245.4   503.6  0.0991  0.0005  0.0046   0.6   2.3
   8..9      0.002  1245.4   503.6  0.0991  0.0002  0.0021   0.3   1.1
   9..10     0.003  1245.4   503.6  0.0991  0.0003  0.0032   0.4   1.6
  10..1      0.009  1245.4   503.6  0.0991  0.0009  0.0087   1.1   4.4
  10..2      0.012  1245.4   503.6  0.0991  0.0011  0.0110   1.4   5.6
   9..3      0.005  1245.4   503.6  0.0991  0.0004  0.0044   0.5   2.2
   8..4      0.012  1245.4   503.6  0.0991  0.0011  0.0115   1.4   5.8
   7..5      0.015  1245.4   503.6  0.0991  0.0013  0.0135   1.7   6.8
   7..6      0.042  1245.4   503.6  0.0991  0.0039  0.0389   4.8  19.6

tree length for dN:       0.0097
tree length for dS:       0.0981


Time used:  0:03
```

---

**11. Main result file for "Free ratio"**

```
CODONML (in paml version 4.8a, July 2014)  cd166.phy
Model: free dN/dS Ratios for branches for branches, 
Codon frequency model: F3x4
ns =   6  ls = 583

Codon usage in sequences
--------------------------------------------------------------------------------------------------------------------------------------
Phe TTT  11  11  11  11  11  11 | Ser TCT  11  10  10  10  11  11 | Tyr TAT  14  14  14  13  14  13 | Cys TGT   1   1   1   1   2   1
    TTC   5   5   5   5   5   4 |     TCC  10  11  11  12  11  11 |     TAC  11  11  11  12  11  12 |     TGC  11  11  11  11  10  11
Leu TTA   5   5   5   5   5   5 |     TCA  10  10  10  10  10  10 | *** TAA   0   0   0   0   0   0 | *** TGA   0   0   0   0   0   0
    TTG   9   8   9   9   9   9 |     TCG   1   1   1   1   0   0 |     TAG   0   0   0   0   0   0 | Trp TGG   6   6   6   6   6   6
--------------------------------------------------------------------------------------------------------------------------------------
Leu CTT   8   8   8   8   8   8 | Pro CCT   9  10   9  10   9  10 | His CAT   5   5   5   5   5   4 | Arg CGT   1   1   1   1   1   1
    CTC  10  10  10   9  11  12 |     CCC   6   6   6   5   6   6 |     CAC   3   3   3   3   3   4 |     CGC   1   1   1   1   1   1
    CTA   7   7   7   8   7   6 |     CCA  16  15  16  16  17  15 | Gln CAA   7   7   7   7   7   7 |     CGA   2   2   2   2   2   2
    CTG   9  10   9   9   8   9 |     CCG   1   1   1   1   0   1 |     CAG  12  12  12  12  11  12 |     CGG   0   0   0   0   1   0
--------------------------------------------------------------------------------------------------------------------------------------
Ile ATT  17  17  16  16  15  14 | Thr ACT  16  14  16  15  14  16 | Asn AAT  16  16  16  15  15  15 | Ser AGT  12  12  12  12  12  11
    ATC  12  12  13  12  14  13 |     ACC   6   7   6   7   7   7 |     AAC  15  14  14  15  15  14 |     AGC   6   7   7   7   7   8
    ATA  13  13  12  12  12  10 |     ACA  21  22  22  22  22  22 | Lys AAA  26  27  26  26  27  27 | Arg AGA   8   8   9   7   7   7
Met ATG  11  11  12  12  12  12 |     ACG   1   1   0   0   0   0 |     AAG  20  19  20  20  19  19 |     AGG   7   7   6   8   8   8
--------------------------------------------------------------------------------------------------------------------------------------
Val GTT   6   6   6   5   6   8 | Ala GCT   9   8   8   8   9   8 | Asp GAT  15  15  14  15  16  16 | Gly GGT   6   6   6   6   6   7
    GTC   8   8   8  10   8  10 |     GCC   8   9   9   8   9   9 |     GAC  13  13  14  13  12  12 |     GGC   9   9   9   9   9   8
    GTA  11  11  11  11  11  12 |     GCA  11  11  11  11  11  11 | Glu GAA  30  30  30  30  30  27 |     GGA  14  14  14  14  13  16
    GTG  17  17  17  17  17  16 |     GCG   1   1   1   1   1   1 |     GAG  13  13  13  13  13  16 |     GGG   3   3   3   3   4   1
--------------------------------------------------------------------------------------------------------------------------------------

Codon position x base (3x4) table for each sequence.

#1: human          
position  1:    T:0.18010    C:0.16638    A:0.35506    G:0.29846
position  2:    T:0.27273    C:0.23499    A:0.34305    G:0.14923
position  3:    T:0.26930    C:0.22985    A:0.31046    G:0.19039
Average         T:0.24071    C:0.21041    A:0.33619    G:0.21269

#2: chimpanzee     
position  1:    T:0.17839    C:0.16810    A:0.35506    G:0.29846
position  2:    T:0.27273    C:0.23499    A:0.34134    G:0.15094
position  3:    T:0.26415    C:0.23499    A:0.31218    G:0.18868
Average         T:0.23842    C:0.21269    A:0.33619    G:0.21269

#3: gorilla        
position  1:    T:0.18010    C:0.16638    A:0.35506    G:0.29846
position  2:    T:0.27273    C:0.23499    A:0.34134    G:0.15094
position  3:    T:0.26244    C:0.23671    A:0.31218    G:0.18868
Average         T:0.23842    C:0.21269    A:0.33619    G:0.21269

#4: orangutan      
position  1:    T:0.18182    C:0.16638    A:0.35334    G:0.29846
position  2:    T:0.27273    C:0.23499    A:0.34134    G:0.15094
position  3:    T:0.25901    C:0.23842    A:0.31046    G:0.19211
Average         T:0.23785    C:0.21326    A:0.33505    G:0.21384

#5: gibbon         
position  1:    T:0.18010    C:0.16638    A:0.35334    G:0.30017
position  2:    T:0.27273    C:0.23499    A:0.33962    G:0.15266
position  3:    T:0.26415    C:0.23842    A:0.31046    G:0.18696
Average         T:0.23899    C:0.21326    A:0.33448    G:0.21326

#6: rhesus         
position  1:    T:0.17839    C:0.16810    A:0.34820    G:0.30532
position  2:    T:0.27273    C:0.23671    A:0.33962    G:0.15094
position  3:    T:0.26415    C:0.24357    A:0.30360    G:0.18868
Average         T:0.23842    C:0.21612    A:0.33047    G:0.21498

Sums of codon usage counts
------------------------------------------------------------------------------
Phe F TTT      66 | Ser S TCT      63 | Tyr Y TAT      82 | Cys C TGT       7
      TTC      29 |       TCC      66 |       TAC      68 |       TGC      65
Leu L TTA      30 |       TCA      60 | *** * TAA       0 | *** * TGA       0
      TTG      53 |       TCG       4 |       TAG       0 | Trp W TGG      36
------------------------------------------------------------------------------
Leu L CTT      48 | Pro P CCT      57 | His H CAT      29 | Arg R CGT       6
      CTC      62 |       CCC      35 |       CAC      19 |       CGC       6
      CTA      42 |       CCA      95 | Gln Q CAA      42 |       CGA      12
      CTG      54 |       CCG       5 |       CAG      71 |       CGG       1
------------------------------------------------------------------------------
Ile I ATT      95 | Thr T ACT      91 | Asn N AAT      93 | Ser S AGT      71
      ATC      76 |       ACC      40 |       AAC      87 |       AGC      42
      ATA      72 |       ACA     131 | Lys K AAA     159 | Arg R AGA      46
Met M ATG      70 |       ACG       2 |       AAG     117 |       AGG      44
------------------------------------------------------------------------------
Val V GTT      37 | Ala A GCT      50 | Asp D GAT      91 | Gly G GGT      37
      GTC      52 |       GCC      52 |       GAC      77 |       GGC      53
      GTA      67 |       GCA      66 | Glu E GAA     177 |       GGA      85
      GTG     101 |       GCG       6 |       GAG      81 |       GGG      17
------------------------------------------------------------------------------


Codon position x base (3x4) table, overall

position  1:    T:0.17982    C:0.16695    A:0.35334    G:0.29989
position  2:    T:0.27273    C:0.23528    A:0.34105    G:0.15094
position  3:    T:0.26387    C:0.23699    A:0.30989    G:0.18925
Average         T:0.23880    C:0.21307    A:0.33476    G:0.21336


Nei & Gojobori 1986. dN/dS (dN, dS)
(Note: This matrix is not used in later ML. analysis.
Use runmode = -2 for ML pairwise comparison.)

human               
chimpanzee           0.0268 (0.0007 0.0278)
gorilla              0.0739 (0.0015 0.0201) 0.0328 (0.0007 0.0227)
orangutan            0.1072 (0.0030 0.0278) 0.0629 (0.0022 0.0355) 0.0656 (0.0015 0.0227)
gibbon               0.0780 (0.0030 0.0382) 0.0548 (0.0022 0.0408) 0.0451 (0.0015 0.0330) 0.0837 (0.0030 0.0356)
rhesus               0.0888 (0.0060 0.0673) 0.0747 (0.0052 0.0699) 0.0722 (0.0045 0.0619) 0.0925 (0.0060 0.0646) 0.1008 (0.0060 0.0592)


TREE #  1:  ((((1, 2), 3), 4), 5, 6);   MP score: 59
lnL(ntime:  9  np: 19):  -2709.995088      +0.000000
   7..8     8..9     9..10   10..1    10..2     9..3     8..4     7..5     7..6  
 0.005149 0.002194 0.003397 0.009329 0.012129 0.004887 0.012361 0.014359 0.041549 5.044678 0.000100 0.000100 0.417944 0.091813 0.000100 0.000100 0.157262 0.128103 0.135245

Note: Branch length is defined as number of nucleotide substitutions per codon (not per neucleotide site).

tree length =   0.10535

((((1: 0.009329, 2: 0.012129): 0.003397, 3: 0.004887): 0.002194, 4: 0.012361): 0.005149, 5: 0.014359, 6: 0.041549);

((((human: 0.009329, chimpanzee: 0.012129): 0.003397, gorilla: 0.004887): 0.002194, orangutan: 0.012361): 0.005149, gibbon: 0.014359, rhesus: 0.041549);

Detailed output identifying parameters

kappa (ts/tv) =  5.04468

w (dN/dS) for branches:  0.00010 0.00010 0.41794 0.09181 0.00010 0.00010 0.15726 0.12810 0.13524

dN & dS for each branch

 branch          t       N       S   dN/dS      dN      dS  N*dN  S*dS

   7..8      0.005  1245.4   503.6  0.0001  0.0000  0.0060   0.0   3.0
   8..9      0.002  1245.4   503.6  0.0001  0.0000  0.0025   0.0   1.3
   9..10     0.003  1245.4   503.6  0.4179  0.0008  0.0019   1.0   1.0
  10..1      0.009  1245.4   503.6  0.0918  0.0008  0.0088   1.0   4.4
  10..2      0.012  1245.4   503.6  0.0001  0.0000  0.0140   0.0   7.1
   9..3      0.005  1245.4   503.6  0.0001  0.0000  0.0057   0.0   2.8
   8..4      0.012  1245.4   503.6  0.1573  0.0016  0.0103   2.0   5.2
   7..5      0.014  1245.4   503.6  0.1281  0.0016  0.0126   2.0   6.4
   7..6      0.042  1245.4   503.6  0.1352  0.0049  0.0360   6.1  18.2

tree length for dN:       0.0097
tree length for dS:       0.0979

dS tree:
((((human: 0.008802, chimpanzee: 0.014039): 0.001934, gorilla: 0.005656): 0.002539, orangutan: 0.010304): 0.005959, gibbon: 0.012624, rhesus: 0.036046);
dN tree:
((((human: 0.000808, chimpanzee: 0.000001): 0.000808, gorilla: 0.000001): 0.000000, orangutan: 0.001620): 0.000001, gibbon: 0.001617, rhesus: 0.004875);

w ratios as labels for TreeView:
((((human #0.0918 , chimpanzee #0.0001 ) #0.4179 , gorilla #0.0001 ) #0.0001 , orangutan #0.1573 ) #0.0001 , gibbon #0.1281 , rhesus #0.1352 );


Time used:  0:23
```

---

**12. Main result file for "Two ratio"**

```
CODONML (in paml version 4.8a, July 2014)  cd166.phy
Model: several dN/dS ratios for branches for branches, 
Codon frequency model: F3x4
ns =   6  ls = 583

Codon usage in sequences
--------------------------------------------------------------------------------------------------------------------------------------
Phe TTT  11  11  11  11  11  11 | Ser TCT  11  10  10  10  11  11 | Tyr TAT  14  14  14  13  14  13 | Cys TGT   1   1   1   1   2   1
    TTC   5   5   5   5   5   4 |     TCC  10  11  11  12  11  11 |     TAC  11  11  11  12  11  12 |     TGC  11  11  11  11  10  11
Leu TTA   5   5   5   5   5   5 |     TCA  10  10  10  10  10  10 | *** TAA   0   0   0   0   0   0 | *** TGA   0   0   0   0   0   0
    TTG   9   8   9   9   9   9 |     TCG   1   1   1   1   0   0 |     TAG   0   0   0   0   0   0 | Trp TGG   6   6   6   6   6   6
--------------------------------------------------------------------------------------------------------------------------------------
Leu CTT   8   8   8   8   8   8 | Pro CCT   9  10   9  10   9  10 | His CAT   5   5   5   5   5   4 | Arg CGT   1   1   1   1   1   1
    CTC  10  10  10   9  11  12 |     CCC   6   6   6   5   6   6 |     CAC   3   3   3   3   3   4 |     CGC   1   1   1   1   1   1
    CTA   7   7   7   8   7   6 |     CCA  16  15  16  16  17  15 | Gln CAA   7   7   7   7   7   7 |     CGA   2   2   2   2   2   2
    CTG   9  10   9   9   8   9 |     CCG   1   1   1   1   0   1 |     CAG  12  12  12  12  11  12 |     CGG   0   0   0   0   1   0
--------------------------------------------------------------------------------------------------------------------------------------
Ile ATT  17  17  16  16  15  14 | Thr ACT  16  14  16  15  14  16 | Asn AAT  16  16  16  15  15  15 | Ser AGT  12  12  12  12  12  11
    ATC  12  12  13  12  14  13 |     ACC   6   7   6   7   7   7 |     AAC  15  14  14  15  15  14 |     AGC   6   7   7   7   7   8
    ATA  13  13  12  12  12  10 |     ACA  21  22  22  22  22  22 | Lys AAA  26  27  26  26  27  27 | Arg AGA   8   8   9   7   7   7
Met ATG  11  11  12  12  12  12 |     ACG   1   1   0   0   0   0 |     AAG  20  19  20  20  19  19 |     AGG   7   7   6   8   8   8
--------------------------------------------------------------------------------------------------------------------------------------
Val GTT   6   6   6   5   6   8 | Ala GCT   9   8   8   8   9   8 | Asp GAT  15  15  14  15  16  16 | Gly GGT   6   6   6   6   6   7
    GTC   8   8   8  10   8  10 |     GCC   8   9   9   8   9   9 |     GAC  13  13  14  13  12  12 |     GGC   9   9   9   9   9   8
    GTA  11  11  11  11  11  12 |     GCA  11  11  11  11  11  11 | Glu GAA  30  30  30  30  30  27 |     GGA  14  14  14  14  13  16
    GTG  17  17  17  17  17  16 |     GCG   1   1   1   1   1   1 |     GAG  13  13  13  13  13  16 |     GGG   3   3   3   3   4   1
--------------------------------------------------------------------------------------------------------------------------------------

Codon position x base (3x4) table for each sequence.

#1: human          
position  1:    T:0.18010    C:0.16638    A:0.35506    G:0.29846
position  2:    T:0.27273    C:0.23499    A:0.34305    G:0.14923
position  3:    T:0.26930    C:0.22985    A:0.31046    G:0.19039
Average         T:0.24071    C:0.21041    A:0.33619    G:0.21269

#2: chimpanzee     
position  1:    T:0.17839    C:0.16810    A:0.35506    G:0.29846
position  2:    T:0.27273    C:0.23499    A:0.34134    G:0.15094
position  3:    T:0.26415    C:0.23499    A:0.31218    G:0.18868
Average         T:0.23842    C:0.21269    A:0.33619    G:0.21269

#3: gorilla        
position  1:    T:0.18010    C:0.16638    A:0.35506    G:0.29846
position  2:    T:0.27273    C:0.23499    A:0.34134    G:0.15094
position  3:    T:0.26244    C:0.23671    A:0.31218    G:0.18868
Average         T:0.23842    C:0.21269    A:0.33619    G:0.21269

#4: orangutan      
position  1:    T:0.18182    C:0.16638    A:0.35334    G:0.29846
position  2:    T:0.27273    C:0.23499    A:0.34134    G:0.15094
position  3:    T:0.25901    C:0.23842    A:0.31046    G:0.19211
Average         T:0.23785    C:0.21326    A:0.33505    G:0.21384

#5: gibbon         
position  1:    T:0.18010    C:0.16638    A:0.35334    G:0.30017
position  2:    T:0.27273    C:0.23499    A:0.33962    G:0.15266
position  3:    T:0.26415    C:0.23842    A:0.31046    G:0.18696
Average         T:0.23899    C:0.21326    A:0.33448    G:0.21326

#6: rhesus         
position  1:    T:0.17839    C:0.16810    A:0.34820    G:0.30532
position  2:    T:0.27273    C:0.23671    A:0.33962    G:0.15094
position  3:    T:0.26415    C:0.24357    A:0.30360    G:0.18868
Average         T:0.23842    C:0.21612    A:0.33047    G:0.21498

Sums of codon usage counts
------------------------------------------------------------------------------
Phe F TTT      66 | Ser S TCT      63 | Tyr Y TAT      82 | Cys C TGT       7
      TTC      29 |       TCC      66 |       TAC      68 |       TGC      65
Leu L TTA      30 |       TCA      60 | *** * TAA       0 | *** * TGA       0
      TTG      53 |       TCG       4 |       TAG       0 | Trp W TGG      36
------------------------------------------------------------------------------
Leu L CTT      48 | Pro P CCT      57 | His H CAT      29 | Arg R CGT       6
      CTC      62 |       CCC      35 |       CAC      19 |       CGC       6
      CTA      42 |       CCA      95 | Gln Q CAA      42 |       CGA      12
      CTG      54 |       CCG       5 |       CAG      71 |       CGG       1
------------------------------------------------------------------------------
Ile I ATT      95 | Thr T ACT      91 | Asn N AAT      93 | Ser S AGT      71
      ATC      76 |       ACC      40 |       AAC      87 |       AGC      42
      ATA      72 |       ACA     131 | Lys K AAA     159 | Arg R AGA      46
Met M ATG      70 |       ACG       2 |       AAG     117 |       AGG      44
------------------------------------------------------------------------------
Val V GTT      37 | Ala A GCT      50 | Asp D GAT      91 | Gly G GGT      37
      GTC      52 |       GCC      52 |       GAC      77 |       GGC      53
      GTA      67 |       GCA      66 | Glu E GAA     177 |       GGA      85
      GTG     101 |       GCG       6 |       GAG      81 |       GGG      17
------------------------------------------------------------------------------


Codon position x base (3x4) table, overall

position  1:    T:0.17982    C:0.16695    A:0.35334    G:0.29989
position  2:    T:0.27273    C:0.23528    A:0.34105    G:0.15094
position  3:    T:0.26387    C:0.23699    A:0.30989    G:0.18925
Average         T:0.23880    C:0.21307    A:0.33476    G:0.21336


Nei & Gojobori 1986. dN/dS (dN, dS)
(Note: This matrix is not used in later ML. analysis.
Use runmode = -2 for ML pairwise comparison.)

human               
chimpanzee           0.0268 (0.0007 0.0278)
gorilla              0.0739 (0.0015 0.0201) 0.0328 (0.0007 0.0227)
orangutan            0.1072 (0.0030 0.0278) 0.0629 (0.0022 0.0355) 0.0656 (0.0015 0.0227)
gibbon               0.0780 (0.0030 0.0382) 0.0548 (0.0022 0.0408) 0.0451 (0.0015 0.0330) 0.0837 (0.0030 0.0356)
rhesus               0.0888 (0.0060 0.0673) 0.0747 (0.0052 0.0699) 0.0722 (0.0045 0.0619) 0.0925 (0.0060 0.0646) 0.1008 (0.0060 0.0592)


TREE #  1:  ((((1, 2), 3), 4), 5, 6);   MP score: 59
lnL(ntime:  9  np: 12):  -2713.890425      +0.000000
   7..8     8..9     9..10   10..1    10..2     9..3     8..4     7..5     7..6  
 0.004966 0.002283 0.003495 0.009399 0.011865 0.004748 0.012407 0.014521 0.041826 5.048905 0.099914 0.091338

Note: Branch length is defined as number of nucleotide substitutions per codon (not per neucleotide site).

tree length =   0.10551

((((1: 0.009399, 2: 0.011865): 0.003495, 3: 0.004748): 0.002283, 4: 0.012407): 0.004966, 5: 0.014521, 6: 0.041826);

((((human: 0.009399, chimpanzee: 0.011865): 0.003495, gorilla: 0.004748): 0.002283, orangutan: 0.012407): 0.004966, gibbon: 0.014521, rhesus: 0.041826);

Detailed output identifying parameters

kappa (ts/tv) =  5.04891

w (dN/dS) for branches:  0.09991 0.09134

dN & dS for each branch

 branch          t       N       S   dN/dS      dN      dS  N*dN  S*dS

   7..8      0.005  1245.4   503.6  0.0999  0.0005  0.0046   0.6   2.3
   8..9      0.002  1245.4   503.6  0.0999  0.0002  0.0021   0.3   1.1
   9..10     0.003  1245.4   503.6  0.0999  0.0003  0.0032   0.4   1.6
  10..1      0.009  1245.4   503.6  0.0913  0.0008  0.0089   1.0   4.5
  10..2      0.012  1245.4   503.6  0.0999  0.0011  0.0110   1.4   5.5
   9..3      0.005  1245.4   503.6  0.0999  0.0004  0.0044   0.5   2.2
   8..4      0.012  1245.4   503.6  0.0999  0.0012  0.0115   1.4   5.8
   7..5      0.015  1245.4   503.6  0.0999  0.0013  0.0135   1.7   6.8
   7..6      0.042  1245.4   503.6  0.0999  0.0039  0.0388   4.8  19.6

tree length for dN:       0.0097
tree length for dS:       0.0981

dS tree:
((((human: 0.008875, chimpanzee: 0.011014): 0.003244, gorilla: 0.004407): 0.002119, orangutan: 0.011517): 0.004609, gibbon: 0.013480, rhesus: 0.038826);
dN tree:
((((human: 0.000811, chimpanzee: 0.001100): 0.000324, gorilla: 0.000440): 0.000212, orangutan: 0.001151): 0.000461, gibbon: 0.001347, rhesus: 0.003879);

w ratios as labels for TreeView:
((((human #0.0913 , chimpanzee #0.0999 ) #0.0999 , gorilla #0.0999 ) #0.0999 , orangutan #0.0999 ) #0.0999 , gibbon #0.0999 , rhesus #0.0999 );


Time used:  0:05
```

---

**13. Main result file for "Model A"**

```
CODONML (in paml version 4.8a, July 2014)  cd166.phy
Model: several dN/dS ratios for branches for branches, 
Codon frequency model: F3x4
Site-class models:  PositiveSelection
ns =   6  ls = 583

Codon usage in sequences
--------------------------------------------------------------------------------------------------------------------------------------
Phe TTT  11  11  11  11  11  11 | Ser TCT  11  10  10  10  11  11 | Tyr TAT  14  14  14  13  14  13 | Cys TGT   1   1   1   1   2   1
    TTC   5   5   5   5   5   4 |     TCC  10  11  11  12  11  11 |     TAC  11  11  11  12  11  12 |     TGC  11  11  11  11  10  11
Leu TTA   5   5   5   5   5   5 |     TCA  10  10  10  10  10  10 | *** TAA   0   0   0   0   0   0 | *** TGA   0   0   0   0   0   0
    TTG   9   8   9   9   9   9 |     TCG   1   1   1   1   0   0 |     TAG   0   0   0   0   0   0 | Trp TGG   6   6   6   6   6   6
--------------------------------------------------------------------------------------------------------------------------------------
Leu CTT   8   8   8   8   8   8 | Pro CCT   9  10   9  10   9  10 | His CAT   5   5   5   5   5   4 | Arg CGT   1   1   1   1   1   1
    CTC  10  10  10   9  11  12 |     CCC   6   6   6   5   6   6 |     CAC   3   3   3   3   3   4 |     CGC   1   1   1   1   1   1
    CTA   7   7   7   8   7   6 |     CCA  16  15  16  16  17  15 | Gln CAA   7   7   7   7   7   7 |     CGA   2   2   2   2   2   2
    CTG   9  10   9   9   8   9 |     CCG   1   1   1   1   0   1 |     CAG  12  12  12  12  11  12 |     CGG   0   0   0   0   1   0
--------------------------------------------------------------------------------------------------------------------------------------
Ile ATT  17  17  16  16  15  14 | Thr ACT  16  14  16  15  14  16 | Asn AAT  16  16  16  15  15  15 | Ser AGT  12  12  12  12  12  11
    ATC  12  12  13  12  14  13 |     ACC   6   7   6   7   7   7 |     AAC  15  14  14  15  15  14 |     AGC   6   7   7   7   7   8
    ATA  13  13  12  12  12  10 |     ACA  21  22  22  22  22  22 | Lys AAA  26  27  26  26  27  27 | Arg AGA   8   8   9   7   7   7
Met ATG  11  11  12  12  12  12 |     ACG   1   1   0   0   0   0 |     AAG  20  19  20  20  19  19 |     AGG   7   7   6   8   8   8
--------------------------------------------------------------------------------------------------------------------------------------
Val GTT   6   6   6   5   6   8 | Ala GCT   9   8   8   8   9   8 | Asp GAT  15  15  14  15  16  16 | Gly GGT   6   6   6   6   6   7
    GTC   8   8   8  10   8  10 |     GCC   8   9   9   8   9   9 |     GAC  13  13  14  13  12  12 |     GGC   9   9   9   9   9   8
    GTA  11  11  11  11  11  12 |     GCA  11  11  11  11  11  11 | Glu GAA  30  30  30  30  30  27 |     GGA  14  14  14  14  13  16
    GTG  17  17  17  17  17  16 |     GCG   1   1   1   1   1   1 |     GAG  13  13  13  13  13  16 |     GGG   3   3   3   3   4   1
--------------------------------------------------------------------------------------------------------------------------------------

Codon position x base (3x4) table for each sequence.

#1: human          
position  1:    T:0.18010    C:0.16638    A:0.35506    G:0.29846
position  2:    T:0.27273    C:0.23499    A:0.34305    G:0.14923
position  3:    T:0.26930    C:0.22985    A:0.31046    G:0.19039
Average         T:0.24071    C:0.21041    A:0.33619    G:0.21269

#2: chimpanzee     
position  1:    T:0.17839    C:0.16810    A:0.35506    G:0.29846
position  2:    T:0.27273    C:0.23499    A:0.34134    G:0.15094
position  3:    T:0.26415    C:0.23499    A:0.31218    G:0.18868
Average         T:0.23842    C:0.21269    A:0.33619    G:0.21269

#3: gorilla        
position  1:    T:0.18010    C:0.16638    A:0.35506    G:0.29846
position  2:    T:0.27273    C:0.23499    A:0.34134    G:0.15094
position  3:    T:0.26244    C:0.23671    A:0.31218    G:0.18868
Average         T:0.23842    C:0.21269    A:0.33619    G:0.21269

#4: orangutan      
position  1:    T:0.18182    C:0.16638    A:0.35334    G:0.29846
position  2:    T:0.27273    C:0.23499    A:0.34134    G:0.15094
position  3:    T:0.25901    C:0.23842    A:0.31046    G:0.19211
Average         T:0.23785    C:0.21326    A:0.33505    G:0.21384

#5: gibbon         
position  1:    T:0.18010    C:0.16638    A:0.35334    G:0.30017
position  2:    T:0.27273    C:0.23499    A:0.33962    G:0.15266
position  3:    T:0.26415    C:0.23842    A:0.31046    G:0.18696
Average         T:0.23899    C:0.21326    A:0.33448    G:0.21326

#6: rhesus         
position  1:    T:0.17839    C:0.16810    A:0.34820    G:0.30532
position  2:    T:0.27273    C:0.23671    A:0.33962    G:0.15094
position  3:    T:0.26415    C:0.24357    A:0.30360    G:0.18868
Average         T:0.23842    C:0.21612    A:0.33047    G:0.21498

Sums of codon usage counts
------------------------------------------------------------------------------
Phe F TTT      66 | Ser S TCT      63 | Tyr Y TAT      82 | Cys C TGT       7
      TTC      29 |       TCC      66 |       TAC      68 |       TGC      65
Leu L TTA      30 |       TCA      60 | *** * TAA       0 | *** * TGA       0
      TTG      53 |       TCG       4 |       TAG       0 | Trp W TGG      36
------------------------------------------------------------------------------
Leu L CTT      48 | Pro P CCT      57 | His H CAT      29 | Arg R CGT       6
      CTC      62 |       CCC      35 |       CAC      19 |       CGC       6
      CTA      42 |       CCA      95 | Gln Q CAA      42 |       CGA      12
      CTG      54 |       CCG       5 |       CAG      71 |       CGG       1
------------------------------------------------------------------------------
Ile I ATT      95 | Thr T ACT      91 | Asn N AAT      93 | Ser S AGT      71
      ATC      76 |       ACC      40 |       AAC      87 |       AGC      42
      ATA      72 |       ACA     131 | Lys K AAA     159 | Arg R AGA      46
Met M ATG      70 |       ACG       2 |       AAG     117 |       AGG      44
------------------------------------------------------------------------------
Val V GTT      37 | Ala A GCT      50 | Asp D GAT      91 | Gly G GGT      37
      GTC      52 |       GCC      52 |       GAC      77 |       GGC      53
      GTA      67 |       GCA      66 | Glu E GAA     177 |       GGA      85
      GTG     101 |       GCG       6 |       GAG      81 |       GGG      17
------------------------------------------------------------------------------


Codon position x base (3x4) table, overall

position  1:    T:0.17982    C:0.16695    A:0.35334    G:0.29989
position  2:    T:0.27273    C:0.23528    A:0.34105    G:0.15094
position  3:    T:0.26387    C:0.23699    A:0.30989    G:0.18925
Average         T:0.23880    C:0.21307    A:0.33476    G:0.21336


Nei & Gojobori 1986. dN/dS (dN, dS)
(Note: This matrix is not used in later ML. analysis.
Use runmode = -2 for ML pairwise comparison.)

human               
chimpanzee           0.0268 (0.0007 0.0278)
gorilla              0.0739 (0.0015 0.0201) 0.0328 (0.0007 0.0227)
orangutan            0.1072 (0.0030 0.0278) 0.0629 (0.0022 0.0355) 0.0656 (0.0015 0.0227)
gibbon               0.0780 (0.0030 0.0382) 0.0548 (0.0022 0.0408) 0.0451 (0.0015 0.0330) 0.0837 (0.0030 0.0356)
rhesus               0.0888 (0.0060 0.0673) 0.0747 (0.0052 0.0699) 0.0722 (0.0045 0.0619) 0.0925 (0.0060 0.0646) 0.1008 (0.0060 0.0592)


TREE #  1:  ((((1, 2), 3), 4), 5, 6);   MP score: 59
check convergence..
lnL(ntime:  9  np: 14):  -2713.893402      +0.000000
   7..8     8..9     9..10   10..1    10..2     9..3     8..4     7..5     7..6  
 0.004965 0.002274 0.003496 0.009384 0.011876 0.004757 0.012407 0.014522 0.041830 5.049053 1.000000 0.000000 0.099134 1.000000

Note: Branch length is defined as number of nucleotide substitutions per codon (not per neucleotide site).

tree length =   0.10551

((((1: 0.009384, 2: 0.011876): 0.003496, 3: 0.004757): 0.002274, 4: 0.012407): 0.004965, 5: 0.014522, 6: 0.041830);

((((human: 0.009384, chimpanzee: 0.011876): 0.003496, gorilla: 0.004757): 0.002274, orangutan: 0.012407): 0.004965, gibbon: 0.014522, rhesus: 0.041830);

Detailed output identifying parameters

kappa (ts/tv) =  5.04905


dN/dS (w) for site classes (K=4)

site class             0        1       2a       2b
proportion       1.00000  0.00000  0.00000  0.00000
background w     0.09913  1.00000  0.09913  1.00000
foreground w     0.09913  1.00000  1.00000  1.00000


Bayes Empirical Bayes (BEB) analysis (Yang, Wong & Nielsen 2005. Mol. Biol. Evol. 22:1107-1118)
Positive sites for foreground lineages Prob(w>1):
    91 N 0.613


The grid (see ternary graph for p0-p1)

w0:   0.050  0.150  0.250  0.350  0.450  0.550  0.650  0.750  0.850  0.950
w2:   1.500  2.500  3.500  4.500  5.500  6.500  7.500  8.500  9.500 10.500


Posterior on the grid

w0:   0.934  0.066  0.000  0.000  0.000  0.000  0.000  0.000  0.000  0.000
w2:   0.315  0.190  0.133  0.099  0.075  0.058  0.045  0.035  0.028  0.022

Posterior for p0-p1 (see the ternary graph)

 0.000
 0.000 0.000 0.000
 0.000 0.000 0.000 0.000 0.000
 0.000 0.000 0.000 0.000 0.000 0.000 0.000
 0.000 0.000 0.000 0.000 0.000 0.000 0.000 0.000 0.000
 0.000 0.000 0.000 0.000 0.000 0.000 0.000 0.000 0.000 0.000 0.000
 0.000 0.000 0.000 0.000 0.000 0.000 0.000 0.000 0.000 0.000 0.000 0.000 0.000
 0.000 0.000 0.000 0.000 0.000 0.000 0.000 0.000 0.000 0.000 0.000 0.000 0.000 0.000 0.000
 0.000 0.000 0.000 0.000 0.000 0.000 0.000 0.000 0.000 0.000 0.000 0.000 0.000 0.000 0.003 0.003 0.048
 0.000 0.000 0.000 0.000 0.000 0.000 0.000 0.000 0.000 0.000 0.001 0.001 0.006 0.007 0.022 0.033 0.084 0.205 0.586

sum of density on p0-p1 =   1.000000

Time used:  0:26
```

---

**14. Main result file for "Null model A"**

```
CODONML (in paml version 4.8a, July 2014)  cd166.phy
Model: several dN/dS ratios for branches for branches,  omega = 1.000 fixed

Codon frequency model: F3x4
Site-class models:  PositiveSelection
ns =   6  ls = 583

Codon usage in sequences
--------------------------------------------------------------------------------------------------------------------------------------
Phe TTT  11  11  11  11  11  11 | Ser TCT  11  10  10  10  11  11 | Tyr TAT  14  14  14  13  14  13 | Cys TGT   1   1   1   1   2   1
    TTC   5   5   5   5   5   4 |     TCC  10  11  11  12  11  11 |     TAC  11  11  11  12  11  12 |     TGC  11  11  11  11  10  11
Leu TTA   5   5   5   5   5   5 |     TCA  10  10  10  10  10  10 | *** TAA   0   0   0   0   0   0 | *** TGA   0   0   0   0   0   0
    TTG   9   8   9   9   9   9 |     TCG   1   1   1   1   0   0 |     TAG   0   0   0   0   0   0 | Trp TGG   6   6   6   6   6   6
--------------------------------------------------------------------------------------------------------------------------------------
Leu CTT   8   8   8   8   8   8 | Pro CCT   9  10   9  10   9  10 | His CAT   5   5   5   5   5   4 | Arg CGT   1   1   1   1   1   1
    CTC  10  10  10   9  11  12 |     CCC   6   6   6   5   6   6 |     CAC   3   3   3   3   3   4 |     CGC   1   1   1   1   1   1
    CTA   7   7   7   8   7   6 |     CCA  16  15  16  16  17  15 | Gln CAA   7   7   7   7   7   7 |     CGA   2   2   2   2   2   2
    CTG   9  10   9   9   8   9 |     CCG   1   1   1   1   0   1 |     CAG  12  12  12  12  11  12 |     CGG   0   0   0   0   1   0
--------------------------------------------------------------------------------------------------------------------------------------
Ile ATT  17  17  16  16  15  14 | Thr ACT  16  14  16  15  14  16 | Asn AAT  16  16  16  15  15  15 | Ser AGT  12  12  12  12  12  11
    ATC  12  12  13  12  14  13 |     ACC   6   7   6   7   7   7 |     AAC  15  14  14  15  15  14 |     AGC   6   7   7   7   7   8
    ATA  13  13  12  12  12  10 |     ACA  21  22  22  22  22  22 | Lys AAA  26  27  26  26  27  27 | Arg AGA   8   8   9   7   7   7
Met ATG  11  11  12  12  12  12 |     ACG   1   1   0   0   0   0 |     AAG  20  19  20  20  19  19 |     AGG   7   7   6   8   8   8
--------------------------------------------------------------------------------------------------------------------------------------
Val GTT   6   6   6   5   6   8 | Ala GCT   9   8   8   8   9   8 | Asp GAT  15  15  14  15  16  16 | Gly GGT   6   6   6   6   6   7
    GTC   8   8   8  10   8  10 |     GCC   8   9   9   8   9   9 |     GAC  13  13  14  13  12  12 |     GGC   9   9   9   9   9   8
    GTA  11  11  11  11  11  12 |     GCA  11  11  11  11  11  11 | Glu GAA  30  30  30  30  30  27 |     GGA  14  14  14  14  13  16
    GTG  17  17  17  17  17  16 |     GCG   1   1   1   1   1   1 |     GAG  13  13  13  13  13  16 |     GGG   3   3   3   3   4   1
--------------------------------------------------------------------------------------------------------------------------------------

Codon position x base (3x4) table for each sequence.

#1: human          
position  1:    T:0.18010    C:0.16638    A:0.35506    G:0.29846
position  2:    T:0.27273    C:0.23499    A:0.34305    G:0.14923
position  3:    T:0.26930    C:0.22985    A:0.31046    G:0.19039
Average         T:0.24071    C:0.21041    A:0.33619    G:0.21269

#2: chimpanzee     
position  1:    T:0.17839    C:0.16810    A:0.35506    G:0.29846
position  2:    T:0.27273    C:0.23499    A:0.34134    G:0.15094
position  3:    T:0.26415    C:0.23499    A:0.31218    G:0.18868
Average         T:0.23842    C:0.21269    A:0.33619    G:0.21269

#3: gorilla        
position  1:    T:0.18010    C:0.16638    A:0.35506    G:0.29846
position  2:    T:0.27273    C:0.23499    A:0.34134    G:0.15094
position  3:    T:0.26244    C:0.23671    A:0.31218    G:0.18868
Average         T:0.23842    C:0.21269    A:0.33619    G:0.21269

#4: orangutan      
position  1:    T:0.18182    C:0.16638    A:0.35334    G:0.29846
position  2:    T:0.27273    C:0.23499    A:0.34134    G:0.15094
position  3:    T:0.25901    C:0.23842    A:0.31046    G:0.19211
Average         T:0.23785    C:0.21326    A:0.33505    G:0.21384

#5: gibbon         
position  1:    T:0.18010    C:0.16638    A:0.35334    G:0.30017
position  2:    T:0.27273    C:0.23499    A:0.33962    G:0.15266
position  3:    T:0.26415    C:0.23842    A:0.31046    G:0.18696
Average         T:0.23899    C:0.21326    A:0.33448    G:0.21326

#6: rhesus         
position  1:    T:0.17839    C:0.16810    A:0.34820    G:0.30532
position  2:    T:0.27273    C:0.23671    A:0.33962    G:0.15094
position  3:    T:0.26415    C:0.24357    A:0.30360    G:0.18868
Average         T:0.23842    C:0.21612    A:0.33047    G:0.21498

Sums of codon usage counts
------------------------------------------------------------------------------
Phe F TTT      66 | Ser S TCT      63 | Tyr Y TAT      82 | Cys C TGT       7
      TTC      29 |       TCC      66 |       TAC      68 |       TGC      65
Leu L TTA      30 |       TCA      60 | *** * TAA       0 | *** * TGA       0
      TTG      53 |       TCG       4 |       TAG       0 | Trp W TGG      36
------------------------------------------------------------------------------
Leu L CTT      48 | Pro P CCT      57 | His H CAT      29 | Arg R CGT       6
      CTC      62 |       CCC      35 |       CAC      19 |       CGC       6
      CTA      42 |       CCA      95 | Gln Q CAA      42 |       CGA      12
      CTG      54 |       CCG       5 |       CAG      71 |       CGG       1
------------------------------------------------------------------------------
Ile I ATT      95 | Thr T ACT      91 | Asn N AAT      93 | Ser S AGT      71
      ATC      76 |       ACC      40 |       AAC      87 |       AGC      42
      ATA      72 |       ACA     131 | Lys K AAA     159 | Arg R AGA      46
Met M ATG      70 |       ACG       2 |       AAG     117 |       AGG      44
------------------------------------------------------------------------------
Val V GTT      37 | Ala A GCT      50 | Asp D GAT      91 | Gly G GGT      37
      GTC      52 |       GCC      52 |       GAC      77 |       GGC      53
      GTA      67 |       GCA      66 | Glu E GAA     177 |       GGA      85
      GTG     101 |       GCG       6 |       GAG      81 |       GGG      17
------------------------------------------------------------------------------


Codon position x base (3x4) table, overall

position  1:    T:0.17982    C:0.16695    A:0.35334    G:0.29989
position  2:    T:0.27273    C:0.23528    A:0.34105    G:0.15094
position  3:    T:0.26387    C:0.23699    A:0.30989    G:0.18925
Average         T:0.23880    C:0.21307    A:0.33476    G:0.21336


Nei & Gojobori 1986. dN/dS (dN, dS)
(Note: This matrix is not used in later ML. analysis.
Use runmode = -2 for ML pairwise comparison.)

human               
chimpanzee           0.0268 (0.0007 0.0278)
gorilla              0.0739 (0.0015 0.0201) 0.0328 (0.0007 0.0227)
orangutan            0.1072 (0.0030 0.0278) 0.0629 (0.0022 0.0355) 0.0656 (0.0015 0.0227)
gibbon               0.0780 (0.0030 0.0382) 0.0548 (0.0022 0.0408) 0.0451 (0.0015 0.0330) 0.0837 (0.0030 0.0356)
rhesus               0.0888 (0.0060 0.0673) 0.0747 (0.0052 0.0699) 0.0722 (0.0045 0.0619) 0.0925 (0.0060 0.0646) 0.1008 (0.0060 0.0592)


TREE #  1:  ((((1, 2), 3), 4), 5, 6);   MP score: 59
lnL(ntime:  9  np: 13):  -2713.893402      +0.000000
   7..8     8..9     9..10   10..1    10..2     9..3     8..4     7..5     7..6  
 0.004965 0.002274 0.003496 0.009384 0.011876 0.004757 0.012407 0.014523 0.041831 5.049037 1.000000 0.000000 0.099134

Note: Branch length is defined as number of nucleotide substitutions per codon (not per neucleotide site).

tree length =   0.10551

((((1: 0.009384, 2: 0.011876): 0.003496, 3: 0.004757): 0.002274, 4: 0.012407): 0.004965, 5: 0.014523, 6: 0.041831);

((((human: 0.009384, chimpanzee: 0.011876): 0.003496, gorilla: 0.004757): 0.002274, orangutan: 0.012407): 0.004965, gibbon: 0.014523, rhesus: 0.041831);

Detailed output identifying parameters

kappa (ts/tv) =  5.04904


dN/dS (w) for site classes (K=4)

site class             0        1       2a       2b
proportion       1.00000  0.00000  0.00000  0.00000
background w     0.09913  1.00000  0.09913  1.00000
foreground w     0.09913  1.00000  1.00000  1.00000


Time used:  0:23
```

---
