# Supplementary material for: The acquisition of novel N-glycosylation sites in conserved proteins during human evolution
Source: BMC Bioinformatics. 2015 Jan 28;16(1):29. doi: 10.1186/s12859-015-0468-5 (PMC4314935; doi:10.1186/s12859-015-0468-5)
Supplement: Additional file 6: — Molecular evolutionary analysis of thyroglobulin. [file 12859_2015_468_MOESM6_ESM.zip › 12859_2015_468_MOESM6_ESM.html]

## Additional file 6. Molecular evolutionary analysis of TG.

**1. Multiple sequence alignment of selected primate orthologs (positive sites by Model A are in magenta background)**

```
human       MALVLEIFTLLASICWVSANIFEYQVDAQPLRPCELQRETAFLKQADYVPQCAEDGSFQTVQCQNDGRSCWCVGANGSEVLGSRQPGRPVACLSFCQLQK  100
chimpanzee  ...........................................................................D........................  100
gorilla     ...................................................................H.......D........................  100
orangutan   .....G..S....V.............................................................D........................  100
gibbon      .....G..S..............................M...................................D........................  100
rhesus      ........S....V.........................R...................P...............D........................  100


human       QQILLSGYINSTDTSYLPQCQDSGDYAPVQCDVQQVQCWCVDAEGMEVYGTRQLGRPKRCPRSCEIRNRRLLHGVGDKSPPQCSAEGEFMPVQCKFVNTT  200
chimpanzee  ..........................T.........................................................................  200
gorilla     ..........................T.........................................................................  200
orangutan   ..........................T.........H...............................................................  200
gibbon      ..........................T.........................................................................  200
rhesus      ..........................M..........................P..............................................  200


human       DMMIFDLVHSYNRFPDAFVTFSSFQRRFPEVSGYCHCADSQGRELAETGLELLLDEIYDTIFAGLDLPSTFTETTLYRILQRRFLAVQSVISGRFRCPTK  300
chimpanzee  ....................................................................................................  300
gorilla     ....................................................................................................  300
orangutan   .........................................................................................L..........  300
gibbon      ....................................................................................................  300
rhesus      .....................N..............................................................................  300


human       CEVERFTATSFGHPYVPSCRRNGDYQAVQCQTEGPCWCVDAQGKEMHGTRQQGEPPSCAEGQSCASERQQALSRLYFGTSGYFSQHDLFSSPEKRWASPR  400
chimpanzee  ....................................................................................................  400
gorilla     .................................................................AK.................................  400
orangutan   ...................HQ..........M............................S.....K.................................  400
gibbon      ..................................................................K.................................  400
rhesus      ........................................V....I...............R....K.................................  400


human       VARFATSCPPTIKELFVDSGLLRPMVEGQSQQFSVSENLLKEAIRAIFPSRGLARLALQFTTNPKRLQQNLFGGKFLVNVGQFNLSGALGTRGTFNFSQF  500
chimpanzee  ....................................................................................................  500
gorilla     ....................................................................................................  500
orangutan   ......................H..............S..............................................................  500
gibbon      ...S...........................R.....S..............................................................  500
rhesus      .....................................S.........................................L....................  500


human       FQQLGLASFLNGGRQEDLAKPLSVGLDSNSSTGTPEAAKKDGTMNKPTVGSFGFEINLQENQNALKFLASLLELPEFLLFLQHAISVPEDVARDLGDVME  600
chimpanzee  ................................................................................................E...  600
gorilla     .....................F............................................................L.................  600
orangutan   ..................................A.......................R.........................................  600
gibbon      ..........................G................V........................................V...............  600
rhesus      .........S....L......V...................VA....I..G.................................................  600


human       TVLSSQTCEQTPERLFVPSCTTEGSYEDVQCFSGECWCVNSWGKELPGSRVRGGQPRCPTDCEKQRARMQSLMGSQPAGSTLFVPACTSEGHFLPVQCFN  700
chimpanzee  ................................A...................................................................  700
gorilla     ..F.............................A...........Q.......................................................  700
orangutan   M...............................A.D.............................................S...................  700
gibbon      ................................A......D........................................S...................  700
rhesus      ...R............................A......D.......S................................S..........Y........  700


human       SECYCVDAEGQAIPGTRSAIGKPKKCPTPCQLQSEQAFLRTVQALLSNSSMLPTLSDTYIPQCSTDGQWRQVQCNGPPEQVFELYQRWEAQNKGQDLTPA  800
chimpanzee  ...................T.............A........................................D..................--.....  798
gorilla     .................................A........................................D.........................  800
orangutan   .................................A...L..........................P.........D........W................  800
gibbon      .................................A................R.......................D......L.W...........Q....  800
rhesus      ...................M.............A............P.................A.........D........W...........E.M..  800


human       KLLVKIMSYREAASGNFSLFIQSLYEAGQQDVFPVLSQYPSLQDVPLAALEGKRPQPRENILLEPYLFWQILNGQLSQYPGSYSDFSTPLAHFDLRNCWC  900
chimpanzee  ....................................................................................................  898
gorilla     ...............................I....................................................................  900
orangutan   ..................F...........G.............................V...........................L...........  900
gibbon      ..............................G.............................V................R......................  900
rhesus      E................G............G.....................N.S.S...V..D.............R...P..................  900


human       VDEAGQELEGMRSEPSKLPTCPGSCEEAKLRVLQFIRETEEIVSASNSSRFPLGESFLVAKGIRLRNEDLGLPPLFPPREAFAEQFLRGSDYAIRLAAQS  1000
chimpanzee  ..........T.A.......................................................................................  998
gorilla     ..........T.A.......................................................................................  1000
orangutan   ..........T.A...........................D...........P...............................................  1000
gibbon      ..........T.A.............................................................P.........................  1000
rhesus      ..........T.A.................L..................................................L..................  1000


human       TLSFYQRRRFSPDDSAGASALLRSGPYMPQCDAFGSWEPVQCHAGTGHCWCVDEKGGFIPGSLTARSLQIPQCPTTCEKSRTSGLLSSWKQARSQENPSP  1100
chimpanzee  ...........................V................................A.......................................  1098
gorilla     ..........P............L...V....V........................L..A.......................................  1100
orangutan   ...........L...............................T................A............Q..........................  1100
gibbon      ......G....L...............V................................A.......................................  1100
rhesus      ...........L..........QL...V...............T................A..................................G....  1100


human       KDLFVPACLETGEYARLQASGAGTWCVDPASGEELRPGSSSSAQCPSLCNVLKSGVLSRRVSPGYVPACRAEDGGFSPVQCDQAQGSCWCVMDSGEEVPG  1200
chimpanzee  R......................................N............................................................  1198
gorilla     ......................................LN............................................................  1200
orangutan   ....................D..............L...N.....................G...............L......................  1200
gibbon      ....................E..............L...N.....S................A......................D..............  1200
rhesus      ....I...............E..............L...N......................S.......E....................T.......E  1200


human       TRVTGGQPACESPRCPLPFNASEVVGGTILCETISGPTGSAMQQCQLLCRQGSWSVFPPGPLICSLESGRWESQLPQPRACQRPQLWQTIQTQGHFQLQL  1300
chimpanzee  ...A.............................T.....A.I................................P..............M..........  1298
gorilla     ...A.............................T.......I................................P...W.....................  1300
orangutan   ...A.S..............V............T...I.A.I...........R....................P.........................  1300
gibbon      ...A.S...........................T.....A.I...........R.G............R.....P.........................  1300
rhesus      ...A.S..............TL...........A.....A.I...........R....R.........R...............................  1300


human       PPGKMCSADYADLLQTFQVFILDELTARGFCQIQVKTFGTLVSIPVCNNSSVQVGCLTRERLGVNVTWKSRLEDIPVASLPDLHDIERALVGKDLLGRFT  1400
chimpanzee  ...........S..............................................S.........................................  1398
gorilla     ..........TG........................................................................................  1400
orangutan   ...........G............................P................S..........................................  1400
gibbon      .......I...G...A.........M..............P.............................Q................T............  1400
rhesus      ...........G...A...........................V...D............Y.......................................  1400


human       DLIQSGSFQLHLDSKTFPAET-IRFLQGDHFGTSPRTWFGCSEGFYQVLTSEASQDGLGCVKCPEGSYSQDEECIPCPVGFYQEQAGSLACVPCPVGRTT  1499
chimpanzee  .....................T............................................................................M.  1498
gorilla     .....................T............................................................................M.  1500
orangutan   ...................D.T...............................................E............................M.  1500
gibbon      ...................D.T................................................E...........................M.  1500
rhesus      ...................D.T...................L.....................................................A..M.  1500


human       ISAGAFSQTHCVTDCQRNEAGLQCDQNGQYRASQKDRGSGKAFCVDGEGRRLPWWETEAPLEDSQCLMMQKFEKVPESKVIFDANAPVAVRSKVPDSEFP  1599
chimpanzee  ....................................................................................................  1598
gorilla     ....................................................................................................  1600
orangutan   ..............................Q...R.G.....................................A.........................  1600
gibbon      ....................................G............Q........................A.......N.S...--..........  1598
rhesus      ........................EE..E.....R..............W....S...................AA........S...............  1600


human       VMQCLTDCTEDEACSFFTVSTTEPEISCDFYAWTSDNVACMTSDQKRDALGNSKATSFGSLRCQVKVRSHGQDSPAVYLKKGQGSTTTLQKRFEPTGFQN  1699
chimpanzee  .....................M........................Q......................R.....................S........  1698
gorilla     ..K.................R.........................Q......................R.....................S........  1700
orangutan   ........A.....................................Q..............H.......R.....................S........  1700
gibbon      ........A.....................................Q......................R...................R.S........  1698
rhesus      ........A.......L...M.........................Q......................R.....................S........  1700


human       MLSGLYNPIVFSASGANLTDAHLFCLLACDRDLCCDGFVLTQVQGGAIICGLLSSPSVLLCNVKDWMDPSEAWANATCPGVTYDQESHQVILRLGDQEFI  1799
chimpanzee  .........................................R.....................................................G....  1798
gorilla     ............................................................................................H..G....  1800
orangutan   ..............................H...............DV........................R.................T.H..G....  1800
gibbon      ................................................V.............A.........R.................T....G....  1798
rhesus      .............................................V..........N...............R..............R..T.H..G....  1800


human       KSLTPLEGTQDTFTNFQQVYLWKDSDMGSRPESMGCRKDTVPRPASPTEAGLTTELFSPVDLNQVIVNGNQSLSSQKHWLFKHLFSAQQANLWCLSRCVQ  1899
chimpanzee  ....................................................................................................  1898
gorilla     .................................V..................................................................  1900
orangutan   ...........I.....................................T.......................P..........................  1900
gibbon      .........................................S.......T............D..........P..........................  1898
rhesus      R........................................S.......T....D.......D.......R..P....L.....................  1900


human       EHSFCQLAEITESASLYFTCTLYPEAQVCDDIMESNAQGCRLILPQMPKALFRKKVILEDKVKNFYTRLPFQKLMGISIRNKVPMSEKSISNGFFECERR  1999
chimpanzee  ....................................................................................................  1998
gorilla     ..........................................V........................................S................  2000
orangutan   ..........................................................................L........................Q  2000
gibbon      ............G.................................R.....................................................  1998
rhesus      ......F.........................L.............R.....Q...............................................  2000


human       CDADPCCTGFGFLNVSQLKGGEVTCLTLNSLGIQMCSEENGGAWRILDCGSPDIEVHTYPFGWYQKPIAQNNAPSFCPLVVLPSLTEKVSLDSWQSLALS  2099
chimpanzee  .....................K..............................................................................  2098
gorilla     ....................................................................................................  2100
orangutan   ............................S.................................................S.............L.......  2100
gibbon      .......................................S...........R..........................S.....V...............  2098
rhesus      ................................L......................................S......S.....................  2100


human       SVVVDPSIRHFDVAHVSTAATSNFSAVRDLCLSECSQHEACLITTLQTQPGAVRCMFYADTQSCTHSLQGQNCRLLLREEATHIYRKPGISLLSYEASVP  2199
chimpanzee  ...................................................................................................H  2198
gorilla     .......................................................................D............................  2200
orangutan   ..A.................................................................................................  2200
gibbon      .................I.......................................................Q..........................  2198
rhesus      .......................................D.....................H...Y................R.................  2200


human       SVPISTHGRLLGRSQAIQVGTSWKQVDQFLGVPYAAPPLAERRFQAPEPLNWTGSWDASKPRASCWQPGTRTSTSPGVSEDCLYLNVFIPQNVAPNASVL  2299
chimpanzee  ............................................R...............................R.......................  2298
gorilla     .............................................E......................................................  2300
orangutan   ........Q........H......R................................................MT.........................  2300
gibbon      ........Q........R..........................R...................................................M...  2298
rhesus      ..L.V.............L.........................R.......................................................  2300


human       VFFHNTMDREESEGWPAIDGSFLAAVGNLIVVTASYRVGVFGFLSSGSGEVSGNWGLLDQVAALTWVQTHIRGFGGDPRRVSLAADRGGADVASIHLLTA  2399
chimpanzee  ........G........................................D..................................................  2398
gorilla     ........G.............................................................................H.............  2400
orangutan   ........G.....................................................................Q.....................  2400
gibbon      ......K.G.........................G...........................................G..................F..  2398
rhesus      ..........G.....................................R......................................A..........M.  2400


human       RATNSQLFRRAVLMGGSALSPAAVISHERAQQQAIALAKEVSCPMSSSQEVVSCLRQKPANVLNDAQTKLLAVSGPFHYWGPVIDGHFLREPPARALKRS  2499
chimpanzee  ............................................................S.........................Q.............  2498
gorilla     ............................................T...............S.........................Q.............  2500
orangutan   ......................................................................................Q.............  2500
gibbon      ......................................................................................Q.............  2498
rhesus      .......................I..........V.........V................I........................Q.............  2500


human       LWVEVDLLIGSSQDDGLINRAKAVKQFEESRGRTSSKTAFYQALQNSLGGEDSDARVEAAATWYYSLEHSTDDYASFSRALENATRDYFIICPIIDMASA  2599
chimpanzee  .R............................Q.....................................................................  2598
gorilla     .R...........E................Q.....................................................................  2600
orangutan   ..L...........................Q.....................................................................  2600
gibbon      .R............................Q.....................................................................  2598
rhesus      .RA...........................Q.....................................................................  2600


human       WAKRARGNVFMYHAPENYGHGSLELLADVQFALGLPFYPAYEGQFSLEEKSLSLKIMQYFSHFIRSGNPNYPYEFSRKVPTFATPWPDFVPRAGGENYKE  2699
chimpanzee  ................S..R............F...................................................................  2698
gorilla     .............V..S...............F...................................................................  2700
orangutan   ................S..R............F...................................................................  2700
gibbon      ................S..R............F..........................................................H........  2698
rhesus      ................S..R............F..........................................................Y........  2700


human       FSELLPNRQGLKKADCSFWSKYISSLKTSADGAKGGQSAESEEEELTAGSGLREDLLSLQEPGSKTYSK  2768
chimpanzee  ..A........................A........K......-.....................S...  2766
gorilla     ..A........................A.....................................S...  2769
orangutan   ..A........................A.....................................S...  2769
gibbon      ..A........................A.............Q.G.....................S...  2767
rhesus      ..A........................A.....................................S...  2769
```

---

**2. Sequence data file "tg.phy"**

```
6 8304

human
ATG GCC CTG GTC CTG GAG ATC TTC ACC CTG CTG GCC TCC ATC TGC TGG GTG TCG GCC AAT ATC TTC
GAG TAC CAG GTG GAT GCC CAG CCC CTT CGT CCC TGT GAG CTG CAG AGG GAA ACG GCC TTT CTG AAG
CAA GCA GAC TAC GTG CCC CAG TGT GCA GAG GAT GGC AGC TTC CAG ACT GTC CAG TGC CAG AAC GAC
GGC CGC TCC TGC TGG TGT GTG GGT GCC AAC GGC AGT GAA GTG CTG GGC AGC AGG CAG CCA GGA CGG
CCT GTG GCT TGT CTG TCA TTT TGT CAG CTA CAG AAA CAG CAG ATC TTA CTG AGT GGC TAC ATT AAC
AGC ACA GAC ACC TCC TAC CTC CCT CAG TGT CAG GAT TCA GGG GAC TAC GCG CCT GTT CAG TGT GAT
GTG CAG CAG GTC CAG TGC TGG TGT GTG GAC GCA GAG GGG ATG GAG GTG TAT GGG ACC CGC CAG CTG
GGG AGG CCA AAG CGA TGT CCA AGG AGC TGT GAA ATA AGA AAT CGT CGT CTT CTC CAC GGG GTG GGA
GAT AAG TCA CCA CCC CAG TGT TCT GCG GAG GGA GAG TTT ATG CCT GTC CAG TGC AAA TTT GTC AAC
ACC ACA GAC ATG ATG ATT TTT GAT CTG GTC CAC AGC TAC AAC AGG TTT CCA GAT GCA TTT GTG ACC
TTC AGT TCC TTC CAG AGG AGG TTC CCT GAG GTA TCT GGG TAT TGC CAC TGT GCT GAC AGC CAA GGG
CGG GAA CTG GCT GAG ACA GGT TTG GAG TTG TTA CTG GAT GAA ATT TAT GAC ACC ATT TTT GCT GGC
CTG GAC CTT CCT TCC ACC TTC ACT GAA ACC ACC CTG TAC CGG ATA CTG CAG AGA CGG TTC CTC GCA
GTT CAA TCA GTC ATC TCT GGC AGA TTC CGA TGC CCC ACA AAA TGT GAA GTG GAG CGG TTT ACA GCA
ACC AGC TTT GGT CAC CCC TAT GTT CCA AGC TGC CGC CGA AAT GGC GAC TAT CAG GCG GTG CAG TGC
CAG ACG GAA GGG CCC TGC TGG TGT GTG GAC GCC CAG GGG AAG GAA ATG CAT GGA ACC CGG CAG CAA
GGG GAG CCG CCA TCT TGT GCT GAA GGC CAA TCT TGT GCC TCC GAA AGG CAG CAG GCC TTG TCC AGA
CTC TAC TTT GGG ACC TCA GGC TAC TTC AGC CAG CAC GAC CTG TTC TCT TCC CCA GAG AAA AGA TGG
GCC TCT CCA AGA GTA GCC AGA TTT GCC ACA TCC TGC CCA CCC ACG ATC AAG GAG CTC TTT GTG GAC
TCT GGG CTT CTC CGC CCA ATG GTG GAG GGA CAG AGC CAA CAG TTT TCT GTC TCA GAA AAT CTT CTC
AAA GAA GCC ATC CGA GCA ATT TTT CCC TCC CGA GGG CTG GCT CGT CTT GCC CTT CAG TTT ACC ACC
AAC CCA AAG AGA CTC CAG CAA AAC CTT TTT GGA GGG AAA TTT TTG GTG AAT GTT GGC CAG TTT AAC
TTG TCT GGA GCC CTT GGC ACA AGA GGC ACA TTT AAC TTC AGT CAA TTT TTC CAG CAA CTT GGT CTT
GCA AGC TTC TTG AAT GGA GGG AGA CAA GAA GAT TTG GCC AAG CCA CTC TCT GTG GGA TTA GAT TCA
AAT TCT TCC ACA GGA ACC CCT GAA GCT GCT AAG AAG GAT GGT ACT ATG AAT AAG CCA ACT GTG GGC
AGC TTT GGC TTT GAA ATT AAC CTA CAA GAG AAC CAA AAT GCC CTC AAA TTC CTT GCT TCT CTC CTG
GAG CTT CCA GAA TTC CTT CTC TTC TTG CAA CAT GCT ATC TCT GTG CCA GAA GAT GTG GCA AGA GAT
TTA GGT GAT GTG ATG GAA ACG GTA CTC AGC TCC CAG ACC TGT GAG CAG ACA CCT GAA AGG CTA TTT
GTC CCA TCA TGC ACG ACA GAA GGA AGC TAT GAG GAT GTC CAA TGC TTT TCC GGA GAG TGC TGG TGT
GTG AAT TCC TGG GGC AAA GAG CTT CCA GGC TCA AGA GTC AGA GGT GGA CAG CCA AGG TGC CCC ACA
GAC TGT GAA AAG CAA AGG GCT CGC ATG CAA AGC CTC ATG GGC AGC CAG CCT GCT GGC TCC ACC TTG
TTT GTC CCT GCT TGT ACT AGT GAG GGA CAT TTC CTG CCT GTC CAG TGC TTC AAC TCA GAG TGC TAC
TGT GTT GAT GCT GAG GGT CAG GCC ATT CCT GGA ACT CGA AGT GCA ATA GGG AAG CCC AAG AAA TGC
CCC ACG CCC TGT CAA TTA CAG TCT GAG CAA GCT TTC CTC AGG ACG GTG CAG GCC CTG CTC TCT AAC
TCC AGC ATG CTA CCC ACC CTT TCC GAC ACC TAC ATC CCA CAG TGC AGC ACC GAT GGG CAG TGG AGA
CAA GTG CAA TGC AAT GGG CCT CCT GAG CAG GTC TTC GAG TTG TAC CAA CGA TGG GAG GCT CAG AAC
AAG GGC CAG GAT CTG ACG CCT GCC AAG CTG CTA GTG AAG ATC ATG AGC TAC AGA GAA GCA GCT TCC
GGA AAC TTC AGT CTC TTT ATT CAA AGT CTG TAT GAG GCT GGC CAG CAA GAT GTC TTC CCG GTG CTG
TCA CAA TAC CCT TCT CTG CAA GAT GTC CCA CTA GCA GCA CTG GAA GGG AAA CGG CCC CAG CCC AGG
GAG AAT ATC CTC CTG GAG CCC TAC CTC TTC TGG CAG ATC TTA AAT GGC CAA CTC AGC CAA TAC CCG
GGG TCC TAC TCA GAC TTC AGC ACT CCT TTG GCA CAT TTT GAT CTT CGG AAC TGC TGG TGT GTG GAT
GAG GCT GGC CAA GAA CTG GAA GGA ATG CGG TCT GAG CCA AGC AAG CTC CCA ACA TGT CCT GGC TCC
TGT GAG GAA GCA AAG CTC CGT GTA CTG CAG TTC ATT AGG GAA ACG GAA GAG ATT GTT TCA GCT TCC
AAC AGT TCT CGG TTC CCT CTG GGG GAG AGT TTC CTG GTG GCC AAG GGA ATC CGG CTG AGG AAT GAG
GAC CTC GGC CTT CCT CCG CTC TTC CCG CCC CGG GAG GCT TTC GCG GAG CAG TTT CTG CGT GGG AGT
GAT TAC GCC ATT CGC CTG GCG GCT CAG TCT ACC TTA AGC TTC TAT CAG AGA CGC CGC TTT TCC CCG
GAC GAC TCG GCT GGA GCA TCC GCC CTT CTG CGG TCG GGC CCC TAC ATG CCA CAG TGT GAT GCG TTT
GGA AGT TGG GAG CCT GTG CAG TGC CAC GCT GGG ACT GGG CAC TGC TGG TGT GTA GAT GAG AAA GGA
GGG TTC ATC CCT GGC TCA CTG ACT GCC CGC TCT CTG CAG ATT CCA CAG TGC CCG ACA ACC TGC GAG
AAA TCT CGA ACC AGT GGG CTG CTT TCC AGT TGG AAA CAG GCT AGA TCC CAA GAA AAC CCA TCT CCA
AAA GAC CTG TTC GTC CCA GCC TGC CTA GAA ACA GGA GAG TAT GCC AGG CTG CAG GCA TCG GGG GCT
GGC ACC TGG TGT GTG GAC CCT GCA TCA GGA GAA GAG TTG CGG CCT GGC TCG AGC AGC AGT GCC CAG
TGC CCA AGC CTC TGC AAT GTG CTC AAG AGT GGA GTC CTC TCC AGG AGA GTC AGC CCA GGC TAT GTC
CCA GCC TGC AGG GCA GAG GAT GGG GGC TTT TCC CCA GTG CAA TGT GAC CAG GCC CAG GGC AGC TGC
TGG TGT GTC ATG GAC AGC GGA GAA GAG GTG CCT GGG ACG CGC GTG ACC GGG GGC CAG CCC GCC TGT
GAG AGC CCG CGG TGT CCG CTG CCA TTC AAC GCG TCG GAG GTG GTT GGT GGA ACA ATC CTG TGT GAG
ACA ATC TCG GGC CCC ACA GGC TCT GCC ATG CAG CAG TGC CAA TTG CTG TGC CGC CAG GGC TCC TGG
AGC GTG TTT CCA CCA GGG CCA TTG ATA TGT AGC CTG GAG AGC GGA CGC TGG GAG TCA CAG CTG CCT
CAG CCC CGG GCC TGC CAA CGG CCC CAG CTG TGG CAG ACC ATC CAG ACC CAA GGG CAC TTT CAG CTC
CAG CTC CCG CCG GGC AAG ATG TGC AGT GCT GAC TAC GCG GAT TTG CTG CAG ACT TTC CAG GTT TTC
ATA TTG GAT GAG CTG ACA GCC CGC GGC TTC TGC CAG ATC CAG GTG AAG ACT TTT GGC ACC CTG GTT
TCC ATT CCT GTC TGC AAC AAC TCC TCT GTG CAG GTG GGT TGT CTG ACC AGG GAG CGT TTA GGA GTG
AAT GTT ACA TGG AAA TCA CGG CTT GAG GAC ATC CCA GTG GCT TCT CTT CCT GAC TTA CAT GAC ATT
GAG AGA GCC TTG GTG GGC AAG GAT CTC CTT GGG CGC TTC ACA GAT CTG ATC CAG AGT GGC TCA TTC
CAG CTT CAT CTG GAC TCC AAG ACG TTC CCA GCG GAA ACC ATC CGC TTC CTC CAA GGG GAC CAC TTT
GGC ACC TCT CCC AGG ACA TGG TTT GGG TGC TCG GAA GGA TTC TAC CAA GTC TTG ACA AGT GAG GCC
AGT CAG GAC GGA CTG GGA TGC GTT AAG TGT CCT GAA GGA AGC TAT TCC CAA GAT GAG GAA TGC ATT
CCT TGT CCT GTT GGA TTC TAC CAA GAA CAG GCA GGG AGC TTG GCC TGT GTC CCA TGT CCT GTG GGC
AGA ACG ACC ATT TCT GCT GGA GCT TTC AGC CAG ACT CAC TGT GTC ACT GAC TGT CAG AGG AAC GAA
GCA GGC CTG CAA TGT GAC CAG AAT GGC CAG TAT CGA GCC AGC CAG AAG GAC AGG GGC AGT GGG AAG
GCC TTC TGT GTG GAC GGC GAG GGG CGG AGG CTG CCA TGG TGG GAA ACA GAG GCC CCT CTT GAG GAC
TCA CAG TGT TTG ATG ATG CAG AAG TTT GAG AAG GTT CCA GAA TCA AAG GTG ATC TTC GAC GCC AAT
GCT CCT GTG GCT GTC AGA TCC AAA GTT CCT GAT TCT GAG TTC CCC GTG ATG CAG TGC TTG ACA GAT
TGC ACA GAG GAC GAG GCC TGC AGC TTC TTC ACC GTG TCC ACG ACG GAG CCA GAG ATT TCC TGT GAT
TTC TAT GCT TGG ACA AGT GAC AAT GTT GCC TGC ATG ACT TCT GAC CAG AAA CGA GAT GCA CTG GGG
AAC TCA AAG GCC ACC AGC TTT GGA AGT CTT CGC TGC CAG GTG AAA GTG AGG AGC CAT GGT CAA GAT
TCT CCA GCT GTG TAT TTG AAA AAG GGC CAA GGA TCC ACC ACA ACA CTT CAG AAA CGC TTT GAA CCC
ACT GGT TTC CAA AAC ATG CTT TCT GGA TTG TAC AAC CCC ATT GTG TTC TCA GCC TCA GGA GCC AAT
CTA ACC GAT GCT CAC CTC TTC TGT CTT CTT GCA TGC GAC CGT GAT CTG TGT TGC GAT GGC TTC GTC
CTC ACA CAG GTT CAA GGA GGT GCC ATC ATC TGT GGG TTG CTG AGC TCA CCC AGT GTC CTG CTT TGT
AAT GTC AAA GAC TGG ATG GAT CCC TCT GAA GCC TGG GCT AAT GCT ACA TGT CCT GGT GTG ACA TAT
GAC CAG GAG AGC CAC CAG GTG ATA TTG CGT CTT GGA GAC CAG GAG TTC ATC AAG AGT CTG ACA CCC
TTA GAA GGA ACT CAA GAC ACC TTT ACC AAT TTT CAG CAG GTT TAT CTC TGG AAA GAT TCT GAC ATG
GGG TCT CGG CCT GAG TCT ATG GGA TGT AGA AAA GAC ACA GTG CCA AGG CCA GCA TCT CCA ACA GAA
GCA GGT TTG ACA ACA GAA CTT TTC TCC CCT GTG GAC CTC AAC CAG GTC ATT GTC AAT GGA AAT CAA
TCA CTA TCC AGC CAG AAG CAC TGG CTT TTC AAG CAC CTG TTT TCA GCC CAG CAG GCA AAC CTA TGG
TGC CTT TCT CGT TGT GTG CAG GAG CAC TCT TTC TGT CAG CTC GCA GAG ATA ACA GAG AGT GCA TCC
TTG TAC TTC ACC TGC ACC CTC TAC CCA GAG GCA CAG GTG TGT GAT GAC ATC ATG GAG TCC AAT GCC
CAG GGC TGC AGA CTG ATC CTG CCT CAG ATG CCA AAG GCC CTG TTC CGG AAG AAA GTT ATA CTG GAA
GAT AAA GTG AAG AAC TTT TAC ACT CGC CTG CCG TTC CAA AAA CTG ATG GGG ATA TCC ATT AGA AAT
AAA GTG CCC ATG TCT GAA AAA TCT ATT TCT AAT GGG TTC TTT GAA TGT GAA CGA CGG TGC GAT GCG
GAC CCA TGC TGC ACT GGC TTT GGA TTT CTA AAT GTT TCC CAG TTA AAA GGA GGA GAG GTG ACA TGT
CTC ACT CTG AAC AGC TTG GGA ATT CAG ATG TGC AGT GAG GAG AAT GGA GGA GCC TGG CGC ATT TTG
GAC TGT GGC TCT CCT GAC ATT GAA GTC CAC ACC TAT CCC TTC GGA TGG TAC CAG AAG CCC ATT GCT
CAA AAT AAT GCT CCC AGT TTT TGC CCT TTG GTT GTT CTG CCT TCC CTC ACA GAG AAA GTG TCT CTG
GAC TCG TGG CAG TCC CTG GCC CTC TCT TCA GTG GTT GTT GAT CCA TCC ATT AGG CAC TTT GAT GTT
GCC CAT GTC AGC ACT GCT GCC ACC AGC AAT TTC TCT GCT GTC CGA GAC CTC TGT TTG TCG GAA TGT
TCC CAA CAT GAG GCC TGT CTC ATC ACC ACT CTG CAA ACC CAA CCT GGG GCT GTG AGA TGT ATG TTC
TAT GCT GAT ACT CAA AGC TGC ACA CAT AGT CTG CAG GGT CAG AAC TGC CGA CTT CTG CTT CGT GAA
GAG GCC ACC CAC ATC TAC CGG AAG CCA GGA ATC TCT CTG CTC AGC TAT GAG GCA TCT GTA CCT TCT
GTG CCC ATT TCC ACC CAT GGC CGG CTG CTG GGC AGG TCC CAG GCC ATC CAG GTG GGT ACC TCA TGG
AAG CAA GTG GAC CAG TTC CTT GGA GTT CCA TAT GCT GCC CCG CCC CTG GCA GAG AGG CGC TTC CAG
GCA CCA GAG CCC TTG AAC TGG ACA GGC TCC TGG GAT GCC AGC AAG CCA AGG GCC AGC TGC TGG CAG
CCA GGC ACC AGA ACA TCC ACG TCT CCT GGA GTC AGT GAA GAT TGT TTG TAT CTC AAT GTG TTC ATC
CCT CAG AAT GTG GCC CCT AAC GCG TCT GTG CTG GTG TTC TTC CAC AAC ACC ATG GAC AGG GAG GAG
AGT GAA GGA TGG CCG GCT ATC GAC GGC TCC TTC TTG GCT GCT GTT GGC AAC CTC ATC GTG GTC ACT
GCC AGC TAC CGA GTG GGT GTC TTC GGC TTC CTG AGT TCT GGG TCC GGA GAG GTG AGT GGC AAC TGG
GGG CTG CTG GAC CAG GTG GCG GCT CTG ACC TGG GTG CAG ACC CAC ATC CGA GGA TTT GGC GGG GAC
CCT CGG CGC GTG TCC CTG GCA GCA GAC CGT GGC GGG GCT GAT GTG GCC AGC ATC CAC CTT CTC ACG
GCC AGG GCC ACC AAC TCC CAA CTT TTC CGG AGA GCT GTG CTG ATG GGA GGC TCC GCA CTC TCC CCG
GCC GCC GTC ATC AGC CAT GAG AGG GCT CAG CAG CAG GCA ATT GCT TTG GCA AAG GAG GTC AGT TGC
CCC ATG TCA TCC AGC CAA GAA GTG GTG TCC TGC CTC CGC CAG AAG CCT GCC AAT GTC CTC AAT GAT
GCC CAG ACC AAG CTC CTG GCC GTG AGT GGC CCT TTC CAC TAC TGG GGT CCT GTG ATC GAT GGC CAC
TTC CTC CGT GAG CCT CCA GCC AGA GCA CTG AAG AGG TCT TTA TGG GTA GAG GTC GAT CTG CTC ATT
GGG AGT TCT CAG GAC GAC GGG CTC ATC AAC AGA GCA AAG GCT GTG AAG CAA TTT GAG GAA AGT CGA
GGC CGG ACC AGT AGC AAA ACA GCC TTT TAC CAG GCA CTG CAG AAT TCT CTG GGT GGC GAG GAC TCA
GAT GCC CGC GTC GAG GCT GCT GCT ACA TGG TAT TAC TCT CTG GAG CAC TCC ACG GAT GAC TAT GCC
TCC TTC TCC CGG GCT CTG GAG AAT GCC ACC CGG GAC TAC TTT ATC ATC TGC CCT ATA ATC GAC ATG
GCC AGT GCC TGG GCA AAG AGG GCC CGA GGA AAC GTC TTC ATG TAC CAT GCT CCT GAA AAC TAC GGC
CAT GGC AGC CTG GAG CTG CTG GCG GAT GTT CAG TTT GCC TTG GGG CTT CCC TTC TAC CCA GCC TAC
GAG GGG CAG TTT TCT CTG GAG GAG AAG AGC CTG TCG CTG AAA ATC ATG CAG TAC TTT TCC CAC TTC
ATC AGA TCA GGA AAT CCC AAC TAC CCT TAT GAG TTC TCA CGG AAA GTA CCC ACA TTT GCA ACC CCC
TGG CCT GAC TTT GTA CCC CGT GCT GGT GGA GAG AAC TAC AAG GAG TTC AGT GAG CTG CTC CCC AAT
CGA CAG GGC CTG AAG AAA GCC GAC TGC TCC TTC TGG TCC AAG TAC ATC TCG TCT CTG AAG ACA TCT
GCA GAT GGA GCC AAG GGC GGG CAG TCA GCA GAG AGT GAA GAG GAG GAG TTG ACG GCT GGA TCT GGG
CTA AGA GAA GAT CTC CTA AGC CTC CAG GAA CCA GGC TCT AAG ACC TAC AGC AAG
chimpanzee
... ... ... ... ... ... ... ... ... ... ... ... ... ... ... ... ... ... ... ... ... ...
... ... ... ... ... ... ... ... ... ... ... ... ... ... ... ... ... ... ... ... ... ...
... ... ... ... ... ... ... ... ... ... ... ... ... ... ... ... ... ... ... ... ... ..T
... ... ... ... ... ... ... ... ... G.. ... ... ... ... ... ... ... ... ... ... ... ...
... ... ... ... ... ... ... ... ... ... ... ... ... ... ... ... ... ... ... ... ... ...
... ... ... ... ... ... ... ... ... ... ... ... ... ... ... ... A.. ... ... ... ... ..C
... ... ... ... ... ... ... ... ... ... ... ... ... ... ... ... ... ... ... ... ... ...
... ... ... ... ... ... ... ... ... ... ... ... ... ... ... ... ... ... ... ... ... ...
... ... ... ... ... ... ... ... ... ... ... ... ... ... ... ... ... ... ... ..C ... ...
... ... ... ... ... ... ... ... ... ... ... ... ... ... ... ... ... ... ... ... ... ...
... ... ... ... ... ... ... ... ... ... ..G ... ... ... ... ... ... ... ... ... ... ...
... ... ... ... ... ... ... ... ... ... ... ... ... ... ... ... ... ... ... ... ... ...
... ... ... ... ... ... ... ... ... ... ... ... ... ... ... ... ... ... ... ... ... ...
... ... ... ... ... ... ... ... ... ... ... ... ... ... ... ... ... ... ... ... ... ...
... ... ... ... ... ... ... ... ... ... ... ... ... ... ... ... ... ... ... ... ... ...
... ... ... ... ... ... ... ... ... ... ... ... ... ... ... ... ... ... ... ... ... ...
... ... ... ... ... ... ... ... ... ... ... ... ... ... ... ... ... ... ... ... ... ...
... ... ... ... ... ... ... ... ... ... ... ... ... ... ... ... ... ... ... ... ... ...
... ... ... ... ... ... ... ... ... ... ... ... ... ... ... ... ... ... ... ... ... ...
... ... ... ... ... ... ... ... ... ... ... ... ... ... ... ... ... ... ... ... ... ...
... ... ... ... ... ... ... ... ... ... ... ... ... ... ... ... ... ... ... ... ... ...
... ... ... ... ... ... ... ... ... ... ... ... ... ... ... ... ... ... ... ... ... ...
... ... ... ... ... ... ... ... ... ... ... ... ... ... ... ... ... ... ... ... ... ...
... ... ... ... ... ... ... ... ... ... ... ... ... ... ... ... ... ... ... ... ... ...
... ... ... ... ... ... ... ... ... ... ... ... ... ... ... ... ... ... ... ... ... ...
... ... ... ... ... ... ... ... ... ... ... ... ... ... ... ... ... ... ... ... ... ...
... ... ... ... ... ... ... ... ... ... ... ... ... ... ... ... ... ... ... ... ... ...
... ... ..A ... ... ... ... ... ... ... ... ... ... ... ... ... ... ... ... ... ... ...
... ... ... ... ... ... ... ... ... ... ..A ... ... ... ... ... G.. ... ... ... ... ...
... ... ... ... ... ... ... ... ... ... ... ... ... ... ... ... ... ..G ... ... ... ...
... ... ... ... ... ... ... ... ... ... ... ... ... ... ... ... ... ... ... ... ... ...
... ... ... ... ... ... ... ... ... ... ... ... ... ... ... ... ... ... ... ... ... ...
... ... ... ... ... ... ... ... ... ... ... ... ... ... ... .C. ... ... ... ... ... ...
... ... ... ... ... ... ... G.. ... ... ... ... ... ... ... ... ... ... ... ... ... ...
... ... ... ... ... ... ... ... ... ... ... ... ... ... ... ... ... ... ... ... ... ...
... ... ... ... G.. ... ... ..C ... ... ... ... ... ... ... ... ... ... ... ... ... ...
... --- --- ... ... ... ... ... ... ... ... ... ... ... ... ... ... ... ... ... ... ...
... ... ... ... ... ... ... ... ... ... ... ... ... ... ... ... ... ... ... ... ... ...
... ... ... ... ... ... ... ... ... ... ... ... ... ... ... ... ... ... ... ... ... ...
... ... ... ... ... ... ... ... ... ... ... ... ... ... ... ... ... ... ... ... ... ...
..C ... ... ... ... ... ... ... ... ... ... ... ..C ... ... ... ... ... ... ... ... ...
... ... ... ... ... ... ... ... .C. ... G.. ... ... ... ... ... ... ... ... ... ... ...
... ... ... ..G ... ... ... ... ... ... ... ... ... ... ... ... ... ... ... ... ... ...
... ... ... ... ... ... ... ... ... ... ... ... ... ... ... ... ... ... ... ... ... ...
... ... ... ... ... ... ... ... ... ... ... ... ... ... ... ... ... ... ... ..C ... ...
... ... ... ... ... ... ... ... ... ... ... ... ... ... ... ... ... ... ... ... ... ...
... ... ... ... ... ... ... ... ... ... ... ... ... ... ... G.. ... ... ... ... ... ...
... ... ... ... ... ... ... ... ... ... ... ... ... ... ... ... ... ... ... ... ... ...
... ... ... ... .C. ... ... ... ... ... ... ... ... ... ... ... ... ... ... ... ... ...
... ... ... ... ... ... ... ... ... ... ... ... ... ... ... ... ... ... ... ... ... ...
.G. ... ... ... ... ... ... ... ... ..G ... ... ... ... ... ... ... ... ... ... ... ...
... ... ... ... ... ... ... ... ... ... ... ... ... ..T ... ... ... .A. ... ... ... ...
... ... ... ... ... ... ... ... ... ... ... ... ... ... ... ... ... ... ... ... ... ...
... ... ... ... ... ... ... ... ... ... ... ... ... ... ... ... ... ..G ... ... ... ...
... ... ... ... ... ... ... ... ... ... ... ... ... ... ... G.. ... ... ... ... ... ...
... ... ... ... ... ... ... ... ... ... ... ... ... ... ... ... ... ... ... ... ... ...
... .C. ... ... ... ... ..G G.. ... ..C ... ... ... ... ... ... ... ... ... ... ... ...
... ... ... ... ... ... ... ... ... ... ... ... ... ... ... ... ... ... ... ... .C. ...
... ... ... ... ... ... ... ... ... ... ... ... ... ..G ... ... ... ... ... ... ... ...
... ... ... ... ... ... ... ... ... ... ... ..T ... AG. ... ... ... ... ... ... ... ...
... ... ... ... ... ..G ... ... ... ... ... ... ... ... ... ... ... ... ... ... ... ...
... ... ... ... ... ... ... ... ... ... ... ... ... ... ... ... ..C ... ... ... ... ...
... ... ... ... ... ... ... ... ... ... ... ... ..A ... ... ... ... ... ... ... ... ...
... ... ... ... ... ... ... ... ... ... ... ... ... ... ... ... ... ... ... ... ... ...
... ... ... ... ... ... ... ... ... ... ... ... ... ... ... ... ... ... ... ... ... ...
... ... ... ... ... ... ... ... ... ... ... ... ... ... ... ... ... ... ... ... ... ...
... ... ... ... ... ... ... ... ... ... ... ... ... ... ... ... ... ... ... ... ... ...
... ... ... ... ... ... ... ... ... ... ... ... ... ... ... ... ... ... ... ... ... ...
... .T. ... ... ... ..C ... ... ... ... ... ... ... ... ... ..A ... ... ... ... ... ...
... ... ... ... ... ... ... ... ... ... ... ... ... ... ... ... ... ... ... ... ... ...
... ... ... ... ..T ... ... ... ... ... ... ... ... ... ... ... ... ... ..A ... ... ...
... ... ... ... ... ... ... ... ... ... ... ... ... ... ... ... ... ... ... ..T ... ...
... ... ... ... ... ... ... ... ... ... ... ... ... ... ... ... ... ... ... ... ... ...
... ... ... ... ... ... ... ... ... ... ... ... ... ... .T. ... ... ... ... ... ... ...
... ... ... ... ... ... ... ... ... ... ... ... ... ... ... ... ... .A. ... ... ... ...
... ... ... ... ... ... ... ... ... ... ... ... ... ... ... ... ... ... .G. ... ... ...
... ... ... ... ... ... ... ... ... ... ... ... ... ... ... ... ... ... A.. ... ... ...
... ... ... ... ... ... ... ... ... ... ... ... ... ... ... ... ... ... ... ... ... ...
..G ... ... ... ... ... ... ... ... ... ... ... ... ... ... ... ... ... ... ... ... ...
... ... .G. ... ... ... ... ... ... ... ... ... ... ... ... ... ... ... ... ... ... ...
... ... ... ... ... ... ... ... ... ... ... ... ... ... ... ... ... ... ... ... ... ...
... ... ... ... ... ... ... ... ... ... ... ... .G. ... ... ... ... ... ... ... ... ...
C.. ... ... ... ... ... ... ... ... ... ... ... ... ... ... ... ... ... ... ... ... ...
... ... ... ... ... ... ... ... ... ... ... ... ... ... ... ... ... ... ... ... ... ...
... ... ... ... ... ... ... ... ... ... ... ... ... ... ... ... ... ... ... ... ... ...
... ... ... ... ... ... ... ... ... ... ... ... ... ... ... ... ... ... ... ... ..G ...
... ... ... ... ... ... ... ... ... ... ... ... ... ... ... ... ... ... ... ... ... ...
... ... ... ... ... ... ... ... ... ... ... ... ... ... ... ... ... ... ... ... ... ...
... ... ... ... ... ... ... ... ... ... ... ... ... ... ... ... ... ... ... ... ... ...
... ... ... ... ... ... ... ... ... ... ..A ... ... ... ... ... ... ... ... ... ... ...
... ... ... ... ... ... ... ... ... ... ... ... ... ... ... ... ... ... ... ... ... ...
... ... ... ... ... ... ... ... ... ... ... ... ... ... ... ... ... ... A.A ... ... ...
... ... ... ... ... ... ... ... ... ... ... ... ... ... ... ... ... ... ... ... ... ...
... ... ... ... ... ... ... ... ... ... ... ... ... ... ... ... ... ... ... ... ... ...
... ... ... ... ... ... ... ... ... ... ... ... ... ... ... ... ... ... ... ... ... ...
... ... ... ... ..G ... ... ... ... ... ... ... ... ... ... ... ... ... ... ... ... ...
... ... ... ... ... ... ... ... ... ... ... ... ... ... ... ... ... ... ... ... ... ...
... ... ... ... ... ... ... ... ... ... ... ... ... ... ... ... ... ... ... ... ... ...
... ... ... ... ... ... ... ... ... ... ... ... ... ... ... ... ... ... ... ... ... ...
... ... ... ... ... ... ... ... ... ... ... ... ... ... ... ... ... ... ... ... .A. ...
... ... ... ... ... ... ... ... ... ... ... C.. ... ... ... ... ... ... ... ... ... ...
... ... ... ... ... ... ... ... ... ... ... ... ... ..A ... ... ... ... ... ... ... .G.
... ... ... ... ... ... ... ... ... ... ... ... ... ... ... ... ... ... ... ... ... ...
... ... ... ... ... ... ... ... ... A.. ... ... ... ... ... ... ... ... ... ... ... ...
... ... ... ... ... ... ... ..A ... ... ... ... ... ... ... ... ... ... ... G.. ... ...
... ... ... ... ... ... ... ... ... ... ... ... ... ... ... ... ... ... ... ... ... ...
... ... ... ... ... ... ... ... ... ... ... ... ... ... ... ... ..C ... ... ... ... ...
... ... ... ... ... ... ... ... ... ... ... ... ... ... ... ... ... ... ... ..T ... ...
... ... ... ... ... ... ... ... ... ... ... ... ... ... ... ... ... ... ... ... ... ...
... ... ... ... ... ... ... ... ... ... ... ... ... ... ... ... ... ... ... ... ... ...
... ... ... ... ... ... ... ... ... ... ... ... ... ... ... ... ... ... ... ... ..C ...
... ... ... ... ... ... ... ... ... ... ... ... ... ... ... ... ... .G. ... ... ... ...
... ... ... ... ... ... ... ... ... ... ... ... ... ... ... ... ... ... ... ... ... ..G
... ... ... ... ... ... ... ... ... ... ... ... ... ... C.. ... ... ... ... ... ... ...
... ... ... ... ... ..T ... ... ... ... ... ... ... ... ... ... ... ... ... ... ... .A.
... ... ... ... ... ... ... ... ... ... ... ... ... ... ... ... ... ..C ... ... ... ...
... ... ... ... ... ... ... ... ... ... ... ... ... ... ... ... ... ... ... ... ... ...
... ... ... ... ... ... ... ... ... ... ... ... ... ... ... ... ... ... ... ... ... ...
... ... ... ... ... ... ... ... ... ... ... ... ... ... ... ... ... ... ... .G. ... ...
.G. ... ... ... ... T.. ... ... ... ... ... ... ... ..T ... ... ... ... ... ... ... ...
... ... ... ... ... ... ... ... ... ... ... ... ... ... ... ... ... ... ... ... ... ...
... ... ... ... ... ... ... ... ... ... ... ... ... ... ... ... ... ... ... ... ... ...
... ... ... ... ... ... ... ... ... ... ... ... ... ... ... ... ... .C. ... ... ... ...
... ... ... ... ... ... ... ..T ... ... ... ... ... ... ... ... ... ... ... ... G.. ...
... ... ... ... ... ... ... A.. ... ... ... ... ... ... --- ... ... ... ... ... ... ...
... ... ... ... ... ... ... ... ... ... ... ... ... ... .G. ... ... ...
gorilla
... ... ... ... ... ... ... ... ... ... ... ... ... ... ... ... ... ..A ... ... ... ...
... ... ... ... ... ... ... ... ... ... ... ... ... ... ... ... ... ... ... ... ... ...
... ... ... ... ... ... ... ..C ... ... ... ... ... ... ... ... ... ... ... ... ... ...
... .A. ... ... ... ... ... ... ... G.. ... ... ... ... ... ... ... ... ... ... ... ...
... ... ... ... ... ... ... ... ... ... ... ... ... ... ... ... ... ... ... ... ... ...
... ... ... ... ... ... ... ... ... ... ... ... ... ... ... ... A.. ... ... ... ... ...
... ... ... ... ... ... ... ... ... ... ... ... ... ... ... ... ... ... ... ... ... ...
... ... ..G ... ... ... ... ... ... ... ... ... ... ... ... ... ... ... ... ..C ..A ...
... ... ... ... ... ... ... ... ... ..A ... ... ... ... ... ... ... ... ... ... ... ...
... ... ... ... ... ... ... ... ... ... ... ... ... ... ... ... ... ... ... ... ... ...
... ... ... ... ... ... ... ... ... ... ... ... ... ... ... ... ... ... ... ... ... ...
... ... ... ... ... ... ... ... ... ... ... ... ... ... ... ... ... ... ... ... ... ...
... ... ... ... ... ... ... ... ... ... ... ... ... ... ... ... ... ... ... ... ... ...
... ... ... ... ... ... ... ... ... ... ... ... ... ... ... ... ... ... ... ... ... ...
... ... ... ... ... ... ... ... ... ... ... ... ... ... ... ... ... ... ... ... ... ...
... ... ... ... ... ... ... ... ... ... ... ... ... ... ... ... ... ... ... ... ... ...
... ... ... ... ... ... ... ... ... ... ... ... ... G.. A.. ... ... ... ... ... ... ...
... ... ... ... ... ... ... ... ... ... ... ... ... ... ... ... ... ... ... ... ... ...
... ... ... ... ... ... ... ... ... ... ... ... ... ... ... ... ... ... ... ... ... ...
... ... ... ... ... ... ... ... ... ... ... ... ... ... ... ... ... ... ... ... ... ...
... ... ... ... ... ... ... ... ... ... ... ... ... ... ... ... ... ... ... ... ... ...
... ... ... ... ... ... ... ... ... ... ... ... ... ... ... ... ... ... ... ... ... ...
... ... ... ... ... ... ... ... ... ... ... ... ... ... ... ... ... ... ... ... ... ...
... ... ... ... ... ... ... ... ... ... ... ... ... ... ... T.. ... ... ... ... ... ...
... ... ... ... ... ... ... ... ... ... ... ... ... ... ... ... ... ... ... ... ... ...
... ... ... ... ... ... ... ... ... ... ... ... ... ... ... ... ... ... ... ... ... ...
... ... ... ... ... ... ... ... ... ... .T. ... ... ... ... ... ... ... ... ... ... ...
... ... ... ... ... ... ... ..C T.. ... ... ... ... ... ... ... ... ... ... ... ... ...
... ... ... ... ... ... ... ... ... ... ... ... ... ... ... ... G.. ... ... ... ... ...
... ... ... ... ... ... C.. ... ... ... ... ... ... ... ... ... ... ..G ... ... ... ...
... ... ... ... ... ... ... ... ... ... ... ... ... ... ... ... ... ... ... ... ... ...
... ... ... ... ... ... ... ... ... ... ... ... ... ... ... ... ... ... ... ... ... ...
... ... ... ... ... ... ... ... ... ... ... ... ... ... ... ... ... ... ... ... ... ...
... ... ... ... ... ... ... G.. ... ... ... ... ... ... ... ... ... ... ... ... ... ...
... ... ... ... ... ... ... ... ... ... ... ... ... ..A ... ... ... ... ... ... ... ...
... ... ... ... G.. ... ... ..C ... ... ... ... ... ... ... ... ... ... ... ... ... ...
... ... ... ... ... ... ... ... ... ... ... ... ... ... ... ... ... ... ... ... ... ...
... ... ... ... ... ... ... ... ... ... ... ... ... ... ... ... ... A.. ... ... ... ...
... ... ... ... ... ... ... ... ... ..T ... ... ... ... ... ... ... ... ... ... ... ...
... ... ... ... ... ... ... ... ... ... ... ... ... C.. ... ... ... ... ... ... ... ...
... ... ... ... ... ... ... ... ... ... ... ... ..C ... ... ... ... ... ... ... ... ...
... ... ... ... ... ... ... ... .C. ... G.. ... ... ... ... ... ... ... ... ... ... ...
... ... ... ..G ... ... ... ... ... ... ... ... ... ... ... ... ... ... ... ... ... ...
... ... ... ... ... ... ... ... ... ... ... ... ... ... ... ... ... ... ... ... ... ...
... ... ... ... ... ... ... ... ... ... ... ... ... ... ... ... ... ... ... ..C ... ...
... ... ... ... ... ... ... ... ... ... ... ... ... ... ... ... ... ... ... ... C.. ...
... ... ... ... ... ... ... ... ... ... ... .T. ... ... ..T G.. ... ... ... ... .TA ...
... ... ... ... ... ... ... ... ... ... ... ... ... ... ... ... ... ... ... ... ... ...
... C.. ... ... .C. ... ... ... ... ... ... ... ... ... ... ... ... ... ... ... ... ...
... ... ... ... ... ... ... ... ... ... ... ... ... ... ... ... ... ... ... ... ... ...
... ... ... ... ... ... ... ... ... ..G ... ... ... ... ... ... ... ... ... ... ... ...
... ... ... ... ... ... ... ... ... ... ... ... ... ... ... ... .T. .A. ... ... ... ...
... ... ... ... ... ... ... ... ... ... ... ... ... ... ... ... ... ... ... ... ... ...
... ... ... ... ... ... ... ... ... ... ... ... ... ... ... ... ... ... ... ... ... ...
... ... ... ... ... ... ... ... ... ... ... ... ... ... ... G.T ... ... ... ... ... ...
... ... ... ... ... ..A ... ... ... ... ... ... ... ... ... ... ... ... ... ... ... ...
... .C. ... ... ... ... ... ... ... ..C ... ... ... ... ... ... ... ... ... ... ... ...
... ... ... ... ... ... ... ... ... ... ... ... ... ... ... ... ... ... ... ... .C. ...
... ... T.. ... ... ... ... ... ... ... ... ... ... ... ... ... ... ... ... ... ... ...
... ... ... ... ... ... ... ... ... ... ... ... A.. .G. ... ... ... ... ... ... ... ...
... ... ... ... ... ..G ... ... ... ... ... ... ... ... ... ... ... ... ... ... ... ...
... ... ... ... ... ... ... ... ... ... ... ... ... ... ... ... ... ... ... ... ... ...
... ... ... ... ... ... ... ... ... ... ... ... ... ... ... ... ... ... ... ... ... ...
... ... ... ... ... ... ... ... ... ... ... ... ... ... ... ... ... ... ... ... ... ...
... ... ... ... ... ... ... ... ... ... ... ... ... ... ... ... ... ... ... ... ... ...
... ... ... ... ... ... ... ... ... ... ... ... ... ... ... ... ... ... ... ... ... ...
... ... ... ... ... ... ... ... ... ... ... ... ... ... ... ... ... ... ... ... ... ...
... ... ... ... ... ... ... ... ... ... ... ... ... ... ... ... ... ... ... ... ... ...
... .T. ... ... ... ..C ... ... ... ... ... ... ... ... ... ... ... ... ... ... ... ...
... ... ... ... ... ... ... ... ... ... ... ... ... ... ... ... ... ... ... ... ... ...
... ... ... ... ... ... ... ... ... ... ... ... ... ... ... ... ... ... ..A ... ... ...
... ... ... ... ... ... ... ... ... ... ... ... ... ... ... ... ... ... ... ... ... ...
... ... ... ... ... ... ... ... ... ... ... ... ... ... ... ... ... A.. ... ... ... ...
... ... ... ... ... ... ... ... ... ... ... ... ... .G. ... ... ... ... ... ... ... ...
... ... ... ... ... ... ... ... ... ... ... ... ... ... ... ... ... .A. ... ... ... ...
... ... ... ... ... ... ... ... ... ... ... ... ... ... ... ... ... ... .G. ... ... ...
... ... ... ... ... ... ... ... ... ... ... ... ... ... ... ... ... ... A.. ... ..G ...
... ... ... ... ... ... ... ... ... ... ... ... ... ... ... ... ... ... ... ... ... ...
... ... ... ... ... ... ... ... ... ... ... ... ... ... ... ... ... ... ... ... ... ...
... ... ... ... ... ... ... ... ... ... ... ... ... ... ... ... ... ... ... ... ... ...
... ... ... ... ... ... ... ... ... ... ... ... ... ... ... ... ... ... ... ... ... ...
... ... ... ... ... ... ... ... ... .A. ... ... .G. ... ... ... ... ... ... ... ... ...
C.. ... ... ... ... ... ... ... ... ... ... ... ... ... ... ... ... ... ... ... ... ...
... ... ... ... ... ... G.. ... ... ... ... ... ... ... ... ... ... ... ... ... ... ...
... ... ... ... ... ... ... ... ... ... ... ... ... ... ... ... ... ... ... ... ... ...
... ..C ... ... ... ... ... ... ... ... ... ... ... ... ... ... ... ... ... ... ..G ...
... ... ... ... ... ... ... ... ... ... ... ... ... ..T ... ... ... ... ... ... ... ...
... ... ... ... ... ... ... ... ... ... ... ... ... ... ... ... ... ... ... ... ... ...
... ... ... ... ... G.. ... ... ... ... ... ... ... ... ... ... ... ... ... ... ... ...
... ... ... ... ... ... ... ... ... ... ... ... ... ... ... ... ... ... ... ... ... ...
... ... T.. ... ... ... ... ... ... ... ... ... ... ... ... ... ... ... ... ... ... ...
... ... ... ... ... ... ... ... ... ... ... ... ... ... ... ... ... ... ... ... ... ...
... ... ... ... ... ... ... ... ... ... ... ... ... ... ... ... ... ... ... ... ... ...
... ... ... ... ... ... ... ... ... ... ... ... ... ... ... ... ... ... ... ... ... ...
... ... ... ... ... ... ... ... ..C ... ... ... ... ... ... ... ... ... ... ... ... ...
... ..A ... ... ... ... ... ... ... ... ... ... ... ... ... ... ... ... ... ... ... ...
... ... ... ... ... ... ... ... ... ... ... ... ... ... ... ... ... ... ... ... ... ...
... ... ... ... ... ... ... ... ... ... ... ... ... ... ... ... ... ... ... ... ... ...
... ... ... ... ... ... ... ... ... ... ... ... ... ... G.. ... ... ... ... ... ... ...
... ... ... ... ... ... ... ... ... ... ... ... ... ... ... ... ... ... ... ... ... ...
... ... ... ... ... ... ... ... ... ... ... ... ... ... ... ... ... ... ... ... ... ...
... ... ... ... ... ... ... ... ... ... ... ... ... ... ... ... ... ... ... ... ... ...
.A. ... ... ... ... ... ... ... ... ... ... ... ... ... ..A ... ... ... ... ... ... ...
... ... ... ... ... ... ... ... ... ... ... ... ... ... ... ... ... ... ... ... ... ...
... ... ... ... ... ... ... ... ... ... ... ... ... ... ... ... ... ... ... G.. ... ...
... ... ... ... ... ... ... ... ... ... ... ... ... ... ... ... ... ... ... ... ... ...
... ... ... ... ... ... ... ... ... ... ... ... ... ... ... ... ... ... ... ... ... ...
... ... ... ... ... ... ... ... ... ... ... ... ... ... ... ... ... ... ... ... ... ...
... ... ... ... ... ... ... ... ... .A. ... ... ... ... ... ... ... ... ... ... ... ...
... ... ... ... ... ... ... ... ... ... ... ... ... ... ... ... ... ... ... ... ... ...
... ... ... ... ... ... ... ... ... ... ... ... ... ... ... ... ... ... ... ... ..C ...
... .C. ... ... ... ... ... ... ... ... ... ... ... ... ... ... ... .G. ... ... ... ...
... ... ... ... ... ... ..T ... ... ... ... ... ... ... ... ... ... ... ... ... ... ..G
... ... ... ... ... ... ... ... ... ... ... ... ... ... C.. ... ... ..T ... ... ... ...
... ... ... ... ..A ..T ... ... ... ... ... ... ... ... ... ... ... ... ... ... ... .A.
... ... ... ... ... ... ... ... ... ... ... ... ... ... ... ... ... ... ... ... ... ...
... ... ... ... ... ... ... ... ... ... ... ... ... ... ... ... ... ... ..C ... ... ...
... ... ... ... ... ... ... ... ... ... ... ... ... ... ... ... ... ... ... ... ... ...
... ... ... ... ... ... ... ... ... ... ... ... ... ... ... ... .T. ... ... .G. ... ..T
... ... ... ... ... T.. ... ... ... ... ... ... ... ..T ... ... ... ... ... ... ... ...
... ... ... ... ... ... ... ... ... ... ... ..A ... ... ... ... ... ... ... ... ... ...
... ... ... ... ... ... ... ... ... ... ... ... ... ... ... ... ... ... ... ... ... ...
... ... ... ... ... ... ... ... ... ... ... ... ... ... ... ... ... .C. ... ... ... ...
... ... ... ... ... ... ... ..T ... ... ... ... ... ... ... ... ... ... ... ... G.. ...
... ... ... ... ... ... ... ... ... ... ... ... ... ... ... ... ... ... ... ... ... ...
... ... ... ... ... ... ... ... ... ... ... ... ... ... .G. ... ... ...
orangutan
... ... ... ... ... .G. ... ... .G. ... ... ... ... G.. ... ... ... ..A ... ... ... ..T
... ... ... ... ... ... ... ... ... ... ... ... ... ... ... ... ... ... ... ... ... ...
... ... ... ... ... ... ... ..C ... ... ... ... ... ... ... ... ... ... ... ... ... ...
... ... ... ... ... ... ... ... ... G.T ... ... ... ... ... ... ... ... ... ... ... ...
... ... ... ... ... ... ... ... ... ... ... ... ... ... ... ... ... ... ... ... ... ...
... ... ... ... ... ... ... ... ... ... ... ... ... ..A ... ... A.. ... ... ... ... ..C
... ... ... ... ..T ... ... ... ... ..T ... ... ... ... ... ... ... ... ... ... ... ...
... ... ... ... ... ... ... ... ... ... ... ... ... ... ... ... ... ... ... ... ... ...
..C ... ... ... ... ... ... ... ... ... ... ... ... ... ... ... ... ... ... ... ... ...
... ... ... ... ... ... ... ... ... ... ... ... ... ... ... ... ... ... ... ... ... ...
... ... ... ... ... ... ... ... ... ... ... ... ... ... ... ... ... ... ... ... ... ...
... ... ... ... ... ... ... ... ... ... ... ... ... ... ... ... ... ... ... ... ... ...
... ... ... ... ... ... ... ... ... ... ... ... ... ... ... ... ... ... ... ... ... ...
... ... ... C.. ... ... ... ... ... ... ... ... ... ... ... ... ... ... ... ... ..G ...
... ... ... ... ... ... ... ... ... ... ... .A. .A. ... ..A ... ... ... ... ... ... ...
... .T. ... ... ... ... ... ... ... ... ... ... ... ... ... ... ... ... ..T ... ... ...
... ... ... ... ... ... ... ... A.. ... ... ... ... ... A.. ... ... ... ... ... ..G ...
... ... ... ... ... ... ... ... ... ... ... ... ... ... ... ... ... ... ... ... ... ...
... ... ... ... ... ... ... ... ... ... ... ... ... ... ... ... ... ... ... ... ... ...
... ... ... ... .A. ... ... ... ... ... ... ... ... ... ... ... ... ... ... .G. ... ...
... ... ... ... ... ... ... ... ... ... ... ... ... ... ... ... ... ... ... ... ... ...
... ... ... ... ... ... ... ... ... ... ... ... ... ... ... ... ... ... ... ... ... ...
... ... ... ... ... ... ... ... ... ... ... ... ... ... ... ... ... ... ... ... ... ...
... ... ... ... ... ... ... ... ... ... ... ... ... ... ... ... ... ... ... ... ... ...
... ... ... ... ... ... G.. ... ... ... ... ... ... ... ... ... ... ... ... ..G ... ...
... ... ... ... ... ... ... ... .G. ... ... ... ..C ... ..T ... ... ... ... ... ... ...
... ... ... ... ... ... ... ... ... ... ... ... ... ... ... ..T ... ... ... ... ... ...
... ... ... ... ... ... .T. ..G ... ... ... ... ... ... ... ... ... ... ... ... ... ...
... ... ... ... ... ... ... ... ... ... ... ... ... ..G ... ... G.. ... ..C ... ... ...
... ... ... ... ... ... ... ... ... ... ... ... ... ... ... ... ... ..G ... ... ... ...
... ... ... ... ... ... ... ... ... ... ... ... ... ... ... ... ... ... ... ... .G. ...
... ... ... ..C ... ... ... ... ... ... ... ... ... ... ... ... ... ... ... ... ... ...
... ... ... ... ... ... ... ... ... ... ... ... ... ... ... ... ... ... ... ... ... ...
... ..A ... ... ... ... ... G.. ... ... ... C.. ... ... ... ... ... ... ... ... ... ...
... ... ... ... ... ... ... ... ... ... ... ... ... ... ... ..T C.. ... ... ... ... ...
... ... ... ... G.. ... ... ... ... ... ... ... ... .G. ... ... ... ... ... ... ... ...
... ... ... ... ... ... ... ... ... ... ... ... ... ... ... ... ... ... ... ... ... ...
... ... ... ... T.. ... ... ... ... ... ... ..A ... ... ... ... .G. ... ... ... ... ...
... ... ... ... ... ... ... ... ... ... ... ... ... ... ... ... ... ... ... ... ... ...
... ... G.. ... ... ... ... ... ... ... ... ... ... ... ... ... ... ... ... ... ... ...
... ... ... ... ... ... ... ... .T. ... ... ... ..C ..C ... ... ... ... ... ... ... ...
... ... ... ..G ... ... ... ... .C. ... G.. ... ... ... ... ... ... ... ... ... ... ...
... ... ... ..G ... ... ... ... ... ... ... ... ... ... ... ... ..T ... ... ... ... ...
... ... ... ... ... ... .C. ... ... ... ... ... ... ... ... ... ... ... ... ... ... ...
... ... ... ... ... ... ... ... ... ... ... ... ... ... ... ... ... ... ... ..C ... ...
... ... ... ... ... ... ... ... ... ... ... ... ... ... ... ... ... ... ... ... ... .T.
... ... ... ... ... ... ... ... ... ... ... ... ... ... ... ... ... ... ... ... ... ...
... ... ... ... ... ... ... ... ... A.C ... ... ... ... ... ... ... ... ... ... ... ...
... ... ... ... .C. ... ... ... ... ... ... ... ... ... ... ... ... .A. ... ... ... ...
... ... ... ... ... ... ... ... ... ... ... ... ... ... ... ... ... ... ... ... ... ...
... ... ... ... ..T ... ... ... ... ..G ... ... ... ... ... ... ... ... ... ... .AT ...
... ... ... ... ... ... ... ... ... ... ... ... ... .T. ... ... ... .A. ... ... ... ..A
... ... ... ... ... ... ... ... ... ... ... ... ... ... ... ... ... G.. ... ... ... ...
... ... ... ... ... ... ... ... ... ... ... .T. ... ... ..C ... ... ... ... ... ... ...
... ... ... ... ... ... ..T ... ... ... ... ... ..A ... ... G.. ... A.. ... ..A ... ...
... ... ... ..A ... ... ... ... ... ... .T. ... ... ... ... ... ... ... ... ... ... ...
... .C. ..A ... ... .T. ..G G.. ... ..C ... ... ... ... ... ... ... ... ... ... ... C..
... ... ... ... ..G ... ... ... ... ... ... ... ... ... ... ... ... ... ... ... .C. ...
... ... ... ... ... ... ... ... ... ... ... ... ... ... ... ... ... ... ... ... ... ...
... ... ... ... ... ... ... ... ... ... ... ... ..A .G. ... ... ... ... ... ... ... ...
... ... ... ... ... ..G ... ... ... ... ... ... ... ... ... ... ... ... ... ... .C. ...
... ... ... ... ... ... ... ... ... ... ... ... ... ... ... T.. ... ... ... ..G ... ...
... ... ... ... ... ... ... ... ... ... ... ... ... ... ... ... ... ... ... ... ... ...
... ... ... ... ... ... ... ... ... ... ... ... ... ... ... ... ... ... ... ... ... ...
... ... ... ... ..T ... ... ... ... ... ... ..C ... ... ... ... ... ... ... ... ... ...
... ... ... ... ... ... ... ... ... ... ..A ... ... ... ... ... ... ... ... ... ... ...
... ... ..T ... ... ... ... ... ... ... ... ... ... ... ... ... G.. ... ... ... ... ...
... ... ... ... ... ... ... ... ... ... ... ... ... ... ... ... ... ... ... ... ... ...
... .T. ... ... ... ..C ... ... ... ... ... ... ... ... ... ... ... ... ... ... ... ...
... ... ... ... ... ... ... ... ... ... ... .A. ... ... ... .G. ... G.. ... ... ... ..A
... ... ... ... ... ... ... ... ... ... ... ... ... ... ... ... ... ... ..A ... ... ...
... ... ... ... ... ... ... ... ... ... ... .C. ... ... ... ... ... ... ... ..T ... ...
... ... ... ... ... ... ... ... ... ... ... ... ... ... ... ... ... ... ... ... ... ...
... G.. ... ... ... ... ... ... ... ... ... ... ... ..A ... ... ... ... ... ... ... ...
... ... ... ... ... ... ... ... ... ... ... ... ... ... ... ... ... .A. ... ... ... ...
... ... ... ... ... ... ... ... ... ... .A. ... ... ... ... ... ... ... .G. ... ... ...
... ... ... ... ... ... ... ... ... ... ... ... ... ... ... ... ... ... A.. ... ... ...
... ... ... ... ... ... ... ... ... ... ... ... ... ... ... ... ... ... ... ... ... ...
..G ..T ... ... ... ... ... ... ... ... ... ... ... .A. ... ... ... ... ... ... ... ...
... ... ... ..C ... ... ... .AT G.. ... ... ... ... ... ... ... ..T ... ... ... ... ...
... ... ... ... ... ... ... ... ... ... ... C.. ... ... ... ..G ... ... ... ... ... ...
... ... ... ... ... ... ... .C. ... .A. ... ... .G. ... ... ... ... ... ... ... ... ...
C.. ... ... ... ... ... .T. ... ... ... ... ... ... ... ... ... ... ... ... ... ... ...
... ... ... ... ... ... ... ... ... ... ... ... ... ... ... ... ... ... ... ... ... ...
A.. ... ... ... ... ... ... ... ... ... ... ... ... ... ... ... ... ... ... ... ... ...
... ... C.. ... ... ... ... ... ... ... ... ... ... ... ... ... ... ... ... ... ..G ...
... ... ... ... ... ... ... ... ... ... ... ... ... ... ... ... ... ... ... ... ... ...
... ... ... ... ... ... ... ... ... ... ... ... ... ... ... ... ... ... ... ... ... ...
... ... ... ... ... ... ... ... ... ... ... ... ... ... ... ... ... ... ... ... ... ...
... ... ... ... ... ... ... ... ... ... ..A ... ... ... ... T.. ... ... ... ... ... ...
... ... ... ... ..G ... ... ... ... ... ... ... ... ... ... ... ... ... .A. ..T ... ...
... ... ... ... ... ... ... ... ... ... ... ... ... ... ... ... ... ... ... ... ... ...
... ... ... .G. ... ... ... ... ... ... ... ... ... ... ... ... ... ... ... ... ... ...
... ... ... ... ... ... ... ... ... ... ... ... ... ... ... ... ... ... ... ... ... ...
... ... ... ... ... ... ... ... ... .C. ... ... ... ... ... ... ... ... ... ... ... T..
... .T. ... ... ... ... ... ..G ... ..G ... .C. ... ... ... ... ... ... ... ... ... ...
... ... ... ... ... ... ... ... ... ... ... ... ... ... ... ... ... ... ... ... ... ...
... ... ... ... ... ... ... ... ... ... ... ... ... ... ... ... ... ... ... ... ... ...
... ... ... ... ... ... ... ... ... ... ... ... ..C ... ... ... ... ... ... ... ... ...
... ... ... ... ... ... ... ... ... ... ... ... ... ..T ... ... ... ... ... ... ... ...
... ... ... ..T ... ... ... .A. ... ... ... ... ... ... ... ... ..T ... ... ... ... ...
... .G. ... ... ... ... ... ... ... ... ... ... ... ... ... ... ... ... ... ... ... ...
... ... ... ... ... ... ... ... ... ... ... ... ... ... ... ... ... ... ... ... ... ...
... ... ... C.. ... ... .T. A.. ... ... ... ... ... ... ... ... ... ... ... ... ... ...
... ... ... ... ..T ... ... ... ... ... ... ... ... ... ... ... ... ... ..T G.. ... ...
... ... ... ... ... ... ... ... ... ... ... C.. ... ... ... ... ... ... ... ... ... ...
... ... ... ... ... ... ... ..T ... ... ... ... ... ... ..T ... ... ... ... ... ... ...
... ... ... ... ... ... ... ... ... ... ... ... ... ... ... ... ... ... ... ... ... ...
... .A. ... ... ... ... ... ... ... ... ... ... ... ... ... ... ... ... ... ... ... ...
... ... ... ... ... ... ... ... ... ... ... ... ... ... ... ... ... ... ... ... ... ...
... ... ... ... ... ... ... ... ... ... ... ... ... ... ... ... ... ..A ... ... ..C ...
... ... ... ... ... ... ... ... ... ... ... ... ... ... ... ... ... ... ... ... ... ...
... ... ... ... ... ... ... ... ... ... ... ... ... ... ... ... ... ... ... ... ... ..G
... ... ... ... ... ... ... ... ... ... ... ... ... ... ... T.. ... ... ... ... ... ...
... ... ... ... ... ... ... ... ... ... ... ... ... ... ... ... ... ... ... ... ... .A.
... ... ... ... ... ... ... ... ... ... ... ..G ... ... ... ..C ... ... ... ... ... ...
... ... ... ... ... ... ... ... ... ... ... ... ... ... ... ... ... ... ... ... ..C ...
... ... ... ... ... ... ... ... ... ... ... ... ... ... ... ... ... ... ... ... ... ...
... ... ... ... ... ... ... ... ... ... ..T ... ... ... ... ... ... ... ... .G. ..T ...
.G. ... ... ... ... T.. ... ... ... ... ... ... ... ..T ... ... ... ... ... ... ... ...
... ... ... ... ... ... ... ... ... ... ... ... ... ... ... ... ... ... ... ... ... ...
... ... ..G ... ... ... ... ... ... ... ... ... ... ... ... ... ... ... ... ... ... ...
... ... ... ... ... ... ... ... ..C ... ... ... ... ..A ... ... ... .CA ... ... ... ...
... ... ... ... ... ... ... ..T ... ... ... ... ... ... ... ... ..A ... ... ... G.. ...
... ... ... ... ... ... ... ... ... ... ... ... ... ... ... ... ... ..A ... ... ... ...
... ... ... ... ... ... ... ... ... ... ... ... ... ... .G. ... ... ...
gibbon
... ... ... ... ... .G. ... ... .G. ... ... ... ... ... ... ... ... ..A ... ... ... ..T
... ... ... ... ... ... ... ... ... ... ... ... ... ... ... ... ... .T. ... ... ... ...
... ... ... ... ... ... ... ..C ... ... ... ... ... ... ... ... ... ... ... ... ... ...
... ... ... ... ... ... ... ... ... G.. ... ... ... ... ... ... ... ... ... ... ... ...
... ... ... ... ... ... ... ... ... ... ... ... ... ... ... ... ... ... ... ... ... ...
... ... ... ... ... ... ... ... ... ... ... ... ... ... ... ... A.. ... ... ... ... ..C
... ... ... ... ... ... ... ... ... ... ... ... ... ... ... ... ... ... ... ... ... ...
... ... ... ... ... ... ... ... ... ... ... ... ... ... ... ... ... ... ... ... ... ...
..C ... ... ... ... ... ... ... ..A ..A ... ... ... ... ... ... ... ... ... ... ... ...
... ... ... ... ... ... ... ... ... ... ... ... ... ... ... ... ... ... ... ... ... ...
... ... ... ... ... ... ... ... ... ..A ... ... ... ... ... ... ... ... ... ... ... ...
... ... ... ... ... ... ... ... ... ... ... ... ... ... ... ... ... ... ... ... ... ...
... ... ... ... ... ... ... ... ... ... ... ... ... ... ... ... ... ... ... ... ... ...
... ... ..C ... ... ... ... ... ... ... ... ... ... ... ... ... ... ... ... ... ... ...
... ... ... ... ... ... ... ... ... ... ... ... ... ... ..A ... ... ... ... ... ... ...
... ... ... ... ... ... ... ... ... ... ... ... ... ... ... ... ... ... ... ... ... ...
... ... ... ... ... ... ... ... ... ... ... ... ... ... A.. ... ... ... ... ... ... ...
... ... ... ... ... ... ... ... ... ... ... ... ... ... ... ... ... ... ... ... ... ...
... ... ... ... ... ... ... .C. ... ... ... ... ... ... ... ... ... ... ... ... ... ...
... ... ... ... ... ... ... ... ... ... ... ... ... .G. ... ... ... ... ... .G. ... ...
... ... ... ... ... ... ... ... ... ... ... ... ... ... ... ... ... ... ..A ... ... ...
... ... ... ... ... ... ... ... ... ... ... ... ... ... ... ... ... ... ... ... ... ...
... ... ... ... ... ... ... ... ... ... ... ... ... ... ... ... ... ... ... ... ... ...
... ... ... ... ... ... ... ... ... ... ... ... ... ... ... ... ... ... ... ... .G. ...
... ... ..T ... ... ... ... ... ... ... ... ... ... ... ... G.. ... ... ... ... ... ...
... ... ... ... ... ... ... ... ... ... ... ... ... ... ... ... ... ... ... ... ... ...
... ... ... ... ... ... ... ... ... ... ... ... G.. ... ... ... ... ... ... ..C ... ...
... ... ..C ... ... ... ... ..G ..T ... ... ... ... ... ... ..A ... ... ... ... ... ...
... ... ... ... ... ... ... ... ... ... ... ... ... ... ... ... G.G ... ... ... ... ...
... G.. ... ... ... ... ... ... ... ... ... ... ... ... ... ... ... ..G ... ... ... ...
... ... ... ... ... ... ... ... ... ... ... ... ... ... ... ... ... ... ... ... .G. ...
... ... ... ... ... ... ... ... ... ... ..T ... ... ... ... ... ..T ... ... ... ... ...
... ... ... ... ... ... ... ... ... ... ... ... ... ... ... ... ... ... ... ... ... ...
... ..A ... ... ... ... ... G.. ... ... ... ... ... ... ... ... ... ... ... ... ... ...
... ... .G. ... ... ... ... ... ... ... ... ... ... ... ... ... ... ... ... ... ... ...
... ... ... ... G.. ... ..C ..C ... ... ... C.T ... .G. ... ... ... ... ... ... ... ...
... ... ... C.G ... ... ... ... ... ... ... ..A ... ... ... ... ... ... ... ... ... ..T
... ... ... ... ... ... ... ... ... ... ... ... ..C ... ... ... .G. ... ... ... ... ...
... ... ... ... ... ... ... ... ... ... ... ... ... ... ... ... ..G ... ... ... ... ...
... ... G.. ... ... ... ... ... ... ... ... ... ... C.. ... ... ... ... ... .G. ... ...
... ... ... ... ... ... ... ... ... ... ... ... ..C ..C ... ... ... ... ... ... ... ...
... ... ... ... ..G ... ... ... .C. ... G.. ... ... ... ... ... ... ... ... ... ... ...
... ... ... ... ... ..T ... ... ... ... ... ... ... ... ... ... ... ... ... ... ... ...
... ... ... ... ... ... ... ... ... ... ... ... ... ... ... ... ... ... ... ... ... ...
... ... ... ... ... ... .C. ... ... ... ... ... ... ... ... ... ... ... ... ..C ... ...
... ... ... ... ... ... ... ... ... ... ... ... ... ... ... ... G.. ... ... ... ... .T.
... ... ... ... ... ... ... ... ... ... ... ... ... ... ... G.. ... ... ... ... ... ..C
... ... ... ... ... ... ... ... ..T ... ... ... ... ... ... ... ... ... ... ... ... ..T
... ... ... ... .C. ... ... ... ... ... ... ... ... ... ... ... ... ... ... ... ... ...
... ... ... ... ... ..C ... ... ... ..C ... ... ... ... ... ... ... ... ... ... ... ...
... ..T ... ... ... ... ... ... ... ..G ... ... ... ... ... ... ... ... ... ... .A. ...
... ... ... ... ... ... ... ... ... ... ... ... ... .T. ... ... ... .A. ... ... ... ...
... T.. ... ... ... ..C ... ... ... ... ... ... ... ... ... ... ... ... G.. ... ... ...
... ... ... ... ... ... ... ... ... ... ... ... ... ... ... ... ... ... ... .A. ... ...
... ... ... ... ... ..T ... ... ... ... ... ... ... ... ... G.. ... A.. ... ..T ... ...
... ... ... ... ... ... ... ..G ... ... ... ... ... ... ... ... ... ... ... ... ... ...
... .C. ... ... ... ... ..G G.. ... ..C ... ... ... ... ... ... ... ... ... ... ... C..
..T .G. ... ... ..G ... ... ... ... ... ... ... ... ... A.. ... ... ... ... ... .CA ...
... ... ... ... ... ... ... ... ... ... ... ... ... ... ... ... ... ... ... ... ... ...
... ... ... ..A ... ... ... ... ... AT. ... ... ... .G. ... ... ... G.. ... ... ... ...
... ... ... ... ... .TG ... ... ... ... ... ... ... ... ... ... ... ... ... ... .C. ...
... ... ... ... ... ... ... ... ... ... ... ... ... ... ... ... ... ... ... ... ... ...
... ... ... ... ... ... .A. ... ... ... ... ... ... ... ... ... ... ... ... ... ... ...
... .C. ... ... ... ... ... ... ... ... ... ... ... ... ... ... ... ... ... ... ... ...
... ... ... ... ..T ... ... ... ... ... ... ..C ... ... ... ... ... ... ... ... ... ...
... ... ... ... ... ... ... ... ... ... ... ... ... ... ... ... ... ... ... ... ... ...
... ... ... ... ... ... ... ... ... ... ... ... ... ... ... ... ... ..G ... ... ... ...
... ... ... ... ... ... ... ... ... ... ... ... ... ... ... ... ... ... ... ... ... ...
... .T. ... ... ... ... ... ... ... ... ... ... ... ... ... ... ... ... ... ... ... ...
... ... ... ... ... ... ... ... ... ... ... ... ... ... ... ... ... G.. ... ... ... ...
... ... ... ... ... ... ... ... .A. ... ... ... ... ... ... ... ... ... ..A ... ... ...
... ... ... ... ... ... ... ... ... ... ... .C. ... ... ... ... ... ... ... A.T ... .G.
... ... --- --- ... ... ... ... ... ... ... ... ... ... ... ... ... ... ... ... ... ...
... G.. ... ... ... ... ... ... ... ... ..T ... ... ..A ... ... ... ... ... ... ... ...
... ... ... ... ... ... ... ... ... ... ... ... ... ... ... ... ... .A. ... ... ... ...
... ... ... ... ... ... ... ... ... ... ... ... ... ... ... ... ... ... .G. ... ... ...
... ... ... ... ... ... ... ... ... ... ... ... ... ... ... ... .G. ... A.. ... ... ...
... ... ... ... ... ... ... ... ... ... ... ... ... ... ... ... ... ... ... ... ... ...
..G ... ... ... ... ... ... ... ... ... ... ... ... ... ... ... ... ... ... ... ... ...
... ... ... ..C ... ... ... ... ..T G.. ... ... ... ... ... ... ... ... ... ... ... ...
... .CT ... ... ... ... ... ... ... ... ... C.. ... ... ... ..G ... ... ... ... ... ...
... ... ... ... ... ... ... .C. ... ... ... ... .G. ... ... ... ... ... ... ... ... ...
C.. ... ... ... ... ... ... ... ... ... ... ... ... ... ... ... ... ... ... ... ... ...
... ... ... ... ... ... ... ... ... ... ... ... ... ... T.. ..A ... ... ... ... ... ...
A.. ... ... ... ... ... ... ... ... ... ... ... ..T G.. ... ... ... ... ... ... ... ...
... ... C.. ... ... ... ... ... ... ... ... ... ... ... ... ... ... ... ... ... ..G ...
... ... ... ... ... ... ... ... ... ... ... ... ... ..T ..G ... ... ... ... G.. ... ...
... ... ... ... ... ... ... ... ... ... ... ... ... ... ... ... ... ... ... ... ... ...
... ... ... ... ... ... ... ... ... .GA ... ... ... ... ... ... ... ... ... ... ... ...
... ... ... ... ... ... ..T ... ... ... ..A ... ... ... ... ... ... ... ... ... ... ...
... ... ... ... ... ... ... ... ... ... ... ... ... ... ... ... ... ... ... ..T ... ...
... ... ... ... ... ... ... ... ... ... ... ... ... ... ... ... ... ... ... ... ... ...
... ... ... ... ... ..A ... ... ... ... ... ... ... ... .G. ... ... ... ... ... ... ...
... ... ... ... .G. ... ... ... ... ... ... ..C ... ... ... ... ... ... ... ... ... ...
... ... ... ... ... ... ... ... ... .C. ... ... ... ... ... G.. ... ... ... ... ... ...
... ..A ... ... ... ..A ... ... ... ... ... ... ... ... ... ... ... ... ... ... ... ...
... ... ... ... .T. ... ... ... ... ... ... ... ... ..T ... ... ... ... ... ... ... ...
... ... ... ... ... ... ... ... ... ... ... ... ... ... ... ... ... ... ... ... ... ...
... ... ... ... ... ... ... ... ... ... ... ... ..C ... ... ... .A. ... ... ... ... ...
... ... ... ... ... ... ... ... ... ... ... ... ... ... ... ... ... ... ... ... ... ...
... ... ... ... ... ... ... .A. ... ... ... ... ... ... ... ... .G. ... ... ... ... ...
... ... ... ... ... ... ... ... ... ... ... ... ... ... ... ... ... ... ... ... ... .G.
... ... ... ... ... ... ... ... ... ... ... ... ... ... ... ... ... ... ... ... ... ...
... ... ... C.. ... ... ... ... ... ... ... ... ... ... ... ... ... ... ... ... ... ...
... ... ... ... ... ... ... AT. ... ... ... ... ... ... ... ... ... .A. ..T G.. ... ...
... ... ... ... ... ... ... ... ... ... ... C.. ... ... ... ... ... ... ... ... ... ...
..T G.. ... ... ... ... ... ..T ... ... ... ... ... ..A ... ... ... ... ... ... ... ...
... ... ... ... ... ... ... ... ... ... ... ... ... ... ... ... ... ... ... ... ... ...
... G.. ... ..A ... ... ... ... ... ... ... ... ... ... ... ... ... ... ... ... T.. ..A
... ... ... ... ... ... ... ... ... ... ... ... ... ... ... ... ... ... ... ... ... ...
... ... ... ... ... ... ... ... ... ... ... ... ... ... ... ... ... ... ... ... ..C ...
... ... ... ... ... ... ... ... ... ... ... ... ... ... ... ... ... ... ... ... ... ..C
... ... ... ... ... ... ... ... ... ... ... ... ... ... ... ... ... ... ... ... ... ..G
... ... ... ... ... ... ... ... ... ... ... ... ... ... C.. ... ... ... ... ... ... ...
... ... ... ... ... ..T ... ... ... ... ... ... ... ... ... ... ... ... ... ... ... .A.
..T ... ... ... ... ... ... ... ... ... ..A ..G ... ... ... ..C ... ... ... ... ... ...
... ... ... ... ... ... ... ... ... ... ... ... ... ... ... ... ... ... ..C ... ..C ...
... ... ... ... ... ... ... ... ... ... ... ... ... ... ... ... ... ... ... ... ... ...
... ... ... ... ... ... ... ... ... ... ... ... ... ... ... ..C ... ... ... .G. ..T ...
.G. ... ... ... ... T.. ... ..A ... ... ... ... ... ..T ... ... ... ... ... ... ... ...
... ... ... ... ... ... ... ... ... ... ... ... ... ... ... ... ... ... ... ... ... ...
... ... ..G ... ... ... ... ... ... ... ... ... ... ... ... ... ... ... ... ... ... ...
... ... ... ... ... ... .A. ... ... ... ... ... ... ... ..A ... ... .C. ... ... ... ...
... ... ... ... ... ... ... ..T ... ... ... ... ... ... ... ... ... ... ... ... G.C ...
... ... ... ... ... ..A ... ... ... ... ... ... C.. ... .G. ... ... ... ... ... ... ...
... ... ... ... ..T ... ... ... ... ... ... ... ... ... .G. ... ... ...
rhesus
... ... ... ... ... ... ... ... .G. ... ... ... ... G.. ... ... ... ... ... ... ... ..T
... ... ... ... ... ... ... ... ... ... ... ... ... ... ... ... ... .G. ... ... ... ...
... ... ... ... ... ... ... ..C ... ... ..C ... ... ... ... C.. ... ... ... ... ... ...
... ... ... ... ... ... ... ... ... G.T ... ..C ... ... ... ... ... ... ... ... ..C ...
... ... ... ... ... ... ... ... ... ... ... ... ... ... ... ..G ... ... ... ... ... ...
... ... ... ... ... ... ... ... ... ... ... ... ... ... ... ... AT. ... ..C ... ... ..C
... ... ... ... ... ... ... ... ... ..T ... ... ... ... ... ... ..C ... ... ... ... .C.
... ... ... ... ... ... ... ... ... ... ... ... ... ... ... ... ... ... ... ... ... ...
..C ... ... ... ... ... ... ..A ..A ... ... ... ... ... ... ... ... ... ... ... ... ...
... ... ... ... ... ... ... ... ... ... ... ... ... ... ... ... ... ... ... ... ... ...
... .A. ... ... ... ... ..A ... ... ... ..G ..C ... ..C ... ... ... ... ... ... ... ...
... ... ... ... ... ... ... ... ... ... ... ... ... ... ... ... ... ... ... ... ... ..T
... ... ... ... ... ... ... ..C ... ... ... ... ... ... ... ... ... ... ... ... ... ...
... ... ... ... ... ... ... ... ... ... ... ... ... ... ... ... ... ... ... ... ... ...
... ... ... ... ... ... ... ... ... ... ... ... ... ..C ..A ... ... ... ... ..A ... ...
... ... ... ... ... ... ... ..C ... ..T .T. ... ... ... ... ..C ... ... ... ... ... ...
... ... ... ... ... ... ... ... ... AG. ... ... ... ... A.. ... ... ... ... ... ... ...
... ... ... ... ... ... ... ... ... ... ... ... ... ... ... ... ... ..G ... ... ... ...
... ... ..G ... ... ... ... ... ... ... ... ... ... ... ..A ... ..A ... ... ... ... ...
... ... ..C ... ... ... ... ... ..A ... ... ... ... ... ... ... ... ... ..G .G. ... ...
... ... ... ... ... ..G ... ... ... ... ... ... ... ... ... ... ... ... ... ... ... ...
... ... ... ... ... ... ... ... ... ... ... ... ... ... ... ... ... C.. ... ... ... ...
... ... ... ... ... ... ... ... ... ... ... ... ... ... ... ... ... ... ... ... ... ...
... ... ... .C. ... ... ... ... .T. ... ... ... ... ... ... G.. ..G ... ... ... ... ...
... ... ..T ... ... ... ... ... ..C ... ... ... ... .T. G.. ... ... ... ... .T. ... ...
G.. ... ... ... ... ... ... ... ..G ... ... ... ... ... ... ... ... ... ... ... ... ...
... ... ... ... ... ... ... ... ... ... ... ... ... ... ... ... ... ... ... ... ... ...
... ... ... ... ... ... ... ..G ... ..G ... ... ... ... ..A ... ... ... ... ... ..G ...
... ... ... ... ... ... ... ... ... ... ..A ... ... ..G ... ... G.. ... ... ... ... ...
... G.. ... ... ... ... ... ... ..C A.. ... ... ..G ... ... ... ... ..G ... ... ... ...
... ... ... ... ... ... ... ... ... ... ... ... ... ... ... ... ... ... ... ... .G. ...
... ... ... ... ... ... ... ... ... T.. ... ... ... ... ... ... ... ... ... ... ..T ...
... ... ... ... ... ... ... ... ... ... ... ... ... ... ... ..G ... ... ... ... ... ...
... ..A ... ... ... ... ... G.. ... ... ... ... ... ... ... ... ... ... ... ... C.. ...
... ... ... ... ... ... ... ... ... ... ... ... ... ... ... ..T G.. ... ..A ... ... ...
... ... ... ... G.. ... ..C ..C ... ... ... ..T ... .G. ... ... ... ... ... ... ... ...
... ... ... ..G ... .T. ... ... G.. ... ... ... ... ... ... ... ... ... ... ... ... ...
... ... ..T G.. ... ... ... ..G ... ... ... ... ... ... ... ... .G. ... ... ... ... ...
..C ... ... ... ... ... ... ... ... ... ... ... ... ... ... ... ..C ... T.. ... T.. ...
... ... G.. ... ... ..T ... ... ... ... ... ... ... C.. ..C ... ... ... ... .G. ... ..A
... C.. ... ... ... ... ... ... ... ... ... ... ..C ..C ... ... ... ... ... ... ... ...
... ... ... ... ..G ... ... ... .C. ... G.. ... ..G ... ... ... ... ... ... ... ... ...
... ... ... ..G ... ... .T. ... ... ... ... ... ... ... ... ... ... ... ..C ... ... ...
... ... ... ... ... ... ... ... ... ... ... ... ... ... ... ... ... ... ... ... ... ...
... ... ... ... ... ..A ... ... ... ... ... ... ... C.G ... ... ... ... ... ..C ... ...
... ..T ... ... ... ... ... ... ... ... ... ... ... ... ... ... ... ... ... ... ... .T.
... ... ... ... ... ... ... ... ... ... .A. .T. ... ... ... G.. ... ..A ... ... ... ..C
... ... ... ... ... ... ... ... ... A.. ... ... ... ... ... ... ... ... ... ... ... ...
..A ... ... ... .C. ... ... ... ... ... ... ... ... ... ... ... ... ... ... ... ... ...
... ... ... ... ... ... T.. ... ... ..C ... ... ... ... ... ... ... .G. ... ... ... ...
... ... ... ... A.. ... ... ... ... ..G ... ... ... ... ... ... ... ... ... ..A .A. ...
... ... ... ..C ..A ... ... ... ... ... ... ... ... .T. ... ... ... .A. ... ... ... ...
... ... ... ... ... ... ... ... ... ... ... ... ... ... ... ... ..T ... T.. ... ... ...
... ... ... ... .A. ... ... ... ... ... ... ... ... ... ..C ... ... ... ... ... ... ...
... ... ... .C. ... ..T ... ... ... ... ... .A. ... ..T ... G.. ... A.. ... ... ... ...
... ... ... ... ... ... ... ..G ... ... A.. CT. ... ... ... ... ... ... ... ... ... ...
... GC. ... ... ... ... ..G G.. ... ..C ... ... ... ... ... ... ... ... ... ... ... C..
... ... ... ... AGG ... ... ... ... ... ... ... ... ... A.. ... ... ... ... ... ... ...
... ... ... ... ... ... ... ... ... ... ... ... ... ... ... ... ... ... ... ... ... ...
... ... ... ... ... ... ... ... ... ... ... ..T ... .G. ... ... ... G.C ... ... ... ...
... ... ... ... ... ..T ... ... ... ... ... ... ... ... ... ... ... ... ... ... ... ...
... G.. ... ... ... G.. ... ... ... ... ... ... ..A ... ... ... ... ... TA. ... ... ...
... ... ... ... ... ... ... ... ... ... ... ... ... ..C ... ... ... ... ... ... ... ...
... ... ... ... ... ... ... ... ... ... ... ... ... ... ... ... ... ... ... ... ... ...
... ... ... ... ..T ... ... ..A ... ... ..A ..C ... ... ... ... ... ... ... ... ... ...
... ... ... ... ... ... ... ... ... ... .T. ... ... ... ... ... ... ... ... ... ... ...
... ... ... ... ... ... ..T ... ... ... ... ... ... ..T ... ... ... ... ... ... ... ...
... ... ..A ..C ... ... ... ... ... ... ..G ... ... ... ... ... ... ... ... ... .CA ...
... .T. ... ... ... ..C ... ... ... ... ... ... ... ... ... ... ... ... ... ... ..T ...
... ... ... ..G ... ..G G.. ..C ... G.. ..C ... ... ... ... .G. ... ... ... ... ... ...
... ... ... ... ..T ... ... ... T.. ... ... ... ... .C. ... ... ... ... ..A ... ..A ...
... ... ... ... ... ... ... ... ... ... ... .C. G.. ... ... ... ... ... ... ..T ... .G.
... ... ... ... ... ... ... ... ... ... ... ... ... ... ..T ... ... ... ... ... ... ...
... G.. ... ... ... ... ... ... ... C.. ..T ..T ... .T. ..A ... ... ... ... ... ... ...
..T ... ... ... ... ... ... ... ... ... ... ... ... ... ... ... ... .A. ... ... ... ...
... ... ... ... ... ... ... ... ... ... ... ... ... ..C ... ... ... ... .G. ... ... ...
... ... ... ... ... ... ... ... ... ... ... ... ... ... ... ... ... ... A.. ... ... ...
... ... ... ... ... ... ... ..C ... ... ... ... ... ... ... ... ... ... ... ... ... ...
..G ... ... ... ... ... ... ... ... ... ... ..T ... ... ... ... ... ... ... ... ... ...
... ... ... ..C ... ... .T. ... ... ... ... ... ... ... ... ... ... .A. ... ... ... ..C
... ... ... ... ... ... ... ... ... ... ... C.. ... ... ... ..G ... ... ... ... ... ...
... ... ... ... .G. ... ... .C. ... .A. ... ... .G. ... ... ... ... .G. ... ... ... ...
C.. ... ... ... ... ... ... ... ... ... ... ... ... ... ... ... ... ... ... ... ... ...
... ... ... ..C ... ... ... ... ... ... ... ... ... ... T.. ..A ... ... ... ... ... ..G
A.. ... ... ... ..G ..T ... ... ... ... ... ... ..T G.. ... ... ... ... ... ... ... .G.
... ..G C.T ... ... ... ... .T. ... ... ... ... T.. ... ... ... ... ... ... ... ..G ...
... ... ... ... ... ... ... ... ... ..C ... ... ... T.T ... ..A ... ... ... ... ... ...
... ... ... ... ... ... ... ... ... ... ... ... ... ... ... ... ... T.. ... ... ... ...
... ..A ... ... ... ... ... ..C ... .G. ... ... ... ... ... .A. ... ... ... ... ... ..G
... ... ... ... ... ... ... ..C ... ... ... ... ... ... ... ... ... ... ... ... ... ...
... ... ... ... ... ... ... ... ... ... ... ... ... ... ... ... ... ... ... ..T ... ...
... ... ... ... ... ... ... ... ... ... ... ... ... ... ... ... ... ... ... ... ..C ...
... ... ... ... ... C.. ... C.. ... ... ... ... ... ... ... ... ... ... ... ... ... ...
... ... ... ... ... ... ... ... ... ... ... ... ... ..T ... ... ... ... ... ... ... ...
... ... .G. ... ... ... ... ... ... .C. ... ... ... ... ... ... ... ... ... ..C ... ...
... ... ... ... ... ... ... ... ... ... ... ... ... ... ... ... ... ... ... ... ... ...
... ... ... ... ... ... ... ... ... ... ... ... ... ... ... ... ... ... ... ... ... ...
... ... ... ... .A. ... ... ... ... ... ... ..G ... ... ... ... ... ... ... ... ... ...
... ... ... ... ..C ... ... ... T.. ... ... ... ..C ... ... ... ... ... ... ... ... ...
... ... ... .G. ... ... ... ... ... ... ... ... ... ... ... ... ... ... ... ... ... ...
... .T. ... GT. ... ... ... ..C ... ... ... ... ... ... ... ..T ... C.. ..C ... ... ...
... ... ... ... ... ... ... ... ... ... ... ... ... ... ... ... ... ... ... ... ... .G.
... ... ... ... ... ... ... ... ... ... ... ... ... ... ... ... ... ... ... ... ... ...
... ... ... C.. ... ... ... ... ... ... ... ... ... ..C ... ... ... ... ..C ... ... ...
... ... ... ... ... ..A ..T ..A ..C ... ... ... ... ... ... ... ... ... ... ... ... .G.
... ... ... ... ... ... ..T ... ... ... ... C.. ... ... ... ..G ... ... ... ... ... ...
... ... ... ... ... ... ... ... ... ... ... ... ... ... ... A.. ... ... ... ... ... ...
... ... ... ... ... ... ... ... ... ... ... ... ... ... ... ... ... ... ... ... ... ...
... ... ..T ... ... ... ... ... ... ... .CT ... ... ... ... ... ... ... ... ... ... .T.
... ... ... ... ... ... ... ... ... ... ... ... ... ... ... ... ... ..T ... ... ... ..A
..T ... A.. ... ... ..C ... ... ... ... ... ... ... G.. ... ... ... ... ... ... ..C ...
..T G.. ... ... ... ... ... ... ... ... ... ... ... ..A ... ... ... ... A.. ... ... ..C
... ... ... ... ... ... ... ... ... ... ... ... ... ... ... ... ... ... ... ... ... ..G
... ... ... ... ... ... ... ... ... ... ... ... ... ... C.. .C. ... ... ... ... ... ..C
... ... ... ... ... ... ... ... ... ... ... ... ... ... ... ... ... ... ... ... ... .A.
... ... ... ... ... ... ... ... ... ... ... ... ... ... ... ..C ... ..C ..T ... ... ...
... ... ... ... ... ... ... ... ..G ... ... ... ... ... ... ... ... ... ..C ... ..C ...
... ... ... ... ..A ... ... ... ... ... ... ... ... ... ... ... ... ... ... ... ... ...
... ... ... ... ... ... ... ..A ... ... ..T ... ... ... ... ... ... ..C ... .G. ... ...
.G. ... ... ... ... T.. ... ... ... ... ... ... ... ..T ... ... ... ... ... ... ... ...
... ... ... ... ... ... ... ... ... ... ... ... ... ... ... ... ... ... ... ... ... ...
... ... ... ... ... ... ... ... ... ... ... ... ... ... ... ... ... ... ... ... ... ...
... ... ... ... ... ... TA. ... ..C ..T ... ... ... ... ... ... ... .CA ... ... ... ...
... ... ... ... ... ... ... ..T ... ... ... ... ... ... ... ... ..A ... ... ... G.. ...
... ..C ... ... ... ..T ... ... ..G ... ..A ... ... ..A ..A ... ... ... ... ... ... ..A
..G ... ... ... ... ... ... ... ... ... ... ... ... ... .G. ... ... ...
```

---

**3. Tree file "tg.tree"**

```
((((human, chimpanzee), gorilla), orangutan), gibbon, rhesus);
```

**4. Tree file "tg-human.tree"**

```
((((human #1, chimpanzee), gorilla), orangutan), gibbon, rhesus);
```

---

**5. Control file for "M0"**

```
      seqfile = tg.phy
     treefile = tg.tree
      outfile = tg-M0-one-ratio.mlc

        noisy = 9  * 0,1,2,3,9: how much rubbish on the screen
      verbose = 0  * 0: concise; 1: detailed, 2: too much
      runmode = 0  * 0: user tree;  1: semi-automatic;  2: automatic
                   * 3: StepwiseAddition; (4,5):PerturbationNNI; -2: pairwise

      seqtype = 1  * 1:codons; 2:AAs; 3:codons-->AAs
    CodonFreq = 2  * 0:1/61 each, 1:F1X4, 2:F3X4, 3:codon table
        clock = 0  * 0:no clock, 1:clock; 2:local clock; 3:CombinedAnalysis
        model = 0
                   * models for codons:
                       * 0:one, 1:b, 2:2 or more dN/dS ratios for branches

      NSsites = 0  * 0:one w; 1:neutral; 2:selection; 3:discrete; 4:freqs;
                   * 5:gamma; 6:2gamma; 7:beta; 8:beta&w 9:betaγ
                   * 10:betaγ+1; 11:beta&normal>1; 12:0&2normal>1;
                   * 13:3normal>0
        icode = 0  * 0:universal code; 1:mammalian mt; 2-10:see below

    fix_kappa = 0  * 1: kappa fixed, 0: kappa to be estimated
        kappa = 2  * initial or fixed kappa
    fix_omega = 0  * 1: omega or omega_1 fixed, 0: estimate 
        omega = 1  * initial or fixed omega, for codons or codon-based AAs

    fix_alpha = 1  * 0: estimate gamma shape parameter; 1: fix it at alpha
        alpha = .0 * initial or fixed alpha, 0:infinity (constant rate)
       Malpha = 0  * different alphas for genes
        ncatG = 4  * # of categories in dG of NSsites models

        getSE = 0  * 0: don't want them, 1: want S.E.s of estimates
 RateAncestor = 0  * (0,1,2): rates (alpha>0) or ancestral states (1 or 2)
       method = 0  * 0: simultaneous; 1: one branch at a time
```

---

**6. Control file for "Free ratio"**

```
      seqfile = tg.phy
     treefile = tg.tree
      outfile = tg-free-ratio.mlc

        noisy = 9  * 0,1,2,3,9: how much rubbish on the screen
      verbose = 0  * 0: concise; 1: detailed, 2: too much
      runmode = 0  * 0: user tree;  1: semi-automatic;  2: automatic
                   * 3: StepwiseAddition; (4,5):PerturbationNNI; -2: pairwise

      seqtype = 1  * 1:codons; 2:AAs; 3:codons-->AAs
    CodonFreq = 2  * 0:1/61 each, 1:F1X4, 2:F3X4, 3:codon table
        clock = 0  * 0:no clock, 1:clock; 2:local clock; 3:CombinedAnalysis
        model = 1
                   * models for codons:
                       * 0:one, 1:b, 2:2 or more dN/dS ratios for branches

      NSsites = 0  * 0:one w; 1:neutral; 2:selection; 3:discrete; 4:freqs;
                   * 5:gamma; 6:2gamma; 7:beta; 8:beta&w 9:betaγ
                   * 10:betaγ+1; 11:beta&normal>1; 12:0&2normal>1;
                   * 13:3normal>0
        icode = 0  * 0:universal code; 1:mammalian mt; 2-10:see below

    fix_kappa = 0  * 1: kappa fixed, 0: kappa to be estimated
        kappa = 2  * initial or fixed kappa
    fix_omega = 0  * 1: omega or omega_1 fixed, 0: estimate 
        omega = 1  * initial or fixed omega, for codons or codon-based AAs

    fix_alpha = 1  * 0: estimate gamma shape parameter; 1: fix it at alpha
        alpha = .0 * initial or fixed alpha, 0:infinity (constant rate)
       Malpha = 0  * different alphas for genes
        ncatG = 4  * # of categories in dG of NSsites models

        getSE = 0  * 0: don't want them, 1: want S.E.s of estimates
 RateAncestor = 0  * (0,1,2): rates (alpha>0) or ancestral states (1 or 2)
       method = 0  * 0: simultaneous; 1: one branch at a time
```

---

**7. Control file for "Two ratio"**

```
      seqfile = tg.phy
     treefile = tg-human.tree
      outfile = tg-two-ratio.mlc

        noisy = 9  * 0,1,2,3,9: how much rubbish on the screen
      verbose = 0  * 0: concise; 1: detailed, 2: too much
      runmode = 0  * 0: user tree;  1: semi-automatic;  2: automatic
                   * 3: StepwiseAddition; (4,5):PerturbationNNI; -2: pairwise

      seqtype = 1  * 1:codons; 2:AAs; 3:codons-->AAs
    CodonFreq = 2  * 0:1/61 each, 1:F1X4, 2:F3X4, 3:codon table
        clock = 0  * 0:no clock, 1:clock; 2:local clock; 3:CombinedAnalysis
        model = 2
                   * models for codons:
                       * 0:one, 1:b, 2:2 or more dN/dS ratios for branches

      NSsites = 0  * 0:one w; 1:neutral; 2:selection; 3:discrete; 4:freqs;
                   * 5:gamma; 6:2gamma; 7:beta; 8:beta&w 9:betaγ
                   * 10:betaγ+1; 11:beta&normal>1; 12:0&2normal>1;
                   * 13:3normal>0
        icode = 0  * 0:universal code; 1:mammalian mt; 2-10:see below

    fix_kappa = 0  * 1: kappa fixed, 0: kappa to be estimated
        kappa = 2  * initial or fixed kappa
    fix_omega = 0  * 1: omega or omega_1 fixed, 0: estimate 
        omega = 1  * initial or fixed omega, for codons or codon-based AAs

    fix_alpha = 1  * 0: estimate gamma shape parameter; 1: fix it at alpha
        alpha = .0 * initial or fixed alpha, 0:infinity (constant rate)
       Malpha = 0  * different alphas for genes
        ncatG = 4  * # of categories in dG of NSsites models

        getSE = 0  * 0: don't want them, 1: want S.E.s of estimates
 RateAncestor = 0  * (0,1,2): rates (alpha>0) or ancestral states (1 or 2)
       method = 0  * 0: simultaneous; 1: one branch at a time
```

---

**8. Control file for "Model A"**

```
      seqfile = tg.phy
     treefile = tg-human.tree
      outfile = tg-model-A.mlc

        noisy = 9  * 0,1,2,3,9: how much rubbish on the screen
      verbose = 0  * 0: concise; 1: detailed, 2: too much
      runmode = 0  * 0: user tree;  1: semi-automatic;  2: automatic
                   * 3: StepwiseAddition; (4,5):PerturbationNNI; -2: pairwise

      seqtype = 1  * 1:codons; 2:AAs; 3:codons-->AAs
    CodonFreq = 2  * 0:1/61 each, 1:F1X4, 2:F3X4, 3:codon table
        clock = 0  * 0:no clock, 1:clock; 2:local clock; 3:CombinedAnalysis
        model = 2
                   * models for codons:
                       * 0:one, 1:b, 2:2 or more dN/dS ratios for branches

      NSsites = 2  * 0:one w; 1:neutral; 2:selection; 3:discrete; 4:freqs;
                   * 5:gamma; 6:2gamma; 7:beta; 8:beta&w 9:betaγ
                   * 10:betaγ+1; 11:beta&normal>1; 12:0&2normal>1;
                   * 13:3normal>0
        icode = 0  * 0:universal code; 1:mammalian mt; 2-10:see below

    fix_kappa = 0  * 1: kappa fixed, 0: kappa to be estimated
        kappa = 2  * initial or fixed kappa
    fix_omega = 0  * 1: omega or omega_1 fixed, 0: estimate 
        omega = 1  * initial or fixed omega, for codons or codon-based AAs

    fix_alpha = 1  * 0: estimate gamma shape parameter; 1: fix it at alpha
        alpha = .0 * initial or fixed alpha, 0:infinity (constant rate)
       Malpha = 0  * different alphas for genes
        ncatG = 4  * # of categories in dG of NSsites models

        getSE = 0  * 0: don't want them, 1: want S.E.s of estimates
 RateAncestor = 0  * (0,1,2): rates (alpha>0) or ancestral states (1 or 2)
       method = 0  * 0: simultaneous; 1: one branch at a time
```

---

**9. Control file for "Null model A"**

```
      seqfile = tg.phy
     treefile = tg-human.tree
      outfile = tg-null-model-A.mlc

        noisy = 9  * 0,1,2,3,9: how much rubbish on the screen
      verbose = 0  * 0: concise; 1: detailed, 2: too much
      runmode = 0  * 0: user tree;  1: semi-automatic;  2: automatic
                   * 3: StepwiseAddition; (4,5):PerturbationNNI; -2: pairwise

      seqtype = 1  * 1:codons; 2:AAs; 3:codons-->AAs
    CodonFreq = 2  * 0:1/61 each, 1:F1X4, 2:F3X4, 3:codon table
        clock = 0  * 0:no clock, 1:clock; 2:local clock; 3:CombinedAnalysis
        model = 2
                   * models for codons:
                       * 0:one, 1:b, 2:2 or more dN/dS ratios for branches

      NSsites = 2  * 0:one w; 1:neutral; 2:selection; 3:discrete; 4:freqs;
                   * 5:gamma; 6:2gamma; 7:beta; 8:beta&w 9:betaγ
                   * 10:betaγ+1; 11:beta&normal>1; 12:0&2normal>1;
                   * 13:3normal>0
        icode = 0  * 0:universal code; 1:mammalian mt; 2-10:see below

    fix_kappa = 0  * 1: kappa fixed, 0: kappa to be estimated
        kappa = 2  * initial or fixed kappa
    fix_omega = 1  * 1: omega or omega_1 fixed, 0: estimate 
        omega = 1  * initial or fixed omega, for codons or codon-based AAs

    fix_alpha = 1  * 0: estimate gamma shape parameter; 1: fix it at alpha
        alpha = .0 * initial or fixed alpha, 0:infinity (constant rate)
       Malpha = 0  * different alphas for genes
        ncatG = 4  * # of categories in dG of NSsites models

        getSE = 0  * 0: don't want them, 1: want S.E.s of estimates
 RateAncestor = 0  * (0,1,2): rates (alpha>0) or ancestral states (1 or 2)
       method = 0  * 0: simultaneous; 1: one branch at a time
```

---

**10. Main result file for "M0"**

```
CODONML (in paml version 4.8a, July 2014)  tg.phy
Model: One dN/dS ratio for branches, 
Codon frequency model: F3x4
ns =   6  ls = 2768

Codon usage in sequences
--------------------------------------------------------------------------------------------------------------------------------------
Phe TTT  58  57  58  60  60  63 | Ser TCT  61  58  59  57  60  52 | Tyr TAT  24  25  25  24  24  26 | Cys TGT  62  62  61  61  62  61
    TTC  83  85  85  82  81  78 |     TCC  64  62  62  61  62  66 |     TAC  42  41  41  42  42  44 |     TGC  61  61  62  62  61  62
Leu TTA  12  11  10  11  11   9 |     TCA  33  33  36  35  35  36 | *** TAA   0   0   0   0   0   0 | *** TGA   0   0   0   0   0   0
    TTG  37  37  39  38  33  39 |     TCG  12  13   7  12  12  12 |     TAG   0   0   0   0   0   0 | Trp TGG  45  44  45  44  43  42
--------------------------------------------------------------------------------------------------------------------------------------
Leu CTT  41  41  43  44  47  44 | Pro CCT  56  53  54  55  53  53 | His CAT  14  13  14  16  13  10 | Arg CGT  14  16  12  13  15  13
    CTC  56  56  55  54  48  56 |     CCC  44  45  46  44  47  46 |     CAC  25  24  25  27  24  24 |     CGC  23  23  22  20  23  24
    CTA  13  12  13  13  14  11 |     CCA  58  60  57  57  56  54 | Gln CAA  54  56  57  54  60  48 |     CGA  18  16  16  18  17  19
    CTG 102 102 101 103 103 110 |     CCG  15  15  17  17  16  17 |     CAG 140 137 140 144 138 143 |     CGG  30  33  30  27  32  29
--------------------------------------------------------------------------------------------------------------------------------------
Ile ATT  29  29  29  29  32  28 | Thr ACT  23  22  23  25  22  20 | Asn AAT  41  39  39  39  36  37 | Ser AGT  41  42  41  42  43  41
    ATC  45  44  45  43  41  46 |     ACC  57  56  56  50  53  54 |     AAC  41  40  39  39  40  40 |     AGC  59  63  62  65  63  63
    ATA  10   9  10  10   9   8 |     ACA  47  47  45  47  49  46 | Lys AAA  34  34  36  38  34  35 | Arg AGA  31  33  31  30  31  35
Met ATG  38  38  34  39  35  36 |     ACG  18  19  21  16  16  15 |     AAG  57  58  57  53  59  53 |     AGG  34  31  34  33  32  38
--------------------------------------------------------------------------------------------------------------------------------------
Val GTT  27  27  29  25  26  27 | Ala GCT  60  62  62  64  64  62 | Asp GAT  58  61  59  64  54  58 | Gly GGT  21  21  23  22  26  21
    GTC  43  43  43  45  46  49 |     GCC  82  85  85  85  86  87 |     GAC  67  64  66  65  70  71 |     GGC  72  71  72  71  69  68
    GTA   9   9  10   7  10   8 |     GCA  44  45  43  45  44  50 | Glu GAA  61  62  61  60  59  61 |     GGA  62  61  62  63  64  65
    GTG  85  86  86  87  84  83 |     GCG  13  14  12  12  13  11 |     GAG 108 104 107 104 106 109 |     GGG  54  55  54  56  58  52
--------------------------------------------------------------------------------------------------------------------------------------

Codon position x base (3x4) table for each sequence.

#1: human          
position  1:    T:0.21460    C:0.25397    A:0.21857    G:0.31286
position  2:    T:0.24855    C:0.24819    A:0.27673    G:0.22652
position  3:    T:0.22760    C:0.31214    A:0.17558    G:0.28468
Average         T:0.23025    C:0.27144    A:0.22363    G:0.27469

#2: chimpanzee     
position  1:    T:0.21302    C:0.25389    A:0.21844    G:0.31465
position  2:    T:0.24810    C:0.24919    A:0.27414    G:0.22857
position  3:    T:0.22712    C:0.31212    A:0.17649    G:0.28427
Average         T:0.22942    C:0.27173    A:0.22303    G:0.27583

#3: gorilla        
position  1:    T:0.21315    C:0.25361    A:0.21749    G:0.31575
position  2:    T:0.24928    C:0.24747    A:0.27673    G:0.22652
position  3:    T:0.22796    C:0.31286    A:0.17594    G:0.28324
Average         T:0.23013    C:0.27132    A:0.22339    G:0.27517

#4: orangutan      
position  1:    T:0.21279    C:0.25506    A:0.21604    G:0.31611
position  2:    T:0.24928    C:0.24639    A:0.27782    G:0.22652
position  3:    T:0.23121    C:0.30889    A:0.17630    G:0.28360
Average         T:0.23109    C:0.27011    A:0.22339    G:0.27541

#5: gibbon         
position  1:    T:0.21186    C:0.25524    A:0.21511    G:0.31779
position  2:    T:0.24584    C:0.24873    A:0.27440    G:0.23102
position  3:    T:0.23030    C:0.30947    A:0.17824    G:0.28200
Average         T:0.22933    C:0.27115    A:0.22258    G:0.27693

#6: rhesus         
position  1:    T:0.21315    C:0.25325    A:0.21496    G:0.31864
position  2:    T:0.25108    C:0.24603    A:0.27421    G:0.22868
position  3:    T:0.22254    C:0.31720    A:0.17522    G:0.28504
Average         T:0.22893    C:0.27216    A:0.22146    G:0.27746

Sums of codon usage counts
------------------------------------------------------------------------------
Phe F TTT     356 | Ser S TCT     347 | Tyr Y TAT     148 | Cys C TGT     369
      TTC     494 |       TCC     377 |       TAC     252 |       TGC     369
Leu L TTA      64 |       TCA     208 | *** * TAA       0 | *** * TGA       0
      TTG     223 |       TCG      68 |       TAG       0 | Trp W TGG     263
------------------------------------------------------------------------------
Leu L CTT     260 | Pro P CCT     324 | His H CAT      80 | Arg R CGT      83
      CTC     325 |       CCC     272 |       CAC     149 |       CGC     135
      CTA      76 |       CCA     342 | Gln Q CAA     329 |       CGA     104
      CTG     621 |       CCG      97 |       CAG     842 |       CGG     181
------------------------------------------------------------------------------
Ile I ATT     176 | Thr T ACT     135 | Asn N AAT     231 | Ser S AGT     250
      ATC     264 |       ACC     326 |       AAC     239 |       AGC     375
      ATA      56 |       ACA     281 | Lys K AAA     211 | Arg R AGA     191
Met M ATG     220 |       ACG     105 |       AAG     337 |       AGG     202
------------------------------------------------------------------------------
Val V GTT     161 | Ala A GCT     374 | Asp D GAT     354 | Gly G GGT     134
      GTC     269 |       GCC     510 |       GAC     403 |       GGC     423
      GTA      53 |       GCA     271 | Glu E GAA     364 |       GGA     377
      GTG     511 |       GCG      75 |       GAG     638 |       GGG     329
------------------------------------------------------------------------------

(Ambiguity data are not used in the counts.)


Codon position x base (3x4) table, overall

position  1:    T:0.21309    C:0.25417    A:0.21677    G:0.31597
position  2:    T:0.24869    C:0.24767    A:0.27567    G:0.22797
position  3:    T:0.22779    C:0.31211    A:0.17629    G:0.28380
Average         T:0.22986    C:0.27132    A:0.22291    G:0.27591


Nei & Gojobori 1986. dN/dS (dN, dS)
(Pairwise deletion)
(Note: This matrix is not used in later ML. analysis.
Use runmode = -2 for ML pairwise comparison.)

human               
chimpanzee           0.3825 (0.0073 0.0192)
gorilla              0.4500 (0.0091 0.0202) 0.2820 (0.0069 0.0243)
orangutan            0.3424 (0.0158 0.0461) 0.2938 (0.0139 0.0472) 0.3069 (0.0156 0.0509)
gibbon               0.3235 (0.0174 0.0537) 0.2627 (0.0143 0.0543) 0.3144 (0.0168 0.0534) 0.2739 (0.0161 0.0586)
rhesus               0.2417 (0.0248 0.1025) 0.2324 (0.0225 0.0969) 0.2299 (0.0240 0.1043) 0.2339 (0.0244 0.1042) 0.2177 (0.0227 0.1045)


TREE #  1:  ((((1, 2), 3), 4), 5, 6);   MP score: -1
lnL(ntime:  9  np: 11): -15128.099934      +0.000000
   7..8     8..9     9..10   10..1    10..2     9..3     8..4     7..5     7..6  
 0.009225 0.017029 0.001567 0.018052 0.013121 0.019335 0.037331 0.037147 0.093304 6.351444 0.356392

Note: Branch length is defined as number of nucleotide substitutions per codon (not per neucleotide site).

tree length =   0.24611

((((1: 0.018052, 2: 0.013121): 0.001567, 3: 0.019335): 0.017029, 4: 0.037331): 0.009225, 5: 0.037147, 6: 0.093304);

((((human: 0.018052, chimpanzee: 0.013121): 0.001567, gorilla: 0.019335): 0.017029, orangutan: 0.037331): 0.009225, gibbon: 0.037147, rhesus: 0.093304);

Detailed output identifying parameters

kappa (ts/tv) =  6.35144

omega (dN/dS) =  0.35639

dN & dS for each branch

 branch          t       N       S   dN/dS      dN      dS  N*dN  S*dS

   7..8      0.009  5782.7  2521.3  0.3564  0.0020  0.0056  11.5  14.1
   8..9      0.017  5782.7  2521.3  0.3564  0.0037  0.0103  21.2  25.9
   9..10     0.002  5782.7  2521.3  0.3564  0.0003  0.0009   2.0   2.4
  10..1      0.018  5782.7  2521.3  0.3564  0.0039  0.0109  22.5  27.5
  10..2      0.013  5782.7  2521.3  0.3564  0.0028  0.0079  16.3  20.0
   9..3      0.019  5782.7  2521.3  0.3564  0.0042  0.0117  24.1  29.4
   8..4      0.037  5782.7  2521.3  0.3564  0.0080  0.0226  46.5  56.9
   7..5      0.037  5782.7  2521.3  0.3564  0.0080  0.0224  46.2  56.6
   7..6      0.093  5782.7  2521.3  0.3564  0.0201  0.0564 116.2 142.1

tree length for dN:       0.0530
tree length for dS:       0.1487


Time used:  0:05
```

---

**11. Main result file for "Free ratio"**

```
CODONML (in paml version 4.8a, July 2014)  tg.phy
Model: free dN/dS Ratios for branches for branches, 
Codon frequency model: F3x4
ns =   6  ls = 2768

Codon usage in sequences
--------------------------------------------------------------------------------------------------------------------------------------
Phe TTT  58  57  58  60  60  63 | Ser TCT  61  58  59  57  60  52 | Tyr TAT  24  25  25  24  24  26 | Cys TGT  62  62  61  61  62  61
    TTC  83  85  85  82  81  78 |     TCC  64  62  62  61  62  66 |     TAC  42  41  41  42  42  44 |     TGC  61  61  62  62  61  62
Leu TTA  12  11  10  11  11   9 |     TCA  33  33  36  35  35  36 | *** TAA   0   0   0   0   0   0 | *** TGA   0   0   0   0   0   0
    TTG  37  37  39  38  33  39 |     TCG  12  13   7  12  12  12 |     TAG   0   0   0   0   0   0 | Trp TGG  45  44  45  44  43  42
--------------------------------------------------------------------------------------------------------------------------------------
Leu CTT  41  41  43  44  47  44 | Pro CCT  56  53  54  55  53  53 | His CAT  14  13  14  16  13  10 | Arg CGT  14  16  12  13  15  13
    CTC  56  56  55  54  48  56 |     CCC  44  45  46  44  47  46 |     CAC  25  24  25  27  24  24 |     CGC  23  23  22  20  23  24
    CTA  13  12  13  13  14  11 |     CCA  58  60  57  57  56  54 | Gln CAA  54  56  57  54  60  48 |     CGA  18  16  16  18  17  19
    CTG 102 102 101 103 103 110 |     CCG  15  15  17  17  16  17 |     CAG 140 137 140 144 138 143 |     CGG  30  33  30  27  32  29
--------------------------------------------------------------------------------------------------------------------------------------
Ile ATT  29  29  29  29  32  28 | Thr ACT  23  22  23  25  22  20 | Asn AAT  41  39  39  39  36  37 | Ser AGT  41  42  41  42  43  41
    ATC  45  44  45  43  41  46 |     ACC  57  56  56  50  53  54 |     AAC  41  40  39  39  40  40 |     AGC  59  63  62  65  63  63
    ATA  10   9  10  10   9   8 |     ACA  47  47  45  47  49  46 | Lys AAA  34  34  36  38  34  35 | Arg AGA  31  33  31  30  31  35
Met ATG  38  38  34  39  35  36 |     ACG  18  19  21  16  16  15 |     AAG  57  58  57  53  59  53 |     AGG  34  31  34  33  32  38
--------------------------------------------------------------------------------------------------------------------------------------
Val GTT  27  27  29  25  26  27 | Ala GCT  60  62  62  64  64  62 | Asp GAT  58  61  59  64  54  58 | Gly GGT  21  21  23  22  26  21
    GTC  43  43  43  45  46  49 |     GCC  82  85  85  85  86  87 |     GAC  67  64  66  65  70  71 |     GGC  72  71  72  71  69  68
    GTA   9   9  10   7  10   8 |     GCA  44  45  43  45  44  50 | Glu GAA  61  62  61  60  59  61 |     GGA  62  61  62  63  64  65
    GTG  85  86  86  87  84  83 |     GCG  13  14  12  12  13  11 |     GAG 108 104 107 104 106 109 |     GGG  54  55  54  56  58  52
--------------------------------------------------------------------------------------------------------------------------------------

Codon position x base (3x4) table for each sequence.

#1: human          
position  1:    T:0.21460    C:0.25397    A:0.21857    G:0.31286
position  2:    T:0.24855    C:0.24819    A:0.27673    G:0.22652
position  3:    T:0.22760    C:0.31214    A:0.17558    G:0.28468
Average         T:0.23025    C:0.27144    A:0.22363    G:0.27469

#2: chimpanzee     
position  1:    T:0.21302    C:0.25389    A:0.21844    G:0.31465
position  2:    T:0.24810    C:0.24919    A:0.27414    G:0.22857
position  3:    T:0.22712    C:0.31212    A:0.17649    G:0.28427
Average         T:0.22942    C:0.27173    A:0.22303    G:0.27583

#3: gorilla        
position  1:    T:0.21315    C:0.25361    A:0.21749    G:0.31575
position  2:    T:0.24928    C:0.24747    A:0.27673    G:0.22652
position  3:    T:0.22796    C:0.31286    A:0.17594    G:0.28324
Average         T:0.23013    C:0.27132    A:0.22339    G:0.27517

#4: orangutan      
position  1:    T:0.21279    C:0.25506    A:0.21604    G:0.31611
position  2:    T:0.24928    C:0.24639    A:0.27782    G:0.22652
position  3:    T:0.23121    C:0.30889    A:0.17630    G:0.28360
Average         T:0.23109    C:0.27011    A:0.22339    G:0.27541

#5: gibbon         
position  1:    T:0.21186    C:0.25524    A:0.21511    G:0.31779
position  2:    T:0.24584    C:0.24873    A:0.27440    G:0.23102
position  3:    T:0.23030    C:0.30947    A:0.17824    G:0.28200
Average         T:0.22933    C:0.27115    A:0.22258    G:0.27693

#6: rhesus         
position  1:    T:0.21315    C:0.25325    A:0.21496    G:0.31864
position  2:    T:0.25108    C:0.24603    A:0.27421    G:0.22868
position  3:    T:0.22254    C:0.31720    A:0.17522    G:0.28504
Average         T:0.22893    C:0.27216    A:0.22146    G:0.27746

Sums of codon usage counts
------------------------------------------------------------------------------
Phe F TTT     356 | Ser S TCT     347 | Tyr Y TAT     148 | Cys C TGT     369
      TTC     494 |       TCC     377 |       TAC     252 |       TGC     369
Leu L TTA      64 |       TCA     208 | *** * TAA       0 | *** * TGA       0
      TTG     223 |       TCG      68 |       TAG       0 | Trp W TGG     263
------------------------------------------------------------------------------
Leu L CTT     260 | Pro P CCT     324 | His H CAT      80 | Arg R CGT      83
      CTC     325 |       CCC     272 |       CAC     149 |       CGC     135
      CTA      76 |       CCA     342 | Gln Q CAA     329 |       CGA     104
      CTG     621 |       CCG      97 |       CAG     842 |       CGG     181
------------------------------------------------------------------------------
Ile I ATT     176 | Thr T ACT     135 | Asn N AAT     231 | Ser S AGT     250
      ATC     264 |       ACC     326 |       AAC     239 |       AGC     375
      ATA      56 |       ACA     281 | Lys K AAA     211 | Arg R AGA     191
Met M ATG     220 |       ACG     105 |       AAG     337 |       AGG     202
------------------------------------------------------------------------------
Val V GTT     161 | Ala A GCT     374 | Asp D GAT     354 | Gly G GGT     134
      GTC     269 |       GCC     510 |       GAC     403 |       GGC     423
      GTA      53 |       GCA     271 | Glu E GAA     364 |       GGA     377
      GTG     511 |       GCG      75 |       GAG     638 |       GGG     329
------------------------------------------------------------------------------

(Ambiguity data are not used in the counts.)


Codon position x base (3x4) table, overall

position  1:    T:0.21309    C:0.25417    A:0.21677    G:0.31597
position  2:    T:0.24869    C:0.24767    A:0.27567    G:0.22797
position  3:    T:0.22779    C:0.31211    A:0.17629    G:0.28380
Average         T:0.22986    C:0.27132    A:0.22291    G:0.27591


Nei & Gojobori 1986. dN/dS (dN, dS)
(Pairwise deletion)
(Note: This matrix is not used in later ML. analysis.
Use runmode = -2 for ML pairwise comparison.)

human               
chimpanzee           0.3825 (0.0073 0.0192)
gorilla              0.4500 (0.0091 0.0202) 0.2820 (0.0069 0.0243)
orangutan            0.3424 (0.0158 0.0461) 0.2938 (0.0139 0.0472) 0.3069 (0.0156 0.0509)
gibbon               0.3235 (0.0174 0.0537) 0.2627 (0.0143 0.0543) 0.3144 (0.0168 0.0534) 0.2739 (0.0161 0.0586)
rhesus               0.2417 (0.0248 0.1025) 0.2324 (0.0225 0.0969) 0.2299 (0.0240 0.1043) 0.2339 (0.0244 0.1042) 0.2177 (0.0227 0.1045)


TREE #  1:  ((((1, 2), 3), 4), 5, 6);   MP score: -1
lnL(ntime:  9  np: 19): -15121.352872      +0.000000
   7..8     8..9     9..10   10..1    10..2     9..3     8..4     7..5     7..6  
 0.009140 0.017174 0.001631 0.017859 0.013280 0.019074 0.037387 0.037172 0.093843 6.356583 0.346918 0.417515 0.268215 0.791542 0.310119 0.478103 0.412931 0.350003 0.267566

Note: Branch length is defined as number of nucleotide substitutions per codon (not per neucleotide site).

tree length =   0.24656

((((1: 0.017859, 2: 0.013280): 0.001631, 3: 0.019074): 0.017174, 4: 0.037387): 0.009140, 5: 0.037172, 6: 0.093843);

((((human: 0.017859, chimpanzee: 0.013280): 0.001631, gorilla: 0.019074): 0.017174, orangutan: 0.037387): 0.009140, gibbon: 0.037172, rhesus: 0.093843);

Detailed output identifying parameters

kappa (ts/tv) =  6.35658

w (dN/dS) for branches:  0.34692 0.41752 0.26822 0.79154 0.31012 0.47810 0.41293 0.35000 0.26757

dN & dS for each branch

 branch          t       N       S   dN/dS      dN      dS  N*dN  S*dS

   7..8      0.009  5782.5  2521.5  0.3469  0.0019  0.0056  11.2  14.1
   8..9      0.017  5782.5  2521.5  0.4175  0.0040  0.0096  23.3  24.3
   9..10     0.002  5782.5  2521.5  0.2682  0.0003  0.0011   1.7   2.8
  10..1      0.018  5782.5  2521.5  0.7915  0.0055  0.0070  31.9  17.6
  10..2      0.013  5782.5  2521.5  0.3101  0.0026  0.0085  15.3  21.5
   9..3      0.019  5782.5  2521.5  0.4781  0.0048  0.0100  27.6  25.2
   8..4      0.037  5782.5  2521.5  0.4129  0.0087  0.0211  50.3  53.2
   7..5      0.037  5782.5  2521.5  0.3500  0.0079  0.0226  45.8  57.1
   7..6      0.094  5782.5  2521.5  0.2676  0.0171  0.0638  98.8 161.0

tree length for dN:       0.0529
tree length for dS:       0.1494

dS tree:
((((human: 0.006964, chimpanzee: 0.008519): 0.001109, gorilla: 0.009988): 0.009631, orangutan: 0.021080): 0.005588, gibbon: 0.022636, rhesus: 0.063843);
dN tree:
((((human: 0.005512, chimpanzee: 0.002642): 0.000297, gorilla: 0.004775): 0.004021, orangutan: 0.008705): 0.001938, gibbon: 0.007923, rhesus: 0.017082);

w ratios as labels for TreeView:
((((human #0.7915 , chimpanzee #0.3101 ) #0.2682 , gorilla #0.4781 ) #0.4175 , orangutan #0.4129 ) #0.3469 , gibbon #0.3500 , rhesus #0.2676 );


Time used:  0:26
```

---

**12. Main result file for "Two ratio"**

```
CODONML (in paml version 4.8a, July 2014)  tg.phy
Model: several dN/dS ratios for branches for branches, 
Codon frequency model: F3x4
ns =   6  ls = 2768

Codon usage in sequences
--------------------------------------------------------------------------------------------------------------------------------------
Phe TTT  58  57  58  60  60  63 | Ser TCT  61  58  59  57  60  52 | Tyr TAT  24  25  25  24  24  26 | Cys TGT  62  62  61  61  62  61
    TTC  83  85  85  82  81  78 |     TCC  64  62  62  61  62  66 |     TAC  42  41  41  42  42  44 |     TGC  61  61  62  62  61  62
Leu TTA  12  11  10  11  11   9 |     TCA  33  33  36  35  35  36 | *** TAA   0   0   0   0   0   0 | *** TGA   0   0   0   0   0   0
    TTG  37  37  39  38  33  39 |     TCG  12  13   7  12  12  12 |     TAG   0   0   0   0   0   0 | Trp TGG  45  44  45  44  43  42
--------------------------------------------------------------------------------------------------------------------------------------
Leu CTT  41  41  43  44  47  44 | Pro CCT  56  53  54  55  53  53 | His CAT  14  13  14  16  13  10 | Arg CGT  14  16  12  13  15  13
    CTC  56  56  55  54  48  56 |     CCC  44  45  46  44  47  46 |     CAC  25  24  25  27  24  24 |     CGC  23  23  22  20  23  24
    CTA  13  12  13  13  14  11 |     CCA  58  60  57  57  56  54 | Gln CAA  54  56  57  54  60  48 |     CGA  18  16  16  18  17  19
    CTG 102 102 101 103 103 110 |     CCG  15  15  17  17  16  17 |     CAG 140 137 140 144 138 143 |     CGG  30  33  30  27  32  29
--------------------------------------------------------------------------------------------------------------------------------------
Ile ATT  29  29  29  29  32  28 | Thr ACT  23  22  23  25  22  20 | Asn AAT  41  39  39  39  36  37 | Ser AGT  41  42  41  42  43  41
    ATC  45  44  45  43  41  46 |     ACC  57  56  56  50  53  54 |     AAC  41  40  39  39  40  40 |     AGC  59  63  62  65  63  63
    ATA  10   9  10  10   9   8 |     ACA  47  47  45  47  49  46 | Lys AAA  34  34  36  38  34  35 | Arg AGA  31  33  31  30  31  35
Met ATG  38  38  34  39  35  36 |     ACG  18  19  21  16  16  15 |     AAG  57  58  57  53  59  53 |     AGG  34  31  34  33  32  38
--------------------------------------------------------------------------------------------------------------------------------------
Val GTT  27  27  29  25  26  27 | Ala GCT  60  62  62  64  64  62 | Asp GAT  58  61  59  64  54  58 | Gly GGT  21  21  23  22  26  21
    GTC  43  43  43  45  46  49 |     GCC  82  85  85  85  86  87 |     GAC  67  64  66  65  70  71 |     GGC  72  71  72  71  69  68
    GTA   9   9  10   7  10   8 |     GCA  44  45  43  45  44  50 | Glu GAA  61  62  61  60  59  61 |     GGA  62  61  62  63  64  65
    GTG  85  86  86  87  84  83 |     GCG  13  14  12  12  13  11 |     GAG 108 104 107 104 106 109 |     GGG  54  55  54  56  58  52
--------------------------------------------------------------------------------------------------------------------------------------

Codon position x base (3x4) table for each sequence.

#1: human          
position  1:    T:0.21460    C:0.25397    A:0.21857    G:0.31286
position  2:    T:0.24855    C:0.24819    A:0.27673    G:0.22652
position  3:    T:0.22760    C:0.31214    A:0.17558    G:0.28468
Average         T:0.23025    C:0.27144    A:0.22363    G:0.27469

#2: chimpanzee     
position  1:    T:0.21302    C:0.25389    A:0.21844    G:0.31465
position  2:    T:0.24810    C:0.24919    A:0.27414    G:0.22857
position  3:    T:0.22712    C:0.31212    A:0.17649    G:0.28427
Average         T:0.22942    C:0.27173    A:0.22303    G:0.27583

#3: gorilla        
position  1:    T:0.21315    C:0.25361    A:0.21749    G:0.31575
position  2:    T:0.24928    C:0.24747    A:0.27673    G:0.22652
position  3:    T:0.22796    C:0.31286    A:0.17594    G:0.28324
Average         T:0.23013    C:0.27132    A:0.22339    G:0.27517

#4: orangutan      
position  1:    T:0.21279    C:0.25506    A:0.21604    G:0.31611
position  2:    T:0.24928    C:0.24639    A:0.27782    G:0.22652
position  3:    T:0.23121    C:0.30889    A:0.17630    G:0.28360
Average         T:0.23109    C:0.27011    A:0.22339    G:0.27541

#5: gibbon         
position  1:    T:0.21186    C:0.25524    A:0.21511    G:0.31779
position  2:    T:0.24584    C:0.24873    A:0.27440    G:0.23102
position  3:    T:0.23030    C:0.30947    A:0.17824    G:0.28200
Average         T:0.22933    C:0.27115    A:0.22258    G:0.27693

#6: rhesus         
position  1:    T:0.21315    C:0.25325    A:0.21496    G:0.31864
position  2:    T:0.25108    C:0.24603    A:0.27421    G:0.22868
position  3:    T:0.22254    C:0.31720    A:0.17522    G:0.28504
Average         T:0.22893    C:0.27216    A:0.22146    G:0.27746

Sums of codon usage counts
------------------------------------------------------------------------------
Phe F TTT     356 | Ser S TCT     347 | Tyr Y TAT     148 | Cys C TGT     369
      TTC     494 |       TCC     377 |       TAC     252 |       TGC     369
Leu L TTA      64 |       TCA     208 | *** * TAA       0 | *** * TGA       0
      TTG     223 |       TCG      68 |       TAG       0 | Trp W TGG     263
------------------------------------------------------------------------------
Leu L CTT     260 | Pro P CCT     324 | His H CAT      80 | Arg R CGT      83
      CTC     325 |       CCC     272 |       CAC     149 |       CGC     135
      CTA      76 |       CCA     342 | Gln Q CAA     329 |       CGA     104
      CTG     621 |       CCG      97 |       CAG     842 |       CGG     181
------------------------------------------------------------------------------
Ile I ATT     176 | Thr T ACT     135 | Asn N AAT     231 | Ser S AGT     250
      ATC     264 |       ACC     326 |       AAC     239 |       AGC     375
      ATA      56 |       ACA     281 | Lys K AAA     211 | Arg R AGA     191
Met M ATG     220 |       ACG     105 |       AAG     337 |       AGG     202
------------------------------------------------------------------------------
Val V GTT     161 | Ala A GCT     374 | Asp D GAT     354 | Gly G GGT     134
      GTC     269 |       GCC     510 |       GAC     403 |       GGC     423
      GTA      53 |       GCA     271 | Glu E GAA     364 |       GGA     377
      GTG     511 |       GCG      75 |       GAG     638 |       GGG     329
------------------------------------------------------------------------------

(Ambiguity data are not used in the counts.)


Codon position x base (3x4) table, overall

position  1:    T:0.21309    C:0.25417    A:0.21677    G:0.31597
position  2:    T:0.24869    C:0.24767    A:0.27567    G:0.22797
position  3:    T:0.22779    C:0.31211    A:0.17629    G:0.28380
Average         T:0.22986    C:0.27132    A:0.22291    G:0.27591


Nei & Gojobori 1986. dN/dS (dN, dS)
(Pairwise deletion)
(Note: This matrix is not used in later ML. analysis.
Use runmode = -2 for ML pairwise comparison.)

human               
chimpanzee           0.3825 (0.0073 0.0192)
gorilla              0.4500 (0.0091 0.0202) 0.2820 (0.0069 0.0243)
orangutan            0.3424 (0.0158 0.0461) 0.2938 (0.0139 0.0472) 0.3069 (0.0156 0.0509)
gibbon               0.3235 (0.0174 0.0537) 0.2627 (0.0143 0.0543) 0.3144 (0.0168 0.0534) 0.2739 (0.0161 0.0586)
rhesus               0.2417 (0.0248 0.1025) 0.2324 (0.0225 0.0969) 0.2299 (0.0240 0.1043) 0.2339 (0.0244 0.1042) 0.2177 (0.0227 0.1045)


TREE #  1:  ((((1, 2), 3), 4), 5, 6);   MP score: -1
check convergence..
lnL(ntime:  9  np: 12): -15124.398270      +0.000000
   7..8     8..9     9..10   10..1    10..2     9..3     8..4     7..5     7..6  
 0.009212 0.017234 0.001579 0.017881 0.013256 0.019156 0.037336 0.037205 0.093376 6.353014 0.334538 0.784733

Note: Branch length is defined as number of nucleotide substitutions per codon (not per neucleotide site).

tree length =   0.24624

((((1: 0.017881, 2: 0.013256): 0.001579, 3: 0.019156): 0.017234, 4: 0.037336): 0.009212, 5: 0.037205, 6: 0.093376);

((((human: 0.017881, chimpanzee: 0.013256): 0.001579, gorilla: 0.019156): 0.017234, orangutan: 0.037336): 0.009212, gibbon: 0.037205, rhesus: 0.093376);

Detailed output identifying parameters

kappa (ts/tv) =  6.35301

w (dN/dS) for branches:  0.33454 0.78473

dN & dS for each branch

 branch          t       N       S   dN/dS      dN      dS  N*dN  S*dS

   7..8      0.009  5782.6  2521.4  0.3345  0.0019  0.0057  11.1  14.4
   8..9      0.017  5782.6  2521.4  0.3345  0.0036  0.0107  20.7  27.0
   9..10     0.002  5782.6  2521.4  0.3345  0.0003  0.0010   1.9   2.5
  10..1      0.018  5782.6  2521.4  0.7847  0.0055  0.0070  31.8  17.7
  10..2      0.013  5782.6  2521.4  0.3345  0.0028  0.0082  15.9  20.8
   9..3      0.019  5782.6  2521.4  0.3345  0.0040  0.0119  23.0  30.0
   8..4      0.037  5782.6  2521.4  0.3345  0.0078  0.0232  44.9  58.5
   7..5      0.037  5782.6  2521.4  0.3345  0.0077  0.0231  44.7  58.3
   7..6      0.093  5782.6  2521.4  0.3345  0.0194  0.0580 112.2 146.3

tree length for dN:       0.0530
tree length for dS:       0.1489

dS tree:
((((human: 0.007011, chimpanzee: 0.008235): 0.000981, gorilla: 0.011900): 0.010706, orangutan: 0.023193): 0.005723, gibbon: 0.023112, rhesus: 0.058005);
dN tree:
((((human: 0.005502, chimpanzee: 0.002755): 0.000328, gorilla: 0.003981): 0.003582, orangutan: 0.007759): 0.001914, gibbon: 0.007732, rhesus: 0.019405);

w ratios as labels for TreeView:
((((human #0.7847 , chimpanzee #0.3345 ) #0.3345 , gorilla #0.3345 ) #0.3345 , orangutan #0.3345 ) #0.3345 , gibbon #0.3345 , rhesus #0.3345 );


Time used:  0:09
```

---

**13. Main result file for "Model A"**

```
CODONML (in paml version 4.8a, July 2014)  tg.phy
Model: several dN/dS ratios for branches for branches, 
Codon frequency model: F3x4
Site-class models:  PositiveSelection
ns =   6  ls = 2768

Codon usage in sequences
--------------------------------------------------------------------------------------------------------------------------------------
Phe TTT  58  57  58  60  60  63 | Ser TCT  61  58  59  57  60  52 | Tyr TAT  24  25  25  24  24  26 | Cys TGT  62  62  61  61  62  61
    TTC  83  85  85  82  81  78 |     TCC  64  62  62  61  62  66 |     TAC  42  41  41  42  42  44 |     TGC  61  61  62  62  61  62
Leu TTA  12  11  10  11  11   9 |     TCA  33  33  36  35  35  36 | *** TAA   0   0   0   0   0   0 | *** TGA   0   0   0   0   0   0
    TTG  37  37  39  38  33  39 |     TCG  12  13   7  12  12  12 |     TAG   0   0   0   0   0   0 | Trp TGG  45  44  45  44  43  42
--------------------------------------------------------------------------------------------------------------------------------------
Leu CTT  41  41  43  44  47  44 | Pro CCT  56  53  54  55  53  53 | His CAT  14  13  14  16  13  10 | Arg CGT  14  16  12  13  15  13
    CTC  56  56  55  54  48  56 |     CCC  44  45  46  44  47  46 |     CAC  25  24  25  27  24  24 |     CGC  23  23  22  20  23  24
    CTA  13  12  13  13  14  11 |     CCA  58  60  57  57  56  54 | Gln CAA  54  56  57  54  60  48 |     CGA  18  16  16  18  17  19
    CTG 102 102 101 103 103 110 |     CCG  15  15  17  17  16  17 |     CAG 140 137 140 144 138 143 |     CGG  30  33  30  27  32  29
--------------------------------------------------------------------------------------------------------------------------------------
Ile ATT  29  29  29  29  32  28 | Thr ACT  23  22  23  25  22  20 | Asn AAT  41  39  39  39  36  37 | Ser AGT  41  42  41  42  43  41
    ATC  45  44  45  43  41  46 |     ACC  57  56  56  50  53  54 |     AAC  41  40  39  39  40  40 |     AGC  59  63  62  65  63  63
    ATA  10   9  10  10   9   8 |     ACA  47  47  45  47  49  46 | Lys AAA  34  34  36  38  34  35 | Arg AGA  31  33  31  30  31  35
Met ATG  38  38  34  39  35  36 |     ACG  18  19  21  16  16  15 |     AAG  57  58  57  53  59  53 |     AGG  34  31  34  33  32  38
--------------------------------------------------------------------------------------------------------------------------------------
Val GTT  27  27  29  25  26  27 | Ala GCT  60  62  62  64  64  62 | Asp GAT  58  61  59  64  54  58 | Gly GGT  21  21  23  22  26  21
    GTC  43  43  43  45  46  49 |     GCC  82  85  85  85  86  87 |     GAC  67  64  66  65  70  71 |     GGC  72  71  72  71  69  68
    GTA   9   9  10   7  10   8 |     GCA  44  45  43  45  44  50 | Glu GAA  61  62  61  60  59  61 |     GGA  62  61  62  63  64  65
    GTG  85  86  86  87  84  83 |     GCG  13  14  12  12  13  11 |     GAG 108 104 107 104 106 109 |     GGG  54  55  54  56  58  52
--------------------------------------------------------------------------------------------------------------------------------------

Codon position x base (3x4) table for each sequence.

#1: human          
position  1:    T:0.21460    C:0.25397    A:0.21857    G:0.31286
position  2:    T:0.24855    C:0.24819    A:0.27673    G:0.22652
position  3:    T:0.22760    C:0.31214    A:0.17558    G:0.28468
Average         T:0.23025    C:0.27144    A:0.22363    G:0.27469

#2: chimpanzee     
position  1:    T:0.21302    C:0.25389    A:0.21844    G:0.31465
position  2:    T:0.24810    C:0.24919    A:0.27414    G:0.22857
position  3:    T:0.22712    C:0.31212    A:0.17649    G:0.28427
Average         T:0.22942    C:0.27173    A:0.22303    G:0.27583

#3: gorilla        
position  1:    T:0.21315    C:0.25361    A:0.21749    G:0.31575
position  2:    T:0.24928    C:0.24747    A:0.27673    G:0.22652
position  3:    T:0.22796    C:0.31286    A:0.17594    G:0.28324
Average         T:0.23013    C:0.27132    A:0.22339    G:0.27517

#4: orangutan      
position  1:    T:0.21279    C:0.25506    A:0.21604    G:0.31611
position  2:    T:0.24928    C:0.24639    A:0.27782    G:0.22652
position  3:    T:0.23121    C:0.30889    A:0.17630    G:0.28360
Average         T:0.23109    C:0.27011    A:0.22339    G:0.27541

#5: gibbon         
position  1:    T:0.21186    C:0.25524    A:0.21511    G:0.31779
position  2:    T:0.24584    C:0.24873    A:0.27440    G:0.23102
position  3:    T:0.23030    C:0.30947    A:0.17824    G:0.28200
Average         T:0.22933    C:0.27115    A:0.22258    G:0.27693

#6: rhesus         
position  1:    T:0.21315    C:0.25325    A:0.21496    G:0.31864
position  2:    T:0.25108    C:0.24603    A:0.27421    G:0.22868
position  3:    T:0.22254    C:0.31720    A:0.17522    G:0.28504
Average         T:0.22893    C:0.27216    A:0.22146    G:0.27746

Sums of codon usage counts
------------------------------------------------------------------------------
Phe F TTT     356 | Ser S TCT     347 | Tyr Y TAT     148 | Cys C TGT     369
      TTC     494 |       TCC     377 |       TAC     252 |       TGC     369
Leu L TTA      64 |       TCA     208 | *** * TAA       0 | *** * TGA       0
      TTG     223 |       TCG      68 |       TAG       0 | Trp W TGG     263
------------------------------------------------------------------------------
Leu L CTT     260 | Pro P CCT     324 | His H CAT      80 | Arg R CGT      83
      CTC     325 |       CCC     272 |       CAC     149 |       CGC     135
      CTA      76 |       CCA     342 | Gln Q CAA     329 |       CGA     104
      CTG     621 |       CCG      97 |       CAG     842 |       CGG     181
------------------------------------------------------------------------------
Ile I ATT     176 | Thr T ACT     135 | Asn N AAT     231 | Ser S AGT     250
      ATC     264 |       ACC     326 |       AAC     239 |       AGC     375
      ATA      56 |       ACA     281 | Lys K AAA     211 | Arg R AGA     191
Met M ATG     220 |       ACG     105 |       AAG     337 |       AGG     202
------------------------------------------------------------------------------
Val V GTT     161 | Ala A GCT     374 | Asp D GAT     354 | Gly G GGT     134
      GTC     269 |       GCC     510 |       GAC     403 |       GGC     423
      GTA      53 |       GCA     271 | Glu E GAA     364 |       GGA     377
      GTG     511 |       GCG      75 |       GAG     638 |       GGG     329
------------------------------------------------------------------------------

(Ambiguity data are not used in the counts.)


Codon position x base (3x4) table, overall

position  1:    T:0.21309    C:0.25417    A:0.21677    G:0.31597
position  2:    T:0.24869    C:0.24767    A:0.27567    G:0.22797
position  3:    T:0.22779    C:0.31211    A:0.17629    G:0.28380
Average         T:0.22986    C:0.27132    A:0.22291    G:0.27591


Nei & Gojobori 1986. dN/dS (dN, dS)
(Pairwise deletion)
(Note: This matrix is not used in later ML. analysis.
Use runmode = -2 for ML pairwise comparison.)

human               
chimpanzee           0.3825 (0.0073 0.0192)
gorilla              0.4500 (0.0091 0.0202) 0.2820 (0.0069 0.0243)
orangutan            0.3424 (0.0158 0.0461) 0.2938 (0.0139 0.0472) 0.3069 (0.0156 0.0509)
gibbon               0.3235 (0.0174 0.0537) 0.2627 (0.0143 0.0543) 0.3144 (0.0168 0.0534) 0.2739 (0.0161 0.0586)
rhesus               0.2417 (0.0248 0.1025) 0.2324 (0.0225 0.0969) 0.2299 (0.0240 0.1043) 0.2339 (0.0244 0.1042) 0.2177 (0.0227 0.1045)


TREE #  1:  ((((1, 2), 3), 4), 5, 6);   MP score: -1
lnL(ntime:  9  np: 14): -15103.554824      +0.000000
   7..8     8..9     9..10   10..1    10..2     9..3     8..4     7..5     7..6  
 0.008981 0.017475 0.001442 0.017991 0.013399 0.019408 0.037709 0.037642 0.095577 6.382618 0.602346 0.306839 0.000001 3.593821

Note: Branch length is defined as number of nucleotide substitutions per codon (not per neucleotide site).

tree length =   0.24962

((((1: 0.017991, 2: 0.013399): 0.001442, 3: 0.019408): 0.017475, 4: 0.037709): 0.008981, 5: 0.037642, 6: 0.095577);

((((human: 0.017991, chimpanzee: 0.013399): 0.001442, gorilla: 0.019408): 0.017475, orangutan: 0.037709): 0.008981, gibbon: 0.037642, rhesus: 0.095577);

Detailed output identifying parameters

kappa (ts/tv) =  6.38262


dN/dS (w) for site classes (K=4)

site class             0        1       2a       2b
proportion       0.60235  0.30684  0.06017  0.03065
background w     0.00000  1.00000  0.00000  1.00000
foreground w     0.00000  1.00000  3.59382  3.59382


Naive Empirical Bayes (NEB) analysis (please use the BEB results.)
Positive sites for foreground lineages Prob(w>1):

    76 N 0.566
   633 S 0.571
   734 S 0.556
   775 N 0.559
   911 M 0.558
   913 S 0.556
  1061 G 0.572
  1140 S 0.571
  1204 T 0.566
  1242 M 0.566
  1498 T 0.575
  1646 R 0.538
  1669 H 0.548
  1691 R 0.572
  1795 D 0.566
  2486 H 0.545
  2530 R 0.538
  2616 N 0.567
  2632 L 0.553
  2702 E 0.557
  2727 T 0.546
  2765 T 0.572

Bayes Empirical Bayes (BEB) analysis (Yang, Wong & Nielsen 2005. Mol. Biol. Evol. 22:1107-1118)
Positive sites for foreground lineages Prob(w>1):
    76 N 0.570
   633 S 0.577
   734 S 0.567
   775 N 0.566
   911 M 0.564
   913 S 0.567
  1061 G 0.578
  1140 S 0.574
  1204 T 0.570
  1242 M 0.570
  1498 T 0.577
  1646 R 0.553
  1669 H 0.558
  1691 R 0.578
  1795 D 0.570
  2486 H 0.555
  2530 R 0.553
  2616 N 0.571
  2632 L 0.563
  2702 E 0.567
  2727 T 0.558
  2765 T 0.577


The grid (see ternary graph for p0-p1)

w0:   0.050  0.150  0.250  0.350  0.450  0.550  0.650  0.750  0.850  0.950
w2:   1.500  2.500  3.500  4.500  5.500  6.500  7.500  8.500  9.500 10.500


Posterior on the grid

w0:   0.987  0.013  0.000  0.000  0.000  0.000  0.000  0.000  0.000  0.000
w2:   0.211  0.178  0.129  0.120  0.112  0.091  0.065  0.044  0.030  0.021

Posterior for p0-p1 (see the ternary graph)

 0.000
 0.000 0.000 0.000
 0.000 0.000 0.000 0.000 0.000
 0.000 0.000 0.000 0.000 0.000 0.000 0.000
 0.000 0.000 0.000 0.000 0.000 0.000 0.000 0.000 0.000
 0.000 0.000 0.000 0.000 0.000 0.000 0.000 0.000 0.000 0.000 0.000
 0.000 0.000 0.000 0.000 0.000 0.000 0.000 0.000 0.000 0.000 0.000 0.000 0.107
 0.000 0.000 0.000 0.000 0.000 0.000 0.000 0.000 0.005 0.002 0.128 0.137 0.106 0.467 0.015
 0.000 0.000 0.000 0.000 0.000 0.000 0.003 0.009 0.001 0.013 0.001 0.002 0.000 0.002 0.000 0.002 0.000
 0.000 0.000 0.000 0.000 0.000 0.000 0.000 0.000 0.000 0.000 0.000 0.000 0.000 0.000 0.000 0.000 0.000 0.000 0.000

sum of density on p0-p1 =   1.000000

Time used:  0:52
```

---

**14. Main result file for "Null model A"**

```
CODONML (in paml version 4.8a, July 2014)  tg.phy
Model: several dN/dS ratios for branches for branches,  omega = 1.000 fixed

Codon frequency model: F3x4
Site-class models:  PositiveSelection
ns =   6  ls = 2768

Codon usage in sequences
--------------------------------------------------------------------------------------------------------------------------------------
Phe TTT  58  57  58  60  60  63 | Ser TCT  61  58  59  57  60  52 | Tyr TAT  24  25  25  24  24  26 | Cys TGT  62  62  61  61  62  61
    TTC  83  85  85  82  81  78 |     TCC  64  62  62  61  62  66 |     TAC  42  41  41  42  42  44 |     TGC  61  61  62  62  61  62
Leu TTA  12  11  10  11  11   9 |     TCA  33  33  36  35  35  36 | *** TAA   0   0   0   0   0   0 | *** TGA   0   0   0   0   0   0
    TTG  37  37  39  38  33  39 |     TCG  12  13   7  12  12  12 |     TAG   0   0   0   0   0   0 | Trp TGG  45  44  45  44  43  42
--------------------------------------------------------------------------------------------------------------------------------------
Leu CTT  41  41  43  44  47  44 | Pro CCT  56  53  54  55  53  53 | His CAT  14  13  14  16  13  10 | Arg CGT  14  16  12  13  15  13
    CTC  56  56  55  54  48  56 |     CCC  44  45  46  44  47  46 |     CAC  25  24  25  27  24  24 |     CGC  23  23  22  20  23  24
    CTA  13  12  13  13  14  11 |     CCA  58  60  57  57  56  54 | Gln CAA  54  56  57  54  60  48 |     CGA  18  16  16  18  17  19
    CTG 102 102 101 103 103 110 |     CCG  15  15  17  17  16  17 |     CAG 140 137 140 144 138 143 |     CGG  30  33  30  27  32  29
--------------------------------------------------------------------------------------------------------------------------------------
Ile ATT  29  29  29  29  32  28 | Thr ACT  23  22  23  25  22  20 | Asn AAT  41  39  39  39  36  37 | Ser AGT  41  42  41  42  43  41
    ATC  45  44  45  43  41  46 |     ACC  57  56  56  50  53  54 |     AAC  41  40  39  39  40  40 |     AGC  59  63  62  65  63  63
    ATA  10   9  10  10   9   8 |     ACA  47  47  45  47  49  46 | Lys AAA  34  34  36  38  34  35 | Arg AGA  31  33  31  30  31  35
Met ATG  38  38  34  39  35  36 |     ACG  18  19  21  16  16  15 |     AAG  57  58  57  53  59  53 |     AGG  34  31  34  33  32  38
--------------------------------------------------------------------------------------------------------------------------------------
Val GTT  27  27  29  25  26  27 | Ala GCT  60  62  62  64  64  62 | Asp GAT  58  61  59  64  54  58 | Gly GGT  21  21  23  22  26  21
    GTC  43  43  43  45  46  49 |     GCC  82  85  85  85  86  87 |     GAC  67  64  66  65  70  71 |     GGC  72  71  72  71  69  68
    GTA   9   9  10   7  10   8 |     GCA  44  45  43  45  44  50 | Glu GAA  61  62  61  60  59  61 |     GGA  62  61  62  63  64  65
    GTG  85  86  86  87  84  83 |     GCG  13  14  12  12  13  11 |     GAG 108 104 107 104 106 109 |     GGG  54  55  54  56  58  52
--------------------------------------------------------------------------------------------------------------------------------------

Codon position x base (3x4) table for each sequence.

#1: human          
position  1:    T:0.21460    C:0.25397    A:0.21857    G:0.31286
position  2:    T:0.24855    C:0.24819    A:0.27673    G:0.22652
position  3:    T:0.22760    C:0.31214    A:0.17558    G:0.28468
Average         T:0.23025    C:0.27144    A:0.22363    G:0.27469

#2: chimpanzee     
position  1:    T:0.21302    C:0.25389    A:0.21844    G:0.31465
position  2:    T:0.24810    C:0.24919    A:0.27414    G:0.22857
position  3:    T:0.22712    C:0.31212    A:0.17649    G:0.28427
Average         T:0.22942    C:0.27173    A:0.22303    G:0.27583

#3: gorilla        
position  1:    T:0.21315    C:0.25361    A:0.21749    G:0.31575
position  2:    T:0.24928    C:0.24747    A:0.27673    G:0.22652
position  3:    T:0.22796    C:0.31286    A:0.17594    G:0.28324
Average         T:0.23013    C:0.27132    A:0.22339    G:0.27517

#4: orangutan      
position  1:    T:0.21279    C:0.25506    A:0.21604    G:0.31611
position  2:    T:0.24928    C:0.24639    A:0.27782    G:0.22652
position  3:    T:0.23121    C:0.30889    A:0.17630    G:0.28360
Average         T:0.23109    C:0.27011    A:0.22339    G:0.27541

#5: gibbon         
position  1:    T:0.21186    C:0.25524    A:0.21511    G:0.31779
position  2:    T:0.24584    C:0.24873    A:0.27440    G:0.23102
position  3:    T:0.23030    C:0.30947    A:0.17824    G:0.28200
Average         T:0.22933    C:0.27115    A:0.22258    G:0.27693

#6: rhesus         
position  1:    T:0.21315    C:0.25325    A:0.21496    G:0.31864
position  2:    T:0.25108    C:0.24603    A:0.27421    G:0.22868
position  3:    T:0.22254    C:0.31720    A:0.17522    G:0.28504
Average         T:0.22893    C:0.27216    A:0.22146    G:0.27746

Sums of codon usage counts
------------------------------------------------------------------------------
Phe F TTT     356 | Ser S TCT     347 | Tyr Y TAT     148 | Cys C TGT     369
      TTC     494 |       TCC     377 |       TAC     252 |       TGC     369
Leu L TTA      64 |       TCA     208 | *** * TAA       0 | *** * TGA       0
      TTG     223 |       TCG      68 |       TAG       0 | Trp W TGG     263
------------------------------------------------------------------------------
Leu L CTT     260 | Pro P CCT     324 | His H CAT      80 | Arg R CGT      83
      CTC     325 |       CCC     272 |       CAC     149 |       CGC     135
      CTA      76 |       CCA     342 | Gln Q CAA     329 |       CGA     104
      CTG     621 |       CCG      97 |       CAG     842 |       CGG     181
------------------------------------------------------------------------------
Ile I ATT     176 | Thr T ACT     135 | Asn N AAT     231 | Ser S AGT     250
      ATC     264 |       ACC     326 |       AAC     239 |       AGC     375
      ATA      56 |       ACA     281 | Lys K AAA     211 | Arg R AGA     191
Met M ATG     220 |       ACG     105 |       AAG     337 |       AGG     202
------------------------------------------------------------------------------
Val V GTT     161 | Ala A GCT     374 | Asp D GAT     354 | Gly G GGT     134
      GTC     269 |       GCC     510 |       GAC     403 |       GGC     423
      GTA      53 |       GCA     271 | Glu E GAA     364 |       GGA     377
      GTG     511 |       GCG      75 |       GAG     638 |       GGG     329
------------------------------------------------------------------------------

(Ambiguity data are not used in the counts.)


Codon position x base (3x4) table, overall

position  1:    T:0.21309    C:0.25417    A:0.21677    G:0.31597
position  2:    T:0.24869    C:0.24767    A:0.27567    G:0.22797
position  3:    T:0.22779    C:0.31211    A:0.17629    G:0.28380
Average         T:0.22986    C:0.27132    A:0.22291    G:0.27591


Nei & Gojobori 1986. dN/dS (dN, dS)
(Pairwise deletion)
(Note: This matrix is not used in later ML. analysis.
Use runmode = -2 for ML pairwise comparison.)

human               
chimpanzee           0.3825 (0.0073 0.0192)
gorilla              0.4500 (0.0091 0.0202) 0.2820 (0.0069 0.0243)
orangutan            0.3424 (0.0158 0.0461) 0.2938 (0.0139 0.0472) 0.3069 (0.0156 0.0509)
gibbon               0.3235 (0.0174 0.0537) 0.2627 (0.0143 0.0543) 0.3144 (0.0168 0.0534) 0.2739 (0.0161 0.0586)
rhesus               0.2417 (0.0248 0.1025) 0.2324 (0.0225 0.0969) 0.2299 (0.0240 0.1043) 0.2339 (0.0244 0.1042) 0.2177 (0.0227 0.1045)


TREE #  1:  ((((1, 2), 3), 4), 5, 6);   MP score: -1
lnL(ntime:  9  np: 13): -15104.130848      +0.000000
   7..8     8..9     9..10   10..1    10..2     9..3     8..4     7..5     7..6  
 0.008984 0.017437 0.001441 0.017916 0.013383 0.019439 0.037707 0.037633 0.095572 6.367275 0.466347 0.238725 0.000001

Note: Branch length is defined as number of nucleotide substitutions per codon (not per neucleotide site).

tree length =   0.24951

((((1: 0.017916, 2: 0.013383): 0.001441, 3: 0.019439): 0.017437, 4: 0.037707): 0.008984, 5: 0.037633, 6: 0.095572);

((((human: 0.017916, chimpanzee: 0.013383): 0.001441, gorilla: 0.019439): 0.017437, orangutan: 0.037707): 0.008984, gibbon: 0.037633, rhesus: 0.095572);

Detailed output identifying parameters

kappa (ts/tv) =  6.36727


dN/dS (w) for site classes (K=4)

site class             0        1       2a       2b
proportion       0.46635  0.23873  0.19507  0.09986
background w     0.00000  1.00000  0.00000  1.00000
foreground w     0.00000  1.00000  1.00000  1.00000


Time used:  0:32
```

---
